# Assembly and Analysis of the Complete Mitochondrial Genome of *Eryngium foetidum* L. (Apiaceae)

**DOI:** 10.3390/biology14091296

**Published:** 2025-09-19

**Authors:** Lihong Zhang, Wenhu Zhang, Yongjian Luo, Jun Liu, Qing Li, Qiongheng Liu

**Affiliations:** 1 Maoming Agricultural Science and Technology Extension Center, Maoming 525000, China; 2 Guangdong Key Laboratory for Crop Germplasm Resources Preservation and Utilization, Agro-Biological Gene Research Center, Guangdong Academy of Agricultural Sciences, Guangzhou 510640, China; zhanglihong200008@126.com (L.Z.); zhangwenhu@agrogene.ac.cn (W.Z.); yongjianluo1996@outlook.com (Y.L.); liujun@gdaas.cn (J.L.)

**Keywords:** mitochondrial genome, homologous fragments, Apiaceae, SSR, RNA editing, RSCU

## Abstract

*Eryngium foetidum* L. belongs to the Apiaceae family and is a perennial herb. The entire plant is rich in essential oils, which have a distinctive aroma similar to cilantro. This plant exhibits significant biological activity and possesses characteristics such as disease resistance and antimicrobial properties, showing great potential in medical and food applications. Additionally, its essential oil has substantial commercial value. Mitochondria play a crucial role as organelles within plant cells; however, the mitochondrial genome of *E. foetidum* remains underexplored. To fill this research gap, we conducted sequencing and assembly of the mitochondrial genome of *E. foetidum*, aiming to uncover its genetic mechanisms and evolutionary trajectories. Our investigation reveals that the mitochondrial genome of *E. foetidum* is a circular structure, similar to that of other species, with a length of 241,660 bp and a GC content of 45.35%, which is within the range observed in other organisms. This genome encodes 59 genes, comprising 37 protein-coding sequences, 18 tRNA genes, and 4 rRNA genes. Comparative analysis highlighted 16 homologous regions between the mitochondrial and chloroplast genomes, with the longest segment spanning 992 bp. By analyzing 37 protein-coding genes (PCGs), we identified 479 potential RNA editing sites, which induce the formation of stop codons in the *nad3* and *atp6* genes, as well as start codons in the *ccmFC*, *atp8*, *nad4L*, *cox2*, *cox1*, and *nad7* genes. Meanwhile, the genome shows a preference for A/T bases and A/T-ending codons, with 32 codons having a relative synonymous codon usage (RSCU) value greater than 1. The codon usage bias is relatively weak and mainly influenced by natural selection. Most PCGs are under purifying selection (Ka/Ks < 1), while only a few genes, such as *rps7* and *matR,* may be under positive selection. Phylogenetic analysis of mitochondrial PCGs from 21 species showed *E. foetidum* at the basal node of Apiaceae, consistent with the latest APG angiosperm classification and chloroplast genome-based phylogenetic relationships. In summary, our comprehensive characterization of the *E. foetidum* mitochondrial genome not only provides novel insights into its evolutionary history and genetic regulation but also establishes a critical genomic resource for future molecular breeding efforts targeting mitochondrial-associated traits in this economically important species.

## Simple Summary

*Eryngium foetidum* L., a tropical herb renowned for its culinary and medicinal uses, belongs to the Apiaceae family. While its essential oils and bioactive compounds are well-studied, genetic information about this species remains limited. Here, we present the first complete mitochondrial genome of *E. foetidum*, sequenced using PacBio HiFi technology. The circular genome measures 241,660 bp with a GC content of 45.35% and contains 59 genes, including 37 protein-coding genes. Our analysis identified 479 predicted RNA editing sites and 16 chloroplast-derived sequences, highlighting dynamic genetic exchanges. Phylogenetic analysis positioned *E. foetidum* at the base of the Apiaceae family, consistent with current angiosperm classifications. These findings provide crucial insights into the plant’s evolutionary history and establish a foundation for future research on mitochondrial genetics, species identification, and molecular breeding of this economically important species.

## 1. Introduction

Mitochondria are semi-independent organelles, and their genomes are found in almost all eukaryotic cells [1]. Studies suggest that plant mitochondrial genomes typically possess greater length and structural complexity than those of animals [2]. Even within a single plant genus, mitochondrial genomes can exhibit considerable species-specific variations, including differences in genome length, arrangement of genes, and number of genes [3]. In certain plants, mitochondrial genomes exhibit linear conformations, while in others they can display branched or multichromosomal architectures [4]. This results from the fact that mitochondrial genome structures are subject to various alterations, such as frequent recombination of repetitive sequences, gene loss, and the transfer of genes from mitochondria to chloroplast or nuclear genomes. These genomic rearrangements and repetitive sequences are integral to the evolutionary dynamics of plant mitochondrial genomes [5,6]. Furthermore, rearrangements of the mitochondrial genome may lead to the generation and accumulation of novel chimeric open reading frame (ORF) genes, which in turn disrupt the normal function of mitochondrial genes, ultimately inducing male sterility and potentially resulting in cytoplasmic male sterility (CMS) [7,8]. CMS-based hybridization techniques have been widely employed in the cultivation of progeny, significantly enhancing crop yield, stress resistance, and adaptability [9]. These methods are considered promising approaches for maintaining agricultural productivity [10]. Therefore, mitochondrial genomes provide crucial genetic information for research on plant evolution, cytoplasmic inheritance, and the utilization of crop heterosis.

In the NCBI database, the number of complete plant plastid genomes is close to 13,000, while the number of complete plant mitochondrial genomes is only 673, and there are merely 285 plant species for which both the mitochondrial and chloroplast organellar genomes have been sequenced [11]. This stark disparity in the number of assembled organellar genomes underscores the significant challenges involved in sequencing complete plant mitochondrial genomes. These difficulties are primarily attributed to the dynamic and rapid structural changes that plant mitochondrial genomes undergo, resulting in complex genomic configurations marked by extensive rearrangements and the transfer of DNA from plastids. Such characteristics make mitochondrial genome sequencing and assembly more intricate compared to plastid genomes, often necessitating DNA reads from large inserts or long fragments [12]. Various approaches for assembling plant mitochondrial genomes using nuclear or organellar genomic whole-genome sequencing (WGS) data have been developed, such as SPAdes, NOVOPlasty, and GetOrganelle, which rely on Illumina short-read sequencing data [13]. When the assembled mitochondrial genomes lack repetitive sequences and possess a single predominant circular structure, these methods can generate relatively complete mitochondrial genomes [14,15]. However, the quality of the assembled mitochondrial genomes varies significantly due to differences in sequencing depth or assembly methods [16]. For mitochondrial genomes containing numerous repetitive sequences, these methods ultimately fail to produce complete mitochondrial genomes, as short-read sequencing data cannot span most of the repetitive regions [16]. Third-generation sequencing data can effectively traverse longer, complex regions, addressing the challenges posed by high repetition and complexity in mitochondrial genome assembly, thus enhancing assembly continuity [17]. Particularly, the high accuracy of HiFi reads (exceeding 99.9%) also ensures the accuracy of mitochondrial assembly [18]. The third-generation sequencing technologies, namely Oxford Nanopore Technology (ONT) and PacBio Single Molecule Real Time (SMRT) sequencing, can effectively span longer and more complex genomic regions, and by addressing the challenges posed by high repeatability and complexity in mitochondrial genome assembly, they improve the contiguity of the assembly while also ensuring its accuracy [19,20].

*Eryngium foetidum* L., a perennial herb belonging to the Apiaceae family, is widely distributed across various global tropical regions [21]. The entire plant contains sterols, and the young leaves are rich in various aromatic compounds, including dodecenal, tetradecenal, dodecyl aldehyde, and lauric acid [22]. As a result, it possesses a unique flavor and aroma similar to cilantro, and is commonly used as a seasoning for legumes, salads, meats, and fish [23]. The plant possesses specialized secretory canal cells distributed throughout its entire structure, which enable the accumulation of abundant essential oils predominantly composed of volatile aliphatic and aromatic compounds [24]. This characteristic enhances the economic value of *E. foetidum* in international trade, as well as in the fragrance and pharmaceutical industries [24]. Currently, *E. foetidum* is cultivated in Costa Rica and Puerto Rico for both local consumption and export, particularly to the United States [25]. In Brazil, its cultivation is primarily concentrated in the northern states of Acre, Amazonas, Amapá, Pará, Rondônia, and Roraima [26]. In China, the plant is mainly grown in small vegetable gardens and family farms. The cultivation of *E. foetidum* primarily relies on spring division, cuttings, and self-pollinated seeding, and these propagation patterns pose significant risks due to their dependence on inbreeding. On the other hand, the commercial scalability of *E. foetidum* is critically constrained by its indeterminate growth habit, wherein reproductive structures develop continuously over an extended period, leading to significant variability in seed physiological maturity within individual plants and complicating the standardization of harvest timing as well as the achievement of uniform seed quality [26].

Aside from the published chloroplast genomes and the utilization of chloroplast-plastid genes (*matK*, *Kim matK*, and *rbcL*) as well as the nuclear *ITS2* gene for species discrimination of two *Eryngium* genotypes collected from the east coast region of India, the comprehensive genomic resources of *E. foetidum* remain extremely limited, which imposes a significant constraint on the genetic breeding work for its commercial development [27]. Organellar genes in crops are associated with key metabolic pathways such as photosynthesis and respiration, as well as important traits including cold tolerance and sex differentiation [28]. The study of the organellar genome of *E. foetidum* is critical for advancing its medicinal and economic utilization, while mitochondrial genomic data can enhance breeding programs by identifying conserved genes associated with stress tolerance and yield traits. Moreover, biodiversity conservation in *E. foetidum* can be promoted by elucidating genetic relationships among species and identifying adaptive traits in its wild relatives.

In this study, we present the first complete assembly and characterization of the mitochondrial genome of *E. foetidum*, representing a pioneering contribution to the organellar genomics of this genus. Our research aims to (1) perform comprehensive functional annotation of the organellar genomes of *E. foetidum*; (2) conduct a thorough codon usage bias analysis of all coding sequences; (3) identify and characterize repetitive genomic elements; (4) investigate intracellular gene transfer events; and (5) clarify the phylogenetic relationships of *E. foetidum*. These genomic resources significantly expand the current understanding of its biology while providing valuable tools for genetic improvement and breeding applications.

## 2. Materials and Methods

### 2.1. DNA Extraction of Sample and Genome Sequencing

**Figure 1:**
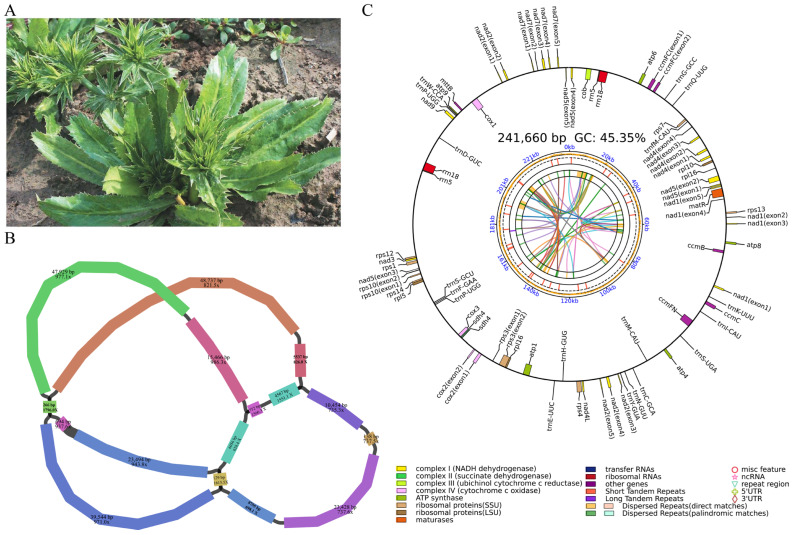
Schematic diagram of the mitochondrial genome assembly results of *E. foetidum.* (**A**) Growth morphology diagram of *E. foetidum*. (**B**) The read abundance supporting each configuration was used to determine the assembly. Different colors are used to represent regions corresponding to distinct contigs. (**C**) A schematic illustration of the mitochondrial genome of *E. foetidum*. Genes located within the circle are on the negative strand, while those outside the circle are on the positive strand. The colors correspond to different functional categories, as detailed in the legend. The colored parabola in the center circle represents the dispersed repeats.

The sample materials were collected from Lufeng City, Guangdong Province (22°5′ N, 115°3′ E) by the Guangdong Provincial Crop Germplasm Resources Collection Team, and subsequently cultivated at the Guangdong Provincial Crop Resources Nursery (sample accession number: 2021442092). On 3 June 2024, young leaves were sampled from an individual 3-year-old *Eryngium foetidum* plant (Figure 1A), and genomic DNA was extracted using the CTAB method—a classical experimental protocol that employs the cationic surfactant cetyltrimethylammonium bromide (CTAB) for the extraction and purification of plant genomic DNA [29]. The quality and concentration of the DNA were evaluated through 0.75% agarose gel electrophoresis, as well as with a NanoDrop One spectrophotometer (Thermo Fisher Scientific, Waltham, MA, USA) and a Qubit 3.0 fluorometer (Life Technologies, Carlsbad, CA, USA). To obtain high-quality complete mitochondrial genome sequencing data for *E. foetidum*, PacBio HiFi circular consensus sequencing (CCS) technology was employed [30]. Genomic DNA was processed using the SMRTbell Express Template Prep Kit 2.0 (sequencing was performed on a PacBio Sequel II by Guangzhou Yuda Biotechnology Company, Guangzhou, China). The adapter sequences of HiFi reads were removed using HiFiAdapterFilt v3.0.0, and upon completion of this program run, quality parameters such as average length and N50 were automatically output. Ultimately, HiFi reads were obtained, with an average length of 15–20 kb and a per-read accuracy exceeding 99%.

### 2.2. Assembly and Annotation of E. foetidum

To assemble a high-quality mitogenome and chloroplast of *E. foetidum*, we performed de novo assembly of PacBio HiFi reads (>10 kb) using the PacBio Multiplexed Assembly Tool (PMAT v1.5.3) [31]. The specific command is: PMAT autoMito -i HIFI.fa.gz -o./mito -st hifi -g 600m -tp all -fc 0.1. The assembly graph was visualized with Bandage v0.8.1 [32]. To ensure the assembly was purely mitochondrial, we conducted BLASTN v2.10.1 [33] searches against the chloroplast genome of *E. foetidum* and a comprehensive plant nuclear genome database. Any contigs exhibiting over 90% identity to chloroplast or nuclear sequences were systematically removed. The chloroplast and mitochondrial genomes of *E. foetidum* are available at https://github.com/luoyong123456/The-complete-mitochondrial-genome-of-Eryngium-foetidum (accessed on 19 June 2025) and Supplementary File S1.

The mitochondrial genome’s dual-circular structure was confirmed through a two-step validation process. Initially, circular configurations were inferred from the Bandage [32] assembly graph, revealing repeat-mediated recombination sites. In the second step, the entire 20 Gb HiFi dataset was mapped to the resolved genome structure, and coverage depth was analyzed. Continuous coverage without zero-depth regions confirmed the integrity of the structural resolution, ensuring that repeat-mediated nodes were correctly resolved and excluding mistakenly incorporated chloroplast or nuclear sequences.

Protein-coding genes (PCGs) were annotated via GeSeq [34], with BLAST v2.13.0 searches against curated organellar gene databases and an E-value threshold of 1 × 10^−5^. The Plant Mitochondrial Genome Annotation tool (PMGA v4.0; http://47.96.249.172:16084/home (accessed on 25 June 2025)) was also used for supplementary annotation [35]. Transfer RNA genes were predicted with tRNAscan-SE v2.0.7 [36], and ribosomal RNA genes were identified via BLASTN v2.10.1 [33] against known plant rRNA sequences. All preliminary annotations underwent manual curation and validation with Apollo v1.11.8 [37] to ensure accuracy and consistency. The final circular genome map was generated using OGDRAW v1.3.1 (https://chlorobox.mpimp-golm.mpg.de/OGDraw.html, accessed on 25 June 2025) [38].

### 2.3. Analysis of Codon Usage Bias and Selection Pressure

The protein-coding sequences were retrieved using Phylosuit software v1.2.3 with its default settings [39]. Codon usage bias and relative synonymous codon usage (RSCU) for the mitochondrial genome’s protein-coding genes were evaluated using MEGA software (version 7.0) [40]. To calculate the GC content in coding genes, the CUSP v1.4.1 online tool (https://www.bioinformatics.nl/cgi-bin/emboss/cusp (accessed on 29 June 2025)) was utilized. The effective number of codons (ENC) was determined using CodonW software (version 1.4.2) with default settings, indicating the degree of deviation from random codon usage. A two-dimensional scatter plot was created in Microsoft Excel 2021, with ENC values plotted on the y-axis and GC3 values on the x-axis. A standard curve was generated based on the formula ENC = 2 + GC3 + 29/[GC3^2^ + (1 − GC3)^2^] [41]. Each gene served as a data point, demonstrating the correlation between codon usage bias and the genomic base composition. Data points falling slightly above or below the curve suggested mutational influences, whereas those below the curve indicated selection and other factors influencing codon preference [42].

To assess selective pressures on mitochondrial protein-coding genes, we analyzed the ratio of non-synonymous substitution rate (Ka) to synonymous substitution rate (Ks) for the shared PCGs across nine plant species in the Apiaceae family. GenBank files of *Saposhnikovia divaricata* (accession number: NC_058846), *Apium graveolens* (accession number: MK562756), *Daucus carota* (accession number: JQ248574), *Ferula sinkiangensis* (accession number: OK585063), *Bupleurum chinense* (accession number: OK166971), *Coriandrum sativum* (accession number: MW477237), *Coriandrum sativum* (accession number: MW477238), and *Panax ginseng* (accession number: MW029460) were downloaded from the National Center for Biotechnology Information (NCBI, https://www.ncbi.nlm.nih.gov (accessed on 3 July 2025)) database. All these files were uploaded to the bioinformatics cloud platform (http://112.86.217.82:9919/#/ (accessed on 5 July 2025)) via the Ka/Ks cloud tool to retrieve the Ka/Ks values for shared proteins [43].

### 2.4. Repeat Sequence Identification and Prediction of RNA Editing Sites

The mitochondrial genome was examined for simple repeats utilizing MISA (https://webblast.ipk-gatersleben.de/misa/ (accessed on 10 July 2025)) [44], with repeat parameters set to 10 for mono-, 5 for di-, 4 for tri-, 3 for tetra-, 3 for penta-, and 3 for hexanucleotides, and a maximum distance of 100 for compound simple sequence repeats (SSRs). Tandem repeats longer than 6 bp with over 95% similarity were detected using Tandem Repeats Finder v4.09 (http://tandem.bu.edu/trf/trf.submit.options.html (accessed on 10 July 2025)) [45], with settings of 2 7 7 80 10 50 2000 -f -d -m. Dispersed repeats were identified using BLASTN (v2.10.1) with a 7 bp sequence length and an E-value of 1 × 10^−5^, while eliminating redundancy and tandem repeats. These repeats were visualized with Circos v0.69-5 (http://circos.ca/software/download/ (accessed on 11 July 2025)) [46]. Deepred-mt v1.01 [47], a tool based on a convolutional neural network (CNN) model, was employed to predict C-to-U RNA editing sites in the mitochondrial genome. For the prediction, all mitochondrial protein-coding genes were extracted and input into the Deepred-mt tool. Predictions with probability values greater than 0.9 were considered reproducible.

### 2.5. Identification of Homologous Fragments and Collinearity Analysis

Chloroplast genome annotation was performed using the CPGAVAS2 software (version 2.0) [48]. Homologous sequences between chloroplast and mitochondrial genomes were identified via the BLASTN software under default settings. The identified homologous fragments were visualized using the Circos package [46]. To investigate species evolution in greater detail, the BLASTN results were retrieved using the BLAST program, and multiple collinearity plots between *E. foetidum* and its closely related species were generated with the MCscanX software v2024.12.20-1 [49].

### 2.6. Construction of Maximum Likelihood Tree Based on the PCGs

We obtained the mitochondrial genomes of 21 species of Apiales with close phylogenetic relationships from the NCBI nucleotide database, which are crucial for elucidating their phylogenetic relationships and evolutionary history. Using PhyloSuite software v.1.2.3 [39], we extracted and identified all 26 protein-coding genes in the genome. Subsequently, all these protein-coding genes were concatenated using the Concatenate program, and the gene sequences were aligned with MAFFT v7.526 [50]. ModelFinder v2.2.2 was then employed to select the most appropriate substitution models. Phylogenetic analyses were conducted using both maximum likelihood (ML) [51] and Bayesian inference (BI) [52] methods. The ML tree was generated with IQ-TREE v2.2.2, with support values evaluated via the Shimodaira–Hasegawa approximate likelihood ratio test (SH-aLRT) using 1000 replicates, and ultrafast bootstrap (UFBoot) with 5000 replicates. For BI analysis, MrBayes v.3.2.6 [53] was used with 200,000 iterations, sampling every 100 generations, discarding the first 20% of trees, and producing a consensus tree from the remaining samples.

## 3. Results

### 3.1. Mitochondrial Genome Sequencing and Assembly of E. foetidum

The complete genome of *E. foetidum* was sequenced using PacBio HiFi technology, generating 2,892,309 high-precision HiFi reads with an average length of 15.83 kb and an N50 of 15.85 kb. The assembly yielded a draft mitochondrial genome composed of 16 contigs, with lengths varying from 129 to 48,737 bp and an average coverage of approximately 897.7× (Figure 1B). Analysis of the draft genome revealed a large circular structure of 241,660 bp with a GC content of 45.35% (Figure 1C), which is comparable to the mitochondrial genomes of other species within the Apiaceae family [54,55,56,57].

A total of 59 genes were identified in the mitochondrial genome, including 37 PCGs, 18 tRNA genes, and 4 rRNA genes, collectively representing 21.87% of the mitochondrial genome length (Table 1). The core PCGs of the *E. foetidum* mitochondrial genome consist of five ATP synthase genes (*atp1*, *atp4*, *atp6*, *atp8*, and *atp9*), four cytochrome c biogenesis genes (*ccmB*, *ccmC*, *ccmFc*, and *ccmFn*), three cytochrome c oxidase genes (*cox1*, *cox2*, and *cox3*), eight NADH dehydrogenase genes (*nad1*, *nad2*, *nad3*, *nad4*, *nad4L*, *nad5*, *nad7*, and *nad9*), one ubiquinol–cytochrome c reductase gene (*cob*), one maturase gene (*matR*), and one transport membrane protein gene (*mttB*). The variable PCGs include three small ribosomal protein subunits (*rps3*, *rps4*, and *rps12*), two large ribosomal protein subunits (*rpl5* and *rpl10*), and one succinate dehydrogenase gene (*sdh4*). Additionally, some tRNA and rRNA genes are present in duplicate, such as *trnP-UGG*, *rrn18*, and *rrn5*. Several genes contain multiple introns, with four PCGs (*nad1*, *nad2*, *nad5*, and *nad7*) each containing four introns, and *rps10*, *cox2*, *rps3*, and *ccmFC* each containing one intron.

### 3.2. SSRs and Dispersed Repetitive Sequences

**Figure 2:**
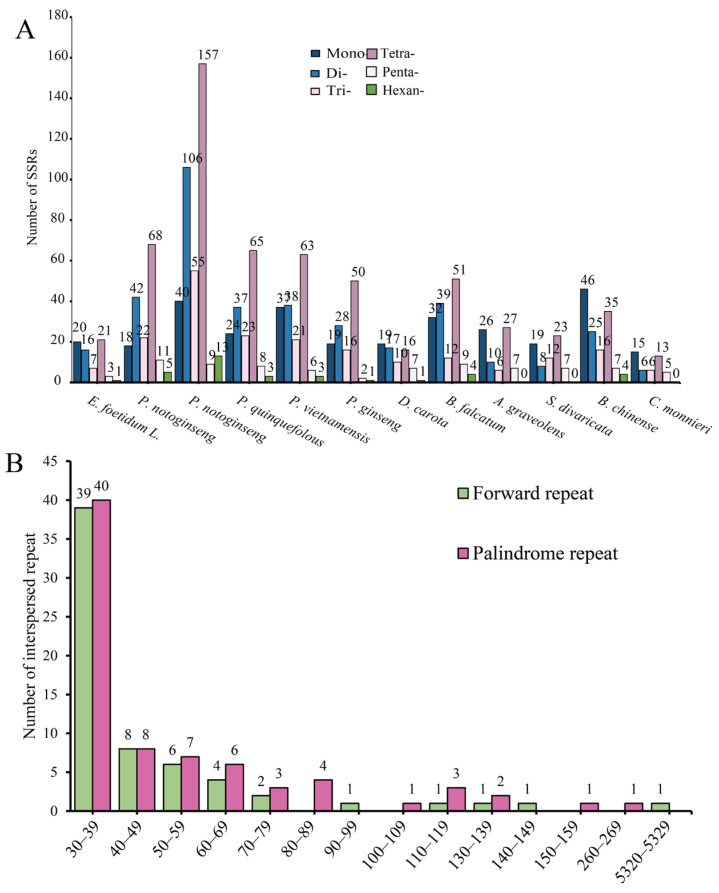
Mitochondrial genomic repeat sequences of *E. foetidum*. (**A**) Comparative analysis of the quantities of simple sequence repeats and the sizes of mitochondrial genomes across 12 Apiales species, with insights from vertebrate mitochondrial genomes revealing repeats and gene duplications. (**B**) Distribution of repeat sequence lengths within the mitochondrial genome of *E. foetidum*.

Repeats are essential to the evolution of plant mitochondrial genomes [58]. SSRs consist of short, tandemly arranged sequence motifs, with lengths ranging from 1 to 6 base pairs. The mitochondrial genome of *E. foetidum* was found to harbor 68 SSRs, with the maximum number of repeat motifs being 21 for tetranucleotide repeats. Additionally, repeat sequences of mononucleotides, dinucleotides, trinucleotides, pentanucleotides, and hexanucleotides were detected at 20 (30.88%), 16 (23.53%), 7 (10.29%), 3 (4.41%), and 1 (1.47%) sites (Figure 2A). In comparison, other mitochondrial genomes within the order Apiales were found to contain between 45 and 380 SSRs. Meanwhile, we also detected that in the mitochondrial genome of *E. foetidum*, tetranucleotide repeat motifs accounted for the largest number of SSRs, whereas hexanucleotide repeat motifs were the least common. In the mitochondrial genome of *E. foetidum*, a total of 140 dispersed repetitive sequences, each with a length of 30 bp or more, were identified. These include 64 pairs of forward repeats and 76 pairs of palindromic repeats. The number of dispersed repeats considerably exceeds that of SSRs. The cumulative length of these long repetitive sequences is 12,260 bp, representing 5.75% of the mitochondrial genome. Most of the repetitive sequences are 30–50 bp in length (97 instances, 68.79%), with the longest repetitive sequence being 5321 bp, representing 21.7% of the total length (Figure 2B).

### 3.3. Characterization of Chloroplast Genome Transfer to Mitochondria in E. foetidum

**Figure 3:**
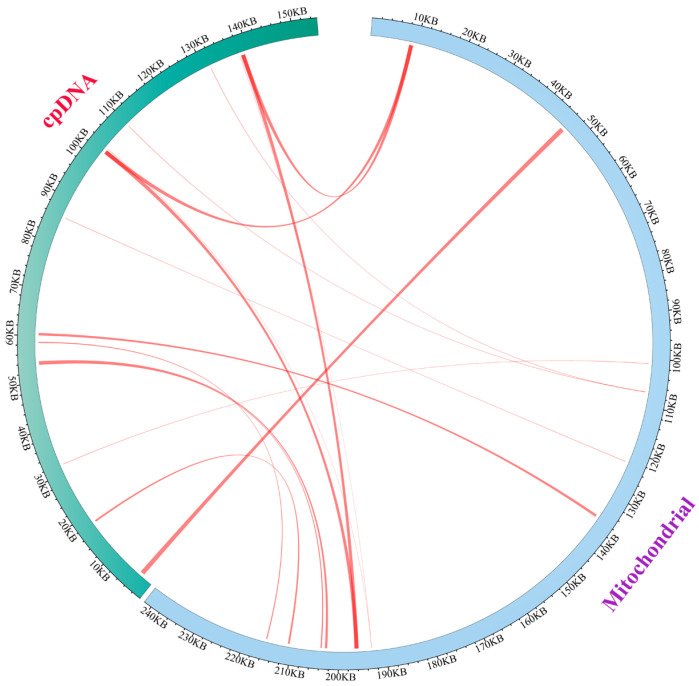
Evidence of chloroplast-to-mitochondria gene transfer is observed within the *E. foetidum* genome. The mitochondrial and chloroplast genomes are represented by blue and green arcs, respectively, while the red lines connecting the arcs represent homologous genomic fragments. The thicker the line, the higher the homology.

In the evolutionary journey of higher plants, the transfer of genetic material among cells is a common phenomenon observed in mitochondrial genomes [59]. Analysis of sequence similarity uncovered 16 homologous segments spanning from 38 to 992 bp between the chloroplast and mitochondrial genomes (Figure 3). The number of mismatches within these segments varied from 1 to 179. In total, these homologous fragments span 6842 base pairs, representing approximately 2.83% of the mitochondrial DNA in *E. foetidum*. This percentage is significant as it reflects a substantial portion of the mitochondrial genome, which in humans is typically 16,569 base pairs long and contains 37 genes [60]. These fragments are designated as MTPTs, indicating sequences transferred from the chloroplast to the mitochondrion. Among them, MTPT 1 is the largest homologous segment, measuring 992 base pairs.

Further annotation of these sequences uncovered the transfer of six complete genes (*trnH-GUG*, *trnN-GUU*, *trnM-CAU*, *trnD-GUC*, *trnP-UGG*, *trnW-CCA*), along with several gene fragments (*rrn16*, *rpl14*, *rpl16*, *rpoB*), from the chloroplast to mitochondrial gene regions or intergenic spaces (IGS) (Table 2).

### 3.4. Analysis of Relative Synonymous Codon Usage

**Figure 4:**
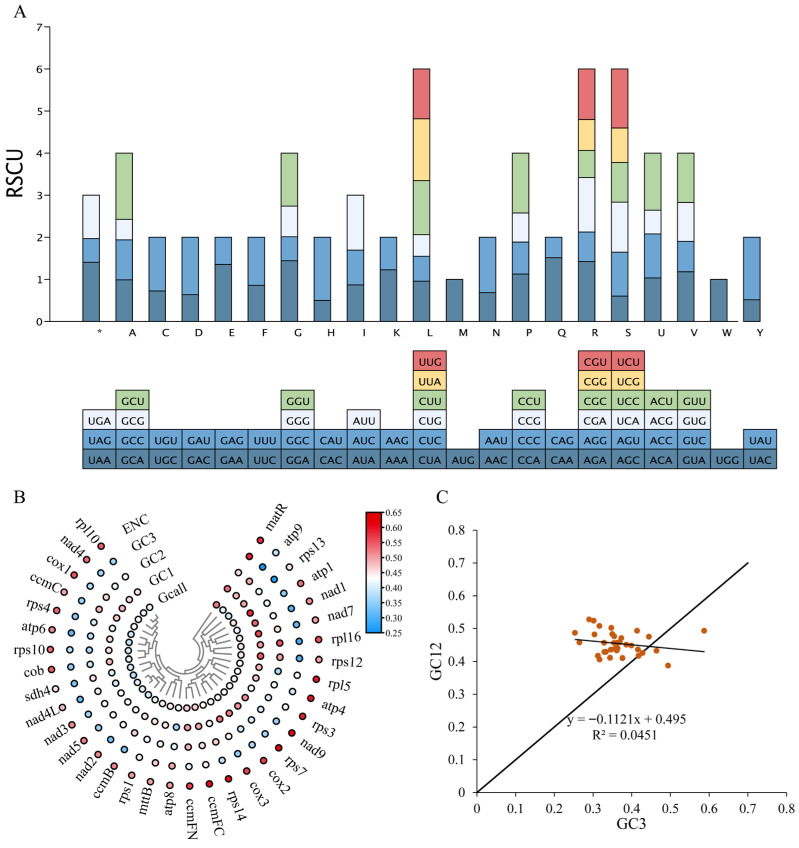
Codon usage bias analysis and GC content variation across different positions in the protein-coding genes (PCGs). (**A**) Analysis of relative synonymous codon usage in the *E. foetidum* mtDNA. *: denotes terminator. (**B**) GC content of different positions from PCGs. Variation in GC content at different positions is indicated by circle size, and values are suggested by different colors. (**C**) Neutrality plot analysis. Points of various colors represent individual genes, while the black line indicates the trend.

In the complete mitochondrial genome of *E. foetidum*, a total of 10,058 codons were identified within the protein-coding genes (Table 3). This genome encodes all 20 amino acids, with 61 distinct codon types observed. Among these, the most commonly utilized codon is UUU, appearing 361 times, while leucine has the highest codon count, comprising 1022 codons (10.44% of the total), followed by serine with 936 codons (9.19%). This is consistent with the codon usage patterns observed in mitochondrial genomes, where specific codons are frequently used to encode particular amino acids. Conversely, cysteine is represented by the fewest codons, totaling only 141 (1.42% of the total). Our analysis revealed that 32 codons are used more frequently than expected (RSCU > 1), whereas 31 codons occur less often than expected (RSCU < 1). Notably, methionine (AUG) and tryptophan (UGG) exhibit no codon bias (RSCU = 1). With the exception of these two codons, the usage patterns of most amino acids show a distinct bias (Figure 4A).

We identified 35 distinct PCGs in the mitochondrial genome of *E. foetidum* and examined the GC content at the first (GC1), second (GC2), and third (GC3) codon positions. The GC1 content ranged from 36.25% to 58.04%, GC2 from 36.75% to 55.17%, and GC3 from 25.33% to 58.69%. The average GC content across these positions (GC1, GC2, and GC3) was consistently below 50%, aligning with the observed preference for A/T bases and A/T-ending codons in the mitochondrial genomes of various organisms, as evidenced by studies on *E. foetidum*. We also computed the ENC for these PCGs, which varied from 38.75 to 52.55, with a mean ENC greater than 35. This implies a relatively weak codon usage bias in the mitochondrial DNA of *E. foetidum* (Figure 4B). Furthermore, the analysis of neutral plots revealed a significant negative correlation of −0.112 between GC12 and GC3, which is considerably lower than anticipated (*p* = 0.05), suggesting that natural selection exerts a substantial influence on the codon usage bias within the mitochondrial genome of *E. foetidum* (Figure 4C).

### 3.5. Predicted RNA Editing Sites

**Figure 5:**
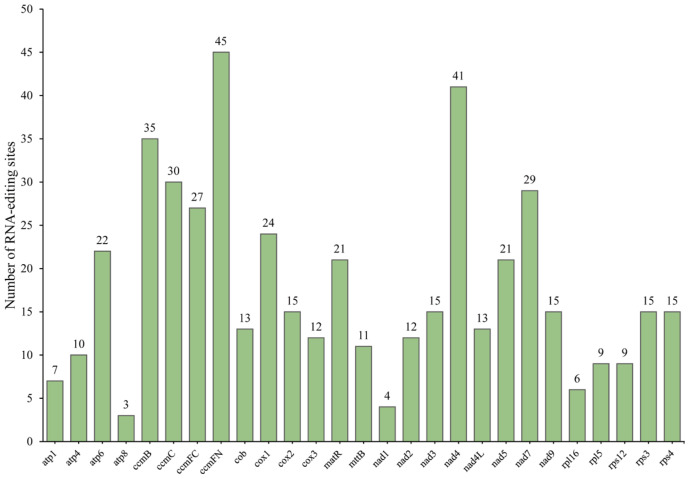
The number of predicted RNA editing sites in the mitochondrial genes of *E. foetidum*. The green bars represent the number of RNA editing sites of each gene.

Using a deep learning approach (Deepred-mt), we predicted C-to-U RNA editing sites within the mitochondrial genome. Detailed information for each predicted RNA editing site is provided in Table S1. The analysis identified 479 high-confidence C-to-U RNA editing sites in 27 protein-coding genes (Figure 5). The *ccmFN* gene was prominent, featuring the highest number of predicted editing sites, totaling 45, followed by the *nad4* gene, which harbored 41 predicted editing sites. Genes like *ccmC* and *ccmB* also displayed over 30 predicted RNA editing sites. In contrast, genes such as *atp8*, *nad1*, and *rpl16* exhibited fewer predicted editing events, ranging from 3 to 6 C-to-U sites per gene (Figure 5). Some RNA editing modifications lead to the formation of premature stop codons, as observed in the *nad3* gene, where the CAG codon is converted to UAG, and in the *atp6* gene, where CAA is replaced by UAA. Additionally, RNA editing events that alter CGA codons to UGA are predicted in the *ccmFC* and *atp9* genes. Furthermore, RNA editing that changes ACG codons to AUG codons plays a crucial role in generating initiation codons in genes such as *ccmFC*, *atp8*, *nad4L*, *cox2*, *cox1*, and *nad7*.

In this study, all predicted RNA editing sites resulted in non-synonymous mutations, indicating a potential impact on protein function. Following RNA editing, a significant portion of amino acids undergoes hydrophobicity shifts: 11.90% remain hydrophilic, 29.85% retain hydrophobic properties, 47.39% transition from hydrophilic to hydrophobic, and 10.02% change from hydrophobic to hydrophilic (Table S2). Additionally, 0.42% of the amino acid sequences result in premature termination due to alterations disrupting the coding sequence. The most frequent amino acid alteration was the conversion of serine (Ser) to leucine (Leu), occurring 109 times.

### 3.6. PCG Substitution Rates and Phylogenetic Tree Based on the PCGs

**Figure 6:**
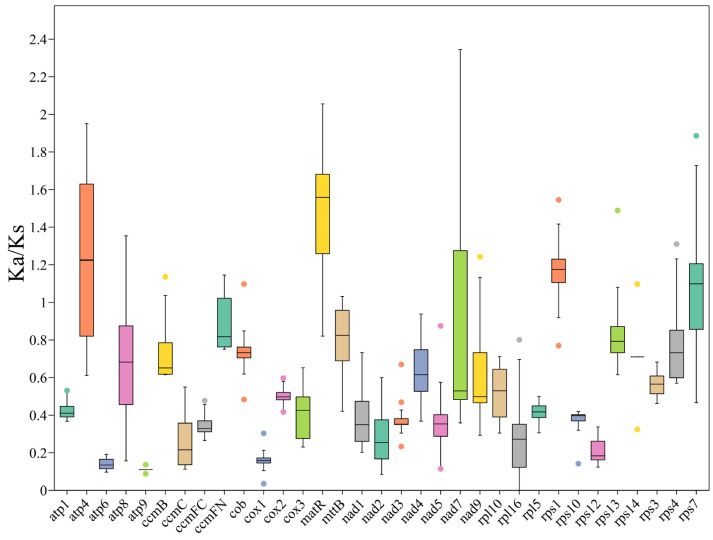
Boxplots of the Ka/Ks ratios (values are *Mean* ± SD) among the mitochondrial genome genes of *E. foetidum* and 8 other Apiaceae plant species. Colors are used only to distinguish genes and do not carry biological significance.

To investigate the impact of selective pressure on the evolution of the *E. foetidum* mitochondrial genome, we assessed the ratio of non-synonymous (Ka) to synonymous (Ks) substitutions in shared protein-coding genes (PCGs) across *E. foetidum* and eight other Apiaceae plant species (Figure 6), and the specific values of the Ka/Ks ratio are provided in Table S3. A Ka/Ks < 1 indicates purifying selection, Ka/Ks = 1 indicates neutral evolution, and Ka/Ks > 1 indicates positive selection. Our analysis reveals that most PCGs in the *E. foetidum* mitochondrial genome are under purifying selection, with Ka/Ks values significantly below 1. Notably, the Ka/Ks > 1 for *rps7*, *rps1*, *atp4*, and *matR* genes, suggesting potential positive selection associated with environmental adaptation.

**Figure 7:**
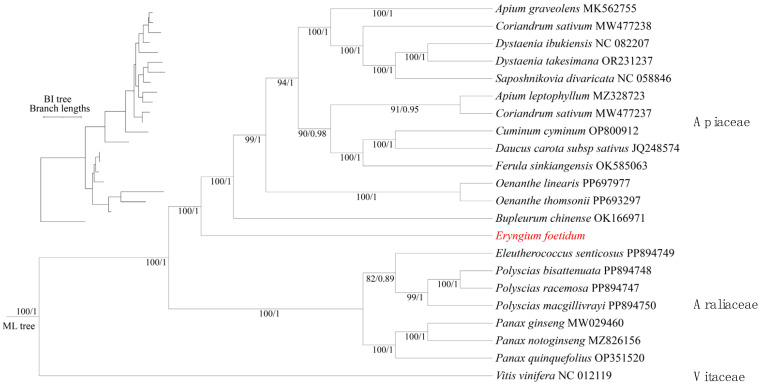
Phylogenetic tree of *E. foetidum* (red color) and 21 other species. A phylogenetic tree was generated using conserved protein sequences and analyzed with maximum likelihood (ML) and Bayesian inference (BI) methods. The reliability of the tree was evaluated with bootstrap scores from 1000 replicates, with ML bootstrap support values and BI posterior probabilities indicated at the corresponding nodes.

Furthermore, we conducted a phylogenetic analysis based on DNA sequences of 30 shared mitochondrial protein-coding genes (PCGs) from 21 species (Figure 7). The results indicate that *E. foetidum* occupies a basal phylogenetic position within the Apiaceae family. This finding, consistent with phylogenetic trees reconstructed from chloroplast gene fragments in previous studies, further validates the utility of mitochondrial data for resolving plant evolutionary relationships due to such consistency between organellar genomes [61]. Moreover, this phylogenetic placement aligns with the latest Angiosperm Phylogeny Group (APG) system, supporting contemporary perspectives in plant taxonomy.

## 4. Discussion

In this study, whole-genome sequencing of *E. foetidum* was performed using PacBio HiFi sequencing data. We report, for the first time, the complete mitochondrial genome of *E. foetidum*, which exhibits a circular structure with a total length of 241,660 bp and a GC content of 45.35%. The mitochondrial genome encodes 59 genes, comprising 37 protein-coding genes, 18 tRNA genes, and 4 rRNA genes. These genomic features are consistent with those observed in most species within the Apiaceae family [56,62,63]. The mitochondrial genome size varies among different species within the Apiaceae family, while the number of encoded genes remains relatively consistent. This suggests that variations in mitochondrial genome size and structural organization are primarily due to differences in non-coding sequences. The Ka/Ks ratio serves as a valuable indicator for assessing the evolution of flowering plant genes in response to environmental pressures [64,65]. When studying genetic variations among individuals or populations of seed plants and their phenotypic implications, the Ka/Ks ratio can elucidate evolutionary trajectories and underlying genetic mechanisms [66]. Our analysis of the Ka/Ks ratios for shared PCGs between the mitochondrial genome of *E. foetidum* and eight other Apiales species yielded results consistent with existing studies, indicating that very few protein-coding genes have been influenced by positive selection [5,67,68,69,70]. Genes *ccmFn*, *mttB*, *rps10*, and *matR* exhibited Ka/Ks ratios greater than 1, suggesting their potential involvement in redox reactions, response to environmental stress, and other adaptive processes [71,72].

RNA editing represents a widespread and phylogenetically conserved post-transcriptional modification in angiosperm mitochondria, serving as a major source of genomic variation through editase-mediated C-to-U or U-to-C conversions that ultimately alter RNA sequences and modify translated protein products [73]. This modification mechanism is closely associated with plant evolution, environmental adaptation, developmental processes, and the induction of crop male sterility [74]. In our study, a total of 479 potential RNA editing sites were identified across 27 distinct protein-coding genes (PCGs), all characterized by cytidine (C)-to-uridine (U) conversions. The majority of these editing sites occurred at the first or second codon positions, consistent with observations reported in other plant species [75]. Notably, RNA editing can induce alterations in both start and stop codons of protein-coding sequences, as exemplified by the conversion of a CAG codon to UAG in the *nad3* gene and the modification of a CAA codon to UAA in the *atp6* gene. Gallagher et al. [76] demonstrated that male sterility in maize pollen is associated with the truncation of the chimeric open reading frame (orf77), which is caused by the premature termination of mitochondrial RNA editing. Additionally, Kadowaki et al. [77] and Quiñones et al. [78] identified novel start codons of *cox1* in the transcripts of tomato and potato, respectively. The novel start and stop codons generated through RNA editing often lead to the production of evolutionarily more conserved proteins, which exhibit high homology with their counterparts in other species and enhance mitochondrial gene expression [79].

Codons in seed plants carry critical recognition and translational information, playing a significant role in the context of genetic mutations. During protein translation, the usage of some synonymous codons is markedly influenced by species-specific variations, resulting in codon usage bias—a phenomenon in which the preferential use of synonymous codons plays a crucial role in shaping the genetic architecture of these organisms [43]. In this study, we evaluated the GC content at different codon positions and the relative synonymous codon usage in the mitochondrial genome of *E. foetidum* using 35 distinct PCGs. The results indicated a distinct preference for A/T bases and A/T-ending codons at the third codon position in the mitochondrial genome of *E. foetidum*, which aligns with findings in most dicot mitochondrial genomes and suggests a conserved pattern of codon usage bias across species [80]. Additionally, neutrality plot analysis revealed that codon usage in the mitochondrial genome of *E. foetidum* is predominantly influenced by natural selection. Furthermore, a total of 32 biased codons (RSCU > 1) were identified, with a notable preference for A/T bases in their composition. Therefore, future studies aimed at generating genetic breeding materials should take into account mitochondrial RNA editing sites and codon usage bias to facilitate the genetic breeding process.

The transfer of genetic fragments between chloroplast and mitochondrial genomes represents a fundamental characteristic of plant mitochondrial genome evolution [81]. Exogenous gene insertions into the mitochondrial genome exhibit a pronounced preference for integration into intergenic regions [22,82]. The length of chloroplast DNA integrated into the mitochondrial genome varies among different species, typically ranging from 1% to 12% of the chloroplast genome sequence in angiosperms [3]. This phenomenon is one of the primary factors contributing to the variation in the number of coding genes within the mitochondrial genomes of different plants. Therefore, tracking gene transfer is crucial for exploring the evolution of plant mitochondrial genomes [3]. The transfer of tRNA gene sequences from the chloroplast genome to the mitochondrial genome is common in plants [73]. In this study, six complete tRNA genes were identified in the chloroplast of *E. foetidum*, which were fully transferred to the intergenic regions or gene fragments of the mitochondrial genome. The tRNAs derived from the chloroplast may possess potential functional complementation. In addition, several chloroplast gene fragments were also identified as having migrated into the mitochondrial genome. These chloroplast genome-derived fragments contain genes that exert important functions in the chloroplast; however, whether they perform functions in the mitochondrial genome remains unclear. Understanding the patterns of sequence transfer is crucial for tracing ancient recombination events and structural variations in plant mitochondrial genomes, yet this area remains underexplored and warrants further research attention.

Plant mitochondrial genomes exhibit slow sequence evolution, with the relative synonymous substitution rates among mitochondrial, chloroplast, and nuclear genes in angiosperms following an approximate ratio of 1:3:16 [83]. Consequently, phylogenetic analyses are typically conducted using the nuclear genome or chloroplast genome. With the increasing availability of mitochondrial genome sequencing data, substantial variations in mitochondrial genomes have been observed, including considerable size divergence and a remarkable abundance of structural diversity [84]. Mitochondrial genomes have emerged as a valuable tool for research in taxonomy, phylogenetics, evolutionary studies, population genetics, and comparative genomics [11]. Our study conducted a phylogenetic analysis based on the mitochondrial genome of *E. foetidum* and 21 publicly available plant mitochondrial genomes. Both maximum likelihood (ML) and Bayesian inference (BI) methods produced identical clustering results, demonstrating a strong concordance between traditional taxonomy and molecular-based classification. The phylogenetic tree shows that *E. foetidum* is positioned at the base of the Apiaceae family. This result is consistent with previous phylogenetic studies based on chloroplast genomes, thereby highlighting the potential of mitochondrial genomic data in resolving phylogenetic relationships among plants [61]. Nevertheless, the sequencing of additional mitochondrial genomes from Eryngium and other Apiaceae species is necessary to further investigate species delineation, phylogenetic relationships, and evolutionary biological characteristics within this large and complex genus.

## 5. Conclusions

In this study, we successfully assembled and annotated the mitochondrial genome of *E. foetidum*. The resulting mitochondrial genome revealed a circular structure with a total length of 241,660 bp, containing 59 annotated genes, including 37 PCGs, 18 tRNA genes, and 4 rRNA genes. In our extended genomic analysis, we delved into critical aspects such as predicted RNA editing sites, codon usage patterns, repeat sequences, homologous segments between mitochondria and chloroplasts, and the Ka/Ks ratios. For instance, the Ka/Ks ratio, a measure of selection pressure, was examined to understand the evolutionary dynamics of the studied genome. Additionally, the mitochondrial genome’s unique features, such as its semi-autonomous nature and rapid evolutionary rate, were considered to provide insights into the organism’s evolutionary history and adaptation, gaining deeper insights into the evolutionary processes that have shaped the mitochondrial genome of *E. foetidum*. Moreover, phylogenetic analysis based on the mitochondrial genome, along with data from 21 other plant mitochondrial genomes, supported the evolutionary classification of *E. foetidum*. This study offers essential genomic information on *E. foetidum*, serving as a valuable resource for future research endeavors in species identification, genetic diversity, and phylogenetic studies.

## Figures and Tables

**Table 1:** Predicted genes in the mitochondrial genome of *E. foetidum*.

Group of Genes	Gene Name
ATP synthase	*atp1 atp4 atp6 atp8 atp9*
Cytochrome c biogenesis	*ccmB ccmC ccmFC* * *ccmFN*
Ubiquinol cytochrome c reductase	*cob*
Cytochrome c oxidase	*cox1 cox2* * *cox3*
Maturases	*matR*
Transport membrane protein	*mttB*
NADH dehydrogenase	*nad1* **** *nad2* **** *nad3 nad4* **** *nad4L nad5* **** *nad7* **** *nad9*
Ribosomal proteins (LSU)	*rpl10 rpl16*(2) *rpl5*
Ribosomal proteins (SSU)	*rps1 rps10* * *rps12 rps13 rps14 rps3* * *rps4 rps7*
Succinate dehydrogenase	*sdh4*(2)
Ribosomal RNAs	*rrn18*(2) *rrn5*(2)
Transfer RNAs	*trnC-GCA trnD-GUC trnE-UUC trnF-GAA trnG-GCC trnH-GUG trnI-CAU trnK-UUU trnM-CAU trnN-GUU trnP-UGG*(2) *trnQ-UUG trnS-GCU trnS-UGA trnW-CCA trnY-GUA trnfM-CAU*

Notes: *: denotes one intron; ****: denotes four introns; Gene(2) indicates the copy number of multi-copy genes.

**Table 2:** Horizontal gene transfer from chloroplast to mitochondria in *E. foetidum*.

No.	Length	Identity %	Mismatches	Gap Opens	cp Start	cp End	Gene (cp)	mt Start	mt End	Gene (mt)
1	992	87.5	110	7	2465	3447	*rpl16* (26.65%)- *rpl14* (55.23%) (IGS)	45,655	46,641	*trnM-CAU* (100%)- *rpl2* (17.62%) (IGS)
2	526	97.34	13	1	60,034	60,558	*rpoB* (16.34%)	135,392	135,917	*nad7* (8.48%)
3	502	95.62	17	1	54,022	54,523	*trnD-GUC-* *psbM* (IGS)	202,447	202,943	*atp9*-*rps12* (IGS)
4	276	97.10	5	3	53,748	54,023	*trnD-GUC* (100%)	203,615	203,887	*atp9*-*rps12* (IGS)
5	215	93.95	10	3	58,651	58,438	*rpoB* (6.66%)	215,418	215,630	*cox3*-*trnS* (GCU)
6	887	74.07	179	37	101,982	102,845	*rrn16* (57.95)	195,740	196,598	*atp4*-*nad6* (IGS)
7	887	74.07	179	37	101,982	102,845	*rrn16* (57.95)	8359	9217	*atp6*-*trnQ*- *UUG* (IGS)
8	887	74.07	179	37	139,163	138,300	*rrn16* (57.95)	8359	9217	*atp6*-*trnQ*- *UUG* (IGS)
9	887	74.07	179	37	139163	138,300	*rrn16* (57.95)	195,740	196,598	*atp4*-*nad6* (IGS)
10	386	80.57	43	13	18,122	17,758	*trnP-UGG* and *trnW-CCA*	210,522	210,896	*rpl5* (65.45%)
11	76	98.68	1	0	85,798	85,873	*trnH-GUG* (100%)	122,443	122,518	*Cox* (9.52%)
12	83	96.39	2	1	109,761	109,842	*trnN-GUU* (100%)	106,978	107,060	*nad2*-*rps12* (IGS)
13	83	96.39	2	1	131,384	131,303	*trnN-GUU* (100%)	106,978	107,060	*nad2*-*rps12* (IGS)
14	79	93.67	5	0	31,919	31,841	*trnM-CAU* (100%)	100,899	100,977	*nad2* (5.18%)
15	38	97.37	1	0	103,140	103,177	*rrn16* (2.55%)	192,948	192,985	*cob* (3.17%)
16	38	97. 367	1	0	138,005	137,968	*rrn16* (2.55%)	192,948	192,985	*cob* (3.17%)

Notes: Gap open: Gap open refers to the penalty for initiating a gap insertion, which is usually larger than the penalty for gap extension; Gene (cp): Chloroplast genes, where the value in parentheses represents the proportion of homologous sequences in the gene; Gene (mt): Mitochondrial genes, where the value in parentheses represents the proportion of homologous sequences in the gene; IGS: intergenic spacer, which refers to the intergenic spacer region.

**Table 3:** The relative synonymous codon usage for each amino acid in the mitochondrial genome of *E. foetidum*.

Symbol	Codon	Count	RSCU	Symbol	Codon	Count	RSCU
Ter	UAA	15	1.4062	Met	AUG	267	1
Ter	UAG	6	0.5625	Asn	AAC	110	0.6832
Ter	UGA	11	1.0312	Asn	AAU	212	1.3168
Ala	GCA	158	0.9875	Pro	CCA	163	1.1261
Ala	GCC	152	0.95	Pro	CCC	110	0.7599
Ala	GCG	78	0.4875	Pro	CCG	100	0.6908
Ala	GCU	252	1.575	Pro	CCU	206	1.4231
Cys	UGC	51	0.7234	Gln	CAA	218	1.5139
Cys	UGU	90	1.2766	Gln	CAG	70	0.4861
Asp	GAC	101	0.6352	Arg	AGA	168	1.4217
Asp	GAU	217	1.3648	Arg	AGG	83	0.7024
Glu	GAA	287	1.3538	Arg	CGA	153	1.2948
Glu	GAG	137	0.6462	Arg	CGC	76	0.6432
Phe	UUC	272	0.8594	Arg	CGG	87	0.7362
Phe	UUU	361	1.1406	Arg	CGU	142	1.2017
Gly	GGA	253	1.4416	Ser	AGC	94	0.6026
Gly	GGC	100	0.5698	Ser	AGU	163	1.0449
Gly	GGG	128	0.7293	Ser	UCA	185	1.1859
Gly	GGU	221	1.2593	Ser	UCC	147	0.9423
His	CAC	61	0.498	Ser	UCG	128	0.8205
His	CAU	184	1.502	Ser	UCU	219	1.4038
Ile	AUA	222	0.8672	Thr	ACA	130	1.0317
Ile	AUC	212	0.8281	Thr	ACC	132	1.0476
Ile	AUU	334	1.3047	Thr	ACG	71	0.5635
Lys	AAA	274	1.226	Thr	ACU	171	1.3571
Lys	AAG	173	0.774	Val	GUA	181	1.1792
Leu	CUA	163	0.9569	Val	GUC	111	0.7231
Leu	CUC	101	0.593	Val	GUG	142	0.9251
Leu	CUG	87	0.5108	Val	GUU	180	1.1726
Leu	CUU	219	1.2857	Trp	UGG	144	1
Leu	UUA	250	1.4677	Tyr	UAC	83	0.5139
Leu	UUG	202	1.1859	Tyr	UAU	240	1.4861

## Data Availability

The sample has been stored at the Guangdong Provincial Crop Germplasm Resource Nursery in Guangzhou, China, under accession number 2021441581. The mitochondrial and chloroplast genome sequences that underpin the findings of this study are publicly available at https://ngdc.cncb.ac.cn/gwh (accessed on 15 June 2025). Additionally, the raw data have been archived in the database https://www.ncbi.nlm.nih.gov/bioproject/1145859 (accessed on 15 June 2025) (Nanopore: SPRJNA1145859). The assembled mitogenome and genome annotation of *E. foetidum* were deposited in a database (https://github.com/luoyong123456/The-complete-mitochondrial-genome-of-Eryngium-foetidum.git (accessed on 20 July 2025)).

## Supplementary Materials

The following supporting information can be downloaded at: https://www.mdpi.com/article/10.3390/biology14091296/s1, Table S1: Detailed information on RNA predicted dditing sites in the mitochondrial genome of *E. foetidum*; Table S2: Analysis of RNA editing sites and types in the *E. foetidum* mt genome; Table S3: The Ka/Ks ratio among shared mitochondrial genome genes across 9 plant species in the Apiaceae family.

## Abbreviations

PCGs	Protein-coding gene
ATP	Adenosine triphosphate
CMS	Cytoplasmic male sterility
ORFs	Open reading frames
RSCU	Relative synonymous codon usage
ENC	Effective number of codons
SSRs	Simple sequence repeats
IGS	Intergenic spaces
