# Supplementary material for: Assembly and Analysis of the Complete Mitochondrial Genome of Eryngium foetidum L. (Apiaceae)
Source: Biology (Basel). 2025 Sep 19;14(9):1296. doi: 10.3390/biology14091296 (PMC12467312; doi:10.3390/biology14091296)
Supplement: Supplementary file 1 [file biology-14-01296-s001.zip › biology-3815664-supplementary File S1.pdf]

Assembly and Analysis of the Complete Mitochondrial Genome of *Eryngium foetidum* L. (Apiaceae)

Lihong Zhang [2,†], Wenhu Zhang [2,†], Yongjian Luo [2], Jun Liu [2], Qing Li [2,*] and Qiongheng Liu [1,*]

[1] Maoming Agricultural Science and Technology Extension Center, Maoming 525000, China
[2] Guangdong Key Laboratory for Crop Germplasm Resources Preservation and Utilization, Agro-Biological Gene Research Center, Guangdong Academy of Agricultural Sciences, Guangzhou 510640, China; zhanglihong200008@126.com (L.Z.); zhangwenhu@agrogene.ac.cn (W.Z.); yongjianluo1996@outlook.com (Y.L.); liujun@gdaas.cn (J.L.)
* Correspondence: liqing@agrogene.ac.cn (Q.L.); maominglqh@163.com (Q.L.); Tel.: +86-13509925221 (Qiongheng Liu)
† These authors contributed equally to this work.

The file of mitochondrial and chloroplast genome sequences and annotation of *E. foetidum*

```
LOCUS       Eryngium_foetidum_L.          241,660 bp    DNA     circular PLN 25-
JUL-2024
DEFINITION  Eryngium_foetidum_L. mitochondrion, complete genome.
ACCESSION   Eryngium_foetidum_L.
VERSION     Eryngium_foetidum_L.
KEYWORDS    .
SOURCE      mitochondrion YOUR_SPECIES
  ORGANISM  YOUR_SPECIES YOUR_SPECIES.
REFERENCE   1   (bases 1 to 241660)
  AUTHORS   __LASTNAME__.
  TITLE     MGA: Mitochondrial Genome Annotator for Angiosperms
  JOURNAL   Unpublished
REFERENCE   2   (bases 1 to 241660)
  AUTHORS   __LASTNAME__.
  TITLE     Direct Submission
  JOURNAL
FEATURES             Location/Qualifiers
     source          1..241660
                     /organism="Arabidopsis thaliana"
                     /organelle="mitochondrion"
                     /mol_type="genomic DNA"
                     /db_xref="taxon:3702"
     gene            75701..76085
                     /gene="nad1"
     CDS             join(75701..76085,58668..58750,60188..60379,
                     complement(54122..54180),complement(50758..51016))
                     /gene="nad1"
```

/exception="trans-splicing"
/product="NADH dehydrogenase subunit 1"
/codon_start=1
/transl_table=11

/translation="MYIAVPAEILGIILPLLLGVAFLVLAERKVMAFVQRRKGPDVVGS

FGLLQPLADGLKLILKEPISPSSANFSLFRMAPVATFMLSLVARAVVPFDYGMVLSDPN

IGLLYLFAISSLGVYGIIIAGRSSNSKYASLGALRSAAQMVPYEVSIGLILITVLICVG

PRNSSEIVMAQKQIWSGIPLFPVLVMFFISCLAETNRAPFDLPEAEAESVAGYNVEYSS

MGSALSFLGEYANMILMSGPCTSLSPGGWPPFLDLPISKKIPGSIRFSIKVILFPFLYI
WVRAAFPRYRYDQLMGLGRKVFLPLSLARVVPVSGVLVTFQWLP*"

exon 75701..76085
/gene="nad1"
/number=1

exon 58668..58750
/gene="nad1"
/number=2

exon 60188..60379
/gene="nad1"
/number=3

exon complement(54122..54180)
/gene="nad1"
/number=4

exon complement(50758..51016)
/gene="nad1"
/number=5

gene 224544..226294
/gene="nad2"

CDS join(224544..224696,225903..226294,108724..108884,
111191..111763,113246..113433)
/gene="nad2"
/exception="trans-splicing"
/product="NADH dehydrogenase subunit 2"
/codon_start=1
/transl_table=11

/translation="MFNLFLAVSPEIFLINATFILLIHGVVFSTSKKYDYPPLVSNVGW

LGLLSVLITLLLLAAGAPLLTIAHLFWNNLFRRDNFTYFCQILLLLSTAGTISMCFDSF

EQERFDASESIVLIPLPTRSMLFMISAYDSIAMYLAIEPQSLCFYVIAASKRKSEFSTE

AGSKYLILGAFPSGILLFGCSMIYGSTGATHFDQLAKILTGYEITGARSSGIFMGILSI

AVGSLFKITAVPFHMWAPDIYEGSPTPVTAFLSIAPKISISANISRVSIYGSYGATLQQ

IFFFCSIASMILGALAAMAQTKVKRLLAHSSIGHVGYIRTGFSCGTIEGIQSLLIGIFI

YASMTIDAFAIVSALRQTRVKYIADLGALAKTNPISAITFSITMFSYAGIPPLAGFCSK

FYLFFAALGCGAYFLAPVGVVTSVIGRFYYIRLAKRMFFDTPRTWILYEPMDRDKSLLL
AMTSSFITSFFPYPSPLFSVTHQMALSSYL*"

exon 224544..224696
/gene="nad2"
/number=1
exon 225903..226294
/gene="nad2"
/number=2
exon 108724..108884
/gene="nad2"
/number=3
exon 111191..111763
/gene="nad2"
/number=4
exon 113246..113433
/gene="nad2"
/number=5
gene complement(48253..50541)
/gene="nad5"
CDS join(complement(50312..50541),complement(48253..49468),
170325..170346,complement(380..774),
complement(240696..240845))
/gene="nad5"
/exception="trans-splicing"
/product="NADH dehydrogenase subunit 5"
/codon_start=1
/transl_table=11

/translation="MYLLIVFLPLLGSSVAGFFGRFLGSEGTAIITTTCLSFSSIFSLI

AFYEVAPGASACYLRIAPWISSEMFDASWGFLFDSPTVVMLIVVTSISSLVHLYSISYM

SEDPHSPRFMCYLSIPTFFMPMLVTGDNSLQLFLGWEGVGLASYLLIHFWFTRLQADKA

AIKAMLVNRVGDFGLAPGISGCFTLFQTVDFSTIFARASAPRNSWISRNMRLNAITLIC

ILLLIGAVGKSAQIGSHTWSPDAMEGPTPVSALIHAATMVTAGVFMIARCSPLFEYPPT

ALIVITFAGAMTSFLAATTGILQNDLKRVIAYSTCSQLGYMIFACGISNYSVSVFHLMN

HAFFKALLFLSAGSVIHAMSDEQDMRKMGGLASSFPFTYAMMLMGSLSLIGFPFPTGFY

SKDVILELAYTKYTISGNFAFWLGSVSVLFTSYYSFRLLFLTFLVPTNSFGRDILRCHD

APIPMAIPLILLALGSLFVGYLAKDMMIGLGTHFWANSPFVLPKNEILAESEFAAPTIT

KLIPIPFSTSGASVAYNVNPVADQFQRAFQTSTFCNRLYSFFNKRWFFDQVLNDFIVRS

FLRFGYEVSFEALDKGAIEILGPYGISYTFRRLAERISQLQSGFVYHYAFAMLLGSTLF
VTFSRMWDSLSSWVDNRSSFILIVSSFYNNKSSQE*"

exon complement(50312..50541)
/gene="nad5"
/number=1
exon complement(48253..49468)
/gene="nad5"
/number=2
exon 170325..170346
/gene="nad5"
/number=3
exon complement(380..774)
/gene="nad5"
/number=4
exon complement(240696..240845)
/gene="nad5"
/number=5
gene complement(130929..132458)
/gene="atp1"
CDS complement(130929..132458)
/gene="atp1"
/product="ATPase subunit 1"
/codon_start=1
/transl_table=11

/translation="MKNSPRAAELTTLLESRISHFYTNFQVDEIGRVVSVGDGIARVYG

LNEIQAGEMVEFASGVKGIALNLENENVGIVVFGSDTAIKEGDLVKRTGSIVDVPAGKA

MLGRVVDALGVPIDGRGALSDHERRRVEVKAPGIIERKSVHEPMQTGLKAVDSLVPIGR

GQRELIIGDRQTGKTAIAIDTILNQKQMNSRSTSESETLYCVYVAIGQKRSTVAQLVQI

LSEANALEYSILVAATASDPAPLQFLAPYSGCAMGEYFRDNGMHALIIYDDLSKQAVAY

RQMSLLLRRPPGREAFPGDVFYLHSRLLERAAKRSDQTGAGSLTALPVIETQAGDVSAY

IPTNVIPITDGQICSETELFYRGIRPAINVGLSVSRVGSAAQLKAMKQVCGSSKLELAQ

YREVAALAQFGSDLDAATQALLNRGARLTEVPKQPQYAPLPIEKQILVIYAAVNGFCDR

MPLDRISQYERAIPSSVKPELLQKLLEKGGLTNERKMEPDAFLKESALNYL*"

gene 95469..96047
/gene="atp4"
CDS 95469..96047
/gene="atp4"
/product="ATPase subunit 4"
/codon_start=1
/transl_table=11

/translation="MRLSSTNMQARKMLFAAILSICASSSKKISIYNEEMIVARCFIGF

IIFSRKSLGKTFQVTLDGRIQAIQEELQQFPNPNEVVPPESNEQQRLLRSSLRICGTVV

ESLPMARCAPKCEKTVQALLCRNLNVKSATLPNATSSRRIRLQDDLVTGFHFSVSERFV
PGSTLKASIVELIREGLVVLRMVRVGGFS*"

gene 17392..18111
/gene="atp6"
CDS 17392..18111
/gene="atp6"
/product="ATPase subunit 6"
/codon_start=1
/note="stop codon is created by C to U RNA editing"
/exception="RNA editing"
/transl_table=11

/translation="MNIGHLYFSFTNPSLFMLLTLSLVLLLVHFVTKNGGGNSVPNAWQ

SLVELIYDFVPNPVNEQIGGLSGNVKQKFSPRISVTFTFSFFRNPQGMIPYSFTVTSHF

LITLGLSFSIFIGITIVGFQRNGLHFLSFSLPAGVPLPLAPFLVLLELIPHCFRALSSG

IRLFANMMAGHSSVKILSGFAWTMLCMNDLFYFIGDPGPLFIVLALTGPELGVAISQAH
VSTISICIYLNDAINLHQ"
gene 64619..65098
/gene="atp8"
CDS 64619..65098
/gene="atp8"
/product="ATPase subunit 8"
/codon_start=1
/transl_table=11

/translation="MPQLDKFTYFTQFFWSCLFLLTFYIAICNDGDGLLGISRILKLRN

QLVSHQENNIRSKDPNSLEDILRKGFSTGVSYMYSSLFEVSQWCNAVDLLGKRRRIPLI

SCFGEISGSRGMERNIFYLISKSSYSTSSNPGWGITCRNDITLIHVPHGQRSFGF*"
gene 210967..211191
/gene="atp9"
CDS 210967..211191
/gene="atp9"
/product="ATPase subunit 9"
/codon_start=1
/note="stop codon is created by C to U RNA editing"
/exception="RNA editing"
/transl_table=11

/translation="MLEGAKSIGAGAATIALAGAAIGIGNVFSSLIHSVARNPSLAKQL
FGYAILGFALTEAIASFAPMMAFLILSVFR"
gene complement(66878..67498)
/gene="ccmB"
CDS complement(66878..67498)
/gene="ccmB"
/product="cytochrome c biogenesis B"
/codon_start=1
/transl_table=11

/translation="MRRLFLELYHKQIFPSTPITSFSLFLSYIVVTPLMLGFEKDFSCH

SHLGPIRIPPLFPFPSAPFPRNEKEDGTLELYYLSAYCLPKILLLQLVGHWVIQISRVF

RGFPMLQLPYQFGRSGMDRLNIPLGSLILTLLCGIHSRSALGITSSSGWNSSQNPTTSP
TSLPPTLSCTSIETEWFHVLSSIGYSSPFVSLSPISVSISSQD*"
gene 80047..80799
/gene="ccmC"

CDS 80047..80799
/gene="ccmC"
/product="cytochrome c biogenesis C"
/codon_start=1
/transl_table=11

/translation="MSVSLLQPSFLMSKTRSYAQILIGSRLFLTAMAIHLSLRVAPLDL

QQGGNSRIPYVHVPAARMSIIVYIATAINTFLFLLTKHPLFLRSSGTGTEIGAFSTLFT

LVTGGFRGRPMWGTFRVWDARLTSVFISFLIYLGALRFQKLSVEPAPISIRVGPIDIPI

IKSSVNWWNTSHQPGSISRSGTSIHVPMPIPILSNFANSPFSTRILFVLETRLPIPSFL
ESSLTEEIEAREGIPKPSSLAESLCIHG*"
gene 19944..22046
/gene="ccmFC"
CDS join(19944..20710,21497..22046)
/gene="ccmFC"
/product="cytochrome c biogenesis FC"
/codon_start=1
/note="stop codon is created by C to U RNA editing"
/exception="cis-splicing"
/transl_table=11

/translation="MVQLQNFFFFITSMVVPRGTAAPVLLKWFVSRDVPTGAPSSNGTI

IPIPIPSFPLLVYLHSRKFIRSTDGAKSGVLVRASRPILLPYIIGRSSSETRARNALFR

FVPVLHFLLLESKGDFSYLESFCGVLRLLFFRTFFSLPRDRSAKRERARRRKRQTLRPN

GNQQRRNDKMRCPGHPHLERRVEGFGPVAFPGPPSSGGACVGGAQPEIGLEALTLPTSR

QLMAVGHDYYKKAPMKMNISHGGVCICMLGVLLSNTKKIQFTQRLPLGSELHMGKERCC

LRGLDHLHGPTSHSICGNLMIYKPSLTNDRLRFEHDESLRADLLPINFPASYENGKLEH

FLHRWMKNREHNNFWLTMFPEKRYFRETTSTTEVAIHTNLFTDLYAPIGTGSSRTGGWY
TTIMKLPFLFFIRIGFMLASLGGSHSLLRQLQKDKLRWNR"
exon 19944..20710
/gene="ccmFC"
/number=1
exon 21497..22046
/gene="ccmFC"
/number=2

gene complement(85916..87655)
/gene="ccmFN"
CDS complement(85916..87655)
/gene="ccmFN"
/product="cytochrome c biogenesis FN"
/codon_start=1
/transl_table=11

/translation="MSIYELFHYSLFPGLFVAFTYNKKQPPVFGAAPAFWCILLSFLGL

SFRHIPNNLSNYNVLTANAPFFYQISGTWSNHEGSILSWCRILSFYGFLLCYRGRPQSH

NVSKRGGHRETLFYSFVSNFVKNSILSIPRYEKKSRAAPQLYTPFVLRTFVDSELRSRR

NRTFDGPALFYVYAPLYPERKMRFAPLGARRSRGSREGKRMSPLLHLTRDDKERASSID

EQRIDGALGIALFFSPFLSASSDPFVRNLFVRTEPLAESNPVPQDPISAIHPPCIYAGD

VASAMGFGLCRSKMMNGIVALHSPPMRKDAAEKNGTLFRSAGYAGSRITSELFTLKLKH

VGAKCYPALLLRSNRSLLMLLRRRFFAFSSLWTGALVDTGREQAKRVVRNGKKETTTSP

LCWTAGANTVVSDQDQEPIRIWILTCRWFLTVGILPGSWWAHHELGRGGWWFRDPVENA

SFMPRVLATARIHSVILPLLHSWTSFLNIVTLPCCVSGTSSIRSGLLAPVHSFATDDTR

GIFLWRFFLLMTGISMILFSQMKQQASARRTYKKEMVVARSTLVHLRHSARAQPRPVML
WKN*"
gene complement(4174..5361)
/gene="cob"
CDS complement(4174..5361)
/gene="cob"
/product="apocytochrome b"
/codon_start=1
/transl_table=11

/translation="MTIRNLRLLSLLKQPIFSKLNQHLIIYQTPSNLNYWAGFGPLAGI

CLVIQIVTGVFLAMHHTPHVDLAFNSVEHIMRDVKGGWFLRYMHANGASMFLIVVHFHM

FRSLYYGSYNSPREFVRCSGVVIFLLMIVTAFIGYVPPWGQMSFWGATVITSLASAIPV

VGDTIVTWLWGGFSVGNATLNRFFSLHYLLPFLLVGASILHLAALHQYGSNNPLGVNIS

MDKIDSYPYYVVKDYVGWLAFAIFSSIFLFYAPNVLGHPDNYIPANPMPTPPHIVPEWY

FLPIHAILRSIPDKAGGVAAIAPVFICLLALPFLNPRMYVRSSTFRPIYQKIYLLFLAD

RLLLGWIGCQPVEAPFVIIGQISPFVFFLFFAIMPIPGRVGREIPNSYTNETDHT*"
gene complement(215671..217254)
/gene="cox1"
CDS complement(215671..217254)
/gene="cox1"
/product="cytochrome c oxidase subunit 1"
/codon_start=1
/exception="RNA editing"
/note="start codon is not determined"
/transl_table=11

/translation="TTNPVRWLFSTNHKDIGTLYFIFGAIAGVMGTCFSVLIRMELARP

GDQILGGNHQLYNVLITAHAFLMIFFMVMPAMIGGSGNWSVPILIGAPDMAFPRLNNIS

FWLLPPSLLLLLSPALVEVGSGTGWTVYPPLSGITSHSGGAVDSAISSPHLSGVSSILG

SINFITTISNMRGPGMTMHRSPLFVWSVLVTAFPLLLSLPVLAGAITMLLTDRNFNTTF

SDPAGGGDPILYQHLFRFFGHPEVYIPILPGSGIISHIVSTFSGKPVFGYLGMVYAMIS

IGVLGFLVWAHHMFTVGLDVDTRAYFTAATMIIAVPTGIKIFSWIATMWGGSIQYKTPM

LFAVGSIFLFTIGGLTGIVPANSGLDIALHDTYYVVAHFHYVLSMGAVFALFAGFHYWV

GKIFGRTYPETLGQIHFWITFFGVNPTFFPMHFLGLSGMPRRIPDYPDAYAGWNALSSS

GSYISVVGICRFFVVVTITSSSGNNKRCAPSPWAVEQNPTTPEWMVQSPPAFHTFGELP
AIKETKGYVK*"
gene 143762..146060
/gene="cox2"
CDS join(143762..144461,145753..146060)
/gene="cox2"
/product="cytochrome c oxidase subunit 2"
/codon_start=1
/transl_table=11

/translation="MIFLEWLFLTISPCDAAEPWQLGSQDAATPMMQGIIDLHHDIFFF

LILILVFVSRILVRALWHFNYKKNPIPQRIVHGTTIEILRTIFPSIIPMFIAIPSFALL

YSMDEVVVDPAMTIKAIGHQWYWTYEYSDYNSSDEQSLTFDSYTIPEDDPELGQSRLLE

VDNRVVVPAKTHLRIIVTPADVPHSWAVPSSGVKCDAVPGRLNQTSISVQREGVYYGQC

SEICGTNHAFTPIVVEAVPRKDYGSRVSNQLQTGEASTKPLFTASPQEAIIREGVPVPP

EKVWEAVSSGEYYYSSQVHIDGDVKDVETINSLSRLNGVLLYIQEKKVNGAIWA*"

/exception="cis-splicing"

exon 143762..144461

/gene="cox2"

/number=1

exon 145753..146060

/gene="cox2"

/number=2

gene complement(150451..151248)

/gene="cox3"

CDS complement(150451..151248)

/gene="cox3"

/product="cytochrome c oxidase subunit 3"

/codon_start=1

/transl_table=11

/translation="MIESQRHSYHLVDPSPWPISGSLGALATTVGGVMYMHPFQGGATL

LSLGLIFILYTMFVWWRDVLRESTLEGHHTKVVQLGPRYGFILFIVSEVMFLFALFWAS

SHSSLAPTVEIGGIWPPKGIWVLDPREIPFLNTLILPSSGAAVTWAHHAILAGKEKRAV

YALVATVSLALVFTGFQGMEYYQAPSTLSDSIYGSTFFLATGFHGFHVIIGTIFLIICG

IRQYLGHLTKEHHVGFEAAAWYWHFVDVVRLFPFVSIYWWGGI*"

gene complement(51492..53459)

/gene="matR"

CDS complement(51492..53459)

/gene="matR"

/product="maturase R"

/codon_start=1

/transl_table=11

/translation="MKEAIRMVLESIYDPEFPDTSHFRSGRGCHSALRRIKEEWGTSRW

FLEFDIRKCFHTIDRHRLIPIFKEEIDDPKFFYPIHKVFSAGRLVGGEKGPYSVPHSVL

LSALPGNIYLHKLDQEIGRIRQKYEIPIVQRIRSVLLRTGRIDDQENSGEEASFNAPQD

NRAFIVGRVKSIQRKSAFHSLVSSWHTPPTSTPRLRGDQKPPFVFPPSSALAAFLNKPS

SLLCAAFLIEAAGLTPKAEFYGRERCNNNWAMIYFFKYCKRKGLLIEPGGEAILVIRSE

RGLARKLAPLKSHYLIRICYARYADDSLLGIVGAVELIIEIQKRIAHFLQSGLNLWVGS

AESTTIAARSTVEFLGTVIREVPPRTTPIQFLRELEKRLRVKHRIHRTACHLRSAIHSK

FRNLGKSIPIKELTKGMSGTGSLLDAVQLAETLGTAGVRSPQVSVLWGTVKHIRQGSRG

ISLLHSSGRSKVPSDVQQAVSRSGMSVRKLSLYTPAGRKAAGEGGGHWARSISSEFPIQ

IEAPIKKILRRLRDRGLISRRRPWPIHVACLTNVSDGDIVNWSAGIAISPLSYYRCRDN

LYQVRTIVDHQIRWSAIFTPAHKHKSSARNIIPKYSKDSNIVNQEGGKTLAEFPNSIEL
GKLGPGQDPNNKEHSTTSLV*"

gene 212375..212749
/gene="mttB"

CDS 212375..212749
/gene="mttB"
/product="transport membrane protein"
/codon_start=1
/transl_table=11

/translation="MGATSTNSLMIKLQPKIYDYIMLTVRISFIPSVCSQVPVIVIRLP

EPRGLSVETSTNNRRFLMVFPLLTAALSTPPDIWCQIVSRFLISLIIELAIFVASIVQV
REEGWTSGMRESGSIDKKEE*"

gene 172680..173036
/gene="nad3"

CDS 172680..173036
/gene="nad3"
/product="NADH dehydrogenase subunit 3"
/codon_start=1
/transl_table=11

/translation="MSEFAPICIYLVISPLVSLIPLGVPFLFASNSSTYPDKLSAYECG

FDPSGDARSRFDIRFYLVSILFIIPDPEVTFSFPWAVPPNKIDPFGSWSMMAFLLILTI
GSLYEWKRGALDRE*"

gene complement(34528..43300)

/gene="nad4"
CDS complement(join(34528..34616,37366..37788,40916..41430,
42840..43300))
/gene="nad4"
/product="NADH dehydrogenase subunit 4"
/codon_start=1
/transl_table=11

/translation="MLEHFCECYSDLSGPILCPVLGSITPLFIPNSRIRPIRLIGLCAS

LITFLYSPVLRIQFDPSTAKSQFVESLRWLPYENINFYLGIDGISLFFVILTTFLIPIC

ILVGWSGMRSYGKEYITASLIREFLMIAVFRMLDPLLFYVLPESVPIPMFIIIGVWGSR

QRKIKAAYQFFLYTLLGSVFMLLAILLILLQTGTTDLQISLTTEFSERRQIFLWIASFA

SFAVKVPMVPVHIWSPEAHVEAPTAGSVILAGIPLKLGTYGFLRFSIPMFPEATLCSTP

FIYTPSAIAIIYTSSTTLRQIDLKKIIAYSPVAHMNLVTIGMFSPNIQGIGGSIPPMSS

HGLVPSALFLCVGVLYDRHKTRLVRYYGGSVSTMPNLPTIFFSSTLANMSSPGTSSFIG

EFLILVGAFQRNSLVATLAALGMILGAAYSLWLYNRAVSGNLKPDFLHKFSDPNGREVS
IFIPFLVGVVRMGVHPKVFPDRMHTSVSNLVQHGQFH*"
/exception="cis-splicing"
exon complement(42840..43300)
/gene="nad4"
/number=1
exon complement(40916..41430)
/gene="nad4"
/number=2
exon complement(37366..37788)
/gene="nad4"
/number=3
exon complement(34528..34616)
/gene="nad4"
/number=4
gene 117460..117762
/gene="nad4L"
CDS 117460..117762
/gene="nad4L"
/product="NADH dehydrogenase subunit 4L"
/codon_start=1
/exception="RNA editing"

/note="start codon is not determined"
/transl_table=11

/translation="TDPIKYFTFSMIISISGIRGILLNRRNIPIMSMPIESMLLAVNSN

FLVFSVSSDDMMGQSFASLVPTVAAAESAIGLAIFVITFRVRGTIAVESINSIQG*"
gene 232773..239068
/gene="nad7"
CDS join(232773..232915,233827..233895,235240..235706,
236765..237008,238807..239068)
/gene="nad7"
/product="NADH dehydrogenase subunit 7"
/codon_start=1
/transl_table=11

/translation="MTTRNGQIKNFTSNFGPQHPAAHGVSRSVLEMNGEVVERAEPHIG

SLHRGTEKLIEYKTYLQALPYSDRLDYVSTMAQEHAHSSAVERLLNCEVPLRAQYIRVL

FREITRISNHSLALTTHAMDVGASTPFLWAFEEREKLLEFYERVSGARMHASFIRPGGV

AQDLPLGLCRDIDSSTQQFASRIDELEEMSTGNRIWKQRLVDIGTVTAQQAKDWGFSGV

MLRGPGVCWDSRRAAPYDVHDQSDPDVPVGTRGDRYDRYCIRIEEMRQSVRIIVQCPNQ

MPSGMIKADDRKLCPPSRCRMKLSMESSIHHFEPYTEGFSVPAPSTYTAVEAPKGEFGV

FLVSNGSNRPYRRKIRAPGSAHSQGLDSMSKHHMPADVVTIIGTQDIVSGEVDR*"
/exception="cis-splicing"
exon 232773..232915
/gene="nad7"
/number=1
exon 233827..233895
/gene="nad7"
/number=2
exon 235240..235706
/gene="nad7"
/number=3
exon 236765..237008
/gene="nad7"
/number=4
exon 238807..239068
/gene="nad7"
/number=5

gene 209358..209933
/gene="nad9"
CDS 209358..209933
/gene="nad9"
/product="NADH dehydrogenase subunit 9"
/codon_start=1
/transl_table=11

/translation="MDNQFIFKYSWETLPKKWVKKMERSEHGNRSDTNTDYPFPLLCFL

KLHTYTRVQVSIDICGVDHPSRKQRFEVVYNLLSTRYNSRIRVQTSADEVTRISPVVSL

FSSAGRWEREVWDMFGVSSINHPDLRRISTDYGFEGHPLRKDLPLSGYVEVRYDDPEKR
VVSEPIEMTQEFRYFDSASPWEQRSDGE*"
gene complement(44154..44648)
/gene="rpl10"
CDS complement(44154..44648)
/gene="rpl10"
/product="ribosomal protein L10"
/codon_start=1
/transl_table=11

/translation="MPFGRSIIQRESLLRVSGEERSPEILISFHSSGSTSNQWRKLKNP

WFPGRTLFRPSCFGTGKKKRFFAQLAHSAGPTCISYLAEEASDRLEFLPSWDSMDQDLL

LLYGQYRSTFTLVDHMDVEKTSHFDELETSLFNFYLPSSYLCFVCSREEFDLFNLGIPP
K*"
gene complement(136751..137185)
/gene="rpl16"
CDS complement(136751..137185)
/gene="rpl16"
/product="ribosomal protein L16"
/codon_start=1
/note="start codon is not determined"
/transl_table=11

/translation="VLYPKRTKYSKYRKGRCSRGCKPDGTQLGFGRYGTKSCRAGRLSY

RAIEAARRAIIGQFHRAMSGQFRKNGKIWVRVLADLPITGKPTEVRMGRGKGNPTGWIA
RVSTGQILFEMDGVSLSNARQAATLAAHKPCSSTKFVQWS*"
gene complement(46232..46297)
/gene="rpl16"
CDS complement(46232..46297)

/gene="rpl16"
/product="ribosomal protein L16"
/codon_start=1
/note="start codon is not determined"
/transl_table=11
/translation="PEDWVSVVKPGRILSEMGGVS*"
gene 166931..167491
/gene="rpl5"
CDS 166931..167491
/gene="rpl5"
/product="ribosomal protein L5"
/codon_start=1
/transl_table=11

/translation="MFPLHFHYEDVSRQDPLLKPNHANVMEVPGSCKIRVVPKAAPYDF

IIKNGKLAMEIPRGQKLIQKKRASTGKSFRSNPFLGSNKGSVSDLARQSTLRGHGMYHF

LFRISTVMSLLDSPVEIRENSIQFSMETEFCELSPELEEHFEIFEHIRGFNVTIVTSAN
TQDDTLPLWSGFLQKEEGESKSK*"
gene 171755..172360
/gene="rps1"
CDS 171755..172360
/gene="rps1"
/product="ribosomal protein S1"
/codon_start=1
/transl_table=11

/translation="MTIYLSRSFPRSNSSFFLCSGNALKSEVLRLREEILLMDAGPGTP

RICMQDELTGVSINRATRFENKVGSLDLVAGESLIKEHILERFFIDLVAGESLIKERAA

ARFKKLVGSTNVVAGEPLLLLPRRFRKNRAWMELNKIWRTNTKVKGLICRKIKGGYSVA
IAGFLTFLPFRRSRKRKSFPRFTIESINPKKVVTIVVF*"
gene 168852..170010
/gene="rps10"
CDS join(168852..169101,169910..170010)
/gene="rps10"
/product="ribosomal protein S10"
/codon_start=1
/exception="cis-splicing"
/note="start codon is not determined"
/transl_table=11

/translation="TTTKIRIVIRSFDHPFFENPFWGLPPYTRKIGLPESRVLYTVLRS

PHIDKKSREQFEMEIKKQFLVIKTERHELRKKFFRLKRQRIFGAQYEILFSCKTRSDKG
KQRLFQKGARAY*"
exon 168852..169101
/gene="rps10"
/number=1
exon 169910..170010
/gene="rps10"
/number=2
gene 173085..173462
/gene="rps12"
CDS 173085..173462
/gene="rps12"
/product="ribosomal protein S12"
/codon_start=1
/transl_table=11

/translation="MPTLNQLIRHGREEKRRTDRTRASDQCPQKQGVRPRVPTRTPKKP

NSAPRKIAKVRLSNRHDIFAHIPGEGHNSQEHSMVLIRGGRVKDSPGVKSHCIRGVKDL
LGIPDRRRGRSKYGAEKPKSI*"
gene 57443..57793
/gene="rps13"
CDS 57443..57793
/gene="rps13"
/product="ribosomal protein S13"
/codon_start=1
/transl_table=11

/translation="MCYISGARSVADEQVRIASTKIDGIGPKKAIQVRYRLGISGNIKI

KELTKYQIDQMEQMIGQDHVVHWELKRGERADIERFISLSCYRGIRHQDGSPLRGQRTH
TNARTCRKQIRK*"
gene 167493..167795
/gene="rps14"
CDS 167493..167795
/gene="rps14"
/product="ribosomal protein S14"
/codon_start=1
/transl_table=11

/translation="MSEKRNIRDHKRRLLAAKYELRRKLYKAFCKDPDLSSEMRDKHRY

KLSKLPRNSSLARVRNRCISTGRPRSVYELFRISRIVFRGLASRGPLMGIKKASW*"
gene complement(137157..140622)
/gene="rps3"
CDS complement(join(137157..138777,140549..140622))
/gene="rps3"
/product="ribosomal protein S3"
/codon_start=1
/note="start codon is not determined"
/transl_table=11

/translation="IARKGNPISVRLDKNRSSDSSRFSDYYYGKSVYQDVNMRSYFGSI

RPPTRLTFGFRLGRCLILHFPKRTFIHFFLPRRPRRLKRREKSRPGKKKKKGRWWAFGK

VGPIGCLHSSDDTEKERNEVRGRGTGKRVESIRLDDRKKQNEIRIWPKKKQRYGYHDRS

PSIKKNLSKSLRVSGAFKHPKYAGVVNDIAFLIKNDYSFRKTKFLKFFFPKKFRSDSPT

SHLLKMKRTLPAVRPSLNFSVMQYLLNTKKKMHFDPVVVLNHFLASGVAEPSTMGGANA

QGRSLDKRIRSRIAFFVESSTGDKKYLAEAKKRLTHFIRLANNLRFAGTTKTTISLFPF

FGATFFFPRDGVGVYKNLEDAREPLLGKLRKKCWNLMGKDKVMELIEKFIDLGGIGELR

KGIEMMLEIILRNRIIPYGYNSYLNEVKKMRSLLFNRTNTNTLIESVKIKSVYQSASPI

AQDISFQPRNKTRSFRSIFSQIVKDIPLVMKKGVEGIRICCSGRSKGAEIARTECGKYG
KTSRNVFNQKIDYAPAEVSTRYGISGVKVWISYKKKKGRAISETYKI*"
/exception="cis-splicing"
exon complement(140549..140622)
/gene="rps3"
/number=1
exon complement(137157..138777)
/gene="rps3"
/number=2
gene 118047..119093
/gene="rps4"
CDS 118047..119093
/gene="rps4"
/product="ribosomal protein S4"
/codon_start=1
/transl_table=11

/translation="MPSLRFQTCRLLSGNVWNRELTIIQRHILQRLRNKKRSIKRKIYS

RENLNSYIQSQTTRKLPLFYGDLPITEMHRGRERTSYIPFPLNPETRSDVLPVRLHFRE

TIPQARQPISHRRVCVNNGMVSITHFKVSHGDIISFQENDARARGEEIRRSFYIEILVE

KIIGKFRDHPVRMWRRTKTEWFRLLKTQRGCRLLLKDRFLQQLRSSMQEEYLERTKKFG

SKKVCLGSSFAEHNRIKRNLYHFKSLFLSNRRNEKNRNLPTRTRSPIVYNSSFYSNSTY

CSAPPHQFTMKRKRKRIELLTHYSEVNHRTLKAVVSYGPNIGHIPHDIRLKDPNLLLRS
GNGRGQNI*"
gene complement(31939..32385)
/gene="rps7"
CDS complement(31939..32385)
/gene="rps7"
/product="ribosomal protein S7"
/codon_start=1
/transl_table=11

/translation="MGGLDGEQKQLIKKLVNFRMKEGKKTRVRAIVYQTFHRPARTERD

VIKLMVDALENIKPICEVERVGRAGTIYDVPGIVARDRQQTLAIRWILEAAFKRRISYR
ISLEQCSFDEILDAYRKRGIARKKRENLHRLASTNRSFAHFRWW*"
gene complement(194924..196859)
/gene="rrn18"
rRNA complement(194924..196859)
/gene="rrn18"
/product="18S ribosomal RNA"
gene complement(7543..9478)
/gene="rrn18"
rRNA complement(7543..9478)
/gene="rrn18"
/product="18S ribosomal RNA"
gene complement(194682..194799)
/gene="rrn5"
rRNA complement(194682..194799)
/gene="rrn5"
/product="5S ribosomal RNA"
gene complement(7301..7418)
/gene="rrn5"
rRNA complement(7301..7418)
/gene="rrn5"

/product="5S ribosomal RNA"
gene complement(149969..150112)
/gene="sdh4"
CDS complement(149969..150112)
/gene="sdh4"
/codon_start=1
/product="succinate dehydrogenase subunit 4"
/note="start codon is not determined"
/transl_table=11

/translation="SCSPRNDPKLDLGLFEIVPLNRNQRCFLVSPFFSEQIEEPNGSNS
SL*"
gene complement(150272..150523)
/gene="sdh4"
CDS complement(150272..150523)
/gene="sdh4"
/codon_start=1
/product="succinate dehydrogenase subunit 4"
/transl_table=11

/translation="MVLAFCRRGSVIPICLYLLVGRYMKKRNNRLKEARRQREERGLFQ
RITAAFPLPFSSCSQEMRYRRKFLFFVITELKRSSGLS*"
gene 104836..104906
/gene="trnC-GCA"
tRNA 104836..104906
/gene="trnC-GCA"
/product="tRNA-Cys"
/note="mitochondrion-native"
gene complement(203762..203835)
/gene="trnD-GUC"
tRNA complement(203762..203835)
/gene="trnD-GUC"
/product="tRNA-Asp"
/note="plastid-derived"
gene 124503..124574
/gene="trnE-UUC"
tRNA 124503..124574
/gene="trnE-UUC"
/product="tRNA-Glu"
/note="mitochondrion-native"
gene complement(160791..160864)
/gene="trnF-GAA"
tRNA complement(160791..160864)
/gene="trnF-GAA"

/product="tRNA-Phe"
/note="mitochondrion-native"

gene 24997..25068
/gene="trnG-GCC"

tRNA 24997..25068
/gene="trnG-GCC"
/product="tRNA-Gly"
/note="mitochondrion-native"

gene complement(122442..122515)
/gene="trnH-GUG"

tRNA complement(122442..122515)
/gene="trnH-GUG"
/product="tRNA-His"
/note="plastid-derived"

gene 78713..78785
/gene="trnK-UUU"

tRNA 78713..78785
/gene="trnK-UUU"
/product="tRNA-Lys"
/note="mitochondrion-native"

gene 82949..83025
/gene="trnI-CAU"

tRNA 82949..83025
/gene="trnI-CAU"
/product="tRNA-Met"
/note="mitochondrion-native"

gene complement(100903..100975)
/gene="trnM-CAU"

tRNA complement(100903..100975)
/gene="trnM-CAU"
/product="tRNA-Met"
/note="plastid-derived"

gene complement(32857..32929)
/gene="trnfM-CAU"

tRNA complement(32857..32929)
/gene="trnfM-CAU"
/product="tRNA-Met"
/note="mitochondrion-native"

gene 106984..107055
/gene="trnN-GUU"

tRNA 106984..107055
/gene="trnN-GUU"
/product="tRNA-Asn"
/note="plastid-derived"

gene complement(160476..160550)
/gene="trnP-UGG"
tRNA complement(160476..160550)
/gene="trnP-UGG"
/product="tRNA-Pro"
/note="mitochondrion-native"
gene 210537..210610
/gene="trnP-UGG"
tRNA 210537..210610
/gene="trnP-UGG"
/product="tRNA-Pro"
/note="plastid-derived"
gene 27463..27534
/gene="trnQ-UUG"
tRNA 27463..27534
/gene="trnQ-UUG"
/product="tRNA-Gln"
/note="mitochondrion-native"
gene complement(161112..161199)
/gene="trnS-GCU"
tRNA complement(161112..161199)
/gene="trnS-GCU"
/product="tRNA-Ser"
/note="mitochondrion-native"
gene 90667..90753
/gene="trnS-UGA"
tRNA 90667..90753
/gene="trnS-UGA"
/product="tRNA-Ser"
/note="mitochondrion-native"
gene 210771..210844
/gene="trnW-CCA"
tRNA 210771..210844
/gene="trnW-CCA"
/product="tRNA-Trp"
/note="plastid-derived"
gene 108108..108190
/gene="trnY-GUA"
tRNA 108108..108190
/gene="trnY-GUA"
/product="tRNA-Tyr"
/note="mitochondrion-native"
ORIGIN
1 ctctaataac gaagaaggtc agctttgaaa ctggctgctc tgctatacta tactagttgt

61 aggtcgctag cgcttgacta atagaatctc aagtataggg actctatcat gatcttacga
121 agctaaagat ctaaaaatgg aagaacgagc tcttttgctc gcccctatct ctaaaggggc
181 gtaagtactt cactcgctag gggatgggat tcattcactt gcattcctgc tagcactaaa
241 aaaagctccg gtcttaacgc ccctactact gctgtgcagc ctttcctcgg gttcgtagag
301 tcgggtttcc cgtttaccca caacggagga gccgccccca ccaggcaggc ggccacgggt
361 cataacgcac tcttcgcaca acaaatccac tttgaagttg acttattcgc tcggccaatc
421 gtcggaatgt gtacgagata ccataagggc ccaatatctc aatagcacct ttgtctaaag
481 cttcgaatga gacttcatat ccgaaacgca ggaacgatct gactataaag tcattcaaaa
541 cttgatcgaa gaaccagcgt ttattgaaga agctatagag tcgattacaa aaagtactag
601 tttgaaaggc tcgttggaat tgatccgcta cgggatttac attatacgca acagaagcac
661 ctgaagtact aaacggaata ggtattagtt tggtaatggt tggagcagca aactcggatt
721 cggcaagaat ctcatttttt ggtagtacga agggggaatt ggcccaaaaa tgggataggg
781 gtcaagacgc tgtcgggcgc cggcggctga ctcaagtgcc ccccgaaccg cgcgaaatgg
841 tcgcctatta cacggctcac gaactctgcc tggggtgggg gtacctattc ttcgtcggcg
901 gtccggcgca cccctactat acaaagccgc cccccaactc acttaaagga tgctgcttaa
961 ttacgaaggc aatttttat taatctagag agttcctttt cgttaagcgc atcgagccag
1021 gagtggacat catcatcatc atctgcagac aaatagaaat tctgtttcat cattagctaa
1081 agtggaatag tatgactggc tttccggaaa agaagacgaa acccgcttta tcgatatgat
1141 aaacatgcgc tcaaaaagac ctcctaaacc cccccagcca agtgatcgag tttagtctga
1201 atttgctgta gaagttgctc ccgctccgga ctgacttctt tgttttcact aactcacttt
1261 ctgattacga gtttatcagt taagggggga ctcgttgacc ttttcctttt ttgtagaggc
1321 ctcggcgcag caatctactc cttccgttta catatgtgat gtaataaggc ttgtcgagct
1381 gatctcatcc cctctacttc aggcctatct gcatttgttg cttagtggca atcccccatct
1441 tttcttgtaa ctgactgact taaagagtct ttctttgaaa ccagttcggt tctgttaact
1501 cacttgtaag ctgtctttcg atcataaata ggtttcacat ctgacatata tgtgttaagc
1561 atatagcctg atctcttttc ttacgctatt taaagtgtct agttgtggtg cttagaatcg
1621 attctttctt cttcaatagt aagaaaagtt tttcttagat tgcccggtga ccggcttagg
1681 ctttgattga agttcccagc caaggtaaat aggaaggagg agatcccacc acccaatgga
1741 ctactagctt aacccgagaa agacaaagac aggcttttga tccatttcac tagctgtagg
1801 actggagcct aaagtgcttc gagaggaaag tatgaatcga ttctgctttc ttcactcgct
1861 tcttctcttt tatctattga agacttatcg taggagattt caagttaggg ataggaatcg
1921 gtagcatgaa cagctgattc agcaagagct caaaaaacct agttttcacc ataaaaagaa
1981 aaatgggctt tttagaaaat ccaattggta agactatact ctcaatccct tcttttacgg
2041 aaagatgggc cttttctcga aaactccttc ttccgacttg gttggtatat aggcgggtga
2101 agccctttca tttcatgctt agaggaatgg gaatagtgtg gttgtgctta cgagaatgta
2161 aagaaagcta cagtcatcct ttagtcagga ggcactcaac gctggcttaa gacctgcgta
2221 agttttgcaa ttggaataca agaggagata ggggctttcg gattcaactc agagccagga
2281 agagtcgcta agaaaatagc caagataaaa agagtcctca cgagtttctg ttttgagatc
2341 ggagaaaaga gttccttcct caagttcttt actcaatgcc aggcgattcg ctaaccggtc
2401 cccttactta gcttagaagg tcagggagac aaggtcaagg taaggatgga ctaatgagtt
2461 ttcccccctt taaagtaaat ttactgctat tcaatagtgg ttaactagct gctcacttga
2521 acaaccaaac tggttttctt tcaaatctaa agtagccacg agtggagtta gccaattcta
2581 tttctatcca ttacgggcat aagttattaa atcggcttgt tttataagaa gctgtctcca
2641 gttagccagc cagcaggagc tcattcatga ctatcaggaa tcggtcaatc ttgctgccac

2701 acttcctcca agggggggag ggcataggaa agcgactaca ccaccattgt ttgagcttat
2761 atcgatcgtt ccccaccaca ggtttctcct gcgatttctc tttactgaac ctggccaaag
2821 gattcgtttt ttccaatgta tatgtcttag ttgacaaagc tgtttacgta ataggctgct
2881 ggctagaaag ataggaattt ggaatgttga gatgactggt ttatcgatac actcgagtag
2941 atgctttcaa caagccccac tcacagatag ccaatgagct tgctctttta aaaagaaacc
3001 cgcaatactg gatgcgatgt ttggaacaaa aagaccacca aaggttcccg aaatgttcgc
3061 aaatgacttg tcatatggcc gtacagaaga acaagcagac atggaccatt tataaaaaga
3121 aaatgatttc ttagacccgg gttcactcca aaaatggaag agcttagcaa agaaaagaaa
3181 gaataaaaag ggtgacccga agcctccgtc ttggtaatga aaaaaagatc cctagcggtt
3241 gttctgacat gtgattaacc gaatcagctg cgtagccctc tttcacaggg gaggtgtggc
3301 ggttaggcat agcctgcttc cagaaagtca ttcatgttct ttattctctt tgacctttac
3361 ctagcccttg ctcgttctcg tttgacacct ttactggcta actaagagac tagtcctctt
3421 cctggcttga aaagtaagta ggtggggtaa acattctaaa gttcaaagag aaaagtgccc
3481 gcttaatccg caaaggaaat aggagaacct tagtccaggt gatagtacac tccatatatc
3541 ccattttgaa tagtgcttcc tgccgggacg aggaagaaag aaattctccg gaaggtaagc
3601 cacatacctg tctatttcac ctatgctttt cttatgttat gtatagccta gacttagaag
3661 tataaggcgc tagaagtagt atgaatagtt ggattcctta ctaggctttt ctcccttttc
3721 taggaactag tcaattaagg tagggttctt tttagtctat gtctaccctt cttttctgat
3781 ctatctgctt gtgctctata aaatagataa gataataggt tccccccagc cctaatagaa
3841 tgttctcctt ttcgatctaa atcgagctaa tagttagagc tatcttttct cgataagtgt
3901 gtttgacaaa gaatgacaag tgcatttaat gaagtaatca tacgagatca gtcctatctt
3961 ttcgtaatga tatggtcttt accttcggga ttatagcatt tttaagagga gcggtgattt
4021 gttacatcga tcatttcttt tggttcctta agtggatagt acttttttcc ctatctaagc
4081 tatatcaaca tagcccacta tcaaactcat ccgcttgtcc attcttggtg taatactcac
4141 tcgtaaattc ttggtgtcaa aatttttaac ttttcaggtg tgatcagtct cattcgtgta
4201 agaattagga atttctcttc caactcgtcc cggaatgggc attatggcaa agaacaagaa
4261 gaagacaaaa ggagaaattt gtccaataat cacaaatggt gcctccacag gttgacatcc
4321 gatccaacct agtagtaagc gatccgccaa aaacaacaaa tatatttttt ggtaaatcgg
4381 gcgaaacgtt gagctacgta catacatcct tggatttaaa aagggtaaag ccaacagaca
4441 tataaaaact ggtgctattg cggctacacc tcccgctttg tcaggtatac tacgaagaat
4501 ggcatggatc ggtaggaaat accattccgg cacaatatga ggcggggtgg gcatcggatt
4561 agcaggtata taattgtcgg gatgccccaa aacattagga gcataaaaaa gaaaaatgga
4621 agaaaagata gcaaaagcta accaacctac gtaatccttt actacataat aagggtaaga
4681 atcaatttta tccatcgaga tatttacacc caatggatta tttgatccat attgatgcaa
4741 cgcggccaga tgaagaatac tggcgcctac taaaagaaag gggagtaaat aatgaagact
4801 aaaaaaacga tttaaggtgg cattgcccac ggagaaacca ccccaaagcc aagtcactat
4861 ggtatctcct actacaggta tggcgctagc taagcttgta attactgtag ctccccaaaa
4921 gctcatctga ccccaaggtg gtacgtatcc tataaaagct gtaacaatca ttaataggaa
4981 gattacaact ccgctacacc gaacaaattc cctaggactg ttataactcc cataatatag
5041 actacgaaac atatgaaagt gaaccacaat gagaaacata cttgccccat tagcatgcat
5101 ataacggaga aaccagcccc ctttaacatc tctcataatg tgttctacgc tgttgaaagc
5161 tagatccaca tgaggtgtgt gatgcatagc taaaaaaacg ccagtcacta tctgaatgac
5221 taaacaaata ccagctaacg gaccgaaccc cgcccaataa ttaagattgc tcggggtttg
5281 ataaattatc aaatgctgat tcagtttgga gaatataggt tgtttaagaa gagataataa

5341 tcgtaggttc cttatagtca tttaattcta tttttagact tttctatcgt gacaactctt
5401 ggggatgaac atcttctgta aggtaagcct gatatgaaaa atgttgccct ccgctaccgc
5461 tctgagagga aagaaagcat cggtgcgata cctatcagac atacggaatt atccatctta
5521 cctttttga aagactttcg cctttaaggt aaggtaatcg tcggacaatg gtccgtagtt
5581 ctctaggttt gataagatat gggaaagatg ctccaaggac tcttccccac ttgaggtccg
5641 gggattatcg agggctctcg ctccacgccc tatccaatcc ccagttgggt cccattgagc
5701 catctttttt attatttgca ccttgatttc gaatagatct tgggcttcca agtggctaag
5761 ataaattcta tccgcagagg gataggggtc ttttgccaaa aggcgccttt gaatagcgta
5821 aacgctatct cccccaattt cttgcatatc agaatcagaa tctttttcga cggggcgagg
5881 ctgaggaggg gctgctggtt gtccttcctg atgcgcttgt ttggcatctt ttaaaaaagg
5941 ttcaacggca gccgataagt catcaacccg cctcggattg ctactcccct cacccgtggt
6001 atccatccga aggggctcat aaaaagtctc tccccgagaa tcctcctgcg ggctcactgc
6061 aggagaaacg ctctttgaaa ggtaaatgaa tgcttctcct ctgtctcatg accggggttt
6121 ttccaccccg agcacacctg agctcctttt agctagcgcc ttctgtttgc ctgtctttca
6181 cgtatagtag tgagaaaggg gacccctgag ccaggttaac tatgcctagc atataccgga
6241 gccctcattt tgaggtttca ctccgacctt ctcctagttc ggaaactaca cgaggtgatc
6301 tcgaccctca ctataagata atgatttcat tgtatcctga aagtgaattg aaagtccact
6361 tcgagtaagg gaataggaca ggtgtgacat acgcccggtc tctcgaccgg gggtgaaaag
6421 acaatcaatc cgctttttc gtttgcacaa gttaaggaag ttgtatagcc agccaaaggt
6481 aaaggtcctg tggtctagtg gtccaaaagg tcaaccagtc atcttagaag cagtagctct
6541 aacagttcct ccggttagct tagagctcct agcttgactt cgctcagcta gtgcttctcc
6601 ttgctaagct taagccagtt tcattctttc tttcacccag taagtgctat cttgaagaag
6661 gaaaagttct gtcttggtcc gagtggggat agcgaaaact cgcaacttcc ccgattcaat
6721 tactttctgc ggctgatgcc tcttcccccg ctttaagttc agttccggtt agccctactt
6781 atggatcgga ttagtcgcca aaaaaaggaa tctatttact aggaaaaaag tagcgcttag
6841 agttcggtgg aaggataaac cattcccgta tggagccagc ctcccttaaa ggaatgtcta
6901 tatggggtca ccactcttat cagtagaaga cagagtggga agcagcggaa catgaagcca
6961 atcgataaag aaaggtgtag tccgtccttt ctttgtccga tgggaatgaa agaagggcag
7021 ctttctcaga ctcctaagag gccaccagag actacgatat agaccaggat ctatcagccg
7081 cttgtccagg cggatcggta aataaagagg aaaaagggcg cagcctcatc ttctgactgg
7141 ccagcgggga agactctgac tcagaacttg cgctagcccc tttcattgat tcgggggaca
7201 agtagaattc atcaacaact gggcttaccg actctacttc aacttcactt cctgaagcag
7261 atggaagtac gctcacgatc agaatctttg cttcatttct tcaccgggct tggaccatgt
7321 ctcccgaaca atctcagtac atatggcgca agacgattcc acatatcgag gtcggaatgg
7381 gatcgggtgt tttcacgtct caccgtagtg cccggtttgt cttgatttcc gattgatgaa
7441 caagaaggaa aatggaactc ttggattttg tgactcgccc gcccgccacc agtggaagca
7501 agctagcccc cggttggggg aagagggcat ttccatcgcg aaggattcaa tccagccaca
7561 ggttccccta cggctacctt gttacgactt caccccagtc gaagacccca ccgtggtatg
7621 cgccaataag accaccaaaa gcctttgtgg cactagtggt acacagaagt catgggtgat
7681 cattggtccg atgcttcggg cgaaaccaat tcccagggtg tgacgggcgg tgtgtacagg
7741 gcccgggtac atattcaccg cggcatgctg atccgcgatt actagcgatt ccaacttcat
7801 gttcccgagt tgcagagaac aatccgaact gaggcaatcg ttccggattc gctccgcctt
7861 acagccttgc ttcccattgt aattgccatt gtagcacgtg tgtggcccag cccataaggg
7921 ccatgcggac ttgacgtcat ccccaccttc ctccagtata tcactggcag tccttcgtga

7981 gtgcggcacg caccttttg tttgtttcgg agccgttttg gcggggcgta ctaaacccac
8041 tacgtaccac accaccgggc ggctcgccgg aatgccgagt ctttctctgc cgtcaactcg
8101 acgtcgtcgt cacctgcaag agaaggccaa aaacttgact ttactaaaca agcgagaaaa
8161 gccctttcta tcttattagt caagcgcgct agctgcaatc aaactaaagc gcacactaga
8221 aagtgcttcg aaaggcgccg gctaccttct tactgacagc acagctacgt gctggcacta
8281 aattagtagc gctggcacgt aactcggctc ctcggctcac ttcggttgca aagactttct
8341 ccttaggcgc atgtctcagc aacacaaaac gagggtttcg ctcgttatag gacttgacca
8401 aacatctcac gacacgagct gacgacagcc atgcagcacc tgtatgaaag taagtaccat
8461 cccattaagg acaggttttg ttgttcatat gtcaagggct ggtaaggttt tgcgcgttgt
8521 atcgaattaa accacatgct ccaccgcttg tgcaggcccc cgtcaattcc tttgagtttc
8581 ggtcttgcga ccgtactccc caggcggagt gtttcacgcg ttagctgggc ccctgatctg
8641 cgtagaccaa gggcgaacac tcatcgttta cggcatggac taccagggta tctaatcccg
8701 ttcgctcccc atgctttcgc accccagcgt cggtagggac ccagagagct gccttcgctt
8761 ttggcgttcc ttcgtagatc tccggatttc acccctacac acgaaattcc actctcctct
8821 gtctcactca agtgaattgg tttcgagagc attccgccag tttttggcga ctttcacttt
8881 caacccgatt caccgcctac gtgcccttta cgcccagtca ttccgaagaa cacttgcccc
8941 ccccgtctta ccgcggctgc tggcacggag ttagccgggg cttcttcctc gagtcctgtc
9001 atgatcgcgc actcgacgaa agagctttac aagcggcatt gcccttcttc actcacgcga
9061 tattgctgga tcgggctttc gcccattgtc caagattccc cactgctgcc ccccgtggga
9121 gtccgggccg tgtctcagtc ccagtgtggc tgatcatccg aaaagaccag ctaagcatca
9181 ttggcttggt cagcctttac ctgaccaact acctaatact acgcaggctc atcaaacagc
9241 gctttttagc tttcttcagg atttggcccg aactgttcgg cagattccca cgcgttacgc
9301 acccgttcgc cactttgttc tcaactcttc tcacctcctg ggcgagacaa gctaccttta
9361 gctaggagcc tcttttcctt ctgtctagct ccccgaaaac aacgttcgac ttgcatgtgt
9421 taagcatata gctagcgttc cttctgagcc aggatcaaac tcttcttttg agtatgattt
9481 ggcccttagt ggtagaacct ggtgaaccgg gcgtagtact tcccgacctt ctgtgaactt
9541 tgcttctctt atgtaattgt aataagagaa tctttcgaaa agtcttcttt ctctatgatg
9601 attctgattc gactaggctc actaggtgac tcctgtatgc ggcgagacta gagacacgaa
9661 gtaaaatgtt cgaaccgaat ggtatccaag ggaaatgaac aacctaaggg agcgccatct
9721 ttctattagg agattggccc caagtctgac cctcgccatt actcttaagg ttacagcctc
9781 ttactcaatt gattagttct accaccccaa gggtttagcg tggaccaagt gtatttgttg
9841 aaaccataaa gaaatgcgcg gaagagctct tgacttattg tatgcacttt actgatgagc
9901 gaaggggctt tcaaggaagg ctattcaata gggtcaaccg gagatcgact gagttggaga
9961 tgaagaattc agcgccctcc cgatcggtct cgcgatcagt tgagatgttt ttaaggcgct
10021 tttgaggcct taatcgcgga gaagagttct ctaatcgaaa ctttaagttg gtaatcatga
10081 ggcttcaaga tcccatacac gtgttcctcg gctaaggact tctgcaaaac ctcttcggat
10141 tgcgtgaagt gatccttgta atgccaggat agggactctc tcttttctga atctctcctc
10201 ttctccggac tcttagctct taggaagaag attctagaag aaaagtcttg tatgcgcgac
10261 atgatacgcc gcattgagcg aaggacttcc cgataggaga agtctgagcg ggatcttcat
10321 gaatgataga attcataggt actattcccg gtggctgaaa tgaaatctat aaggaagaga
10381 agcaaatgta gagcgcagcg tgtctcgtgt gttacctcct cctaaagtcg gtcgataata
10441 gatatcattg agctcctcaa ttgtctcact aagttaattt catcttgaat agtcaggctt
10501 gtttctctgc tgccaaacta atactaaggc agccctccct ctttttctct atctatgtta
10561 aatgtcagaa gcttgactca tctcagcaag tttatttata catttgattg ataggatcga

10621 tgcccgcttg gttctttaaa tcgattgtta gttaaggtcc gggctctatg cttggccact
10681 atgttgagga aatattggtt ctaccaaatg gtagtttgaa cgccccctcgt gttgttggct
10741 gtctcggtat tcgttcttga atcattgact gagagtaagg gctagtgatc aagaaaataa
10801 gactagtggg gcatcttcct tttcgtagta agcaactctc tttagaaagc tctttcttga
10861 tcgaagtaaa gaggaactaa ggagtaacgc tttttcgttc attccaagga agctccttaa
10921 agaggattgc acttgggatc ttagcagagg agctccgaga cttagaatgt cctagataac
10981 acagttaaca gtggttttcc ctttcatcta aagaagatcg agttgtctgg actaatgatc
11041 ctaaaggtct tttttcggtt caatccgcat aggaactttc ttaggaaagc tagccttaac
11101 caaaaaagaa aggaagaaac cctcttccta tagtaaggtc gagtaagttg taactcccctt
11161 tcaagatacc cgctagctcc atacctagct cgttcactcc ttacctacta gccttgctac
11221 taagaatatt ccataagatt gcacacgttt acctcagatg tagatcgaac ggcataaacc
11281 gaagaaagca aagaaaatga ctctagataa gaatattcaa cctcaaccga ataaggagca
11341 ggtttagcgg tatttgcggc tgaaagaaag gtttcaaaaa aagtctatag gagcctaggg
11401 tccccttata gaggagaagc agcggtctgt cctaccataa aatctattgg ttgatgtggt
11461 ggggggatac attggatacc ctgcagctgg tgtggctggt atataagaaa gaaatatata
11521 ccaaaaagat tccagtcaag tagaggatag gtgatttaaa gaaatcaacc taacctattt
11581 gatgcctttt accaaaagca tgcttgcgcg tcgaataatt taccgcgtaa gccgctgcat
11641 ttttgggagt tgcttgtgtg taaaatgtat cccagtgtcg acgaccggtg gcaagaaccc
11701 ttatctcatt cttctgaggc caaatctcaa ctcggtaagc aagaacgcct attctgaggg
11761 tcttctccat gcttatcctg atggggtcaa tctcaactcg ctaagcaaga acgcctattc
11821 tgagggtctc catgctggta ggcaagaacg cctattctga gggtcttctc catgcttatc
11881 ctgaggggtg cgctatagga aatgatgttg ataaggtatg atatgccatt ccaaatcaca
11941 gcttcgatcc taggttgttt gctgtgtgaa tatgatacaa aaatgatcca gtgttctctt
12001 cctttcttta atttctttct tattcgttgt attcaacgaa gtactgcagg gctcttcacc
12061 tgatataatg aaacatatcc ctatgttcat aggtgcggtg tccgcttttt actatctttt
12121 taagtcaaga agcctctttt cgaggctcat gacgctttat tcctttctac taagttcctg
12181 ttatcttttc atggttaatc atgtacatgt tctagaaata gccattttag tggtggtttg
12241 tgtgtgtagt tgctttctca tatcttatta ttttctatct gagaaaatct ctatgaataa
12301 tcagttagat ttctgttttt tgcttctgtt gacaagtcgc tttttcatgt caccagaaat
12361 ggttacatgg gagattgcct tagtgctaga actcattttg ctctttgtat tcaattattt
12421 ggaagatccg gaatactacc aacataggaa gggggtcttt ttctctttaa cagtcttatt
12481 ccttctgtct tctttctatc tatttacata tatagactcg catattcata tcgttgtggt
12541 tgccgtttta gcggcagcag gggcgggagg tgtccagttc atgtgtttca gtaagactga
12601 acggggacaa aacgttgcat tactattcta tataatcaat aatattttgt tgggtttcat
12661 ttttagaaaa gaaggcgaga tcttccccaa ggtcacaatc tgctttctcg ttgccattct
12721 ttgcttgttc atctccttat ggctcattag tcaaataaag aaggattcaa tctattccat
12781 tctttctgaa tccaagaaca cgcttcgtgg gattaccttc ccaggattgt ttttgctaaa
12841 tagcttcttt ttaaatgatg cttctgttga tctaaacctt tggatcacta gcctcatgat
12901 tcaacttctc ggttttcagc tcctttattt cattctgaaa aaggaagacg aagatccaga
12961 tagagaaggg agataaaaga aatcattgtt caaagcgtga gaaaaagagt gacgatgtta
13021 gaaggtggag gctgacatgg tatatgcgtg agttctaggg aatttgttcg gtgtctcggg
13081 agttgttccc atttttggct tttttgtcgg gcacaaagga aaaggctctc catgggcagc
13141 gtctgcttcg gctcaaagcg aatagactgg cagtcttgaa gcgaaggaga attctttaac
13201 tgttcggtgt cggcatcttt tcctacttct aactgttctc ctaaccgggc aaggtaatcg

13261 gctaaccggg aattcatctg cagcccagac ctgctactcc tgtaagcaag acagtttgtc
13321 caccctattc ttcctaatac cccctttcta ctactattat agggatcaca ggccctagct
13381 agattcctaa aagctcccct tttgcatag gaaagaattg ggcattgcct tttcgatagc
13441 acaagtacag gtctggtgcg cataactgat acctgccatc catactcgga gaaagggacc
13501 ggaagagcct atttattctt attcaatgtc cgtgggtatc ttctactccc cgaaagaagt
13561 taggacagcc aaatcccctc tttaaggcac ctaggattag tactctcggc cattaccggt
13621 ggatgcggca tatctaggac ttttttacaa aagaaaagaa ggatatctaa accttttcga
13681 tgccatctct tccgaaagag gttattatgg gcatcttcga tgagaagtcg aaagcgctaa
13741 gatcggaaat gctgttgaaa gatgaatcct cctcgtgtgc gtggctagct gaacgcttcg
13801 caccttcctc tagagtagca gattctctaa gacatttggc atgagacaga cgcttctaag
13861 ttctcattag tatgatcccg ggaatcctct tgtttaggtt tgttgttcag aatcctataa
13921 aggagagcac ttcttttatc taagtctttc cgccttctta aggctttcct gagctattcc
13981 ccttgttcta aagatgggac ccagttgatg ttctcactgc atcgatagca agttgcttcg
14041 gcacccctat aatagaatgc ggtgcagttt caaggaagat gcactttccc ttaacaacaa
14101 tgagaagaag atgcaaatga agaagagcct atttaaccaa gcattcttca gccactaagg
14161 gcattccctt tcgatacaag caacgaaaga agagaagagt cggcattagc ccatctgttg
14221 gaggaaggac tacaggtagt ggtgccgtaa gggattcctc actcagaaga acaagaggaa
14281 tagaaggctt tcaagaagat atttccccgg gtccggtccc atgccacgtg tggatctata
14341 aggtccctag aaggtccacc tttccgaaca tcctatgtaa aagaaggtct ggatcgacat
14401 ctcgtcccct cagctcctaa taaaagaaga agcgactaag gcatcagtcc tgtcaatgat
14461 agaaggaaat ctcctaacaa ctataacaag agctactact agctcataag ccctatatta
14521 ataagggaat gctcctttga tctataggtc cttttccttg ctttaaacaa tactttcccc
14581 gggtcagatc ctatataaaa caagaggcat cccctcatcc ttttgacgag aaaggctaga
14641 tctgcagctt tcaaaacatc gataaggtaa gtctccctct tttacttagt gttagccctg
14701 gtttcggctg ctggctatgc ctgttgagag gggattggac tacccgaact gctgttggtg
14761 ggaaagacag atgccaaagc agcttccttc cgtctatata gtagcaggat ttttctaact
14821 agactcgatg gtagcggctt gcttgactaa gctattccgc tttccgccta aatggaggtg
14881 gtatttaacc catcctaata ctttctctgt acaagctctc tttcctgatc gaagaccacg
14941 aaagaccctt taacaagcta acgatcctgg ctcagaaggt gctaggcttg ttatgaacca
15001 agctacttgt ttacttactt gctcattaga ggggggaggc taaagcaaat ctttagttcc
15061 ctattactaa ttagtagcat ggaagcaaat cctgtagtta gctcgaaacc gggaaagcag
15121 gaaaggctgc taggggaaag agaactacaa taagtctact cagtcccaat actccttatc
15181 gataagtaag gcggggttgc tgtttagttc cagtaatcta gctaggctgt ttgttggcta
15241 ttcatagatc aggagtcttc gtcctctgga ttagtctgtc agtccagtcc gtcaggtcaa
15301 tcaattgcca ttgcaatgta agagtcaaaa tgggattatc ctctctctca tactgtcgta
15361 agtacgctcg ttcctgtcct tgcgactggt tcttccccag tctggccaag gcaagcgacc
15421 tcaactgcac tacctagcca accatcttca ccccatttat cccccgtagg gcagagcgtt
15481 cctcaatggc aaactaggaa cacggaagcg atgagagcat gcccactctc gccggctaac
15541 acaatggatc aatgcagggg agactagccg agaagcctaa gctagctatc agctgccgta
15601 ccccagcgga gtctagtctt cttatctaga tgagcatggg tgttcaagcc cccagaactg
15661 agagactcaa ttcaatgtat tcgctcaagg gagataaatg tttcgactca ctaagctttg
15721 caagcatgcg tctaattggc tttccggttg agtatcctaa tgtgctaatt cagtaattaa
15781 gaaatgcgtt aacaatgcct ttcttaatct ttctagatcc cagaggtgtg ctggttcgaa
15841 tccagctcgt gacaaagggt agccctttct ctttaacctt gaagcgaaga gtttcacccg

15901 gtctacgatc aggacaaagc gattgatcca gttgagagcg tagcgtagga gacagggaag
15961 atgaccgtca acgggcaaaa gcctctaatt cttattatga aacttttatg cttcaatcag
16021 gcctatattc gcatttgaat gaggccctta accttaggta cctccggaac aacttgaagg
16081 cagtttattg accatagcaa ctaaatcttc tttgggaggg caaagcacta gggagaagct
16141 tcctttggga ttgttttcct ttttttgcac ttatacgcga gtctctgtct ttgatgtcaa
16201 gcccagtccc agtgacccgg atcactccct taggcggact aaatatgggt tgtaacttgt
16261 aaagtccttg aatgaatgct ttggaattgt ataagagcag gctgtgatgc gaggactctc
16321 agggacctac ttaagtctgc gatcactcta aaagcggata ggaccaaacc ttttattccc
16381 ctagcagcgg ttatgtctca aaccaccggc aacaggttga gctcctacga tcacaccttc
16441 tcttgcaaac atagcactgg atggaaggta tgcttcaccg acctcgttta acaccatgct
16501 tcctccgaag ctttcatctg agtagtcact acgccctagc ctatcgctga gtctttggaa
16561 gcggtttcac tcctttatag gtcggaactc tggaacctaa agactttatc aatcagacag
16621 gatagatgga ttgagtgcgc gttgccttga tttgaggtga actccttttc ctcttcttcc
16681 ggaccggacc cttccatgtg agaagctgga agtcgagtta ttgatgaatg agaatctaat
16741 gtcttattaa acctttagga gcgatctttt catccaactc gaaatatcgt aagagaagaa
16801 aaagaatctt acgcccaaca actcccatgt ctttcttggt cggaccaacc caaccataaa
16861 tttcgttcca agtcttttg catttttaga gcaagaagcg gaactacaag aatactctca
16921 ataatgccta gatttctacg catctttcac tttgatgaaa acagtcttaa tagtaatcaa
16981 tcgtctacta ctcaatctta tactactctt acaagtcaat ctcaatcagc gacgactatt
17041 aatgattatg cttttcactc atctggcact tctggcactc agggttcttc taatggtatt
17101 ttcgaagatc accctggtct taacccttca agtgaacgta tagtagagct gcaatctgat
17161 atacacgcta aattagagga gttgatgatt aacagaagtc ccgaagaggt tttggttgcg
17221 gccgaagctt tacatgggga aagcgacaat atccctttta tggagttcct tttgaatgat
17281 ttgaacataa atggtataca aggtgaagcc tatactgatg ctctgaatct agcggggctg
17341 gtccccatcc ccagcccact tgagcaattt gccattctcc cattgattcc tatgaatata
17401 ggacacttat atttctcatt cacaaatccc tctttgttta tgctactcac tctcagtttg
17461 gtcctacttt tggttcattt tgttactaaa aacggaggag gaaactcagt accaaatgct
17521 tggcaatcct tggtagagct tatttatgat ttcgtgccga acccggtaaa cgaacaaata
17581 ggtggtcttt ccggaaatgt taaacaaaag ttttcccctc gcatctcggt cacttttact
17641 ttttcgttct ttcgtaatcc acagggtatg ataccttata gcttcacagt tacaagtcat
17701 tttctcatta ctttgggcct atcattttcc atttttattg gcattactat agtgggattt
17761 caaagaaatg ggctacattt tttaagcttt tcattacccg caggagtccc actgccgtta
17821 gcaccctttt tagtactcct tgagctaatc cctcattgtt ttcgcgcatt aagctcagga
17881 atacgtttat ttgctaatat gatggccggt catagttcag taaagatttt aagtgggttc
17941 gcttggacta tgctatgtat gaatgatctt ttctatttca taggagatcc gggtccttta
18001 tttatagttc ttgcattaac cggtccggaa ttaggtgtag ctatatcaca agctcatgtt
18061 tctacgatct caatctgtat ttacctgaat gatgctataa atctccatca aagtcaagtg
18121 gttatttatt tataattgaa caaaagcgag gagcgacggc cgcaatagcc attattttta
18181 ctcttttata aagaaaagag accgcccttg atctgctacc tccccacttt ccatctgatt
18241 tggaaacggt ccttcgtttg gtacttgtgg actagtaact gacacaggag catctccatc
18301 tcgatcaaag tttcatgtct cgctcgatag caacagccaa acccttctct ttgtcagctc
18361 gatctgctct ttttcttgta gaggaatgtg gagcaaggac tctcacaaag atagaacgga
18421 atgcctattg cttggtctct tccttgccac tctcatttca attcgctact tccatgatca
18481 ctgtggcagg gtaagtttcc ttgctcggta tcaggtatag cttgctttgc tcactcactg

18541 gggctggggg gcgggataag aatggtattg tcatacccag tttcatgtgg caaggtacgg
18601 tgacgaatga aaagaggttt ctcctactgt tttagctgtt attgtggcta ttatagcaag
18661 tagctaggag ttattgtagc taatcaacaa gcaggtgtag ctttacttcg taaccttgct
18721 ttccctcctg cttccgagag gaaagtgtag ctaatcaggc aaatctagct attagactct
18781 tactacgggt catctaggtc tttgatccgc aacaaaggct ctttctggtc atcacacatt
18841 gaaggtgtct cttctctttc aaatggagtt ttattacaca tcctattatc ttcctgtgtc
18901 gccatcttct cgtattcttg cccagaaggt tcttctttct ctggtttcaa aaccccaacc
18961 ggtaaccaaa acaagcaagc cagcttcggt agtagcttca aaggaattgg tagtatgtct
19021 tgaataggaa agcattcata atgtatgaac tcaacctatg ttcaagagct tgaacaatga
19081 agtgaaaggc tttactttag agttcgatct ggttttcatc attgtcgaca gaaagaaggg
19141 agttttttaa tttcctttgt ttgttgaccg aatcatccca tctttctcag ttacatgccg
19201 atattttact tttgttttgg cttttaatta gcctaaaagt tcaagagtag gcccgaaccg
19261 ctcccccttt ccatttaatc actgacaaaa cctacctttc agttcgactt aacgaaagga
19321 cttcaacttt agaacaatca atcgaacggc aggtaagggc ttgtactctc tcttctgtgg
19381 atacgctacc ccttccggct atggtagggg ggaaaggaag tgagatctac cccttgattt
19441 gatattagat gagcggccgt actatatgat cgttcgggag acccggccca ttcaaataaa
19501 aatagaacga gcacctacga ctaaggggaa tggaatgtcg accacagggt gagagaacct
19561 tctaatacga gagcgagtga ctggtaaagt cattaaactt agtcattttg atcaatatgg
19621 ttgcaagtgg actgggttta tgtgtttaaa ctgactacga ccaaagtcgt ggctacttca
19681 accaaaaaaa gggcaacccc ctattcaaac cgaaataagt acctaactta cgaagaagta
19741 gttggagctg ggctccgaac tatgagagtt tgcttttttt ttctgtttct aagagcggtc
19801 tgatccctta tttgatcaga ccgctcctgg tgttagtcat taatggtcgg cttcattagt
19861 accctttcgg tatgtgttgc gaacactttc atttttagct tctcatcttc tctagagaag
19921 caaaactcga acggatagaa cagatggtcc aactacagaa ctttttcttt ttcattactt
19981 ccatggtcgt gcctcgtggc acggcagcac ccgtactatt gaaatggttc gtcagtagag
20041 atgttcccac aggtgcccct tcttccaatg gtactataat tcctattcct atcccttcat
20101 tccctctttt ggtctatcta cattccagga aattcatacg ctccacggac ggagcaaaaa
20161 gtggagtctt ggtcagagca agccgcccta ttctattacc atacataatt gggagaagct
20221 catccgaaac tagagctaga aacgccttat ttcgtttcgt tcccgttctt catttccttc
20281 ttctcgaatc caagggggac ttctcatatt tagaatcttt ctgcggtgtg ctccgtttac
20341 tattttttcg tactttcttc tctttaccac gcgataggtc agcgaagcgt gagcgggcgc
20401 ggagaaggaa acgccaaaca cttcggccta acgggaatca gcaacgacga aatgacaaga
20461 tgaggtgccc cgggcacccc catttagaaa gaagggtcga aggtttcggg cctgtagctt
20521 tccccggccc cccttcgtcg ggcggtgctt gtgtgggggg tgcgcaacca gaaatcgggc
20581 ttgaagctct caccttacca acgagccgac agctgatggc tgttggtcac gactactaca
20641 aaaaagctcc aatgaagatg aatatttcac atggaggagt gtgcatctgt atgttgggtg
20701 ttcttctgtc gtgcgacccg gcggcgtatg tgcgacctgt ggcccacgcc tcctatttgt
20761 tcagggcggg cggcgtgaac tctgattcga tccgggtatt aaatcccgcc gctgagatgc
20821 tcagttgact ccttaacctt gataggaaga tggcttattc aagaattcgt gcataagggt
20881 aaggaacttt ggatgaacta atgcgaatgg gtgtaagcct cgctgctcgg aaacacccag
20941 tgctgaccac actgagagac acgaaagcgc aggtaacgcc agttggcgaa gtggcgttaa
21001 gcatccctag cggtacgaaa agagaggtcg tgatgatatc atctacgtcc gtaccgctcc
21061 tcgtggagta gatcccgcat ccaaccaagt ctttgaccag ggaacgggag aaaccccact
21121 accgctggca ggccagccgg gccgtgagcg cggtgggaac gggcttccca aaaagccagc

21181 ccgggccggg gtcagcatag aatgaaaggg acggccctaa tgttgtgttg gcaaagccaa
21241 cttcctgggt tgcgggcgga gaaaggcgga cgtggggact cgggtcgggg cgcagcgtaa
21301 ctaagagggc cattccattt agggcgagac aaaatgggcg gtctggtgtc cgccccgact
21361 ggcagctgct gggtctcccc atctctccta aacttccccc ggtcttcggc ccgagctgta
21421 tgaggcagaa actcgtccca cgtacggttc ggaggccgag ccccacccca gcagtaaggg
21481 tgcggcttag gtcaactaac acaaagaaga tacagttcac tcaacgattg cctttgggtt
21541 ccgaactcca tatgggaaag gagcgttgtt gtttgcgagg tctcgatcat ttacatggac
21601 ccacttctca ttccatttgt gggaatttga tgatctataa accgtcccta acgaacgatc
21661 ggctcaggtt tgagcatgat gaatcacttc gtgccgacct gttgccaata aactttccgg
21721 cctcatatga gaatggaaaa ctggagcatt ttctgcatcg gtggatgaag aatcgagaac
21781 ataataattt ctggttgacc atgttcccag aaaaaagata ctttcgagaa acgacgagca
21841 cgacggaagt ggctatacat acaaatctat ttacggatct atatgctccg attggaactg
21901 gaagttccag aacaggtggc tggtatacta ccataatgaa actgcctttt ctttttttta
21961 ttcggatcgg atttatgttg gcttcgttgg gaggctcgca tagtttgtta cgtcagctcc
22021 aaaaggataa gttgcgttgg aatcgagaaa gttccgtgga gttcataatt gcataaaagg
22081 agtcaaagta agtggccgcg gcgcgttgag gcacttcttc gacggtcctc gtacgcccgg
22141 cctgacggcc cgctctgaag aattcatggg acgagcgaca gacagtcttc tttttcttag
22201 tcttctctat ataaagggag tttcgattcc gcagaaccta taggagcaat agacggagtt
22261 agtgtggtat agctgttagt aaggcgtaga gcagtagcgg tcttcacttt tcctgttaca
22321 gaatcgacta taaacgctgt tcatggcacg gttagcggtg aaaaaaaaaa aagaatagcg
22381 attgttgaat cagtttgctc tttcgacttt gattgctctt gtgaagctct gaggtgaggt
22441 tttattcctt tactttagga gtaggagtga aggcaagatc cgactttaaa tcgagtgaat
22501 agcaggcttg tagttgaatc gagattggcg atgagtacaa gatcgaaaat ctttctcgca
22561 gctcgtgttt tcaagcgaac gagtggaact aaaggaatga ttgttgacta gaggatacaa
22621 acaggagact cccttttgc ttggcttgac catatagggc agccccactt caatagtctt
22681 tgtattccta tatagctatg ccataagaaa gaaagctcta actagaacga ataccccttc
22741 atatatataa gctatgccaa agtggaagtg actcttccga ggacagcccg atgggatacg
22801 gcattagatg tttttagctt tttccgttgt tctggtatta gcagtttctg ttctagcaga
22861 tgcactagag gtcttaaagc tagaagtctt acctaatgct tcttagggtc ttagcaacta
22921 gaacccgtgt ttgactccct taccttaata taagctatgc aagaagctct aagcgagtat
22981 accaaagtgg gatacgatct ataaccaatg tcttgttttg atgcctttaa gctagaagta
23041 taccgttgca agggtgtttt actaccttag aatgccatag taccttactg caagcaatct
23101 cgactaaacg agaagactat gagagtttag ttgcaacata gctaacaata gagagaaaga
23161 ggccgatatc tagcatctat ctttagctcc taattagatt gtgcttcaac aggcaataac
23221 cgttgttata aacccttctc ttctcattgg caatgaaatc gtagctgcac tccaccttat
23281 gctttctagc tatgagttcc ttacagtcga gttactccgt atcctagcta ggaagagaga
23341 atagctcaaa gccttaactc cctctcacag acgttcgagt tactccgtat cctatcgctc
23401 gtccccctgt ttacatgctt agtcgcatag actatggagc gaaagggctg cttgccggaa
23461 cagcagatcg agcagcctat tctttaaacc atttctgccc caactattcc aaaaagggta
23521 acaaatgggt agaattctga gacaaaagta atttaacatc cctgattcgg aaactgctgc
23581 agtgggaact ctacttgcca ttcctccttc cgaagtgaaa gttttcattc tccccttggc
23641 ccagttgaaa agcctaaccc caggtgaagt accagattct gaagtgaaag taaggaattg
23701 ccctgtctga ttcgtaaagc tttcggagaa ggaggggaga tccgcggagg tagaattctg
23761 aatacgggta cgaagggtca tccagaagcg ctggaagggc tggaggaagg ggacgaaccg

23821 catcgattga gttatatccg gcaggctaac acccacgtac ctagaaaggg aggggaaaga
23881 cgatagcaag tttatgaatg aattcttgag ctactatgct agtgactcta cctcgggtga
23941 gggagaggta tgtaatactc gaccaggaac gattagaaag aaaaagcgct tgagtagaaa
24001 tatgtgctat acctgaaagc aagtgagtat gccttgggta aggtatacga gcgtatgact
24061 cttccgaggt agttgactcc cgcgattatg caagctagaa aagagactct atctttctct
24121 ctttcttttg aatcgtagcc tactattcta ttagtgtatg cctaaaactg aacgaattct
24181 cctacatgaa cccatttctt tactttttcc atgttgctgt acgaaaacct aagaaaactt
24241 tccagtcaat cggaagttgt tctttacccg gagaagtctt ttatataagt tcgaattgat
24301 agcatttgcg ccattcaatt cattgaattt cccttccagc gtttccattt tgtattccca
24361 accatttttt ggttgtatta tagattcgcc aatgagtcgc tccctttgct cggtccaaaa
24421 aggatccgga tctaattccg acatacgttg aattcttcct gttttcacag caattctttg
24481 ctgtatgctt tctgggggga atacatcatc atattgccga aataagcgaa aaaggatttg
24541 ttgttcgggg gatggtccct cgttccacag gcgccgcatg gaggggagaa cgaatggctc
24601 aggaatcgac cggttccgag tagactcgtg gagctgcagt ttggattatc gtagaataag
24661 aggagctgta tgaggaaatg acttcactta aaagaaaaga cagatgccgc tgcgaggcga
24721 gggagccact tgtctggtct tgcggtgcac tcattcccgt tacgagaatg aaatgacgcc
24781 ccagcttgac tcgagaccaa gactccttac aactcgcacc aatagcggca ctgagcggtc
24841 ggagatttat tgaaaagaca gccccccccta gccgcctacc tactcgtgtt cgtagctcga
24901 tcggaggtca agactttggt ccggcggaaa aacagacgtc gtccgtatca agtgatacgt
24961 gaattgctca gtagtgcttc gccactaccg tagctggcgg aaatagctta atggtagagc
25021 atagccttgc caaggctgag gttgagggtt caagtccctc cttccgctcc tggcttcgtc
25081 gtttagtggt aacgagtgga gtgcataagc ccctttagag agaggggtga gcagcaagca
25141 gccccttgta ttgtataggg ttccaaacct atcttactaa taacatagaa aggggcaagc
25201 caaaacctac tcctaacaag ttgctggctt ttcatcagcc gttgcgcgct tctgtttttc
25261 agcaggcttc ttcaaatatc ccaaaaatga aatgaagtag gggctataac tataagtgta
25321 agtagctagc tagcgcgcta gcgttttccc ttactagtaa gggggctgct agttgtagcg
25381 cgcagtgtac tagtgaatag gaaaggcgaa tttagattac taatgacgga tggaggttga
25441 ggatagaaca tgttcggtgc ctcgcccttg tctccttcgc cggagacttc aaatgatggg
25501 aatcctatcg ataaagaaga ggcataagca tcgcctctcg atccaatccg tcttccctgg
25561 cctccccaac acactctgga tacgaaggaa acctcccgcc atagagaaga gatctaaagt
25621 ctgggtcccc tcgatcaccc tcccttgaac ctgaacaggt cgagagagat gaacccgcac
25681 cacattttat aaccttcgaa ccgaaacccc caactagaga tggaattcaa gaacctaaac
25741 cactcagttg agagctccgc gactggttca agggaaggtc caccaatgat tgataggccc
25801 cctctatccg tctcttgatc cctcattcca ctaatccgtt gttactgatt cattcaaggc
25861 atagactccc catttctttg tcttattagc gcaggtgttc gtcaacgatg aaagccgctg
25921 aacttaagac tcgagttgag aaaagaaaaa gggctaggcc cccgggaggg ctttcaggga
25981 ctggggaggg tggattagat tatagagcta ggggcaaatc gaagggtttt ccattgggat
26041 tgggcatgga cgagcctcga ctgggccagc attgatgaaa aaaaaaactt atcccatcgc
26101 atcattaacc ggtgtaggat taaagatcag gtccgggatt cgagtcaaat cgaccttccc
26161 tctcatctca gggtgaggca aggaattcaa tccattcttt tcttttatgc cagcccaaac
26221 aaaacgaatg actctctttg gaaaggaagg aatgcaggga acaactcttt cctttccact
26281 ggttcttgaa agctatcaat ccccccctct cgcatcggtt ctgcatcaga tgatgagccc
26341 atgcgcaaac tttgtctttt attggcgccc ggctagtaga ttgagtcgaa agcctaccaa
26401 cgcataaaga aaggttccga ttcgagcgag agcccaaacg ggggaggcga gaacatcttg

26461 atcgaaagcc ccactgttct gaatagtgag aaagactttt caaaattcga aaaaatagat
26521 cgagaatatc atcatctgtt aggtgggcca accgaccgag ttgggatggg cgtccatgtc
26581 acagaacctt tctctagcca agaatcccat tattgaccga atcggggaat tcattggcaa
26641 atgaaaaaga taccgttttc attttacact cagaaaaaac ctagaaaaga ggaagagtca
26701 ctaagacaag agctaggtaa gcaagcaaga aggagaggct agaaaaggcg aggcaagggg
26761 ctctctatct ctaggaagat tgtgtccagg atctggtaat ctaagccttg cttcttctgg
26821 tcggctcgta ttagcctgtg ctttattaca ggtagtcatt cccccgttcc tttagtcttt
26881 tcaacggtac ttgaatctta agtcgcggtc atgtctcgcc cccccagtat agtgaccggt
26941 tccatctttt tcccgaattt catcagttcc aggctcaagt gcctgttcat ccatcttcat
27001 tccaaaggcg aacatctcca ttgagtgact gtaactgcta tggtttacag tcaatagttc
27061 ttaaccaacc ctttattatt atgtctttct gaagggcttg cggttcatta caccaaaggc
27121 tttcagtgaa ctattgaagc tatcgagaga gtaccaaatg ctgacggtga cgaaggttac
27181 cgactttcgg tggacgcgga gccaacgagt tcagtcgaac agcgtaacga gtctaaggtt
27241 acagaagcgt tcgccaactt taataggcat agcattgcaa gtaaatagag cagagagctt
27301 accctttgtt tctattagag tcgcgagctt gttgtagtcg gtcctaaagg gagaaccttg
27361 ctgcttactt gctatatccc cgcgtccttg cacggctcgg cttcttcgag ccctgcttct
27421 cccttccccc tctccccgtt ctaccgtcca aaacaggccg tatggagtat agccaagtgg
27481 taaggcatcg gtttttggta ccggcatgca aaggttcgaa tccttttact ccagattatg
27541 aacacccgat cggatctgtc aagaacgagc tgacgactac aggggaagcg gctgactgca
27601 gtccctgagc ccagcttgta gcaagccaaa agtttgactt cagcctactt ttttttcgc
27661 tcgcccagcc actgccgtct cgtacttttg ggagcgtcaa gtagatatgt gagctctaag
27721 tcagtagcag ttgcacgaac gaccagtaga cggaaaatgt aggaaccaga gtttgatcac
27781 tttccaagca tggtattaac acatgcaaga actgcactcg catctgcttc ccctattata
27841 taaccgggga cctggaaggc taccggactc aatcaacctg cgctgcgaac ataaggagtt
27901 ttcctaaaag tagccgtccc ttaccttctc ggacgatgac ctaagggagc tcttgccggg
27961 agaacgttct gctcctctac acgtaacggt gattctattc taaagggcta cagcattcct
28021 ctctccctga tcgcgatact ggggttgaat gtttgcccat gttccaggct ccgatatctg
28081 gacaccccag agtaaagtct caccccatcg gacatgacat tgctgcgtat gacagttaaa
28141 aaaggtcttc catcgggctt acgcttacac agcgtaaggc ttgacagcgc ctcattcagt
28201 tggacatcaa gttcgggcct acagtagtcc caatctaatt ctatgtgctg gacattgaag
28261 catccttcaa ccggcaaaaa agagttataa gtcatcaagg cgtccgcaac agatctatca
28321 taaataaggt tctttcatat cccgtccctt gagggattga ggcttcaaag gcatcgcaca
28381 agcccacttc aagcgcacac gccctagacg cgtacaagcc cgcgtacgca cctacggcga
28441 ggctttcact caagccattc atccaaatca gtagacaccg cattcaagat caagagattc
28501 cacgcggaag gatgcgtcag aaagcgctat ctatccacaa cattatcatc ggagtcgggt
28561 tgaatcgggg cgctatgata aggtaggctc atacacctca acagcgcgat gtccatcctc
28621 ttttgggtga ccggatagct gagttcacct tggcttgctt attcagtccg tgatacgagt
28681 agatgaatcc acttcatccg gaaatggtga aagctcggtc aatgaaagtt cgcaaatctt
28741 cccacttcaa aggtatacta gactctttgc ctttttgctt cacagcaaga aaagtcatta
28801 agtagctctc aaacagagcg ccatgcgaat aaaccaaaat gtaaagcagc catcattaaa
28861 ttaaggcagc aagcaaacct ggaaaaaagg tcagccgcag atctctcttg ccaattagat
28921 catcaatagg catcccgcgc actctatacg acctactctt atcgcatcta tagagaagga
28981 aggccacgaa cctcttcatc atcaccttca ggcgaccgga gggagggctg gcatgggcca
29041 atcgagcatg ccagaggtct gctgtctcct ggcgatgggt cttgtcgaca tatgttgtac

29101 cagcggacaa cacacacgta ctacatttga actctgattc cttccatgag taccaccaac
29161 atctgccttc gctaacactt tgggttgaga ccgaagagaa gataatgaat gcccggcccc
29221 tctcacgtaa gcatttgtga aaccgaaacg aaattcttct tcattcctgg aacatgataa
29281 acatattcta acaatctcga attccctttc attggtgaga tcgcgaggtc acccacgtgc
29341 tccacggcac gggatagacg gagttatacg cagtcacaac cgtaccttca tacttccgta
29401 aataaactga ctcatataac cggtcaggtt atgcgagcat ccttcagaga ctatccaatc
29461 cgttttttcc tgattatcgt cacttaaatc ccactagctg aaatagaatc cgtcaagtaa
29521 gagataagcc agagatccag atcattagaa tacgttaccg gtccaagatg ggcctcatcg
29581 cgaaaaagag tatgttattc ctatcactag gagtagtagc gctagcgacg cagaaggaga
29641 aagtagctaa gcgctaagaa gctatctaga gagtttccag ctgagggaaa ttggcttcga
29701 ttgttcttag tttcgactcg tagtcttcga ccacttttat tcttctcccc gagggttgct
29761 tgcttcattc attcgtgaca ttactggtta ttcttactaa gtttcgcctc tagtgactct
29821 tggaatcatg cgcatggctc catgcatatt catgggaggc ctaacgagtg ctacgagtga
29881 atgcgtagct ttgtcttctc ttattcaatt tcgagttaca ggttagaggg aaagagaact
29941 ccccaaagca gccattctct taagaagagc tattttacct tgttatttca aaaatgtgta
30001 tcaccatact gaatctagcg caaagcaagg aatgattctc ggaagttggc tacttactta
30061 ggtaaataag ttccttctct aagacctaac tgcgctgtcg agggtgatct tgaaagagcg
30121 catgaaggat agggagggac cggaattccc ctttttgaaa gagctgcttg tactcaagct
30181 tgaaggtgga gatcttataa aggtttccag taagcaagaa gcaagcaggt atctattagc
30241 agcaatagtc agagtcatat aaaaggtagc caaaaagaag caagaaaaga agatagtgat
30301 ttggctaacc tcaggtcagg tccactcctg ggtcttctct tgcctatcgt ccacttcggt
30361 gactccaacc ccggaaacat gatctcttcc actaacctct tcacatgaac ttccggagtc
30421 cctatctaat tcctaagaaa gctaaactaa accgattcaa atctctttcc cattcagaaa
30481 gaggactctc atacaagacg cattgccctt ttttgatctc tcccatgtgt catttgtgaa
30541 agaattcaat ttcctttgta gttcactaag agggagttcg caggaaaggt aaaaaaacct
30601 attctattaa taaatggggc ggctacaaag agctagcttt gaaaaagaac cgacttttaa
30661 ccttgaaagc cactagagct tcttccccgt gcatgcatgg taacaatgat taacacttgc
30721 aattcttcct ttctaatcga tcgagcctcc ctatggtagc agtctttagc aaccgtccat
30781 gcaagagtag cacttgcgcc tatagcaaag gttcagttaa aagaccatct gcgaataagg
30841 cagtgatgcc tcttgacaag gataaagctt tgttagtact aaagcaaaga tgatcacatg
30901 cccttcgcaa tgcttcgtgg atcttatgaa gagcattcgc tggtatcaaa accaagtcaa
30961 aagcggacta cggcttgccc gcgagaaccc tactgagctt cattaagctg ttggaaaaaa
31021 ggaattcaca gtcaaagaca gaccgaagcg agtaaactac ctcctaggat tgagatagat
31081 acgaggctgg ccctgaactc tagcgaacca ctacaggacc cgaattggac tgaacccccg
31141 cctaggcact cctacaacgg atagagcctt gacttctttt ttcttcgggt gatgccttaa
31201 actaagaatc ccgtactagc ttttctaagt agagctgcgc tctccctata acgccgcaaa
31261 tcccaataga acgaagaact taccttatta ttacccaaac gaggagatag actatcagct
31321 taccacttac ttagatacgg ctgaagcaga gccggggaac gagactccct tcgtagaaag
31381 tgactggttc cgtgtattaa gagaaggatg ttccaactga atgaagattc tggcctttca
31441 tcttgaatat ctcttttgac tagactttca tctttctttc actggcgaag acatatcacg
31501 agcgagggaa ccacgtctgc atcagtcgaa ggggtggaac gccctactga ggatggttgg
31561 gttgggaaga gaatagcata cgaagatcaa gtagaggtag tctatggatt gttcgcatgt
31621 gtcctaaaag gtgccccata tagttgttat tgaaggaatt aaagcaggta taaccttgat
31681 cagtcccgac ccaaaaatga atagaatggg aagcgaggcg agggtctttc attaaagggg

31741 ggaaaagaag ggtggggctt tgttctgggg ggccgccttg cgcagctttg gcaaggagat
31801 aatttcataa agtcactctc tccaaccttt tatatatccg gctttagaaa gtcaatatga
31861 gatttgcgtt ggtttttcca acgaaactaa gcactttttt tctttctcat tcggaagagc
31921 tctttttgtg gtctcacttt accaccatct gaaatgcgcg aaacttcgat tggtggaagc
31981 cagtctatga agattctccc ttttcttacg tgcaattccc ctctttcggt aagcatccag
32041 tatctcatca aatgaacatt gctctaagct tatcctgtag cttatacgtc gtttgaaagc
32101 tgcttcaagg atccaacgaa tagctaaggt ttgttgacga tccctggcta caatcccagg
32161 gacatcataa atagtacctg ctcttcctac tctttccact tcgcatatgg gctttatatt
32221 ctctagggcg tcaaccataa gtttgattac atcgcgttca gttcgagctg ggcgatgaaa
32281 agtttgataa acaatagcac gaactcttgt ttttttacct tctttcatgc gaaagttgac
32341 caacttcttg atcaattgtt tttgctcacc atccaagccc cccatatagc ttagaatttc
32401 cgagcaattg gaagccgctt tcgatgacga ggccgaagaa tttagattac gcaatcgtta
32461 ctgtccatct ttctcagcca actccccagt ttccaacagt gactgtctct gatcccccta
32521 tttattctga gcaatagagt gagtgatctg ctaagtcaac caacctttttg tgtaagtaat
32581 aaagcctttc gctcattggt cgtcttcgct tcaagtactt tcttcaatta gttcaactca
32641 gctgctcgga gataagcaac aaacaacggc tcctcccgct cactcgactc tagtgtactc
32701 tcgagcttta agaaaggatc ttagccaaac gttgaccggc ttcgcgagac catttcggtc
32761 taaacaaggc taagcacttt ggtcgttggt ttttcgatca actctctgac ctcgaagatc
32821 tcacgagcag gactgacgaa agtctctcaa gtgattaggc ggggacagga ttcgaacctg
32881 cagtcttcag gtcatgagcc tgatgagttg accattcctc taccccgctt cttatccgac
32941 tgaacaccaa cccaatctcg ataaagaaaa gtaaaagcaa ctacatcatg aaagaaggtt
33001 catataactt atctacgcaa ccgtagaatc tactctaccc tacgctactt cttatcttat
33061 gatatttcga gttttcgatt cgccgccatc catattgttg aacagaaacc tatcgtctct
33121 cccgtttcag aaggaggtcg gtggtacaag ctgtaaatag agaggaaagc cgggactaaa
33181 ggacaggact catactagaa ctccttctac tctcttacag tttagctaaa gaagtagata
33241 ttagtatagc agctaggact cttttactat tgtatgcacg gtcaagctac tttcagtaaa
33301 tttccggaaa tagtaactgc cgcaaaaagg cattctgttg aaagttttca gtaatcgtct
33361 gctcatagaa gtcggattaa atcgggcttg atgccatctt cttcagttag tcttagggca
33421 aggagttctt ccgtccctcc cacagcggta atgccgatag ctcttattac cggaattgct
33481 ttagggattg gattcttctt ctttatcttc gatgcgaggc atattcatat tctcgatatg
33541 ccactagctc aggaaagaca gacacgcaca cctcctttca ttgccattat ctgcctttct
33601 atcttggcta ccatgcccgg gagagactgg gagtgctttc gagaaaacat gcaccgggaa
33661 ttccttctaa gatcttattc tttgtcctga gaacagcctc cttctctatc tatttcccct
33721 catttgcacg agatgacggg attacttgca tactcgtaag gcatgatttt tccacttgct
33781 tgaagctctc ttcccgtgtc gatgaggggc tattcctact acgcgataac tatcagagca
33841 ggaaagaaag agttggttca agggctctct attacctaat agatgcaagc taggaaagag
33901 agtcaatcca ggaagtcagt ctagaagcta gcagagtgaa gtaggtcatc actatcagta
33961 gtgacgcaaa aagtcgcaac gcccctctgc taggagaatc agctgaaaca agagacgcat
34021 tggcatccaa agaagcaagg gtgagctaaa accctgcatt caataatggc ataaaaagaa
34081 gactataagg gctttcgggc ttagcacccg aacaaagaca cgacccgtat tcttttctgt
34141 ttccgcttag tcttttgttt tttcggaatc agaggagcag ccaagggggc acctatttgc
34201 atctgaaaag cggtacagtc tgaccccccct tgctttcggg ttaggaaaag actctcacct
34261 caccatccaa ctttcaatcc gaaaaaaagt cttagaatct gctgctagtc aaggaagaga
34321 agggcttctg tagcctttgg agagagaaga ggtggaggac aagataaaga gagttttggt

34381 agcatccgat ttgtgtaact actcgtatca gctcaaccag taattggtgc ctttcggaac
34441 tctattacct tccctcctca gcccgaggac aatctctcaa atacacatta agatgttgat
34501 ccgttttatt ttctttgctg attcctctca atgaaattgg ccatgttgca ctaagttact
34561 tacggatgta tgcatgcggt ccgggaacac tttggggtga acacccatcc gaacaagtag
34621 ggtcaatagt tcagcattta ggccgtaaca tttagcaaca aaaaaagatt tcacccaaga
34681 agtgctctcc gaaccaagct agatagtctc ctatcactag gctcaccaac caacctggac
34741 tttcattctt attattccta ccggatatag gatataaaaa ccataaggct tgtttccagc
34801 catgagttcc cctatgacat aagcgctctt aggcgggctg ggccattcca taaaatcttt
34861 gagaaggggc ccaatctcgt atttgatggt cggcgtggcg ataggcttcg cttctacgac
34921 tgccttcccg gccgttgcaa acactgagcg agaacggtaa ggggcgcaca aacaatgtcg
34981 ttgcgcgggg atgcgattcg ccaagctggg gcaaacaaag ccgtccggcc ctaccggatt
35041 aggaaagcga aggcctttcc ttcgaaagga gaccggggaa agaaagagct atgctattga
35101 ccgacccttt tttttcattt tgtttgaagc gggtcaaata gaaaatgaaa tgcagaaata
35161 ggggaagggt tccaaacctt ttttgggttt aagggctgct cgctctaccg aagtaccaaa
35221 tgctttcgag gcttacacct ccctattaca cgacctaaaa aaaggctttc agtagcgccc
35281 ttagaatcta agccttttga aggaccagta gttgtagctg tgggtagggc tttcgttctt
35341 atcgctccgg gttccaatct atatgacctc cgggcggcac aaagtacacc tcttccgtat
35401 aagtaggtag taacctttaa gagtctgttc aaggggggag ccttcttatc tatcaatgga
35461 aagatctcca tgtgtggcgt gataaggaat cctagaaaag atcgatggag actccctcca
35521 cggtcccacg aagatctcac ttgtccagtc ctggacaagt gagatcttcc ggtctgcgtg
35581 cgcgggagca gctcaaacaa agaagagtct ggtccggtct gaattcaggc acgccccccg
35641 cccgtctgct gctaggtccg ggaatggcgc caggcgaggg cgccgctatg ccccgctgct
35701 ctgctgcggc cggcccccgg actatgcaaa agaatcgagg ccggggggtct cgtccatagt
35761 ggtccccccc ccgcctttgg tgattgaccg aacaaacaaa taggcctaga tctatgcccc
35821 ttaatgaatc gaatggtcct tcgaccttcg tttcattccg acagccggca tagatctcat
35881 gagacctgcc cactattact acgaaaaagg ccctgccccc tttccttcgc tactaggaga
35941 gtaggctact tctattagcg ccggaccttg agtcgaattg atcgtgtcat gtgcaacgtc
36001 tatcaatgat ggttcattaa tgtaaattga tttagactcc ttcccccttc tcaactacga
36061 aaaagatcga gaggggctcg aaagccccca aaccaagaac ggaaacggaa gcctttcgat
36121 tcactcccca ttctatctga aataaaaaaa gctcgccctc gagccctggg ccggtcggcc
36181 gggtctttcc ctgttgttct gttcccgcca cgagaaagac gcacggaaga ggtatgccca
36241 gcgcgcggag gatccattga gagtttgtct cggtaaggaa ctgtacgatc ttttccccta
36301 ttgtattgaa aaaaaaagag gtcagtgcta cggcccccta ttgtttgatc caatattgac
36361 cggggacgag ccccgacttc cataggtcct tggtttgacc tcccgtagtg gtccttgctt
36421 ttaagaaagc ggagcggggc caatttatcg tacttgctca gtgtgccccc tttcgtcgag
36481 tgccccgccc ggttcactgg gctgttggcc taccaagcac ttgcccgccg ctcctgccgc
36541 ccgcttggcg ggcggagcgc tcgttatcct tacttttctc tctgccgggg cccctcccat
36601 tgctctagcc ataagctatg gcagctccag ctcggcaacc acaggggccc gccctgcgag
36661 gccagagtgg gctcagcggt cagccccat tcgaattatg ttagggggtc tttcgctttt
36721 gccagatcta gagagatact acaagtcctc cgtcccagat tccatcgccg cggccatctg
36781 gttcaccggt actaccaaaa agtctcatgc ttacgttact gttactgatg gttttattat
36841 cttggaccac gaagacccca gtcgtgcttc tggtttccat tcccatcata ttgatgataa
36901 atctcctcgt gctctgccat aagaacttgg agtccgtatc atggtgaaga taaggtaacg
36961 ctgtgattct tgctcaaaaa aaagaccgaa cgcgttcgag ttattggctc gtccaacccg

37021 gcagcattca tgagtcgctc gttcaactgt ccctcggaga cacggtcgag aagtaccatg
37081 catggttgcc ccccgtcctt tctttctctc ggtcccaccc agcaagatac ggccccgcca
37141 aaaaacgtga gcgcccctgc gcgagcctta ttttattagt ttagtaaagt aaagctttgg
37201 ctccctaaag actaaagaaa ttcataaaaa agggggactt ctgcaacggg ctatgccacc
37261 caaaccaact gagggaagtc gcatccgtcc catcgcaagc acctacaatg tcatgatcgc
37321 attggtttac cctttttct ttggtagttt aacggacggt cgcccctcca acaagaaaag
37381 gtataaatat ggaaacttct ctgccatttg gatcggagaa tttatggagg aaatcaggtt
37441 ttaaattccc agaaaccgca cgattatata gccaaaggga atacgccgcg cctaaaatca
37501 tcccaagcgc tgctaatgtg gctactaagc tatttctttg gaaagctcct actaagatga
37561 gaaattcccc gataaagctg ctagtaccag gtgaactcat attggccaaa gtggaagaga
37621 agaaaatggt agggagattc ggcatggtgc tcactgaacc tccgtaatat ctaacaagtc
37681 gagtcttatg tcggtcatat agaacaccaa cacatagaaa aagggctgag ggaaccagtc
37741 catgacttga catcggtgga atgctacctc caattccctg tatgttcgat agagccaaag
37801 atcccccccc ggagtacgcc gattaccccc cgaaccgtac aagatagtta ccacctatca
37861 tacggctttc taacttcact tctttttctg gtcgttccgc tctgtcgatc gatggtagta
37921 taagagatct gtaaccccca aaaattgaaa aaagaaaggt tcctgcttcc tacccatagt
37981 tagcaggtat ctcccccggt catactttag tatcacatcg tagaccccca ccccgactct
38041 atctttctta tcagtgaacc ggaacatagt gcataggtac tattcgatgc agcggggacg
38101 agacgacgtg acatactacg atgacgtaca gaagtgctgc ccttcggcaa tatggaacct
38161 ctcaacagct cccgttcctt tatggggttc tgtcgggata ggtaggccca agcctttccc
38221 ctccataata ccctctttct ctttcccctt cgagaaagag aagaaaggat tcatttgttt
38281 ctttcatgtg aacggcccta tcttgtatcg aaaggttttt cgtgctatac accacgttga
38341 gaaagaccaa cctcctatgt accgtactct tttgtacct cgaaagggag gtcctcctta
38401 tgatgctacg gacggctgtc cgaagtgcaa ggggtaaact cggactcgga taggattgtg
38461 cgtgccaggc ccttcgggtg tcatattttg gccgagttta ccgtacctcc ggccccccatg
38521 ttaggctgcc ataatatttt tttacgttct accgctgtta cgccagaaat aaaaggttcc
38581 acacgagctc ccgttctgcg gcgtggtggc aggaccgata ggggattggc tccgggtgta
38641 gataggcttc aatctgcagg ggtcatgcaa atacataggc cccttccctt atcttttgga
38701 tgaatggggt ggtttagaag gcgctttccg atctacggtc cctcggagcg aagggttagg
38761 caggtagtat gggccctctg acttccagct atgtgctgtg cggctcccgc gaagcgaatg
38821 aagggaagct cgaagagctt acgggtgagc ttacgcttac tctcagcctc tttgattggt
38881 aggcgggcgg gctaagcccc ctacttaaga ttcaattcat aaggctcatt ttatgttgtc
38941 gtgacgcttt ggttaggccg actactatag gctacttcta gcttctatag tggcctttcg
39001 actttgatgt agttttagtt tgtattggta ctgaatgaac acatatcaaa caaaggcgag
39061 cagtataagc ccttatccgg cctggaaaaa gcagtgaact ctagaagcta gggctttgtt
39121 caccttttgga cacgaacact attccgttct caacggaaac ccgccttaga gattgaacgt
39181 cgacttatct tattgccgtt caatctaaga cagcgctgat agcttgtagt gaacagccca
39241 gcccccctta tgaaaccaac cgaaagcagc ctttggtact gtacttaagt agttaacctt
39301 gcaactatga tagaaagccg tttgcttgtt gccaaaccct tcgccttttt tttaataaaa
39361 gtaggtcgcg actgttgtat tttagtcggt cgggggaagc taccgcttct acgacagctc
39421 cgcttcctcg tcttgcttct ggcaaagctt ctactttgct tgcttctctc gcgcttgctt
39481 gcgcgaaggc ttagagtcct tttcgctagg tccaaaagct tccgtcaaag caaaagatct
39541 gatccaacag aaatcccgca tcgcatattg agcgcagctg atatgcggct acggctaggc
39601 gatcatatca tgccataaaa aactttttta tgtatagata gaatagatgt tcatggatag

39661 acagacattc cattgattct tagtttttgg gctgaaaaag ctcgtcgatc caaatgaatc
39721 attcttctgg tctctcttcg ctccccgccc cgaaactgac caacagcaca gcgcccattc
39781 gcttcgcgcg cgggaaggct tagaagttca gttcatgaga ccgcagcgga gtggagcagc
39841 cgcgacacga agttccttac cttagctgga tctcgccggt gccacctaaa cggtaggtag
39901 gcggcggatg tgtgacgttt ggagttgtcg ttcgtgcacc tgtccccaat ggaagaatgg
39961 gtgatccgtt cccctgcaga tcatggccgt accactcggt ccccgatggc cagcggggga
40021 ttcggtatag cagctcacgt tcgtttgcgc tgcgcgttcc cgctggactg gacacttgtt
40081 agctgtagga agtatggttc cgaaagtgac ttcggttcgc cagttcaggc tcaactcctc
40141 gctttacgtt agcggaccta caggtcaggc cggggctctg cgctctttct ttctgtgtgg
40201 gacgatgccg gggagagaag ggaatgcgtc gaggcggcga acaatacaac ccaatttttg
40261 cttttccctc catgagatga gcccagtgca ttggaccgat ggataagaga actgagctgg
40321 ccccaaaaga gcttgggcgc tctacgtagg atgtagactc catcagatac atattgattt
40381 ctttctgatg ggatggatca agaatagaaa gttgtctcat caatagatag aaatagggga
40441 tttcccctta ttattgatag atttcctctc tatatctttc gatctatcag aacagatcag
40501 gctttctatc aatcgatttg aaaatagcac gttcgtctcg cttttcgttc atttccgcca
40561 cgccaacata gccgccataa taagaagcag ctagaagcgc tcgcttcacg ccctttcatt
40621 atcattcacg aatgaattgg gaaagccaac agaaagattc gattctgact tcgtagagcc
40681 ggtgctgctg ttgcctgcgc gtagggccgt cccccactcc cgtcccgggg cgctaaggag
40741 tttttgtttt tggaacagac ggcagtgttt tgctgacgcc tcgaatcaag cttttgttgc
40801 gacatgctat gttttctccc ccattgctag taggttgatg ggtcgcacgt ccggcacaca
40861 tgttttggcc ttgtgtgtcc ataactcaaa ataggtgacc taacggccgc cgcccgacta
40921 aacataccaa tagtcaccag attcatatga gctactgggg agtaagcaat gatcttctta
40981 agatcgatct gtcttaaagt ggtcgaggaa gtatatatta tagcaatcgc gcttggagta
41041 taaatgaaag gagtggaaca aagtgttgct tcgggaaaca tgggtattga aaatcttaaa
41101 aacccgtagg ttcccaattt taaaggaatt cctgccaaga tgacggatcc cgccgtaggt
41161 gcctctacat gagcttcggg tgaccaaata tgaactggta ccataggcac tttgacggcg
41221 aaagaggcga aagaagcaat ccatagaaag atttggcgcc gctcactaaa ttctgtggtt
41281 aatgatattt gtaaatcggt ggttcctgtt tggagaagaa tcaacagaat agctaatagc
41341 ataaaaacag atccaagtaa agtataaagg aaaaactgat atgctgcctt gatctttctt
41401 tgtctcgaac cccatacccc tataataatg gtagagtaag gggtggcccc aaagcggaat
41461 tgaccggtgg tggttggtcg aagctccagt aggtagccac tcccttctca gggaaccgta
41521 cgtgagactt ccgcatcata cggctccgtc ccgagcttcc gtcgtcggcc ttgtcattag
41581 accactatct atgcatgtat tttccgcctg gactctcgaa tcctttgctt ctcgggtggt
41641 ggcgaacaat gctgcttgcg ctgcgggaga tgcccgcccg tagggcccgg cttgcttccc
41701 gcccgaaaga accgctcact agctagccgg actagtgggg gccctatctg gagacatact
41761 gaaaaccatt cgaaccgaac gcaacgcccg tcttaaaaaa aaagggctcg ttgggaataa
41821 agatggagtg cggcctgtag agcacttgta acgcacagtc gggttgctca agcagcggat
41881 ctctctctct atagatagag agagagaggt gtgaaagaaa gtaatagcct tatttatgtt
41941 atagccgccc cccgacttac ggcctttacg ctactactac tgtgatgtga gcggttcaaa
42001 tgggtttgat ctttcccaat cattctggcc gaaacttgta cgcagcactt gtacggaggt
42061 gcatgcataa aggtttggaa ctcttttctt atctgaatct taaggacagg accctaccct
42121 attctcgaac cctctgccga gctctaccct gcccttttga aggggccaaa gaaatgtctc
42181 cacctgctgc caacagtctt tcttcggaat tctactgaac gggtcgatcc tcccccaccc
42241 tttgttttag caacttatcc taaggtctcc tcgccaagag ctccccggcg acgacagagg

42301 aagcaacccg cctgctgtgg cggggcggct tttttctttc acaataagac ctggacgatt
42361 atccctaccc tcggtagctt acgagtttac gtccatccct ggtcgggtac agtttccttt
42421 cgatttatcc cttgtagccg ttacccgaat tcgcgcaagt gtgtccacac cccccaaact
42481 gacttgacga ggaatccatt ctcgcgaaca aacacaaccc ctacacacac ctaggagcac
42541 cccttcctgc atatccatac aagacccgag taggcgggtc gaggtcctac gaggtaccca
42601 ctactcggag taccctccca aaaggaaaaa tatgtgtctg tcaggttcgg ttcgtcttag
42661 ttgggttggg cgggttcgac cagaaatgct atgcttacac acaagactac ccctcttccc
42721 gaaagcttcg cggggactac tttacgacga cggacgaccg cccgtagggg gtttactgca
42781 caaggcccct gcagaggaaa agctttttcc cagcgaatat aagatgctca gctccgcaca
42841 acatcgggat tggcacgctt tcgggaagaa catagaatag tagaggatcc agcatgcgga
42901 acacggcgat cattagaaat tcacgaatta gagatgctgt aatatactct ttcccataac
42961 ttctcatacc agaccaaccc actaaaatgc aaatagggat cagaaatgtg gtcaatatca
43021 cgaagaataa agagataccg tctataccca aataaaaatt tatgttttca taaggaagcc
43081 atcgaaggct ttccacaaat tgagatttgg ccgtagaagg atcgaattgt atccgaagaa
43141 caggggaata caaaaaagta ataagagagg cgcacagacc aatcaatcgt atcggtcgta
43201 ttcttgaatt tggaatgaaa agaggagtaa tgcttcctag cacgggacac agaataggac
43261 cacttaaatc agaatagcat tcacagaaat gttctaacat agagtagaat cgaacattga
43321 agagattagc tgacaacagt caaaagacgg gcgcctaatt ccaagacaat aggggtctat
43381 ccgtgcaagt cagctgaaca ccgtgattga tgagtgggta ggtggattag gtttcgcgtc
43441 gatctcttag aagataaatt ggcagtctat tattctaata attcaattcc tgctcacctt
43501 ttccgcgtcc aagcagttct ttcttgcctt tcttttataa cataaaaaga ccctcctgct
43561 acgaaatgaa atggagaata tgcgctaccg cttccagata atattcgctt tgtatacctt
43621 gttccgcaag tgagttataa atgttttgaa gaaagtcaat ctcttccatg tcaaaatgga
43681 tatgggcggc cacatcttga atactcattc attcgcgcgg gcaactgcac accctgcgac
43741 cccaaaagct cctctagttt attggagatg agttctttca aggaatcaaa ggcaaaatct
43801 gacaacttct cttccatatt ctattatccg gagttaaacc gtgaacgcag cgtccagcac
43861 gaaaggctat gtacactgat aaagtgattg acatccccat acaagaaaag tagcgatttg
43921 catatctgag acgggtggca tctttctttc gaacaaagga ttccctccag acagaaagag
43981 agtccttcct gtacgagtag tgaagaagta tacttttttt ggttgttttc caagaaaggc
44041 atgggagata gaaagctgcc caaacgaaac tttttccaac caagatgtat gtcgtccgac
44101 ccgacctcag actctttctt cccgacctat ttgactttac tctctggccg cagttattta
44161 ggtggtatcc cgagattgaa gagatcaaat tcctcccggg aacacacgaa acaaagatat
44221 gaactgggta aatagaaatt gaaaagagat gtttccaatt catcaaaatg agaagttttt
44281 tctacatcca tatgatctac taaagtgaaa gtagatcggt attgcccgta taataaaagc
44341 agatcttggt ccatggagtc ccaagaaggt aagaactcca acctgtccga tgcttcttcg
44401 gccaaatacg atatacaagt gggacctgca ctatgagcaa gctgtgcgaa aaaccgcttc
44461 ttttttccgg ttccgaaaca acttgggcga aatagggttc taccgggaaa ccatggattt
44521 tttagttttc gccattggtt actggttgag ccactggaat ggaatgagat aagaatctct
44581 ggagatcttt cctctccact tactcgtaac aggctttccc tctgtataat acttcttccg
44641 aatggcataa ttgtcctatt ttttcctcga tcttcaaaga aaactgactc ctcctactcc
44701 ttcttctcct ttaggatcgt cgttggtcct tctttcacta atgatctcac cattttatag
44761 tcgttcgcgc gagggactat cctttctatc gcttgccctt gcttttcgct ctcaagacaa
44821 ggctctctct tctataaagc gaagcaaagc gcatggcgct gaagtgaaga aagctcaaaa
44881 aagacacacg aacacgatgc agggcatagc ataaatgaaa ccgctgatca tttcataaaa

44941 catatatgct tgacctttct cgatgctttt ttgggggtga ctcaccaacc gacccagcag
45001 tgatcgatga cagaatggta cgaacaaaaa aaaggaattc gatttggtag ttctccatgg
45061 acttctctct ttaccccttt gcatatgttc gtgcaccccc agatgggcgg gggccggcct
45121 cccactccct tatgcagcat ttattagctt agctgggttg ggtggatcgt gcaataagcc
45181 tctctcgacc cttgaacccg aaacaagaaa agcaggatcg ggcgccccgg ggattctttt
45241 ctttcgtgct tgcttatggc aggcccttac tgaactgagg ggtggtatcc gcgcagcgca
45301 agagggcttc ttctttcgcc gcttccgctc cttctatatg gatatggata taaggaaagt
45361 aagctctccc cttttgtttg gctattgacc ggtcttctac tctaccccac ttattatcaa
45421 tgaatcaagg agaattatat ctcttgacct ttattacgag atttcgaaac ctttctcgaa
45481 caaatgaaga aagcccgcat tcaaaatcag tccattatga attaaccatc catccccggg
45541 gaatacttgc ctaatcttat aaagatcaac ctctttctgt ttgaaaagga gcttattcaa
45601 cctctttctg agagcttatg aaagctgggc ctttctattc tataatggtc tcatcttaga
45661 atacctcagg agctaatgaa actcttttag tcaaattaaa ctgtctcaat tcccgggcaa
45721 tcgtaccaaa aactcgagtt cctttttgat ttccttcttg atcaatgaca actgcagcat
45781 tgtcatcata tcgtattatc ataccgttgt aacgtttgac ttcttcacat gtacgtctaa
45841 ttacagctct gatcaattct gatctttcta gaggcatatt gggtactgct tctttgatta
45901 cagcaagaat aacgtcacca atatgagcat atcggcgatt actagctcct atgattcgaa
45961 tacacatcaa ttctcgagcc ccgctgttgt ccgctacatt caaatgggtc taatgttgaa
46021 tcatatcttt tttttatgtt ctttcaatgc aaagggcgaa ggaaataaat atatatccct
46081 tttttgtcca aaaataaaga aacctgctat tgtttttttc atcacaaaga tctctttcct
46141 ttggttccac atccctatcc cgcaataatg aattgagttc tcgtataggc atttttgacg
46201 ccgctattga aatcgctttt atggctacaa tttatgaaac tccgcccatt tcagaaagta
46261 ttcgacctgg tttaacgaca gatacccaat cttcgggaga tcctttcccc gaacccatac
46321 gtgtttccgt aggtcttact gtaattggtt tgtctggaaa taaacgtacc catatttttc
46381 caccacgacg tgtatatcgt ttcattgctc gtcgctccgc ttctatttgt ctagatgtga
46441 tccaagtggg ttcaagtgcc tgaagagcat atctaccgaa acaaatacga ttacctcgat
46501 aagatattcc cttcattctt cctctatgtt gtttacggaa tctggttctt ttggggttct
46561 agtttatggt tctttctcaa ttccatctct actacagaac cggacatgag agtttcttct
46621 catccagctc ctcgcgaata aaaggattca aaaagattaa gatatacatt tatttaatga
46681 ataatacact taatcatggg attctttgag atttcatcta atctattctt tttttttcg
46741 taaaggatcg cccccaattt agcaatcaaa taaaataaag ctttcggcgg gcaagcaccc
46801 ggagtctgtt tagttatcca tagtaaacag gaactacaca ataaacttcc ttgtccacgc
46861 cttctgctgg cttcttcaat tagggtatct gaaatagatt cattagtaga ttgtgtctca
46921 cgcatatacc tttacgaaat aaaaaaggac cagacggcac ctggctgcaa tcagatgtag
46981 gagagaagtt cgtttaggag ccaccgcata cgtttactct ggaatttctt cgtgagattt
47041 gtgatcgccc tttcctcccg cctcgctggt cctccaaatc cccttgagta ttcaaagaaa
47101 aatggtttga tcattcaatg gatcaagcaa ggtgttgatt tttgaatttg aaaatggaag
47161 tcttcttcga aagaaccaga gttagccgag tagctcgagc aggagtttat gaaaagctga
47221 aaaacggaaa actgggaccc ttaaaaaaaa gatattgaaa accagaaacg gaatacctag
47281 gtcgcaaggc aaggaaggac ggaaccccac ttcgactcac caaggagaag gtaagtactg
47341 ttatatcgtt tcttctgaag tgatatagat caatgcctgg attggggggg cttcggccca
47401 cgggaaaacg tttgcagatg cgaccatgaa tcgaaccaga tcgaaacatt cagctgtcga
47461 cggacaaact ttgccggaac gccggccgca aacgaaggat ggccaggccc cggttcccag
47521 ggttagggta ttccctatta tgagcctact cagatcagaa ctaaactagt tgattctctt

47581 tcgcctatcg gccggccggc tttaagcaac cttcgcattt cctgctgaga tcccaagtct
47641 ccaagtgggc cctcttggcc aaccacgacc ttggcttttt agagaatccc gctcctgtgt
47701 cgtaggactt gtagctcggc agtccaccgg gtaggtttgt ttatccttcc agtttcgagt
47761 gtcttcttgg atagttatag cggcccatag gcgcgagatg taccttgtgg gggggcggcg
47821 gtcccctgga catagtcctt tcaggcagtg gccgtttagt ccatggtcca ttggatggtc
47881 ggtgcaaggc cagaaattgg aacacattga ttccgctcgt tcccgtcctt cgcttcaggg
47941 cctgtccctc ggtgtggtca gtactccata ctgtcgggca gcgaagctta cacttgttca
48001 ctaattatga cggttcacca gggcctcttt cctcctccct tttctgctca ctcgtagggg
48061 cccggacccc cacaaagggg gagggagtcg actgaacatc tcagccattg gcgggaattt
48121 cgcccgcatc cgatccccaa ttcttgttca ccccgtatga tcgtgttggg tgaattgtga
48181 cctcgtacga tcgtgtcggg tgagcaacag ccgcttcgtc acagtactta cttatgggct
48241 aacgggtcac actttggcca agtatcctac aaagagactc ccgagagcca gaagtattaa
48301 aggaatggcc ataggaatgg gcgcatcatg acatcgtaag atgtctcgcc cgaatgaatt
48361 agttggtact agaaatgtta gaaaaagtaa acgaaaagag taataagaag tgaaaaggac
48421 agagacactt cccaaccaga aagcaaagtt cccactgatg gtatacttag tgtaagcgag
48481 ctctaagatc acatctttgg aataaaatcc agttggaaaa ggaaatccaa ttagagataa
48541 gctgcccatg agcatcatgg cataggtaaa agggaatgag gaggcaagcc cccccatctt
48601 ccgcatatct tgctcatccg acatggcatg aatcaccgaa cctgcactca ggaatagtaa
48661 tgctttgaaa aaggcgtgat tcattaagtg aaagacgcta accgaatagt tagagatgcc
48721 gcaagcaaag atcatatagc ctaattgact gcaagttgaa taagctatga ccctctttag
48781 atcgttctgt aatattccag tggttgccgc aaggaatgac gtcatagctc ctgcaaaagt
48841 aataacaatc aaagccgtag gtgggtattc aaataaaggg gagcaccttg ctatcatgaa
48901 aacgccagct gttaccatag tagctgcatg aatcaaagcg gatactggag tgggaccctc
48961 catagcatcg ggtgaccaag tatgcgatcc tatctgtgca gatttcccaa cagcaccaat
49021 aagaagtaaa atacaaataa gagttatggc attcaatctc atattgcgag aaatccaaga
49081 atttctgggg gcactagcac gagcaaaaat ggttgaaaag tctactgttt gaaagagagt
49141 aaaacaaccc gaaatcccag gagctaatcc aaaatcacct actcgattga caagcatagc
49201 ttttatagct gctttatctg cctgaagtcg tgtaaaccag aaatgaatta acaaatatga
49261 agcaagacct actccctccc atcccaggaa taattgaaga gagttatctc cagtcaccaa
49321 cattggcata aaaaaagtag gaatggataa ataacacata aatcgagggc tatgcggatc
49381 ctcagacata tatgaaatgg aataaagatg gaccaagcta cttatggatg taaccacaat
49441 taacatcact acggtcgggc tatcgaacac ggggtcagaa gtgaattacg agtcggacca
49501 atctgcgaat cgagcgagct ccccttgcat gcaatgatgt ggtggtgaac ctctcattct
49561 aattcagtgc tctccgaacc gtgcgggaag gtttcccatc acacggctca ccaacttgat
49621 cttccgcggg aaccgtatgt ccgaacaggc ctggaaaaac aggtaaggtc tcgctttcct
49681 ttgccactca agtgtacggc atctgccgtg cttaggcccc ttcttcccta atgaaagagt
49741 ccaccgcctg ctttagtagt ctcaaataag gcgtgcaggc ctgccccttt tttgagtagg
49801 tgattcacta ccgaagcgaa gaaaaggctg gatcaataaa agggggtact acgagccctc
49861 tgccccacgc atctaaccag ctcgcgtggt tcaccggttc caccgactag acccttagag
49921 tgattcagtc gatacagagg tgcgcttgaa gtggggggtg tgctgtccct attgggccgg
49981 gccctccccc ataaggcccc accgtcgggg cataagcgcc ctcttgctac ccatatgcga
50041 ggcgccgtct tagccttccc tgaccaggat cgctcccaca cctgtagcgt tcgtgatcgg
50101 cctcctcaac tgtgtatcga tcgaaaggca gggcggcaca gcccccaacc aagggagtgg
50161 ttacgtccag tatgtccccc cttcttcccg acatgctatg gtgccccgtg gtgggtagga

50221 gcgggtcgag tccatatcgc cgcggagcaa cagccgcgtc cggatctgat ctatctattc
50281 agcaattcat ccggtgactt cacggtcgcc aaagaagccc caagaagcat caaacatttc
50341 cgatgagatc catggagcaa ttcttagata gcaagcacta gctcccggtg cgacttcata
50401 aaaagcaatc aaagagaaga tcgaagagaa tgaaaggcac gtagtggtta ttatggcggt
50461 tccttctgat cctagaaaac gtccgaaaaa acctgctacg gaactaccga gcaggggcaa
50521 aaatacgata agtagataca taatttcgag tgtgatcaga caaccaaaaa tcagacaatg
50581 acagagcggc ctgtgatttt ttgagtgata gattgatcga cgcccgaaga gcgctcgacc
50641 gaaggaatga gtcacaaggt gcatttttag ccccaacgac aaaggaacct acgggcgggg
50701 cattttcggg gagtagcccg cttcccatta ctcaataggg caattcctcg cacataatta
50761 agggagccat tgaaaggtga ctaaaacacc agaaacgggg actacccgag ctaatgatag
50821 aggcaagaac actttccggc caagtcccat taattgatca taacgatatc gtggaaatgc
50881 tgcacggacc catatatata ggaacggaaa cagaatcacc ttgatactaa accggatcga
50941 gcccgggatc ttcttggaaa tgggaagatc taggaaaggc ggccaacctc ctggagagag
51001 cgatgtgcat ggaccaggtg agtagggatg cagctccgtg gaccgctcgt cgggcctgat
51061 aggtggtggt attacaccct tctcaaagga accgtacgtg agactctcgc gtcatacggc
51121 tccgccccgg aatcaggacc tcccctttcc tttgaccaac gggtcctcaa accaacctgt
51181 cctccctttc tattcctcgg caagcggttt ttttcattca ttcattgata ttgattcaag
51241 gtagctgtag cttgcttcca agtccaagcg ctagcggtag aagctagtcg tcagaagcga
51301 acttccgggc cgactacaag actacgacta caatacaagt catgagcgat agcgaagcca
51361 agccgaagag gcttttttct gtcgaaagcc ccaaaactag ctatcttata gcagacaact
51421 aatgcaagcc tactcaacga atctcataag taaaggcctg ttcgcatcgc aactaataga
51481 aaaaacaact actagactag actagtagtt gagtgctcct tgttgttcgg atcttgaccg
51541 ggtccgagct tcccaagctc tatgctgttg gggaactctg caagggtctt accaccttct
51601 tgattgacta tatttgagtc tttggagtac tttgggatta tattccgcgc cgaggatttg
51661 tgcttgtggg ccggggtgaa tattgcagac cagcggatct ggtggtcgac aatcgttcgg
51721 acttggtaaa ggttgtcgcg gcacctgtag taggacagag gacttatcgc gatgcccgcg
51781 gaccaattta cgatgtctcc gtcgctgacg ttcgtcaagc aggccacgtg gattggccag
51841 ggtcttcttc ggctaatgag acctcgatcc cgaagccttc ggagtatctt tttgataggc
51901 gcctctattt gtatggggaa ttcgctgctg atagatctcg cccagtgtcc ccctccttcc
51961 cccgccgcct tccgacccgc gggagtatac aatgacaact tccgaacact catgcccgat
52021 cgtgagactg cctgttgaac gtccgatggc accttgctcc gacctgagct atgcaacaac
52081 gagatccccc ttgatccttg ccggatgtgc ttgacggtcc cccataatac gctcacttgg
52141 ggacttctta ctccagctgt tccaagagtc tccgctagtt gaaccgcgtc cagtagactc
52201 cctgttccgc tcatcccctt cgtcagctct ttgatcggga tactcttacc taggttccta
52261 aacttggaat ggatggcgga gcgtaggtgg caagcagttc tatggatacg gtgctttacc
52321 cgtagacgct tctccagctc tcgcaagaat tgtatgggag tcgtcctcgg agggacttcc
52381 cgaatgaccg taccgaggaa ttctaccgta ctccgtgcag ctattgttgt tgattctgca
52441 gagcctaccc aaaggttcag gccggattgt aggaagtggg cgatccgttt ttgtatttct
52501 atgataagct ctacggcacc cacgattccc agtagtgagt cgtcggcata tcgcgcgtaa
52561 caaatcctta ttaagtaatg gctttttaag ggggccagct tacgggccag gcctctctct
52621 gacctgataa ctagtatcgc ctccccgccc ggctctatca gcaggccctt tcttttgcaa
52681 tacttaaaaa agtatatcat ggcccaatta ttattacagc gttctctacc atagaattcg
52741 gccttcgggg tcaacccggc ggcttctatg aggaaggcgg cgcaaaggag gctcgagggc
52801 ttgttaagga aggcggcaag ggccgacgaa ggggggaaaa cgaaaggcgg tttctggtcc

52861 cccctgagcc ggggggtgct tgtggggggg gtgtgccacg acgaaacaag ggaatgaaag
52921 gccgatttgc gttggatgct ctttaccctc cccacaatga aggctctgtt gtcttgggga
52981 gcgttgaagc ttgcttcttc tccagagttt tcttggtcat caatacgacc tgtccttaat
53041 agaaccgatc tgattctctg aacaatcgga atttcgtact tctgtcggat cctccctatc
53101 tcctgatcga gcttgtgtag gtagatgttg cctggtaggg ccgatagtaa tacactgtgt
53161 gggacggagt aagggccctt ctcacctcct acgagtcgtc cggcggaaaa gactttatga
53221 atggggtaaa agaacttggg atcgtcgatc tcttccttaa agattgggat gagtcgatgt
53281 cggtcgatgg tgtgaaaaca cttcctgatg tcgaattcca aaaaccagcg agaggttccc
53341 cactcttctt tgatccgtct tagggccgag tggcagcctc gacccgagcg gaagtgcgat
53401 gtgtctggaa actcgggatc gtaaatggat tcgagtacca ttctgatcgc ctctttcatg
53461 atcctttcta taggtagaac tactgtgagc ggtctaaact tcgaccctta tttctttctt
53521 catattgtaa aggggagcaa agcccatttt ttgctccctc tattgataga tcaagggccc
53581 tcctgccgtc gtttcagtga ctcatagggc ttccctcagc tcggtctttt tggttcttga
53641 gaatggtcgc catctctccc aggaccggaa tgattatccc tgcccgatgg gtctacatcc
53701 atccctgaat gtcgtcgggt actgttaact tccccgccat tcttgtaact gtcacctgta
53761 atccgcgcag gtgtgtccgc acccccctga gtggacgaga aagaaaggat ttctcggagc
53821 aaccccccag gttccggacc caggagtcaa ctttcccgta tgagcattcg gtacttgtat
53881 cagtccgtgg aagagtgaaa gggtcaccac tactgaggat ctcccccta atcttagata
53941 ggtcgtctga gggttcgccg cggttcattg ctgtgcttac acactaggct accctttcc
54001 gaaagctccg cgggaccacc taccactagt cttcggccgg aggggtttat tgcacaaaaa
54061 cgccgggacg caggctcccg aagagggaag cccaacgaat gtcagatgca aagccccgca
54121 cctcattaag atcatattgg catactctcc caaaaaagaa agagcagacc ccattgaaga
54181 cgagagtgag gtacaacaag gccatttctg tccaccgccc ttctcacgga gccgtacgtg
54241 gacgttaccg ctcatacagc tcccagccag caagcagtta gccttcctct acaaggaatg
54301 gaagtgtgga tgaatcgaca tcaaatagag gaattcggtt tttcttttca gcaataacat
54361 gataagagca tccccttcac aaaaaactac agaccccct attctgccac ttcagcatct
54421 tgtgatcttt gagaagatca ttacgagccc tctcctagaa tgtttttgta gattcaaaaa
54481 attccatggt ggatcaggac gagaatgaat ccgggaatcc aggacatact catcctggag
54541 cttatgctct cctctgggga gggggttttt ttctagagag ggaggtgagt cgagggtagc
54601 ctcccgcttg acctattttt cggagtcgga ggaagatctc tcatctgatg accctgaaca
54661 agaaggagct gctctcgatg tgagatcagc agcaaaagaa agaagaggaa ttggatgccc
54721 cagagcagca gcccccggat aagccttaaa aaaaaaacac acacaactat taacgctctt
54781 tccgcaggtc cggcacgcac agttaagttc aacctttcct tctttctttc tctacgcaat
54841 tctttttcgg atggaagcaa gagaaagagc agcaccctaa ttcctatctt ataccgtgaa
54901 gtgaccctgg ggcacgtctg gctcacaagt tttgtttcca accgccctag cttcaggggg
54961 agacgatcag ctcatatcta tttatattag aaagaatccc tcggggaacg aaggctataa
55021 gagaatcagt agctttctag aggaaaggaa gtacagatga gctctcttct gaccctaagg
55081 gctaactgaa ctcactttta agagggagac aaagatgtga acatgaagtg aagaagttcc
55141 ccagacgtgg gtatggggca cgaacgaaag aaggcttggc tgacaagaaa ggaaaggaca
55201 gacagcaggg aataagaatc tcttgagcca agaaaccaaa gccttggcgc tgcttttagg
55261 gaaggcgaga aagattcaaa ttcagttgga atccacaaaa gaagacagaa aacatgaagt
55321 aagggagaaa gaggagaacc tagggaacgt aacctttgcc ccggactcgg gggttgatag
55381 gtttaggctt acttttcttt ggcaacgtgg tttcgggttc agtgaactga atcctatagt
55441 ataccggcag gtctggtggt agcatcggat tacgggtcac tagcttttta gcagacgcaa

55501 ggtgaagctg ctcctaggct cgcaggcaag ggcggatgag aactaaattg ccttatcttt
55561 tctttccctc ggcctttctg ccctgccgct agcaagaaga ggaagcgaga agagcgcatt
55621 cgaggcttct gattcaattc ccaggcaaga aagaagtgaa cgggaggaaa gtcaggccgt
55681 cgtgagagct gttggtggtt tagtaaggaa ggtcattagg cttaggccgg ggctcaaggg
55741 aaggagtcgt agctgcactt aaagcaagga accaagaggg cgtaagggat agaatgtgtc
55801 attttattaa gaagaagaca gaagttctct tcacccaaga agcctgggcg ccggggctta
55861 gacagcaagg aagttagcat aggcatctcg ccaaaagcac gaaactggtg acccataata
55921 gggcgttggg gagtaggctt tctcttcttt tgaagagggg ttgtcaggtg aagtacttcg
55981 aggaatttga aggtaggtag aaagatgaat aaagactttt aaaaaagagt cttgctaatg
56041 cacattacta tggagtccat atagcaaaat aagagacgag caagggggtc gaaagaaagc
56101 tctttcaatg gccgatagaa gcccagccca gttttcgcct tttttatcgt gttgccttac
56161 taacctatag ggttttggtt tgtctgccca ttcaagcaag ccagagcggg agggataaaa
56221 agggtaccgg gtcaaagaaa agccagtagc tatccagtcg aatgcgagcc aggcacaaaa
56281 gcaagactag tcctcatgat gaaaggcctg ggagtgaggg cttgggaata gcagacacaa
56341 gatcggattc tactttgacc ccacctgcga gggccatggc tactactcga atgaattccg
56401 gcgaaaaagc ccgataggca atctcagatt gttggcattt gagatcagat tacggcaaaa
56461 aagtgaacgg aagctaccct ttaagccgct gggttaggac tcggacctgg cgtgtgagtt
56521 taggcgacgg cgggacatac taccacctat tgatagatca ggatttgaac ctgaatgccc
56581 ttcttcctat ggttagttag gttttttcct atctttcttg actataaagc tatgtagtgc
56641 cagacttctg aaatgtcatc gctggaccag ctatttcatc tttcatttct ggtcagctct
56701 ctcgtctttc cagcaaaaga aaggaatttc gtctactaat agaaaagtat gttctcactg
56761 agcactaact ataaatatca taagagaaga agaaaaagct tctggtttac tgtctttctt
56821 aatttaagta agatagttga tcgagaaacc accgcccaaa aagctttcaa ccttagggtc
56881 aagccggtct ccgtctggca gagtagaaac cgatcgatca cttgaggacg cgctaagaac
56941 cttttctcgc ttgtgtagcc tatgcgaagc aagcctacat agggtacaag atcgaaaaga
57001 atgcattgga tggatgcagt gtccgatttg agattccgca gagcggccct ttctcaaaca
57061 aggtaggtgc tcgtgggacg ggagttgtcg cagtaaaggt agattaccta gtttattctt
57121 ttataagttt caactgggaa tgtgcctata tatgagtctt ttcttatccc gcgggaatga
57181 actaagatag gaaccaggac agggaaagtg ggatagtgaa cccgaaagag agggaatgcc
57241 ttcgaagctg caaggaagtt catcactggt tagctctatc tttgttttgt tttatttgct
57301 ttattgcccc gccgtccatg ctatggagcc tagcggcttg ggggatatag tgcccatttt
57361 ggaagaaata agggtcgaag tttagaccgc tcacagtagt tctacttcca gccgagaaga
57421 ctagtaaaag gaaggaacaa gaatgtgtta tatttcagga gctagatcag ttgccgatga
57481 acaagtcaga attgcctcaa caaaaataga tggaattggg cctaaaaaag ccattcaggt
57541 tcgttatcga ttgggtatca gtgggaatat aaagataaaa gaattaacta agtatcagat
57601 cgaccaaatg gaacaaatga taggtcaaga tcatgttgtt cattgggaat tgaagagggg
57661 agaacgagca gacatcgaac gattcatttc tctttcttgt tatcgtggaa ttcgtcatca
57721 agatggatcg ccattacgcg gtcaacgaac tcatactaat gctaggactt gccgcaagca
57781 aattaggaaa tgaaagaaat ctaccgaaag cccttggtac ttgtctgatc aatcacactg
57841 atagcttcaa tagttcactt acattttttt tagggttttt tgtatttcac cggttactag
57901 aaaagtatcc gtatagtagg cctccttcgc cactccacta gtggctcggc ttcgtctcgc
57961 ttctttcctt tcgcccgcct atcatatcgg ccgtcctaga agctactgct tctgcctctc
58021 taggcttcgc tatcgctcat gactggtcta tggatctctc tatgtagtgg tcggccttct
58081 agtcgcttcc cgaatgcctc ttgactttcc gccaacgtac gctactagga gagtaagcaa

58141 gctagcttgc ttgcattcta gaacggtagc ttccgcgccc ttcctgcctg cttaaatgaa
58201 attgatgtga atgatcgtga cagctcttca aatcctattc agtctgatta gatatgtaac
58261 tgaaacaaag tgattccgtt ctgtctctgt tttattttcg gattccgagg atgagccggc
58321 cgatcctaac atcatttatg aggagccgga cgacgaagcc tcctcctcat agaaagatgt
58381 ctccgacgcg actctcccgg caagaaccac tttttttct ttttttgcgg gttcgggcag
58441 gagggacaaa agagagatgc tttatataag taaaaaaaag cagatactgc cccccatgct
58501 cccacggtcc gcttaccgag gaatagaaag gaaggtccgg gggccagagc aagttgggtt
58561 ggggtataga gccgtaagcg cggtgggggg tgacagagga cgtgctcgta cggttcatat
58621 aaggatatga aaaagtcgtt gcctgatggg aactttcact cctctatatt cgaaatatgc
58681 ctctctagga gcattacgat ctgcagctca aatggtccct tatgaagtat ctattggtct
58741 tattcttatt gtgcgccttg tgagcgcgtt tggatccgcg aaggcaatcg ctcggatgtt
58801 cccctaaccc aacccgggaa cggaccggag ggaaccgcag catggggaat gtccgcgtct
58861 cgtcgcaagg ctcattttga gttgtgggtc atagggcggg cggctttatc tgatcaaggg
58921 ccggggcaca agggtcctgg tactatccag gtgcgaagaa ccccggagat gactgcaatg
58981 agcagaaatc tcacttaccg gcctaaacga cgagcaaaca ctcgaacgtg agagcaaggg
59041 atcacccaac gaatggacga gctcaaaggg gggaggagag agggaggcaa gaaccatgct
59101 ttcagagaag tggcggtccg aatcttatct gaactgcgag aataactgac taagccgtgc
59161 cataaaaggg gtcattctcc aaacgggacg gggccaagcc tttaggttgt ttaagtaggt
59221 tgggtgacag atcggccata ggagtactcc gggatataaa catataaacc agggcaacaa
59281 aagttgagca tacaacgatg ccgccccgttt tcatttcatg gaagtccccg gcagaggaaa
59341 gggctgtagg tgatggcgcg ttctgctttt tctctagaga ggggcgcttt aaaatctggg
59401 tcgtgcgtgg cagctggtat agatgaagaa aggcggaccg cttgaacggg acctattctc
59461 ttaataggcg gcaaggctga ataggaaagg ggccgagctg actgatgagt tcctttttag
59521 cctttttcta aaggctctca tgtggagcta acatggctgg ctacatacaa gtatagccaa
59581 atcaagatga gacggtcaga ggccgcagcg ggacttccat aggaaagccc gcccccgcaa
59641 gctaacatag aaagagattc ccggatagta gtagtctcta tgtgaatctc ttcccgaccg
59701 ggccaggcca ccacttggga tgggaatggc tcagcccaca tgcatcattt gatgaaagca
59761 ctccggtcgc ctcctcgaag tggtttgtca accacgctct ccctccctca aaacaatgct
59821 cctcaccaag tgcccttcct tgggttgggg caacacagca atgagtagtt cgctccagga
59881 gcccaccccct cgagagcagg atgccggccg agatggagct gggagccagc ctatactttc
59941 ctggggcttg ccttgcctca acatctaaat aaagaaaggg cgggcgccga aaaggaacca
60001 gcccagcctc ttcaagaaag ctttgcttgg taggcgctgc ctactcactc ggacaatgct
60061 ctgaacacga aagtgtgcag ttccaccccc ttctcccatg ctgagtcacc ggcagcgcct
60121 cggaaagcac ggacgagcca catgcaggga aacttgcacg tgtggttctg gccgggggcc
60181 ccggtatact gtactaatat gtgtaggtcc ccgtaattcg agtgagattg tcatggcgca
60241 aaagcagata tggtccggta ttcccttgtt ccctgtattg gttatgttct ttatttcttg
60301 tctagcagaa actaatcgag ctccgtttga tctcccagaa gcggaagctg aatcagttgc
60361 aggctataat gtagaatatg cgcgggatgc gatccttaat agttcactgt tggcggaagc
60421 caatgtcccg gggttccggg gactcattct gactaaaaca aggggcgggt ctttaccaac
60481 tttaaaaccg tcgattttag ggaagccaaa aaacgtgagc gcctagcgcg agccttattg
60541 aaaaaggccc tttactaata taatagaagg taaggccggc tttcgagctg tagctttaca
60601 acgatagggc ttagataagg gcccttcatt attattagta agataggttg cttgagcgca
60661 ttatcccctt ttctattttt aaggaaaagg tatcgatcaa ggcttttctt gagttttcaa
60721 taagggacaa caagcaacgg attgagctga aagcccttgc tctaatgcct cgttacgtcg

60781 tatacttcac tcgctcgtta gcgcttgact aatagaaagc aaagggcttt tcttgcttgt
60841 ttagtaaagt ctcgcttttt tataggagta ggtgcttgct agggcggagc actcttcatt
60901 cgaaactaat gaatgtcggg tcgggggttt ctgccttgtt atcaaaaagg gagttgggta
60961 aagcaaactc cctccttgga cgagcacggg cttagtcaag tagaaccggg ttgcgctgct
61021 tgatgctccg atcgaaaaca aataagtggg gccatgccgg tacgactgaa tatgcgttag
61081 aaaggtcaat ccctccccta cgacaccaaa aaaccgggcc gaacttccgc ccgcttccat
61141 ataacaatat cgtggcaacg tagacctaag tggtcatatt ggatccttgg gaaccatcac
61201 aagtacggcc ggcctatatt tatttgaacg ataatgccgt gtcgtccagc ggagcctaga
61261 agaaggtgac tcgcgcagac agctgactcc ttttcaatag aaaagaaaag ccaaaccaac
61321 ccagctggct ggtcaatctc agaaatattc tcggccggca aaccggagac ggacgaccac
61381 ggtcccgacc ttaccagcac ctgaggtgta ctaatcaatg aaccccсgaa acctacttta
61441 ttttctgaca aaataaacca agcctaaccg gtcacgatat gttgttgata ttgattgagt
61501 tggggggggc tttcgctgac tgaatccatc cgaccccggt aaccttcgta accaaagcgt
61561 cacgacaaca tccaaaggta acctttggat tcttaagtgg gggggcaagg aggagcacgt
61621 aggaatgccg accactacat aagccactag tggctgagag aaagcgagca gcaccttgta
61681 taggttgggc ggagcttgaa gaagcgagcc cctatcagag aatgccattg cgcgctagcc
61741 tagctagcta acgtttttca gctggctgtt atgttagttg tagcgcgcct tcccccсttc
61801 tctcacttcg ggaagattta gcagtatttt cttatgatga cctggtcgag agagtacgat
61861 acatcggtgt aaaagattga gttggaattc acgtgacctt ctatcctgat cagcaaattg
61921 attcattctt tttgcgggta acaacgggct aaatcaacat ttcttcgcca cttttgcgag
61981 aaattgtggg cacgaggaga cctcttgcaa gaagaaggta agggcttttt tcggtatacc
62041 gctccgcgag cagagcgaat gagtcaaaga attacccaaa atgaaacgta aggcagaatc
62101 tcgacaaagg ttcagatcca taaaggacct ttatgatagt acttcttcct tactcattca
62161 caaattcctc tttgtttatg ctactcactc tcagtttggt cctacttttg gttcattttg
62221 ttaggaggaa gaaagaaagt aagtaccaaa tgcggaaggt ggatagtctt tgcgagcaag
62281 gtctgaggta gtcaacggcc ttctctttcc cttccaagag tcatcgtctt ttgccagaac
62341 gtcttcaaag aagaaatttg agtctttttg ttgaattgat tcgggcgctc aaaggtagac
62401 tccaaaagtc gcaagatcat gccgagaaag tcgaaaagcc acaggctctc gttcatctta
62461 ccagtctaca agaaagaagg caatgcgcag tgagtgccag gtagagcagc ggggtagtaa
62521 ggtacgagaa gcggtggtat cgggttgagc agccaaagct gtcaggcgag caatggtccc
62581 gagtagttca atcagacccg caagagaaga agcgggcggt aaacaaactt aagcaagagt
62641 gaagtaaaca gaaggatgaa gtaagtgcaa caagggccag tcaacgagaa aaaggacctt
62701 cactctcgtt ctctgtcttc gagtcttggt tcagttcgtt cctctctcgc cctatcttac
62761 taataggagt tgatccgtgt agcaatcaat gtctgaggac atagaaaacc ttaacaggga
62821 ttcgataaac gaaagtatgt atactgtacg ggacagagcc aagggcgaaa ggtcagaaat
62881 taaagaacgc attcgggtca agactataac gcgctatccg gattccccta cctcaacacg
62941 ctcgctcgct tctcgagaga cttgattgga acagaggaag aaggctccac attactgcca
63001 cttcaccaga agatcaaggc catgtcacaa ctgaaatacc agaggcactt cctcttagcc
63061 attcactata gttttttctt tttaaagaaa gggagcgaac ttgaaatcca tacataggag
63121 tgcgcgagag cctccgctaa agaaaaggat tgaatgcgcg tacctctgcg agtggcatct
63181 cctaggccta tgctacgatc ctgggattta agcaaggtgg cctgatatgt acgtcctttt
63241 tgccggttca tcaaaccgca ctcgcccccct tattttagtt aggggcttaa taggccgagt
63301 aaggccccat tctgcccttt agagataggg gcgagccagc tagtgccatt tttctttaag
63361 gctgaaagtc ctacatatac cggcggctcc ttcttaatgc caaaagcgga gcggaaggag

63421 aaccgacttt attctttttc gcactgggtt ccaaaattct ttcaagggtg tgatgactca
63481 ctccgagccc cctataaaaa gaaaggcaac cgagacaaaa ctttctctct gactccctac
63541 cccatgctcg gaaattctaa gctatatggg gggctattca ttcctcttct cggaactgaa
63601 ctactttttc tgactgaagc acttagggtg ggccatgaga ccagaaagga cgacctctat
63661 caatgtaagc caggccgttc tctccatcca ggtcgagcaa cttctagtct taaatatctt
63721 taaacattat cgctgcttta cattacataa aaccccctaa aattggatga aatacgagac
63781 aaacgcgagt tacgcctctt cctgggtcaa agttcatagg acccgcctac tagtggaatt
63841 caaaagaaag cttgttcccg ccaactttca gggcctcgct tccttctttt tatgaactat
63901 aaatttcgta tggtaatcta tctcatgtgt ttgcggctta ataagcagct ctccctattt
63961 cgtgttaccg ttaccgcgtg tgcactgttt aagaagtaag aaccccgggc tggctttatg
64021 aactgagcaa aaaaatgtac aaatgctatt ttctctgctt cggaaaagac tttctctctt
64081 tgatgtcaaa gagaccgtac cttcccccga ggaatgggat ccttgtactc ttttttgctc
64141 tatggtcgga tcaatactga tttgttattg tataaaacgc ctgctgctgc cgggaaaccc
64201 gcctctcacc tatgagcaag cacagcatga actgagctta atgcatcctg agaccctctg
64261 gggtcaaatc gagcaagtac ttttcttgta tttaaccaat tggggggaaa gaatgccacg
64321 tgggcatacg tggcatgaac tcgcggttca actacacgga gagtcgcggg actgcttctt
64381 tctcttgtcc atcatagcgg acattactca aaatgggatg cacggggggga atgggccata
64441 gctgccattg ccatcattca tggctggaag ttcccctcat cttcattggg aagttaggta
64501 gttatttgat tcttcccatg cctttcttgg aaaacaacca aaaaaagtat acttcttcac
64561 tactcgtaca ggaaggactc tctttctgtc tggagggaaa cctttgttcg aaagaaagat
64621 gcctcaactg gataaattca cttatttcac acaattcttc tggtcatgcc ttttcctctt
64681 gactttctat attgccatat gcaatgatgg agatggacta cttgggatca gcagaattct
64741 aaaactgcgg aaccaactgg tttcacacca ggagaacaac atccggagca aggaccccaa
64801 cagtttggaa gatattttga gaaaaggttt tagcaccggt gtatcctata tgtactccag
64861 tttattcgaa gtatcccaat ggtgtaacgc cgtcgactta ttgggaaaaa ggaggaggat
64921 ccctttgatc tcttgtttcg gagaaataag tggctcacga ggaatggaaa gaaacatatt
64981 ctatttgatc tcgaagtcct catatagcac ttcttccaat cctggatggg ggatcacttg
65041 taggaatgac ataacgctaa tccatgttcc acacggccaa agaagcttcg gtttttaggt
65101 ggggggggta tttgttgaat tcgtaagtca ctgagtgagt gcttgaagga cttggcctgg
65161 aagaagaaaa tgaaatacga acaaccgcgc tggtcgtaat agatcgactt tcatgctcca
65221 gcgtgaaagt tccatttcag ggaaggacga cgtactatga tactttctgt tttgtcgagc
65281 cttgctttgg tctctgcttt gatggttgta cgtgctaaaa atccggtaca ttccgtttcg
65341 tttctcatct cagtctttcg caacacttca ggtttacttc ttttgttagg tctcgacttc
65401 ttcgctatga tcttcccagt agtttatata ggagctatag ccgtttcatt cctattcgtt
65461 gttatgatgt tccatattca aatagcggag attcacgaag aagtattgcg ctatttacca
65521 gtgagtggta ttattggact gatcttttgg tgggaaatgt tcttcatttt agataatgaa
65581 agcattccat tactaccaac ccaaagaaat acgacctctc tgagatatat ggtttatgcc
65641 ggaaaggtac cgaagttgga ctaatttgga aacattgggc aatttacttt atacttacta
65701 ttccgtctgg tttttggttc ctagtctgat tttattagta gccatgattg gggctatagt
65761 acttactatg cataggacta ctaaggtgaa aagacaggat gtattccgac gaaatgctat
65821 tgattctagg aggactataa tgaggaggac gacagaccca ctcacgatca actaaaagga
65881 gaagtcgcgc cgaaaggagc atattggttc gaatctatat agaaagaatt cattttttta
65941 tacttcttac ttactattag agtgaaagca agcaaataca ctttagctct atggctcatt
66001 aagagcggga tccggggtaa gaaaggtgaa ttgaactact gaataaataa gagaagcatt

66061 gggatttcct tgatttaagg gctttagcgt ctcattgcgg aagcagaagt gaaatcattc
66121 ctcattcact tcgagccata gatgatagca tcgagggatc cgctcttatt gctagctcct
66181 ttttgcctgg ctggacgtag ctttatgctc ttgaatcctc ctatcacttc cttttttttt
66241 aatagtggga gcaagattga atctttgcaa tagtttccat tataacaaaa gagaaaggga
66301 gaccaatcaa gcttgacacc tcccccatga gagaatctca cagatctgcc gtggtttctt
66361 tgccaaggta tgtaatgaat tagatctgca gaagcccatc ttaccgggtg ttatgattgg
66421 tgaaggaagc aatgtcttct ggaagcgctt cgaatatgag ggcctaccta caatcttttt
66481 gttgtggtaa gataggctat gagctgtagg actgcactga gatgccagat gagagatagt
66541 ggaaaactag ttactctttg ggggctagca tgaacagagc ctttgagaaa gagagtcttg
66601 ccccccgtat tacttactcc acagggcagc gaggagcatg ctgtggaacc aaccaagatg
66661 tatgtcgtcc gacccgacct cagactcttt cttctaaaaa agaattttct tattctatag
66721 atctttattt ttctttttta aatagctgca agaccaaaac ggggtgcaaa gaaccgaaac
66781 cccttctttt aagttcctcc cagatacatg gcttacatac ccggctcaac attcttggaa
66841 aacaatcgaa tcgagggttc ggaggagaat tggccattca atcttgtgaa ctaatcgaga
66901 ccgaaattgg agaaagagat acgaacggag aggaataacc aatcgatgaa agaacatgaa
66961 accattctgt ttcaatagag gtacaagaaa gggttggggg caatgaagta ggtgaagtgg
67021 ttggattttg cgagctgttc caaccactgc tggatgtgat tccaagagcc gaacgagaat
67081 gaataccaca cagaagagtc aagatcaggc tccctaatgg aatgtttaac cgatccattc
67141 cggatcgacc gaattggtac ggaagttgta acatgggaaa accacggaaa acacgactta
67201 tttgaataac ccagtgacct accaattgta gaagtagtat ttttggcaag caataagcac
67261 ttaaataata caattcaagt gtaccatctt ctttctcatt tcgaggaaaa ggtgcggaag
67321 gaaaaggaaa caacggaggg atccgaatcg gacctaaatg ggaatgacat gaaaagtctt
67381 tttcaaaacc tagcattaag ggtgttacga cgatatacga gaggaataga gaaaaactcg
67441 tgattggtgt ggaggggaag atctgtttat gatatagttc aagaaagagt cgtctcattt
67501 ccttacttct tcgttctttc taattcattc acttcaagac tggttctgaa cgccgcataa
67561 catcatcttc aacgaacctc caccgtacgt acctttcggt gcgaccacta aagaattgct
67621 tatacactaa acagctgctt atatacgctt actaaaagac tgactttcat tcattcatta
67681 ggccagcgtg aaactaagaa agagagatgt gcctaaggcg gcatgtacgc aaactgtgca
67741 cgctaagaga agaggagaga tgttcgcaat tactaccaca agagccccct aaaaagaaga
67801 aaattgtcca gtcctggcga gggataggcc tctatccaga tgagagattt aagaagaaag
67861 cgatgtgtgc cccgcctttc ctcgaaaggt ggtcgcaacg ggctttcatg aatctcattt
67921 gcggaagtag agcccattaa agttaaagta aaggtgcgga gcctaaataa gtgggtaggg
67981 gtaaaatttc ctctaaacga gagtcagccg aagatgagag cattcgttca tccttcagtt
68041 caaagcgcca acaggattgg ctacgacaaa cgtaaaccat ttctgacttc caaacaaagt
68101 ttgaacgttc tctaatcaaa tcccaagtct acccgaagac tttttggtgg agtttttaat
68161 tgtcggcctg aaggacgaga gttggggagg agtactgctg cttaggccca ccaccttcat
68221 gcgcaagctc aaatgcttgc gcaattaaag gaggagaaca gccaatgacc gagcttgaga
68281 tggaaataaa gagaatattt gaaagaaata gattcttcct tttatcttcg cagaaggccg
68341 gattgatgca acttatcaat ccttgtggga gcccctatgc gatggcttta atacgccctc
68401 cctattctaa ggagaaatgc caatactatt gaactatagc cccctttacg agatgaactc
68461 aacagagaag aggggaggaa actcaagaaa tagcgtagat aaggaagtgg ctattctttt
68521 tcatgactcc gctgcgctcg aataagctct ttttgcgcaa gaaagggata tgagatagat
68581 accatcaatc ggtaaaaaaa tagagtcact tactggtacc cctgtcactc ttgtgaagat
68641 gcttaaatca aggcctttct aaaaaagaaa gaggtctgat gtgtcactag ccgagttaga

68701 gggctaacct tcaatagact ctttctgcaa ggaaattaca taatttcttt ctaattagaa
68761 taaggaaaat gcatcgaatg ctcttgttac agaataggca gctgtgaggg gaggttagga
68821 ttggacaaga agggctttgc gccttagacg gtcttgtctt tatctttgtg cgaatccagt
68881 gctggtgtcg aaggtttagc ccagcccagt gaatcaagca attgaatcag gaagcgctgc
68941 tagaagagaa tactacattg ccgctgcaag tgcttgagaa tgagttcacg ctcttcttgt
69001 tacagtaagt gctgcaattg aatcagctct tgatgcaata ggagttctta gtgtaaccgc
69061 agttgagtaa atatcagccg tggttgaatc actaagctgt ggtagtgctg gagtactcct
69121 atcccctgct ggtgggaaaa gaaagaagac agaacagcta tttcatcagc tctggaactt
69181 tcagcgagaa gatatctggt tgataagaca agacagactg ccgggcctcc ttctggggcc
69241 ccattttacc ataataatct tcttctttt gtgaagaata ttccaacaaa ccaaagaaaa
69301 cgaccttcta atttaatgtg ttaagccgtc catattgtac tttactgttt gtcaggctaa
69361 cgcttacctt tattataaaa caagaatgga agtcacaatg ggaagctcaa ccgcgtggca
69421 caaaacagtg ggtttgggtt cctggtcttg tcttccttcc ggctaaagct tcaagtctca
69481 atccgaagtc aaagataaaa taatagagaa atagatgccc actcttctcc cggtgtagct
69541 gcaaatcacc tattgagtta cgagagggaa tccacccttt cttttcacac gcacgagttt
69601 cgtcttcccc cctgatctag ttgactcacc ttacgtttcg atcacatagg agacatcgcg
69661 tcaagagcac atggaaagac taatctatct aataaaggct tttcgcttcc gaggaagggg
69721 atttaccaat gggacaatgg gagaaggtct ctggttcagt gccacattaa aggaaggctc
69781 aaaagcaaag tcggtttgaa gtcggacatt gattgattga cgttcgttca aagcaatcag
69841 ttggggaagc tacagcgtaa cagcctggaa aaaaaatccc agaaaaggct tcaatattca
69901 aagatcaatg aagacaagca ttctcgaatt tcccttcact ctgatttacg ctacccggtc
69961 cactcaaagc ttgaaggatg aagaggctcg ggaaagtcga agagatagat cggtcgaggc
70021 ttacctctgt ttttgaacaa gaaggagatt ggtcaaggtt cgttatctta agcattcctg
70081 ttggaacgac tggctggaaa ggcgctatag ctttgccctt cgggaacatc cggacattga
70141 ttctgattct gatggtaata catacttgtt tagcagacct tctgccgaaa gccttcaaaa
70201 gactcttcca gtcgcttacc tacctcgagg agttactctt tctggtgtgc cagtatcaac
70261 atggttttcc cagcttagca aaagggtgct cctaagtcaa aagaagaaag agcttctctt
70321 gaggcgttag agttttccaa gtactggtta tgagtttccc acctggaccc ggactgtgac
70381 ttcttgactt cattgattta cgcaatccct gctcggaact ctaaaagtgg atgtcaaaaa
70441 gtgtcctttt ccgaacctta gaatcagtcg atagggggaa caaggatcta tgtctccaaa
70501 ctaaaatgaa tgggtacgga ccttccagct cttgatgaaa gggttgccga agccccggga
70561 gggcattggt aaacctgagt ttatgaagtg atagctgttc ttttctcctt tccgtcctgt
70621 ccttactggt tttggagctt tctttcttgc tgctgagcta gtgctctaaa aagaagggga
70681 aagcgtcttc agatatatct atatagggtc ccgcggctct catctcaagg cattaatgaa
70741 agctaggtcg atcgatcccc attctcccat tcagggtaaa gcggttatca aggaagaaac
70801 cctcggttaa agcttcaact actcttccaa actcgatacc ttttcaatta catctatttt
70861 ctcactcaac tcaagctcac attcccggaa ctacccaacc tattcattcg ggatagtcta
70921 gtcacgcttt tcagtcttca tccaagaact acaagggcaa ttacatacaa taggccagtg
70981 gaatcaagac ttgaacggta gaaagatggt ttagcccata aactggttta agggtttact
71041 ctctagctcg aaaacctggg aataaacctc catattgaag gtccatatat ttagattttc
71101 cttgcaagcc gattccccag ctcctgaata ggtgcaaaaa tcttaatgta tgccgcccta
71161 caaatttaga ctcaagcctg tctccgattt cgatgaaagc tttgttgaca attcgatcat
71221 tcctaggcct tacccagcta tgctttttaa gatccgcaac ccgaatattt tgtttcggtt
71281 agctggtgct ggttttagag tacgatcgcg cctatactct tctcgacgat catcacgttg

71341 ggaacgatta tgggtggtag cgtctaagcc acctggttcc tctcaattac ctttagaatg
71401 gtggattggt cttccttgtg tctatctttt cttaaagggg gggtattctt tctgagtagt
71461 ttaaagatcg gctcacagat gggtgtgagc tgggcaatga acctgctgat gtattgtagc
71521 ctgctacaaa tttatttctc tgtctttggt accggcatgc cgataattgc tttggctttt
71581 gcaggtcgac ttcgatttct cttctgctga caatgaaccc gagtagctta cctgacgaag
71641 caccaaacct cctcccggct ttatatgctt ttgcggatga agacgaaaag aaagagaata
71701 gcaggcagtg cacctttgcc ttcggtcagc atactccaag gtgagtaaca aatagtcatt
71761 cccaacctcc attgttatag ctttgaagcc tatagctcta gtaccagtac cagaccttct
71821 aagcgggata ttttgagtag aatggcccta cagagcctat tttgctggac gcggaagtag
71881 ggattgttgc acagttcttg tgattgcgct ttcccccatg atgggatttc cctggccagt
71941 ctagatgtcc ctgccctttc gattgcggat tcgagaacaa ccgatgccat acttccacta
72001 attccgtcta ttcgacatcg gcacaggcta aaaaggaacc taatgcctca gatgtcaaag
72061 aaatggatgc ttcctgcttt cagtaaagtg aggcactaca ggtattggat tccgcttgaa
72121 ctaatgaacc aggagttgtt cccagtgaaa aggaatttca ataatggaac taaagaagca
72181 gaatcaatag gggaccatcc cataccatag gaagttgctt cttggaatgc cgtcgttcaa
72241 gcacaggcgg accactgatt caatcttcaa accaaaagcc agatcttgct actagaaagc
72301 cgaaggagca cacaaagcaa acgaagggac tcaccaagca acaacgtagt tgtttgtgta
72361 ggcggaatcg agtttcaaat cataagatga cgaagtgact gcaccaacga atcaagctga
72421 acacatcttt gatccactct acgaactgaa agcgaaatct tgcaaaacaa gcacgcaacg
72481 cccggatctg caagaatggc tatgtaggta aggggatcag cgctatgatc gctttgagtt
72541 ctgctcgaag tttgtatgct tacgtacgac ttgctattcg gctgtagatc gggttctggg
72601 ttcttccctc gaactccccg agtggtattt cgtaatgtaa agctagtcta aagtaaatgg
72661 ctatttagaa gctggaaagt atgatattcc aatgtggatg agccaaggaa gggtgctcgg
72721 ctaagcagca gagggggttc caacatcaga aagagaagaa agagattcag aatctagttc
72781 ggaatcagaa atggggtaac cctcaaccgg ggaatcacct gagatagaat cacctcgatc
72841 gactaagaca gaagaaagag ggcttcttgc tttaaccagt cctggaaaga ctagacggat
72901 agtagggtaa gactgggtaa ctcataacga gtagccaggg cattttataa ctagtgagga
72961 aagatcgaat attggcttcg gaaacgagtt tgctcgctca aaagaattgg aagcgggttt
73021 aataacagag agaagcttta caaagtgggt aggttcctag ctcaacagag gaaggatcaa
73081 aaacagaagg tagctttccc gaggctttac ttaaaggttt ttgaggtact tactcaagta
73141 tgggagggaa gattactcta caaactaaga ctgagagggg ataccccagg gagtggggaa
73201 gacgttaagt tgccagttcc gatcgaagga ataggggaca aggtgctcga ccaactaatc
73261 agtattgggg agaactaaga tagctattca ctaaaggtag attgctttca agctactcgg
73321 ctaactagga tcggagtgga atgaagactc tcctctcgga gtttcactgt gaactgactt
73381 acccccttgac ttctttccct cacatttacc tcacatcaag gagagcattc ttcaagacat
73441 ttgagaagca tcaaggattc cttgttcttt ggaagggaat ccacgggaag tcgaaagata
73501 acttacaggc tttcctcaaa gctcacagct agtcctcttt ttttaataaa attcccaagc
73561 aggcggatta gaaggtaatg tgccaaggtc aaggctaagc ttagtcggat tcttccccta
73621 ggtgaactac ctttagcttt cgagaagaca gtgaaaagac taacttgagg atgtcacgtg
73681 gaagctttcc taaatccatt tgccttctga ccttctgaga tgcctgttct caggttctcg
73741 ggaagaagac aaggaagaat acgcagctag tcttttcaat gttttcaagc ggagccaata
73801 agactgattt cgagatgctt tgaatagcgt agtcaaagta gccaagccgg ctcgaccgta
73861 tacagtttga gggattcgcc tttgcctcag acagaagaac aacggattga acaggacagg
73921 acaaccggga agggaagcct gacaaagtag atatttattt gaacaaagga acttaaaccg

73981 gtaccagaaa ccaaacaaga gattgaattc aagattgaac agatgaccga cctacaataa
74041 gacgaaaata gaattccatt ctctaagacg aactaaaggg atgtctctaa gcagccaagg
74101 ccaagagcaa gcaggaggag tagtcctatc tgcctagaag gattcgataa agagaatgtg
74161 agtgggcagg agattcatag acacaaaaag agaagaccaa aaggagcaga aggcactcag
74221 ttgtttatgt gaaatcaagg agcaacaggt taaactttta cactttcccg aagagagctc
74281 aaagcaataa gatgaaagat gggatgagat gctagcctta gcgcagaaga tcaatcattg
74341 agccttaggc cactaccggg cagcaatgga ggatcataac cacttgagag atgatcctta
74401 cttgaaaagg cgaagacaat acctcttttt tgttgcgaca aatctagttt caaaagccca
74461 tttatcaatc acattttcag gcacaccaga gacatcggac ccatgaaagt ccccctttt
74521 gactaatata cacgggcttc tgagaggatt ctttaagaaa ccaaaacctg caacagagtc
74581 cttcatttct accacttaca agttgagaga tgttcaattc ccaatgacag aagaaggatg
74641 caagctggaa ggacttggac aagatattct tcactcggac aggagatgcg gcgcgcttag
74701 cgtcggaaag atccccctac atgggtgtgc tatcaaacta ggagaagaac agaactaaac
74761 ggatcaattc ctttggccgg tcgtttcgag cttccagttc ttctggattg ctaacgtggt
74821 tcgatgacgc cgacggaagg aagcactgga aattcatcca acttcgatcc cttaaaggca
74881 agtgcgtagg ctatagaatc cctccttcgg ctctaaacag ctactgtgct tatttggtct
74941 atcaactact cggccagctg ctgacctaat tgggagcttt tcagtcaagg tcgtcggatt
75001 tagaggtcct ttctttcgca agggtccaga tcattccatt gtggagaaag ccttccataa
75061 ctagaaacta gaccccctc aagttctact tctacgtcta cacgggtttt gacttcttcc
75121 tgaaagcgtg ccttatccca gtctgttggg aattggtaaa gggattggga tagatgctcc
75181 ttcagccgtg gcctccggag gttccacaag atgacctgcg ggatgagcta gaccagcccc
75241 tcatcagtga agcggagcct ctgtcgtgtc atgagctgaa ggagaggctt actgtccttc
75301 tctcttcctt tggtaggaaa agggcatcga tccgctttgt gaggcggaca gcggacaagc
75361 tgggcctgag gaatgcctcc ccgcgaaaga ggattctaat cctcaaagaa atagaaaagc
75421 taactcccaa cccccgcaag cgtatggcca attctgcgca gaatgcggcg gtcctattaa
75481 tcaataaggt tgcggcatgg gagcaaacca ggagggccag gggtaaagat atatgagaag
75541 ggaggcgggc taagattcca ttcgaagtaa cgtaacagga ttcgatgttg caccatcttc
75601 aactcttctc gggatccgga gtttttcgag aaaaggtccc atccttcaat atcatgattg
75661 ggtcgaccag gccagatcat gagtgaatag aaaatctaaa atgtacatag ctgttccagc
75721 tgaaatactt ggaataattc taccacttct actaggagtc gcctttttag tgctagctga
75781 acgtaaagta atggcttttg tgcaacgtcg aaagggtcct gatgtagtgg gatcatttgg
75841 attgttacaa cctctagcag atggtttgaa attgattcta aaagaaccta tttcaccaag
75901 tagtgctaat ttctcccttt ttagaatggc tccagtggct acatttatgt taagtctggt
75961 cgctcgggcc gttgtacctt ttgattatgg tatggtattg tcagatccga acatagggct
76021 actttatttg tttgccatat cttcgctagg tgtttatgga attattatag caggtcggtc
76081 tagtaattag ggggcggccg ttcggtcgcc tatgatacta ggaccaatag gtcaaaaatg
76141 ggtttgtgcc gcaggtgttg aacgatctac tctacacagg tgtgggctta cagggctagg
76201 gctcataaac cctttctttc attcatcaat agggctctgg tcggccactt ttctgggccg
76261 ggatcgataa gtggaagtca taaaaaagag atctttctct tcgcacctca gatcaagagg
76321 aagggtagct tggcaagctg gcctatataa taataataga aagatataaa tacaaagatt
76381 cgctgtcttt taaccggctg ttcttctttg tcaacgctac taaggaccta taggttcgcc
76441 tacttgactt atgaaagata atgagaacgg gttattcaaa aaggaaaagg cgctacttag
76501 ccctacatta gtagaagtaa gcggctgagt tagcctaccc tacttaatgt attgtatagt
76561 aggcgagcga gttagcgaag acgcttaggt taatagaaaa gagagcgtcg gtgcgtagcc

76621 ttcattctac tacgctacga aaaggttacg aagtcactta gctcgacgtt aggagagtca
76681 agggggaacg ccatacctac atagaagaac cgctgttcgc tgactttcga aggtctaacc
76741 ttttgctccc ctactgaaaa ggggcaaagc cgctttgcac cactgataga ctacttaaga
76801 ttcaattcaa aaggccctac ttagtttcgt aagcctttgt cattgtcagt aaactaagta
76861 gggcccgagg cgccccctgc gaatccgtaa atctgaggag catgccgcaa caaaaggatg
76921 gtcccctatg cattttattc tttccgaaag ggagaaaaag tacccccat catagtgaac
76981 ctctccttgt gatcgggatg aggtagatgc ctcccagccg gggggcggat cgaatcggag
77041 tttccttagg tagccaccga cctacagtta tccttaaact tccgtgcttg gtggagaaga
77101 agcgaacaaa ggtacgctcg cttgctgtct tgttctctgt cgcgaactgg gatcgctcgc
77161 cagctaggtc agattggagc aacattgtat gagaacatat tacccatatt cggggacaag
77221 gggcggaacg acctctcgat ctacttactg cagcccagga ataaaaacgt cgtctaggcg
77281 ttccctgttg ctccgatctc ccctacgcct aggacgttgt ctgggcccaa gagccatagt
77341 gagttgctgt ttccattttt ttctcgttgt tgataccggc aagacccagc cagatgatgt
77401 ctgctggttg gtagtgagag gactcttagt acctgcatac ccaaaagggg gcgctggttc
77461 aaaaacaatc cttttttttt cttgctctcc cttagcagcg ggaaaggagt ctatctatct
77521 gcctagcttt ggtagatttc ccccaacgcg atccacagaa attccacgaa tccattggtg
77581 gataagtccg acgactcagc agcagtgcgg aattgagttt atcgtccggt ggatctgatt
77641 ccatagttag ggggcaatac ataccaaaag gttcgaagaa ggaacgcgca taagagtata
77701 taaccaaccc acccttctgt ataacctatt cacattagtg aagcggctgg atgcataagg
77761 gaagcgcacg tttgtgagac cttagtcggg atgcatgggg gagttcacct acgagcacgg
77821 aagagcgtgg taccgctgcc cctctctaag taaaggtcaa cgtacgtagc agcaaggatg
77881 gtgcattgcc tatttatcgc tcgggagcca aaacgaacac gcctgctacc tactgtactg
77941 gaccctcgac caggcattct acctttgtcg aatcggaacc aataccactg cgctcggggc
78001 tcaacgattt cagcattaag aaaatgaggt tagcagcgaa agaaagagcc cggcattcat
78061 tcatataaat agctgaggct gctttctctt ttagtagact cagctattta atagattcat
78121 tcttcatacc tggtccagcc tcacaaccct tcgcgggttg gtaagaagtc agtcttgttt
78181 ggtaagacgg aattgggcaa agaaaaaaga gtgctcgacc ggaacaacgg ccaacaaaga
78241 ccgtaattac atagataact gctcctcccc ttctccgtct ttaaggcgaa ccgtatggcg
78301 gctggaaaga aagacgacct caccttctcg tagatggtgt cagtgagagc cattttagtc
78361 tcccccgttg accttctttt gtagatagaa tcttcaatga gtggaaacaa gcaaccttac
78421 taataaaggt gttgaataag gccggctttc ttcgcatgct tgagcgcatc tttctcatat
78481 cgatcaaggc tttccttgag ttttcaataa ggttagctag cccgttttct tgcaggagtg
78541 ctaacgcgca cccttacgtt ttcttcgctt gctctgcagt acgagcctca tacagagccg
78601 cgccccttta atatgatgag aagaagaggg gctgaaggcg gctttgctat ctaagcgaag
78661 gggccgccct cttttccgca ccaatccttt atttactact acatcttttt tggggtgtat
78721 agctcagttg gtagagcatt gggcttttaa cctaatggtc gcaggttcaa gtcctgctat
78781 acccaaacct accttactat aaactatgag taaggatgcg tagtagcgtt caaagatcac
78841 tctttggcct ttcctttaga cgcgcttcgg cggcggatag cagcatgggc aaagcactct
78901 gctgcggcga gcagccggtt cttattgcat tcatcaagat agtctttctc gcttctgaac
78961 tctgcggcga gttcagccac cacctccggg ccgtttttaa gatattaagg aagaatcacc
79021 tctatgtaaa gaaagagaaa tgctcttttg gacagacgga aatcctattc ctagggcatt
79081 ggatgggcat cagagccatc gaggagtggc aagcacctgt gagctaagat cgtttttagg
79141 gctcgtgaac tattaccgaa gattcatcgt aagttactcg aagagggctg ctcaactcaa
79201 aaatatccta aactcaagaa ggacgtacgg accgatcatg gcactgcact tttaggaaga

79261 gtgtcagtca aagagctttt aaaggaccta aagcaggcag tgattgatga ccccgtattg
79321 cagttgccag attacactaa gccatttgaa gtacacacgg atgggtcaga ctgtgccata
79381 ggcggtgtgc tagtacaata aggtaaccct atcgcatatg agagccgaaa gctcaatgag
79441 accgagaggc gcggtccaag agaaggagat gacaagcaat agtgcactgc ttgagaacgt
79501 ggaggcgggt cacgattcgt ggtcaagacg gagaatgtgg cgacgagtga cttcctgatc
79561 cagaagaaag tcacgccaaa acagggacgg tggcagacaa acttgccaag tttgattgat
79621 tttgtgctag agtataagct tggacggacc aaccaggtcg cggatgcctt aagtaggaag
79681 gcagagctgg cggcatttaa gtgtgaggca gtggcagcca ccagcaaggt cacgagtacc
79741 ctaccgcatc gtattcgaga tgggcttgaa aaaaaaagga cccgcgcaaa tttgcaccaa
79801 gctaaggaat gcaaaactcg gcttggatca aggatgggct cttggtcaca aaaaaagtaa
79861 tagacttttg atattccaaa acctttcttt ttcggtatgc cgcccccgcga gcaaggagtg
79921 ccacgcacaa gcggagcgaa aacaaagcaa gggaaatttt tctatttttt gggggaaatc
79981 ctttgattgt gtatggaata tagatccatg tctttcttgt tccactagct aggaccaaat
80041 tctcacatgt ccgtttcgtt attacaacct tctttttga tgtcaaagac cagaagctac
80101 gcgcaaattc tcattggatc tcggttgttt ttaacagcga tggctattca tttaagtctt
80161 cgggtagcac cactagatct tcaacaaggt ggaaattctc gtattccgta tgtacatgtt
80221 cctgcggctc ggatgagtat tattgtttat atcgctacgg ctataaacac tttcttgttc
80281 ctattaacaa aacatcccct ttttcttcgc tcttccggaa ccggtacaga aataggtgct
80341 ttttctacgc tgtttacctt agttactggg gggtttcggg gaagacctat gtggggcacc
80401 tttcgggtgt gggatgctcg tttaacctct gtattcatct cgttccttat ttacctgggt
80461 gcactgcgtt ttcaaaagct ttctgtcgaa ccggctccta tttcaatccg tgttggaccg
80521 atcgatatac caataatcaa gtcttcagtc aactggtgga atacatcgca tcaacctggg
80581 agcattagcc gatctggtac atcaatacat gttcctatgc ccattccaat cttgtctaac
80641 tttgctaact cccccttctc aacccgtatc ttgttcgttc tggaaacacg tcttcctatt
80701 ccatcttttc tcgaatcttc tttaacggaa gaaatagaag ctcgagaagg aataccaaaa
80761 cctagttcac tcgctgagtc tctttgcatc cacggctgaa tggttaaagc gcccaaccta
80821 tacaaggggc aaattcgtag gttcgattcc tgctggatgc acttggaacg ttcagttcaa
80881 tcgctgctca cggaatttat acatatagct cgcccttata gcttatgggg tacttcactc
80941 gctcaacact cgttcttcac tcgctaggga aagaaaaagg atagatgact gaaaatcccg
81001 cttagagatc gcaactatag tcagagtagg agttcccatc catcatctcc gtcgaggcct
81061 cgttttacct gccaattacg gtggatccgt cggattgaaa cctccattat attcggcaac
81121 taaggacgag attaccattg catcggcttt gatgtcccga atgttctcgg tcgccttgac
81181 cgggctcttc gccgtataac ttttacccgt tcagctggag cacagctaga ctttcatcgc
81241 tttcactaga accagagtca taggggcact tttttttcc ttccttaagt ccagggatga
81301 cccctatgtt ttttcgtgga gcgcagaatt tgccttaatc ggcgatagta tatacaatct
81361 atttcactgg caagagcatg attgagggct ttctactgtg gtctctaact ctttaattgg
81421 tcattggctc tacctagact agatggtgcc attaaactag ctgccagaac gagttcaaga
81481 gatgaggatc caggtagtga agtcaccctc ggggaaagaa acatgcgcca ttcttagtta
81541 gggagagagt atgttcacat tgagtatcct cgtatgctca gtcgagtgac aaacgtacct
81601 atccccgacg tgggtaacgc atctacaaaa ccatggtgtt aacaaggctc tcccttctcc
81661 taaatcccat ttttttttca aaaagaaccg cttgatactt gaaaactgga attttttcca
81721 agaaagtttg gatccctcct atgttgtaaa aggaaataaa gcaaaaattc ctttccttat
81781 ctttactgat acagatgatt gttcgggcat ctatctcctc tccaatctct actaattatt
81841 ataagtttag tcagaaggct tctatccctc gaggcaggaa cgtgggatag aggcggtaga

81901 gagagaatac tgaatctacc ctcctttcca ctaagaagct tgattctcga tcgctccttc
81961 cactaataat ccgaggcagt ctctggtcca gtcttctata ctctctatgg aggagtaaat
82021 acgatagact gacttatcta gtcaaaataa atgatggctt tcaagcttta ctaatagggg
82081 catagctgga gtcaaaggta tttgactctt attccggcat ttgaataaat cttctattac
82141 ttactgcttt ttttagggct ttttgcaaag aatggttagg acagagcata gccccttgga
82201 aggaggactc tctagaggag tcgagttcaa ggcatcaata gtcgtccttt ccctcaccca
82261 atctcatgag aagcaagccc agcaggccct gccttaacct gtccccagac agccagccct
82321 caccaggctg ctggcattgc taaatgctcc gccacgaagc aagctctccc gaatacgaca
82381 gatgcggaag tggggaagcc ggaagttact aaagaagtcg gaggaagaaa gtagttggac
82441 aactgtatac acgagggtac attagtattc tgggaattgg caacattgac agaaagggga
82501 aggggtagga aaaagctttt ctaggtgtag ctatactaaa gtagtcggcc gttcccgtaa
82561 ccaagttttt ctttctcact tctacttttt ctgaagtgtg gggcaaatgg cgccctctac
82621 ttctacttct aactaaaggc gaagaaccgg cattgcaagc aaatagagag cccccgcccg
82681 tttgagccat tgcgagccgg aaagcgtacc ggcagtttga gtcgcgaaag ggccgcttgc
82741 ttaacttcat tctatttttc tcattttatt tcatttcata ataatagata gataatatta
82801 ttattatcta taaacaagaa atgaaaaata gaaaattttt tccaattatt cattcctggg
82861 aagaggaaga agcagataga gcaaaggcct cctctttccg tccgctcttc ccgaagtgag
82921 cgaattgcat gtagagatcc gtacgtaggg gcttatagtt taattggttg aaacgtaccg
82981 ctcataacgg ttatattgta ggttcgagcc ctactaagcc taccaccccc ttctcttcac
83041 ctgatacaaa aggcagtcga agtccccgcc gccctgcaga tctcaatcta gcgacggcac
83101 ctggaactac actgctgccg ctgcccttcg ggcacgcctc ctacgcttat cactcaccta
83161 cgctcttgca gcatgccccc ttcgggcaat acggctttct aatacgcgaa gcagctgagc
83221 gatagctctt cgatcgctat attcttgcga tagcgaagga tcgaagagcg aagttacggc
83281 tttaggcgaa gagcgatagc gaaagcctta attattgttt tgttcccgaa caacgatcat
83341 tcattacggc cggcccgacc aaagactgaa agcccggtcc gggcaagcta gattatggaa
83401 ctgatctgac cgaactgggt ctaatggaac aagatctcga tctcaataag gaatttgaat
83461 ctggaagggc cggcaaaaag aagtgcgata gcgaggttga gcagtagcga ggggcgatat
83521 agcgaaggcg tggttcatcc tctaagagag agtagatgcg aaggttcgga tcgaagatct
83581 tcgcgagacg ccccatgaat ctcgaggatc gagcgcgggg cttgaagacc ctgccgcatt
83641 tcggccccgg ggcaccatag catgtcggga agaagggggg acatactgga cgtaaccact
83701 cccttggttg ggggctgtgc cgccccctatc cttccaattt tgtgattccc aggtattttt
83761 gattctcact gttggaagat tattgaagac gacctggtca gggcggttca caatatcttt
83821 gctgaaggcg ctgttaagcc ctaagtttga attccaattt cattgccctt attccgaaag
83881 tctaaagtcc ttaacatttt caccgattcc aacctaaagg agagctttgt cactttttct
83941 aaaaaatgat ctctagctcg aagaatgagt tggattctcc ctatcctttt tttaaggaat
84001 aaggggcttt tgttaaagga ctaatgtaga gaagatctcc ttggctcacg aggtggcttt
84061 aagtgatagg ggatttaaac cgaaaggctt caaaggaggg gggtcaaaag aaactcagga
84121 tatggcaaaa gcctcttttt tttttggatc gaattggctt tttaggagta atgaaaaaag
84181 tcggcttttg tgataaacag cggaatttca aacttttttt gtaatgtaat gaacactttg
84241 gagtgcttgt ggatggtgtg ccacataggt actttccagc tgcttctcga gggctgcgac
84301 aagggttcca aacctagcct attttagctg aagaagtgct aagccggggc ctacaggagt
84361 tggtatcatt gagaatgata acacctttac attgttcgag atcgtgcccc gcactctccc
84421 actttctctt tgcgaacgat gttcgaatat tagataatgg ccataaacgg aatcttaaga
84481 agctgattct aatgttgtaa tggccaaaag gtcaatttcg ataagagcca gtgcttcctc

84541 tcgaacagag ctcctcgcag aaattccaga tcattgaaga ggaataaaaa atggtagttt
84601 tcctataaag tacccgggtg ttcccttgtc cctcaagaga ataaagagag cacattgcct
84661 tcctctactg gataaaatga aaaagagaat ctcaggttgg aaaagcaaat tgttgtcctc
84721 cggaggtcga ctaacgctta ttaaacatgt tctagtcttc cgatgcatat gttaaagttt
84781 tttccttaat tagccggcct acacttcggc tactaataag gggataagac cttcaaacta
84841 gattggagat gatcccttct ctccagtgag tgcagtgaag gactctcgtc tcacccgccg
84901 gtttacttat ggcatcatcg acttgctttt caatctaagc gatttcataa ccagaaagaa
84961 agacctctct ttcgggatca gtcccgtccc tagatgtagt aggcaacctt tctttctcgc
85021 ccttcttcca ttccagcttt ttccaaataa aagaaagggc taatagagtt gcgctttctt
85081 agtaattgcc tttcattacc aacctgtcgt tttacagcac gaagttacct gatgttcgct
85141 actactaaaa agggcggaga gatgttaacc tttcgctatt agtaagcact cccctagttt
85201 tgacctaatg agctttcttt acgttgagtt aacccggcgg gttcgcctag atgtctctag
85261 taaagaacga gctggtggga aagagctgga gatcatgaaa agcatttcca cacaaggagg
85321 caggcggccg ggaagctttg cttctagaat gccgactaca tgggaacaga tgttttctaa
85381 gtttcttcta attcttgctg cccgctcgat ttcgagatgt tcccgagaag aaagagctcg
85441 cgagaaagct cgtcagtgga aattgtaaaa agtctcgatt ccgaggaagg tgtagtcggg
85501 gtcgaccaca atttcccaag taagagatag aatctcacta attggaagga aaaggagggc
85561 ttcgatccat tgtgattcgg ttccttggtg tggagagatc tctgccataa aagagagagc
85621 tcgtaggcct tattctgcag atcaaagagc gacttcccca ttcattcgaa agagtctttt
85681 agtatagaac agaggcattc tgggccgact acatgcgcat ctagtgcagt ggcttggaag
85741 taagctacct tgaccatctt ccgaagttct aaagaatcta ctgatcaaag gctgtaaggg
85801 cggactgctc tacattcagc cacaccacag tgacccccga agcgatcttc ttctagttgc
85861 gggacgaaat ccgacagcca attgctggct ctgaataacc agcccagcaa taagctcaat
85921 tcttccataa cataacgggg cggggttgcg cgcgagccga gtgacgtagg tgcacaagag
85981 tacttcgcgc cacaaccatc tcttttttat aggttctacg ggccgatgcc tgctgcttca
86041 tctgggagaa aagaatcata gatatgccgg tcattagaag gaagaaccgc cataaaaaga
86101 ttcctcgtgt atcatctgta gcaaaactat gaacgggagc tagcaatccg gaccgtattg
86161 aagaggttcc tgagacacag catggaagag tcacaatatt aagaaacgag gtccaagaat
86221 gaagaagggg tagaattact gaatgaatac gagctgtggc taatacccga ggcataaaag
86281 aagcattttc tacgggatcc cgaaaccacc agccaccccg acctaattca tgatgagccc
86341 accaacttcc tggcaaaatg cctacggtta aaaaccaccg acatgtcaag atccaaattc
86401 gaattggttc ctggtcctgg tcagagacca ctgtgttcgc gccggcggtc caacaaagag
86461 gcgaagtagt ggtctctttc tttccattac gaacgacacg cttcgcctgc tccctccccg
86521 tgtccactag cgctcctgtc cagagcgaag agaaggcgaa aaagcgccgc cgaagcagca
86581 taagcaggct tctattgcta cgtaacaata aagcaggata gcattttgcg cccacatgtt
86641 ttaatttgag ggtaaagagc tcgcttgtta tacgggatcc ggcgtatcca gcagagcgaa
86701 acaacgttcc attcttttcg gcggcatcct tccgcattgg cggcgagtgg agtgccacaa
86761 tcccattcat catttttgat ctacataagc caaagcccat agcactggcg acgtctccgg
86821 cataaatgca aggaggatgt atagctgata taggatcttg tggaacagga tttgattctg
86881 caagcggttc ggtacgaacg aataaatttc gaacaaaagg gtcggaactc gctgatagga
86941 aaggagagaa aaacaaagca atgccaagag ctccgtcaat ccgctgttca tcgatagacg
87001 aagctctctc tttatcatct cgtgtcagat gcaacaaagg actcatcctt tttccttctc
87061 gcgaaccacg ggagcgccta gcgcccagag gagcaaagcg cattttcctt tcagggtaaa
87121 gcggcgcata aacataaaaa agggctggcc cgtcaaaagt ccggttcctt cgcgaacgaa

87181 gttcagaatc aacaaaggtt cgtagaacga agggagtgta caactggggc gcagccctac
87241 ttttttttc gtaacgaggg atagatagaa tggagttctt cacgaagttc gagacaaagg
87301 aataaaaaag agtttctcta tggcctcctc gttttgagac attatggctt tggggtcgac
87361 cccggtaaca aagaaggaat ccataaaaac ttaggatccg gcaccatgat aaaatactac
87421 cctcatgatt agaccatgtc cctgagattt gataaaagaa aggtgcatta gcggttaata
87481 cgttgtaatt ggataagtta ttaggaatat gacggaacga aagaccaagg aaagaaagaa
87541 gaatgcacca aaatgcaggt gctgcaccaa acactggtgg ttgtttcttg ttgtaagtga
87601 atgcaacgaa aagacccgga aataacgaat aatgaaacaa ttcatatatt gacatttcgt
87661 gctaatttaa aaatttctgc tttgttattc ccatcatccg gtaaccacag gatgatccac
87721 aagaaaggtg gcaggattcg aacctatggc cggcccaccc ctgacctgct gggttgggtg
87781 gccgggttag cacccctcgt cgcctctgta cccgaaacag atgcgctgcg ctacccagcg
87841 cgtaaccttg tctcccctac ctctcttctg gttgtgccat tacccatcgc gggtaacccc
87901 cggtccggcc gcccctgacc taataagaac gattatcctt atgaccaaac aaggaccagc
87961 ttacttactt ctcgagcgat agttccacga tcccgaccag caacttgctt gttgggagta
88021 ggggcatcca agcttgccca acctatacaa ggggcttgga gatagagggt tttctggggg
88081 agatactgtg tttccaggtt ggatttttta gagaaaatag gactagttgg gtagatagag
88141 gtggtgaaat ctaacctttg catcgatgtt tagcagggcg ggtcgcttga gtgtcaaaac
88201 caagcggtgg ttgtttttcc ttggcttatc gaaccagcgt atgcctattc ctcctttgag
88261 gactcccagt agagaaagcc tcaattttcc gatgtggatt gaaaggaagt tggggatgga
88321 catagatcct tccgcctatc cggagtgttg gaaagaaagc aatggttttt ttctttctta
88381 tttcccgatc attggaagca atcttcactg gcacaaggat ctcctcacag caacctccac
88441 tacgatagaa cccgacaatg agtttacgaa ggcttcgagt agtgcgggat aggctaatca
88501 ccagactgct ctggaatagg ttaatcgccg gagaaaataa cgagtgaatc tagtttcgag
88561 agcatgcctt attctaatag ggggcgtaga gtttctaagt gaaggcaagc gcaactatct
88621 atttcatatc ctccctgtca gcaaggcagg tccgctataa agcccaccgg ccagaaagtg
88681 ctcaagaacg agaaagccga ccaaaagact attccataac caactcttgt tgccgaatcg
88741 agggggcttg gcttgtacca ggctctaaaa gacctttctt cgagcgagcg gtctttctat
88801 ctttttgggt tgcatgccca aggcaatgct ttttttagat tgatcttcgc tatcgtgcct
88861 cttccttgta ccagttgatg ctgcggcagt gcctaatatg actttgctcc tgcctcagac
88921 agctttgagg ttcccatcga tcgattacag aggtttcaac cactgaactt gcttatgctc
88981 ccctttgatc gagtgctatt tctataaaaa agattgagta ggaaaatctt gaattggctt
89041 caatcgagat tgtctcggct tcttaggcac atgaggaacc ggcctaacat cttttcaatc
89101 gaaatcccaa tcaaagacaa gttctaccaa ggccagaatc tgaaagagaa gattcacttt
89161 ccggaattcc aagtgacatg attccttcag aagctaaagt tgaatgatcg aagtctcctt
89221 caggttcagt cgaaaagatc gaaacgaagt catcaattca accattcgca tggtctcccc
89281 taagacttac ctcttttcca aatgaaccga acctcgatac cacattcatc aaagagagac
89341 ggccatctct ctaggttgca caaccccttt agatcgttct attcgtgctt gaaaaacgac
89401 cccgtcggct cgacattttt gcaaacaccg acccttactc aatagggcaa ttcaaagagg
89461 agaacgagga cagctgacta ccgctaaaag taagactacg agtacacatt gtatgattca
89521 aaactgctgc tgcatactac ccggtgcttg caggtctgac tggtgccttc tctccaacag
89581 ctgctttaat caatgttaag tctgactacg aggcttctta gcccgcagct gactacttat
89641 tgaaagaccg aacaggaaat gaaaagtcgt actacaacaa ctgtaaacat tccctccccg
89701 ctctgacttt gatcgacttg cccgcacagc gtcttttttg agagaagaag tcaaggggct
89761 aagtgactct caaaagtcgt cttttgtatc gcgccaagag aaatgacata gataagatca

89821 ttgcttaaag agtttcattg tcgaattttt ctcatcccaa tagtagctgc tgcccaaagg
89881 tgctttatga aagccggtcc acagcagtga aagtacgatg gattccgtcg atcgaacgaa
89941 aacagcttct tgcttttttt tgcctctctg gcttgactac tctgcttgct taacacaagt
90001 cttatttaac cttcacggac cacttcacta cccagcaagc gtaggctcga aagcaacaag
90061 cagcggatta agcaacttgc gcgaaacttt agtagttctc cataaggggc aacaagcaac
90121 ggattgagct gaaagccctt gctctaatgc ctcgttacgt atacttcact cgctcgttag
90181 cacatccgtt ttcttgctcg ttagcgcttg actaatagaa aggttagggg gcttttctcg
90241 cttgtttgag accatataag gctttctttc aatcggcaaa caaggggaag tctggtaggg
90301 ctttaagaga cagggacagg gacaggggca tgcctgactc tttttaaagt atacaagtgt
90361 tgaaagagtg aagtctgggg acaacggtaa acgtgaggaa tgggacctcc aaaccgcctt
90421 actaaagaag tttgcaagca agggacatgc cgaacaccta cctttctttc caactaagtc
90481 caacgcgagg aggtcaatct taagtgttgg aagctactgc taaggtactc cccctcatct
90541 atactagaaa ggataggcaa ttgagagata atagggcgat agcccggttt tggttgaata
90601 tcctttcctg tgcttgtctt gtattgaggt ataccgaaga tatgagtcaa agtaggtaac
90661 agaaggggat ggatgtctga gcggttgaaa gagtcggtct tgaaaaccga agtatttata
90721 ggaataccgg gggttcgaat ccctctccat ccgcgaggtc ataagttctc tcttgcgtta
90781 tctatagata agaacgaatc ggatcgactc gactgatatg atagatggaa tggtagcttg
90841 tgttatgatt tagttaggac tttgtctccc tttcgttatc ttccgctctc cttgtatagg
90901 ttggggaagg gccgggatca agcctatttt ttgtagttct gtcagttctg tctaagatac
90961 gagtggtaat gtgcaggcgg ctagcatctc aacgagtact cgagctaatg ctacggatca
91021 tctaagtcac tctttctcgt catcatagtt caaataagga tactttcttc atacactata
91081 gagcctttac ttaccgcttt tcaaattaca aatacttgga aacgttttca gttctttgat
91141 tcattctgta gctagaaatc cttagcttat agcgactctt acgctcttac gaggtaaaaa
91201 tctacgttac gttggaagag aaggggaact ggccacagaa tgggcgggga aaaacctatc
91261 gtgtggaata tgaattcatc ccctcgattt gctgcgcatg cggtcgaaag tctgatagct
91321 tcagctagta gtttaaaagc gatgttttga ctttgagttc ctctttaacc aaaatcggga
91381 gaaaaagcct agcgcgcaga aaagtagata gagtgctccg gcttcgattt ctcgtcaaaa
91441 gtaggccgat ggatctgtca aggtttcaaa gccaaatcct gcatttgaag agtggctctg
91501 caaagatcag ctgctggtca gttggataaa ctccacctcc ctttacatcc gcgctcaaat
91561 cctcaagccc aagaaattct gacaagatcg taaatctatt cgtctgttga cttcccatca
91621 gcagattatg gccaatccta tataggaagg ttgaattccg accggttccc cttggtttct
91681 ttcgggggcg aggaagatag aaagagggtt tcggcgaaga atgctttttt ctacggaaat
91741 tagaactctt acttttatcg tgcccctaat atatagttta ctttcgtaag gaacggtatc
91801 aaatacatga tttctgacga catatagatc atattggttg taagacctag gagacttccc
91861 aattaggcgc taaacattcg ttgaaaggcc agtattgtgg gcttgtagtt gaagtttttt
91921 attggtcggg aatcccagga agaaatgttt acagttgaga atgaagggag tgcaacagcg
91981 ggctatccta gccttttatc ttgctcgcga gtgggctatt tcttttcttt cccaactctc
92041 tcaaataaca tatctaccgg gggtcttact tacaggcata tgcctatgta tggtatctgt
92101 aaaccccctt ttgtggctca attgttggcc tttagttcga actataagga aaggttacgt
92161 agtatctcga ctgtcaagga aaccagcagc agctaactca gaaagcagga gaaaacagcc
92221 atgagttcca tcaataggag aagaagcagt gactcaatca cctctttctt tctatagatc
92281 gggttcagcc ctactatagt aggagtaatc aaggcaactc ttccctatct acctgttgga
92341 tacggttttc aaatccagct acttaagggt ttgataccga tgagttgttg cttattctcc
92401 gacaatctgc tctaggttac gaaaccattg tatgaagccc attctgatca tatggaaggg

92461 gttactttcc acctgaccta ttacctatta ctcgagtacc atcgtactag ggcaatcaat
92521 tagacgacat ctcccaaact ccatgcttca gagaaaaggc attccatgtg ctgttgcttg
92581 tttacatgct tagtctttta cgctacggat catctaatag ttgaaagaag ccatcatcac
92641 tccagaaggt caaaagtgag aatgttggag gggagatgcc atgatcctag gtctagattg
92701 gcttagaata gggcataata gtctaatgag aaaaaaagaa ggcataagac tctaatgagt
92761 agattcttat caccttatac aacttctttt gatagcccaa ataagctata taggcacatc
92821 tgggcttaca gatatcatgt gtcatggata ggttgaaacg tgggatatca gccccttttc
92881 aaatcagcca ttggaacgaa attagctaac atcaagctac ctctctctcc ggccaagccg
92941 taccttaggg gattccctgg caattcgttt aggccagtta ttagggtttt tctttttaca
93001 agtcaagata ctacgtaacc taagccccat tgccaggaac tgccataacc ggacaagaga
93061 aagaaagcaa gcaaacaacc tttcgacaaa gctgtaaccc cagggttctg aaatagctca
93121 accattggga tgtaattgct cgatcggaag tgatgaggtt ctgaaagaaa gagtggaagg
93181 gcgtgtagct acggaatagg tatcgtaagg actgattccc aggctgcttt agctactgtt
93241 cgcccagaaa cccgaataag aacaagagca ggaactcagc ctcaaactcc aattccatgg
93301 actggtggat ctcatactcg aatgattgat tggccagata gacccttcct tccaagagaa
93361 agaagaagaa catatctcta ggccggctgc ttaactccta agcaaggccg gtttccctta
93421 ctgtacgagt aggattgatg acgtggacca ctttcgaaag tgggagattc ggttttgaac
93481 aactcttttt cggctccaat cctcccctta tgcgcaggag taggattcaa tggtgaaaga
93541 accttgtttt ataggctttt gttcaactcc gatctttcaa ctcggaatag tatcaacaag
93601 aacaggaaca agagcttggg taagcattca aagcaaaagc agtctccttt cacgaccact
93661 ttccagtaga gggagaaagt cctctcatta ttcccggaag tcagagcaat tgcctttctt
93721 ctttatcgca ttgagccgag tgtgggatcg atccctttcg agctaactta actgccaaca
93781 agagaaagaa gaactagcga ctctccgttc aagctttcaa agagcagatt ctcggcttgg
93841 ccactcacta aggtcttggc ttagccattc attctattaa ggtaagcttt cccatctcag
93901 aaggaaagtc tttattcaaa ccatgctacc actaaggatg gcagtcaata gctctaagat
93961 cacatcgggt tctaaccctc aggtgacttc actcgcaaca tcggaatctc tttccctgct
94021 atctcctctc tttctcaata agtatgagct ggggtagctt tagaacaatt cccaggatta
94081 gcaataggaa tcggaactgt tttcactgta gtcgataacc ttattagagg atgggaccct
94141 ctccttactc taacaagtaa gcgaataaac taccttcctt tagcgagtca acttcctttc
94201 ttcctccatt cgtttgtcgc cttccactgc atgtatcgct tgaaaggcca aaatacggta
94261 aggaaaacct gactttggta cctttcgtgg ggattggtca tctgatgtct tcctcgagct
94321 atttctctct atagaaggtg ttcttttaac cagttttcct ctatataaga ggtgttctcc
94381 gaaccgcttt cttctataat aaggaagaac agagctactc ttgatcagat gcttattcca
94441 ggcaagttta tcagtcaagc aagtagggaa cagatcctca ttgcctttgc ctttatattc
94501 ataggtgtgc tatatgatgt ccttactctg gtttgtttgg ttatacggca gttgaaccct
94561 gctttcagca ttagtcttct ctatccccgt tctgggaact tttatttagg taactaggaa
94621 cgataccta tccctacgac aagaacacgt gctaacgaga ccctctcttc acaagactgc
94681 agtgaacagg tgtcccacgg agcaataaag aagctaacct tccccggggg ttaaccttct
94741 ttatagaagt tctctctagg cccaatagga gaggttctgg ttgggcttcc ctgggagaga
94801 tttacatcat agtaggttca ttctgattct agttcccagt aggtctttcg aggaggtgtc
94861 cactccatct ccctaagcaa gcattcgttg tctctcttct ctgtgctatc tttcattctg
94921 gttcgggtac gcatacagaa taggtccctt ggagttgagg gggaagaaca ggctaccccg
94981 tcatatgcac cttacttcag ggagtttatc agtctctcta ttctatttct ttctctacgg
95041 aagctggagt tgaaccagcg ctctaagagc attacttact cttcagagtt gaccccattc

95101 cgttctggct acttttattt aggtaacaaa taaagaactt ggaacacgtg ctaacgaacg
95161 aaaccttatt acaagaactg ggaacacatg ctaatgagaa gaccctctct tcacaagaga
95221 acgataaggc taggatggtg aataagtccc acggagctaa cctcaccaaa gatttgacct
95281 tattcatagg taaggaaaga attttaataa ttcacttcct tttttcggtc gttctcttct
95341 gtcttctttc ttttgacttg gttggcaggg tcagggcctt tctcgctggg cgagcgcatc
95401 cgattctaaa gtcctttcct aaaccacttc ccgttcagtt gctgaaagat agagataagg
95461 ctttctaaat gagattgagt tccacgaata tgcaggctag aaagatgcta tttgctgcta
95521 ttctatctat ttgtgcatca agttcgaaga agatctcaat ctataatgaa gaaatgatag
95581 tagctcgttg ttttataggc tttatcatat tcagtcggaa gagtttaggt aagactttcc
95641 aagtgactct cgacgggaga atccaggcta ttcaggaaga attgcagcaa ttccccaatc
95701 ctaacgaagt agttcctccg gaatccaatg aacaacaacg attacttagg agcagcttgc
95761 gaatttgtgg caccgtagta gaatcattac caatggcacg ctgtgcgcct aagtgtgaaa
95821 agacagtgca agctttgtta tgccgaaacc taaatgttaa gtcagcaaca cttccaaatg
95881 ccacttcttc ccgtcgcatc cgtcttcagg acgatctagt cacaggtttt cacttctcag
95941 tgagtgaaag atttgtcccc gggtctacgt tgaaagcttc tatagtagaa ctcattcgag
96001 agggcttggt ggtcttaaga atggttcggg tggggggttt ctcttaagaa taaagaagac
96061 gaatagaatc taattcatgt ttatgctaac agaagagcgg atccaatact aagacgactt
96121 ctttctcagg aagtgcagca agtacttgag gaattttggg atttcatgcc ccacgagtga
96181 gttgccaagt gcccccttgt ctaaaagttt tctaccgcat gcgtcgtgtc ctacttttgc
96241 tttaactact acaagtctca atttagtgca tgatatagcg cccttgtgtt aatagtgctt
96301 cgttttattg ctgctttatt gaagtcaatt gatcctgtta tcgtccgcta caattgtttc
96361 cgatattcgg tcagcaagtg ttgatcctag cccgtcagca taccttttct gatcctttct
96421 gggaccattc gtatctatta tgagacgagc gttaagtgag gtagacgtgc agaggttggt
96481 cattcagaag cgtacttatg tgatgaacta accctttgtc gacttcgaaa ggtgatccat
96541 atatcatagg cctggcaatg gatggtgctt ctctgcccct cccttttctc ttgctttagg
96601 tgaattaccg gtgcttgatg caataaccgg tgtgccagta ggaactcttg gtcttatatc
96661 atatcagttg ccgcggttgt cggactagaa attgaataac taaccactgc ttgagtttcc
96721 ggtgttcttg ttgagttcat agccgctgtt agagtgatcg tagcagctgt gatagaatca
96781 gttgtcaaag gatcatttcg ctggaatgct tgcttgaata tagctactcc catgaaaagg
96841 aatttatcac tctatacggt atggctacaa accgaatgaa gaagtcggaa gcagttcatc
96901 gcctgtggga gctttctgat agtgaggaag ccctaatcta tctatcggat tgcctatttg
96961 tctatctgtc tggtgaagag agaatggaag agactctcaa tagggcaagc acccaatacc
97021 tcgttgagtc caaagctcca gctcaagcag ttcagtccaa aaaagagatg tctgccttcg
97081 ttctcgctca agctaaagct aagtctgcta gctcaggtcg caagtcaagt agtggagatc
97141 cagtgacaga cagaaaggag agttagactt gaatgcaaga atgaatctca aaaaggtctg
97201 ctcccgctgt taggattgct actttgtagg tttccaataa aaggtatacg taaaaatagg
97261 atttggttta cccctatgta ttctactcgt atcgtagcat gagaccctct cggaagtagt
97321 tgaagatcta aatatgaaat cccgggctaa ttgattcttc aactctatcc gccaatgggg
97381 agatccgatg acggactcca tggatcgagc cttttccttt ctcctactat taaagtgggg
97441 tggtcggcat cagcgatctc atcgttcgag tccccggacg aaggtgagac caaccttatt
97501 atgattagca aagtcctttc caactccgtc gaatgctttc actcctcttt tccttcttct
97561 gtcagttcct tgtcagtcag tcgactcccg tccctcaaaa gattttcgaa cttcacgctt
97621 attcatcgat atatgggtaa tccccggcgg tcagagaaga aaagatctct atgaaagcag
97681 gaggtgttaa acattagtct gtcttcaaat gcgggaaggt tagacattca gttcgatgag

97741 ggcaggttcc tacacattca tttgaatttc ttgattggga aagctggttc cgtagcatta
97801 gaagctgaag tatccggaaa gaaggaggga atgagggtgt agaaaagaag agaaggtttt
97861 agaccttata ttcgctgttt aaataaagca gcggctaggt ctctatcccc ggattcactg
97921 attcaaggct tttagatggg atggacagaa gagtttgaag cagtacgtaa gagtttgaac
97981 gggcgagtga ctttacttta gggttccact ggttccaaat ccaaaggcta aaagccggag
98041 tagttgatcc agtagttcta aatatagata gaagactccc gaatgaacaa ccatccacaa
98101 atgtcgattc attcaagcaa gagagtgcaa caatcctttc ctttgacaca ggtcccgtaa
98161 acgagtgaaa aatgccttgt acagtttccc tttcagccag tccaagagtt cagttcactc
98221 gaaggcgggt agccattaag gaaagcggct gttggatatt tggtttgaac cggttccgcc
98281 ccacccctta tagtaagtac tggtgagagg gaaagagcgc tcctgccctt tacccaaaca
98341 gtacaagaat ggcaatccac ctattggtgt gctgctgccc tcaaccgacc tgatcgtccc
98401 aatccggttg tcctcactaa gaaaagatcc aaaatctctt atctaatgaa tatgaatttg
98461 ctatctaatc ttcctttgcc tagctgccct agaacttcca gcgctaaggt ggttggtgtg
98521 gtaaggcaag caactcctct ttccgaagaa gagtgcccgc ccgagctatc ttaaagtaaa
98581 gctctatagg ttagctatag gtaaagttat cccagagccg gagacagggg ccgctactcc
98641 ttaataaacc gggaatccgc tagtaaggat aggacactga ataaggacct aaagtagttc
98701 gtcggatgag accgagaacc gaaagatgtc tatcaaagag cgagagggac tgactagacc
98761 gctatcaacc agctctgaag gttgcaaagc ctttagagtg ccccttagg aagggagttt
98821 actcacttag tgcttgcatt atggtagtaa aactcgctta gagcttatgg cacagctttt
98881 atataaaaca agccattcgg tcaggcttta gctttatagc aattggtgtt aaaaaaaagt
98941 actaagtcat ttttgttagg gaacttcaat caaaccatat ccatccgcca cattgagctt
99001 caattctccc acgcctgtct ccaatgctac acgttccgct gacttggacc tcttcttagg
99061 ttgatcgtca gacggccttg ggtcagtaac ctttgaggat gccatggctc caagcacaac
99121 ctcgctctga tacctgttgt cacggacttc tagcttgcct agctaacttg cccacgcggc
99181 acttgtgttg tgcaacggac ctctcttgag tagaaccctc aagcctatct aagacagaag
99241 agaaggaagt tagacgacat tagcaaactg agtattggtt ttcagctatt ggttaattag
99301 tgtctattag ggcttagttg ataagcggct gtcagtgcag gtgaaccgaa ctcgtatggc
99361 tagtatgtat tgtttccgtc agtagtctgc gtgcgagata gagtactgtc aggggttcgc
99421 gaagtctatt agttgcttgc tttcattttg agtctttccc gcatgcgtga ctaccattct
99481 ctcttttcca tattggtagc tccccccgat attagttctc gcggcgaagg atcgtcctct
99541 aactaccagg ggtttacttg tctcttttc ctttgctgga ggtcttacta gccttccctc
99601 ccgactaaga ttttgagtga cctgtctatc agggaaattc cccagtattg ctagtgttct
99661 atctttcttt atctagtttt atggctggtc ttccccgcaa gcagttaatt tgctcgcccg
99721 aagccttagc atggttctta gtaggattga cccaccctta gcagggtacc aaagaacatg
99781 agtattcgca agtctttggc agctagggtt gcaattggct agtagggttt agtatctaag
99841 cggctggcta atgttgggaa tccacccatc ttgtcagtag agtaaatcgc tcttcttcct
99901 tttccttagc cgaagggaag cactaagaca gagtctgcag ctggtagagg aggacggcgc
99961 cctagcgaaa gtcgaattat cttataagaa aagaaccatt agttggtcgc tggtcttatg
100021 gtaatggtga ctatctgctt cgtttccaat gcgtagtagt tctcaagtgt aggaggggct
100081 tttagctttg gaaagagtac gcgtgcccgt ataggtggcg tgccagtata gttgtctttc
100141 tggtaatagt tttagttgca gttatgctcc gtggaaatgg ttttcctttg agttgatggt
100201 atgggtattg gaagtgttaa tagtttggtg attgtattgc tagatctatt cttcctatac
100261 ctaattatct agtcatagat gtattgttgc gaatgcgcct cagtctccag tgttttagga
100321 agtgagcgaa gttcccaatg tgagaggaat aattgctttc gtcctaaggc ttcatgagag

100381 ccttaaaact ggggtttggt tcatgactga tatatctata tagtttttaa aagaatctat
100441 attttgtata aaaaaagggt acgctctcta gaagatttgc tcggagctgt tcactttcac
100501 ttggtcgcct ggtctcttga aacctctttg cttgcggggg caagagtgga aacgtatgag
100561 atagcgcttc gcgaaagaat cgtgtggcgc ggtgaaaggt ttcccgttgg ggtttccggc
100621 cctccaggtc tccacctact tgtggaagag ggaggtgagt taagtatcaa ctctctctct
100681 cgcgcctttc ttcagcttgt tcctgttcgt ctattcggga cggggcaggc accccacata
100741 catgaagaga agaagagagg gggattcctt gatattaagg tgtcaaggcg gtcgaagaag
100801 gccacaagtg gaagcaaccg atgctttggt cattcagttg actccttgct gccagtccgt
100861 gcttgctgtc atactggcaa aagcaggggt ttcggtcggt tttacctact attggatttg
100921 aaccaatgac tctcgccgta tgaaagcgat actctaaccg ctgagtcaag taggtcaaga
100981 tgagtaaagt cagagaaaat agaccсctat aagagaaagc cacgcaacca aagttcccct
101041 tactatacaa gggggggcgc ggagcgctaa gaaagagaaa gcgttatcac tatacgaaat
101101 gaagcaccgg ctaccccttt cgagctcagc tgaccccttc ttgattccgt ttttccccta
101161 tttgagatag atgaatcaac ggaactttga tccccggttt cctgcttggt caattcctgg
101221 aaggccccta ttaatgtaat gattgaacaa tcaaagtgcg tttgccgcga ctacgagctg
101281 ccagtgcgcc tttctttgag tcgcctacgc tcggggtcga gaactggaaa aaaagtagaa
101341 gggggtccaa aacgagctaa cgcgagtttt cgaacgacgc ttttcccgtt tttgaacatc
101401 actgagatag gcttttcccc gaaccattga cccttgttcg ggagggattc attcaatgga
101461 gctttatttg tttgatctag taaggggggt gttagaaata agccaccgcc cgacgaaaac
101521 aacagagcga cccgggacat cgagcgaaaa gctccaatgc gcgtattcgg agctacgcgg
101581 atgccgtagt cgcagaagcc ggatcaacat catgacaacg gcattctgga aaccgttggg
101641 ccagccacaa ttggtctaat gtctgggtgc gtatgggcgg tacactgaga gcacctttca
101701 tttcaagtcg gctgaaaaag aaagaaaaag ggttcgtcgt ctttttcccg ctttcacctt
101761 cgattgcgaa gggatttgag gccggttttc aattcgcttc tctctctccg gcgaggtttg
101821 aacttcgcgt ggtgggaagg ccctcacgat tcgcatcttc ccgtccagca aaaaaaaggt
101881 gaaggggagc ggacagcgag ttggaggacg ctgcagaacc gcgtgattga tccatttccc
101941 taattgaata aagttggaaa gccttcagag cctcagcccc taccattaga ttcaaaaaaa
102001 tgaaagctga ctagcaatcg ctcgtttttc ttattagcgg atgttaataa tgaaagacat
102061 aacatatatt agaaggcaat ccccggggaa ttcctatcga tttccgatgg ataacaatcc
102121 ctctttctcg ctttcgctct ttctcccaat cagccgcggg ggttccgggc atgagaacgc
102181 aagtgccgga atcaaacaaa aggtccgaac gtccgaggac gaaagaaaaa atgctccgcg
102241 gggaactcgt ttcatgtcgg cgtattcgtg ccggttagac gtcggccatg aaatccatca
102301 ctataccgag cagatcacgg gcattcacat ctcggtcaaa gggaactgtg cgcgattctc
102361 tcgtgaatcg ccttcagcaa ggtatggcac tcagggtctg aaccccggga taaatctttc
102421 ttcatagata caagacattc gttgctgatc taaaaaagat cttctcccgt gggcgttagc
102481 ggacgttttt cattcttcac ccggtcctta gtagcgttga caaaggtaac cgggggttta
102541 ctttggaaac gcgctaaaca ggcctatagg ttcgcctttc taatagaaga agtctaatta
102601 gatataagta tatagaatta gccagatcgt ctgaagcatg aacagaatag cctttgttcc
102661 gaaggcctag cgagttaagc gagttcctta ttttgttttc aaaggcggtc cttttctttc
102721 cccggaccga tgactccctt gttcagtact gctaactatt ataggaggat gtcgtcccct
102781 cgggactacc agtcagtagc ttcggttgtt gaatctcgtg caagttaggt tgtggttgta
102841 ttggctgcaa gcggaacgta ggcgctttag gtgtgtgttg accatgcact ctcgcgccgt
102901 gtaatgtcgt atgtatgata gaggtggtgc ggtgattgac ctcaatgcta atggttatcc
102961 ccaaggttca acgaatggag ctatgatcgt acccagatag agatgatggt cttcttctga

103021 tgtctccaag ccggccataa tagaatagat aggcgaagca gccaatagca gtctgttctc
103081 gcctatcgat agatagcagg ttgcttccaa gctcaaatca tttgaaacgg aattgctact
103141 tttttattgt tattgataga gtggtattct ccgcccctgt aaaagaaaag aaagtagagg
103201 gggagaactg gaagagagaa ggaaaaagag caaaggattt acaagtctaa tgggagaaga
103261 atggctgaca cgttgactgc cttcgatact ctaaaataga agccaagcgg cctaaccccc
103321 ggggccgggc cccaaccgaa agacgctaga cggactaacg gcaagcgaga taaagaaagc
103381 agctggccct atgtgtaaaa ccttgccgcg ggagagtctt tctccaatga gaataaagaa
103441 agtttttggg caagacaaga aaggaactcg cccttctcca ccaagggaga agagctgatt
103501 gctggcgatg acttggtgaa gggaatctat gagagagaag gttctcgaac cgggttccga
103561 ccgaatagag cgcggagcca acgagttcag tcgaacaacg taacgagtct aagggcggga
103621 tcaagctaaa aagaaaagga atggacgggc gtaggcttaa tagttaaggc agaccccccg
103681 ctgatagata tgagatcaat gacttaaaag aaaagacaga tgtcgagaga aactgttaga
103741 cttctttatt gcgttgcgaa gagagatcga agacgaagat tttatgacgc agtgcgggca
103801 ctggatggaa aagaaagaga agggaacaac ctaccggaag ggcatacctc gaggagcacc
103861 aatctccccc ggcaaacatc tatctgcacg aagccgacct acggatagaa agaaagatgc
103921 aggaaaggaa aatgaaatag aaaagagaaa tatgctttgg gagtgtgaga taggcggacg
103981 gggagtacgc agccgaacaa ccgcaggggc ttccagcagt gatagctgaa ctaaccagat
104041 gggccgccct ggaacttaca ttgaaatcgg aaatcaacgt cggatcccgg ctatccgaaa
104101 aacgcagact gaagcggagg atcacaaaat gcaacacaac aaggggcgaa ccaaacgctt
104161 gcgcgaagga agaagctaat caatcagaat ggagacaggg aggagaggcg aaagagagat
104221 tttcgagcct gcaagcagca agcaacaacg gctaaaaagc cgttgcgcgc tagcttgtag
104281 tttaggggta tagtaggggt gccccttcg aaaactgttc tctaatataa gtataaggaa
104341 gggaagcttt ttggaaccct agttttcccc aacctatacc ttactaatca aaaggggctt
104401 acgtttttca gctgcattgc actgccgcat aaacaatgga tccgctgggc tggatgagca
104461 accttctctg gaaaggaata gtgaaaagcc atgtcacttg gaactggttg cttacttact
104521 tatagtaaga ccactttttt ggtcagaaaa atgattatta gataggcatg gttgtttaca
104581 agacaagagc tagcttctag atgttcttcg cctgaacata catacatata gaagaagcaa
104641 ccttctatct ggcctctgta ccagtacagt agtggagtgg cttgctactt tcaatcagaa
104701 aaggaagatt gagcaaggca aaggagaaag aagttgtccc ctcttctctg ggaacccgcc
104761 gccgcatatg tagaaaaaga gggagcgggg aaggaagaac aaccctttga ctttggcact
104821 tgaggtggcg ggtttggcta ggtaacataa tggaaatgta tcggactgca aatcctggaa
104881 tgacggttcg accccgtcct tggcctcgag gagtggtgag cagcacggaa cttgaatcaa
104941 tcaaatggta gggctttcct ttgttagaaa tccacagaaa acattctgga acttccaaac
105001 caaagaaatg caacatcaac atgagaagtc gctttttacg ttacgctttc attttaatag
105061 aatgaaatga atttggtaag ggtagaatac gccctttggt cgatcgaagg tcctgtgcca
105121 tttgaactca aatagaaaat ccatctttgt tccagccagc tcataacaaa atgttaggcc
105181 ggctgacaca accgaattgg ttgaggaaaa ggaaacccgc ccccaaaagc ggagaccgaa
105241 gaaccgccag gttgtcgagg acacgtctga agaagaggcg ggcgccctct aagtttacca
105301 ccctacccac taatggacta tgtatgaggg gggcaggcga gcgggaaccg accgaaaacc
105361 cgaaccgctt gactgccccc tgaatactcg attcattagg gttgggggat ggatggaacc
105421 aatcagaagt gcaaatcgcg caagcgaagc cgggaaacgg accgcagcgc aacgactagg
105481 actcgtgtcg gaggagtttt gcgcgtgagc taccactcat gcagcggcaa gggcccgcaa
105541 ctctcgaagg ggccaccaac cgcccgaatc actgtagcaa acccccettt actcaatgga
105601 tagcgcttat tctctttcaa tcaacaaaaa gagaaatgag ggagaaagaa aagaaagagg

105661 tctttattga agaggctttg cacctgaaat aggactaatg cagatccaga agactgggtc
105721 tgggctttcg aaaagatgaa gatgcaaagg gtaaagagaa agtcttttga acaaccaacc
105781 tggcctaagg attctagctc gcccacttaa agcagatgga gattctcgga cggggaaaag
105841 tggcactcgg catctatcta ttaaacaaca ccaagaacaa agccatacca tgccctcggg
105901 ataaactagt gatgattgcc atgaatgctg ccctcgagga cgtgtacaag aaggtctttg
105961 ccgattccta tcaacatggt gcacctctta gtagaggggc ccgtggagac tttgaccgaa
106021 tgaataggga gaaattgata gacattttga ttgaggaaga taatccagga tgaacgcttt
106081 tttcattcgc tatgcgctga ctggatgcgt actcgcgcga tcaatcgatg gacgggggaa
106141 ttgcgtgaat gacgagaccg gcctaaccta aagtaaaggc tctttcttcc ctctcttgat
106201 ggcgaatctt gattgactga cactaggaac cgattcggag aaagaagaag catggcatga
106261 gataggcggc ggaaaaaatgt ccgatagcga attacttgac tcgatggatg gagggagagc
106321 cctccaactc aagcacgaaa tcaatgttga gtcaacaatc aatcatcgag agaggcacca
106381 tcaagcgaga tcgagaccag cccatagggt tccaaacccg cgcttcttca aatatcccat
106441 aacataaaag taggggcttc aaagctttac taacaagcga aaaacttgac tttgagaaag
106501 aaaaggggcg cgctaacgag caagaaaggc cccttactat agatagggcg ttagtgcttg
106561 actaataaca tatatagttt ccgcttgatc ggaatcgatt ctatgattga atagcagaag
106621 tgctacttag tagtataaac tcggagagac agacagacag agaggggact ctttcatacc
106681 tgctaactcc taggatgaga agtaggtccc ttgtttttgg caggaatagt tccagccttt
106741 gttgcccctc ggtagagtga gtctacttag cgaactggca ggacgcggag atcgattgat
106801 caatatgaat ctcgagagtg gatggttgat cgccgcctcc ttgttctgga ggctctccgg
106861 ccggggaggg gcttgctatc gtaacccttt ctcccggtgt atgtgaaaaa gagtgacgaa
106921 ctcctttttg ttcggtcata ggttggtcca aagaacgaga gtcggcttcc ttaagtccgt
106981 tgttcctcag tagctcagtg gtagagcggt cggctgttaa ctgactggtc gtaggttcaa
107041 atcctacttg gggagaattt atagttctcg cttttctgac ctagcgaccc ctgtccttct
107101 ccttagtttc taaactagca gaatcgtgga acatcaaaag tgggaagttt cttgttggtt
107161 tttcttcctc actctcgtat aggtgaactt ggttcgttcc attcttaggg agaagaagga
107221 tccactggga atgaatggga agactggagt agtatcttca ttagccggaa ggaattagtc
107281 tccactagcc tcattcgaaa aacgaacaag aaaagggtga ctttttcttt taggtaggat
107341 agatggatgt ggtccaatgg ctaaagctct gccagcttat tgtagactga cttctcttta
107401 ggctccgagt ttttttgtt ttgggtggga tggtaaaatg gatcttcgcc actcactagt
107461 ggcaacgaag ttcttggtaa ttagcaaggg ggaaggaaga tccccactca tttcattcat
107521 tcagtgcctg cggtgaggtg cgacccacaa caaacgaagg gggaaagctt gctttgcttg
107581 ctgtagccct atttgagtac ctaaggcttg gtaagcgtaa gctatttaag taggctcgag
107641 aagggtaggc tcgcagcttc gctcagcaga agagccttac tttcctattc tattagtcaa
107701 gcgctagcgc ccaacctata caagggcttt gaagggatag gggaagggaa gaaaacaaag
107761 atggtcactc cttttaggaa catggctcgt tcattcactt gctcatgaac tcgcagcttc
107821 gccagaagcg actagtcgcc tcctttcctc cgccgaaaga tgcttagcca gaagcgacga
107881 aggggggccg gggaaggctg acgactacat gaggggaagc tatcatagaa gccttgcccc
107941 ttgctttaca gatattgtta tgaactgact aaatgactag attctcccgg aacgctagct
108001 aagctaatag tcttagttcc cttcaatgaa atcaataagc ttgttgattg attcagagcg
108061 ccgccctcct tcttagaacc cattgagggg ggcctcggcc cgggaagggg agagtggccg
108121 agtggtcaaa agcgacagac tgtaaatctg ttgaagtttt tctacgtagg ttcgaatcct
108181 gcctctccca cttgtttgtt gtagacttca gagaagagaa aagaggcatt cgtcagcgta
108241 ggaaggccaa ccgagcgaag ctctttcttt cttttttgc cgtgtcgtga agtgaaattg

108301 tatcgtatgt tagttagaga ggttggcgaa ctactacgat ctatagattc cccatctata
108361 tccaatccca acgagaatag aagcgagtcg cttccggctt ggcttgtagt cgtagtcttt
108421 tagcttatgg agtcgtcggc cttcctacac gcacgcgccc gcaagaatgc ctccttggtt
108481 cgggacgaag ccaagccgat acatacgata ggcgaaggaa agacccccca tttaatagcg
108541 cctggggaac gcaagtttga tcgaggattg gagaggagag gtggaataaa agaaaagaaa
108601 aaaaggctcg ggatggattt aggagtcttt gtgcgagccg tatgcggtga gagtcgcacg
108661 tacggtaacg aggggggttc gcgtctatac gtgtagtgtg gtggttgggc ctacccaccc
108721 tatttgttcc atgatctatg ggtctactgg agctacccac tttgatcaat tagccaagat
108781 tttgaccgga tacgaaatca ctggtgctcg atctagtggt atttttatgg ggattctatc
108841 tatcgctgta ggatccctat tcaagatcac tgcagttcct tttcgggcgg ctgtaggaca
108901 gacggccacc tataggtggt agggtagggt gggtggtacc gctcagattg cggccaatct
108961 tcctaaccgc gcgcgggccg cgcttagagc gcgtgaaact aatcactacc tcgtaagggc
109021 gttgagacca tagcatgtta cacgaaagcg ccgctttctc tgtagtgttg ccacacagct
109081 gcccgcctag aagagccact cgctctgtag tgttgtcaca caagataagc acgccgcccg
109141 cctgctggcc gggcgaatcg aagttatctt ccggtcaact gtccacccag tcaagtgcaa
109201 aaacacagta gaatcacgca acgcacgcta ctggtgtgct tcctgctcgc gaggaaagaa
109261 agaagagcga caaagtgaag ttcagatttg actgtttgca gcatgggagc agattaccca
109321 aaaaatacct gggaacaatg aaaaaaaaga tatctcggta acgaaaacta tagggggctg
109381 tattggcgag atccaacggt gaacagctgc ccaaaagaaa aaccgcctgg aagtccgagg
109441 acctttagta ctgtactcta cccccgaacc agcagccttc gcgccaagca agaccgccct
109501 tgtccctttc ctttctccat tccgcctcct tctttgcttt gttccaatag agtctaaggc
109561 aaagcaaaag tggttcgtat gcctacttta cctacttgac gaaagggaac gagctttgtt
109621 tcgtttccgg gtttatggat tggattcagt cagcgtcacg acataatcaa aaggaaggag
109681 tgacgctttg gttaccggcg aacgcttcca aaggcgaccc tctcgagttt ccggctgttt
109741 tctagattga agtagccttt cgtcgcccta ccaaacgaaa taagtcacta tcaaacagct
109801 cgccctactg aagtaccaaa ggtgcgctca gcccggttac taagaaatgg gtttgcactt
109861 gaatttaagt gatgaggtcg caaagaggga aatagggctc ttatttgact aaaagttggt
109921 tcttcgcttt cctttagaat gaaagtcgct atgaagcccc tactacaacc tttgtttgat
109981 taaaaaggcg aacagccccc caactagtcg tatggggcgg ggtgcttgtg ataagctgcc
110041 ttggatatga ggaattctta aaaaaaggca aagtccggta ttttactaag atctttctat
110101 caatagatag gaatgagcgt tcgatatagg ggataggatc catctgctct ttatgaacgc
110161 ggagcccgtt ccgaatgctt cttcgatccg gaagttctcg cgaagagaat aagatggtgc
110221 tccccctccc tctgtctttt cccgctttgc gaatcttccc ctctaacgcg ggccgggcgg
110281 cggcgggcgc gggaggaaga aagctaagag aagacgctct tctcttaggt cctttttttt
110341 tcagtgtaac acaggaaagc gccctcttct ttttgtcatc cctgcagctt tcctttgtat
110401 tgaacgcatg gcgtagctag gacccttcaa atcatgtttg agcttatgtt caaaccaacc
110461 ccacccccta tttgagtaag gctggaaaga atgtccgcca agttcagata agggaatgct
110521 tcccccaacc attaaaggaa aggctcgacg aggggaggga gatgcggcgg gggaaggaaa
110581 aggctttcgg agatcgagat ttttttttt catcgaaaac gaagaaggcc gaggatggcc
110641 tacggtgcgt cttatctgaa gggaacgcgc tttttcgacc gcggtggtat gattgtcggg
110701 ctctctcctc gttccgcccg ctggcctatt gggatagcag ccttcgggct ttacctgccc
110761 tttctaagag aataaagaat tccggctcgg cccgggaaag cgctggcaac aacagaaagg
110821 aaggggtcca tgtagctgct gcgcccgccc ccctcttagt caatggggca gcaggttcgg
110881 catctactac aaaagagaga atccacttca gataaccacg cctctgctag ggcagcgtgt

110941 ggaatggccg agagcggacc tttttggata tataatccaa gtcgagagta gagttacggg
111001 aacagccgtc tgatggaaaa ctactttcac gttcggttca gagagcactt ttttcgttga
111061 gaattcgtcg ttccctttcg tgtgaattcc ccagcggcga attaaaaact tgtgggccct
111121 atctattcca tctctcgagc cccgaagaaa accacccccc cctcggattc catatctctt
111181 ttgactctat atatgtgggc acctgatatc tatgagggtt cacccacccc ggttacagca
111241 ttcctttcta ttgcgcctaa aatatctatt tctgctaata tttcacgtgt ttctatttat
111301 ggttcttatg gagctacatt gcaacaaatc ttctttttct gcagcattgc ttctatgatc
111361 ttaggagcac tggccgccat ggcccaaacg aaagtaaaaa gacttctagc tcatagttca
111421 attggacatg taggttatat tcgtactggt ttctcatgcg gaaccataga aggaattcaa
111481 tcactactaa ttggtatctt tatttatgca tcaatgacga tagatgcatt cgccatagtt
111541 tcagcattac ggcaaacccg tgtcaaatat atagcggatt tgggcgctct agccaaaacg
111601 aatcctattt cggctattac cttctccatt actatgttct catacgcagg aataccccccg
111661 ttagccggct tttgtagcaa attctatttg ttcttcgccg ctttgggttg tggggcttac
111721 ttcctagccc cagtgggagt agtgactagc gttataggtc gttgggcggc cggaaggttg
111781 ccacgagtaa gtcagtttgg gggaccgaag gcagttctcc gtgcaccgga cacgtagctt
111841 accgaatcag ttgcgacacg gatgggaatg catgctacga aagatagggt cgagtctgat
111901 acatcaaccg tctactcaat atccttgtac gagttcacaa tcactacacg agatgaacct
111961 tggtttggtg aattgaagtt ggccttaggt gtaataggac tcccagttac tgcgcgcgat
112021 cgtatactga ggtgctcccc gccggttgtt ggaacgacgc gagccgggcc gggtctcgat
112081 tcagaaagat gaagggccaa aaagtctaaa tagggggtta caaattcccc atctcattgg
112141 gggcggaaaa cgaatcgaca tctcgatgtg atacagcctt ttccatttct gttgggaaag
112201 aacggcgaag tccatccgaa ccgtccaatg aagaataaga ggagagcaaa gcgccaatgg
112261 cgcgcgaagc gcatgcggaa cgggcacgga tcaaaaaagt ttggaggaga agcagccgag
112321 ctcattccct tcgcttcctg ggcccaaagc agtgcagtct ttcctggcca aatcaaggat
112381 ttggggcttc ttgctacgct acttaactat ataaatcctt ttttttagta atatatatga
112441 atagaaagat agatccatcc atctatccta tccgatttct atttttatat ctaaaaaaga
112501 atcgatttca ttcaacgttt gattcaaaga actgtgctta gcccccccgc tcatgaaacg
112561 gctctgctgc aatggatggc agagggtccg tagtacccga agcactggag tgatccagta
112621 gccgggaagg ggcctagaag tgcctactac tacaccacac tacacttggc tctacacatt
112681 tacagagcta acccctgtcc agtgcctggt agagctaagg gggcttcaat ccttactctt
112741 tatccccatc ttcgcccagg ctaacgggcc ttacttattc aggggggaga gtagcactgt
112801 tgagaggaag atccttggcc cctcttcatt ctctacaggg ttccaaatct ttcttcaaca
112861 taggtgacaa cgagcgaggc agagatggaa gagataaaag acgagaataa gaagcaagct
112921 cgccttcctt ttttttaga gggggatgga gaaagtggac aaaacagact cgcatttccc
112981 agtcgaaaag atgggttcct ttttcgtctc gcatttctca tcgaacaaat acaggaaaag
113041 aatcatattg aaaacgatcc taacccaacc ccttccttcg tagagccgtg tattgtaagt
113101 gatccgaacc tgcccggagc gagcccccca tagaggcaag tgaagttggt gagccgtatg
113161 atgggcaact atctcttgcg gttcggagag gactcagctg ttagttagta cccccttggt
113221 ttcggggtgg accctttcac tctattttat tatatacgct tagcgaaaag aatgtttttt
113281 gatacaccta ggacatggat tctatatgaa ccaatggatc gtgacaagtc gttactacta
113341 gcaatgactt cctctttcat tacttcattc tttccatatc cctctccttt gttctcagtt
113401 actcatcaaa tggcactcag ttcatatctt taagttcgat cattgacaag gttcaaagaa
113461 agggtgggaa cactatggtt tcgaatcagg gtcgtgtaaa tggagaatct gtgctctttg
113521 tattgcttta gccaccagtc ccaggggtta gagtcttcct tttgtttcct cctggaatgg

113581 ggatggaagt ctttcacact aatgggagtg ggtactctct ctctcgctgc cctgctagag
113641 ctccatggca tctactttta aaaatagggt ataaataacg tgaaacgggc tacatagtac
113701 tggaaatcgc tagaaataaa gtaagaactt aaaagtcaca tcatctgaag cagcagcctc
113761 gatttgagac ctaatttgat acgggattgg cgactggaat tgaacccata taaagccttt
113821 tacctgttgc cgatccgatt accgattcat aggattaacc agaagagaag gaagttagga
113881 ccggctgaaa gaaggacagc ggggaaagtt ctctaacctg ggaataaaaa ccttagaatc
113941 cgtctttttt ggacggaaag ggcaagtagt agttcagaga aaagtgacaa gaccctctcc
114001 cttatggtcg agctactccg ctccaataac gggaatttcg agcacttctt ccctctttcc
114061 gccctaggcc cactcactcc cgtcgacgag aagggaaagg aggaactgag gcgacaatcc
114121 cataaaggaa ggggtccgat ctgcatgtgt taagcaccgc gcatttcatt taagaaaaga
114181 gagaagctag cgtccgattc catgcttccc tatgctatca aatcgcggac gatgtcgatg
114241 catacggacg atctcttgcc gctgcaagtc ccacatccgc ggttggtgag agtaatgtga
114301 tagaggaaga cttttgttcg aaatcgggat agtgttcaat aaaccaccgt tgatgcaagg
114361 gaaggcattc ccgctgctac agttggagtt gacggtccta gttctgttac agtaagagct
114421 gttgctggaa taagcagtgc tagtgacttg actgttggag gaagaaccgc tgttagaaat
114481 gtgcacgtag aagctcctat ttcatctgct ggtatagacg aagctcttgg gatcttatcc
114541 gtttcggagg tttgaggagg ttaatcaaaa ggggaatcag ttggtattga accttcctct
114601 atcaatttct tgtttctttt ttggacatct tatattatat gcattttcaa ccaaacaagg
114661 atctgactca attcaataga aacttcactt ctaccctgtg tggtatattt tggagcttag
114721 tccactagaa agcgtatttt ttagctttca tcttagccgt gaaagtttga gttttcaaag
114781 agaaggagaa ctctttatta cctttctgct ttgcctttgc tttaatgcgc ccgattcctt
114841 accggacgat ttggcttttc tttcagaacc tctcttgcag ctaaaggaag aattgttttc
114901 gaataagctg tttgaaggaa tctctccata gtagaactac acccatgcac aaaagagtga
114961 atccttgaag agcaggtata ttaggcatag aattggacga cggagggagc tcttactgct
115021 cgagaaggag ctgtgagact tatgttctcg catccagggc agggactgat tagacgctct
115081 cctggcataa gtggtatcat attttcgaac taatggcact aatccggaaa tccacgcaag
115141 gaaagggcag tgcttaatat tattatatat acgatgaatg tgcagctagg agagccaaga
115201 ttctttactt gaagacttca gcgctaacgc catttgacag ctacctaggt ttttcttcca
115261 agcatgaact cctactagtt gattgaatca ctccccgatt ccgggaagaa agctaatttc
115321 gtaaagattc aaacaagaaa tcgacgaaag caagagaaat gaaaatatca aatagatagg
115381 gcgccagagc taacgttcct tgggaatagc aattatctgc ttacttaata gaaaagacag
115441 ggagacctat tctccatcca tcaccagaga gatttcatct tccaattcat aaatgatgaa
115501 ccacttatat aatatatcga atatatatat atatatatat attgcttgtc ctgaatgatg
115561 tagttgcttg gttttctttt tcaactgcca tcaatatgag gtatgtggtc aggtggaaag
115621 gatagttctt ttctttcttt tctgaatatt ggtgttcagt gtattcttcc cggaagaagt
115681 cattccaaac tttctgcccc tccggtggag cattccttcg caaacctctc cttgatggtc
115741 gagatagcta ttcgcaaacc taagacagaa ggtcatgttg gcatggtgga aggcaacggg
115801 ctttgaaaaa ccgaaatcga aagacgaaca gccacacccc acacttcaat tatggcgaaa
115861 gaaacctgtg caaaggataa gattggctga ccagcggaga agctgatgat ggagcggtca
115921 gggtaattgg aaccagaaag tcaggttttg gccctagtga gatgcgagag tgagagttgt
115981 tccagcttgt cccagcgcgt ataacaagta agcgagtaaa ctcccatgta taggatacta
116041 agcaaggtag tttacttgct ttcactctaa tagtactata gaagctacct atattctggt
116101 ttagttgcta tagtttacca ggttagttgc tacttgacta tattatatac aagactcttc
116161 tagtttattt gcacatctat aagccaagac cataagctag ttggctttct agttacatag

116221 cgtgtatggc tactccggtt gttgctagca agactctata caacatagca gtacttgcta
116281 attgattatg acgcttgcat aaagatagaa agaatttcgt tatataaaaa acatatgtaa
116341 tcctagtaga gtaacaatcc tgctttattc ttttcttgat cgtcctaaag tgacttatga
116401 gaacgaagac agagtgcgat catggagtgg tgcccctttc gaacacttag tctcaaaagg
116461 ctagattagt atcgaggaga aggccttaat tgctcggcta tactcgatca atacctccgc
116521 tagtagttct tggaagaact tcctggattc tcaagaagag atgagaagct gtagtggggc
116581 atctaactaa aagagtcctg ctatatagac taaggttgtt gctgttgaag cgaattacgc
116641 tgggagttct ttttcctctc cctatcggtc gttctttcag agggtcttta gcaccttaac
116701 tgcaagagct ccgagctgca atagcgactt tcgagatctc ggttcagatc tgtctgaaaa
116761 gtagcaagac gaaaactgct cctttagctg caggtggttg gcttacttag tgtcctacgc
116821 tgaggagtta actgctgcta ttgtagcttg tagctcggag ctggctaatg aggccccgtt
116881 aacaaagtta acaaacaaag tggatccttt cttcccttcc ttattgcaat agtgggcaaa
116941 tgctgtgatt gtgatctttt ctgctgtgat tgaatcagta agtgctgcag atatgatcgt
117001 ataaagaatt accctcattc gatctcagat gggatgagaa ggcagggagt agcactaatc
117061 tcagggacaa atgccacaga aggaatagaa gtagctgctc tcgaatgccc tcttgctgtg
117121 ctcgaagaag ctgttttcga cgctaactct aaatttcata agagagagaa agcaaagaag
117181 gaattgatag aggtgcgccg agaagtttac cttctttcat gatgtacttt catcgaaatt
117241 gggttggtgt tcagttagag caagacatta gctatccaag cttccacttt tcaaaagaaa
117301 ggaaccgagg ccctgaaaga agataatggg gtccgacttg tactattcat aggactcatt
117361 ctcctttcta gtgagcgcgt gccttatttt attggtcttt tttatctggt gccatttatg
117421 tttttcattt gaatttctct gacattctac gttcccgaaa cggatcctat caaatatttc
117481 acattttcta tgatcatctc tatttcaggt attcggggaa tcctccttaa tagacgaaat
117541 attcctatta tgtcaatgcc aatagaatca atgttattag ctgtgaattc gaactttttg
117601 gtattttccg tttcttcgga tgatatgatg ggtcaatcat ttgcttcatt ggttccaacg
117661 gtggcagccg cggaatctgc tattgggtta gccattttcg ttattacttt ccgagtccga
117721 gggactattg ctgtagaatc tattaatagc attcaaggtt aaacatgact cccagagagt
117781 taccaaaata cgaagtgatc ttctcctttc gttctcaaaa gaagagtttg atcctggctc
117841 agaaggaacg ctagctatat gcttaacaca aataaatcga tttgatattc agtaagtcag
117901 tcggtgaatt ctttcttttt gtaagaataa atcccggtag ctaagtggtt tagcgggcga
117961 cagttagagt tcaagtgaat ggtcgttctt gtgtgggttc gactcccata gccccgatgt
118021 gacgaaaaag actgattcaa cgagatatgc cttcattaag atttcaaact tgtcgtctac
118081 tttcaggaaa tgtttggaac agagaactta caataataca acgccacatt ctccaaagat
118141 tgaggaacaa gaagagatct attaagagaa agatttattc gagagaaaat cttaacagtt
118201 acatccaatc acaaactaca cgaaagttgc ccctttttta tggagattta cccatcacag
118261 agatgcacag aggaagagaa cgaacttcat atatcccttt tccactcaat ccagaaacaa
118321 gatcggacgt tcttccggtt cgtctccatt ttcgtgaaac tattcctcaa gcaaggcagc
118381 cgataagtca tcgaagggtt tgtgtgaata atggaatggt aagcattact cattttaaag
118441 tgtcccacgg tgatataata tcttttcaag aaaatgatgc gagagcccgc ggtgaagaaa
118501 taaggagatc cttctatatc gaaatcttag ttgaaaaaat aataggaaaa ttccgggatc
118561 acccggtaag aatgtggaga agaaccaaaa ctgaatggtt ccgcctactc aaaactcaga
118621 ggggatgccg cctactacta aaagaccggt tttgcaaca gttgcgttct tctatgcaag
118681 aagaatactt agaaagaaca aagaagtttg gatcaaaaaa agtatgctta ggcagttctt
118741 tcgctgagca caacagaatt aagaggaatt tgtatcattt caaatcccta ttcttatcga
118801 atagaaggaa cgagaaaaac cgaaatcttc ctacccgaac aagaagtcct atagtttaca

118861 actcttcttt ttatagtaat tcgacctatt gctccgcacc cccccatcag tttactatga
118921 agagaaaaag aaaaaggatc gaactactta ctcattattc ggaggtgaat catagaacac
118981 taaaagctgt ggtatcttat ggacctaaca taggtcacat ccctcacgac ataagattga
119041 aagatccaaa ccttcttctt cggagcggaa acggacgtgg ccaaaacata taaagatcgg
119101 cgtagtcgct catagggaca tatctatccg gatagaggat agtctagatc gattcataga
119161 tagatctctc tccatataga taggtatctc cgtggataga gataaagata gggatccatc
119221 tagatcttga ttcactattt atttccattt ttttgacgac aagtttgaaa tcttaatgca
119281 ggtaagtcga agacttctcg tcgaatatta aaggaaacat ctttttaaaa ttcttgcttg
119341 ggtcggagcg tagacgggcg agtagagccc aggaaacagg gaagctcgtc atagagggag
119401 cagttgacta aaaatagaag actgctgcct tgagagaggg cggcctctct cgggaaagat
119461 ggcccaattt ctcttctttc tttttcacct cgttagctca gcggtctctt ttttgaataa
119521 tagcatagca gccggtttga gatatttcgt tttctaattt cctgctattg cgttcctttc
119581 agagttccaa aacttaaaaa atggaagcat agacgcgacg tagccaactt gggatttcct
119641 gcgagatctt ttaaaccgcc gggactgaat ggaatccgtt tggaaggatc tgatattgaa
119701 aaagcgatcc aaatagttcc aaacctccat agcaagctaa ctcttgatga agaggtgatc
119761 aagactttct ttatcatgct ccgaacagca caagcatttg cttgctaatc tgataccttt
119821 cgtcttcatt ttctcgccct cccttaactt attttgaaat cccctcatac aaagtaaaat
119881 aagaggcttc cacgcaaagc aaaaatgctt cgatatcatt gaaaaagcag tttctatgtg
119941 catcaagcac atctattctt tattggaaat aactagtaaa ggagttggcc tggtcaataa
120001 gagggagtca gactttgaac tgcattttga aaaagaaagt ctctgatagc ccctacggtt
120061 tgctgcttcc aaacctggtt gttacgcact tggagtgaaa tctgttctat tctcaggcaa
120121 ccccgccaac acttaagggc tcaaaatggt tgattggaaa tggagctaga acagatggga
120181 ccagatctat caatcaaaag caacacaacc ctaatagaga aataagaaga acttctactt
120241 ctttttttta gactattcct cctcactgaa caccaactaa atatcgatga aaggtgtagt
120301 cgaagctact cgatcattca acaggacagc tggaatctac gctaggatcc aacctgcact
120361 actagctttc ttctcttaag atttttgaga ttttatttag ataaagtttg atttttctca
120421 aagagagtct cagtctagtc accccggctt cctacccaat cctacaataa tcctgctcaa
120481 tcgccaattc taaatcatca agttatgttc taacctaaat cctaagctca accatctccg
120541 gtaactaatc ctacccagac cgtagcactt cagaccacta aacctcaggc tcggggttct
120601 atcgaggatg actgcgtccg acttgaagag gatgattaga gacctcatga aagagcctac
120661 gcctctaaca acgtatttta gggggttagg gcggttcctt aggcaccttc acatctacat
120721 ctacttataa caagcacgta cacacaagca agctttaatc ctgttatctt aactgagaat
120781 gccgttactt atagcctttt aacggcagct acacattgac cgagaatgaa tcttttagag
120841 gttttggttc catgattaga gttcaggcca ttcccgagag agggttattg ctcttctcaa
120901 tggataggca agtgccaaca agcggaggtt ccgggcgaaa ggggcatatt taggtcgaaa
120961 ttgtaggacg aattgtctca tttctgatcc caactactaa gggagtccgt cgaggccaaa
121021 gataggctat tccagcaacc ctttcttcgt gggacgctct ctgttccata tcaaagaccg
121081 ggagtcctag aaccgatcgt gtacttaaca aaggctgtgc ttggcgcacc acctggatgg
121141 cccgactggc gaatcagatt gatagctttt tgaatggggc gaggctaacg gctccgatag
121201 aaagggtcta tctgctccag agaaaagaga tgtatggtat gcctcacccg gtcaatgatg
121261 aaagaaattc taaaaaaaga gagtcacaca tgggaatacc ctatctgaag ggttctcggt
121321 aagaccatac agaagtagcc aaaccatagt tccatgggtt gatcagtcat tagggaaacc
121381 ttcctctgcc ttgaggaatg ggaccgaaat tgcatcatat aagaaagagt agtagccgaa
121441 ccaactgaca tagctagatc atcatcaaaa gaaggggctt cctttcgctc tcaagtgcta

121501 gtctcaaatc aaggagcctt caacggcgaa tcgcgtgcgg aaattgaatt gtttcatttt
121561 cagcatcttg tgttggtctt cagtcagcta tactaaatgc attggatgcc tgagcaactg
121621 catcgatccc attcctactt ctcttgcgtc cctccatccc agatctctag gaacagggct
121681 gacagcaaca ccactaattg aacgagtcgg gatgagaatc tcacatggtg ccgttcctgc
121741 gttctctctg tccaactcgt tacgcaattg acgtagtaga cctctcgtat gattgtttct
121801 ctgtctcgaa ggcaggttaa gtcaagtgta aaatcagggt agtgcattaa gaagctgcaa
121861 ctatcgcatc agctagagcg gcattaggca ttggaaaagt gcttcattcc gatagcgatt
121921 ttaggctttc agagcgggag aggaaaagaa agcatttaca gatagtcgct tttccttgct
121981 tgaattccta cttcatttgt caactggaac tgttagctcc attcgatcgg acggaaagcc
122041 aggaaggctt aagggaagga aaagcggtat ttgtgaacct tatccgattt cccccctttg
122101 cgccagccgt tttcttgctt ccgagaaagc ctattacaaa aggactagag tttttgttaa
122161 agggagggga gattttatct tcatccgggg tttatttcat tcattatgaa ctaaaaagtt
122221 taaggctgat tagtgaggtc ttaaaaactt catttcattc ataatgatcg gccattccat
122281 ttgtgctttc agcaagtagg cgacgcgaat ctatgctttc tatgttcaat cggataccaa
122341 tttcattgct tggatgcgtt tgaaagcctc gagattactt gcagaaaaaa gctttctagg
122401 tttgtcttgt gtctccgtaa caaatggtcc cttttttat gggcgaacga cggggattga
122461 acccgcgcgt ggtggattca caatccactg ccttgatcca cttggctaca tccgccccta
122521 cccccacaca ggtttaagtc tctatctacg atcagatcct ttctgaactc ccgctatcaa
122581 agagaagctt tttcctatac tatagttgca agtctattgt tgtgtttcgg aattagggga
122641 aacaaagctt caacaagtag aagcttcctg gcaaagcttc aaacaaagag gttgggctgt
122701 tcacttcatt gatttgactt cactttactt aagattaaag agaggggccg ggcgggcagt
122761 gaaggctcat ttagtagaca gcgagcaagc agcaagcacc tactccgatc aaaatgaaaa
122821 gcagcaagcg gaacttgaag aagcgagcct ctatcagaga aagcccttgc tctaatgcct
122881 cgttacgtcg tatacttcac tcgctcgtta gcgccctcct tcagctggct tcgccctatc
122941 tattaatctc tttttcaaaa tggatattta gggatctctc ttgttcatag atcttgaaac
123001 cagatcaata tccatttctc aatatatctt tctatcgata gaaagatata gatctctata
123061 tggatctatc tccatatcta aatatggaag aaccaacact gaaagggtgt aaccaacgga
123121 aggttctcac cctgtccacg acattgttct ccgacgatgt cccctgggcg aaaggggcca
123181 ccattctatt ttttcttcaa tcgttccgat caggacaagt gggttggttc gttttctgtt
123241 ccaatctaca caattttatg tcttcgttcg tgatcatgtt gggcgaaagg tagttgtaga
123301 ggagcggctg tgaaacggcg attccccccct ttccattgaa gtagggaggg cctccttccc
123361 caccattccg gccagggatt ttataccgat gctgaaggta gcttgcttac caagccaccc
123421 acttgagaag ctagtcgcta gaaagaagcg cgaagctgtc tacgaagcaa agcttctccc
123481 cctgtagtcg taatactcgg ctcgtcgcta ctttgattca aaacaaaagg taaccgaggg
123541 ttcccgactt tctccggttt ctgcaaccgt caccatttgt cattccgatt acttcatcca
123601 taaaccttat tttattattt aaaaatagcg atacgctcta aaaaaagtaa aggataagct
123661 tgcattctag aagcggtagc ttcccgcctc cttcattcct actgcctcct tcggtgataa
123721 gttcccttcc ctttttcaaa atgcctgatt ggtctgctca gcaaacgtac aaagtggacc
123781 gcagtttggc tgaccctctt cttcccattc gggttgggtt gacccagaag tacagtaaaa
123841 aggaatggaa ccactggaga gtcgtctgaa gttcacacac acttgggcaa agacgactga
123901 ctttggaagc ggaacacgaa cctatttccg ctattccggg ttattattga gcgtagcgaa
123961 atcagatgag aaagtaagtg aagtttctat ggtgttctac ctttgcgtag tctcggtcca
124021 cagctgacgc aactccgtac cgacgtctta ctactattta acttaattaa tgaacggcta
124081 aactatttca aaacctgagc tttccttaac cagctgacct tacttcgtaa ccttagcccc

124141 tttctttata gttcgactaa gtgacttcgt aacctcaacg tactttttt cttactgtag
124201 cataggcctt cgccacttca aatgggcgct tcgccccccc cctgtttttt attagaaaga
124261 gcggggcctt acttattcag gggggatgaa agaaaaaaaa ggatccgtgg gctttatgat
124321 cggagaaaga gagggggcgt ggttggtgtg tcactgggtc ggtgggggaa cccgtaggaa
124381 ggggggacga cccgccgaat tcacatcaga aatcgccaac atgaacacaa acgaaatctt
124441 gaattgcgta tagaaacaaa acgaaccact tctattctcg gagctgagag aatggctttt
124501 tggtcccttt cgtccagtgg ttaggacatc gtcttttcat gtcgaagaca cgggttcgat
124561 tcccgtaagg gataggtact cattcccggc cgctttcagt tagtgttcat tgctgagtga
124621 tcgctcgcta tctggcttga aagggtggtc cggaagcttc ctctctccca gcaagcaaga
124681 cgagatcacc acttctctca gtaatggact tccttgaatt cttccttagc cttagatgtc
124741 ctttcataga ttctcgaacc tccgacagac agaatctcca tgcatgcgtt ctttctctcc
124801 tcccctcggc catacatctc ttttgttccc tttcttttct tttttccgtt tttgttaggc
124861 attgaacccc accttaggtg ctgagtgctg tcctcccctc cctaatacct aaaaagagtt
124921 ccgtctatgg agcctaaagc ttcaaccatg gcagtaagta gtaagctggc ctagtctctc
124981 gcccagcctt cgccttcttc agtggccaag ttgaagcgtt caacggttct tcggcctacc
125041 acctcaaggt tgagcttgtt caattgaagc taatgctatg ctgcccttcc cttgttcaag
125101 ataaagcgag gactttcttt tagctaccgg cccaaacaaa agagattagc ctcttgatcg
125161 atcgattgat tggatcgaag tacgaagggg ttgattgaag tgtgagatca gcccaagact
125221 aaggccgagc aactagctgc tgcaggattg gacgtctatc tatctcacca cgctccccta
125281 ctcctataaa ggccggcgga aggaatgctc tgctcatctt tcttacggct cgttaccgca
125341 gcgctcgttc accccctgac tataaagctt cttccattca aaaaaaagag gcaggccttc
125401 tgagacaaag caatcttctt aaagggggggt actcaatgaa aggatcggac tttcgctcaa
125461 atggcttcgt tactcatctg gctttctcgg gagatgcact catccgggtg agggagtctt
125521 tcttgggatc gggatcagaa taaggagaaa gatccggatc ttattttgtg gaaatggtat
125581 atccgggatc gaagtgagaa ggactgatat cgaccaaagg gcttaaaacc gaagctaaag
125641 agattcctta ctttgaggaa ggagtttcac tcagacaagg cttaaagcaa tagttgttga
125701 gtacccagat gcaaggcaat cattccggtt atcggagtgg aagcttatct aataagataa
125761 ggaaagggtg gcatagaaag aagagttctc ctcactcgaa taagagtcag acgaaaggaa
125821 atcgaatccc aatattccct aataagaaag ggtgtagaca ggttgtcaag ggaattgaac
125881 tctctcttgc agctgcagtc tcgggctttc actaagtctt ttttcttcac cggaattgga
125941 atctgagaca agagaatcaa gctagatccg atgccggaaa agaaaccttc ccttaagccg
126001 aaagcggcta gagcaagatc gactgagtac cagtactgcc atgcccaaag aaagtggaac
126061 ctttgaaaga ggcccgaaag caagagaaag aaggtttcgt ttaccactgg aactggagta
126121 gcagaactag gacgacgaac aatgctcggc actctgtcaa gtgagcccct ctcattctct
126181 ataagcccta tagttgccct gtatcagtat tcttggagct ggccatgccc taccgtattg
126241 agagaaagta gaatcgcaac tcacggatca aaccaaagac ttatacagtg agtcgatcta
126301 gtagacgaag gtagagtctg agggcaattg ttgactaggg cttgaccaac gtaggtgcgc
126361 tcaccacggc caatcccatt tctgttagga atccatcagt gggagacccc agaccagtta
126421 ggagcagatc cccgcaccct gatagtctga ggcaggccgt tcatcggtct acgatcaggt
126481 tgggcgaata gacggttatg taatcagtct ggaggaaact tccttttacc acccctctgc
126541 cgcatagaag tcagctttca agttagttcc ctcatctcag ctggaaaaaa atccaggatt
126601 ttgccatcac ttcttggaag ggaaaaagct ttcaagtcaa agttagaaaa ctctccttgg
126661 ctacaactgt ttttagtcta tggatggaga gaaaccagag atgtttcaga ggatgcaaat
126721 cttatgcttc taaagtcttg gcagatattg aacatttgat ccaagggaag ctggacaaag

126781 atcccaagga actatgggaa ttggactctg tgtaaagaat gggggattac ggactctgtt
126841 ctttgtaaat cgtttttctc tttgttttgt ttttgtgcta gttctctaac tggtgttgcc
126901 ctttgtttgt ttctggggta tgcccctttg cgtctgtaca ttctttctcc tttttcatac
126961 aaattttact tacctccccc caaaaatcat ttgaccggga cgattctagt ttcgaaatta
127021 agacgttaga ttagggtcat attctaccct gtggagcaac ttccttcagt taagatgtga
127081 gctaggtgtg gggctggccc tggttaggct aaggaagcgg gccccctcttc tgaggaacgt
127141 catggtctaa gacccctacg acttaattac ccctcgggaa ggaaccaaga agaaggcgaa
127201 aaaaaaaaca tatggttcat agcgccggtg aggctcacaa cagggaacga aggctggtcg
127261 agccctccaa accttccttt catagaaatg ggcttattct tcagccggaa aagctgcctt
127321 ttattcgtta tctcttctca cccgccacct catgcgccaa actaaaccgg ctcgctcacc
127381 ttcaagttca agagtcgtaa gccgaaacgg cagcatcatc gttgaaggat tcgagtatgc
127441 tgcttagctt aaggctttga cttatataat ataacttttg ataagtgaag aataagaatg
127501 aatgaaagga gttgcgttca tgcgataatt tgattcattc cttatcagag agagacccaa
127561 gttaagaact aaggagagga aagtattcaa tccttgagga ttgaccaact tcatagcacg
127621 aatacgaatt agggctcttt aacagcatct cgtctccgac tgagctgatt agctgattca
127681 gtgccttttt tgtgagtgaa agtgtgggta aagaatcttg aacttgaagg tgagcaagac
127741 tcaaaactag gcaacaggtt gcgttgcccg gggtgaaaga caccatagac gaagtgttta
127801 atgtagcagc tgtggcaccc gaagacatag gaaaactgac tcttcatctt tttctttttt
127861 taatagacgt cacccactaa aacgtcaact aaatgaaagc ctcttccggc gcttgtccaa
127921 aaaaggaaaa aagcaactca taaacccaaa aagtttcacc caggtataca tatccttacc
127981 tcatctctgg ttcaggacca accatgcact cacacgactc tattcttaag cttgcagtag
128041 tgccgttgct ctagtagtcc aaccatgcag tagcatacca atgcagatat cggagatgat
128101 actccatcca tctttctttc aaaaatcttt aaaacatgca atctgcatcg tcaccataga
128161 aagtacgaat ccgatcaatt tcaacttcat tgaagtttga tggacgtaca cgctccatga
128221 taaacgccct taccattcca tactgctctg cagtcaacac tcccaaatct ggaaatgcta
128281 accaaaactt caatggtaaa ggaagtatgc cagtcagcga aaacattaag agataatggc
128341 gcttccaacg tttagatgcc ggaagacctt gacgtgagta gaccttataa gagaagatcc
128401 accgcgtaac ctgtacgaac acagcaaatt gactcgcagt tccgaaaaca ggaaagtgga
128461 aactccagaa gagaagagag agtacggaga acccgaaggg acacaggact aaagtaaagt
128521 gtaaccttat agaataggcc ataaatcttt tagcaaattc tagagcactc acgttaggga
128581 cttttatgga ctgatcagca cccctttggt cgacttctct tgtcacaaca gcgtaaatga
128641 ccccgatttc tttgctctcg cgctcaaact tcttcttggt gagcaattgg aggcgcttct
128701 gtctttatct ttaaagggag gccctttagt aggggcgtac tcattagact actgaaagga
128761 atatgatggc ttaacgcccc aactgttatg tagttctctc ttatatagaa ataggtatcc
128821 tcgccctagt agtgcggact cttttctgag tacgtggcta tcccactagt agggacttat
128881 ttgcctgtaa gtagaaggcc ttttctatct atttaagaag aatccctttc taatttaata
128941 ggatagtact ttttttatag ttattgctat agttatcagt cattatcagg tagatcatct
129001 tggtatttga tgcgcccatc gatcagatca ggatatttat ccaaaaatga aggaaacatt
129061 gtggatatcg tcttttctaa tgccgggtta gtaacctctg taggtttctt ttcatcttct
129121 atttcatgaa atatatctgt aataagctcc ccagagaatc tcttgactag taaatcaagg
129181 aactcatccc atcagtgccc tcttgttagt gctagtcagc taccatcctt gatataggtt
129241 gttctcctgt gctcctgtaa gaagtccttc ctatacctga tgcgcttttt atcctttttt
129301 tagttaatgc aaagacccaa ttcgcccggg ctgggatggc ttccgaccta agcggggttg
129361 caccgtaggt acccgcagtc tccagagtgt tagtctgcag gcagagactt gtcatcatcc

129421 caacgtcatc aaaaggcctt caatccagta gtatacttat aggtgtccgg tctttgaaac
129481 cagaccgcag ttaaaagccc gtcaactagc tagcctacca taccacttag aaagacggtg
129541 acgaaagttg tcacaaatca accctatttc ctagagcaga tatagttaac cgaaggggtt
129601 gagggattct tgctttaatc cgtcccgcca ttccttactt aggaaggaat atagtaagag
129661 acgactgctt atccatcatc ttttatgagt gaaacaacga gctctatgac tatttgtatt
129721 ttagccgaag gccttttgac tgttatagtc tctcgcttgg actcttctca cgaattgatt
129781 cgaaccaata tgctcctttc ggcgcgactt ctccttttag ttgatcgtga gtgggtctgt
129841 cgtcctcctc attatagtcc tcctagaatc aatagcattt cgtcggaata catcctgtct
129901 tttcacctta gtagtcctat gcatagtaag tactatagcc ccaatcatgg ctactaataa
129961 aatcagacta ggaaccaaaa accagacgga atagtaagta taaagtaaat tgcccaatgt
130021 ttccaaatta gtccaacttc ggatcatcaa tgggaggttc tcctttctct ctcagaatga
130081 cgacccttaa attctcgtaa atgatattat ttctccaata cgggggcttg tcgtctaaga
130141 aagatagttc ttgttttata accttatgaa agtccttgag gcttgtaagg gaagaaggag
130201 atgggttctc gaagccgtag tcgcttacaa gtgatttccg caaagattca aaaaaggtaa
130261 gagtgcctat ctctacggca gcctctggat attcttggat gatctctttc tcaaagatat
130321 ccagatatct agagatacca ttaagaagac tagttctttat ggcttctttc tcattcagta
130381 ctgcttgctc gagcgtaagc tcggagcttg atgctccaga cgttccatca ggcaacatca
130441 tcttatctat agggagcgtt gcttccgcag tgacgagcgc tctaacagca caccctagaa
130501 gcattgcact cgccccccgaa aaacccattt tagtgaaaat ggaagaaaga attttgactg
130561 ctactaagcc cgcccctttt tttagaacac ctaaaaaaaa ggggatcgcc ccaatattaa
130621 gacaaaaaac atataaagca atcaagacaa ctagaagtaa taaaaaagga atgaaggctc
130681 gtgctacgct tggaatgtga gcaaatatat catgtcgatt gctcaaccgt actttggcta
130741 tcttacgtgg agctgaatta ggttttttcg gtgaagtgaa atttttgtta gtcatcatgg
130801 gaaagaaagg cagaaataag aaagagatta ttcccttaga gcttgtccaa taccaccagt
130861 accagaaacg catctccttc gcccgcgcaa tagacttcta tgccagccgg gaaaaatggc
130921 tctgtgcatc ataggtaatt taaagcgctt tcttttaaga atgcatctgg ttccatcttt
130981 ctttcgttag ttaacccgcc tttttctaaa agtttttgta gtaattctgg ttttacacta
131041 cttggaatgg ctctctcata ttgagaaatt ctgtctagtg gcattcgatc acagaatcca
131101 ttgacagctg cataaatgac tagaatttgt ttttcaatag gaagtggtgc atattgtggt
131161 tgtttcggta cttctgtaag ccttgcacct ctattgagta atgcctgagt cgcagcatca
131221 aggtctgacc caaattgagc aagggcggcc acttcgcgat attgtgccaa ttccagtttt
131281 gaactaccgc agacttgttt catagctttc aactgagcgg cagacccgac gcgactgaca
131341 gataggccga cgttaatagc aggtctaatt ccgcgataaa agagctctgt ttccgaacag
131401 atttgtccat cagtaatggg gatcacattg gtgggaatat aggccgatac gtctccagct
131461 tgtgtttcaa tgaccggtaa ggcggtcaag ctacctgcgc ctgtctggtc cgatcgttta
131521 gcggctcttt ctaagagacg ggaatgtaaa tagaaaacat cccctgggaa agcctcacgg
131581 cctggtggtc ggcgtaacaa taatgacatt tgtcgatatg ccaccgcctg tttactaaga
131641 tcatcataga ttattaatgc gtgcattcca ttatcacgga aatactcccc catggcacac
131701 cccgaatatg gggccagaaa ttgcagagga gcaggatccg aagcggtggc tgctacaaga
131761 atggaatatt ccaaagcatt cgcttctgaa agaatttgaa ctaattgtgc cacagttgag
131821 cgtttctgtc caatcgctac atagacacaa tacaatgtct cactctcaga ggtgctcctt
131881 gagttcattt gcttttggtt taatatggta tcgatagcaa ttgctgtttt tccagtttgt
131941 cggtccccga ttataagttc tcgttgacca cggcctatag gaaccaggct atctaccgct
132001 tttaaccctg tttgcatagg ctcgtgcaca gatttacgtt caataatccc aggggctttc

132061 acttcgacac gtcttcgctc gtgatcgctt agagcccctc ttccatcaat aggtactccc
132121 aacgcgtcga ccacgcgccc tagcatagcc tttcccgcag gaacatccac aatagatcca
132181 gtgcgcttga caagatctcc ttctttaata gcagtatcac taccaaagac aacaatcccg
132241 acattctcat tctcaagatt caaggctatt cctttgacgc cgctggcaaa ttcaaccatt
132301 tcaccagctt gaatctcgtt caatccataa acacgtgcaa tcccatctcc aactgagacc
132361 actcgaccga tctcatccac ttgaaaattc gtgtaaaagt ggctaattct actttctaat
132421 agagtcgtta gttccgcagc tcttggagag tttttcattt gaaatatttt tgtatgtttc
132481 tagagtcaag ggagaatgcc gttttttgtt tcatccgact tagattgaac tcatgcatgc
132541 tgtcgaaagt catcttttgt tcgaagtaaa tgaatttctt tcttctttta cgtaattata
132601 aacgaccttt ttgacgatcc acaggttcta gtggaagagc tggaccattt ttttttttct
132661 tttcttttct ctcctcttgg agaaattaat tgcactggac gttttggtat tggatccgct
132721 cttaaaaggc ccctcctttg caaaatcacg taggtcgact tttctttatt tctttcatta
132781 attccagaaa gaaggcggat gcttctttct gcgctaccaa ggaatgtaaa ttatccaacc
132841 aatccaaatc tcccctgccc tcgtggagtt ttcttacggc cgcctccaat tcccctctgt
132901 ataatggaaa ggaatgcctg cattcattat gaaggacttt gaacatttct cccctccatc
132961 gatccaccaa taccggatta caaggctccg gttcgctctg tctaaaacga gaaaaatccg
133021 gcttccagtc gagaattcgc tgaagccgct cttcctctga gagaggcggc tcagaggcca
133081 agggcagtga atcataactt ttctgattca tacgaggaat gactagcttg ggaacatcac
133141 tcttagatac gagatttccg gaccttgcca ttaagttagc ggcaaggctt gtcgtaggct
133201 catcccttac ttcaagtggg agatcagcgg cattggtaag cattacggtc ttaagccaat
133261 cagtctgagt agacagaaag cgtaggggcg ttagtgcgat tgattctccc ttagacctcc
133321 ttatccataa aagcccttct tagtcaagct tcggccctat ggagtaaagg gaagtgcgga
133381 tctttagata cacaattctc agtccagtcg aatgcgagcc aggctaggta agccagtgga
133441 tcaatcaagc gagccaatcg gtccaaagca aagggcttgt cttcggtctg tcaattcact
133501 gccttctcta ttcgagcacc ccggctttag tacgaccact cagaattcac cgattttcca
133561 ttctgaaatt gagccatgcc acgtatcagg gcattcactg ctataagcta agagcatgat
133621 ccgggttcca tctcaaccat cgggaaataa gcagaacggg atagaaaggc tattgatcaa
133681 agcgcctttc ttatctagaa gcatcccatc aagcaagaaa gcaagtccat caaaagaagt
133741 ccattccatt ctgcattctt ttacttggaa gactaaaccc gcgccttacc tagggctgca
133801 agtaaggact tcacatccgg attggacagc aaagactgga aaccggtaaa gcggaaagtt
133861 ggcttgcctt gttgagatag gggtggaaga gtaaagatag tcaaaggctg actttacata
133921 agcagaggat tcaatccggg catatggcta gatgcctaat acgagctgaa ccttcctttt
133981 tctaaaaacc cgaattgaga accaccgggg tctcactagc gtcccgttct gcgatcactg
134041 attaaaagaa ctaatctttt accaccttat atataggggg ctgcactcac tcagaagcta
134101 gtctcttagc tgcaccgtag gaagagggaa ggatatacta tagctagccc aacagaaagg
134161 gaaaagaatt gggaagaaat gggggccctt gatcctagat ataagtgata tttcccatgg
134221 ctggaaagat agcgagcgct attctcaaaa tctttctttt ttcgaagggt ttcagtggac
134281 ataagggcta gatcctcttt aatgcattcc ttattgctct atccatagat ataagacaaa
134341 gaggcaattc aaggtcttat gcttagagtg attaaagaaa gtaagacagg ccagggaagg
134401 gggttagcac ggtagcggta tattgatccg gtacgtatgg atcaattcca ttttttccg
134461 cagaagcaga cgaaagaaag gctgaggcat atacatctgc ttcggcatca ggaaaggaat
134521 tatatgatta tgaagcgttg gcataatcag aagaaagaga gggaacaatc cgagcatgca
134581 acatcagata ggtaggaaca ctcatattca tgaggaaaaa gcctcttagg caattataca
134641 aagagctctc atagttcaaa aagacgtgtg ctaggattct aattgtacaa aggcgtgggc

134701 gtgaagagag attaatgaag aaaagaaagc aagaccgaag cataattgac ttagaaaagc
134761 aaggggaacg acctgggcac tacggttcac ctgggtgctc acttggaagc aagagaggta
134821 agaggccacc tacgggaggt ctcctttcat tccgagctgg gtcaagaaag aaaaaagtcc
134881 taggtagaga tacagcagga ccaggaggaa gtgcggacag atggattgcg ggaaactctt
134941 tattttcctg cttagacaga ggccttgtaa actacatatt caaattagca tgaaagtaat
135001 attttatatg atacgcctcg ctgagctctc tcttgggctg tgaaagcggt gcgaaagaag
135061 ttgggctacc ctgccttgag atcttcccct gccctgtatg agttgagagg ctgaggtcca
135121 gtctactaac tataaggcta ctccacctga cgattgttgt cttatttcaa taatgctttg
135181 ttggtcaccg ggcacagcgc tgcgagaaaa agcctagcgc gcagaaaaca gagaagaagc
135241 aggagccaag cagtccaccg attcggctat caatcattta cttgccttgg ctgcgttagg
135301 ataggactag tgggccaaag tagagcttca agcatggcta tcaattgacc ctatattttt
135361 ttgcggtgga cctcgaacga gtcccagcgc cccccgctaa cccgagtgaa cattcaaata
135421 tctgtcctac attcaattcg tgaaggtact cctaatgggt tgaagaccat atcaacaggt
135481 cttccatctt gcaaataagg catatcttgt ctaggcaaca tttttgaaat gatacccta
135541 tttccatgtc ttccagctac tttatcgcct acctaaattt cacgtttctg tgaaatatat
135601 acacgaatcg tttctggatt ataactagaa cctccccttt tctggatcca tctcacatca
135661 ataacccgac ccctaccgcc tataggtagt tttagacaag tttcttttga agtagatacc
135721 tgaatgccaa gtatggctcg taacaatcta tcttccgggg catacgatga ttctttcacc
135781 atttggggcg ttaatttacc tactcaaatc tcacccgtct ctacccaaga tcccagcatt
135841 acaattccgt ttttgtctaa attgcggagt aaatgggctt ctaaatgcgg tatttcctta
135901 gtgacccttt cggggccccg agtcaggaaa gctaaaaaga gagtcagtat agaatactgg
135961 taaccgctga tgaaagccat tatacgttca agaataagtg atgaagtctt cagtttagag
136021 taacgaacaa ttcaccaata agctaagcct ttgtaggcga agaattgggg acaaaggaga
136081 ttttgactag cacttcaaga gtaaagtaga tttacctgcg ttcccaaggg catcccctta
136141 gtatccccgg ccagtagcaa gactatacgc tgctcacgct catcttattt tgaattccac
136201 ctttcgttca ttctcctaga aagacaaagt cattcaatag ttattaaata caggattggt
136261 ttctttttcg cattttaagc taaaagcttg aagaaaatag acagcccttt agagatagag
136321 ataagagcgg cacctcaacc agccacaacc gcattagcag ttcgcgtgga atcagactaa
136381 accaaggtca aggaagactg aggtgaccaa atcgaggacg atgagctatt caattcagac
136441 gaggaatttg acacaaggac ggggctttca tcccaaagaa gaagagctag cggcgagtaa
136501 gcctacgaac gaccaccgct tatccaattg aatttccaat tgcagcagaa gactgacggc
136561 agaattaaac cgttttgtat cggggtcgta cgcctggaac aagaaggttc gtcgtacaat
136621 ttcggcgatt aaaaaaagaa aggagtatat ccctctccct tagtgtcttt ccacttccgt
136681 cgttcaggaa caaacacttc gcctaattct tttgaatccc ggcccggttt ttccccacta
136741 actaattacg ttacgaccac tgaacaaact tggttgacga acatggttta tgcgccgcta
136801 atgtagcggc ttgtcgagca tttgacaaac tcacaccatc catttcaaat aggatttgtc
136861 ccgtggacac acgagcaatc caacccgtag gatttccttt tcctcttccc attctgactt
136921 ctgtaggttt cccggtaata gggagatctg cgagaactct tacccatatc ttaccatttt
136981 ttcggaattg tccgctcata gcacgatgga attgtccgat tatagcccga cgcgctgctt
137041 caatggctcg atatgaaaga cgaccagctc tacaactttt agtgccatat cttccaaaac
137101 caagttgtgt accgtccggt ttgcaacccc tactacatct gcctttacga tatttactat
137161 attttgtacg tttcggatat agcacgtcct ttttttttt tatatgaaat ccacactttg
137221 acacctgaga ttccgtaacg agtagatact tccgcaggag cataatctat tttctggtta
137281 aatacattac gagatgtttt tccatacttt ccgcattcag ttctagctat ttctgcacct

137341 tttgatcgac ctgaacaaca tatacggatc ccctccaccc cctttttcat tactaatgga
137401 atatccttca ctatttgact aaaaatggaa cgaaatgatc ttgttttgtt cctcggttga
137461 aaagagatgt cttgagcaat cggagaagca ctttgataaa cagattttat cttgaccgac
137521 tcaattaagg tattagtatt tgttctatta aacaacaaag atcgcatttt tttaacttcg
137581 ttcaaataag agttgtaccc gtacggaatt attctgtttc tcagtatgat ctctagcatc
137641 atctctattc cctttctcaa ttctcctatc ccacctaggt ctatgaattt ctctatcaat
137701 tccattacct tatccttacc catgaggttc caacattttt tccttaattt acctaggagt
137761 ggttcccggg cgtcctcaag gttcttatac accccaaccc cgtcccttgg aaagaaaaag
137821 gtagcaccaa agaaaggaaa gagcgagatg gtggtttttg ttgttcccgc gaagcgaaga
137881 ttattcgcca agcgaatgaa gtgggtcaac ctctttttgg cttcggccaa atacttttta
137941 tcaccggtcg agctttctac aaaaaaggcg atgcgagaac gtattctctt atctaagctt
138001 cttccctgtg cattagctcc ccccatcgta gatggttctg ccacgcccga tgccaaaaaa
138061 tgattgagaa ctacgacggg gtcgaaatgc atttttttct ttgtattcaa taagtattgc
138121 atgaccgaaa aattcaagga agggcgcact gcagggaggg tccttttcat ttttagtaga
138181 tgactcgtcg ggctgtcgga gcggaacttc tttgggaaaa agaactttaa gaacttagtt
138241 tttctgaaag agtagtcatt ttttatcagg aacgctatgt catttacaac cccagcgtat
138301 ttcggatgct tgaaggcccc gctgacccga agtgatttag aaagattctt ctttatcgat
138361 ggtgatcggt catggtatcc atagcgttgc ttctttttcg gccaaatccg gatttcattt
138421 tgcttcttcc ggtcgtcgag cctgatcgac tcgactcttt tccctgtccc ccggcctctc
138481 acttcgtttc gttctttttc tgtatcgtcg cttgaatgaa gacacccgat cggcccgact
138541 ttcccaaatg cccaccaccg gcccttcttc tttttctttc cgggtctgga tttttcgcgt
138601 cgtttcagtc gtcgtggtcg acggggaaga aagaaatgaa tgaatgttct tttgggaaaa
138661 tgtagaataa gacacctacc gagacgaaag ccaaaggtga gtctcgtagg tggacgtatc
138721 gaaccaaaat aagatctcat attaacatct tgatacactg atttaccata ataataaata
138781 gagtcaatgc tgactccgtc gtgggaagag ccgcttcttt cttgcttgat aggggggggct
138841 ttcattcacc agcgcagact aaaagtgctc tccgaaccgt gctggatagt cacccatcac
138901 acggctctca aacccaacct gtggtgtatc ccggggggaca aagtaaaagc gtttgatcct
138961 ttgccccata ctttgagaga ctcctccccg ctgatcaagc agcgacgctg ctcgtgataa
139021 gcttagcttt aagctgctat cgtttggttc ggataagtca agtcccttcg gtcggttcgc
139081 tcaggtggtc tttccttatc ttccctaacg ttcagattct ggtttgagaa aggagcaccg
139141 gccgagcgcc ctgaatgaat aaaaaattga cagcagaggg aatcaatgat tcttattcga
139201 ccgagttgaa gctagcaaga tgtcgattac acacacaccg gaggttggta agaactgagg
139261 gacaatgccc ccctaaccta agtaggccta ccagcgaagc aactggtact tgatcgggcg
139321 ggggcggttt ggtttcgtgc aacgccctcg cagcaggaag aggcattttt tgaaaggaaa
139381 cgccctttgt tttgtatagg gttccaaacc tgcctgatga aaaagccctt tctatcttat
139441 tagtcaagcc taaacgtcct tattgaaaac ggccttgcta acgagcaatc tttctaaagt
139501 gctaacgcgc cttctattag aatcttagta aagcaacctt tcgcctgaac aagcttactt
139561 actaattaat aaagcggcaa caagcacctt ctttctcaaa gtcaagtttc tcccttcttg
139621 ctttagtttg attgcagcta gcgcgattga ctaatagaat agaaagtcaa ggctcttctc
139681 ctgttctacg aatctacaac tctctttact gagcgagact ccatccatat ccctactagt
139741 ggttattctt tcttttccat tctatcattt gtttgacagc ttaaactagg ccccacttta
139801 gataggtttt cggtcttaat caagataggt caagggcgcg ccggaacggt cggtagctgc
139861 ttagtctcat ccgagagtat aatctccttg tactgctttt tttcaaagaa aaaagaaaga
139921 agggggttga tttaggctcc ttcccttcca ctgcatagct gcgctagcag gtactacgag

139981 ccctctgtcc cacgcatcta accagctcgc gtggttcacc ggtttcaccg aaaactctca
140041 tttgttgaag cggagcatag tgcgctttag gcgccgagcg agaaacgtct cttctttcgt
140101 ttcggtttat tccttgatct gaaacttagt acttgttttc ttattgattc caagggcggc
140161 ggcgttcttg aatgaagtct atccgaaccg aattagcact gacttctcag atcaggctag
140221 gcctctccag cggagctggg cgccgggccc tggatgcgct agcgattggc acaaagggga
140281 ttgcccgggt ttctcggcct ggtatcctgc acctcgcgtt catgatatct acattcaact
140341 gtgccccgga gacacggtcg aagcacgccc gcccagtgtg cttctttcac gacatgctct
140401 ggtcccgggt gtcttccagt ggtcttctag cgttagaaga agtcgtgtcc gtcccggaca
140461 tctgcaacgt cctcccacgc ctttcttttt ttcttttgga caaacggaag gaaaagactg
140521 acccatttgg ttactttcgc ggtcgccctc actgaaccga cttgaatctg aactacgatt
140581 tttatcaagt cttaccgaaa tcggatttcc ttttcgtgct atatgcgctt atactgaact
140641 ttattttccc ctttttttc ccggtccaat attagttctc gaaggccgtg taaaagcaaa
140701 ctatccatat tgatgaccaa gcaaggtcgc cgagaacact aacggaacac tttattattc
140761 ccactgaagc tagtacgcaa taaagaccaa tccacgagct accttatgac atccaatagc
140821 ggagacataa attgcatctc cgtcttggct agaatccttt tggttcttct tcttttttat
140881 cgaatcttaa ccatgaaagt cagtcaatgc tggcttcctc tcagagttgg ggtcctcacg
140941 accctttcct tgattacctg agtgaggatc ggaagaagat caactgccaa tgcaaagtcc
141001 gcttagttca aaactaagaa agccgaaggg aggaaggcat catagggttt ttttctaggt
141061 tcaggggcga atcgctaaag gtcgaagtca acggcatttg aaaatcagac tgaacccgcc
141121 catctcatct agaataaagg gttgatactg ggaaaatttg atcttctatc acgccatttc
141181 tttattagta gcgagtagca agctacaagc atgaggtctc accttatctt ataggtcata
141241 tatctatttt atttaaatcg gccaggagcc ttggttccac tcgaccggag atacgacgtc
141301 atttatcgaa aagacctttc actagccttt caccccttt taaagcaatg aatgggatca
141361 gcgtggtagc tcgaccggat ggagttggcc aatagtgact agccacacgg agtagggatg
141421 ccttgaggat tcggtatcat cttcttcacc ggacacctcg gaagccaact caaactcaaa
141481 gcattagtga tcaccgcctt tatgatgagt tttccgcatt cgattgaaac gaactttctc
141541 tttctatagg aaattggatt ttctttttga atggcaccta catctaattg aattgatatg
141601 gtcgtgtact atactagata cacagagggc tcccttcagc ttggcagcta ggacagaaag
141661 ctataagaaa gaaaaaagaa aaagcgcact caaggtcctt ccttcactta aatggtctaa
141721 tggggcaggg ctagcctgaa agaaagtgaa atacaaatga ttgacacttg gcgaagactc
141781 tttcccctaa tccatgaatc taatttttcg ctaagaaagc ggcgggctac tccaataatg
141841 actttttgtc tgggttcttt cataaaaaaa caaaacaaca gcttgtagct tgcttctgga
141901 ttgagattga ttgtggcgat tcccgtcttc tgttcgtgac tcgtacctga agctaagtcg
141961 gactcaagga ggcttcaaag ccagatctga tcatttcata tagaaaagat acctatccat
142021 ctcaagttat cccctttgg cttgaatccg accgtcaact tcttcaactg ctctgatgcc
142081 gctaaccttt tttttgattc tttttactaa tagtagagca acctatataa gggaaagact
142141 cccctaggga tctatactcc tatataaatg atctactgtc ctaagttagt gttttttata
142201 taagcaaggt ggtgggctag tgttatatta tatgctgtac tggggtactc tacttaagtt
142261 atctacctat actacatgat atcttcctac taaaaaaacc cttgctagtt ctatatccct
142321 tggtagggtc tttctatatg cctagattgg ttaatatgcc ggtaggttag gtagggtatt
142381 ctatctccct tgctctaata aatctcccag ttttcgcata tccgctgtgc tgtctaataa
142441 agagaagtta ccttccctat cgtatcggtc tgtccagata gtcatttcat ttgtgattcc
142501 gcttttcact ctgtcggact gccttgaatg aatgctttca ttcagggcat cagctgacta
142561 agggaaggga gatgcgatct ttccccaagt ccatgccatc agagctcaat tcaatcgagg

142621 ttttcattgg tgtatagata gcgcgaagcg aaggaccgga ggtccgagaa ggtaagggac
142681 ggctactttt aggaaaactc cttatgttcg cagcgcaggt tgattgagtc cggtagcctt
142741 ccaggtcccc ggttatataa taggggaagc agatgcgagt gcagttcttg catgtgttaa
142801 taccatgctt ggaaagtggt ccccccgcct taacaaattc accgatgtga tgtctcaagt
142861 acggcacttt ctccagaagg gaaaggaagg gcttaacagc gcctaatcgg gaccttgtta
142921 aagttctctt ttctaggatc aaactctggt tcctacattt tccgtctact ggtcccgggt
142981 tccgggtgag ccagaatcgc agatcactct ctctccgaat agatcgatca agcttccctc
143041 ttcaagccaa atatcactcc aaacgttggt attcgagcca tccccaatga tggatcttat
143101 ataccttga aagaacgtct ctgattgaga ggatcgatct caaaatgttg taggtgtggt
143161 aggatggacg tacatcccag aaggtgctcc cgcgaatgta gtcttcctgg attcaattta
143221 cccataagca aggtttggtt tgttggaaac tatattccct cttaacatca ctaagaaatt
143281 ctttttcttt ttagttccgg ctcagcccgg ttcaagatcc tttccatacg cttaagcgtg
143341 cctttctgga tcgcaaaagc cgataacaag aattcatccc attaggacgg attgaaagag
143401 gtctcgttta gagcctttga ttcattccct aaagagcagg cttggaactt gaggaaaggc
143461 aagtaacctc ttggcctgtt gcgcccccaac ctaccttctg tctcatttgc ttcatgtctg
143521 gttgagtgac tagactgaga ctctctttga gaaaaatcaa actttagaga agaaagaaga
143581 aatatcgtaa gagaagaaaa agaatcttac gcccaacaac tcccatgtct ttcttttctt
143641 ggttgaacca accagcgatt tctttgattt cgttccaagt ctcttctctt ttcattttta
143701 gagcaagaag aagtctctct tttttggggg ggagcagagc agtcaaagaa taatgaacca
143761 aatgattttt ctagaatggc tattcctaac aatttctcct tgtgatgcag cggaaccatg
143821 gcaattagga tctcaagacg cagcaacacc tatgatgcaa ggaataatag acttacatca
143881 cgatatcttt ttcttcctca ttcttatttt ggttttcgta tcacggatct tggttcgcgc
143941 tttatggcat ttcaactata aaaaaaatcc aatcccgcaa aggattgttc atggaactac
144001 tatcgagatt cttcggacca tatttcctag tatcatcccg atgttcattg ctataccatc
144061 atttgctctg ttatactcaa tggacgaggt agtagtagat ccagccatga ctatcaaagc
144121 tattggacat caatggtatt ggacttatga gtattcggac tataacagtt ccgatgaaca
144181 gtcactcact tttgacagtt atacgattcc agaagatgat ccagaattgg ggcaatcacg
144241 tttattagaa gtggacaaca gagtggttgt accagccaaa actcatctac gtattattgt
144301 aacacctgct gatgtacctc atagttgggc tgtaccttcc tcaggtgtca aatgtgatgc
144361 tgtacctggg cgtttaaatc agacctctat ttcggtacaa cgagaaggag tttactatgg
144421 tcagtgcagt gagatttgtg gaactaatca tgcctttacg cgtgcgcccg gaaagatagg
144481 ccgactgctg agcccactct ggctcagccg caccacccag ggtgcgagcc acccgagaag
144541 caagctatta cagcgagcgg ctggagagca gtatggagcc gaggagcaag gcagtagata
144601 agatagaaga aggggccagg ctcccggtgg agcagaggat acggttagga taacgaactt
144661 gaaacgcgga gcccgagcgg ccggcgagtg agtggttagt ggccaatagc gccctagttg
144721 atggcattcc tctctgcggc tggcactcga ggaaccacgg ggcactccat acagagcaaa
144781 caagtcttag ggatgagacg cccgcgcaag gacctcaatt ctcattagga ggtcgaacca
144841 aggacctatg gaagtcgggg ctaccccgtc cctatgggca acgcaacagt gtcctgaggg
144901 aggagtttag aggccttata gtagcacgga cttttttgct ttctaggtca tgcgaagggg
144961 gccagtccaa gatcgtactg ttcctttaca aagacaacag acgctctcaa cggctaggcg
145021 ctactctctt tctgagttct tccagcttct tcatgatttc gtgccgcggt gaaaaaaaaa
145081 aaaagagggc catctcagcg gaaagagaag gacctgcaac ggcagagact actgaccctc
145141 ttcttgttcc tagccgtctg ttacgagtcc gggaagcctc ctcgagggat ccctcaaaat
145201 agagaagggc gggcagggct tccgcaagag cctccccccc tcttcccggt caagatagat

145261 gggaaggagc cctatcaagg ccataaccag cctctttctt tatgtacgta cacctttgtt
145321 tcagggtggg gccgcccttc ctcctgctaa tccggccatt tccgaacctg tcttttggag
145381 aggttggagc tatgaagctg accttcttcg ttattagaga aaggggaaag ggctttttca
145441 gcttgcttgc tcgctttctc gcttccgaga ggcgaagggg cctgacttac ggtttccaag
145501 cctggcgcga agggattgga tttcacctat gatcagatcg gcgggcaacc aaagtttact
145561 tttttcgtgg tgttgttgac gttgttgaaa gcgaatttta aagtaggcct cgcttttcag
145621 agctgctccc agctcacact atggtggagg aggtgcccgt gaagatctag gagtgtgagc
145681 agtacgagct gaaaggctcc catactgttt ggagggcagg gggcatagat gccaaacaaa
145741 cctgacccct atctatcgtc gtagaagctg ttcctaggaa agattatggt tctcgggtat
145801 ccaatcaatt acaaaccggg gaagcttcca cgaaaccgct ttttacagcc tcgccccagg
145861 aggcgatcat ccgggaggga gtaccagtcc ctccagagaa agtctgggag gcggtttcct
145921 ccggcgagta ctactactca tctcaagttc acatcgatgg agatgtgaag gatgtggaga
145981 ccatcaatag tttatctaga ttgaatggtg tcctcctgta tattcaagaa aaaaaggtga
146041 atggggctat atgggcctga ccttgttcaa cctatccagc tagaagtaga ccagacggaa
146101 gaagccttac ttccgtctaa gatggacgaa atatgtcgga gggaggcagc gtactttggg
146161 atcaaactcc ccagttcata ctttaagaag gactaggcgg gttaattaac gaaagaaaga
146221 tggaaccaga tgcattctaa aagcactttt agataataat aaaaaaaaaa gagtgacccc
146281 ggtggacaaa tgaaatcgtc gtaaggggga ggccttggtt aatcaatagc agtgagttga
146341 ttgaccaagg gaggaggggt ggtgtggcgg gccctcctca aaatggtttt ttagtcgtct
146401 ttgctagccg cttaggggta tgaatctcaa caaggtttct tcttcctata ctctatggac
146461 aaaaaaatca ttcaataatg agtttgacta tgcggtcttc ttcgagatgt aattgactgc
146521 taaaccatgt ctcccccttg tcaggaagtg aagcttcagt ctttcttttc tttttttaat
146581 aaagagtgcc tttggaaaga ccccatatgc atctggttta tccacacacc ccctatgaaa
146641 aaggaaagac tccttgggta ggtcaaagag agctggggaa ggatcattcc ataggcttgt
146701 tcgctcaggc tcagtggtgg gtagagtaac tggccgagaa ggttagcgag gtccttgcta
146761 tggttaagtt caaaatcttt cactatagtg gggagaagac aggtgggagc aagcggagcg
146821 agagcaaagc aagccctagc ggtggcaggt cccaaaaaac tatttttcat cgacatagtc
146881 agaagcaatg gagatgttga aaacgcacaa gcgaagcacc ttagtcagcg gaagaagtca
146941 ccctcaattc gatctagcaa gaaatgcttt tccgagaaag taaagcgaga gacagtagat
147001 tccttcttag ggttccaaga aattcgacta gtgcaaagaa agatgcagta tccagttgta
147061 gggcgacgca gcagagggaa gttggcacaa tatgtaatcc ctagggtatc gatgtcggag
147121 aaggggatca ctcaatgctg ctacaggcac tcattagttc ggattgggta gcgtgaagga
147181 gataatagaa ggacccgccg aaagtagttc agttttacca ggtcttccgg agtacaaatc
147241 tcctcatcca atagcattct tagttagtac cattcttata agcctgtttt tgacgagtga
147301 agcgactagg ctaagcatgt agcctagtat tcccggattg gtatggtatt ggataagaaa
147361 gattccgctt tcttcccact catgaagcat gaagaacaag aaagtggggg gagggcttct
147421 gtggaaaaag gggaagtttg ggactaaact aaaatagtag tcaccaaacc caaagaaatt
147481 tgactatgtt aaggcgaatt gataataaaa ggtacttcca tgtgtataaa agcacaaggg
147541 gaggaggccc ctagtccaag tatcatagca taggagggtg aacttgggct ggggggaagg
147601 gggagtggaa acgtcgtccc ccttaaatga aaagccagat cgaaaaaaga ctaaaaaaag
147661 aaagaataga cacattagat ataatatttt tttagaaaag tgttcggttc aaaagaattt
147721 atagttttcg tcgcatcaag gaagcttttc tttaccgggt caaggataag aacagggtag
147781 tgaaatggcc ttgctcatcc agtaaacttt tatattacct aggctattat agatagttgc
147841 catgtgaaaa aatggagtgt gacgatctcc atcacacaag cagtgaaccc taatttgcta

147901 cttccataga acttcttgct gatgcagaag gttatggtag aggactactt tgtgttaagc
147961 cttattaaca agctccctta ctccataggg tttcccggtc atcaagaaaa aatgactctt
148021 tccttcctac agccagccct aagaagccta taatagttgt gaaccctttc tagtgatgtt
148081 tacacttttc gtgaacctat tcattcttgc tcgaaatgtt cttcgctcgg tatggcacaa
148141 tagataagta gttggatgag cccaaaaaag ggcaaggact agtgccaaaa ggtagcattc
148201 ctccttctct tggtttgttg gtactgactc ggtgcgaaaa ggttagctca gttctcgcct
148261 ggatcgatta agtagctcag gtcagcccct tggtatgcta agatcagtat cacgcgattg
148321 atccactcca cttggtatgg ttccgctcag cgagctcact tggctagaaa gagagcttct
148381 agcaaagagt tcaatcgata gcttgggaaa gaaaaaggaa tttctcttaa aagtatgcga
148441 gattggaggc tggcctttga ttcgatacgg cagcggggta tggcttgctt tgctcatttt
148501 atcttgatac gcttaccttg cccgtttagg tttgaaagaa gtagacacgg gcagggaatt
148561 tcattcctag ctaggttctt cgtctcagtg ctctctcggc atgttttgct tactagaaag
148621 gtactcgtat gccactagtt cctgtcgaag ggtgagctaa gtctctggag tcactctgcc
148681 tggatactcc tcaaggcatt gtttttgtac gagcagtaat agtataagaa aggacaactg
148741 ctattgaatg cgctgttggg ggagaatgcc ccatttcctt gtctctagaa cccatagggc
148801 caatagacgg ttagtgccat agtagttgta gtcttagtgc cttagggtac tttagatagc
148861 tattccgatt caaagcaaag gaaacttcct aagctaagga ggaagggctt taaaaaaaag
148921 gggtaaggac gaaaaagggc ttagagaaga agctcttctt gaagtgaaga aggagatgtg
148981 gcactttgaa tcgtatcatc catagatagc aaaggaacta gccattggat atcttgaata
149041 ggagacagaa ggaaagtccg tggggtacgg tcagttaaca tttccggaaa gaccacccct
149101 gttaatataa ggaagtccta aagcccctta tcggatcgta gataccagtc aataaaaaaa
149161 aagaaatgat ctaagcactg ccattagtca taggcgacag ttctgttgct aagttggttt
149221 aatatggtat cgatagcaat tgctgttctt acctttactt agagaggggg caacggtacc
149281 acgctctttc gctaccgctc cgtggggaca gttgatcttt cttttctacc ccgtgctaag
149341 atctttttgt gcgggtgcgg gccgttcatg cctttggaat cgtcaggtgc tggcatcttc
149401 tgtgtgacga acatggcatc tatcgccaac tgagtccttc tcctacccta cagtttaaca
149461 acatcaaatt agagctaacg cctagcggtc ttccctggct ctctctttgt ctgcttctta
149521 attgactctt atgagctagt ttccagtcga tagtgaagac catactaatg catatgcggg
149581 attaccggaa tagcaactac cacaggtagc atgtctatca tagtcctaat cttttttct
149641 ccttgttcag aagaaaggga acgctagcta gatgcttaac cacacaggta gcatgtccac
149701 gcttccgatc gctagctttc tttcttcagt aaagaaagag taggaatagc tcggtcatag
149761 gtaccatacg aagaaaggtc ttccattcat tgaagaaaag ataaaacgaa tcaaaaagca
149821 acaacaggct ctaactcggg aatcaaataa caactggaat gcttcatctc gtaagcagac
149881 tttgattgat cgaatgcttt ttctctccta cttcattact tttatatcta ggtctaccat
149941 taggcacccct tcattagaaa gaagtagttt acaaggagga gttcgatcca ttaggttctt
150001 cgatttgttc agaaaagaaa ggagagacaa gaaaacatct ttgattacga ttcaaaggaa
150061 caatctcaaa tagaccaaga tccaatttcg ggtcatttct tggtgaacat gaattagatt
150121 ctattcgttt tcgtctttct cataaaaaaa aagaaccccc ttcttacggc gagtatggta
150181 gcgcatgagt gcgcggtaga ttaaaagaga accaaaacac atttttttgt actatcgcag
150241 tctcgacaaa aatggtttcg gcaccggatt ttcaggacaa ccctgaagac ctttttaatt
150301 ccgttataac aaaaaaaagg aatttgcggc gatatctcat ttcttgagag caactagaaa
150361 aagggagtgg gaaagctgca gtaattcttt ggaaaagccc tctctcttct ctttgtcttc
150421 gagcttcctt taatctattg tttcgtttct tcatatacct ccccaccaat agatagagac
150481 aaatgggaat aaccgaacca cgtctacaaa atgccagtac catgcagctg cttcaaagcc

150541 aacgtgatgc tccttggtca gatgaccaag atattggcga ataccacata tgatcaagaa
150601 aatagtacct ataatcacat gaaacccatg aaagcctgtt gctaagaaaa aggtagaacc
150661 ataaatacta tccgaaagag tggagggtgc ttgataatat tccattcctt gaaagcccgt
150721 gaatactaga gccagtgaaa cggtagctac taaagcgtaa actgctcgtt tttccttccc
150781 cgcgagtata gcatgatgag cccaagttac ggcagctccg gatgaaggga gaataagggt
150841 attaagaaaa gggatttccc gaggatctaa aacccaaatc ccttttgggg gccaaatacc
150901 tccgatttct accgtaggtg ccaaagagga atgagaagaa gcccaaaaaa gagcaaaaag
150961 gaacataacc tccgatacga taaacagaat aaaaccatat cgaggtccta attgtacgac
151021 tttggtatga tgtccttcca acgtggattc acgtagaaca tcgcgccacc atacgaacat
151081 agtatatagg ataaatatga ggcccaaact gagaagtgtt gcaccccctt gaaatgggtg
151141 catgtacatc acacctccta cggtggttgc caaagctccg agtgaacccg aaataggcca
151201 tggacttgga tctaccaaat gataagaatg cctctgagat tcaatcataa actactttgc
151261 ctcggttgta tgtaaacccc ccccttcacc cccaccccct aaagtggtaa agaagggctc
151321 tttggggtct aattttcttt ctatctgaca ggacaaacaa ataggaaggg atggttcttt
151381 cattgcattg atagaagtct aactagaaaa agatctctct attactttga gaagagaatc
151441 gttggtttga ccgacggact acgtgggaaa tacgagatct aggcaagcat gggagataga
151501 ggaactggct caaaaaacat agtggatcaa atcgagaaag ttgtgaagtt cctcgaataa
151561 ccaactatcg aatccccgca aaaaaacatc gtaataggtt tcttgcagat gacccgggag
151621 ctgaagagcc tcatctccca tgacagcgcg aataagatcg gggacgttcc attgaggtgg
151681 aatcgtctcc tcgataagca aatcggccat cctgtcacaa atggcctgga ataagcggtc
151741 ttgctcgggg ggcgcacttg ccgcgtcgga caaagtagac gagccccccg aactaaagga
151801 ccgcaaggaa aaggaattct caccagagaa aaaaaggaga ggtcagcact tgttggatcg
151861 ttccaaagga gaatgtggtt ctcgctaggc atctttcttt cgaacaaagg tttccctcca
151921 gacagaaaga gagtccttcc tgtacgagta gtgaagaagt atactttttt tggttgtttt
151981 ccaagaaagg catgggagat agatgtacaa acaaaagagg aaacgaaact ggaacatcat
152041 aacaacgaat taataagaat caatatcaac tacaacgacc gagacctaca ccgacgaaga
152101 tactgtactg aatgactcga tcgatcgtac tagatagtct cagatagacc ataaaccttt
152161 catacatacg aaatttaacg atctttttca ttaatgcgca agagaaagcg atccatcaga
152221 ctagagggaa gttggtcttt ctcaacgtgg tgtatagcac gaaaacccat tcacaaggtt
152281 tcgctataat caagtgtcaa tggcgtggca cgcgtacttg atcgaggccc gtgagactga
152341 aagaaggatt ttattaccag aaagatggtt atggaaagcc tatcatgaca tcgtttatcg
152401 cggttcatgt gtggctgctc gctcttgcct ttccttgtcc aactgccagc gattgagaga
152461 aagatttatc ctagtaaatg caaccgaact cctctgttta tggtagaaaa caaacgtcct
152521 tctcgtgaac ctacatgact tggagcttaa tctcgatctc aatctagaca ttaagtgccc
152581 ggtcttcagt cttcaatcga ttgcaccgtc ttgatcaagt aattcagaga tgaaactaga
152641 gtcttctttc ttgctaggcg gattaatgtt gtcaatagta tgtttcacaa atcattaatc
152701 ttattcttac ttactgaaac tccttcgctt ttggctcttc tcgggaaggg ctggtctgct
152761 tgggtagctc aattccaaag cgtagctgaa gcaccttttt ggggctaggc ataaaagctt
152821 tcgcggaata ggaatagcga atgcaggcac aagagaaagg gtaatccttt gttgaatgca
152881 ttacatgctt agtagcagtt cctcgatcgc aagccgctac atgttctccc cttgcccttg
152941 aacgatagaa gaactactta tcttctctta ggtagcagtc gatcggttcg catctatggt
153001 caatcaatag gtccgcggat ctattcttct atacgataaa tcgccatctc gtagcaccac
153061 cacgacaccg acaaaacccc cgatttcccg acagttaggt agccgatagt agtataagct
153121 ctaaggccgc tattcctgac aaggaataga gtaaggcctt ggttcaactc ctagctcgaa

153181 aggctgccaa ctccttactt agcttagagt cccaaaacaa agggtgttat aagcttgaag
153241 ctaagaagct actcgtttat gagtaggagt tcccacctgt cttgttcaaa actatggaac
153301 tcatctgaaa agtagatttg ctagatcaga acgtgaacta tgagaatagg ggtatacaag
153361 ttaaccactg aagtgtcttt tacactaact ttcctacaaa atgtaaccat aaaaagttta
153421 ttatattgat ttgttggtag tctgtaaaaa agaaataaat gaaaatacca tgatttatgg
153481 accctaaagt tgagtagcta aattggattc gtaggatcaa gcacaaaacc tatgagccat
153541 gaaaggccct gttatcataa atgaatgatt gggcctttag tacatgaatt tcattaggag
153601 aatgtagtac atagatatac atgaagcatc aatttttcat ttttcttgta aaacataagt
153661 catgcatttt gtagataaaa tacaaagtgg aagtccaaga aatgggggaa ttataagtag
153721 cgcttactat atatgtatac cttgcttgaa gggaagtgca caacctatcc ccaaaatcca
153781 taacttggta accaggcaaa tcagagccca tctccaagcc attgatacat agcaaatgtc
153841 caaagccatt agaatggaaa acaccatgta aaatatgccc ctgcatttcc attattaagt
153901 tcaacttacc cattactttc tcgattcccg cgccattgct gttcccttca tagctcaaac
153961 atgcagctaa tgtgtccttt gatggcacca aaaaatgata cttcttattg catattatat
154021 gctctcccca ccctatgtac atataatgaa atatttctgt tactctataa taagaaagaa
154081 acatatatat aaatgttatg atataagagt ggtagttata acatatacca atataaaggc
154141 agtgtttgca gtgtctgttg agtgaaagtt caacaggttc ttcaataaca aagagcaaga
154201 catgagacag aggattgcga tgaacttgta gttgaaatga gcagctaggc atgccactcc
154261 atgaaacagt ctctgtgttt ccaaattcta agagggcttc cacattgtgt cgaaaagagc
154321 cgtgattaaa ctctattgga tgaccttgtt cgccaaacgt cttgaatttg tacactttct
154381 ctcctgatct cttccttttc ttgcagttac tacttgcatc cgacttcgac attttgtata
154441 tctgatatgc atacaacaag ttagttagta ctatatattg aacagaagaa tttcatagat
154501 ttattttaaa gggtggttat atctctatac ctgatagatt gatcacacat aaagtgccta
154561 gagagaggcc agtagatatg ataacttcta gaaaagtatc acaattttag ggatcatatt
154621 catgggtagt atatacaaat cacacacatg tatgtgagta catatacgtg tcatccatct
154681 ttaagtagaa atagagagat aagtactaga gaggatttca gatcatagga atattgattg
154741 ctaactagct tgctcacaat gtgcatgaaa tgataataaa ataggggagg tgcaaatcta
154801 tcggacaagt gcatggaatt gacaagtaga agaagtagta aagtaatagt aacatgcttt
154861 tgagtccatt tcatttctta tgtattccgc caatttctgt ggtcagccgc ctcacgttgt
154921 gctacccctc atttttagta ctctacacac cttcatttct tgcccaccta ctccatcttt
154981 ttacttgctc catttcaatc catttataac ttcttaatca agactatcat aatatatata
155041 tatatatata taaccttata catacataca tatacctaga taatatctaa ttaagatcac
155101 taacatactt atattttgat gatttatcta atacatgtgt tagagatata tgcatatatg
155161 ctattatgaa gagtatatta aatatatcag cttacagaaa gtcataatgt ttggttgctt
155221 ggtccaacga tttcccttta aacaatataa gggtactaga tcgttaagtc atgcagtttt
155281 gcaatataag gagataagct tactggcacc tcttatatag agaatcaccc ctatactata
155341 ggcgatgaaa gcgagcttcc cttcggatag ttagcatcta gggctagtct accagttcgc
155401 ttcttagctg ctgcaactag ggataaggag tgttgtttga tctttcttac ttcacctcta
155461 gttggttggg gatacaagat tataccacta tgtggctatc tctttgagaa gaagcaaagt
155521 cctttcttca agacttttgc ctgcggatct ccttcttcaa aacatatcct tcggcgctag
155581 tctttcctgc cttaccttaa gctcgctttc acgtggcgaa tcggtttgaa gtttctgtga
155641 tcattttacg gcattcttta agcttggtca ccagacagag tgagaactgc tcagcttgct
155701 ttcttttctg gagataagtt taaatcctgg ctaactaaga gatagagaga aagaggtaga
155761 gtcgtccttt ctcttcctaa cctttgtaga ttcgccccga aaacagactt agaattgcat

155821 gtgtgaagca tagtgctaag atcgatcggc gagggattcc cagaaaatcg ttagtttaag
155881 ggaagctgtg ctcgacgaac caccggtaca aacgaatacc gttttgaaga agagacggga
155941 gggtgcaatg aatgcgctgt agctagcctt gtcagctagt ttcatctatc tcctgtagaa
156001 agaatagtct ggaataggga ctgaaggtcc agttagatca atttatatat aaagaacttt
156061 agactattat gccctattct aagccaatct agacctagga tcatggcatc tcccctccaa
156121 cattctcact tttgaccttc tggagtgatg atggcttctt tcttttgttt ttggggggaa
156181 taaagcggcc cccttttaga ttgcggcggc agagtcggag tcggcagcct ccctttaacg
156241 attgtgctgc tattcatcac aataagaaga aaaaacatta tatagcatat tcttgccatc
156301 actggaaaac aaattgagct gtaggcatca atcaaggtaa gggaccaaga ttatatactg
156361 ctgcagaagc ggaatcgaag gggtaggttg aaaggtaagg atagcgtgat tggccgattg
156421 gttgaaaggt aaggatagcg tgattgactg gttagttgta ttagcagctg tttttgtcca
156481 cataacagca agagagaagt gttaatgtat ttggcatgaa tattatgtgt taaattgatg
156541 tagtttatga agtgtagtaa aattctattt ctgtcctaat gaaaaccatt tccattgaaa
156601 aaacattatg tgttgatttt ttgttgtatg taagaaacat aaaactcatt ttatctggaa
156661 gatgagataa atttctcatc tttgtctaga catactctct attcatatca agatctttat
156721 tcaaaccatt tctttttta caatctaggt ataagcatac tcctttggat tgcttccgtg
156781 tcctttcact agccaaatgc aacccaagac ttctatgtgg aaagttagtc ttctcaagag
156841 agtacattaa aaagtctggc accctatttt ggttacatcc aaaagcatat tttgcataca
156901 aattgaaata agcaagggta ataaagaatt tatatgagga atatgcgtgt catttcttta
156961 ctaattattc tttttacttt ctacgatttt tctcattgtt tctgagagca ttcgcggttg
157021 gatgttctca agtagcagat gttattctat gcgcgtatgt tcttcctagt agtgagagtg
157081 agcatcccat ttttgatcac ggtgtaagaa caaaggcaac tactataaag agtctatgtg
157141 gggctgaagg cggctttgct atctaagcga aggggccgcc ccctgactcg aggttgggtt
157201 cgtgcgggtc ggttcctgat aaacagcccg gcaaagaagc atattaagtg tttgggtcgg
157261 ggcgtgtcag cccatgacga ggtggttctg ccatctgtca tcctggaatg accagtggta
157321 aagcctttct agctatgata gtagggaaca gtcgtgctac tagctcgata tcaaaactac
157381 ccttcgtcta gaaattctgt tgtcgcaggt tcaaatcctt ccggttatga gacaagaacg
157441 ggtctctgat aaaaagaagc tttcagcgga tagtggcctc gatcttgcta tataaagaag
157501 tcgatttgcg gcgacacccc acgaacgagg tacgtagtgt gaagtccata aagaattcgt
157561 tggaaagacc acaatacagg cctttcaacc ttagtcagaa ggaacccaca ttagtgtaca
157621 aaagcccaac gcgtcgaaca ctaagcagag gtattgtgtt tcatatagag atgaggtatc
157681 tgcgcaaact gacgtagcag tcctattccc ttcctccccc tggctcttag tttgtgtcgt
157741 acttcggatc ttcccctacc ccacataggc tatgaacatg aaagctaaac aagcaaactg
157801 acacgttgct gtctcctctc ccccctggt tcttagttca cggggatgga gagtagcctt
157861 cctttgattg gttgagtctt taaaagaggt attgtgtttc atatagaggt ctctgagcga
157921 gcaaactaag gtagatgtct gtcctcttcc accatactct ttgcattgta ccttaacagc
157981 agctttctat agtctccctg ctaacggaca taggaacaag acaactaagc tttcactgga
158041 ttcaaatacc ttggatgagt caaggggtac acagccggta aaccaatcta tcctctcact
158101 ctttcttttc cattcccatc gggatgtgct aaacagcttt cggttttcgc tttatacttg
158161 atagtttact ttctagggca tataaagttt tgtatcatac gagagttagt gcaaggagca
158221 gcctaaggta aggcaaagaa gaagaagtag cgatatatgc cgcttctagt gactcttccg
158281 aggtacgtgg aaatctcaat cttgcgtatt ctattgaaag aagggttgca agcaagggac
158341 aatctatggc gtaccactcc tacaacagaa aagaaaggtg acatactcta ctaaatgaca
158401 aaaacgattt cctatgcttc gtaagtagga aagggagaat atagaatatc atagagaaag

158461 agatagatta cagctagagg gtgacaccct cttcttgaat gaaagtgaag caggcgccta
158521 tctaaagctg ctgcagcagt agctgaagtg ctaccgcaaa ggtctcggcg tccccgacct
158581 tggggcctag tcgcaggctt gatggacgct ctcatttcat gggaattctt cagtctatgg
158641 ctgaccggtc aggtaaagct tgcgcgagac tctaatcact ttaagagtct cttagtctct
158701 aatagtagaa gtcatcagga ctcaatatcg atcgattcgc tattccgcct acgtgaactc
158761 tgttctatgc tccctttcga gctcgctcta tctcgtggac agccggaaag aattcaacca
158821 agcgagaaca gatattagta gtcccgatgc aagctgcatg tccggggttc acaactccca
158881 agactagagg ttcaaatcca gctacttagg tcaacgtttc acatagttca actgcttctc
158941 tgccccttct ttgtccttgt tgaaggaaga gagcatacta tgaagatgca ttccggctca
159001 ctaatcaact caaaactctg ggccttcccc tgtgatttct ccgggagtta gatgttggag
159061 tcacatcgtt tccgttccgc ggtcaataca ttcttcgctc tatttcaatc atttcttttg
159121 atagatggag tacccgggca ttctcttgtc ttcccattcc tctagctctc atttcgcgca
159181 tgcgctttgc ttaaccacat ctcagaggaa ttgaacctct tcctgttctg tgaaaacgga
159241 atccatttga tgtcctcctc cttcctccgt gagtttagct gcaacggccg cagacgcaga
159301 aaacaagtgc cattctaatc taacgtccac cttttttctt tcgaacagga acttactaac
159361 ttgactcatt taagaagcac caaactcggg gttagcggca taaacgaaaa tgaatttgtg
159421 tattagaaag ataagtgtcc tagattcaaa agattcaatt ccctcggtaa ggcctaagat
159481 atgctatata gaatagatca tcttgacatt ttacacgcca cgaaaatgtg atagaaattc
159541 ggtatacaca acagaatttc tttgatattg taaaagattc tggagccatt gctccaacgt
159601 gttttgtaac atcacttttt caggataggc atgagggtgg ggattcccga gatttaacaa
159661 gtggagtctc tcttcaataa attctaaagc ttcggacctt atggcccttt cctgaaaagg
159721 ggattccctc aaaatttcga gtaggcgctc atttggttct gtcatcaaca gatcaaaaag
159781 tctatcttgc gtattcgctt ttaattcgag caccgtaatg tcgaaaagct cgttttgaag
159841 agcactttga taatgcataa tgtttcttgc ttggttgagg ttgtctctca ccaaggtttc
159901 gtattcaccg aagttcagtt gagggggtaa ccgttcgatt aagcggcttt cgagtaagcg
159961 gatccgggca tacaattcga tttccccgtc tgggtcttgg gtcccaaaaa gagacaagag
160021 gttccccccc gcttggggat tctcgggaat cataagcgaa tggggtgaat ctgaaattat
160081 gttagtggtg gaacttgagg tgccgtcgtt gctggtagat aagctctcag ctgaaacaaa
160141 aaaaagaaaa caagggaaaa tagattccga aatcgaagaa tgacctaaat gtaaaaccaa
160201 atagcatcta acgagactgc catacgcgta aataagaaag agaaaggaga gagagattgc
160261 aacttttagt aagccgtact tcatttgaaa atgaatccga cgaaactgaa caatcaaacc
160321 gataagaaca attccaatca ttattaaaaa agaaccatct tctgatccta gaaaacgtcc
160381 gaaaaacccc gcgaaaaaaa ccccgagaac tctagaaagt acagacaaaa gttgactcat
160441 atgttttccc ttgaaaaaaa tgttgtttca tcacatcaag gtgacaggat tcgaacctat
160501 ggccctctgt acccaaaaca gatgcgctga ccagactgcg ctacacctcg tcttaccacc
160561 ccggagcata tagatccacc cgatcgatga acactcaaac cgaactcgcc catccttttg
160621 ggcacaggga cgcgagaacc aatctgagta atcaaccgct ttttatctgc ctccagcaag
160681 atagggcgac gccaaaaagc cgtatagtgc aggagcgctc acattttttg gcttactctt
160741 tcactttcat tttgaaatcc ggctcgctcc cggcctttgg acccttcgcc cgcttagaag
160801 tggattcgaa ccactgacac aaggattttc agtcctttgc tctaaccagc tgagctacct
160861 gaaccacctt cctaaagtat gttttcttcg ttgaatagcg ccctacctta ccacttgact
160921 agtaagggaa aagcccttta ctaaaaaaaa gagaattgat aggctttttc gcttgttcgt
160981 caagctttcg agcagctctt tgccgcctaa tcccccaacc atgctcaaga accgagagcc
161041 gtccagggac tggactccac caagaggttc tttctctcac gtaatttcgc ccgcccgttt

161101 cacttccgtg ccggaggaaa tgggattcga acccatgata caatcttctt gtatgtcgat
161161 ttagcaaacc aatgccttaa gccactcagc catacctcca agttgttgat cggaattgaa
161221 tttgatgtgc cgggtttggt tggttgcccg aagggctatc tgatccgatc aactgcgtaa
161281 gccgtagcgc gcgtactctt cctctatcta tagagcgaga ctccacccaa atctgccatt
161341 cgttgggcga cgccttcttt tctatgaaca ccccaccgct gaatgatgaa ctttgcaagt
161401 tttcgcctcc gacccgacca ggggggaaca cacggtcaag ccaagccctt acccgcctga
161461 atggaatgaa tatctccttc gtctggtcga gaagggagaa agcccgggga actggactgt
161521 gactcttcta ttatattacg aagaagtcca ttctcgtcat gatcgatcca cgtcctaccg
161581 tagtgcccgg agaaccaggc ttgcttggct tacaaagcta gcttactaac gcgaaggatt
161641 ttttcgttga ttgcacgagc tagccaacaa ccccccccct actcacctct cactctataa
161701 aaagcccttt ctccttttaa ctaggagaga aagctccgcg agctgcccct ttactctata
161761 gaaatggatg gaaatgcccg ccctgatcaa tgcgaaattg atcgagatga gatcatgatt
161821 tgattggaaa tgcgtaagta agaggagatg ggagtaatcg ggcgttctat atactatgat
161881 tcaccttttt catttctttc attttataga aactagtgat gtacttcttt agcctttcta
161941 gtgaagttca ctcctttggt attgaaacct ccttatcttc ttttagcagc agaaaattcc
162001 ttcctccttg ctgctacaac gcgcggttgc tattagctta tttatttact tgaggcatag
162061 gcgggatagg gctttgcttt agcatagggc gaaaaagaag ctctataagc cgaaattgag
162121 tacccgtaaa cccttgagtt ccttttcttt acatagggta gggggcggct ttgaagctat
162181 agaaaaaaag gctacttact ttgcgatagg agcttgcgcg tgggcccaag ctctatagga
162241 aaaaaatagc ataggcatcg cagcggctac tcttttgcgc taggagttat gaatttttcg
162301 tttagttttt ctttcattta ggagtgaaaa ggaacttatg agatgaacgt aatctatatc
162361 tctcctttct ttggttagga acaattcaat agcttgcctt aacatcgaat aacatagcga
162421 gcggctacta gcttgctgcg gctcttacta tgagcttagg ttccatagct ttaggagcct
162481 tcaacaaaaa tagaatagct ttgcttagcg gcgactactt tctttctagt agctgcttcg
162541 ctttaggagc catttggtat agaaccacat agcttgcttt caggcatagc acatatttga
162601 ttgatactca agtgctttct cgcgagagca tggagcttgc ggtcttagtt tcaaagaaaa
162661 gctttctttc atcgaaatag tcagcgcagt ccgcagatag agaagagtta gtttagggac
162721 tggtagagta gtcaagtagg cagtcagcct agataaacct atcaagaagg ggaggcccat
162781 atctattcag agtaagcttg gcgggtgtca ccggtttttc aattattttc ttccgttgaa
162841 gactgtagtg tatggccaaa atggcatctt taaagctgct atgaactcag cgtaactacc
162901 ccttcgcagg caggtaactt atctccagta tttggtcttt cgtatacccc tgttttcggc
162961 ctgtttgacg aaattccctt tgactttcaa aagtagaggg cgattagcca atactcattc
163021 tgtagcctat ttttttacaa tgaaagcaga ctggcccgaa cggaattttc gtatcacaca
163081 gcggatgcaa gggcccttcg gaacttgtat aactatatta cctcctatca tagaaaatat
163141 tctacgtggg cagacgagat caactcgcac tggctctttt ctgtgagccg ccccgatccc
163201 aagccataag tcaatagggc ttcctttttc tttcaaaaat aggaacgcaa agagagagta
163261 gcgattctct ctttgcctgg cttattagcg gaaatatggc attaactacc cgatacgcta
163321 cttcacacct ttcgttccgt tgccacttca ctcgttcgtt gccataagct gattggaatg
163381 gcaactaaac aaccataata caagcaggag atatgccaga caactcagcc aggtgatcct
163441 agatgtttgt ttccacacac atccccccaat agtagaggtg cacacaatgg tgattttggt
163501 cttataaaat acatccataa taatatttta aatttaatgt ataaaaattt tttttcagtt
163561 tgtaaattat atctctttaa atttttattt ttaaaatcga aattgacacc ttttaaatat
163621 cgtataatat atattttata attcaaatta aacgaaaata tcttaaatta tcatattaaa
163681 ctaccttttg atcatcaatc tgtgataaag aaggaaaagg ttgaggccac gtagctagcc

163741 ccgggcgaag gcttagtcgg aagacttaag ttagtacctc ggagacggac agaatcgcct
163801 ggcgaaacta ggcgtagatt accgaagtac ttcaatcgca aatcgatgtt tagaagaaaa
163861 gcatagccta gcaggaaaaa aaaagagttc agcaagaagt gaagcttctg cttcaagttg
163921 ctgttcttgt cgttttcata ggagcgattc ttttccatag cgtaaaacgc taagcaagtc
163981 aactccctcg gtgtatcagt cctgtctgtt tactaaagtc aatgtcaaac tcgatccgat
164041 ggtaggtgga ggtgagaagc ccattctgac aaggagaagg aaatcgtact gctctttcgt
164101 gatagccgtt gaacgacaat cctcaagatt aggtagttgg taaggaggaa tagagaaggc
164161 agtttaccta ctgccgagat gcaccattag ggtttcatgg catagattaa aggacatcta
164221 agaaggtatt ctctttatac aatatagcta ccagcggtgc caatcaaccg agccgtttcg
164281 ttcgtatttg tgtccgggaa agactccctt cactgggaaa gagaaaggaa gctactttag
164341 ctccatgcta ccaacaacca gaggagaaat agcattcaag aaaggcgact tatgagggat
164401 tctctttcat taaggtagct acacacagta gttccggacc ggagagattc tattacaggt
164461 agctacatac ggttatttcc cggaattgcc caaccgctat tatggttgat tggcacatcg
164521 tggaatcatc atagagaagt agattacgcg accccgaatc ctgctattcc ttcttattga
164581 tagcacagga aagagaactc ctagctctag gactagggat attcttcgca tctcgaaaga
164641 aatgtatctg gtctggtctc taggggagtc attcctcaaa tgtcattgct agcaagagca
164701 gagagatcag aagcaacggc attaccaccg ggctattgga gctgttaatg caattgaatc
164761 agaaaatgct cttactgttg gagaataccc tgcaataaga cttgtttggc atcccgtatt
164821 tccggagtca aataggctag gcctcctcgc acatagaatg gaagaatccc ctgttgacgg
164881 aataagagct gttatataga atgcgcctta ccttcggact ttactattga ttaagggctt
164941 tgatgggcaa atgccaatag ttcagatatc cacgagcaca gaataggaga tggggctcat
165001 tttcacgtga agaagaatag gctcaggcta agggcgagag aaagattgaa ttcacagaga
165061 agtcaggcaa tgaagtcgct agcctagcta gatgtcacct tttcttttag gtagtcgatg
165121 ttcgaaagtg aactgtccgg gttgtggaca aatacgttct ttgcgacgaa gggtctgatc
165181 cctagccttc agcggacgat cctatcagtt agataatcga agtccatacc ggacattacg
165241 cattagactg attggaccac ttaataggac ccctttggct tggctattct aggccttctt
165301 ctattcgtac cagccgtttc taccccgcca ttaaagcttt ctggtccggg gccatctctc
165361 aggcttacac aagtctaatt cttccgctac cgatattggg atctgagcct tccttgattg
165421 aaagaatcgc aaacaaagag taaattgctt atgaatatta ttaggtagca aacaggaaaa
165481 gcaaacaaaa gggctcgcat tctctttctc gaagacttcg tgaatggaat ccaccaactt
165541 gtaagtagaa atcagggcgg gtagctttct tgctagttga aggtctcttt tggttagcct
165601 aaaagtctta cttctattta gtcctgatat tctagctatg acaataccaa tctcacaccg
165661 taaaggagtg tgaagctcat gatcagataa tatcgtaatc aaattcgtgc acaatccaca
165721 cttcagtgta gtcaagaaag ctaaccatca tactatactg ctggctgcag cggatatgcg
165781 acatcccttc ccttctttct atctttcttt ttagaaaaag agtgaacgtg aaaagacccg
165841 atcccatatc atagcaggag gctcgcgctc agcgtcaggt attcacacaa acccttcatg
165901 acttatcggc tgccttgctt gctacgcgtc gtgcaaaggc tcaggcgaga ctgaatcctt
165961 cttcctcttc cgcaccttct gggagtagta gtgagtccaa tgacaaacac gactccgttc
166021 tcattgaggg gttcaaaaga aaaatagctt gtgcgattaa agaactcctg ccgcaccgcc
166081 aggaaagcat tcgccggtgg gagaacacac tgaacgagct atacccccat tcgggatcgg
166141 agacaaaaga gtttttattc gcaaaattct acgaattaag aacagatggc caagacagtt
166201 tctttctaaa agagatctta actttccaag atctcgctaa tctcattcgt taactatctt
166261 ttttctatag aatgaataaa gaaaaaaaaa aagaggaaaa gaaggtaaga taagggaggt
166321 aggggttgaa gaaggtccca agggttcggt tgttcgccga ttcaagtggt acgtgagttg

166381 ggtgataata ggtcgttttt catttgaatt tctctgacat tctacgttcc tcaaacatga
166441 acaactcttt gccagatttc ctcataactg cttgggattc cttaatgaga aacccagaga
166501 ttgtactcgc accttttgtt ttggtagtgt cggtgtggag aattgtccgc ctgcgggaca
166561 ttagtgtcga aaatgtggat tctcgtttga atgatactgt atgtaagaca gtaatcgaat
166621 atacatcaaa aaagattgcc gccttacttg atcaagagca ccttatctta ccgcacgggg
166681 tacatcctgc ggacatcgtg gagaggatgg ttgcccatga cccccagaat ctggtcttcc
166741 tttctcaact gtacaacagt ttatccgaga aaggggtaca aagtgaattt ttccaacaag
166801 cagtggcgat tgcactgcaa ttcatcgggg ggggggaggt tcaaaggaac gaggggaaga
166861 atcgacgagg aatctatttg ggacagatag ttcacagcgc tcattctata gatacttgaa
166921 aagctaactc atgtttccac tccattttca ttacgaagat gtatcacgtc aggatccgtt
166981 gctcaaaccg aatcacgcca acgttatgga agttcctgga tcgtgtaaaa taagagtagt
167041 accaaaggca gcaccttatg atttcataat caaaaatgga aaattggcta tggagattcc
167101 gcgcggtcag aaattaatac agaaaaaaag ggcttcgaca ggaaagtcgt ttcgatccaa
167161 tccattcttg gggtcaaata aagggtctgt cagtgaccta gcacgacaaa gcactctccg
167221 agggcatgga atgtatcatt ttttgttcag aatctccaca gtaatgtctc tgttagattc
167281 tccggtcgaa atacgggaaa actccattca attctcgatg gaaacggagt tttgcgaatt
167341 atccccggaa ctggaagagc atttcgagat cttcgaacat attcgagggt tcaacgtgac
167401 tattgtaact tcggccaaca cacaagatga tactttacca ctgtggagcg gctttttgca
167461 aaaagaggag ggggaaagta agagtaagta agatgtcgga gaagcgaaat atacgagatc
167521 acaaacggag attgctcgcg gctaaatatg aattgagacg aaagctttat aaagcctttt
167581 gtaaagatcc cgatctttct agtgagatgc gggacaaaca tcgttataag ttgtccaagt
167641 tgccaagaaa tagttcctta gcacgagtaa gaaaccgatg tatttccacg ggccgccctc
167701 gttccgtata tgagttattc cgaatttctc gtatcgtttt tcgtggatta gcatctcgag
167761 gtcctttgat gggcataaag aaagcgtctt ggtagcccaa aacctatttc tttcctctat
167821 ctctttgggg cagatagctc acagtgctca ttctacagag acttcttttt ttttagtatg
167881 agcatttcct ttgtgctctt ttgtatcttg ggcgactgct attgaaaaga cttttctttt
167941 tattcctctc ttgtttgctt tgggactggg ccttgacttg gggggtctat ttctttggtc
168001 catttgtgta ctttatatat atttcggcca tggaaggatt tcatctcccc gtcggaggcg
168061 gtgtcggctc gtctattccg gatctcaacg cctgaggctc ccctccctca accggctgaa
168121 gacgtctatc acgaaaaaga tcctgatcgc ataaaagcca ttcttgagcg gaaaaaggca
168181 gagaaagatg cttcggcggt ctcaaatagg aaggccgaga tcctcactca aataaaagag
168241 attgtagagg aaactttggg tgggaatcat ccgaaaaaag aagaatgcat ctcggaaatc
168301 aataataatt ttctcaagag aattcttaaa aaaaaggtgg aaaaggcggc cttaattaaa
168361 aaggggggttg aacaaaaaaa atagggacag gggggggcag ctatatttcg aacaatgaga
168421 cgatcacgga ttttctcaag aactactttt tggggatttc tgaaaggtta aatccaagtg
168481 ctaaagcacg aaagccccat tactgatgga caaatctgtt tggacaagag atcgctcggg
168541 gtaaagccga aaggtgcgat gacatcgaat gcgccgcgga ttttgtagtg cgagaggttg
168601 aggagaaagc ggagaagaac caattcccat ttctcgctcg cttgaaagac ggcctggggt
168661 cttatcccga gatagcaagg tctggcgtct gatcaaaaga taagtaaatc ggtggaaggg
168721 agggggatgg gagccttagc ccttctaatt gaatgaatca aattataaac cggcgtggcg
168781 ctgctagatg cttctgatca atagaacagg aagaggagtc agcaaaaaaa aaagaaagac
168841 caccaaaagt aacgaccacc aagatacgca tagtgattcg atcttttgat cacccatttt
168901 ttgaaaaccc tttttggggg cttccgcctt acacacggaa gattggattg cctgaatcac
168961 gagtcttata tactgtgtta cgatcacctc atattgataa aaagtccaga gaacaatttg

169021 aaatggaaat caagaaacaa tttctggtca taaaaacaga aaggcatgaa ttgcgcaaga
169081 agttctttcg gttaaaacgc cgtgcgactc ggaggacata agacttcttg gtcaaaccaa
169141 aaaaaaagat tgccgactgg atgctcctac tccaccatgc ctggcccttt accttacctt
169201 aagaagaaag aggggggtat gaagcgtggg aaagaatgca agaagtgtat gatacaacag
169261 gtaaccttaa ggagtggcgg caaacctctg gattgatcaa cgcgagtgaa ctgtgcttag
169321 acgcttcgta aaaccgcacc gatctacgag aggatgagca ggcttcccct ttcgattcac
169381 ggaatgtgat ctgggacacg atgggagttt gcgtgcctcg gtaggaaaga gatcaccgga
169441 gtatagcaca agatcgccct ttcttgctgc catggggtcg acctgtgaac aaggtaaacc
169501 caatggggac aaaaaaacgg gggtaccgca ttgggcagaa gacaatccaa aaagcgaagg
169561 ctcacccagc tgaaggaagg agctttggaa gcttacctta ttatgaaaag gggaaagggg
169621 gtgagatagg caacgggtag cttgcttcca agccggcccg gccgcgagga agcaagagat
169681 ctttgccggt gctgacttgg atctcgggtg acggaataag gcggccagaa gcgacgagca
169741 gtcgcggtcg aggcttggct ttagccgata ggctagcagt aagcttggct acagcgaagc
169801 gcttaccaag cgcgaaggaa agggccttcg ccacttgagc cgtatgcggg ggaactcgca
169861 cgtgcggttc ttagggggggg agagctagta ggagccatcc catcccaata gcgtatattt
169921 ggagctcaat atgaaatcct attttcttgc aagacccgtt cggataaggg aaaacagcgc
169981 ctttttcaaa agggcgctag agcttactaa agaagcctac tcaaaaccgg atacgtgagt
170041 tgggtgataa tcgatcgtca caagtcagaa ttgcctcaac aaaaatagat ggaattgcac
170101 gtgtagaata ggtgcgaaac ttccttaagc agtcgtcgct caaagcgggc tcatgtagga
170161 agatcttttt ttgctttttg attattctta tgaggtcgtg tacaacaacc ttaaccgcac
170221 aagcctgggc tgggcctacc tccatcccta gaggagccgt atgaggcgga agctccacgt
170281 acggttttga agccgagcct ttccagcaat ggggcctagg gaccgatatg atgattggtt
170341 taggtagggc ggccggccta ctacgggcac ctgtagggat tagtgtgtga gaccgcgatc
170401 cacaaactga cgcatgggac tcacccttta cttgggaata gagaggggaa acatagcatg
170461 tcacaagaac gaggcgaggt ttggaaccct actgcgagag gggcgcctcg cgagccgggc
170521 ttttagagat gaggcctttt ggcgaagcca agtcaatttc gggccaccaa accctgcaac
170581 tgatgagaag gccctatgga gtaaagggaa gcgtgtacgt tgtcacactc tctgccttcc
170641 aaaggtgcct agaggacggg ccagacgcag cagagcgacg acccgggagc ggattcccca
170701 ccggcagggg gacaggagac ggccatctca aggcacatca cgacctacag gcaacaccgg
170761 cgagacctgg gaaggcaacc cgattgggag tcagaggatc catagtacct gcagcctccc
170821 ggacttcata ttcataattt tttaaagcgg ggggaaggga tcttttttct gcaacggaaa
170881 aaaacggagc agatttgact cggcacaacc taacgataca tccaatacca atgatctgtg
170941 cctagaatgc cttgctagat ctctgttcta gaaagctata caggcaacaa cgaacgcggt
171001 ttgacccggc ccttcccttt cattacttct ttcacgacga gagaaccgcg cccgcatcag
171061 tcgaagtggt ggaaggcccc ctgagtcaaa gggagagctt ctgcaactct ctccaagaga
171121 tacgattcgg tatctgaatc ggctgaagga gagtgaagag gcggctactc tcccagcccg
171181 cgggaaagcc tcttctttct ggtctacatc aagtgcattg aatgccccac aatcagaatc
171241 aaagaggatg ggtcaagggg aggttcggaa cgaaaccaga gtggatctgc ggcaaagcgg
171301 agcctccgga ggtatggcgg cttgtttgta ctgtgccgtc atttcggatt cccgaccgat
171361 ttcggcacta gttaaatggg gtgaatcgcc cagatcttga atcaactagt agatcatcaa
171421 cacagggccc aattcattct ccagatgagt tcggagctgg ggcagcccgc gcgcgactca
171481 taccaatcct acacttcttt ctactattgt ttctttgtca cttcttggaa atcacgaaga
171541 ggatttccgg actggactga gcgggagggg ggaggacttc tttttcattc tatatagggg
171601 gagctattgt gaagcctaga aaggcggaag cggcaaatcg cttctaccga gttccccaac

171661 agtagcttag cttagtaggt agcttaattc tattggcgtg gcgctgctgg atgcttctga
171721 tcaataggaa gaggaggaaa agaaatagct tatgatgacc atctatttga gtcgatcatt
171781 tccaagatct aattcaagtt ttttcttatg tagtggaaac gccttaaaat ctgaagtttt
171841 acgcttaagg gaagaaattt tattgatgga cgcaggacct gggaccccca gaatttgtat
171901 gcaagatgag cttacaggag tgtcaatcaa ccgagccacc aggtttgaga ataaggtggg
171961 atccctggat ctagtggccg gtgaatcact gatcaaagag catattttgg agagattctt
172021 catcgatcta gtggccggcg aatcactgat caaagagcga gcagccgcca ggtttaagaa
172081 attggtggga tccacaaatg tagtggctgg tgaaccgctt cttcttcttc cacgaagatt
172141 cagaaaaaac cgagcttgga tggaactgaa caagatttgg cgaacgaata caaaggtaaa
172201 aggccttatt tgtcgtaaaa tcaaaggagg ttattcagta gccatcgcag gctttcttac
172261 ttttctgcct ttcaggcgct ctcgtaaaag gaaatctttt cctcgattca ccattgagag
172321 cattaacccc aaaaaggtgg ttactattgt ggtgttctaa cagcggcagt caatgggtgc
172381 cagagctgct acaattgctc agcaggagtc ttcttatcag tcgccggacg ccaatgtcaa
172441 cgccccttaat ttcataaagg attacgtaac ttcattgttc cgtaccatcg taattcttct
172501 aattggggtg acgacatgaa cgagcacttc ttcttcgacc tagtccgtcg cgacccataa
172561 gggggctttc gttcccaaca aggtagctgt agggataacc tatgggctgg attgaatcct
172621 tctttttccg gtatgccgct ccgcgagcaa ggagcgatag aaccaagtgg gctgtggtga
172681 tgtcagaatt tgcacctatt tgtatctatt tagtgatcag tccgctagtt tctttgatcc
172741 cactcggtgt tccttttcta tttgcttcca atagttcgac ctatccagac aaattgtcgg
172801 cctacgaatg tggtttcgat ccttccggtg atgccagaag tcgttttgat atacgatttt
172861 atcttgtttc aattttattt attatccctg acccggaagt aacctttttcc tttccttggg
172921 cagtacctcc caacaagatt gatccgtttg gatcttggtc catgatggcc tttttattga
172981 ttttgacgat tggatctctc tatgaatgga aaagggggtgc tttggatcgg gagtaaccac
173041 tagtgagagg gcaaaaatag gggggaaata caaaggaaag agcgatgcct acattaaatc
173101 aattgattcg tcatggtaga gaagaaaaac ggcgcacgga ccgtactcga gcttcggatc
173161 aatgtcccca gaagcaagga gtacgcccgc gtgtaccaac gagaacaccg aaaaaaccta
173221 attcagctcc acgtaagata gccaaagtac ggttgagcaa tcgacatgat atatttgctc
173281 acattccagg cgaaggtcat aattcgcagg aacattctat ggtcttaata agaggaggta
173341 gagtgaaaga ttcgccaggt gtgaaatccc attgtattcg aggagtcaag gatttgctgg
173401 gaattccgga tcgaagaaga ggcagatcca aatatggtgc agaaaaaccc aaatcgatat
173461 gaatggaaga tgcctctttc acttttttc tcggtcagtc gagggaacaa ccacaacatt
173521 acgccacaaa agaaaactat acggagccct tccgaatgac ctataatata gtttactaca
173581 ctagccaaga aagagtgtag tagactatat tcgcccgtgg agaaagaatt aaaaaaatag
173641 aaaggtattg cttctaatag ttgctcgttc ataaattcac tcgaccactt gttagtcttg
173701 ctacactaca cgacacgagt ctagggtaaa gttgaagcag acacggtcaa gtgaagtcga
173761 cttcacaagt gattcaacat accatattta aataataaag aagagaaggt ctcgcccagg
173821 cggctcccgc aaggtaacga cttcaaactc aaacaaagaa cgcccttctc caaggtatga
173881 gtcaaagaaa atgctgtata taatgcaaga cccgtcgata agctaaggct acgacatgaa
173941 ggaaggccag aagaactaca gaaccacgcc caccagagct aaaggagacc gaagggagtt
174001 gcgggaggaa accgaaagat aggtaatcca tatggtgaag catgagaaag agggattgct
174061 tcaaaccgga agaaatcgag cccacccaca cttgagtgag ctatcaggct ccacacattc
174121 gtcggccaac cacttcgttc ggaaaagaaa gaaaaaggcg aacattctct ggcttcattg
174181 aatgtcacac ttttgaaagc gagtcaagca ttccaatcct agcggatgag tcaatagcag
174241 cagagtcatg aaaagggcta aaagcagcct ttctatacat acgaaaccac cagatttgcg

174301 tatgaaacaa gagcagcttc gtctgtaaca gcttattcaa cctcccttcg agaagtaaga
174361 gcaattggcc cttgccttga gccagttcca gtcatgggag gacctttccc ttactctttt
174421 aatgaaatat cttttttacc tgcctgggat accatgaatc ggtagcgtag atctttcttc
174481 tccccgataa gggcttattc tgccaagact agctgcgtct ttttcgccta gtgagttgtt
174541 gccgtcactg aaagaccttt cgagtcgaac tatagtggat aaaatagctg cttattatta
174601 ttaaacggat ccatcttctt tcgctcctaa atgaaagaaa gaaaaaggct tttgtcggct
174661 ctgaggctga ggaaagccgc tgaccaaaac ctagtcacaa aaggactact tcagtctatt
174721 cgttcgcacc ccttgcgaag cggaagaaag ggaaaagtga gttgcaagtc gagaaagaga
174781 gagagaactg cctaaaagga gaaggaatca aattagtcct cgtaaagcct aaggtaagga
174841 aggcagttca ccaggtaagg cctcccatcc gctagttcgg tcgttaggtc gagaagaaag
174901 ggactccttt gaaagaaagg gaacgagtgg agtatacgaa gctaaaaggg aaggtaggag
174961 ggggtaaggt aaagttgaga taggatcgta aaccgccgcc cttccgcatc cgattgatca
175021 aacggcctca tcaacaataa ggtggacttc aattgacgcg ataaggtgtt agaaacaccc
175081 tgacgggcca gggtatgccg ttggttggag ggctactgaa catcattctc taagaaatca
175141 gccccatgat atcatcccgt tgcacccctc acgcaacgtt agagggctgg gtcccctccc
175201 aagtcaaaga aaaacaacaa caacgaaatg gtctatgcct cacgatcaat tctcatccat
175261 tctacgactc atctcatcgg atgagtacca tctatcgcca ctaacctatc ttgttatacc
175321 ggagtatgct ttcaaagagt atcttaccga agtcttgcta ccagaagatc ccgatctaat
175381 gattcgaaaa tgtcgccata ggagtggtaa tatagaagtt cttttacctc ataaggatga
175441 tgtagtggtc tacacaggat tagcacgtct cttatttcgt ctctctcgcg gtagcttacc
175501 tacaaatgac gattacctaa tattgaatag atcagacatc gaatgttttt ataagggtct
175561 tctaggtaga agtgttcgaa ggttgtattg tatagaatta gatgaatcat attgaatttc
175621 attccaaaaa gtcttctatt agagaagttt aagactctta ttcttgatag atctgtctat
175681 aggctcatat cagagttgat ggacctcgat atcttttatt atgacttgta tcatacaccc
175741 tccccttatg gcataccaac tataggtgaa atgtgcatag tcttattcaa tctcttctta
175801 gccgaaagct ttgatcgtgg gtttgatcgc aagtatccgg gtattgagtc ttcgaggtat
175861 tttgacaccg ttttggcagt tttaaagata ggattacgag gcatggaagc tttctgctat
175921 gctattagag gagagttact gtaaacacac aacttccacc ccgttggctg tagttttagc
175981 ttgtatgtgt gagaaattaa caagaaaaaa agatttttct gcacaaaaca gggaaagaga
176041 atgaactaga acagagcaga tttttattct tcttcattcc agcactacaa gcaagttttt
176101 atacatgaga gtacatccaa taggaagctt acacacacat ggaccagcca cacaactaaa
176161 cacaacataa gaattcgtag atctccacca aacaaagaca aagaacacca tacattaaaa
176221 cattaaaata cactactttg cgtctacaga cacttattta aatcttctag aaagagtcga
176281 cggactcttc tagtctagcc tccacgatca atggcagcct gcacaatgag cgcttgctgt
176341 tcttctacaa gcgccctcct cctgtcttct agaccgcgaa tgcggtccct tatttgttgc
176401 atcaacccgc ctataacctg agggttgaga gcaggcggat gctcccaaaa gagacccagc
176461 ctttgctccc attcgagctt ttcctcttcc acctgagaga tttcttgtga aataagcgcc
176521 agtctttcat ttccatcggg atccatggct actaacttta ttgccgactt ctaagtgccc
176581 tttgccaaat agagactgaa agaagcttct atttataggc gttggaggcc cttaactctt
176641 tattcttatt agtaaccctc ttatttatta gattcgtatt tttaatatag gttgtcgtaa
176701 catggatcat ttcataacat aatggttttt catgaatatt taaccccata cactatcttt
176761 tagtgcgctg aaaaattgtc ctttccctgt tcggatttga gtacgaaagg cccaccaaca
176821 aaaatgatct caatagtgga agttcctatg ttttgccacg cggctaaccc cggcccctac
176881 cctaaagaat aggacgcaat aatagagcgg aataggacaa taatagagcg aagcgtcaga

176941 tagtactcca ataataaggc gccaaagccc ctttcataag agatagagca atcctcactc
177001 gacacctcaa agctgaccag tacaaaaaca atgtgatccg tcctcgttta cgtacccca
177061 cttgcttcta gttgtctttg actggcttat ttccagctac atgatggaac tcccccccta
177121 tcacttaaag gcaatcgtca cttgccagcc cctatctaaa ctaaaggcct tgcttagaat
177181 tagaaaaaaa gatgtttggc ttcaccccta tctcgtgatt gatcagacgc ttgctttttc
177241 ccgcgtgctt gttctctttc tattatttct accactatag atttggagcc aatcaggtag
177301 caccgcgtgt caaactgtgt caggcgggag acagaaggaa aaagtattct atcagggtca
177361 ttttaaaatg tcttttttat cgtacaagaa acgaattgaa tttgtgtatg tgctcccgag
177421 aaacatatgg tactctattc cattacatgg atcgggagca atcgaaaaac cagacccagt
177481 ctctcttgaa atcccgaata tgccaataat tctattgggg agagcttgct agtgtagtcg
177541 attgctttgg gaaggtatac gaacgagaaa gcgtatgact cttccgaggt atgcacaaga
177601 cactcgacca ggcaataacc cgtgttatat gcatcttcaa aatagcaatc aaactagtgc
177661 tatgcctgaa agctctaagc aagtgactct tcccgggtga aggagagatt gtcgtactta
177721 tgggatccaa agtgactagc cttggtctgg gagtccttca aatagaattg catagtagaa
177781 gaaaagtata ggagcagatc ttggatcgag gaagcaaggc ccaatgtgcg ccccactata
177841 ggtctcgaaa tgaacctgga gaagccggta aaccttgaat aagattctat ctctactttt
177901 gcttggattc tgaacgatag ctatatgctt aacacatgca agtcgaacgt tgttttcggg
177961 gagctagaca gtctatttag ataatggata ttatcatatt gattttcaat ttaaccgacg
178021 tgataacaaa ataaaaaaaa gagccagaaa ttttgaacat gaatatgttt ttttttgctg
178081 aaaatgttat atggaatgga tgtagtatta taaacaccat tgcacagcat acaagaaaga
178141 taaagaaatg taggcataga tatatatata tagtcatata tagaatatcg gaggggcggg
178201 taaatgaggc aggccagttg gaggagtgta gtacgaagca gaggtgataa aaatgcataa
178261 gtagaagaat cccatgacag aatactacta gtatatcaaa ctgttggcat atatggctct
178321 gaaactaaat ctctttctct cctctgccct tagattttca tgtctcctct tcctctctgt
178381 ctctctatct ataaattaat acctacacac acatatacac acactcacat cctctcttcc
178441 tttttgtata ctctgaatgt tatatatctg ttttatgtaa tttacattag tattatgtag
178501 ttttgcatga aatatctcta tgtataaaca tagtgattga atgtgtgttt aggattaatt
178561 atgaagaagt taattaagga gctttcttca gtccagtccg aggcggtttg agagctgata
178621 acacaactgc tgactcgttt gttcatgaac acatgcatca tcagataaaa aaggatgtgt
178681 ttttgatcga acgatgcagg aggtgtgttc agtttttgct gtcgcgtctt ggactgaggg
178741 gagctcccta taatatgttc atactacctt cctcttattt tctattttct gatattatat
178801 ataggtttta cataaaaatg ttaattattt aagaattcca actccaaaat gtatgtattt
178861 aatactttga tattgggttg atgaaaatca tgatgcagta taacaatatt ctaactgcag
178921 atttgttttt acttatttgc agccagaggt aaataacttg agtaatactt ctattgtttt
178981 gggttttata taacaccaga catttgtatt tgcaggttta aaaatcgctt tcttcattaa
179041 tctgttggag attattgttg atttgaagtt tgtgcgttta gagatgattt ctgatttcaa
179101 agccatgtga ttcaacaaag atactggaag gcatcgctta tgaattacta ctgtgttttt
179161 tctagttgtt tataatgatt gttttaagat ttttttagac aacgagcaac aataaaatatc
179221 ttgtgatagt ggtttgacca caaatatggt gtaatatttt ttttgtacgg acatattgag
179281 gcaaagtaat ttcaccctct ccatagtcaa acctagattc tttcttctat ttttttctag
179341 acaagacaga taagcagaaa gaaaaagtag atagaccaga tgatgacctg aaagaacctt
179401 ttcagaggat taaagaccta gatgatccgt agtaagacga aagcaagctt ctgaaagaat
179461 tacataaata agaacctttg catataggaa aaggataacc aaagaaagcc aaatgatgct
179521 cctaatcggt tagcgttgca ggtagtttac atgctttgtg ttcgacactc ttttgaatgg

179581 aattgctatt gatcgggcca agtagtagtt ggtcgggtaa agcaaaggat gctcctaata
179641 agaatttata gctatagcta caagacaaca aaactccctt ctaacgaacg aactgggacc
179701 ttataaaacc ccatatctgt acctcgttca ggccgataag ggtttgttta gtggccttcc
179761 tttagacata ttattctatc tggagatatg ccacttgagg agaagagctt cccgggtgga
179821 agtcccattt gaatgaatag gtcaattcgt gagatagcag tgctcaaggg atttgatcta
179881 ccttgggtta gaaacccacg ttctaaagtg acgaatcata agtctgtctc gaaaaatagg
179941 tggtcatctg cgagttagtt tcttccgtga acggatacct tcaaagctgg ttgtttacat
180001 gctaagacta tggaattttg aaaatgcaaa aacaaagagt aaggttttga cacctcaggg
180061 ctttcgaccc cccttttat agggtttagc acaaggcaaa agaagagggc tttaggtagc
180121 atatgtacag gataggtatc cagatctatt cgtaggttga aaggttggaa atccgctact
180181 aaaacaacca ttagaatgaa tgaataagca gatcgtagta ctcggttaag gttattcaaa
180241 gaccagagga tggttatgaa agaaggcaag tcggctcaca cagccattct agcgggtcgg
180301 gcagaccagg ctggtagttt gcttggcgaa agggcttaat aagccaaagc ttaaggaagt
180361 ttacatgctg atcgaagtaa actcactttg gtagtagagc ttgaacctct cctcgctagc
180421 aaccaagggt taccaacctt ctttctgtat aataagacca aatcttgtta cagtctcaga
180481 acctccctat cagtcgagct agtcaacttc gtaaccttgg ggaaggcctc atcctgcctc
180541 tcgtctggaa tctagggccg gggaagaact acttttcttt atttacttta tttagttcac
180601 gagttagggg acatccagag gggaagaagc aagcaccctt gacgaaaggg aagaactgca
180661 gctgatggga gcatcggttg cagctgataa gatgagataa gactgtgcgc attagaagcc
180721 gcagctcgtt tgagcgtccg ctgctcttgg tttttggcag taggaagctg gagctgtctt
180781 ctctttggca gtcggatggc accccgggca agtgattcaa tgtatttccc gctgttaggt
180841 cttggtagct agtgggaata ccttatgcct cagacatgat tctttcttca cctacgaaat
180901 ctcgtataga aagatggttc agctctagta ctataatgaa gctagtgtct agttctgaat
180961 gctttattcc cttttgagtc actttaatgg ttgcaagcag cctattcaac ccgaggaccg
181021 tgtccattgc ttggtgttct ttccacgtaa acttctattt tttgaactga cctttcatga
181081 gggaacgata ttgatcctca ttcatctata tgtagctgcc tttgaggggg tttctatagc
181141 ggtctgctgc gatggagtct tttcatttgg agcggacctt tcccttccag ggaaggcgtc
181201 aatcctcgat cctagctagt ttgatctctc gaattaaggt ttccgattgg attggttata
181261 agtctaccat actgtatcga aaagaatgca tgttcatccg gacattgaga aggaaggaaa
181321 ttctttccta atccggtgtc aacacttgac tgaccctagg taggtcctct cttttggaat
181381 ttcatccaca gaaccaagaa ctgatggcat caacatgaca ttacgacttt ttccttggtt
181441 gatcggatcc cctaagcgtg tagtagaaga aagaatctct cttataggtt taggacttcc
181501 tttccttaat gggctggtag ttgagtaagt tgagtgcatc gaagtgcctt ggaagtgcta
181561 tcatagacag agccccccatt gtgcgctatc ctaaggagga gagctgctac ctattctcct
181621 atgcattgct gcttccgctg tgtagaagat attgtctgaa atgaagcggg tgctagcccc
181681 ggatatcgcg acattaagag gttggtagtg gggggggcct aaaggtaaga gcatggtcca
181741 gccattgatc atgggtcaac gacggaagaa agaagtcaag ttggttgttc ctccgtgtcc
181801 tactaattgc gtaagtaaac gaaagtagct ttcgcgagct atatttgaga ttccgtaagt
181861 gactttttgc ttcaaaaaaa agaggcaagc tctttaaacg ccctaagaaa taggcttcag
181921 tcatcggttc aaatccgata agggcttctt tttggtatgc cgctccgcga gaaaggagcg
181981 aatgagcaga aatacaagca gcaaatctca tgaatgtctt tcggtcgttc tctaatcatc
182041 ttcggctatt ttcgcgtaaa tttttgattt tcctctttct aacgactatc gcctttctcc
182101 tttgtcttca atttcacttg ctcaatttct tctttttctt gcttgagaaa gttgggctct
182161 ccctgggcag tcgagctcta tcccttgcgg ggggcggggg tattgcctta acaaggcgaa

182221 tgttgctagt cgttcaattc gtctactcta ttttccgatc ctactggatt ttctttcttt
182281 ttgtttccga tggcgccaaa caaaacccgg ttcttttttg attcctacgg cttgtcatgt
182341 atcagattct tttgtggcaa ggcctgctat tcatggaaga cctttgggat gctgtaagcc
182401 ctttcctaag aggtgcagag gggggtaatc aacccgtagg tgggggattc aatccacctc
182461 ccggtccttc ggagggctcc tcaggggcag cagtggtaca gcatcaggag caggatccgc
182521 ccttcatctt gccagaggaa ggccttccgc cctccgagct cgacgcatct gagccgcctc
182581 tctcagagga gcctatacat gagccctctc ctgcgcctga gctagaaccg ctgatctcag
182641 atatggagag gcaggccgaa ataataagaa tatataaaag ggccagccgc gagaagggga
182701 cggtgattca tccaaagatg caaagtgtca aaaggattgt ggagctagag cgaaagatag
182761 aaagcgctct acgccgagat tttaccgacg agagtgttct tcggaactat ccccgttggc
182821 gcgacatcag cgtaagacag aactacgtcc cgtattattt tattatatgc gggatgaaac
182881 ctttaggaac gcgctcaatt gccaagacgt gcgcgactcg gggcctttca aacgtatctt
182941 aaaggggata cgggagagta aattcacgtt taaaaagcgg taggggttgt tgatctatat
183001 tatcgcccca gcccgcagat gcagggataa aacctgaact gcgttaccaa agcgactcct
183061 cttgacggaa gataggaaaa tattgaatcc aattttcttt ttctttatgc cagttcgagc
183121 ctggcgagtc gctcctttat cgtgtgggca gtcttcaatg cgagttgtgc gtggaatgta
183181 gcctaaatcg ttttttagtg gctttttttg ttttcgattc attttgagtt gacttccttt
183241 ctacaggaac gcccacgcag cggggatatg ggggtaaaca gggcaagcat acctaggaaa
183301 tcgagattgg gatcccattg atccagcgga agcaacggct agggcatgga caataaaaga
183361 tccttttggg ggtccggagg tttctcgtag ccgcagaagc tacgacatgg gcatagctag
183421 gtgctggcta aagtactggt gaaagtacct gaaccagtgg tgacatcggc aggagaggct
183481 ccagcactgg taggtaaggg cgaagtcatc ataagtggtt gactgggcct aggaacgacc
183541 atcggtcggg gcccgcgagc ttttaaggca aattcatagt ttttcgctcg tgtttcatgc
183601 gtgcctgtat gaattgcata agtaattgtg ttttggcgat ggagaaaaaa agaagtgaac
183661 agtgtcttag tatttagttg tgtgaagcgt ttttgacaag acttactcaa ctcactgctt
183721 tcacctgctt ccggtgacaa ctaccactct cttatgaatg ggagggtaga gtaccaagac
183781 ataggggttg tccagcgaag gcggtttatt tagtaccaga tatacgtatt tcgaattctt
183841 ttcatggcag ttcaaagaca cgagagagaa ggaaaactga aaagcctctc aaaccagagt
183901 aggaattcga tcaatcgcta tcaatatcaa tgccaggaga aaaaatccga gaagagtaaa
183961 gcgagggata ggatagatga ataggtttaa gcctttatcc gcttggaggg attcatttcc
184021 aatttctaag gcttcgtttt tctccggcaa actgctaggg aagcttttat tcccaagcga
184081 tagggcttcc tttttttcct agtctccgat caccctcgta aataaaagaa agagaaaaac
184141 atctccttct ccgatgagag catactatag gaatagcgaa aataggcttc ccttttgttt
184201 tgatccgctt cttctcctat atgtgcacta ctctctaatc agatagcttg ccgttgaaga
184261 tgatgctaac agtgagagag gagctatccc aaaagaggta cgaacggagc ttcaaaacaa
184321 gaatcatcaa atagcaaggg ctctaccgct cgggtccctc aaccgagagc ccctcactag
184381 ctttgagcca ctggatactc cgagcactac aagagcggac aatagaacga gcacggtcct
184441 acgacaaatg cttccagcgt gcctgtacat tgcgtcatcg ccgcccctag tcccccttt
184501 ttttcccggt ccaatattag ttctcgaagg ccgtgtaaaa gcaaactatc catattgatg
184561 accaagcaaa gtcaaagttg tttcgtttag tacgagatcg cttcacacct cgcggtgctt
184621 acacctctcg cctatcgaag ttctgttcaa aaacctcgtc cgagaacttg tatagagaag
184681 gatttcccgc ggcgcagcag ttcttccata ccaacttagc tgcccggcgc tgctattggc
184741 ataacaaccg gtacaccata ggttggccca acccagtcct ctcgtactag ggttggctcc
184801 tcgcagttct ccctttcaca ccaacggtag ataggaaccg aactgtctca cgacgttcta

184861 aacccaactc acgtaccact tgaatcggcg aacaaccgaa cccttgggac cttcttcaac
184921 cccaggatgt gatgagtcga catcgaggtg ccaaacgact ccgtcgataa gagctcttgg
184981 gagtcatcag cctgttatcc ccggcgtacc tttgatccgt tgagcgagag cccttccaca
185041 cgggactccc ggatcactat ggccgacttt cgtctctgtt cgaccagtcg gtctcacagt
185101 caggcaggct tataccatta cgctcacgag cagaatctta gcttgagcct accttcgcac
185161 acctccgtta ctctttagga ggcatccgcc ccagataaac tacccacctc gcagtgtccc
185221 gcccccccg aatgatcggt gcggcggtta ggcatcctta gacgaaagag tggtctttca
185281 ggattggtcg ttgtgtgtca ccacctccca cctatcctac acattcgatc aaggttgtca
185341 ctgcgaagct atagttaagg tgcacggggt cttaccgtct agccgttggt actccgcatc
185401 ttcacggaga attcaatttc accgggtcca tgtcggagac agcggggcag tcgttacacc
185461 attcgtgcag gtcgctactt atgcgacaag gaatttcgct accttaggac agttagagtt
185521 actgccgccg tttaccgggg cttccattca aagcttataa cacttctcct tccgaccttc
185581 cagcaccggg caggtgtcag actctataca tcgtgttacc acttagcaga gtcctgtgtt
185641 tttaataaac agtcgctacc ccctggtatg tgccgctttc ctaatcaaaa gataggagag
185701 caccccttct cccgaagtta cggggtcatt ttgccgagtt ccttcgacat ggttctctca
185761 agcgccctag tatactctac ttgttcacct gtgtcggttt ggggtacggt cagttcaccg
185821 ggaggatcgc cctcccaatt cgaagttttt tcctggaagt ttcaaccttg ttgactatga
185881 caacagttgc cagtcgcgta gggcggccgg gccaggccga gtcagaaagg ctttgatgac
185941 tcaaggttca tattagggaa aggagagtga ggggaagagg gggcagccct cggcccgatc
186001 atccaattcg ctccaacaga caggcatggt tctgtagtca aagcaacttc gtcactttcg
186061 tgtacccatc ggacggcagc cctttcgggg tttccttagg gaccgattca ctctgcgtag
186121 attgactgaa cgcagaaaac cttccactgg caggcgatcg tgtttttcac aggatttctc
186181 gttactcatg tcagcattct cacttctgat atctccaggt gttgtcacca aaaaccttcc
186241 ccgattgaca gaacgttccg ctactgacac ttgaaaaagc agctttcaag gtctcgtcgc
186301 ttcggtgaat cacttgagcc ctgatacatt ttcggtgcca tggagctaga ccagtgagct
186361 attacgcttt cttcaaagga tggctgcttc caagcccacc tcctggttgt catcgctcga
186421 tcacttcctt ttccactaag tgattgctta gggaccttag cgtacgatct gggctgtttc
186481 cctctcgact ttggatctta gcacccaaaa agtctgtctg tacaaacgat ctaggcctgt
186541 attcggagtt tccctggggt tggtaaggcg aaatgggcca ccctagccca ttgagtgctc
186601 tacctcgggc catcgacatc atacgctcta ctgaaataga tttcgcggaa aaccagctat
186661 atccgatctt ggttggcctt tcacccctag ccacaagtca tccccgtatt ttgccacata
186721 cgtgggttcg gtcctccaag gcctgttaga gctctcttca acctgctcat ggctagatcg
186781 atcggtttcg ggtcaaatag gaagaactag aagattccac ctctggaaag cgcctacacc
186841 taatggctta agccgctgtt cccatttcct cgctgaccca tcatgcaaaa ggtacgccgt
186901 tagagtgagt gcgcttgact aatagaaagt caaggctcta agctccgctc cttcgactga
186961 ttgttcgcat cggatctcag gttctctatt gcactcccta aatagggttc ttttcacctt
187021 tccctcacgg tacttgtacg ctatcggtca ttgaggaata cttaggctta gagggtggtc
187081 cccctttctc gcgtaaaagc gatcagaatt cgaacacgcc gcgttttact gggaaggatc
187141 gaaccatggg aacgaatcta cagggctatc accttctttg gccagatctt ccaacctttt
187201 cacaattaca gttcactgcg cccttagtac cctaaggcac taagaggctt gaaagctgac
187261 cttcttatta gagaaaggtt tggaacccta gttttggagt tttcgcccca acctaggtta
187321 gggggtcgtt agaccgtagc ttgctgctga aaaacgtaat aggctagcgc ggctcgcttc
187381 gctcttcaag ctccgccccc cttactcaac cactctctct ataaaaaagc cctttctcct
187441 ttttttctaa gaaggtccgc cttgaagccg tagcttgctg gtttttccat catccaaaaa

187501 aaaaatcgaa tgaaacctgg cgaaaaagaa gtgaacactt tgctgaggaa cgaagcttcg
187561 tgtttgtttt tcttcaaacc cccaatccgc tctcgctcgc cgctactaac ggagtctcgg
187621 ttgatttccc ttcctttagc tactcagatg tttcagttcg ctaagtttga aaagtccaaa
187681 gagcgcagac tagccacgga gcttggatac ggtttcccga tcggagatcc atggatcaca
187741 gacggtatct ccccatggcc tttcgcctct gaaagcgtcc ttccttctca atgcccgggc
187801 atccatccaa tgcattcttt tcgatcttgt accctatgta ggcttgcttc gcataggcta
187861 cacaagcggt acactgaaca ccaacccaat atttcttaaa aaaagaaaga atagcaacta
187921 cgaaaacgac aagcaactac atcaactgag gggagataga atcggtagtc tcgttcctag
187981 cggttctagc tcttattccc tctcatacga ttgaggaaag tgttctttct ctacgaaatt
188041 ctaagtgagg tcataatagt cctttctttc gaagtaaact ttcagcatcg aatgagaact
188101 tctctaatta gatcaatttt gttttgaaaa aggattgaaa cgggggaaac tcttgctttc
188161 gaacgaacag agacagagac aaaggcagca gtctagcttt ttccttttgt tttcgtcttt
188221 ctactttctg tcagctataa ccaagttgct atttcggggt taagaaagtt agttgtaagt
188281 cgcgtaatcc ccctgcctcc cttgcagtca aaactctcct ctaatactaa tagcagaaag
188341 cttccatgcc ctattctcta ttatggaaaa ggagcggagg ctagcctttc cagtttcctc
188401 gatcttggtt cttcccttat ctccatgtcg tctgattgcg ccttttctct tgtctgactc
188461 aaccacagac agggaagatt gatctgagct cgttcaatca gggagggggc cctcttcctt
188521 gttccagtgg tgtggtctca caatcctgtc agcgagagtc atagtcgttg ggaagcagaa
188581 aaggattgct tttcagtgga cataagggct agatcccgag tgttgagtag caggtcatgg
188641 tccacaagcc agtcaagtca agccggaaag agcaagaagg attgaaatca tgaattcctt
188701 tcctactaat aataagaaag gggtatgct ttgataaggg cttttctggg ctataaaaga
188761 aagtaaataa ttgctggatg ttttttggct ccgttgactg ctctctgatc tcggtttcct
188821 atatatgttg tatatcctcg gcatctgcta ttgtctcccc tgccattcca actccgcctc
188881 ccagatttac taatgagtcg gtgtgaaagt ggttccttat ctttcaggcc ttttttttct
188941 tttgttgtta ccccgtcctc tgccttagtc gctcagacgt cccagatttt cttctttttt
189001 cattaagctt ctcacttcga gttcaaacct tttcagccgc tgttagatct atagtcactc
189061 gtgactctaa aacatggtaa tccttatctt cctgatgccc atctttgtct cagtagagtc
189121 aattccttgt tgagtcttct tggctcctag ttatggaccg gtctcctccg gtcgcctatg
189181 gcttcctatt ccgataaaaa atgaaagacg agcatgcagt cgtctttcta aactccaagc
189241 cattgcagct aatcaccata gcaatgaata acaagcaagt gatcacattt ataagcccca
189301 ctccgcggtt cacatctttg agttgtaaga aaagagaatt tcatttggca ttgaacgcat
189361 accaagcccc atccgtcacc acggaatgaa aggtctgctc agttcttggc attccagatg
189421 attgtacctt tttcgaagat tggtaaaaga gatagacagt tttatttctt tcctgctact
189481 tagggactct ttggtgagag gggagacgac agtcttagct tgaacccacc cggaaagaag
189541 gaagattgaa ggttggaata gcaggcctat atgggccggt cccaatcgtt ggatagtgaa
189601 aagaaagcag aaggcgctgt ttaagccttc tatactggtc gcaatctgcc atgtagagta
189661 gaatggaatt ccaccactca gttgagagct ccgtgtcctt tctaaatgaa ttgaatgtgc
189721 gagcttctac ctggagtgct agcttggctc tcactcaacc cggaaaacta ggcaaaggca
189781 aaggtaggaa aaaggatgat tcagttaggg acttttccac tcataacgag ttctcaagct
189841 atacctgctt tagccgggat atcttttatc aggtaggttc ccttctcctc tacaggaaaa
189901 tcagttacga gttacttatt ggtagggaga ggagcgtaga gctacttagc gttagcgact
189961 ttgcgagtta gcgttagcta catagaggtg tgtagcagcc atgtgaagct tacgagcttg
190021 tgatgggccg agcctaaggc gtcccttcct acctatgtgc gttggacagc gtatatttcc
190081 cgggtctctt tccgaacttg gtaccactct gtcttacctc ttgagtttcc acggctcttt

190141 cccatcgaag tccttgttgg gcaggtgcta ttcgggcaga ttcaacttcg agctaggctt
190201 tgctgacttg aaggtccagg tggaagtagt aagaattgct gacaagcaga gctctactga
190261 acccactttc taaatgcaat cgccggatga tttcccatct cctgacaaat atcctaagtg
190321 aggtggactt tatccatacc gtactggcag gtacgatgag ctttcttaag aataccaatt
190381 ctttcggacc gaagcgcgcg atatggcttt ttggggcttt tcgcgatgcc gggataacta
190441 actacccctc ctcaaaaatg aaggggatgg accgcaagca gagcaaagtg gtttctaaaa
190501 cttcaacaag ctcggcaggg aactttccga gagcagggct tacaaaggga actccaattg
190561 gaattcaaag taacttggcc tattgatctt gaccagcctc gactggttcc acagcaaatg
190621 taacttgcct gtaaactatt ccctcagctg gcacgacatt gagaagagaa ttgcaaatat
190681 cttcactggt tttataaaat tgagtccagt cctggaaatg gttcgcacaa ggaataaaaa
190741 gaaaaggagg gtctcacacc ggtgtaaagt aaaagcaatt tccttagact cagagattgc
190801 atttgctggc ctttaactgg ctttacaaaa caggctttta tcacagcctt gatcacagga
190861 tgggaaaagt accggcctgg acccccaaaa aagagatcac catcgggacc ggtactgcta
190921 ctagattacc ccctttaaag taaacacttt caaactagcc agctggatct caaactggag
190981 aaagagaact ccccaaagca gcccttctct tatatacgat accaccagct accttattca
191041 ttaaagaaat gaagacttac ggctcgccct aatctcctgt cgaactggga aacttggagg
191101 ggaagctccg ctttcgtcca atcgatgacg tggtacttct ttctcaattc ttctggccct
191161 atccactcga ccaaatcctt tctcctatct gatctactcg actatactat aaggtattct
191221 taatcgctct acttatcagt agaggagtgg gaagcgaaga ccgtctcatc gatccaaaag
191281 cattcttgtc ctacctaaac ctaaagacag gcacccattg gggaggaaat gtatgggact
191341 tggctcttaa cggcgaatgg tacagggtca gggctcggct cttaactcat gcggtaagct
191401 ggagctaata ccagggtagc ttactctccc tcaaagtaga acgagcataa tcgaagacag
191461 agggtagcgt attctaagta gttgatctca catcctcctg tcttcttagt cttcgtctgc
191521 tctcaatgag tcaatgcccg gaacaggctc tgaatgaaat agaaatcccg ttagttaagt
191581 caccctctga ctatgggtag ctggcttgat gattgatctc ggtgagttcc acccgtctat
191641 ctaaggatgg atccccttg ccctcttttt gaggcaagtc ctatacctgt agggtgggat
191701 tcttcctttc tagcagctct caaaaggctt ccaaaatgga ttgagttgag ccctgggatg
191761 aggcctccag cacgtgcgcc ttacagaatg tctcaaccgg aacttgcaca gagcttcgca
191821 agcagttgac ggaaatgcta gagtcgggca ttgtcgttcc tgctaagtct ccatacgggg
191881 caccggcgct gtttcaaaag aagcatgatg gcacgcttca tatgtgcgtg gattataggg
191941 cgctcaacaa gattattgtg aagaataaag acccgatacc actcatcgct gattgctttg
192001 atcagctgtg tcacgccaag ttctacacaa agattgacct gcggtcaggg tattggcagg
192061 ttcgaatcaa ggcgagggat gaacacaaga caacttgcgt gacgagatat ggggcatacg
192121 agttcttggt gatgccatct atccttttgc ggctcatacg ccggtgaaca tttccttcgg
192181 cttgcttcat tttggcctca aaatcttccc actctgtaag gtctctggtt acctcttcat
192241 tgactactca gttagggaag gccaatgagt cttgtttcgc gaacaatttc ttcatcttcc
192301 cagatcggag catataaagc gtgccaagca tgcgtgccga ctttctgtcc tagtgatagc
192361 tgttattcct gtcttagtta gaggataata agtaaggaag tctgcaaaga ctgacagaga
192421 tagattaact ggattaagtt tgaaagagca gtatttactc ccaccaatag ttcagtcggg
192481 agatagcggc ctaaggtgtg gctcgggatt gacctaaaat gcactattgg gctaacactc
192541 ttcgtgaagc tttaaaatga tagcataaag ttaaagacat catagaagca aaataaaaaa
192601 aaaagaaaag gccagtccct gagagattat gacaatgcgg ctagtgcccg ctctgagtaa
192661 gactattaaa taccattctt cgcttccttg gcaccggctg gaacataatg atcttccaat
192721 agaacataga cttattcgcc tgctttgttt caaaagaaag aaccgttacc ttactacttt

192781 ttttgcttgg ttcattaaat agaggtgaac ctggttctac cctttagttt aggacaagat
192841 catcttctta caagacaaga agccatctat ctttcctgaa caggctacct acttccatac
192901 atagtaggag tggatgaagc catcgctatt aactgtccat ttgacctccc ctccttgcgg
192961 ttaaggtaac gacttcgggg atggcggaaa gctgctaggc ctgctttgtc ttagagcatt
193021 caagttgaga gagagaagtt cgatcaaggg caaggtggat gggactaagc cctcggcgat
193081 tgagagatct gtccggattt taccttactt aaaggagcta acaaggatct ttagagtgag
193141 tctacctttg tagtgagcga gtcatgggta tggctcgtga acgggctaag aacgaggagt
193201 taaaactagg caaagtccgt acccggaatt ctaggattcg agctggtggc aagggaggag
193261 tgaatacccg cttagccctt tttttatggc ttaccctcgt acccaaggga ggttttaccc
193321 accaagtcca gctttcttgt gaaaaagctc tttttccatg tctgttgatg gcgcttgcta
193381 ttgaatgtct tgattgcgca tagccctttc atgcttcttt gtagcatact taagatgctg
193441 caggagaaac gctctttgaa aggtaaatga atgcttctcc tctgtctcat gaccggggtt
193501 tttccacccc gagcacacct gagctccttt tagctagcgc cttctgtttg cctgtctttc
193561 acgtatagta gtgagaaagg ggacccctga gccaggttaa ctatgcctag catataccgg
193621 agccctcatt ttgaggtttc actccgacct tctcctagtt cggaaactac acgaggtgat
193681 ctcgaccctc actataagat aatgatttca ttgtatcctg aaagtgaatt gaaagtccac
193741 ttcgagtaag ggaataggac aggtgtgaca tacgcccggt ctctcgaccg ggggtgaaaa
193801 gacaatcaat ccgctttttt cgtttgcaca agttaaggaa gttgtatagc cagccaaagg
193861 taaaggtcct gtggtctagt ggtccaaaag gtcaaccagt catcttagaa gcagtagctc
193921 taacagttcc tccggttagc ttagagctcc tagcttgact tcgctcagct agtgcttctc
193981 cttgctaagc ttaagccagt ttcattcttt ctttcaccca gtaagtgcta tcttgaagaa
194041 ggaaaagttc tgtcttggtc cgagtgggga tagcgaaaac tcgcaacttc cccgattcaa
194101 ttactttctg cggctgatgc ctcttccccc gctttaagtt cagttccggt tagccctact
194161 tatggatcgg attagtcgcc aaaaaaagga atctatttac taggaaaaaa gtagcgctta
194221 gagttcggtg gaaggataaa ccattcccgt atggagccag cctcccttaa aggaatgtct
194281 atatggggtc accactctta tcagtagaag acagagtggg aagcagcgga acatgaagcc
194341 aatcgataaa gaaaggtgta gtccgtcctt tctttgtccg atgggaatga aagaagggca
194401 gctttctcag actcctaaga ggccaccaga gactacgata tagaccagga tctatcagcc
194461 gcttgtccag gcggatcggt aaataaagag gaaaaagggc gcagcctcat cttctgactg
194521 gccagcgggg aagactctga ctcagaactt gcgctagccc ctttcattga ttcgggggac
194581 aagtagaatt catcaacaac tgggcttacc gactctactt caacttcact tcctgaagca
194641 gatggaagta cgctcacgat cagaatcttt gcttcatttc ttcaccgggc ttggaccatg
194701 tctcccgaac aatctcagta catatggcgc aagacgattc cacatatcga ggtcggaatg
194761 ggatcgggtg ttttcacgtc tcaccgtagt gcccggtttg tcttgatttc cgattgatga
194821 acaagaagga aaatggaact cttggatttt gtgactcgcc cgcccgccac cagtggaagc
194881 aagctagccc ccggttgggg gaagagggca tttccatcgc gaaggattca atccagccac
194941 aggttcccct acggctacct tgttacgact tcaccccagt cgaagacccc accgtggtat
195001 gcgccaataa gaccaccaaa agcctttgtg gcactagtgg tacacagaag tcatgggtga
195061 tcattggtcc gatgcttcgg gcgaaaccaa ttcccagggt gtgacgggcg gtgtgtacag
195121 ggcccgggta catattcacc gcggcatgct gatccgcgat tactagcgat tccaacttca
195181 tgttcccgag ttgcagagaa caatccgaac tgaggcaatc gttccggatt cgctccgcct
195241 tacagccttg cttcccattg taattgccat tgtagcacgt gtgtggccca gcccataagg
195301 gccatgcgga cttgacgtca tccccacctt cctccagtat atcactggca gtccttcgtg
195361 agtgcggcac gcaccttttt gtttgtttcg gagccgtttt ggcggggcgt actaaaccca

195421 ctacgtacca caccaccggg cggctcgccg gaatgccgag tctttctctg ccgtcaactc
195481 gacgtcgtcg tcacctgcaa gagaaggcca aaaacttgac tttactaaac aagcgagaaa
195541 agccctttct atcttattag tcaagcgcgc tagctgcaat caaactaaag cgcacactag
195601 aaagtgcttc gaaaggcgcc ggctaccttc ttactgacag cacagctacg tgctggcact
195661 aaattagtag cgctggcacg taactcggct cctcggctca cttcggttgc aaagactttc
195721 tccttaggcg catgtctcag caacacaaaa cgagggtttc gctcgttata ggacttgacc
195781 aaacatctca cgacacgagc tgacgacagc catgcagcac ctgtatgaaa gtaagtacca
195841 tcccattaag gacaggtttt gttgttcata tgtcaagggc tggtaaggtt ttgcgcgttg
195901 tatcgaatta aaccacatgc tccaccgctt gtgcaggccc ccgtcaattc ctttgagttt
195961 cggtcttgcg accgtactcc ccaggcggag tgtttcacgc gttagctggg cccctgatct
196021 gcgtagacca agggcgaaca ctcatcgttt acggcatgga ctaccagggt atctaatccc
196081 gttcgctccc catgctttcg caccccagcg tcggtaggga cccagagagc tgccttcgct
196141 tttggcgttc cttcgtagat ctccggattt cacccctaca cacgaaattc cactctcctc
196201 tgtctcactc aagtgaattg gtttcgagag cattccgcca gtttttggcg actttcactt
196261 tcaacccgat tcaccgccta cgtgcccttt acgcccagtc attccgaaga acacttgccc
196321 cccccgtctt accgcggctg ctggcacgga gttagccggg gcttcttcct cgagtcctgt
196381 catgatcgcg cactcgacga aagagcttta caagcggcat tgcccttctt cactcacgcg
196441 atattgctgg atcgggcttt cgccccattgt ccaagattcc ccactgctgc cccccgtggg
196501 agtccgggcc gtgtctcagt cccagtgtgg ctgatcatcc gaaaagacca gctaagcatc
196561 attggcttgg tcagccttta cctgaccaac tacctaatac tacgcaggct catcaaacag
196621 cgctttttag ctttcttcag gatttggccc gaactgttcg gcagattccc acgcgttacg
196681 cacccgttcg ccactttgtt ctcaactctt ctcacctcct gggcgagaca agctaccttt
196741 agctaggagc ctcttttcct tctgtctagc tccccgaaaa caacgttcga cttgcatgtg
196801 ttaagcatat agctagcgtt ccttctgagc caggatcaaa ctcttctttt gagtatgatt
196861 tggcccttag tggtagaacc tggtgaaccg ggcgtagtac ttcccgacct tctgtgaact
196921 ttgcttctct tatgtaattg taataagaga atctttcgaa aagtcttctt tctctatgat
196981 gattctgatt cgactaggct cactaggtga ctcctgtatg cggcgagact agagacacga
197041 agtaaaatgt tcgaaccgaa tggtatccaa gggaaatgaa caacctaagg gagcgccatc
197101 tttctattag gagattggcc ccaagtctga ccctcgccat tactcttaag gttacagcct
197161 cttactcaat tgattagttc taccacccca agggtttagc gtggaccaag tgtatttgtt
197221 gaaaccataa agaaatgcgc ggaagagctc ttgacttatt gtatgcactt tactgatgag
197281 cgaaggggct ttcaaggaag gctattcaat agggtcaacc ggagatcgac tgagttggag
197341 atgaagaatt cagcgccctc ccgatcggtc tcgcgatcag ttgagatgtt tttaaggcgc
197401 ttttgaggcc ttaatcgcgg agaagagttc tctaatcgaa actttaagtt ggtaatcatg
197461 aggcttcaag atcccataca cgtgttcctc ggctaaggac ttctgcaaaa cctcttcgga
197521 ttgcgtgaag tgatccttgt aatgccagga tagggactct ctcttttctg aatctctcct
197581 cttctccgga ctcttagctc ttaggaagaa gattctagaa gaaaagtctt gtatgcgcga
197641 catgatacgc cgcattgagc gaaggacttc ccgataggag aagtctgagc gggatcttca
197701 tgaatgatag aattcatagg tactattccc ggtggctgaa atgaaatcta taaggaagag
197761 aagcaaatgt agagcgcagc gtgtctcgtg tgttacctcc tcctaaagtc ggtcgataat
197821 agatatcatt gagctcctca attgtctcac taagttaatt tcatcttgaa tagtcaggct
197881 tgtttctctg ctgccaaact aatactaagg cagccctccc tctttttctc tatctatgtt
197941 aaatgtcaga agcttgactc atctcagcaa gtttatttat acatttgatt gataggatcg
198001 atgcccgctt ggttctttaa atcgattgtt agttaaggtc cgggctctat gcttggccac

198061 tatgttgagg aaatattggt tctaccaaat ggtagtttga acgcccctcg tgttgttggc
198121 tgtctcggta ttcgttcttg aatcattgac tgagagtaag ggctagtgat caagaaaata
198181 agactagtgg ggcatcttcc ttttcgtagt aagcaactct ctttagaaag ctctttcttg
198241 atcgaagtaa agaggaacta aggagtaacg ctttttcgtt cattccaagg aagctcctta
198301 aagaggattg cacttgggat cttagcagag gagctccgag acttagaatg tcctagataa
198361 cacagttaac agtggttttc cctttcatct aaagaagatc gagttgtctg gactaatgat
198421 cctaaaggtc ttttttcggt tcaatccgca taggaacttt cttaggaaag ctagccttaa
198481 ccaaaaaaga aaggaagaaa ccctcttcct atagtaaggt cgagtaagtt gtaactccct
198541 ttcaagatac ccgctagctc catacctagc tcgttcactc cttacctact agccttgcta
198601 ctaagaatat tccataagat tgcacacgtt tacctcagat gtagatcgaa cggcataaac
198661 cgaagaaagc aaagaaaatg actctagata agaatattca acctcaaccg aataaggagc
198721 aggtttagcg gtatttgcgg ctgaaagaaa ggtttcaagg agttttttta actcgacgga
198781 agggtactac taaaaggata ggccctgggg tacgagacta ataagtaaag gggcacccgc
198841 tcttctttct aaaagaaaag gaaatgaaca ctcgacaggc gggcctaacc caataggtct
198901 tgagtaaact agccgcctcg ctcggacttg cctcgaaagg tcaattcctt aggattaaca
198961 tcttcttttg aagtgttttc tttgtttggt ttgatttgtt gtgtttgcat gtatgggcta
199021 gccgtcagtg ttcaatgctt attttaatag aataacgtaa gtaaaaatgt gctgaaaatt
199081 catgttcaag ttgaaataag gccgaagggc taagtgtact actggagatt aagcttttcc
199141 agcaataaaa gaaggaagaa ttcacttcag aatatgcatt gaaaacactt tctttaactc
199201 gccaaacgtt aagtgcaaag gaactacaat gtgctctccg gatggatatc ccctcgtaaa
199261 ctcattctcc aaatcaccca aattaggaat cactctacaa ataaacctca gcagttgatc
199321 aggctaatcg acgaaaggcc attctttaac aaaatgttta ctgccaatga ctattccaac
199381 agccaaatca acccattcat tatcatgata atccaaaatc acattcttgt acaagtattc
199441 aatgtctgag tgaacatcag tcccaagcac aatagcctcc tctctggttg agccagagga
199501 agcactacta aatccatttc tggatcaacc tgagcgtccc caatctgttc tacatcttga
199561 attttgtatt ccaacacacg cgtgcgagga tccacagcac gacaaacaac ataaccacca
199621 acaatgacat tattcctttt tttttaacac ataatcaatc aagccagtgt ctccaatatg
199681 catacgagca gcatcccgaa cttcttgccg agtcattcca caattacaac ccccatttgc
199741 tgctttcttt tctttcagtg cattaacaac aacttcagca acatactcca ttcttttcat
199801 aggccaccta ctgtccaaat ttgtagccag tgtagtaaat ttcttacatt ttcaattttt
199861 tcctttcact acattcttat tctgcacaat ttttcttgct gaagatctca aacaacccga
199921 tactgcagca tcagtaggtg actttcttgc cattaggcat cgggacttga gagaaagcat
199981 gaagtaaaat agatcccgga ttgtgatcaa ttcggactca ctcaaatctc tgtaataata
200041 aatgatccgc ttgacatcac catgaagaac agaaatcttg aatgatctga tcaagtccaa
200101 gagaactaag aagctgtatg gccctttacc gagtgtgaag atttgtgcaa attctgtccc
200161 agagatccat aatttcttta ccacacaaga atttagatct accgtccatc ccattcggag
200221 cggttggaag gggtttttgg cctgtccttg gcttttgcca gttttgttga agaggaattg
200281 gtatttcata accgatttca tccgaaagct gaaacaagga gcatgcgagc ctaggtaaat
200341 gagttccaga gtgggacgac aaagagaaag agtacaaagt atagttccag gggaaggatt
200401 ggaagaagca gctagaaagc ttggccaatg gaagtagaag gcaaagctta acccgtagag
200461 ttcgtgtggt tggatgaatc caaagcagaa gcggaatccc attgctgagg ttagttgacg
200521 aagctatgtc tgatccctcg tgagccagac tatttggaac aatcctgtta tgaagggccc
200581 aaacatggtg tattggcgcc cgcccttgtc cagatcaggc ttggcaggga ggaaggcgta
200641 tgcccatgct tggaagcttg ccagactctt agtgaaagag taaggcaaca agaccgaaga

200701 tagtgttatg gtgggcctaa tcctaatccc agctaaaata aacgaagtag atagagttgg
200761 gtgggccagc cccatctttc gatcgttcgg gaaggacgta gaatgtgagt cccatagtgt
200821 agaacaccca acgagtaaac taccttgcct gcttgctcgg ttgttttgat gcaatacttt
200881 ttccaaataa agtcaacgta gagtctggcc ctgtatccga ggtttcatct ggaatcacgt
200941 ttgatgcctc tgtttgtaac aaccctgggg caaggcccct catctagtgg gccgatccaa
201001 ggagcccttt ccttggttcg tttggtaagg aggagcggcg aatcccatgg tttcattcct
201061 gttttggaaa ggggctaaga cctattaaag gttcggagag gaaagcgaga gcttgattcc
201121 tataattaga atagaataaa gaagtggagc agcaactgcc cctttttaga ttgatggtaa
201181 tggatgggat taggcaaagc cttcgcgccc agggctttcc cctaattgta tgttaggttc
201241 ttagtagcat agccatccta aactatagat cgataggcaa ttcttaccta gcatttcaat
201301 gctttagctt tcggtcatta gctctttctc gaaatatcgt aagagaataa aagcatatgg
201361 aagttgactc gcttactcgc taaggggagt gagctcaact gcaatcgtgc tactggcttt
201421 taaccggatt gctactggtt cgctggcaaa gcattccctc tctcttcttt ctttactcga
201481 gtacttcgct tgtgctgctt cccgagctta tgtactctcg cttaactgac tacgatatga
201541 gttcgcttcg caccttactc tatcacttag ctctagtggg cctagctgac cggcacggaa
201601 aggaatggaa tttatttcag cttcactctg ctatcagact agttgcgaat ggtcaccttg
201661 ttgagagctc ggtagtctac acacaaccgc aagttcccgt cgatggtatg agtgactgag
201721 tggagtggtg cgagcatcac tcttaaataa attggaatat tttcaaaaga aagaatgaag
201781 ctatataatt gcgtagatag tattgtgaag aactcttctt ctagcagccc tttctcctct
201841 accgaaggaa agatgttaat gcgagcagcg tgaccagtcc taccatagtg cccggagaac
201901 gagaactggg cttgctttca taagctagct tactaaggcg aaggaatgca tggtaagtcg
201961 gacagccaat cgaaagctat ctcagttgct aaaggaaaaa atctttctat ggaaccttct
202021 cttacaggcc cgggtggaag caccgcaact cctcagcacg ttcactcttc tcctaacact
202081 cataggcggg aaaggcagac taataatggt tcaaaagaag aatagttttc gtggacttga
202141 accagaaacc tcgcctgtac gtaaacctgc tgcgccatct ccttcctcat cgaatggctt
202201 catcttcgtg gtccgactga tctttgtaaa gcatatagct agcctcccct tgtacccttt
202261 gtaggctttc ttcttcaaga aagagggaaa tgctagtgca ataaaataag actttatgaa
202321 ggctgtgagg gagctgcttt gagagaaatt atgcctattc cattcatttt attcgatagc
202381 atccgattac tgaacacctt caacgacggc acactgaaac aactcaagcg aaagaagccc
202441 ccactcccca tagagatgag aaatctttcg cctgtaaatt caatgaatga attacctctc
202501 aatgatcttg aatcagatca atatcatgaa taacaatctc tgagctatca aatcaattcg
202561 tcatcgcgaa ttgaatagta taacatagga agacgtttta tccatactga atccaaaata
202621 ggattcccag cccaatcaaa aatgggaatc aaattttcaa tttcccatta gtaattgagg
202681 ttacacaaac aaaaccaaaa cggggagggg ttcttttta aggcggaaaa gtattctatc
202741 gggggaattc tgtgaaattc attttgtttt gtattgtaca cgaaaggaat tccatttttt
202801 tgtatgtgcg cttgagaaac atatagtcct ctattccatc gaaaaagcag actcagtctc
202861 tcttggaata ctgactctat tgctccatca attgtactaa tctacatatg tctttctact
202921 accaatgagt accagttgaa gtactcattg cattcatctt gtcactgcat tcagcaatga
202981 gcagcaaaac catatgtctt cctccttgct gtaggagcta tggcgattct aatatatata
203041 tagaataata ttctataaaa tagaattttt ataaaaaaat attacataaa aatatgtttt
203101 caatccacct aaaattatgt gtgaaaagtc aaagcatcag ttatttaggg acggagggag
203161 tatcaaataa ttttttttta gttagaaaga aaacgggttt accctttgag taggtgaagg
203221 gtcatctgtg agtccgtgac tctttctcag aaaaagtcta gccatgaatc acagaagcta
203281 aaataaaaaa ataccggact gagcagttct atactagttt tgccgtttgc tgtaatggtt

203341 tttcattcta ctatttccga gacacctttc tatattaatt aatacaaaaa aatagttggg
203401 aacaacaaat aattaaatca gagtatggcc atatctgaca gcttccacag acaaatctta
203461 tcactaacag agaccggcag taggagtact gtttagatag tagtactact tttattaact
203521 aaacacaatg aaattgtcaa aagaaatatt atcatgaaaa atgaaagtat tcatcgaatc
203581 tgttttattt tatgtgaatt tattattctt tatataacga aaattatcct atctatttgt
203641 ttgatgagaa gggcgaaacg aaaaaggaaa gaaaaaagaa ccccccttgggg aatgaaattt
203701 ttcttcttgt tcccccgttg acagaaaaag gaaagctgat tgatgtactt attgaatctg
203761 tcgggactga cggggctcga acccgcagct tccgccttga cagggcggtg ctctgaccaa
203821 ttgaactaca atcccaggga aataagggat ctagcagaaa ttttgattct ttttttatct
203881 tcattcgaac tatttacttt cgccgtgttg taacagaaac acgaatgata gactatattc
203941 ttatatcatt atatatattt ttaaatgcag tagactcata gtgggctaat tcatgaatgg
204001 aatcaaatgg gcccttttca ctaaagagtc acgctcttta aacgccctaa gctaagaaag
204061 aggcttaagt catcgattca aggcttcttt cttttccacg aaactcctgt cttagtcttc
204121 atttttaagc agaggatata ggtaaataga accagccgtc gattcgattg ttttccaaga
204181 atgttgggcc gggtatgtaa gccatgtatc tgggattctt ttcaaaatga atctcatctg
204241 gacataaaaa ataaattttg agttccccgg taattccttc aatagcggct ctaatgtaaa
204301 atgaaaacct tccacccaga tctgattcta gcacaactgc ggttagtgat tcaatcacag
204361 cgggtagacc aagagcagtt ctttattcaa cagtataagg agtaaattcc ttcaaagctt
204421 ccaccgggaa agagccaagc agctgctatt tgaacaacgg atatggagac agcgtatgat
204481 ttcaaagttc aaaaatattg aacaacggat tcagccattt aatagaatat ctacaactta
204541 tgactccact ggtagtatgc tagatgcaac ctattctgta acaacttatt ctggctatag
204601 cacctgctca ttccctgact tcctttcttc tctcaggaca ttctcttctt ctttaactat
204661 gtcatttgct ttcactagca ccggttacga gaacagtcaa agcagattct atataagagt
204721 accttctgcc ccatttggct caatagccgg agatgaaata gcagcttctt ctttcacaag
204781 tgaatgccca attgaatacg aattaaaaga gcgtttatcc cgctaagcgg attctagcac
204841 aacaatttca ttgcctccta ttacaaatcc cgctaagcac tctcgttcta gctttccatt
204901 ggattgcttt ggatgcttcc gcttagcaga aaaagaaagt ggagattcat ctctagaatt
204961 tgaattaggg gattttcatc attaacttta agctctcaag ctgccccctcc tccctgctgc
205021 tttcctaaaa agtacaagaa gacagttggg tcaaaagcat cccaactaac atgggtaccg
205081 cccttgacca gctaaaggga taggataact tgtctaggag aagcaagata caaaggtcca
205141 atagacacct tcggggcact cacgaaattc attccttacc tttgggcacc tttgacaatt
205201 agcttgaatg agtcttctcg ggaaacccaa gaaaagatag ctgaaaaaga acctaaaaga
205261 agaaaaacac aattgcataa gtgagggcac gaacaaaaaa gacagagaaa gccctcaagg
205321 gtgtaatcaa agcagaatca gagcatcgag gaccaacgac gatgaaggag gagaaggaga
205381 tgagccatat gtggaccaga tggagggaag gggtcaagta caaggaggga ggattgcttc
205441 tctcacccgt acgtaggcga aagggaaggc atcggaagag ccaaagtccc aaaccaaaga
205501 acgagttcag ccacttccgt cagatacgag attgaaagat atggctttgt cccagccgtt
205561 agagcgttaa gccgctcatt ctcttaggtc gtctcaaagc gcttagtcga aagactttgg
205621 gtcaacgggc ttagtggaaa ggtcaggggt ttgatcaata gtattgattc tttctttatt
205681 tgaggatcta agccctgtaa ggacaggaag taagtttata agaaactaat gccccgcaggt
205741 aaattgcaag aagtcaagca gtctcaaact cgactgtgaa atgggattct atgtatagat
205801 cgtttattcc gaaacctaac aaaccgggaa agatgagagc tatcacgcaa ccggctaacc
205861 ggttaagagg gacataatcg ttatggatgg gctttctctc ccaaagggaa tccactatat
205921 gaaccatcaa acgcctattc taatatctaa tttgttgtct ttctaaactg gtcaataaac

205981 tgccttcaag ttgttccgga ggtacctaag gttaagggcc tcattcaaat gcgaatatag
206041 gcctgattga agcataaaag tttcataata agaattagag gcttttgtaa ccgatgccac
206101 aaaggttgaa aacggagatc cgttcgcttg acggaagcta cttaccttat tactatcaaa
206161 gggaatgaca tggcaaagcc tttagcacaa tcaagcaatc aaagtaagag gaaaagatcc
206221 aagctagccc gagccccgaa aggaatcaat ctcttttaac caaccccctct tactagatta
206281 gattgagaaa aggcgatctc gatacgatct ttctaaacca atctatcact ggcactggaa
206341 cgaaccattc tatactagta ctagtatact gagtagcgtg atcaagtcaa gtccttcctc
206401 cttatttccg gatttcaatt agattaatag attaatgtgc gctcgtatgg gagtagaaga
206461 agccactact tccctcttcc cccgagatga actactctcg caaagaacct tccttctatt
206521 ggctatatat ctgtagtcac acagttccag ctatcttctg gatagaagcg caggtgcttg
206581 agtgaaaggc agacaggtaa gggtaggtag taaaggcaag cgcatagttc agtgatctac
206641 ggccgaaaga gggaactctc ctcgttccga gtcgccatga ggcataagag caaaacattc
206701 ccctagaagg gagcttcgat aggtatcctt ttttttacgc tatttgcttc gatgctttac
206761 tactctttca tgtcggaaat cggattctct tttttcttct ttcttgtctg agactgatgc
206821 cagcttagaa agaaagtgag gcactattcg ctaatcagga aagaggcttg actcgatctc
206881 tattcctcct cgtacattaa gcagagcagg gaataggaat agtaaaagaa agtaggaaag
206941 tcatcaggta ggtaagcaag ccgccctggt gccaagctaa agacaaggat cacatcgata
207001 agagcaaaag ctttgacggc ttatgccgac tcacagaaaa ccaagcctaa ggactttcat
207061 accaggaaat ggaagatgtt cctctatcaa cgtaaagtta gtcccagcga atcgctagaa
207121 agactatgag gaaggagttg gcatcgctgc tataaccttt ccaatgaact aggttagggc
207181 ggtggcggaa cgtacctacc gtcagggact taggagctag gaaagtgaag cttcactctc
207241 tccaaccatt cacaatttct cattcgtctg cttgacaccg gattcaggtt ttgttttaga
207301 gacagagttt ttcaatgtag ttgttaaata aaccatgtcc gctggaataa tgaaaaagag
207361 aagctctatc ggctctttct ttcccatatc tatattttct taataatatt tattaataat
207421 aaaggtaaaa ttatgcttct agaacagaaa gaactaagat gaaccaaggg agtactggag
207481 ctaagcgaac aaaggtaccc ctccccccag tttctcctca tttgagcctg tattttatgc
207541 tttaaggtaa tttttgagat aagtgtgagc agcgcgctct atcgctcctt cccatccaaa
207601 aacgcccgat tttaggaact tagctctttt tggttgactt ccgggtgaac aaatttccca
207661 ggagggggttt ccgcttattc aatagccatt agaccggatg tcatgattga tccccatccc
207721 cgacagggta gatctgtcgc caacaccata tgggaggggg gaagactatc actgaccgag
207781 cttctctata ctgaccttct tttcttcgat tgaatgaagt acttctgaac ttaaatcatg
207841 aattgggttt gaaccaatat ctctttttgc gacttccatc atgatgggaa gatcccgctg
207901 cccaggatct cggcatccct cccacctttc ggcgagatat ttgccgtgag tacttttcca
207961 ttccagttta aaaaaaaaaa aaagaaagct attgcagctg ctcccgcacc gattgattta
208021 gcaccttcta acatcgttac tcttttctcg aattctcctc acgcttttca ctcttttttt
208081 tatctccctt ctctatctgg atcttcatct tcctttttaa ggatgaattg aagcagcaga
208141 agaacgagaa tttgaataaa gaggctagct atccaaaggc tcatatcaaa agaagaatca
208201 tgtaaaaaga attgatttag aagatagaat gccgggaaga aaacgccacg aagcgtggtc
208261 ttggattcag aaagaacgga ttcgtttttc atttgactta gtagccataa ggagatgaac
208321 aagcaaagaa tggcaacgag aaagcagatt gttatcttgg ggaaggtctc gccttctttt
208381 ctaagaatga aacaaaaaaa aaaattagag attatataaa cccacaatcc taccgttctg
208441 tcttactgag acacatgaag tgaacacatc ctgccccccgc ggcggataat agaatcacta
208501 aaaggaaatg aatatagcgg aaaactccgg aataaaatag atagaaactc ggaagaagga
208561 agaggagaat gaaagagaag aagacccaca taggaagttg gttgttttcc ttcttctctc

208621 tataattgaa gccaaacagg aatatgagtt ctagtactaa ggcaatctcc cataggacca
208681 tttccggtga catgaaaaag cgacttgtca aaatcgtcaa aaagaatata tagaattcca
208741 tggagatctc ctccgagaga aaaaaagaag aaaagaaaaa ggaacaacag acgcaaatca
208801 ccactaaaat ggctatgtcc ggttcatgca catgattcat cacgaaaaga gaagacgaac
208861 tgagcataaa ggaatagagc gtcatgagct tggaaaagcg ccttcttgct ttaaaaagaa
208921 agtaaaaggc ggacgcccca cccatgaaca tagggatttg ctttattatc ccaagggtct
208981 ccgtctgaag tatctcggag aggataagaa tgatcaaata ggaaaaagaa atcaacagaa
209041 ttgcagaaag gtatttgatg accatattct tttttttac tatatgaaat ccacactttg
209101 acacctaaga ttccgtaacg agtagatact tccgcgggag cataatagat tcaacctgat
209161 tcaccgcctc cgtgccagca gccgcggtaa gacggggctt cttcctgatg aactcgaaat
209221 atcgtaagag aagaaaaaga atcttacgcc caacaactcc catgtctttc ttggtcggac
209281 caacccaacc ataaatttcg ttccaagtct ttttgcattt ttagagcaag aagcggaact
209341 acaagaaagc tttctttatg gataaccaat tcattttcaa atatagttgg gagactttac
209401 ccaagaaatg ggtcaaaaaa atggaaagat cggaacatgg gaatagatct gataccaata
209461 cggactaccc atttccgttg ttgtgctttc ttaaattgca tacctataca agggttcaag
209521 tttcgatcga tatttgcgga gttgatcatc cctctcgaaa acaaagattt gaagtggtct
209581 ataatttact gagtactcgg tataactcac gcattcgtgt acaaaccagt gcagacgaag
209641 taacacgaat atctccggta gtcagtctat tttcatcagc cggccggtgg gagcgagaag
209701 tttgggatat gtttggtgtt tcttccatca atcatccgga tctacgccgt atatcaacag
209761 attatggttt cgagggtcat ccattacgaa aagaccttcc tctgagtgga tatgtggaag
209821 tacgctatga tgatccagag aaacgtgtgg tttctgaacc cattgagatg acccaagaat
209881 ttcgctattt cgattctgcg agtccttggg aacagcgtag cgacggagaa taaagaatca
209941 gaataggtcc agtccagggg acaaattaat aggaaatgct atttgcttcg taagaataag
210001 aattcacttc tatgaaatga aagagtttca cgggaattgt cttgatcgtc ggatatcatt
210061 gataaaaaaa taggaagaga agaaagtgtt ctcgaacgat ttcctgcaat tcctcccagt
210121 atggaatgag taaagaatca agctacaagt ctcgatctat acgaaacgca ctggtttttt
210181 tttcggtttc attgatagca gaatatcctg actctatagg ggcgctagcg cttgactaat
210241 agaatctcaa aggggcccga atgaaactta acttaactta ataggctgct actctaggct
210301 cgcttcttca agctccgccc cttattgaaa acggccttgc taacgagcaa tctttctaaa
210361 gcgctaacgc gccttttcta ttagaatctt agtaaagcaa cctttcgcgc taggcgctcg
210421 cgttttttgt ctgggtggaa gctggcggca gggcttgctt aaccggatac acggaattgg
210481 ggtctctgtc gcatgcaaat ctttctatcg ggaaagcctc ggacactgta gaccaaaggg
210541 atgtagtgca gcttggtagc gcctttgttt tgggtacaaa atgtcacggg ttccaatcct
210601 gtcatcccta cctattcttc tcctctgggc agtaacgagg gatccattga gatcgattca
210661 aattgggcat acagaagatt tttcattttt ttagactata tgcatgcaag atgtattggg
210721 ggtacaaaaa gaatcctttt ctttacagtc gtatctactg tgataagaaa gcgctcttag
210781 ttcagttcgg tagagcgtgg gtctccaaaa cctgaagtcg taggttcaaa tcctacagag
210841 cgtgattccg ttattgttag gtctaattac aattaaagaa cttaactaac ttgaacagca
210901 gaaaatagaa aaactagaag agataaagcg tgacgagaat tctctaatta tatacataca
210961 aatattatgt tagaaggtgc aaaatcaata ggtgcaggag ctgctacaat tgctttagcg
211021 ggggctgcta ttggaattgg aaacgttttc agttctttga ttcattctgt agctagaaat
211081 ccatctttgg cgaaacaatt atttggttat gccattttgg gctttgctct aacagaagct
211141 attgcatcgt ttgctccaat gatggccttt ctgatcttat ccgtattccg atcgcaggtt
211201 gaagtttcat aaagcctcct tagtcagtct aagccgcgct gaagctttca cgaagaggaa

211261 aaaaaagagt gaccccggtg gacaaatgaa atcgtcgtaa gggggaggcc ttggttaatc
211321 aatagcagtg agttgattga ccaagggagg aggggtggtg tggcgggccc tcctcaaaat
211381 ggtttttag tcgtctttgc tagccgcctg cattcccact atatcttgga cagaactaca
211441 taggcttgat cccggccctc tcgttcagtg atccccggag cggggcgcgg tcaagggatt
211501 cgggggtaga ggaggtcggg catccactct aaacttcgtg ctagcattct ggtaagtcta
211561 gctcctctaa acagcctggt aaagagcaga gcacggccgg ggcatctgcc caagtcctag
211621 gttgagctat ttatgattga ttgtcttacg acgagaagcc ttctttcttc cccgccgcta
211681 agacacacct ctttatatgt gtgcgcgccc gtccgtattc tttcttcttc cagggagttt
211741 caccccaaat agcatacttc acctagggat agagatcttc cttaagcaat ggaaagtgtg
211801 cggaaagcga gtttttcaat aaagtatact gcgagatcct cggaaaaggg ggtcggaatg
211861 ggtagcttta ttttatggag caatcatcaa gggaaagggt gggtaagttt aggatcaaac
211921 tgaactaaat atttcaattt atcctttgaa ttactcatat atatcctatt aatttcattt
211981 cgcaccggaa acttttctag gagaagttcg aatccgtttc gttcggatat tgatcggttt
212041 tggtttgaca tggtttacgc gttactggtt cccggaagag ttaatatctc cattagctaa
212101 accctttctt accctgcctt tggactcgta ttttgttcgt acacaatcaa cggaggcctc
212161 cccgacatat gttgcaacgt ctccaatagc atgctcttac ttcgtctttc ccttaataag
212221 tcatcaaatt tggtgctttt tgatccccag ttgctatggg gaacaaagga cgaaatacaa
212281 tcgattcctc catttaagtg gttctcgctt ctccttgttc ctgttcttaa ctcttccccg
212341 ggtaattccc aatgtttggc actttccata cttcatgggt gcaacatcaa caaattcgct
212401 catgatcaag ttacaaccta agatctatga ctatattatg ttaactgttc gtatttcgtt
212461 cattccatcg gtatgctccc aggtacctgt aattgtgatc cgtttgccag aaccaagggg
212521 tctttctgtg gaaacctcca cgaacaatcg tcgtttttg atggtttttc cgcttctcac
212581 agctgctctt tccacacctc cggatatctg gtgccaaatc gtctcccgtt tccttatttc
212641 tttgataata gagttggcta tctttgtggc atcgattgta caagttcgtg aagagggctg
212701 gacgagtgga atgagggaga gcggctcgat cgacaaaaaa gaagagtagc ccccccctaga
212761 acctggcaaa gtcattatca atgaattccc gagcaattat caaccaacat atgcgattca
212821 ttcccgtaaa gattataaca cacagaagac ttcttacctt aggagcggga tgagtgatac
212881 atccggcgag cgcccggtgaa gaacgcaagc aaagctggag ctcgggtatt gatatattcc
212941 atcaagagaa aggaggcaga ctggtaagaa ggccgaccac atagggggag cgaaccgaaa
213001 agctcagctt tgcggagcgg accaatcttc cgtaccgccc ccttactctc tctctataaa
213061 aaaaagcctg cgggaagcgc gaagagctaa gccactaatg tcgtggcgaa ggttatacct
213121 cagaaagtaa gcgaagctaa ggtttcgtat gtgttatatc ctttcgatcg agcgacaact
213181 tctaaggaag gccttcattc ccctttgaaa gaagttaaaa aaagagtgct agggaaatgg
213241 agagagagaa agttgcagaa gtgcttctag atctcggccg ctaaactaaa ggagtccttt
213301 accaagtaag ctagacaacc gtctttaccc gccttaggac ccctactgtg acaacagcta
213361 ctacgcatcc cacattataa aatgctatcg acgaaacaaa cctttatcaa gcatagcacc
213421 atgacctggt acagaaccca tctttacttt tcgacatccg taacggatta agaaaagcgt
213481 tctaacagca attacacagc ccctgaatgt cttagtgtaa gtgaaagaag gtaagtagct
213541 gggaatgcgg ctagctcagc tagtttactt acttgtttgt actccaatct gaaatactac
213601 ttaaacgaag aaatctccca cttctcctac gaactaccat gctctaactc cagtttacag
213661 aaagctaagc ttgcttgttt cacagacctt ttcccctaac aagcagccct ttcgctccat
213721 agtctatgcg actaagcgaa gctcagctcg gcgataaaga aaatgactct tcataaatcg
213781 ttcgtatccg tagctcagtc gctagtacgg agactgcacc gaaaaggcag aaaagtagtt
213841 tagtttactt acttttcttt cttaagataa agaaggaatt ctcacaggtc aggtggcact

213901 cccagatgtg caatcaaagt ggatttgctt ccatcaaaaa attttggaat aagaggcttc
213961 attctggcca caatcacttt tgaaactgtc ttgtaaacag tcttacaaca agagattggt
214021 ctgatctcat agtattaggg ttggccactt tagggataaa agatatagca gtactattcc
214081 attctttgag cagcttacca gatctgaaga aagaagtcac agcggctaca acatccttcc
214141 ccacaatctc ccagttatct tggaaaaaac gactaggaaa cccatcaggt ctaggagatg
214201 ccatgagaag ggatactcat tttcagtctt ttactttggc agatagttga gttcagtctt
214261 atgttagaga cccacgattc gttttagtag tatgtctatt acaagggttt acgatattgt
214321 gggcttaaac acaccctcta cttctgatga gaccagacct gtgttgttcg cctcatcatt
214381 tataagtagt gcgtctggca acttcatgcc ccatggtgca aataggatac gatttctgtc
214441 cgcactcact ccattgtgca caggtaatca aagattgctc tcaccctacg aaaccgtctt
214501 tgacccccta gggctcaacg cactacgacc atctagtaaa gccatctcac tttccatcat
214561 gctaggtgca gtagtagtag cgcaggcctt aggagaaaaa gtctccacat tctaagctaa
214621 gcaaaggcta aggttagagt caaaaaagac taggaaagcg aatctaaccg ataaaccctt
214681 ttttttagga gggtctattt cctttccaca cctgtgtcgt acaaccacct aaagcgcttc
214741 actcttagct agacgaatgc ttctcacctg agaatccctt ctgctactaa cggaaatcct
214801 tgcttcgaac tgacttaact taatagggta gggcggctaa tgaactaata cgactttcta
214861 gatggatgca ggagaccact ttgctaccgt tagccttaga aagccagaag tgagcttctt
214921 tgtcgactca aattcccccc ttccctgtcg cctttggcta aattccttgc ttccctatac
214981 agctaaagaa caactacgac tgcacaacga ctataaaggt tactaaggat aacacccttg
215041 tttctagcca gctcctctag ttgaaagaga atcccgaaga gtttttcttt tggtgagata
215101 ctcatgcata gattgagtat ccttcttttt ctttaagagg tcaaacttga gctgtatttg
215161 acgagcagca gcttgatcga cataaagctt gtggattgca tcccaagttt tcttgacagt
215221 agtggtaccg gtaggagcat agacgtgtta aaggatttca acggtgatgg tggagttgat
215281 ccatgtaaga agtaagaacc aaggtgtcga gttgacgcca ttgaacatat gctggattga
215341 gggagagaga gccattttaa ccaggaagaa agtaaggagg ggcagggggtg gcgccggtga
215401 catgtccctc aacattgtgg gctcgtgtga gtaggaaaca aaaaatttag attctagttc
215461 tatcatcagc tatgggttcg aatctaagag aaattctaga gaatatttgc tatcctgaaa
215521 tatttttgtc ttttctgact gattaaggag aaaaaaaaga gggtcaaaag aaaatgccat
215581 tttggagttt taccaacaat ttgcttgtgt aggcggggat ccggtatttt atgttaggtt
215641 cttagtagca atcggagact ttttcttctt ttacttcaca tagcctttcg tctccttgat
215701 ggctggaagt tctccaaaag tatgaaaagc tggaggactt tgtaccatcc attccggtgt
215761 ggttggattc tgttcaacag cccaaggact tggagcgcat cttttgttgt ttccactgct
215821 tgaagtgatt gttacgacca cgaagaaacg acaaatccca actacggata tataagagcc
215881 agaactgcta agggcattcc atccagcgta agcatctgga taatctggaa tgcgacgtgg
215941 catacccgaa agccctaaga aatgcatggg aaagaaggtc ggattcaccc cgaaaaaagt
216001 tatccaaaaa tggatttgac ctaaagtttc aggatatgtc cgaccaaata ttttacctac
216061 ccaatagtga aatcctgcaa ataaagcaaa aacggctccc atagaaagta cataatggaa
216121 atgtgcaacc acataataag tatcatgtag agcaatgtct agcccagaat ttgccggaac
216181 tattccagtg agtcctccta tggtgaacaa aaagatggac cctacagcaa ataacatggg
216241 tgttttgtat tgtatcgaac ccccccacat ggtagcgatc caactaaaga tttttattcc
216301 agtggggaca gctatgatca tggtagctgc ggtgaagtag gcacgggtat caacgtctaa
216361 gcccacagta aacatatgat gagcccaaac aagaaatcca agaacaccta tactgatcat
216421 ggcataaacc atgcctagat acccgaagac cggttttccc gaaaaagtag aaacgatatg
216481 acttatgata ccggatccag gcagaatggg aatatacacc tctggatggc cgaagaaccg

216541 aaagagatgc tggtataata tggggtctcc ccctccagcg ggatcagaaa aggttgtatt
216601 aaagtttcga tcggttaata acatggtaat tgcccctgcc agtaccggaa gtgataataa
216661 aagtgggaat gctgtcacta gaacggacca cacaaatagg ggtgatctat gcatagtcat
216721 tccaggtcca cgcatgttgg agatagttgt tataaaattg atagaaccta aaatggatga
216781 aacaccagat agatgaggac tagaaattgc tgaatcaact gctcctccag aatggctggt
216841 aataccactt aagggcggat agaccgtcca cccagtgccg ctacccactt ctactaaggc
216901 tgggcttaat aggagcaaga gacttggtgg caacaaccag aatgaaatat tatttaatcg
216961 tggaaatgcc atgtcaggcg cacctatcag aatcggaaca gaccaattac cagatccacc
217021 tatcatcgcc ggcataacca taaaaaagat cattaaaaag gcgtgagccg ttattaaaac
217081 attataaagt tgatgattcc cgccaagaat ttgatcgccg ggtcgtgcta attccatacg
217141 aatcagtact gagaagcatg tgcccatcac tccagcaatg gcaccaaaga tgaaatatag
217201 agtacctata tctttgtggt tagtggagaa cagccatcgg accggatttg tcgtaaaatt
217261 gagattcttt agtttccttc cttatcagag aggggtccct tagggagctg gggcttcttt
217321 ttattgggga gctaatgcac agggaagaag cttactagaa aaaacctaca tacctacaat
217381 agggccatac gtctctagaa cttgaagaaa gtagatactg ctcgttccat tactaatgag
217441 atcaaaatag atttggttta gcccgagaat tttctcttgt atactctcct ctattagaaa
217501 ggcatcttcc actatgtctt tgaaactgag tccttcttcc aattgcatat ttggattatt
217561 atgactatat tccttccaaa gcatctcgag ttttgatttg gtcttttctg tcagtttttc
217621 tagatccaac tcatggatac gttgcgaaag attacttcgt tcaataactt ggccagcttt
217681 aaaagcccca aaaaagagag actcattctt gctctccgga aagaaccagt tggcttagtc
217741 taaccaagaa agacatggga gttgttgggc gtaagattct ttttcttctc ttacgatatt
217801 tcgagttgga tgaaaagagc gcccctaaag taaagaaagc cctcgttcgc tcattcgtcg
217861 ctacgtacct ctattccact gaactcttac ctgagaacta ttatcagcaa tctctttctg
217921 aattatgtaa aaagattcct ttgcctccaa agttcttcaa tactttattt ttcttgcctt
217981 ttcagcttac tatcagtctc attgagttgc aggagagatt gaacagactg aagtgctgct
218041 ctagccatct gtactttctc agaacagttt gctccagact tcctattcag atcagagagg
218101 tcctttttca ccaatctaag ttttgcaaag actttataca tgggatcacc tttgacatca
218161 gtttgccaca ccttactcac aatagggtgg aactcaggga gtgggagttc agtcatgaag
218221 ttgaaaaagg gaggatatgc acccctgttc taaccacaca ggcactatga tcagagaggc
218281 ccttgggggt gaattcaact tcatattcag ataaaggcag agagccaaag catttaaaat
218341 tagcaagagc tctatccact tttatggaaa tacaagaggc ctcatcatct ttcttagacc
218401 atgtgacgaa gtgtcccata tatctgatgt cctcaagtcc tgcactttga agacatgaaa
218461 tcattcatag cagaggacca agaataagtc cctccattct tttcttccaa acatctaaag
218521 atgttgaaat ccccaagaac agacccacag aatcagaaat atccaacctt ccaaaccaaa
218581 gatgactcag aaatctacaa gcccaagggt actttaaaaa caaggaggac tacaagcatc
218641 tatagaccat taagccattg aaagtgctat agggaacaag atagaaagag cccctgctcc
218701 atggaaggaa gtgctccgtt tcgcggataa gaaagaagga actgctgaaa cctaagttcc
218761 atgcagaatt ccttttgcga atgaggtgaa agttaagttt actatagctt tttggcaagc
218821 gggaggtaca agtgtctgct tacctcatct tcacttcgtg cttacacctg ttgggctggg
218881 gccgggcccc caccttcctt cttttacgga aaaatgcaac tttcgcacca ggcgaaacga
218941 aaaagttctc gttttgatcg ctcgtcagga aggaaatcgt attacgtcgg tttttcgcgg
219001 taaaatgaaa caatttccgt ttttatcgac cgcttagggc agaaatcgct ttgctagctt
219061 ttgcaccagg gaagatgaaa gttttcagaa agtcagagcc ttctattagt ggaaatcgtg
219121 ttgctagctt ttgccccagg cgagcagcgg aagattgagt tcttcaagcg ggggagtcaa

219181 ctgcttcttc catagctttg atgatcgcaa gcctgcctat attgtgaagt tcttcgagga
219241 agatccgtca agtagagata tcgctgcatt ctctagtagt aggtagaagc ataaggacta
219301 gacctacata caaagaaaga aagctttagt gagatgttca ctaaggggaa ggctccctag
219361 ccatatacat atagaaaagg ttggattcta gtccttgctc taagagacct aaactttaag
219421 cctccacttg ctgcctacgc tagctagttt cctacctccc tctcggccta ccggtctcgc
219481 cagccttcct cttgactaag ccccgcttgc tccgtagtgc gcggattaag gcatttccta
219541 cgtggaaata agcactttaa gagtcttgag tcagccaatg attagcgagc aagcaagcca
219601 agccgcaagg aaagggtcac cgaagtaggg aaccgaagtg aaccgttaga aagcaattga
219661 catctaatag tcagaaattt agcaaaaaat cctttctcct cgcatcaaag acttctcctc
219721 gaaggcgaga gatcgaatca tagcctagtg ccatcttaat aagagtggga attcatcaga
219781 gtaggaattg gtataactcg tcgaacgaat aaggcggaaa gagaagcaga accgggggca
219841 tacttcccat ctgttcccgt gccaatgtga ccatcgatag ctttctctat tgaaatggaa
219901 tccccggtcc tatctttctc aattcatcga tcgacggccg ccctacttct aatgatagat
219961 agaaaaggcg ggagagggaa tcaactagga actggcatct cctggcccct tccaaccatt
220021 aaaggcacgt gcttattaca gcttaaacca ttacagatac aggggttcat ggtcgagcga
220081 gtactcttac tcatccgctg ggaataggaa ttggatcgat tggcgggtgc gagaataaag
220141 ccataagctg gttgctggcc gactaagaat agtatatatg aaagggatcc ccatcattcc
220201 taaaactcga gaagtcagag ggactttgat tgggcagcta ctctctagct tccagctatt
220261 cttccttctc ttatgggact tgaagccaag taaaggcagc ttgctgttga gttctagtct
220321 ttaagatatg ggacttaggc gggaaacctc agtgaaacaa actagtaatc taattactca
220381 tctaatctta ggcttcgcta cctgacttat cagccagcaa gtaaactacc ttaaccccct
220441 gaaatataag taaggagttg gcagcctttc gagctaggag ttgaaccaag gccgctattc
220501 cggacttaga gatagtagtt cccaagtatg atatgaaata ggaaggaatc tagtaaaggc
220561 cttgtcctac tctactctaa tacaagtaag tagctaacgc taccttaatc ccctgaagta
220621 aggatttgga agctcaggag caagagtcac tgtagttgct acggccaggg gagctagtcc
220681 cgtaactgcg ggcagttcga gttctagttc tttacaagac agatgggaac tgtgatctta
220741 ctccatagga acggaactta tacccagttt gggtggagct tagaccaccg gatctcgtct
220801 tttgctgctt gaatggccga gaggtagggt aagttcttag gaatcgattg aatcacagga
220861 aatctcagat ggacagaagg agctgcgaaa tcaacaggaa agagacgacg agccagacgc
220921 ggattaaatt agaactgctt ttcaatgcct agtttcgtag ccaagcaggg ggaatcacct
220981 tctgacctga ccttcgacct tgaaggtggc aatctaatct agcttccgtt gaaaaagcaa
221041 ggtgcggagt caggactgag cacttctttg aattgagaag aggtgcctac ccttaattag
221101 attaggcgcc aataaaatga agagatcaga cttcaaggca tagggccgtg aggtgctttt
221161 agcgcagccg ggattgatta aggtagttgt tcagccgagg gaaagaatca ttcaatgcta
221221 gggaccaggt tactgagacg gataaaaggt tagacccatt cacggagtag tagtttgggg
221281 ctttcaagct ggctctttct ggcatggacg tagggaagag tacaacatgg aaggctgtga
221341 cggaacatag tctttacacc taaaggggct tagtgaattc ttcttcaact tatttaggta
221401 agcactaagg gaagggccca gccagtagcc cattcgttga caatggaaaa cctgatttcc
221461 aggcagtggt gcctcttggt ccaggtcttt cccactcata cataaaagat tgtccaaaag
221521 agagtgtcga atgagaaggc tgagcgctac accttgagag ggacattcgg tgtcgtgaac
221581 gaaagatttc cctgcttttc ctgatgcaag atgcgctgct gacgcgagat ggactgctgc
221641 tgtgagagaa actttgatct ttatccatct caagaagtca atgaggaaaa aagggtcttc
221701 tccagtaggg aggcctccaa ggacaagtcc gcaatggaag aaagacacgg ctcttcgaag
221761 cgtgacaaac aaatgctgta aatgagatgg cccagaatgc cgatcaatga ttgctttcaa

221821 gccagaggcg aggggcaaca accagtgccc ggggatgatg ttgatgctgg acccagcttc
221881 ttcacccgat gatcaaaaga gattcccaga aaactaggac ctgagagagt ccccgttgga
221941 aggcgaataa gagatcaagt tttggtgtgc atgacccgaa caagggtaag atgtctagcc
222001 aatcagagga gttgttgcac agcgtccagg ggcttccacg ccaggcttaa gagttattct
222061 cttaagcggc aaagaaagtt cttcccaaag acgagtttca cgccttctac agggatatgg
222121 tcttatcgtc tgaaagagtc tcctttcagc cgggtccctg ttagctactt gctttccacc
222181 gtcattagac taggcttccc ggatccccct ttcttcggtg ctgacaaaga actagacggt
222241 taaaaaattt ccccacaaaa gaaatatcca tcagctgagg actttcgcaa tggggattgt
222301 tttcttttga ggctttctta ctggataaag taatgttacg tacgctttcg aaggctctat
222361 gaagagaatc ttgcagatct ttattccagt gccaattcgg ttggagatgt ttgtcccaat
222421 tccgattcga tttctcctga tccagcttcc atactttcat ctgctactga gacaattgtc
222481 attgcctttt ttttcatttt gcacctacta gcattggtcc agcagtgact ttgttcttcc
222541 cacgacacgg ctgaagcgtt cggttcatta gatactgaga aagattgttt ccttagttgt
222601 ggaattcccg gctatccttc catccgccct ttatcaagct acaagtcagt ctgcccaatt
222661 cttttgccct ttactttccc tttaggtagt tcatttcggt taaatcgata gagggcaggt
222721 cccactgttc ccgggcaatc aggaagtcat tgcagctaaa gcagggaata catagactat
222781 aagaagtact ggtgctttaa actccttata tcacttgtag ttcactaagg agaaaggctc
222841 gacttccggt atatgaggag ggcgagggta ggaaagctcg ggagttgctg gagttatagt
222901 tgtataggaa gaatagacaa agcctgtgct tctataccga tctgcccggg gatgagaaag
222961 agaagaccag ttctccttgg atgcacccaa agcccgttaa ccaaaaggcg aatagttagc
223021 cgaggaaggg aagaccagtt ctccttggat gctaactcac cttataagga gacttcttac
223081 tgggagttca ctaagtttcc ttaagaagcc cgaaggtatt gatgtgagac aaaggactgc
223141 cgctcgactg tacaggaaag ggtataacaa aggtggtgag aacttgtgag actaaaggat
223201 gcggaaacct cgatctaagg tggagtagcc cgtaggtggg ttttgttcga gagggagtgg
223261 ggactaagac tgataaggag ttaagaccaa tagtcagttg tttttgtgtt acgacctaag
223321 gttgtaaagc tagaccaagg gtaagggtag cttgcttatt gcttgtgcta aagtagtgct
223381 gcctcgaccg tagttgacca ataaagagga gtctgagaaa gaagttaaga ggtccgatta
223441 gcaccagtct tataaccagc ctttctgtaa tcccttatct aactgaaagc ttcctaacta
223501 agctaagtaa gtaaataaag agaggtggtg ctgtcctaca cttttgataa ggagagtttt
223561 tcacctttca acttatatgt atgccgtaag gggataggtt cgtcttatac ccttcttttt
223621 ttcactttag gtagctagtt ttcctccctt atattcgact gtaccatagg gaaatagggc
223681 tttctatcga aggtgagtga gagtgtatta cggcatgaaa cgatcctttc catagaatcg
223741 gtagagttcc ttagatagta ctcgatctct ggagcgaagc ttgctcgaag aagaggagtc
223801 ccaggatccg gctagtatgg ctttcctcaa gcttatgaat gctttgaagg ccaatccact
223861 gactcccaat cttgtagatt gagatctcat ctatgtcgag ggaaaggtca acggcaggac
223921 tgcccatatc atgagtaaat tatccatcta gattagatag gggtagcact cataccttca
223981 tccgtactag tgagaaaagg aagacgaatc cgcgacagag aatgggattg atgttaagct
224041 aaggaatccc ctactcatcg aaagagggga attggggtgt aaagaaaaaa ggagaagctc
224101 tacaactaga accttttctt tgatgttaca gcctctccct cagcgaggaa atagtagcag
224161 cagcctcccc taaaggttac agtcccaacc aagtcaagag tccggtccga acaaaggcat
224221 tagatgaaag ctgtcccatt tacccattca aggaagggat tcgagttaga ccacttttaa
224281 gcaccttctg gcttacaaga gaccttccat gtaagatatc ttcttagact gtctggttat
224341 ccgaaaatgt gagtctttgt ttcccagtcc ttagaaggag gagagcctac aatgtccgaa
224401 tgttcgaaat tacacattca ttgttggtct agcgagcctt cctagccgcc tagagtgcag

224461 cctgcctgcg gaagaccgta acgtaagtaa ctctgtgctc ctgacggggg aaatgaaagc
224521 agaattcgtt cggatcctct cacatgttca atctttttt agcggtttcc ccagagatct
224581 ttctcattaa tgcaaccttc attttgctca ttcatggagt tgtatttagt acgtctaaga
224641 aatatgatta tccaccgtta gtcagtaatg tgggttggct tggattactt agtgttgcgc
224701 gcctaggagg gcagcgcgct ttgggatgcg gagcagctat tatcgcccag ctccctaacc
224761 taatgcggca ccgggttcgg accgcaggga accgtagcat ggggtaacgt ctaatccttt
224821 tgccgccgca aggctggcta atcgtacgca gcaggctcga agaccсctgg ttctggaagg
224881 cacttgagtc cgaacgctat gttgaatgtg cgaccgacac tacgtaggta cctgtgcagg
224941 tgaggcgtcg gtcggtccta gaaacggcgg cagcggcgcg aggagttaac gaccgaacgt
225001 gctgcaacct agggatcacc aggcagctct ttcgctctat aagggatcgg gggacatagc
225061 acaattcttc ttggaggagg gttggtttgc ccgggtgact gatcctgcca taatgtactc
225121 ctacctataa caccgccaac cgaagtcgag catacatacg acgatcttca tgcgtgaata
225181 gccgggagga aagaaagggc ggtagatgat aatgagaaag ggcgccgcgt ctgggcgggc
225241 ggtcggaatg gatgagcaaa aggtgtcatg ccatggagtg gggaatatcc cgagtctcat
225301 gtaagacaac taaatgcgcc tcgaggtgct gccgaggaac tgagggacct tatcgagtac
225361 aggataggta attgagagag agagctagcc tgttcgttat ggggaatcaa tgctccgggc
225421 caaatagagc ttacctacgg aacctggctt tttccggcgc atattaggtt agctgcgctt
225481 aataggccgg tttggcttcc tccctggcgg cctccttccc cccgctacac tctcactcca
225541 agctacgcct gctgagcaag atgcattgcg atttcctctt ctgtggacgc accggagtat
225601 gacccgggac gggaagagaa aaactttttt atccggcgag ctttctctcg taaagccaaa
225661 gacgccccaa gacaaggtac aactgttggg aaggggacat gatcaataga acctggctgg
225721 atccgggctg ggatagtggc gcccattcct aaccagttcc gaaaaggttc gctcatgctg
225781 gagggagcat ccccacttag tgatcggtcc gctagttcac gcaaatccga agaagttatc
225841 acggacgagc cacatgcagg gaaacttgca cgtgtggttc tggccgggct ttcctgaggt
225901 atctaataac cttgcttctg ctcgccgctg gcgcacctct tctaactatt gcccatttat
225961 tctggaataa tctttttagg agggacaatt ttacatattt ctgccaaatc cttctattat
226021 taagtacggc tggtaccatt tcgatgtgtt tcgattcttt cgaacaagag aggtttgatg
226081 cttctgaatc cattgtatta attccacttc ctactcgcag tatgctcttt atgatctcgg
226141 cttatgattc aattgccatg tatttagcta ttgagcctca aagtttatgt ttttatgtga
226201 tcgcagcatc aaaaagaaag tctgaatttt ccacggaagc tggttcgaaa tatttgatct
226261 taggtgcatt tccctctgga atattattgt ttgggtacga ccggacaact accgatatct
226321 attaatatat tttcttagaa tgttgttgtt aactatatat ctatctatat cgtaaactat
226381 cggatcgggt cttacttaga tgttaaataa acttgcagac ctcattggtt gtgatattta
226441 tcttacgggg ggaacgaaat aaaagaatat atagacttgt agagaccctc tatgtagttg
226501 tctatctagg gcgatcgatc tacatctaca tctttcccca tagccctggg gctgtgtctc
226561 gatctcctat gtgcgaaatc taagggactc gttcctatgg agattcccct tggtctaatt
226621 ccatgcctta tgacgaaagg ggagtcggcg tggcgtaaag tgaagattgg gggaagtaga
226681 gaaaaattcc cttctggggt attccgaagg tgtaacctag gcaacgaaaa aggggcccga
226741 tacttcaact agaggagcga cctgttggtt caccaaaccc cgacccggcg tcacacgttc
226801 tccaggtccg aagggatcca gtgcccaact accgactcct cccggaattg cgttatcgga
226861 gccgagccag gaatggccgt tagtggacac ccaccacttt tcaccggtta gagaggccct
226921 ctttaataca ttaagaaaag atgttcacag gggccagaaa gactcggtca taggaactta
226981 gaccacctac gcgctggtaa acgaaaacca cctcgaccgg atcagagtaa acaacaatgt
227041 cgaatcaggc cgccccttgc cttgaaagaa ttcacacgcg gccaccagct ttccaaaaag

227101 aaggggagat ctgctgctta ctgctcacgg aaacactaga tttcttcatt ttcttcagta
227161 ctctttgggt agagagtaac atccttagcc ttgcacataa aacgggaagt attctctcta
227221 ccacaatgat acacatcacg atcatagagc caaggtcgac cccataatat gtgtgtaaca
227281 ttcatgggaa caacatcaca ccaaatagaa tagaatcttt agaatgacca gaagcaatag
227341 aaactaaaca gcgttgagtt accggtatgg tagttttgtt gatccatgcg acatgatatg
227401 gttgtggatg tggctcggta ggaagcttca atctctcaac tgtagaggct gaaaccacat
227461 tcatgcaact cccaccatca atgaccagct tccgtaattc tttgccgcaa gtgacaagtg
227521 tttgaaagat ggttgttcta ttccaatctt ccttctcaat ctttggggca gcgagagccg
227581 aaccaccata gcaagggagg tatctacgtc agaatccacc aagtcatctg cattgaactc
227641 tggattctct tcatcttcct cattttctgc tccttcttgt tgaagctgtt cttcttgttc
227701 cacttgtagg ctggactttg ttgggagagg tgttgtccta gcagttcggt tgccatcagt
227761 aagccttttt gctgctatct ccccagcttg gtaagttctt ttcttgacgg ttggcacccg
227821 taagtactcc tccatatcta aggccacttg aaaagcatgt tccacactat aaaggggttg
227881 ccgaagcaac tctttcctta tatcgaacct caggccagat tttaatctag ccaaagtgtg
227941 gcgaggatct tcaacaattt gactccgagt cttgagttcg tcaaaccttt gcatgtactc
228001 cgctactgtc atgttgcctt gcttgagatt gaaaagttgt taaaataact tgtcttggta
228061 attaggcggc atgtatttct ctcgaagctt tgccttcatc tcttgccaag tagctattgg
228121 tggttgtccc ttcttatatc accttcaaaa ccgttccacc acaccttcgc gaggctcact
228181 agcttcatct taacgaaccg aactcgccgc tcgtccgaca tgtcatacca atagaaagac
228241 tcctcaatcg cagacaacca atcggcaaag gttgttggat ttagtctacc atcaaaatct
228301 ggaacgtcta ctttcacctt ctttgtgatg tcttcttcag tgtcatgagg ctcatagtaa
228361 tctctttcaa gcaacttcct attccagtct aatctaagaa gggagtttgt ggcgcttgtg
228421 ggggcctgcc aacccggact tcccgtctcg gaactggatc aggtggggct ggtccttttg
228481 ttgttcacct tgttcagcat ctccttcctt attagtcttt cctccggatg attctcctga
228541 ccgccaagtt tcctgtaaac tgaggctgct gcttcagatt ctagatctcc cacacgagca
228601 ttcaggctac ggagttgttc attcatgctc tggaattgct ccaccaactg tagtagcaac
228661 tcgtctcgtt gataatcggt taatcttcct cgaaaggatt ttaccgtacg agtagacatg
228721 attcgaaacc cgctctgata ccagtcacca agcatacact cgtcaacagc tgcattacaa
228781 ttcattggga tttgttcgcc tgccacgtga ccggtttcag atgtctccat ctcattaatt
228841 gctgctaacg aggcaaaccg agaaccagat aaggctggat tagaactaga tcctgggtta
228901 gctctttgag agcgaaccaa ctcagtcaat ttttgctttg acacagcccc cactcttggg
228961 tattcgacca ggtataggta tacttgctgc cctcataacc gcagtgaatg atgccacaat
229021 cattaatcat tgcattgaat tcaaaaaaat atacaagatt tggaggtctg caaccaagtt
229081 tttcagacac atctgaaatt gcgttaaaat cacccgcaac catccatgga ccttgtatgt
229141 cttaggacag taatggaagt ttttccaaaa gaagccgttg aatggagcac tttgcataaa
229201 gaacggtaag ccttacccga tcagaattaa ggaagctgat gtcgactgtg atacactgtt
229261 caaacgcgtc gacaagcact acatgaactt catgattcca acaaagccat attttatcat
229321 ctgcctcttg gtttgccaat gaaaagttga aaccaattct gttggataat tcctgaatct
229381 tattagcatg gtgaagaggt tcaagaatag caatcaacga tatattatac atctttaaaa
229441 gcttgccaac cctccttctt gatttgatgt tgcctatgcc tctgatattc cacaagaggt
229501 tcttaatcat ggctaacaaa acaatggaag aagcttgatg aaaagaacga gccaacgctc
229561 tagtaatcct tctggagctc ttcaagttcg attcttgtac tttttgattg aagatgtatc
229621 tggttgctcc ttgtaggatt cagattaggg tacaatcagt gttcttgaat atcgaagctg
229681 tagataacac ttgagagaat tccttacaaa tggtaaaaaa ttcctgaaaa aaaagggggg

229741 tttgcttctc tcataggttc agagtccgat aagtctccct tggtctgagc agcttgttcc
229801 tttggaacgt tagcatcatc tttggatttc agtcaaacaa gggatatatt agactccatg
229861 ttgtttatga gttcatgatc aggctgagag agaataggca taatagcctg aatactataa
229921 ttatcaacat tggaattgtt agcagccaga gatgagtcaa tggcatctac tattacctga
229981 tgagaagaat caaccctcaa ctcagaatca tgagaagcag tactgcttga aggatcggca
230041 gaaatctgta tgtgctgaga agtttcttta ttaaaagaaa aaagattctg ctccgtctga
230101 ttgtccgcag tattgtttgg aagataatat gtattatcaa aagatgattg gacggtgttg
230161 cccgttggcc tgtagacttg acgtggggaa gcttttcctc cttgaccttg tgcagatttt
230221 ttcctagagg cattagagtt aggagctgac ggctgctggt tggacttctc tttgttggct
230281 gcatcagttg cactggagtt tttcgaacga agtttgcagt tttactttgc atggccttgt
230341 ttggaacaag acgtacaata ccttaagtcc ttctcataga cgattttctg ccatcttcct
230401 tcgttttgtc ccatgcctaa ccagacccta ttagtatagt aggatttttc ttgagaagat
230461 ccacctcaac gcatactctt gtgacaccgg gtcgagaaag cctgggggtc ggaccatcga
230521 ttaacagcac tttgcctact acgttagcaa tagatctaag gtagtagtct tgaaaaaaac
230581 agatcggaag attcggaaac tgaatccaca taggaacgat tgaaggttca gcccccgatt
230641 tgaagtccgc agaccattta acgaatctga agagataatc cttgatgtac gttgcatctt
230701 tcaaccaaag cttaatgtaa tcctctggaa atgttggaat atagatctca ccattagtaa
230761 taaattttta catttttgca atgcaaatca tttttgact tttcttctaa aaaaaaaact
230821 agaaggaaag atcagagagc ttcccattcc ggaaatggag aaagatgaaa tgccagaaag
230881 cctccaggga gaagaggcat ttgagacttc tgtctcccct tccagttatc aaggaagaac
230941 tcgaacaaga gctcaaaggc attcttcttt ttgttggttc atagtccaca cggggttctg
231001 gtcttgtttg ttactttatg ttatgttccg gagtcaattg atccctcgat caaactagaa
231061 atctcttaag ataagaagag aaatcgtttt gattcgaggc gaaacccttc tccgccgtta
231121 cggagttctt ctgctctaat ctccgaaatc gaaggtcagc tgactgagga gttagtattg
231181 attcatgcaa agatgctcct ctaaagctag aataagggat tgtccacctc accgccccga
231241 agagcagtgg ctctgttgtt ttgcatgcct acagagagat actaaaaaag taccgacgct
231301 caatcgaaag aaagatcaat ctactatatt cttaactcat gccccaggaa tctctagaca
231361 caggggggtac gagcgaatct tttactggag ggaaagctgg ggaaaagcat agagcgtgcg
231421 ggctcagctc tcgccctcct cccatccgcg aagctgaggc gggagaaaaa gcgtaactcc
231481 ccggtgtcat aggttacctt tctatcttcc tcccccgtga cgctcccaaa ccaatcgcta
231541 tccttttctt gctttcttgt tgtcttgact tgttatagaa cggctttttc tcaggtatag
231601 ttggtaggat gaagcgttag tggatatagc aagtgtgccg gggatgcggc tcgggcgtag
231661 tgggtctgga cgcctagcat gaaacagcct tctctggtag cggcactata ccttagtctc
231721 tctctagctt ccagtctata taaaacgtag ttagcagagg taagtctgtc tgacgttccc
231781 tcgcgtcaag ggctatactt ttgcatcgac tctctcgtta ggtaattccc gaggcgaaag
231841 cagctctctc ggaagtaagt ttccccgcca tcttagtggg actagtataa taaactttca
231901 cttgaactgc caagactcgg tttttgtgg taacgccctt ctagaattac gtttaacgcc
231961 cctttatgac tcggcgcctc gggtcggtag ccgggctccg ggacattact accacggcta
232021 ctatcgaccc gagcccaaag aaggaaaaga acgaacggac taaagcgagg agttcattgg
232081 ccgtacatag ttaaggagga tgggggtaaa cctctttcat gacccatggc cccggtgtgt
232141 cacttggtta gcatttctag aaaaaaaggg agtagggttt gtttttcgat agggaaagag
232201 acaggagagt tggctgtaat tgatacacct ttttcggatg gatttcttcg agacggttaa
232261 ccacttttta aaccgtgaga gggaggtcat tcagaacccg ctggctccag aacggggggc
232321 cagcaccgac ggaagtagcc caccccgcta ccgataaaaa agagcgcgcg gcacttcgaa

232381 gggacattca aactttgatt cgtgagcaac ttcacgaaca ttgtgaaaaa gggaaagggc
232441 ggctgtccgc gcactttccg gaaatgacgg aaatagactc aagtgccgcg aaggcgataa
232501 tgtcttacga tctggagatc cccgcggaga cggatacgga aaccccccgc agatgggcgg
232561 agaatagaag gggggaccct aatctcctaa ggccactcat tagggaatac cggtcaaatt
232621 agtctgtctg ccgctttgtt tcataacaga gccatttttc ccggctggca tagaagtcta
232681 ttgcgcgggc gaaggagatg cgtttctggt actggtggta ttggacaagc tctaagggaa
232741 taatctcttt cttatttctg cctttctttc ccatgacgac taggaacggg caaatcaaaa
232801 atttcacttc gaatttcgga cctcaacatc ctgctgctca tggtgtttca cgatcagtat
232861 tggaaatgaa cggagaagtg gtggaacgtg cggaaccaca tattggatca ctccagtgcg
232921 gcacgaagcc gctgatgccg agtcggctcc tatgccgcta gctatgccct gcttggtccc
232981 ccggcacggg tggaggttcc gtagcgcgtc atgagcaccg ggctaagggg cggttgagca
233041 actcaagcga accaccctac cttactacaa catagggaca gaagggagaa ggttgtgaag
233101 gtggcctcgt tatccacacc tccggtcgga tgaatggagg accggccgac ccgggttttc
233161 acgagcgttg gcgggttctg gagtgcctgt caagggcgct agcgcatacc ccggggtgat
233221 catcaccacc tgcacctcac atctcggcac agtggaacgt gtaacccgcc tgctgttcca
233281 ttcaattaca tttgttcctg taatccatag cctaacagaa cgcagcagcg agggacaaac
233341 ccgcctatat atacagccag cggggaggat ggcactactg gcaaagaccg tctggcgaaa
233401 acgccgcagg cgcgaagcgt ggtaggcctg cgccgggggga gcatagggag gaaagggagc
233461 ccggacggtg gaagagccag gggaggccgg gtcatttgac ggaaatggag ggcttttccc
233521 tattagatta gagaaggccc tatggaggaa agggaagtgc atgaattctt ggaaaaagag
233581 ggagcgagcc tatagaaaat aaaaaatgta ataaagaaaa ttctatgaat agagtcaacg
233641 gtacgacaga cagcgctgcc tacacgcgaa ttagctcccg agatcgagca gtctcaattt
233701 cactacagga tttacgaatg aatgctggac tgggccacct cgaatggcgt gagccgcatg
233761 cggggagacc cgcacgtacg gtttttaggg gggtctggtc gaaagaccgg ccggcgccca
233821 cccgactaga gggactgaga aattaataga gtacaaaact tatcttcaag ctttacctta
233881 ttctgatcgt ttagagggcg atcgcggggt cactgaatga agtcctccgt ttctttcggg
233941 ggtgctgacc cgcagcgagg cagagatgac taagtgacat atggaatatg gcgacgacaa
234001 cagcatgtcg tagaaggaga taacaagtgg agccaacgac ccactaagac taacgtatct
234061 acaactacat ccccgagcgg cagtcaaagg gggggcgtga atgcgagatg ccagcggaat
234121 gatcggccgg acagaggcta gggctgcttc cttcccaccg cgtccttcct tgtgtgtcgg
234181 agatataaag cgagtgcacc ggaaaagaaa agggaactgg gtcgatctat tctatggcga
234241 agcatccgaa gcataactgc acactcacac gatctttgcc gagagatagg agcattcggt
234301 ggaaccggtg aactacactt gcttctggat agatgtgtgg gacagagggc tcgtggtacc
234361 tgctgcccaa ccttcctcct ctgctttgag aaccgtgtga acggagagtg ggcagaaggg
234421 aaggaggtcc tcatacggag tcgcacactt acttgagcag tgcgggagac tggggaatgg
234481 gtcaagtaaa gtcccctggg gccggaaaaa gaacagacga agctttactt agatagggct
234541 tttcttggtc ttttttttt tcttagtgat tgaaaaggag agcgcctgag tatgttatag
234601 aaagggaagg cagaggcatc gaatggcgaa gagaggcggc tcggcaggga aggaatggga
234661 aatcgtcaag gatgacttct ttgttggcga aacgaagggc taaatagcgc cagtgattct
234721 ctgaaccggt ctggatttcc aacgcctcag gaaactggtc gagatagatg acagcacctt
234781 tttctcctct tccttaccgg gagggcaatc ttctcttatg gccctcacct cccgcccggc
234841 ccggaaatcg agaacccagc cccctttga tccattcatt tccgcaagca gggaggatcc
234901 agagctaagc ccccgcctat tcgaggccgg gcggcctcgc cccacaagca cccaaccttc
234961 cttttgctct tccttcggtt tgcctccacg aacgcgccac ctaggagccc tactcatatt

235021 ccaaaggcga cctgctttca atactagttg ttaccttacg ccgaaaatta aaaaaaggat
235081 tgaatagcat ggcctggggc taagataact caagtgggag agccgtgtta tgggtgacct
235141 tattgcacgg ttcagagagc acttgtgtat gtgatgcaag tgaacgtgta cgaaaaagct
235201 gtcgtaaagt ttcgttgttc gttccgtcgt tgaccctatc tatgtttcta cgatggccca
235261 agaacacgct cattcttcag ccgtagagag acttttgaat tgcgaggtac cattacgagc
235321 tcaatatata cgagtgttat tccgtgaaat aactcgaatt tcaaatcatt cacttgcttt
235381 aactactcat gctatggatg tgggagcatc aactccgttc ctgtgggctt ttgaggagcg
235441 ggagaaattg ttggaattct atgaaagagt ctcgggagcc aggatgcatg ccagtttcat
235501 acgaccaggt ggagtggcac aagatctgcc tcttggctta tgtcgagata ttgattcctc
235561 cacacaacaa tttgcttctc gtatcgacga attagaagag atgtcaaccg gcaaccgtat
235621 ctggaaacaa cgattagtgg atattggtac tgtcactgca cagcaagcaa aggattgggg
235681 attcagtggt gtaatgttaa gaggtcgtgc gacatgaaga cattgatagc aatatggggg
235741 aagttcccat caggcaacaa cggttctgcc tgaccctact aaagcatgca tattatgtca
235801 gtgaagactt ggtgtgaagc cttggagctt acgttagaag agcaaaagcc ccggcggggc
235861 tagggtgagc tgagggggga cagcgtaagt gagcgaatgt gtgtaagccc agtcaaacat
235921 gactgttcta ggcggggcgg gccacccacc ttcgaatggt gttggtccta cggaccgtga
235981 acggattcgc ctctggcctc tgggcacgtc ggaaccgcat gagttcaccg gggtggagca
236041 cggtccgcca aaatcggcat aggttaggtg ctattgatgg aacatggtaa gcctatcttt
236101 ctccatatgg aagtgctgcg ggcacataga gatgtgggta gaggaagcct caaaaagcga
236161 aggccgagct gtaggtcacg tgacctgcac cgagttggtg gctgactggg ctttttatgt
236221 gatcaaagcg gataagctcg ccttcttgtt atccaaaagt aggtcgaatc gggcgggccc
236281 ctgccccgga acgtcgaatt gaataggtgg ccgggttctc tttctacgac ccttttgata
236341 tgataggccc ggccccgtc cccctatccc ttatgtttag gagcgggatc cacccaagaa
236401 cgaccattct tgtggtggta cggaaggcac ggactccgcg aacgtcccgc gcccctttac
236461 taataaggga aagaaagcct caaccagaac cacattcctt ttgcgtgcgg gtgtagctaa
236521 gtgtctgact ctattggtca tagtttcctg ctgttgcggc cggtgctcgt ttgcgcgcgc
236581 gtgaaccaac ccaacaaaca aggaaaggat gcctctcggg gcatctgaga atgattcgag
236641 ccgtatgaag ggaaactctc acgtacagtt tgttttttg ggggggaagg agcccgacag
236701 ggtcccccca cttacttggc ccgggcctaa gtgaaagtta agtggtgggc ctacccatcc
236761 caaccagggg tatgctggga ttcgcgaaga gcagcacctt acgatgttca tgaccaatcg
236821 gatcctgacg taccagtagg taccagagga gatcgctatg atcgttactg tattcgtatc
236881 gaagagatgc gacaaagtgt tcggatcatt gtgcaatgtc ctaatcaaat gcctagtggc
236941 atgatcaaag ccgatgatcg taagctatgt cctccatcac gatgtcgaat gaaactatcc
237001 atggaatcgt gcgccgtgtg aaacgtagat catcgccgtt cttaaccgag actcaggtta
237061 agctccgtct cggaaccttg tggggttagg agtaaagcat cccgaggttg gcgcatctcg
237121 ttgagcgtgg agaagcattg ggaaccccaa tttctttctt cggagccgtt tcttttctcg
237181 tccccccgcc ccggcatagc gcttcgcttc cggttcttcg gaagaatcaa cttacttcta
237241 ccttcttcat tgatctgggg gaaaaggaac cgtctaccag ttgggaagct agacatcaag
237301 taagtggctt gatgaggata actaagcgga cacgccggag ttggctgctg gcacaacagg
237361 gtggtgcctt accgcaccgc aggcggacgc gcggtagcgt tcgtggtggt acttcaggat
237421 tccaatgtac tgcgtccaag atcagaacga gcttgccggc ggaccactgc cgtcccattc
237481 ttgagtgagc tggagcgcag ccatcttatc cactgaacta gctagaagct atcgcttcgg
237541 gtcgaagcac taaaagaaaa ggaccgggaa aggcggcggc ataggaacca cgggaccccc
237601 tactagtaaa gggaaaacgg aagtgcgctc ctgcgcatca gctgaaaaag ccctttcccc

237661 tttctctaat aacgaagaag gtcagcttca tagctccaac ctatacaagg ggctttgatg
237721 ccctttgtat aggttgctgg atacgggatc ctcgtagtag gctggaccaa catccagccg
237781 agagagggca gtctttagaa gcaacaggtt gggaaaccaa gagaacgccc cgcgttttat
237841 tctcttcttc ccgccctagg agtagcacaa aaagagggat tagcattatg gacccaatga
237901 taaaccacta aaaccttact cgttggggct tcgcgcactg ggaaaacgct ttagcgacgg
237961 ggaaccagtc actagttaca aagctccaat aaggtaaggt atcgagaggg ctatcacagt
238021 caggtgcgaa gcaattagcc ctatttggaa agtccccctc ttcctattta gggagttgag
238081 gcgaaaattt tattgatgaa ccgttccgtt cgccacgcac tggacccatt cacttgctta
238141 tcgtagaggc tgtaagtaca cagtgcccca caactattaa taggccgtgg ggttgaaaga
238201 caggagtgcc cgcccctcca ctcaagtagg ctactttttc cggaacgcag tccgagataa
238261 ccgtaaccgt gtatataaaa aaatatctct tcgatgctgt catttcgaaa tgtccgcttc
238321 aaccgctgtt tcacctccca aagagcaaag ttggcttgat gagcgcagat gaggaagcgg
238381 gagcaagaaa accaataata tctttcttgt ccttctactt aaggggcaaa gagaagcgct
238441 tttgctactg agaaagcgaa cggtcagcaa cgaaggttta agacttagcc gagcgttagc
238501 gaagctagat tctcatagcg aggcgcttcg agttagcgaa gcgctgtagt agcgccgaag
238561 cactatgtgc tataatgctg aaccaaggac gctccgcctt atctatagaa gcagtcaact
238621 gagttctgaa cgaatgaaat tagctccttg gtaatggctc aatctataga tagaaagccc
238681 tatgatggga aactaccacg ttaggtttgg agagagatgg gaccggttat ataatagggg
238741 gagcagatgc aagctttttc tttcaatagc cggccaaatg actacaggat catcggtcta
238801 ctctacctca attcaccatt tcgaacctta tacagaaggt ttttccgtac cagctccttc
238861 tacctatacc gcagttgaag cacctaaagg agaatttggt gtctttctag tcagtaatgg
238921 aagcaatcgt ccctaccgtc gtaaaataag agcacctggc tctgcccatt cacaaggact
238981 cgattctatg tccaaacatc acatgccagc agatgtggtc accatcatag gtactcaaga
239041 tattgtgtct ggagaggtgg atagatagga ctactagttg ctcgatcagg cccctagctt
239101 tattgcgagc caaaaacctt gatggattcc ccttcccctt ctattctcag tactagagat
239161 gaacgaattc catacgcccg aaattcagat cgaagaattt tctttatgtt gttggtattg
239221 gaatttcatg aacgcggagc caacgagttc agtcgaacag cttatatagt aaatagaagt
239281 ggaagagcct ttcgttgata ctattctatt gttgcggctt ctggttgttc atgctacact
239341 ttcaattaaa aaaactaaga gtagcccaag gaataaggcc tatttatgag ttcttgtatt
239401 gctttcgcca ccccttggtc gccaatgttg tcatagctcc tatgaaagca aggttacctg
239461 tgttgagcta aggatagcaa agagcagttc agatcatccc aattgcctgc taaagctaag
239521 actagttgaa agaaagaagc ccccactctt ctatgctttg aagtgaagcg acttcgttca
239581 ttcccgagtc atcaataaga ttcagaggat ggaaccctat cctgtaatgg cacatcagct
239641 taccgattcc ttaataagca ttcattcaag cgatagcaat gaggcattca agctcttctc
239701 tgcgaaccga tcacaaggtg gatttactat gatgtatgat gaattgggat ccgcgttaac
239761 caactcgcct gcaatctcgc tttcagtctt ctcgttgtca tgaaacattt tgactagccg
239821 tgtgtgtgca gtggaggaag caaaccaata ttggatatgt gcgcaagagc cttgtttaca
239881 ctttcttgca tgctcagtag tcagactcct cttcttttta ttgaatgagg ggagcccctt
239941 tggggcgaac gaatgctagt atcgatgtct tgaggtttaa gccctgatat ctattcttcc
240001 cagtgccttt gccgtagcaa gcctaagcat aataaatcat ctgaatgtac cctcattctt
240061 tcttggtagc tagtcttatt cccgatcgat ggagaggtaa ctaaaccata tatctgactc
240121 ttcccatcga taacgcaatt agagctatga acagtttgat tgttcttggg caacagaatc
240181 agtaacacaa tcaggggtac cgggcttgcc tcaatatcgg gatagaagga ggtctaggaa
240241 actaggctgt tgaccgggct aaggtaacga agttagaccg agatgaacga ccgacttcct

240301 ctacaaaaga agttgttgaa taagatgatg ggatcgcccc ctgcccatat tagctaaaaa
240361 cgaaggaaga ctaaagccgg ggaggagacg acttgcaggt tatttcccgc ctattatgcc
240421 taacattaga attacataag agaagcaaag ttcacagaaa gtccacgcac gaaggagaat
240481 acccccegcc gcctctcctg attagtcgag ataactacgt aatgtgcccc gttcccctac
240541 ggcgattggc cccttccccg gaaaggtgat tatacatata gtaaaaaatg agagagaaac
240601 ttaaaaagaa agaagagaaa gaaccgggag ttggcgccta cataactgct tgcttgctta
240661 ccaagccatc ctggcaagct cctccagttc gcttattatt cttgacttga cttattatta
240721 taaaaactac tcactatcaa aatgaaagac gatcgattat ctacccaaga agatagagag
240781 tcccacatac gagaaaaggt cacaaataga gttgaaccaa gtaacattgc aaaggcataa
240841 tgatagtagg gtcgggatat ccccgccctc cgaaccggac gtgaaggtct cccctcatcc
240901 ggctctctgc aggggaatct ccactcactg cttcccctaa tatcctccct ttaccacatc
240961 atggggggttt acaggagatc ccagaggctc gctcggaaag gctgctatac catacctttt
241021 gacttaactc tactctagta gtcactagac tcactatagt agtccgtccg gctgctcttg
241081 ctgaaatcat tactcttcat tatcccgtgc tctagcaatt gtgctcccta gaccactacc
241141 gtgtagttag gtagggacaa tcaatccgta gtgacgggaa tctaggaatg aatggggatc
241201 cctattcata taaaaatgaa gaatatctaa ttagagtttt ctccctttct ctcactcaat
241261 agatgtatct ggtctgataa ggtacagtac aatacgagac gatggaatgc aatgggatag
241321 atggtagagg gctgcctgcg cccaaaagcg atgattcact tgtccccttg tccataggga
241381 cctcgtggca tacaaccgaa acgactcccg ctagatagcc gcccctttct ctctttttac
241441 agcctcgtgg acggacgaaa gaagggaagt tacagaactg ggcagtgaag gctcgcgaag
241501 tagacagcaa gcagcaagca acagcttctc agccccctaa cctttctatt agtcaagcgc
241561 taacgagcaa gaaaacggat gtgctaacga gcgagtgaag tatacgtaac gaggcattag
241621 agcaagggct ttcagctcaa tccgttgctt gttgcccctt
//

```
LOCUS       1033_126_1        155272 bp    DNA     circular    16-JUN-2025
DEFINITION  1033_126_1 chloroplast
ACCESSION   1033_126_1
SOURCE      chloroplast 1033_126_1
  ORGANISM  1033_126_1
            1033_126_1;
REFERENCE   1  (bases 1 to 155272)
  AUTHORS   Tillich,M., Lehwark,P., Pellizzer,T., Ulbricht-Jones, E.S.,
            Fischer, A., Bock, R. and Greiner,S.
  TITLE     GeSeq - versatile and accurate annotation of organelle genomes
  JOURNAL   Nucleic Acids Res. 45 (W1), W6-W11 (2017)
   PUBMED   28486635
COMMENT     GeSeq Version 2.03
            Job          GeSeqJob-20250601-162423
            Subjob       1033_126_174_copy_301_174+%28circular%29
            Creator      anonymous
            Program      BLAT v36x7
                         blatxcutoff=25                blatncutoff=85
                         blatxmaxintron=750000         blatnmaxintron=0
                         annotate_ir=true              shortmatches=false
                         mpimpchlororefset=true
            Program      HMMER 3.3.1 disabled
            Program      ARAGORN v1.2.38 disabled
            Program      ARWEN v1.2.3 disabled
            Program      MFannot v1.34 disabled
            Program      Chloë v0.1.0
                         annotate_trna=false           annotate_trna=false
                         annotate_cds=true             disable_dropping=false
            Program      tRNAscan-SE v1.3.1 disabled
            Program      tRNAscan-SE v2.0.6 disabled
            Program      RNA Editing disabled
            Submitted    16 Jun 2025 08:24:25 GMT
            Started      16 Jun 2025 08:24:34 GMT
            Finished     16 Jun 2025 08:24:59 GMT
FEATURES             Location/Qualifiers
     source          1..155272
                     /organism="1033_126_1"
                     /organelle="plastid:chloroplast"
                     /mol_type="genomic DNA"
     gene            join(complement(98779..99572),14548..14661)
                     /gene="rps12"
                     /info="annotated by Chloe v0.1.0"
                     /annotator="Chloe"
                     /trans_splicing
```

CDS   join(14548..14661,complement(99341..99572),
complement(98779..98804))
/gene="rps12"
/annotator="Chloe"
/trans_splicing
/product="ribosomal protein S12"

/translation="MPTIKQLIRNTRQPIRNVTKSPALRGCPQRRGTCTRVYTITPKK

PNSALRKVARVRLTSGFEITAYIPGIGHNSQEHSVVLVRGGRVKDLPGVRYHIVRGTL
DAVGVKDRQQGRSKYGVKKPK"
misc_feature   1..85872
/note="large single copy (LSC)"
/info="annotated by OGDRAW v1.4"
/annotator="OGDRAW"
gene   join(155215..155272,1..221)
/gene="rps19"
/info="annotated by Chloe v0.1.0; blatX_hit
rps19_Stuberosum_GeSeq-SRS_v6, position 1 - 279, psl
score 94.7, coverage 100.00%, match 94.62%; merged"
/annotator="Chloe, blatX; merged"
CDS   join(155215..155272,1..221)
/gene="rps19"
/annotator="Chloe, blatX; merged"
/product="ribosomal protein S19"

/translation="VTRSLKKNPFVANRLLRKIDKLNTKAEKEIIITWSRASTIIPTM

IGHTIAIHNGKEHLPIYITDRMVGHKLGEFAPTLNFRGHAKSDNRSRR"
gene   288..779
/gene="rpl22"
/info="annotated by Chloe v0.1.0"
/annotator="Chloe"
CDS   288..779
/gene="rpl22"
/annotator="Chloe"
/product="ribosomal protein L22"

/translation="MLKKLKKIKTEVYALGQHISMSANKARRVVDQIRGRSYEETLMI

LELMPYRACYPILKLVYSAAANASYTMDSNESNLVISKAQVCEGTATKKLKPRARGRS

FTIKRPTCHIAIVVKDISLDEYIEIDFLDSLRWSKKLKSKNKYTALAYHDMYSNGGVW
DKK"

gene 764..1420
/gene="rps3"
/info="annotated by Chloe v0.1.0; blatX_hit
rps3_Abelladonna_GeSeq-SRS_v6, position 1 - 657, psl
score 92.1, coverage 100.00%, match 92.09%; merged"
/annotator="Chloe, blatX; merged"
CDS 764..1420
/gene="rps3"
/annotator="Chloe, blatX; merged"
/product="ribosomal protein S3"

/translation="MGQKINPLGFRLGTTQSHHSLWFAQPKNYSEGLQEDQKIRDFIK

NYVQKNMKMSSGVEGIARIEIQKRIDLIQVIVYMGFPKLLIENRPRGIEELQMNLQKE

FNCVNRKLNITITRIAKPYGSPTILAEFIAGQLKNRVSFRKAMKKAIELTEQADTKGI

QVQIAGRIDGKEIARVEWIREGRVPLQTIRAKIDYCSYTVRTIYGVLGIKIWIFIDGE
"
gene 1576..2949
/gene="rpl16"
/info="annotated by Chloe v0.1.0"
/annotator="Chloe"
CDS join(1576..1584,2551..2949)
/gene="rpl16"
/annotator="Chloe"
/product="ribosomal protein L16"

/translation="MLSPKRTRFRKQHRGRMKGISYRGNHISFGKYALQALEPAWITS

RQIEAGRRAMTRNARRGGKIWVRVFPDKPVTVRPAETRMGSGKGSPEYWVAVVKPGRI
LYEMGGVTENIARRAISIASSKMPIRTQFIISG"
exon 1576..1584
/gene="rpl16"
/annotator="Chloe"
/number=1
intron 1585..2550
/gene="rpl16"
/annotator="Chloe"
/number=1
exon 2551..2949
/gene="rpl16"
/annotator="Chloe"
/number=2

gene 3078..3446
/gene="rpl14"
/info="annotated by Chloe v0.1.0; blatX_hit
rpl14_Stuberosum_GeSeq-SRS_v6, position 1 - 369, psl
score 92.7, coverage 100.00%, match 92.68%; merged"
/annotator="Chloe, blatX; merged"
CDS 3078..3446
/gene="rpl14"
/annotator="Chloe, blatX; merged"
/product="ribosomal protein L14"

/translation="MIQPQTLLNVADNSGARELMCIRIIGASNRRYAHIGDVIVAVIK

EAVPNMPLARSEVVRAVIVRTCKELKRDNGMIIRYDDNAAVVIDQEGNPKGTRVFGAI
ARELRQLNFTKIVSLAPEVL"
gene 3648..4052
/gene="rps8"
/info="annotated by Chloe v0.1.0; blatX_hit
rps8_Abelladonna_GeSeq-SRS_v6, position 1 - 405, psl
score 91.2, coverage 100.00%, match 91.11%; merged"
/annotator="Chloe, blatX; merged"
CDS 3648..4052
/gene="rps8"
/annotator="Chloe, blatX; merged"
/product="ribosomal protein S8"

/translation="MGRDTIADIITSIRNADMDRKRVVRIAATNIAENIVKILLQEGF

IENVRKHQETKKSFLVLTLRHRRNRKRPYRNILNLKRISRPGLRIYSNYQRIPRILGG
MGIVILSTSRGIMTDREARLERIGGEVLCYIW"
gene 4165..4398
/gene="infA"
/info="annotated by Chloe v0.1.0; blatX_hit
infA_Eguineensis_GeSeq-SRS_v6, position 1 - 234, psl
score 91.5, coverage 100.00%, match 91.45%; merged"
/annotator="Chloe, blatX; merged"
CDS 4165..4398
/gene="infA"
/annotator="Chloe, blatX; merged"
/product="translational initiation factor 1"

/translation="MKEQKWIHEGLITESLPNGMFRIRLDNQDLILGYVSGKIRRSFI
RILPGDRVKIEVSRYDSTRGRIIYRLRNKDSKD"
gene 4515..4628

/gene="rpl36"
/info="annotated by Chloe v0.1.0; blatX_hit
rpl36_Abelladonna_GeSeq-SRS_v6, position 1 - 114, psl
score 96.5, coverage 100.00%, match 96.49%; merged"
/annotator="Chloe, blatX; merged"
CDS 4515..4628
/gene="rpl36"
/annotator="Chloe, blatX; merged"
/product="ribosomal protein L36"
/translation="MKIRASVRKICEKCRLIRRRGRIIVICSNPRHKQRQG"
gene 4736..5152
/gene="rps11"
/info="annotated by Chloe v0.1.0; blatX_hit
rps11_Nundulata_GeSeq-SRS_v6, position 1 - 417, psl score
92.1, coverage 100.00%, match 92.09%; merged"
/annotator="Chloe, blatX; merged"
CDS 4736..5152
/gene="rps11"
/annotator="Chloe, blatX; merged"
/product="ribosomal protein S11"

/translation="MAKAIPRIGSRRNVRIGSRKNTRRIPKGVIHVQASFNNTIVTVT

DVRGRVVSWSSAGTCGFKGTRRGTPFAAQTAAGNAIRTVVDQGMQRAEVMIKGPGLGR
DAALRAIRRSGILLTFVRDVTPMPHNGCRPPKKRRV"
gene 5227..6300
/gene="rpoA"
/info="annotated by Chloe v0.1.0"
/annotator="Chloe"
CDS 5227..6300
/gene="rpoA"
/annotator="Chloe"
/product="RNA polymerase subunit alpha"

/translation="MVREKVTVSTRTLQWKCVESKADNKRLYYGRFILSPLMKGQADT

IGIAMRRALLGEIEGTCITRAKSEKIPHEYSTLIGIQESVHDILMNLKAIVLRSNLYG

TCTAAICVRGPGSVTAQDIILPPYVEIVDNTQHIASLTEPIELYIGLQIERNRGYLIK

TPNNNFQDGSYPIDAVFMPVRNANHSIHSYGNGNDKQEILFLEIWTNGSLTPKEALHE

ASRNLIDLFIPFLHTEEENVHLADNEHMVPLPPFTFHDKLAKLRKNKKKIALKSIFID

QSELPPRIYNCLLRSNIYTLLDLLNNSPEGLMKIEHFRIEDVKQILGILEKHFAVYLP
KKPKMGFESLAQFRYSESIPIPE"
gene complement(6483..7737)
/gene="petD"
/info="annotated by Chloe v0.1.0"
/annotator="Chloe"
CDS complement(join(6483..6957,7730..7737))
/gene="petD"
/annotator="Chloe"
/product="cytochrome b6/f subunit IV"

/translation="MGVTKKPDLNDPVLRAKLAKGMGHNYYGEPAWPNDLLYIFPVVI

LGTIACNVGLAVLEPSMIGEPADPFATPLEILPEWYFFPVFQILRTVPNKLLGVLLMV

SVPAGLLTVPFLENVNKFQNPFRRPVATTVFLVGTAVALWLGIGATLPIDKSLTLGLF
"
exon complement(7730..7737)
/gene="petD"
/annotator="Chloe"
/number=1
intron complement(6958..7729)
/gene="petD"
/annotator="Chloe"
/number=1
exon complement(6483..6957)
/gene="petD"
/annotator="Chloe"
/number=2
gene complement(7961..9361)
/gene="petB"
/info="annotated by Chloe v0.1.0"
/annotator="Chloe"
CDS complement(join(7961..8602,9356..9361))
/gene="petB"
/annotator="Chloe"
/product="cytochrome b6"

/translation="MSKVYDWFEERLEIQAIADDITSKYVPPHVNIFYCLGGITLTCF

LVQVATGFAMTFYYRPTVTDAFASVQYIMTEANFGWLIRSVHRWSASMMVLMMILHVF

RVYLTGGFKKPRELTWVTGVVLAVLTASFGVTGYSLPWDQIGYWAVKIVTGVPEAIPV

IGSPLVELLRGSASVGQSTLTRFYSLHTFVLPLLTAVFMLMHFPMIRKQGISGPL"
exon complement(9356..9361)
/gene="petB"
/annotator="Chloe"
/number=1
intron complement(8603..9355)
/gene="petB"
/annotator="Chloe"
/number=1
exon complement(7961..8602)
/gene="petB"
/annotator="Chloe"
/number=2
gene complement(9486..9707)
/gene="psbH"
/info="annotated by Chloe v0.1.0; blatX_hit
psbH_Nundulata_GeSeq-SRS_v6, position 1 - 222, psl score
91.0, coverage 100.00%, match 90.99%; merged"
/annotator="Chloe, blatX; merged"
CDS complement(9486..9707)
/gene="psbH"
/annotator="Chloe, blatX; merged"
/product="photosystem II subunit H"

/translation="MATQTVESGSRSRPKPTAVGKLLKPLNSEYGKVAPGWGTTPLMG
VAMALFAIFLSIILEIYNSSVLLDEISMN"
gene 9810..9941
/gene="pbf1"
/info="annotated by Chloe v0.1.0; blatX_hit
pbf1_Stuberosum_GeSeq-SRS_v6, position 1 - 132, psl score
97.8, coverage 100.00%, match 97.73%; merged"
/annotator="Chloe, blatX; merged"
CDS 9810..9941
/gene="pbf1"
/annotator="Chloe, blatX; merged"
/product="photosystem biogenesis factor 1"

/translation="METATLVAIFISGLLVSFTGYALYTAFGQPSQQLRDPFEEHGD"
gene complement(10028..10129)
/gene="psbT"
/info="annotated by Chloe v0.1.0; blatX_hit
psbT_Ljaponicus_GeSeq-SRS_v6, position 1 - 102, psl score
93.2, coverage 100.00%, match 93.14%; merged"

/annotator="Chloe, blatX; merged"
CDS complement(10028..10129)
/gene="psbT"
/annotator="Chloe, blatX; merged"
/product="photosystem II subunit T"
/translation="MEALVYTFLLVSTLGIIFFAIFFREPPKVPTKK"
gene complement(10339..11865)
/gene="psbB"
/info="annotated by Chloe v0.1.0; blatX_hit
psbB_Vvinifera_GeSeq-SRS_v6, position 1 - 1527, psl score
94.5, coverage 100.00%, match 94.43%; merged"
/annotator="Chloe, blatX; merged"
CDS complement(10339..11865)
/gene="psbB"
/annotator="Chloe, blatX; merged"
/product="photosystem II 47 kDa protein"

/translation="MGLPWYRVHTVVLNDPGRLISVHIMHTALVAGWAGSMALYELAV

FDPSDPVLDPMWRQGMFVIPFMTRLGITTSWGGWSITGGAMPNPGIWSYEGVAGAHIV

FSGLCFLAAIWHWVYWDLEIFSDERTGKPSLDLPKIFGIHLFLAGVACFGFGAFHVTG

LYGPGIWVSDPYGLTGKVQSVNPAWGVEGFDPFVPGGIASHHIAAGTLGILAGLFHLS

VRPPQRLYKGLRMGNIETVLSSSIAAVFFAAFVVAGTMWYGSATTPIELFGPTRYQWD

QGYFQQEIYRRVSAGLAENQSLSEAWSKIPEKLAFYDYIGNNPAKGGLFRAGSMDNGD

GIAVGWLGHPIFRDKEGRELFVRRMPTFFETFPVVLVDGDGIVRADVPFRRAESKYSV

EQVGVTVEFYGGELNGVSYSDPATVKKYARRAQLGEIFELDRATLKSDGVFRSSPRGW

FTFGHASFALLFFFGHIWHGARTLFRDVFAGIDPDLDAQVEFGAFQKLGDPTTRRQVV

"
gene 12353..14384
/gene="clpP1"
/info="annotated by Chloe v0.1.0"
/annotator="Chloe"
CDS join(12353..12423,13249..13540,14154..14384)
/gene="clpP1"
/annotator="Chloe"
/product="ATP-dependent Clp protease proteolytic subunit
1"

/translation="MPIGVPKVPFRSPGEEDASWVDVYNRLYRERLLFLGQEVDSEIS

NQLIGLMVYLSIENDTKDLYLFINSPGGWVIPGIAIYDTMQFVRPDVQTICMGLAASM

GSFILAGGEITKRLAFPHARVMIHQPASSFYEAQTGEFILEAEELLKLRETLTRVYVQ
RTDKPLWVVSEDMERDVFMSATEAQAYGIVDLVAVVE"
exon 12353..12423
/gene="clpP1"
/annotator="Chloe, blatX; merged"
/number=1
intron 12424..13248
/gene="clpP1"
/annotator="Chloe, blatX; merged"
/number=1
exon 13249..13540
/gene="clpP1"
/annotator="Chloe, blatX; merged"
/number=2
intron 13541..14153
/gene="clpP1"
/annotator="Chloe, blatX; merged"
/number=2
exon 14154..14384
/gene="clpP1"
/annotator="Chloe"
/number=3
gene 14548..142366
/gene="rps12"
/info="blatX_hit rps12_Stuberosum_GeSeq-SRS_v6, position
1 - 372, psl score 88.2, coverage 100.00%, match 98.12%"
/annotator="blatX"
CDS join(14548..14661,141573..141805,142342..142366)
/gene="rps12"
/annotator="blatX"
/product="ribosomal protein S12"

/translation="MPTIKQLIRNTRQPIRNVTKSPALRGCPQRRGTCTRVYTITPKK

PNSALRKVARVRLTSGFEITAYIPGIGHNSQEHSVVLVRGGRVKDLPGVRYHIVRGTL
DAVGVKDRQQGRSRYGVKKPK"
gene join(14548..14661,141573..142366)
/gene="rps12"
/info="annotated by Chloe v0.1.0"

/annotator="Chloe"
/trans_splicing
CDS join(14548..14661,141573..141804,142341..142366)
/gene="rps12"
/annotator="Chloe"
/trans_splicing
/product="ribosomal protein S12"

/translation="MPTIKQLIRNTRQPIRNVTKSPALRGCPQRRGTCTRVYTITPKK

PNSALRKVARVRLTSGFEITAYIPGIGHNSQEHSVVLVRGGRVKDLPGVRYHIVRGTL
DAVGVKDRQQGRSKYGVKKPK"
intron 14662..141572
/gene="rps12"
/annotator="blatX"
/number=1
exon 14548..14661
/gene="rps12"
/annotator="Chloe, blatX; merged"
/number=1
exon 141573..141805
/gene="rps12"
/annotator="blatX"
/number=2
intron 141806..142341
/gene="rps12"
/annotator="blatX"
/number=2
exon 142342..142366
/gene="rps12"
/annotator="blatX"
/number=3
gene 15434..15820
/gene="rpl20"
/info="annotated by Chloe v0.1.0; blatX_hit
rpl20_Abelladonna_GeSeq-SRS_v6, position 1 - 387, psl
score 93.1, coverage 100.00%, match 93.02%; merged"
/annotator="Chloe, blatX; merged"
CDS 15434..15820
/gene="rpl20"
/annotator="Chloe, blatX; merged"
/product="ribosomal protein L20"

/translation="MTRIRRGYIARRRRTKIRLFASSFRGAHSRLTRTITQQKIKALV

SSHRDRGRQKRNFRRLWITRINAVIREMGVSYSYSRLIHDLYKKQVLLNRKILAQIAI
SNRNCLYMISNEIIKEVDWKESTGII"
gene complement(16061..16366)
/gene="rps18"
/info="annotated by Chloe v0.1.0; blatX_hit
rps18_Vvinifera_GeSeq-SRS_v6, position 1 - 306, psl score
98.1, coverage 100.00%, match 98.04%; merged"
/annotator="Chloe, blatX; merged"
CDS complement(16061..16366)
/gene="rps18"
/annotator="Chloe, blatX; merged"
/product="ribosomal protein S18"

/translation="MDKSKRPFLKSKRSFRRRLPPIQSGDRIDYRNMSLISRFISEQG

KILSRRVNRLTLKQQRLITIAIKQARILSLLPFLNNEKQFERTESTARTAGLRARNK"
gene complement(16572..16772)
/gene="rpl33"
/info="annotated by Chloe v0.1.0; blatX_hit
rpl33_Slycopersicum_GeSeq-SRS_v6, position 1 - 201, psl
score 92.1, coverage 100.00%, match 92.04%; merged"
/annotator="Chloe, blatX; merged"
CDS complement(16572..16772)
/gene="rpl33"
/annotator="Chloe, blatX; merged"
/product="ribosomal protein L33"

/translation="MAKGKDVRITVILECTSCVRNDENKVSTGISRYITQKNRHNTPN
RLELRKFCPYCYKHTIHGEIKK"
gene complement(17237..17365)
/gene="psaJ"
/info="annotated by Chloe v0.1.0"
/annotator="Chloe"
CDS complement(17237..17365)
/gene="psaJ"
/annotator="Chloe"
/product="photosystem I subunit J"

/translation="MRDLKTYLSVAPVLSTLWFGSLAGLLIEINRFFPDALTFPFF"
gene complement(18194..18307)
/gene="petG"
/info="annotated by Chloe v0.1.0; blatX_hit
petG_Stuberosum_GeSeq-SRS_v6, position 1 - 114, psl score

94.8, coverage 100.00%, match 94.74%; merged"
/annotator="Chloe, blatX; merged"
CDS complement(18194..18307)
/gene="petG"
/annotator="Chloe, blatX; merged"
/product="cytochrome b6/f subunit G"
/translation="MIEVFLFGIVLGLIPVTLAGLFVTAYLQYRRGDQSDL"
gene complement(18460..18555)
/gene="petL"
/info="annotated by Chloe v0.1.0; blatX_hit
petL_Hbrasiliensis_GeSeq-SRS_v6, position 1 - 96, psl
score 92.8, coverage 100.00%, match 92.71%; merged"
/annotator="Chloe, blatX; merged"
CDS complement(18460..18555)
/gene="petL"
/annotator="Chloe, blatX; merged"
/product="cytochrome b6/f subunit L"
/translation="MPTITSYFGFLLASLTITSALFIGLSKIRLI"
gene 19641..19892
/gene="psbE"
/info="annotated by Chloe v0.1.0; blatX_hit
psbE_Vvinifera_GeSeq-SRS_v6, position 1 - 252, psl score
97.3, coverage 100.00%, match 97.22%; merged"
/annotator="Chloe, blatX; merged"
CDS 19641..19892
/gene="psbE"
/annotator="Chloe, blatX; merged"
/product="cytochrome b559 subunit alpha"

/translation="MSGSTGERSFADIITSIRYWVIHSITIPSLFIAGWLFVSTGLAY
DVFGSPRPNEYFTENRQGIPLITGRFDPLEQLDEFSRSF"
gene 19902..20021
/gene="psbF"
/info="annotated by Chloe v0.1.0; blatX_hit
psbF_Vvinifera_GeSeq-SRS_v6, position 1 - 120, psl score
98.4, coverage 100.00%, match 98.33%; merged"
/annotator="Chloe, blatX; merged"
CDS 19902..20021
/gene="psbF"
/annotator="Chloe, blatX; merged"
/product="cytochrome b559 subunit beta"
/translation="MTIDRTYPIFTVRWLAVHGLAVPTVSFLGSISAMQFIQR"
gene 20044..20160
/gene="psbL"

/info="annotated by Chloe v0.1.0"
/annotator="Chloe"
CDS 20044..20160
/gene="psbL"
/annotator="Chloe"
/product="photosystem II subunit L"
/translation="MTQSNPNEQSVELNRTSLYWGLLLIFVLAVLFSNYFFN"
gene 20293..20415
/gene="psbJ"
/info="annotated by Chloe v0.1.0; blatX_hit
psbJ_Stuberosum_GeSeq-SRS_v6, position 1 - 123, psl score
96.8, coverage 100.00%, match 96.75%; merged"
/annotator="Chloe, blatX; merged"
CDS 20293..20415
/gene="psbJ"
/annotator="Chloe, blatX; merged"
/product="photosystem II subunit J"
/translation="MADTTGRIPLWIIGTVAGILVIGLIGIFFYGSYSGLGSSL"
gene complement(21479..22441)
/gene="petA"
/info="annotated by Chloe v0.1.0; blatX_hit
petA_Abelladonna_GeSeq-SRS_v6, position 1 - 963, psl
score 93.3, coverage 100.00%, match 93.25%; merged"
/annotator="Chloe, blatX; merged"
CDS complement(21479..22441)
/gene="petA"
/annotator="Chloe, blatX; merged"
/product="cytochrome f"

/translation="MQTRKTLSWIKKQITRSISVSLMIYIITRTSISSAYPIFAQQGY

ENPREATGRIVCANCHLANKPVDIEVPQAVLPDTVFEAVVRIPYDMQVKQVLANGKKG

ALNVGAVLILPEGFELAPADRISPEMKEKIGNLSFQSYRPNQKNILVIGPVPGQKYSE

ITFPILSPDPATKKEAHFLKYPIYVGGNRGRGQIYPDGSKSNNTVYNATAAGIVGKII

RKEKGGYEITIADTSDGRQVVDIIPPGPELLVSEGESIKLDQPLTSNPNVGGFGQGDA
EIVLQDPLRVQGLLFFLASVILAQIFLVLKKKQFEKVQLSEMNF"
gene complement(22722..23411)
/gene="cemA"
/info="annotated by Chloe v0.1.0; blatX_hit
cemA_Vvinifera_GeSeq-SRS_v6, position 1 - 690, psl score
91.1, coverage 100.00%, match 91.01%; merged"

/annotator="Chloe, blatX; merged"
CDS complement(22722..23411)
/gene="cemA"
/annotator="Chloe, blatX; merged"
/product="envelope membrane carbon uptake protein"

/translation="MAKKKALTPLLYLASIVFLPWGISLSFNKSLESWITYWWNTRQS

ENFLNDIQEKNILEKFIELEEILLLEEMIKEYSETHLQNLRIGIHKETIQLIKIHNED

RIHTILHFSTNLICFVILSGYSILGNEELVILNSWAREFLYNLSDTIKAFSILLLTDL

CIGFHSPHGWELMIGSVYKDFGFVHNDQIVSGLVSTFPVILDTIFKFWIFRYLNRVSP
SLVVIYHSMND"
gene complement(24031..24585)
/gene="pafII"
/info="annotated by Chloe v0.1.0"
/annotator="Chloe"
CDS complement(24031..24585)
/gene="pafII"
/annotator="Chloe"
/product="photosystem I assembly factor II"

/translation="MSCRSEHIWIEPITGSRKISNFCWAVILFLGSLGFLLVGTSSYL

GRNLISLFPSQQILFFPQGIVMSFYGIAGLFISSYLWCTISWNVGGGYDRFDRKEGMV

CIFRWGFPGKNRRIFLRFLIKDIQSVRIEVKEGIYARRVLYMDIRGQGAIPLTRTDEN
VTPREIEQKAAELAYFLRVPIEVF"
gene complement(25008..25118)
/gene="psaI"
/info="annotated by Chloe v0.1.0"
/annotator="Chloe"
CDS complement(25008..25118)
/gene="psaI"
/annotator="Chloe"
/product="photosystem I subunit I"
/translation="MTTFDFPSVLVPLVGLVFPAMAMASLSLHVQKNTTV"
gene complement(25393..26850)
/gene="accD"
/info="annotated by Chloe v0.1.0"
/annotator="Chloe"
CDS complement(25393..26850)
/gene="accD"

/annotator="Chloe"
/product="acetyl-CoA carboxylase subunit beta"

/translation="MKRWWLGSILFKKEFERRCGLRKSMGGLGPIENTGESEDPNRND

MNKNIHSWGGRDNSSYSNVDHLFGVKDIRNFISDETFLVMDSKGNSYSIYFDLENQIF

EVDSGHFFQSELESSFSSYWTFSYLTDDPHYDRYMDDTQDSWNNHINSCIDNYLQSQI

FIDTDIVSGSDNDSNSYISSSLCGESGDSNLSTSTNGSDFAIKNSSNDLDVTQKYRHL

WVQCENCYGLNYKKFFKSKMNLCEQCGYHLKMSSSDRIELSIDPGTWDPMDEDMVSLD

PIEFHSEEDPYKDRIDSYQRNTGLTEAVQTGIGQLNGIPVALGVMDFQFMGGSMGSVV

GEKITRLIEYATQKCLPLIIACASGGARMQEGSLSLMQMAKISSALYDYQSTKKLFYV

PILTSPTTGGVTASFGMLGDIIIAEPNAYIAFAGKRVIEQTLNKTVPEGSQAAENLFQ
KGLFDLIVPRNPLKSVLSELFQLHAFFPLKQNEIK"

gene complement(27532..28959)
/gene="rbcL"
/info="annotated by Chloe v0.1.0; blatX_hit
rbcL_Vvinifera_GeSeq-SRS_v6, position 1 - 1428, psl score
93.5, coverage 100.00%, match 93.49%; merged"
/annotator="Chloe, blatX; merged"

CDS complement(27532..28959)
/gene="rbcL"
/annotator="Chloe, blatX; merged"
/product="ribulose-1,5-bisphosphate carboxylase/oxygenase
large subunit"

/translation="MSPQTETKAGVGFKAGVKDYKLTYYTPDYETKDTDILAAFRVTP

QPGVPPEEAGAAVAAESSTGTWTTVWTDGLTSLDRYKGRCYDIEPVAGEENQFIAYVA

YPLDLFEEGSVTNMFTSIVGNVFGFKALRALRLEDLRIPISYVKTFQGPPHGIQVERD

KLNKYGRPLLGCTIKPKLGLSAKNYGRAVYECLRGGLDFTKDDENVNSQPFMRWRDRF

LFCAEALYKAQTETGEIKGHYLNATAGTCEEMMKRAIFARELGVPIVMHDYLTGGFTA

NTTLAHYCRDNGLLLHIHRAMHAVIDRQKNHGMHFRVLAKALRMSGGDHIHSGTVVGK

LEGERDITLGFVDLLRDDFIEKDRSRGIYFTQDWVSLPGVLPVASGGIHVWHMPALTE

IFGDDSVLQFGGGTLGHPWGNAPGAVANRVALEACVQARNEGRDLASEGNEIIREAAK
WSPELAAACEVWKEIKFEFAAMDTL"
gene 29731..31227
/gene="atpB"
/info="annotated by Chloe v0.1.0; blatX_hit
atpB_Vvinifera_GeSeq-SRS_v6, position 1 - 1497, psl score
94.5, coverage 100.00%, match 94.46%; merged"
/annotator="Chloe, blatX; merged"
CDS 29731..31227
/gene="atpB"
/annotator="Chloe, blatX; merged"
/product="CF1 subunit beta"

/translation="MRINPTTSDSGVSTLEKKNLGSITQIIGPVLDVAFPPGKMPNIY

NALVVKGRDTAGQQINVTCEVQQLLGNNRVRAVAMSATEGLTRGMEVIDTGAPLSVPV

GGTTLGRIFNVLGEPVDNLGPVDTRTTSPIHRSAPAFIQLDTKLSIFETGIKVVDLLA

PYRRGGKIGLFGGAGVGKTVLIMELINNIAKAHGGVSVFGGVGERTREGNDLYMEMKE

SGVINEENIAESKVALVYGQMNEPPGARMRVGLTALTMAEYFRDVNEQDVLLFIDNIF

RFVQAGSEVSALLGRMPSAVGYQPTLSTEMGTLQERITSTKEGSITSIQAVYVPADDL

TDPAPATTFAHLDATTVLSRGLAAKGIYPAVDPLDSTSTMLQPRIVGEEHYETAQRVK

QTLQRYKELQDIIAILGLDELSEEDRLTVARARKIERFLSQPFFVAEVFTGSPGKYVG

LAETIRGFQLILSGELDGLPEQAFYLVGNIDEATAKAMNLEMESNSKK"
gene 31224..31625
/gene="atpE"
/info="annotated by Chloe v0.1.0; blatX_hit
atpE_Vvinifera_GeSeq-SRS_v6, position 1 - 402, psl score
90.3, coverage 100.00%, match 90.30%; merged"
/annotator="Chloe, blatX; merged"
CDS 31224..31625
/gene="atpE"
/annotator="Chloe, blatX; merged"
/product="CF1 subunit epsilon"

/translation="MTLNLCVLTPNRTVWNSNVNEIILSTNSGQIGVLPDHASIATAV

DIGILRIRLDGQWLTMALMGGFARIGNNEITVLVNDAEKSSDIDSQEAQQTLEIAEEN
FKKAEGKRQKIEANLALRRARTRVETINAIS"
gene 33819..34181
/gene="ndhC"
/info="annotated by Chloe v0.1.0; blatX_hit
ndhC_Stuberosum_GeSeq-SRS_v6, position 1 - 363, psl score
94.8, coverage 100.00%, match 94.77%; merged"
/annotator="Chloe, blatX; merged"
CDS 33819..34181
/gene="ndhC"
/annotator="Chloe, blatX; merged"
/product="NADH dehydrogenase subunit C"

/translation="MFLLYEYDIFWSFLIISSLIPILAFFISGVLAPINKGPEKLSSY

ESGIEPMGNAWLQFRIRYYMFALVFVVFDVETVFLYPWAMSFDVLGVSVVIEAFIFVL
ILIVGLVYAWRKGALEWS"
gene 34231..34908
/gene="ndhK"
/info="annotated by Chloe v0.1.0; blatX_hit
ndhK_Vvinifera_GeSeq-SRS_v6, position 1 - 678, psl score
93.4, coverage 100.00%, match 93.36%; merged"
/annotator="Chloe, blatX; merged"
CDS 34231..34908
/gene="ndhK"
/annotator="Chloe, blatX; merged"
/product="NADH dehydrogenase subunit K"

/translation="MNSIEFPLLDRTTQNSVISTTSNDLSNWSRLSSLWPLLYGTSCC

FIEFASLIGSRFDFDRYGLVPRSSPRQADLILTAGTVTMKMAPSLVRLYEQMPEPKYV

IAMGACTITGGMFSTDSYSTVRGVDKLIPVDVYLPGCPPKPEAVIDAITKLRKKISRE

IYEDRIKSQRENRCFTTNHKFQVGRSIHTGNYDREFLYQPPSTSEIPPETFFKYKSSV
SSRELVN"
gene 35014..35490
/gene="ndhJ"
/info="annotated by Chloe v0.1.0; blatX_hit
ndhJ_Ntabacum_GeSeq-SRS_v6, position 1 - 477, psl score
94.6, coverage 100.00%, match 94.55%; merged"
/annotator="Chloe, blatX; merged"
CDS 35014..35490

/gene="ndhJ"
/annotator="Chloe, blatX; merged"
/product="NADH dehydrogenase subunit J"

/translation="MQGPLSAWLGKHGLIHRSLGFDYQGIETLQIKPEDWHSIAVILY

IYGYNYLRSQCAYDVAPGGLLASVYHLTRIEYGVDQPEEVCIKVFAPRGNPRIPSVFW

VWKSVDFQERESYDMLGISYENHPRLKRILMPESWIGWPLRKDYIAPNFYEIQDAH"
gene 38108..38713
/gene="rps4"
/info="annotated by Chloe v0.1.0; blatX_hit
rps4_Nundulata_GeSeq-SRS_v6, position 1 - 606, psl score
93.9, coverage 100.00%, match 93.89%; merged"
/annotator="Chloe, blatX; merged"
CDS 38108..38713
/gene="rps4"
/annotator="Chloe, blatX; merged"
/product="ribosomal protein S4"

/translation="MSRYRGPRFKKIRRLGALPGLTNKRPRAGSDLRNQSRSGKKSQY

RIRLEEKQKLRFHYGLTERQLLKYVRIAGKAKGSTGQVLLQLLEMRLDNILFRLGMAP

TIPGARQLVNHRHILVNGRIVDIPSYRCKPRDIITARDEQNSRALIQNSFDSSSHDEL
PKHLTLYPFQYKGLVNQIIDSKWVGLKINELLVVEYYSRQT"
gene 39912..41975
/gene="pafI"
/info="annotated by Chloe v0.1.0; blatX_hit
pafI_Vvinifera_GeSeq-SRS_v6, position 1 - 507, psl score
91.6, coverage 100.00%, match 96.84%; merged"
/annotator="Chloe, blatX; merged"
CDS join(39912..40037,40758..40983,41821..41975)
/gene="pafI"
/annotator="blatX"
/product="photosystem I assembly factor I"

/translation="MPRSRINGNFIDKTFSIVANILLRIIPTTSGEKEAFTYYRDGMS

AQSEGNYAEALQNYYEAMRLEIDPYDRSYILYNIGLIHTSNGEHTKALEYYFRALERN

PFLPQAFNNMAVICHYRGEQAIRQGDSEIAEAWFDQAAEYWKQAIALTPGNYIEAHNW
LKITRRFE"
exon 39912..40037

/gene="pafI"
/annotator="blatX"
/number=1
CDS join(39912..40035,40756..40985,41823..41975)
/gene="pafI"
/annotator="Chloe"
/product="photosystem I assembly factor I"

/translation="MPRSRINGNFIDKTFSIVANILLRIIPTTSGEKEAFTYYRDGMS

AQSEGNYAEALQNYYEAMRLEIDPYDRSYILYNIGLIHTSNGEHTKALEYYFRALERN

PFLPQAFNNMAVICHYRGEQAIRQGDSEIAEAWFDQAAEYWKQAIALTPGNYIEAHNW
LKITRRFE"
intron 40038..40757
/gene="pafI"
/annotator="blatX"
/number=1
exon 39912..40035
/gene="pafI"
/annotator="Chloe"
/number=1
exon 40758..40983
/gene="pafI"
/annotator="blatX"
/number=2
intron 40036..40755
/gene="pafI"
/annotator="Chloe"
/number=1
exon 40756..40985
/gene="pafI"
/annotator="Chloe"
/number=2
intron 40984..41820
/gene="pafI"
/annotator="blatX"
/number=2
intron 40986..41822
/gene="pafI"
/annotator="Chloe"
/number=2
exon 41821..41975
/gene="pafI"

/annotator="blatX"
/number=3
exon 41823..41975
/gene="pafI"
/annotator="Chloe"
/number=3
gene 42747..44999
/gene="psaA"
/info="annotated by Chloe v0.1.0; blatX_hit
psaA_Ntabacum_GeSeq-SRS_v6, position 1 - 2253, psl score
95.6, coverage 100.00%, match 95.56%; merged"
/annotator="Chloe, blatX; merged"
CDS 42747..44999
/gene="psaA"
/annotator="Chloe, blatX; merged"
/product="photosystem I P700 apoprotein A1"

/translation="MIIRSPEPEVKILVDRDHIKTSFEEWARPGHFSRTLAKGPDTTT

WIWNLHADAHDFDSHTSDLEEISRKVFSAHFGQLSVIFLWLSGMYFHGARFSNYEAWL

SDPTHIGPSAQVVWPIVGQEILNGDVGGGFRGIQITSGFFQLWRASGITSELQLYCTA

IGALIFAALMLFAGWFHYHKAAPKLAWFQDVESMLNHHLAGLLGLGSLSWAGHQVHVS

LPINQFLNAGVDPKEIPLPHEFILNRDLLAQLYPSFAEGATPFFTLNWSKYADFLTFR

GGLDPVTGGLWLTDIAHHHLAIAILFLIAGHMYKTNWGIGHGLKDILEAHKGPFTGQG

HKGLYEILTTSWHAQLSLNLAMLGSLTIVVAHHMYSMPPYPYLATDYGTQLSLFTHHM

WIGGFLIVGAAAHAAIFMVRDYDPTTRYNDLLDRVLRHRDAIISHLNWACIFLGFHSF

GLYIHNDTMSALGRPQDMFSDTAIQLQPVFAQWIQNTHALAPGATAPGATTSTSLTWG

GSDLVAVGGKVALLPIPLGTADFLVHHIHAFTIHVTVLILLKGVLFARSSRLIPDKAN

LGFRFPCDGPGRGGTCQVSAWDHVFLGLFWMYNAISVVIFHFSWKMQSDVWGSISDQG

VVTHITGGNFAQSSITINGWLRDFLWAQASQVIQSYGSSLSAYGLFFLGAHFVWAFSL

MFLFSGRGYWQELIESIVWAHNKLKVAPATQPRALSIVQGRAVGVTHYLLGGIATTWA
FFLARIIAVG"
gene 45025..47229

/gene="psaB"
/info="annotated by Chloe v0.1.0; blatX_hit
psaB_Vvinifera_GeSeq-SRS_v6, position 1 - 2205, psl score
95.2, coverage 100.00%, match 95.15%; merged"
/annotator="Chloe, blatX; merged"
CDS 45025..47229
/gene="psaB"
/annotator="Chloe, blatX; merged"
/product="photosystem I P700 apoprotein A2"

/translation="MALRFPRFSQGLAQDPTTRRIWFGIATAHDFESHDDITEERLYQ

NIFASHFGQLAIIFLWTSGNLFHVAWQGNFESWVQDPLHVRPIAHAIWDPHFGQPAVE

AFTRGGALGPVNIAYSGVYQWWYTIGLRTNEDLYTGALFLLFISAISLIAGWLHLQPK

WKPSVSWFKNAESRLNHHLSGLFGVSSLAWTGHLVHVAIPASRGEYVRWNNFLDVLPH

PQGLGPLFTGQWNLYAQNPDSSSHLFGTSQGAGTAILTLLGGFHPQTQSLWLTDMAHH

HLAIAFIFLVAGHMYRTNFGIGHSMKDLLDAHIPPGGRLGRGHKGLYDTINNSLHFQL

GLALASLGVITSLVAQHMYSLPAYAFIAQDFTTQAALYTHHQYIAGFIMTGAFAHGAI

FFIRDYSPEQNEDNVLARMLDHKEAIISHLSWASLFLGFHTLGLYVHNDVMLAFGTPE

KQILIEPIFAQWIQSAHGKTSYGFDVLLSSTNGPAFNAGRSIWLPGWLNAVNETSNSL

FLTIGPGDFLVHHAIALGLHTTTLILVKGALDARGSKLMPDKKDFGYSFPCDGPGRGG

TCDISAWDAFYLAVFWMLNTIGWVTFYWHWKHITLWQGNVSQFNESSTYLMGWLRDYL

WLNSSQLINGYNPFGMNSLSVWAWMFLFGHLVWATGFMFLISWRGYWQELIETLAWAH

ERTPLANLIRWRDKPVALSIVQARLVGLAHFSVGYIFTYAAFLIASTSGKFG"
gene 47354..47656
/gene="rps14"
/info="annotated by Chloe v0.1.0; blatX_hit
rps14_Stuberosum_GeSeq-SRS_v6, position 1 - 303, psl
score 94.4, coverage 100.00%, match 94.39%; merged"
/annotator="Chloe, blatX; merged"
CDS 47354..47656
/gene="rps14"
/annotator="Chloe, blatX; merged"

/product="ribosomal protein S14"

/translation="MARKGLIQREKKRQKLEQKYHLIRRSSKKEISKVLSLSDKWEIY

GKLQSPPRNSAPTRLHRRCFATGRPRANYRDFGLSGHILREMVHACLLPGATRSSW"

gene complement(48445..48633)
/gene="psbZ"
/info="annotated by Chloe v0.1.0; blatX_hit
psbZ_Stuberosum_GeSeq-SRS_v6, position 1 - 189, psl score
95.8, coverage 100.00%, match 95.77%; merged"
/annotator="Chloe, blatX; merged"

CDS complement(48445..48633)
/gene="psbZ"
/annotator="Chloe, blatX; merged"
/product="photosystem II subunit Z"

/translation="MTLAFQLAVFALIATSSILLIGVPVVFASPDGWSSNKNVLFSGT
SLWIGLVFLVGILNSLIS"

gene complement(49343..50728)
/gene="psbC"
/info="annotated by Chloe v0.1.0; blatX_hit
psbC_Ntabacum_GeSeq-SRS_v6, position 1 - 1386, psl score
95.5, coverage 100.00%, match 95.45%; merged"
/annotator="Chloe, blatX; merged"

CDS complement(49343..50728)
/gene="psbC"
/annotator="Chloe, blatX; merged"
/product="photosystem II 43 kDa protein"

/translation="VETLFNGTLALSGRDQETTGFAWWAGNARLINLSGKLLGAHVAH

AGLIVFWAGGMNLFEVAHFVPEKPMYEQGLILLPHLATLGWGVGPGGEVIDTFPYFVS

GVLHLISSAVLGFGGIYHALLGPETLEESFPFFGYVWKDRNKMTTILGIHLILLGVGA

FLLVFKALYFGGVYDTWAPGGGDVRKITNLTLSPSIIFGYLLKSPFGGEGWIVSVDDL

EDIIGGHVWLGSICILGGIWHILTKPFAWARRALVWSGEAYLSYSLAALSVFGFIACC

FVWFNNTAYPSEFYGPTGPEASQAQAFTFLVRDQRLGANVGSAQGPTGLGKYLMRSPT

GEVIFGGETMRFWDLRAPWLEPLRGPNGLDLSRLKKDIQPWQERRSAEYMTHAPLGSL

NSVGGVATEINAVNYVSPRSWLATSHFVLGFFLFVGHLWHAGRARAAAAGFEKGIDRD

FEPVLSMTPLN"
gene complement(50712..51773)
/gene="psbD"
/info="annotated by Chloe v0.1.0; blatX_hit
psbD_Ntabacum_GeSeq-SRS_v6, position 1 - 1062, psl score
95.7, coverage 100.00%, match 95.67%; merged"
/annotator="Chloe, blatX; merged"
CDS complement(50712..51773)
/gene="psbD"
/annotator="Chloe, blatX; merged"
/product="photosystem II protein D2"

/translation="MTIALGKFTKDEKDLFDFMDDWLRRDRFVFVGWSGLLLFPCAYF

ALGGWFTGTTFVTSWYTHGLASSYLEGCNFLTAAVSTPANSLAHSLLLLWGPEAQGDF

TRWCQLGGLWTFVALHGAFGLIGFMLRQFELARSVQLRPYNAIAFSGPIAVFVSVFLI

YPLGQSGWFFAPSFGVAAIFRFILFFQGFHNWTLNPFHMMGVAGVLGAALLCAIHGAT

VENTLFEDGDGANTFRAFNPTQAEETYSMVTANRFWSQIFGVAFSNKRWLHFFMLFVP

VTGLWMSALGVVGLALNLRAYDFVSQEIRAAEDPEFETFYTKNILLNEGIRAWMAAQD
QPHENLIFPEEVLPRGNAL"
gene 54566..54682
/gene="psbM"
/info="annotated by Chloe v0.1.0"
/annotator="Chloe"
CDS 54566..54682
/gene="psbM"
/annotator="Chloe"
/product="photosystem II subunit M"
/translation="MEVNILAFIATALFILVPTAFLLIIYVKTESQNQNKKD"
gene complement(55753..55842)
/gene="petN"
/info="annotated by Chloe v0.1.0; blatX_hit
petN_Hbrasiliensis_GeSeq-SRS_v6, position 1 - 90, psl
score 97.8, coverage 100.00%, match 97.78%; merged"
/annotator="Chloe, blatX; merged"
CDS complement(55753..55842)
/gene="petN"
/annotator="Chloe, blatX; merged"
/product="cytochrome b6/f subunit N"
/translation="MDIVSLAWAALMVVFTFSLSLVVWGRSGL"

gene 57934..61146
/gene="rpoB"
/info="annotated by Chloe v0.1.0; blatX_hit
rpoB_Ntomentosiformis_GeSeq-SRS_v6, position 1 - 3213,
psl score 93.1, coverage 100.00%, match 93.06%; merged"
/annotator="Chloe, blatX; merged"
CDS 57934..61146
/gene="rpoB"
/annotator="Chloe, blatX; merged"
/product="RNA polymerase subunit beta"

/translation="MLRDGNEGMSTIPGFTQIQFEGFCRFIDQGLREELYKFPKIEDT

DQEIEFQLFVETYQLVEPLIKERDAAYESLTYSSELYVSAGLIWKTGRDMQEQTIFLG

NIPLMNSLGTSIVNGIYRIVINQILQSPGIYYRSELDHNGISVYTGTIISDWGGRSEL

EIDRKARIWARVSRKQKISILVLSSAMGSNLREILENVCYPEIFLSFLTDKEKNKIGS

KENAILEFYQQFACVGGDPVFSESLCKELQKKFFQQRCELGKIGRRNMNRRLNLDISQ

TNTFLLPRDILAAADRLIGMKFGMGTLDDMNHLKNKRIRSVADLIQDQFGLALVRLEN

VIRGTICGALRHKFIPAPQNLVTSTPLTTTYESFFGLHPLSQVLDRTNPLTQIVHGRK

LSYLGPGGVTGRTASFRIRDIHPSHYGRICPIDTSEGINVGLIGSLAIHAKIGRWGSL

ESPFYEISDRSKGARILYLSPGKDEYYMVAAGNPLALNQGIQEEQVVPARYRQEFLTI

AWEQVHLRSIFSFQYFSIGASLIPFIEHNDANRALMSSNMQRQAVPLSRSEKCIVGTG

LERQAALDSGVLAIAEHEGKVIYTDTDKILLSGNGDTLSIPLVMYQRSNKNTCMHQKP

QVQRGKYIKKGQILAYGAATVGGELALGKNVLVAYMPWEGYNFEDAVLISERLVYEDI

YTSFHIRKYEIQTHVTSQGPERITKAIPHLEAHLLRNLDKNGIVMLGSWVETGEILVG

KLTPQMVKESSYAPEDRLLRAILGIQVSTSKETCLKLPIGGRGRVIDVRWIHKRGGSS

YNPETIRVYISQKREIKVGDKVAGRHGNKGIISKILPRQDMPYLQDGRPVDMVFNPLG

VPSRMNVGQIFECSLGLAGSLLNRHYRIAPFDERYEQEASRKLVFSELYQASKQTANP

WVFEPEYPGKSRIFDGRTGDPFEQPVIIGKPYILKLIHQVDDKIHGRSSGHYALVTQQ

PLRGRAKQGGQRVGEMEVWALEGFGVAHILQEMLTYKSDHIRARQEVLGTTIIGGAIP
NPQDAPESFRLLVRELRSLALELNHFFVSEKNFQINRKEA"
gene 61173..63982
/gene="rpoC1"
/info="annotated by Chloe v0.1.0"
/annotator="Chloe"
CDS join(61173..61604,62366..63982)
/gene="rpoC1"
/annotator="Chloe"
/product="RNA polymerase subunit beta'"

/translation="MIDRYKHQQLRIGSVSPQQISTWANKILPNGEIVGEVTKPYTFH

YKTNKPEKDGLFCERIFGPIKSGICACGNYRVIGDEKDDPKFCEQCGVEFVDSRIRRY

QMGYIKLACPVTHVWYLKRLPSYIANLLDKPLKELEGLVYCDFSFARPIAKKPTFLRL

RGLFEYEIQSWKYSIPLFFTTQGFDTFRNREISTGAGAIREQLADLDLRIIIDSSLVE

WKELGEDGPTGNEWEDRKVGRRKDFLVRRMELAKHFIRTNIDPEWMVLCLLPVLPPEL

RPIIQIDGGKLMSSDINELYRRVIYRNNTLTDLLTTSRSTPGELVMCQEKLVQEAVDT

LLDNGIRGQPMRDGHNKVYKSFSDVIEGKEGRFRETLLGKRVDYSGRSVIVVGPSLSL

HQCGLPREIAIELFQTFVIRGLIRQHLASNIGVAKSKIREKKPIVWEILREVMQGHPV

LLNRAPTLHRLGIQAFQPVLVEGRAICLHPLVRKGFNADFDGDQMAVHVPLSFEAQAE

ARLLMFSHINLLSPAIGDPISVPTQDMLIGLYVLTSGNRRGICVNRYNPCNHRNYQNE

IIYENNSKYTKEKEPFFCNSYDAIGAYRQKRINLNSPLWLRWRLDRRVIAAREAPLEV

HYESLGTYYDIYGQYLIVRSIKKEIISIYIRTTVGHISIYREIEEAIQGFCQACSYGP
"
exon 61173..61604
/gene="rpoC1"
/annotator="Chloe, blatX; merged"
/number=1
intron 61605..62365
/gene="rpoC1"
/annotator="Chloe, blatX; merged"
/number=1

exon 62366..63982
/gene="rpoC1"
/annotator="Chloe"
/number=2
gene 64195..68352
/gene="rpoC2"
/info="annotated by Chloe v0.1.0; blatX_hit
rpoC2_Nundulata_GeSeq-SRS_v6, position 1 - 4167, psl
score 90.9, coverage 99.78%, match 89.85%; merged"
/annotator="Chloe, blatX; merged"
CDS 64195..68352
/gene="rpoC2"
/annotator="Chloe, blatX; merged"
/product="RNA polymerase subunit beta''"

/translation="MPERANLVFHNKVIDGTAMKRLISRLIDHFGMAYTSHILDQVKT

LGFQQATATSISLGIDDLLTIPSKRWLVQDAEQQSFILEKHHHYGNVHAVEKLRQSIE

IWYATSEFLRQEMNPNFRMTDPFNPVHIMSFSGARGNASQVHQLVGMRGLMSDPQGQM

IDLPIQSNLREGLSLTEYIISCYGARKGVVDTAVRTSDAGYLTRRLVEVVQHIVVRRK

DCGTARGISVSPQNGMMPERIFIQTLIGRVLADDIYMGPRCIAIRNQDIGSGLVNQFI

TFRAQPIYIRTPFTCRSTSWICRLCYGRSPTHGDLVELGEAVGIIAGQSIGEPGTQLT

LRTFHTGGVFTGGTAEHVRASSNGKIKFNEDLVHPTRTRHGHPAFLCSIDLYVTIESE

DILHNVHIPPKSFLLVQNDQYVESEQVIAEIRAGTSTLNFKEKVRKHIYSDSKGEMHW

NTDVYHAPEFTYGNVHLLPKTSHLWILLGEPRRSDLVSLSVHKDQDQTNARSFSAKKR

YISNLSVTNDQVRQKFFSSDFSGQKEEKRPDYSELNRKGRCNLRDPDTLHANYDLLAK

RRRKRFIIPLQSIQERETELMPFSGISIEIPINGIFRRNSILAYFDDPQYRRKSSGIT

KYGTTEVYSIVKKEDLIEYRGVKEFRPKYQLKVDRFFFIPEEVHILPGSSSIMVRNNS

IIGVDTQITLTIRSRVGGLVRVERKKKKIELKIFSGDIHFPGETDKISRHSGVLIPPG

TGKTNSKESKRKNWIYVQRITPTKKKYFVLVRPVVTYEITDGINLVTLFPSDLLQERD

NMQLRVVNYILYGNGKPIREIYDTSIQLVRTCLVLNCTQDKKSSYIEEACTSFVEIRI

KGLIRDFIKIDLSKSPISYTPKRNDPSGSGWISENGSYRTNRNPFFSSFFYYSTERIK

ESLNQNQGTIHTLLNRNKECQSLIILSSSNCFRMGPFNDVNYHNVIKESIKKDPLIPI

RNLLGPLGTALQIQIANFYSFSHLITYNQIFVTNYLQLENLKQTFQVLQYYLMDENEK

IYNPDSCSNIILNPFNLNWYFLHYNDCEEMSTIMSLGQFICENVCIVKNGPHLKSGQV

IIVQVDSVVIRSAKPYLATPGATVHGDYGEILYEGDTLVTFIYEKSRSGDITQGLPKV

EQVLEVRSIDSISMNLEKRVEGWNECITRTLGLPWGFLIGAELTIVQSRISLVNKIQK

VYRSQGVQIHNRHIEIIVRQITSKVLVSEDGMSNVFLPGEFIGLLRAERMGRALEEAI

CYRAVLLGITKASLNTQSFISEASFQETARVLAKAALRGRIDWLKGLKENVVLGGMIP

VGTGFGGLVHPSGQHNNISLESKKKNLFEGEMRDILFHHRKFFDSCLSKNFHDTSEQS
FLGFNDS"
gene 68581..69291
/gene="rps2"
/info="annotated by Chloe v0.1.0; blatX_hit
rps2_Vvinifera_GeSeq-SRS_v6, position 1 - 711, psl score
92.5, coverage 100.00%, match 92.41%; merged"
/annotator="Chloe, blatX; merged"
CDS 68581..69291
/gene="rps2"
/annotator="Chloe, blatX; merged"
/product="ribosomal protein S2"

/translation="MTNRYWNINLEEMMKAGVHFGHGTRKWNPKMAPYISAKRKGIHI

TNLTRTARFLSEACDLVFDAASRGKQFLIVGTKNKAADLVAWAAIRARCHYVNKKWLG

GMLTNWSTTETRLHKFRDLRTEQKMGGLNRLPKRDAAMLKRQLSHLQTYLGGIKYMTG

LPDIVIIVDQHEEYTALQECITLGIPTICLIDTNCDPDLADISIPANDDAISSIRLIL
NKLVFAMCEGRSSYIRNP"
gene 69568..70311
/gene="atpI"
/info="annotated by Chloe v0.1.0; blatX_hit
atpI_Stuberosum_GeSeq-SRS_v6, position 1 - 744, psl score
95.6, coverage 100.00%, match 95.56%; merged"
/annotator="Chloe, blatX; merged"

CDS 69568..70311
/gene="atpI"
/annotator="Chloe, blatX; merged"
/product="CF0 subunit IV"

/translation="MNVLSCSINTLNGLYEISGVEVGQHFYWKIGGFQVHGQVLITSW

VVIAILLGSATIAVRSPQTIPTGGQNFFEYVLEFIRDVSKTQIGEEYGPWVPFIGTMF

LFIFVSNWSGALLPWKIIQLPHGELAAPTNDINTTVALALLTSVAYFYAGLSKKGLGY

FGKYIQPTPILLPINILEDFTKPLSLSFRLFGNILADELVVVVLVSLVPLVVPIPVMF
LGLFTSGIQALIFATLAAAYIGESMEGHH"

gene 71484..71729
/gene="atpH"
/info="annotated by Chloe v0.1.0; blatX_hit
atpH_Vvinifera_GeSeq-SRS_v6, position 1 - 246, psl score
97.6, coverage 100.00%, match 97.56%; merged"
/annotator="Chloe, blatX; merged"

CDS 71484..71729
/gene="atpH"
/annotator="Chloe, blatX; merged"
/product="CF0 subunit III"

/translation="MNPLISAASVIAAGLAVGLASIGPGVGQGTAAGQAVEGIARQPE
AEGKIRGTLLLSLAFMEALTIYGLVVALALLFANPFV"

gene 72129..73401
/gene="atpF"
/info="annotated by Chloe v0.1.0; blatX_hit
atpF_Ptrichocarpa_GeSeq-SRS_v6, position 1 - 555, psl
score 87.4, coverage 100.00%, match 91.17%; merged"
/annotator="Chloe, blatX; merged"

CDS join(72129..72272,72991..73401)
/gene="atpF"
/annotator="blatX"
/product="CF0 subunit I"

/translation="MKNVTDSFVSLGHWPSAGSFGFNTDILATNLINLSVVLGVLIFF

GKGVLSDLLDNRKQRILNTIRNSEELRGGAIEQLEKARARLRKVELDADQFRVNGYSE

IEREKLNFLNSISKTLEQLENYKNETIHFEQQRAINQVRQRVFQQALQGALGTLSSCL
NNELHLRTISANIGVLGAMKEITN"

exon 72129..72272

/gene="atpF"
/annotator="blatX"
/number=1
CDS join(72129..72273,72992..73401)
/gene="atpF"
/annotator="Chloe"
/product="CF0 subunit I"

/translation="MKNVTDSFVSLGHWPSAGSFGFNTDILATNLINLSVVLGVLIFF

GKGVLSDLLDNRKQRILNTIRNSEELRGGAIEQLEKARARLRKVELDADQFRVNGYSE

IEREKLNFLNSISKTLEQLENYKNETIHFEQQRAINQVRQRVFQQALQGALGTLSSCL
NNELHLRTISANIGVLGAMKEITN"
intron 72273..72990
/gene="atpF"
/annotator="blatX"
/number=1
exon 72129..72273
/gene="atpF"
/annotator="Chloe"
/number=1
intron 72274..72991
/gene="atpF"
/annotator="Chloe"
/number=1
exon 72991..73401
/gene="atpF"
/annotator="blatX"
/number=2
exon 72992..73401
/gene="atpF"
/annotator="Chloe"
/number=2
gene 73455..74978
/gene="atpA"
/info="annotated by Chloe v0.1.0; blatX_hit
atpA_Ntabacum_GeSeq-SRS_v6, position 1 - 1524, psl score
93.6, coverage 100.00%, match 93.50%; merged"
/annotator="Chloe, blatX; merged"
CDS 73455..74978
/gene="atpA"
/annotator="Chloe, blatX; merged"
/product="CF1 subunit alpha"

/translation="MVTIRADEISNIIRERIEQYNREVKIVNTGTVLQVGDGIARIHG

LDEVMAGELVEFEEGTVGIALNLESTNVGVVLMGDGLLIQEGSSVKATGRIAQIPVSE

AYLGRVVNALAKPIDGRGEISASEYRLIESPAPGIISRRSVYEPLQTGLIAIDSMIPI

GRGQRELIIGDRQTGKTAVATDTILNQQGQNVICVYVAIGQKASSVAQVVTTFQERGA

MEYTIVVAETADSPATLQYLAPYTGAALAEYFMYRERHTSIIYDDPSKQAQAYRQMSL

LLRRPPGREAYPGDVFYLHSRLLERAAKLGSRLGEGSMTALPIVETQSGDVSAYIPTN

VISITDGQIFLSADLFNAGIRPAINVGISVSRVGSAAQIKAMKQVAGKLKLELAQFAE

LEAFAQFASDLDKATQNQLARGQRLRELLKQSQSAPLAVEEQVMTIYTGTNGYLDSLE

IGQVRKFLVELRTYLKTNKPQFQEIISSTKIFTEEAEALLKEAIQEQMERFILQEQP"

gene complement(77114..77224)
/gene="psbI"
/info="annotated by Chloe v0.1.0; blatX_hit
psbI_Slycopersicum_GeSeq-SRS_v6, position 1 - 111, psl
score 94.6, coverage 100.00%, match 94.59%; merged"
/annotator="Chloe, blatX; merged"

CDS complement(77114..77224)
/gene="psbI"
/annotator="Chloe, blatX; merged"
/product="photosystem II subunit I"
/translation="MLTLKLFVYTVVIFFVSLFIFGFLSNDPGRNPGREE"

gene complement(77633..77818)
/gene="psbK"
/info="annotated by Chloe v0.1.0; blatX_hit
psbK_Vvinifera_GeSeq-SRS_v6, position 1 - 186, psl score
96.3, coverage 100.00%, match 96.24%; merged"
/annotator="Chloe, blatX; merged"

CDS complement(77633..77818)
/gene="psbK"
/annotator="Chloe, blatX; merged"
/product="photosystem II subunit K"

/translation="MLNIFSLICICLNSAFYSSSLFFAKLPEAYAFLNPIVDLMPVIP
LFFFLLAFVWQAAVSFR"

gene 79579..80659
/gene="rps16"

/info="annotated by Chloe v0.1.0"
/annotator="Chloe"
CDS join(79579..79618,80463..80659)
/gene="rps16"
/annotator="Chloe"
/product="ribosomal protein S16"

/translation="MVKLRLKRCGRKQRAVYRIVAIDVRSRREGRDLRNVGFYDPIKN
QSYLNVPAILDFLEKGAQPTGTVRDILRKAEVFK"
exon 79579..79618
/gene="rps16"
/annotator="Chloe"
/number=1
intron 79619..80462
/gene="rps16"
/annotator="Chloe"
/number=1
exon 80463..80659
/gene="rps16"
/annotator="Chloe"
/number=2
gene 82307..83824
/gene="matK"
/info="annotated by Chloe v0.1.0; blatX_hit
matK_Ntabacum_GeSeq-SRS_v6, position 1 - 1530, psl score
85.0, coverage 99.22%, match 83.46%; merged"
/annotator="Chloe, blatX; merged"
CDS 82307..83824
/gene="matK"
/annotator="Chloe, blatX; merged"
/product="maturase K"

/translation="MEEFQRYLKLNRSQQHYFLYPLIFQEYIYALAHDHGLNRNRSVL

LENVGYDNKFSLQIVKRLIARMYQYQQKIFTLSANSFNQNIFFGHNKNLYSQMISDGF

AVIVEIPFSLRLISSLERNGIVKSQKLRSIHSIFPFLEDKFSHLIYVLEILIPYPAHL

EILVQALRYWIKDASSLHLLRFFLHEYRSWNTPNKASSFFAKRNQRLFFVLYNSHLCE

YESIFVFLRNQSSHLRSTSSGTLLERIFFYGKTEHFVEVFAKAFQANLWLLRNPFMHY

VRYQGKSILASKGTPLLMKKWTYYFVNLWKFHFDLWYQPGRICLNQLSNHSLDLLGYL

SSARLNPSMVRGQMLENAFLIDNAITKFDTIVPIISLIGSLAKAKFCTVLGHPISKAV

WSDFSDSDIVGRFGRICRKISHYYSGSSQKKSLYRIKYILRLSCARTLARKHKSTVRA

FLNRSGSGFLEEFFTAEEQVLYLTFPRASSASQRFYRRRIWYLDILCINDLANNE"
gene 84365..85426
/gene="psbA"
/info="annotated by Chloe v0.1.0; blatX_hit
psbA_Ntabacum_GeSeq-SRS_v6, position 1 - 1062, psl score
96.5, coverage 100.00%, match 96.42%; merged"
/annotator="Chloe, blatX; merged"
CDS 84365..85426
/gene="psbA"
/annotator="Chloe, blatX; merged"
/product="photosystem II protein D1"

/translation="MTAILERRESESLWGRFCNWITSTENRLYIGWFGVLMIPTLLTA

TSVFIIAFIAAPPVDIDGIREPVSGSLLYGNNIISGAIIPTSAAIGLHFYPIWEAASV

DEWLYNGGPYELIVLHFLLGVACYMGREWELSFRLGMRPWIAVAYSAPVAAATAVFLI

YPIGQGSFSDGMPLGISGTFNFMIVFQAEHNILMHPFHMLGVAGVFGGSLFSAMHGSL

VTSSLIRETTENESANEGYRFGQEEETYNIVAAHGYFGRLIFQYASFNNSRSLHFFLA

AWPVVGIWFTALGISTMAFNLNGFNFNQSVVDSQGRVINTWADIINRANLGMEVMHER
NAHNFPLDLAAVEAPSTNG"
repeat_region 85873..112035
/note="inverted repeat A (IRA)"
/info="annotated by OGDRAW v1.4"
/annotator="OGDRAW"
/rpt_type=inverted
gene complement(85995..87474)
/gene="rpl2"
/info="annotated by Chloe v0.1.0; blatX_hit
rpl2_Stuberosum_GeSeq-SRS_v6, position 1 - 825, psl score
95.9, coverage 100.00%, match 98.30%; merged"
/annotator="Chloe, blatX; merged"
CDS complement(join(85995..86428,87084..87474))
/gene="rpl2"
/annotator="Chloe, blatX; merged"
/product="ribosomal protein L2"

/translation="MAIHLYKTSTPSTRNRTVDSQVKSNPRTNLIYGQHRCGKGRNAR

GIITAGHRGGGHKRLYRKIDFRRNEKDIYGRIVTIEYDPNRNAYICLIHYGDGEKRYI

LHPRGAIIGDTIVSGTEVPIKMGNALPLTDMPLGTAIHNIEITRGRGGQLARAAGAVA

KLIAKEGKSATLKLPSGEVRLISKNCSATVGQVGNVGVNQKSLGRAGSKRWLGKRPVV

RGVVMNPVDHPHGGGEGRAPIGRKKPTTPWGYPALGRRSRKRNKYSDNLILRRRSK"
exon complement(87084..87474)
/gene="rpl2"
/annotator="Chloe, blatX; merged"
/number=1
intron complement(86429..87083)
/gene="rpl2"
/annotator="Chloe, blatX; merged"
/number=1
exon complement(85995..86428)
/gene="rpl2"
/annotator="Chloe, blatX; merged"
/number=2
gene complement(87493..87774)
/gene="rpl23"
/info="annotated by Chloe v0.1.0; blatX_hit
rpl23_Vvinifera_GeSeq-SRS_v6, position 1 - 282, psl score
99.3, coverage 100.00%, match 99.29%; merged"
/annotator="Chloe, blatX; merged"
CDS complement(87493..87774)
/gene="rpl23"
/annotator="Chloe, blatX; merged"
/product="ribosomal protein L23"

/translation="MDGIKYAVFTDKSIRLLGKNQYTSNVESGSTRTEIKHWVELFFG

VKVIAMNSHRLPGKGRRMGPIMGQTMHYRRMIITLQPGYSIPPLRKKRT"
gene 88118..94456
/gene="ycf2"
/info="annotated by Chloe v0.1.0; blatX_hit
ycf2_Soleracea_GeSeq-SRS_v6, position 1 - 6396, psl score
94.0, coverage 99.11%, match 89.17%; merged"
/annotator="Chloe, blatX; merged"
CDS 88118..94456
/gene="ycf2"

/annotator="Chloe, blatX; merged"
/product="Ycf2 protein"

/translation="MKGHQFKSWIFELREILREIKNSHYFLDSWTQFNSVGSFIHIFF

HQERFIKLFDPRIWSILLLRNSQGSASNRYFTIKGVILFVVAVLIYRINSRNRVERQN

FYLIGLLPIPMNSIGPRNDTLEESVGSSNINRLIVSLLYLPKGKKISESCFLNPKEST

WVLPITKKCSMPESNWGSRWWRNWIGKKRDSSQLKGSSDQSRDPLDSLSNEDSEYHTL

INQREIQQLKERSILWDPSFLQTERTEIESDRFPKSLSGYSSMSRLFTEREKQMINHL

LPEEIEEFLGNPTRSVRSFFSDRWSELHLGSNPTERSTRDQKWLKKQQDLSFVPSRRS

EKKEMVNIFKIITYLQNTVSIHPISSYPGCDMVPKDEPDMDSSNKISFLNKNTFLDLF

HLFHDRKRGGYTLHHDFESEERFQEMADLFTLSITEPDLVYHKGFAFSIDSYGLDQKQ

FLNEVFNSRDESKKKSLLALPPIFYEENESFYRRIIKKWVRISCGNDLEDPKQKKVVF

ASNNIMEAVNQYRLIRNLIQIQYSTYGYIRNVLNRFFLMNRSDRNFEYGIQRDQIGKD

TLNHKTRMKYTINQHLSNLKKSQKRWFDPLILISRTERSMNRDPDAYRYKWSNGSNNF

QEHLEHFVSEQKSRFQVVFDRLRINQYSIDWSEVIDKKGLSKPFRFFLSKLLFFLSNS

LPFFFLSFGNIPIHRSEIYIYELKGPNDQLCNQLLESIGLQIVHLQKWKPFLLDDHDT

SQKSKFLINGGTISPFLFNKIPKWMIDSFHTRKNRRKSFANTDSYFSTIFHDQDNWLN

PLKPFHRSSLISSFYKANRLRFLNNPHHFCFYCNKRFPFYVEKARINNDDFTYGQFLN

ILFIHNKIFSLCVGKKKHAFWGRDTISPIESQVSNIFIPNDFPQGGGDETYNLYKSSH

FPSRSNPFVRRAIYSIADISGTPLTEGQIVNFERTYCQPLSDLNLSDSEGKNSHQYLN

FNSNMGLIHTPCSEKYLPSEKRKKRSLFLKKYVEKGQMYRTFQRDSAFSTLSKWNLFQ

TYIPWFLTSTGYKYLNWIFLETFSDLLPIHLLPIHRLPILSSSHTFVSIFHDIMHVLD

IAWRILQQKWGLPQRNPIRKISSQCLHNLLLSEEMIHRNNESPLISTHLRSPNVREFL

YSILFLLLVAGYLVRTHLFFVSRASSELQTEFEKVKSLMIPSSMIELRKLLDRYPTSE

PNFFWLKNLFLVALEQLGDSLEEIRGSASGGNMLLGGDPAYGVKSIRSKKKYLNINLI

DLISIIPNPINRITFSRNTRHLSHTSKEIYSLIRKRKNVNGDWIDDKIESWVANSDSI

DDEEREFLVQFSTLTTEKRIDQILLSLTHSDHLSKNDSGYQMIEQPGAIYLRYLVDIH

KKSLMNYEFNTSCLAERRIFLAHYQTITYSQTSCGANSFHFPSHGKPFSLRLALSPSR

GILVIGSIGTGRSYLVKYLATNSYVPFITVFLNKFLDNKSKGFLSDDIDIDIDDIDIDDI

DIDASEDIDASDDIDASDDIDRDLHTELELLTTDMMSEKDQFSITLQFELAKAMSPCI

IWIPNIHDLDVNESNDFSLGLLVNHLSRDCERCSTRNILVIASTHIPQKVDPALIAPN

KLNTCIKIRRLLIPQQRKHLFTLSYTRGFRLEKKMFHTNGFGSITMGSNARDLVALTN

EALSISITQKKSIIDTNTIRSALHRQTWDLRSQVRSVQDHGILFYQIGRAVAQNVLLS

NCPIDPISIYIKKKSCNEGDSYLYKWYFELGTSMKKLTILLYLLSCSAGLVAQDLWSL

PGPDEKNGITSYGLVENDSDLVHGLLEVEGALVGSSRTERDCSQFDNDRVTLLLRPEP

RNPLDMMQNGSCSILDQRFLYEKNESGFEEGEGALDPQQIEEDLFNHIVWAPRIWHPW

GILFDCIERPNGLGFPYWSRSFRGKRILYDEEDELQENDSEFLQSGTMQSQTRDRSSK

EQGLFRISQFIWDPADPLFFLFKDQPFVSVFSHRELFADEEMSKGLLTSQTDPPTSIY

KRWFIKKTQEEHFELLINRQRGFRTTSSLSNGSFRSNTLSESYQYLSNLFLSNGTLLD
QMTKTLLRKRWLFPDEMKIGFMEQEKDLPFLRRKGMWP"
gene complement(95739..97956)
/gene="ndhB"
/info="annotated by Chloe v0.1.0; blatX_hit
ndhB_Vvinifera_GeSeq-SRS_v6, position 1 - 1533, psl score
97.1, coverage 100.00%, match 98.43%; merged"
/annotator="Chloe, blatX; merged"
CDS complement(join(95739..96494,97180..97956))
/gene="ndhB"
/annotator="Chloe, blatX; merged"
/product="NADH dehydrogenase subunit B"

/translation="MIWHVQNENFILDSTRIFMKAFHLLLFDGSLIFPECILIFGLIL

LLMIDSTSDQKDIPWLYFISSTSLVMSITALLFRWREEPMISFSGNFQTNNFNEIFQF

LILLCSTLCIPLSVEYIECTEMAITEFLLFVLTATLGGMFLCGANDLITIFVAPECFS

LCSYLLSGYTKKDVRSNEATMKYLLMGGASSSILVHGFSWLYGLSGGEIELQEIVNGL

INTQMYNSPGISIALIFITVGIGFKLSPAPSHQWTPDVYEGSPTPVVAFLSVTSKVAA

PASATRIFDIPFYFSSNEWHLLLETLAILSMILGNLIAITQTSMKRMLAYSSIGQIGY

VIIGIIVGDSNDGYASMITYMLFYISMNLGTFACIVLFGLRTGTDNIRDYAGLYTKDP

FLALSLALCLLSLGGLPPLAGFFGKLYLFWCGWQAGLYFLVLIGLLTSVVSIYYYLKI

IKLLMTGRTQEITPHVRNYRRSPFRSNNSIELSMIVCVIASTIPGISMNPIIAIAQDT

LF"

exon complement(97180..97956)
/gene="ndhB"
/annotator="Chloe, blatX; merged"
/number=1

intron complement(96495..97179)
/gene="ndhB"
/annotator="Chloe, blatX; merged"
/number=1

exon complement(95739..96494)
/gene="ndhB"
/annotator="Chloe, blatX; merged"
/number=2

gene complement(98256..98723)
/gene="rps7"
/info="annotated by Chloe v0.1.0; blatX_hit
rps7_Vvinifera_GeSeq-SRS_v6, position 1 - 468, psl score
99.4, coverage 100.00%, match 99.36%; merged"
/annotator="Chloe, blatX; merged"

CDS complement(98256..98723)
/gene="rps7"
/annotator="Chloe, blatX; merged"
/product="ribosomal protein S7"

/translation="MSRRGTAEEKTAKSDPIYRNRLVNMLVNRILKHGKKSLAYQIIY

RAVKKIQQKTETNPLSVLRQAIRGVTPDIAVKARRVGGSTHQVPIEIGSTQGKALAIR

WLLAASRKRPGRNMAFKLSSELVDAAKGSGDAIRKKEETHRMAEANRAFAHFR"
exon complement(98779..98804)
/gene="rps12"
/annotator="Chloe"
/number=3
intron complement(98805..99340)
/gene="rps12"
/annotator="Chloe"
/number=1
exon complement(99341..99572)
/gene="rps12"
/annotator="Chloe"
/number=2
gene 101769..103259
/gene="rrn16"
/info="blatN_hit rrn16_Nundulata_GeSeq-SRS_v1, position 1
- 1491, psl score 100.0, coverage 100.00%, match 99.93%"
/annotator="blatN"
rRNA 101769..103259
/gene="rrn16"
/annotator="blatN"
/product="16S ribosomal RNA"
gene 105448..108257
/gene="rrn23"
/info="blatN_hit rrn23_Rcommunis_GeSeq-SRS_v1, position 1
- 2810, psl score 99.6, coverage 100.00%, match 99.50%"
/annotator="blatN"
rRNA 105448..108257
/gene="rrn23"
/annotator="blatN"
/product="23S ribosomal RNA"
gene 108356..108458
/gene="rrn4.5"
/info="blatN_hit rrn4.5_Vvinifera_GeSeq-SRS_v1, position
1 - 103, psl score 98.1, coverage 100.00%, match 98.06%"
/annotator="blatN"
rRNA 108356..108458
/gene="rrn4.5"
/annotator="blatN"
/product="4.5S ribosomal RNA"
gene 108711..108831
/gene="rrn5"
/info="blatN_hit rrn5_Vvinifera_GeSeq-SRS_v1, position 1

- 121, psl score 99.2, coverage 100.00%, match 99.17%"
/annotator="blatN"
rRNA 108711..108831
/gene="rrn5"
/annotator="blatN"
/product="5S ribosomal RNA"
gene 110164..112131
/gene="ycf1"
/info="annotated by Chloe v0.1.0"
/annotator="Chloe"
CDS 110164..112131
/gene="ycf1"
/annotator="Chloe"
/product="Ycf1 protein"

/translation="MILKSFLLGNLVSLCMKIINSVVVVGLYYGFLTTFSIGPSYLFL

LRAQVMEEGEEGTEKKVSATTGFITGQLMMFISIYYAPLHLALGRPHTITVLALPYLL

FHFFWNNHKNFFDYGSTTRNSMRNLSIQCVFLNNLIFQLFNHFILPSSMLARLVNIYM

FRCNNKMLFVTSSFVGWLIGHILFMKWVGLVLVWIRQNNSIRSNKYIRSNKYLVSELR

NSMARIFSILLFITCVYYLGRIPSPIFTNKLNPQTEEGWESEEETASETKGTKQEQEG

STEEDPSPSLFSEEKEDPHKIDETEEIRVNGKEKTKDEFHFTETRYNNSPVYKGLFWF

EKPLLTFLFDYKRWNRPLRYIKNKRFERTARKEMSQYFFYTCRSDGKERISFTYPPSL

STFGEMIQRRMSLPTLEKLSSDELYNHWVSTNQHKNNNLNNEFLNRIEALDTGFFSRD

ILEKSTRLCNDKTRKDYLPKMYDPILNGSYRGTIKKKFSPSIIKKISLENFIETMEIN

KIHSILLPDTDYQEFEQKIDRFDKKTFSTENRHFFTLISKFDRESGSSLNLKDLSLFS

EKEQGRIGSAKRAKFYKFLLNTILTSPNGQKRKNVVIKEISKKVPRWSYKLITELEFM
IDTKSNKLIKKLIQRINTRKDKKRCARHLQI"
gene complement(112031..114268)
/gene="ndhF"
/info="annotated by Chloe v0.1.0"
/annotator="Chloe"
CDS complement(112031..114268)
/gene="ndhF"
/annotator="Chloe"

/product="NADH dehydrogenase subunit F"

/translation="MEQTYQYAWIIPFLPLPVPLLIGAGLLLFPSATKSLRRMWAFQS

VFLLSIVMIFSINLAIQQINGSSIYQYVWSWIINNDFSLEFGYLIDPLTSIMSILITT

IGILVLVYSDNYMSYDQGYLRFFAYMSFFSTSMLGLVTSSNLIQIYIFWELVGMSSYL

LIGFWFTRPVAANACQKAFVTNRVGDFGLLLGILGFYWITGSFEFRDLFKIVNTLISN

NEVNSLFVTFCAVLLFAGAVAKSAQFPLHVWLPDAMEGPTPISALIHAATMVAAGIFL

VARLIPLFIVIPHIMNFISLVGIITVLFGATFALAQKDIKRGLAYSTMSQLGYMMLAL

GMGSYRSALFHLITHAYSKALLFLGSGSVIHSMETLVGYSPNKSQNMVLMGGLTKHVP

ITKTSFLVGTLSLCGIPPLACFWSKDEILNDTWLYSPIFAIIAWSTAGLTAFYMFRIY

LLTFEGHLHVHFQNYSGKKNTPFYSISLWGKEDSKRITKNVRLLTLLTMNNYEISSFF

SKKAYRIDQSAKNITQPFITIPCFGNKKIFSYPYESENTMLFPILILVLFTLFIGFLG

IPFNQEGVYLDILSKWLTPSINPLHQNLNNSIDWYEFSKDALFSVSIAFFGIFIAFFL

YKPVYSSFLNLDLMNSFVKTGPKRIFLDKIINSIYDWSYNRGYIDAFYATFCTEGIRG

VAKLTSFFDRRVIDGITNGVGVLTFFVGEGIKSVGGGRISSYLFLYLSFVSIFLLIYY
FLYLS"
misc_feature 112036..129109
/note="small single copy (SSC)"
/info="annotated by OGDRAW v1.4"
/annotator="OGDRAW"
gene 114869..115033
/gene="rpl32"
/info="annotated by Chloe v0.1.0"
/annotator="Chloe"
CDS 114869..115033
/gene="rpl32"
/annotator="Chloe"
/product="ribosomal protein L32"

/translation="MAVPKKRTSISKKRIRKNIWKGKGYWAALKALSLGKSLSTGNSK
SFFVRQTNKS"
gene 116155..117132

/gene="ccsA"
/info="annotated by Chloe v0.1.0"
/annotator="Chloe"
CDS 116155..117132
/gene="ccsA"
/annotator="Chloe"
/product="cytochrome c biogenesis protein"
/translation="MIFSTLEHILTHISFSIVSIVITIHLITLLIDEIIKLYDSSERG

MIATFLCITGLLITRWIYSGHFPLSDLYESLIFLSWGLSVIHIVPYFKKKQSHLNTIT

ASSVIFTQGFATSGLLTEIHQSSILVPALQSEWLIMHVSMMILGYAALLCGSLLSVAL

LVITFRKNINLFCKRNPLLNLKLNESLSFGEIQYMNERNNILGNVSFFSAKNYYRFQL

IQQLDYWSYRVISLGFILLTIGILSGAVWANEAWGSYWNWDPKETWAFITWIVFSVYL

HTRTNRNLQVENSAIVAAMGFLIIWICYFGVNLLGIGLHSYGSFTLTSN"
gene complement(117390..118892)
/gene="ndhD"
/info="annotated by Chloe v0.1.0; blatX_hit
ndhD_Vvinifera_GeSeq-SRS_v6, position 1 - 1503, psl score
92.0, coverage 100.00%, match 91.95%; merged"
/annotator="Chloe, blatX; merged"
CDS complement(117390..118892)
/gene="ndhD"
/annotator="Chloe, blatX; merged"
/product="NADH dehydrogenase subunit D"

/translation="TNFFPWLTIIVVLPIFAGSLILFLPHRGNRVIRWYTIGICVLEL

LLTTYAFCYQFQLDDPLIQLEEDYKWINFFDFHWRLGIDGLSIGPILLTGFITTLATL

AAWPVTRDSRLFHFLMLAMYSGQIGSFSSRDLLLFFIMWELELIPVYLLLSMWGGKKR

LYSATKFILYTAGGSVFLLLGVLGVGLYGSNEPTLNFQTVVNQSYPVALEIIFYIGFF

IAFAVKSPIIPLHTWLPDTHGEAHYSTCMLLAGILLKMGAYGLIRINMELLPHAHSLF

SPWLVIVGTIQIIYAASTSPGQRNLKKRIAYSSVSHMGFILIGIGSITDTGLNGAILQ

IISHGFIGAALFFLAGTSYDRIRLVYLDEMGGIAIPMPKIFTMFSSFSMASLALPGMS

GFVAELIVFFGIITSQKYLLISKILITFVMAIGMILTPIYSLSMSRQMFYGYKLFNAP

NSYIFDSGPRELFVSIAIFLPVIGIGMYPDFVLSLSVDKVEVILSNFFYR"
gene complement(119014..119259)
/gene="psaC"
/info="annotated by Chloe v0.1.0; blatX_hit
psaC_Hbrasiliensis_GeSeq-SRS_v6, position 1 - 246, psl
score 94.8, coverage 100.00%, match 94.72%; merged"
/annotator="Chloe, blatX; merged"
CDS complement(119014..119259)
/gene="psaC"
/annotator="Chloe, blatX; merged"
/product="photosystem I subunit C"

/translation="MSHSVKIYDTCIGCTQCVRACPTDVLEMIPWDGCKAKQIASAPR
TEDCVGCKRCESACPTDFLSVRVSLWHETTRSMGLAY"
gene complement(119513..119818)
/gene="ndhE"
/info="annotated by Chloe v0.1.0; blatX_hit
ndhE_Vvinifera_GeSeq-SRS_v6, position 1 - 306, psl score
92.5, coverage 100.00%, match 92.48%; merged"
/annotator="Chloe, blatX; merged"
CDS complement(119513..119818)
/gene="ndhE"
/annotator="Chloe, blatX; merged"
/product="NADH dehydrogenase subunit E"

/translation="MMLEHVLVLSAYLFSIGLYGLITSRNMVRALMCLELILNAVNIN

FVTFSDFFDSRQLKGAIFSIFVIAIAAAEAAIGLAIVSAIYRNRKSTRINQSNLLNK"
gene complement(120036..120566)
/gene="ndhG"
/info="annotated by Chloe v0.1.0; blatX_hit
ndhG_Ntomentosiformis_GeSeq-SRS_v6, position 1 - 531, psl
score 94.2, coverage 100.00%, match 94.16%; merged"
/annotator="Chloe, blatX; merged"
CDS complement(120036..120566)
/gene="ndhG"
/annotator="Chloe, blatX; merged"
/product="NADH dehydrogenase subunit G"

/translation="MDLPGPIHDFLLVFLGSGLILGGLGVVLLPNPIYSAFSLGLVLV

CTSLFYILSNSHFVAAAQLLIYVGAINVLIIFAVMFMNGSEYYKDFHLWTVGDGVTSM

VCTSLFVSLITTIPDTSWYGIIWTTKSNQIIEQDLISNSQQIGIHLSTDFFLPFELIS

IILLVSLIGAIAVARQ"
gene complement(120925..121428)
/gene="ndhI"
/info="annotated by Chloe v0.1.0"
/annotator="Chloe"
CDS complement(120925..121428)
/gene="ndhI"
/annotator="Chloe"
/product="NADH dehydrogenase subunit I"

/translation="MFSMVTEFMNYGQQTVRAARYIGQGFMITLSHASRLPVTIQYPY

EKLITAERFRGRIHFEFDKCIACEVCVRVCPIDLPVVDWKLETDIRKKRLLNYSIDFG

ICIFCGNCVEYCPTNCLSMTEEYELSTYDRHELNYNQIALGRLPMSIINDYTTRTILN
LTEIKNA"
gene complement(121522..123694)
/gene="ndhA"
/info="annotated by Chloe v0.1.0; blatX_hit
ndhA_Vvinifera_GeSeq-SRS_v6, position 1 - 1092, psl score
90.9, coverage 100.00%, match 92.86%; merged"
/annotator="Chloe, blatX; merged"
CDS complement(join(121522..122062,123144..123694))
/gene="ndhA"
/annotator="blatX"
/product="NADH dehydrogenase subunit A"

/translation="MIIDTTEGQAINSFFRLESLKEVYGIIWMLVPIFTPVLGITIGV

LVIVWLEREISAGIQQRIGPEYAGPLGLLQALADGTKLLFKENLLPSRGDTRLFSIGP

SIAVTSILLSYLVIPFGYHLVLADLSIGVFLWIAISSIAPVGLLMSGYGSNNKYSFLG

GLRAAAQSISYEIPLTLCVLSISLLSNSSSTVDIVEAQSKYGFWGWNLWRQPIGFIVF

LISSLAECERLPFDLPEAEEELVAGYQTEYSGIKFGLFYVASYLNLLVSSLFVTVLYL

GGWNLSIPYIPGPDIFEINKAGRVFGTIIGIFITLAKTYLFLFISIITRWTLPRLRMD
QLLNLGWKFLLPISLGNLLLTTSSQLLSL"
exon complement(121522..122062)
/gene="ndhA"
/annotator="blatX"
/number=2
CDS complement(join(121522..122060,123142..123694))

/gene="ndhA"
/annotator="Chloe"
/product="NADH dehydrogenase subunit A"

/translation="MIIDTTEGQAINSFFRLESLKEVYGIIWMLVPIFTPVLGITIGV

LVIVWLEREISAGIQQRIGPEYAGPLGLLQALADGTKLLFKENLLPSRGDTRLFSIGP

SIAVTSILLSYLVIPFGYHLVLADLSIGVFLWIAISSIAPVGLLMSGYGSNNKYSFLG

GLRAAAQSISYEIPLTLCVLSISLLSNSSSTVDIVEAQSKYGFWGWNLWRQPIGFIVF

LISSLAECERLPFDLPEAEEELVAGYQTEYSGIKFGLFYVASYLNLLVSSLFVTVLYL

GGWNLSIPYIPGPDIFEINKAGRVFGTIIGIFITLAKTYLFLFISIITRWTLPRLRMD
QLLNLGWKFLLPISLGNLLLTTSSQLLSL"
intron complement(122063..123143)
/gene="ndhA"
/annotator="blatX"
/number=1
exon complement(123142..123694)
/gene="ndhA"
/annotator="Chloe"
/number=1
intron complement(122061..123141)
/gene="ndhA"
/annotator="Chloe"
/number=1
exon complement(123144..123694)
/gene="ndhA"
/annotator="blatX"
/number=1
exon complement(121522..122060)
/gene="ndhA"
/annotator="Chloe"
/number=2
gene complement(123696..124877)
/gene="ndhH"
/info="annotated by Chloe v0.1.0; blatX_hit
ndhH_Stuberosum_GeSeq-SRS_v6, position 1 - 1182, psl
score 91.6, coverage 100.00%, match 91.54%; merged"
/annotator="Chloe, blatX; merged"
CDS complement(123696..124877)
/gene="ndhH"

/annotator="Chloe, blatX; merged"
/product="NADH dehydrogenase subunit H"

/translation="MTAPATRNDLMIVNMGPQHPSMHGVLRLIVTLDGEDVIDCEPIL

GYLHRGMEKIAENRTIIQYLPYVTRWDYLATMFTEAITVNAPEQLGNIQVPKRASYVR

VIMLELSRIASHLLWLGPFMADIGAQTPFFYIFRERELIYDLFEAATGMRMMHNYFRV

GGVAADLPHGWIDKCLDFCDYFLTRITEYQRLITRNPIFLERVEGVGVIGGEEAINWG

LSGPMLRASGIQWDLRKVDHYESYDEFDWGVQWQKEGDSLARYLVRMSEMTESIKIIQ

QALEGIPGGPYENLEIRRFDKVRDPEWNDFDYRFISKKPSPTFELSKQELYVRVEAPK

GELGIFLIGDKGVFPWRWKIRPPGFINLQILPQLVKRMKLADIMTILGSIDIIMGEVD
R"
gene complement(124969..125241)
/gene="rps15"
/info="annotated by Chloe v0.1.0; blatX_hit
rps15_Rcommunis_GeSeq-SRS_v6, position 1 - 273, psl score
83.9, coverage 100.00%, match 83.88%; merged"
/annotator="Chloe, blatX; merged"
CDS complement(124969..125241)
/gene="rps15"
/annotator="Chloe, blatX; merged"
/product="ribosomal protein S15"

/translation="MIKNPFNVIILKEENKDNKGSVEFQVLCFTNRIRRLTSHLELHK
KDFLSQRGLRKILGKRQRLLAYLSKKNRVRYKDLISRLEIRETKNR"
gene complement(125657..130981)
/gene="ycf1"
/info="annotated by Chloe v0.1.0"
/annotator="Chloe"
CDS complement(125657..130981)
/gene="ycf1"
/annotator="Chloe"
/product="Ycf1 protein"

/translation="MILKSFLLGNLVSLCMKIINSVVVVGLYYGFLTTFSIGPSYLFL

LRAQVMEEGEEGTEKKVSATTGFITGQLMMFISIYYAPLHLALGRPHTITVLALPYLL

FHFFWNNHKNFFDYGSTTRNSMRNLSIQCVFLNNLIFQLFNHFILPSSMLARLVNIYM

FRCNNKMLFVTSSFVGWLIGHILFMKWVGLVLVWIRQNNSIRSNKYIRSNKYLVSELR

NSMARIFSILLFITCVYYLGRIPSPIFTNKLNPQTEEGWESEEETASETKGTKQEQEG

STEEDPSPSLFSEEKEDPHKIDETEEIRVNGKEKTKDEFHFTETRYNNSPVYKGLFWF

EKPLLTFLFDYKRWNRPLRYIKNKRFERTARKEMSQYFFYTCRSDGKERISFTYPPSL

STFGEMIQRRMSLPTLEKLSSDELYNHWVSTNQHKNNNLNNEFLNRIEALDTGFFSRD

ILEKSTRLCNDKTRKDYLPKMYDPILNGSYRGTIKKKFSPSIIKKISLENFIETMEIN

KIHSILLPDTDYQEFEQKIDRFDKKTFSTENRHFFTLISKFDRESGSSLNLKDLSLFS

EKEQGRIGSAKRAKFYKFLLNTILTSPNGQKRKNVVIKEISKKVPRWSYKLITELEFM

SGESHEGIPTDYDIRSRKCDRTMLYKPAKRRSRKASAKNWVLIRDYAEESDFRRDLIK

GSIRAQRRKTVIFKLFQANAHSPLFLDRIKKSFLFSFNILEEMNLIFKNWMGKGTEFK

DYTEEQKKRQKKEEANLREREEEENQREREEEENKIKEKALLKIAEAWDFLPYPQATR

SFILLIQSIFRKYIFLSSLIIVKNIGRILLSQPPEWTEDFEEWNREKHIICTYDGVQV

PELELPRNWFKEGIQIKIVFPFYLKPWHKSKPRSSFSSYKDLKKEQKKFCFLTAWGTQ

TNIPFGSTHKKYSFFKPIFKEVKKKIRKMKKNNFQVLIIFKEKTKKILQDSKETKRGV

TKNGLFLYIKRIKKELSKVNPTRLFRLREGYEYIETTQEKDPIINNQTIHDSFSQSKF

RDRTNFLLREKKIQNLTDRTSTIRTKIKSITKEKKKVTPIKKSSPNTTSYNVKSPKNI

WQILKIKRRSTRLIYKLDFFIKIFIKKIYTDFFLCIIHIARIPTRLVLESNIFLFKKY

SYNNETNHEKKKKKNPKESHFISTLKRALYNIKNSKENSHLFFDLSYLSQAYLFYKLS

QSHIIHLYKLRSILDYCGTSFFLTTAIKNSFVTQGMFHSELRHTKLSSLGMNQWKNWL

RGHYQYNFSQIRWSGLIPQKWRTTINQGHMTKKKDFKKWNSYEKDRLLHHKKQKDFQI

YPLPNQKDNFPKYSIHNLLSYKSINYEMKKDSYIIYESPLEVNNNQKISFNYNNYKQK

FFERLEDIPINNYLEKDDIMYMEKNSDRKYFDWIEMNEEILNLRSTNLKLPLFPEVVP

LSNVYKIKRWIIPSKLLLLNFNKKKNINEKQKWNFFRPEKKTENFELMNRNQDEKEPA

GQRGFRSYAQNQGKTKKQDKIRNKMKQEVILLREHYLLFQWIIDDGLIPNLTERMINN

IKIYCYLLGLKNPRDTTISSIQRKELNLDIMVIRKKLTPIKWKKKGILFIEPIRLSVK

NDGHFLMYQTIGISLVHKNKYQTPQRYREQRYIDKNQFEESIPRYQRRTQNRDKKHYG

FLVPEKILSSRRRRELRILFYFNSKNRNGLDINPVFCNKKNLKSGNPFLDQSKHLDRD

KNEFIKLKFFLWPNYRLEDLACMNRYWFDTNNGSHFSMLRTYPQLKIG"

repeat_region 129110..155272
/note="inverted repeat B (IRB)"
/info="annotated by OGDRAW v1.4"
/annotator="OGDRAW"
/rpt_type=inverted

gene complement(132314..132434)
/gene="rrn5"
/info="blatN_hit rrn5_Vvinifera_GeSeq-SRS_v1, position 1 - 121, psl score 99.2, coverage 100.00%, match 99.17%"
/annotator="blatN"

rRNA complement(132314..132434)
/gene="rrn5"
/annotator="blatN"
/product="5S ribosomal RNA"

gene complement(132687..132789)
/gene="rrn4.5"
/info="blatN_hit rrn4.5_Vvinifera_GeSeq-SRS_v1, position 1 - 103, psl score 98.1, coverage 100.00%, match 98.06%"
/annotator="blatN"

rRNA complement(132687..132789)
/gene="rrn4.5"
/annotator="blatN"
/product="4.5S ribosomal RNA"

gene complement(132888..135697)
/gene="rrn23"
/info="blatN_hit rrn23_Rcommunis_GeSeq-SRS_v1, position 1 - 2810, psl score 99.6, coverage 100.00%, match 99.50%"
/annotator="blatN"

rRNA complement(132888..135697)
/gene="rrn23"
/annotator="blatN"
/product="23S ribosomal RNA"

gene complement(137886..139376)
/gene="rrn16"
/info="blatN_hit rrn16_Nundulata_GeSeq-SRS_v1, position 1 - 1491, psl score 100.0, coverage 100.00%, match 99.93%"
/annotator="blatN"
rRNA complement(137886..139376)
/gene="rrn16"
/annotator="blatN"
/product="16S ribosomal RNA"
exon 141573..141804
/gene="rps12"
/annotator="Chloe"
/number=2
intron 141805..142340
/gene="rps12"
/annotator="Chloe"
/number=1
exon 142341..142366
/gene="rps12"
/annotator="Chloe"
/number=3
gene 142422..142889
/gene="rps7"
/info="annotated by Chloe v0.1.0; blatX_hit rps7_Vvinifera_GeSeq-SRS_v6, position 1 - 468, psl score 99.4, coverage 100.00%, match 99.36%; merged"
/annotator="Chloe, blatX; merged"
CDS 142422..142889
/gene="rps7"
/annotator="Chloe, blatX; merged"
/product="ribosomal protein S7"

/translation="MSRRGTAEEKTAKSDPIYRNRLVNMLVNRILKHGKKSLAYQIIY

RAVKKIQQKTETNPLSVLRQAIRGVTPDIAVKARRVGGSTHQVPIEIGSTQGKALAIR

WLLAASRKRPGRNMAFKLSSELVDAAKGSGDAIRKKEETHRMAEANRAFAHFR"
gene 143189..145406
/gene="ndhB"
/info="annotated by Chloe v0.1.0; blatX_hit ndhB_Vvinifera_GeSeq-SRS_v6, position 1 - 1533, psl score 97.1, coverage 100.00%, match 98.43%; merged"
/annotator="Chloe, blatX; merged"
CDS join(143189..143965,144651..145406)

/gene="ndhB"
/annotator="Chloe, blatX; merged"
/product="NADH dehydrogenase subunit B"

/translation="MIWHVQNENFILDSTRIFMKAFHLLLFDGSLIFPECILIFGLIL

LLMIDSTSDQKDIPWLYFISSTSLVMSITALLFRWREEPMISFSGNFQTNNFNEIFQF

LILLCSTLCIPLSVEYIECTEMAITEFLLFVLTATLGGMFLCGANDLITIFVAPECFS

LCSYLLSGYTKKDVRSNEATMKYLLMGGASSSILVHGFSWLYGLSGGEIELQEIVNGL

INTQMYNSPGISIALIFITVGIGFKLSPAPSHQWTPDVYEGSPTPVVAFLSVTSKVAA

PASATRIFDIPFYFSSNEWHLLLETLAILSMILGNLIAITQTSMKRMLAYSSIGQIGY

VIIGIIVGDSNDGYASMITYMLFYISMNLGTFACIVLFGLRTGTDNIRDYAGLYTKDP

FLALSLALCLLSLGGLPPLAGFFGKLYLFWCGWQAGLYFLVLIGLLTSVVSIYYYLKI

IKLLMTGRTQEITPHVRNYRRSPFRSNNSIELSMIVCVIASTIPGISMNPIIAIAQDT
LF"
exon 143189..143965
/gene="ndhB"
/annotator="Chloe, blatX; merged"
/number=1
intron 143966..144650
/gene="ndhB"
/annotator="Chloe, blatX; merged"
/number=1
exon 144651..145406
/gene="ndhB"
/annotator="Chloe, blatX; merged"
/number=2
gene complement(146689..153027)
/gene="ycf2"
/info="annotated by Chloe v0.1.0; blatX_hit
ycf2_Soleracea_GeSeq-SRS_v6, position 1 - 6396, psl score
94.0, coverage 99.11%, match 89.17%; merged"
/annotator="Chloe, blatX; merged"
CDS complement(146689..153027)
/gene="ycf2"
/annotator="Chloe, blatX; merged"
/product="Ycf2 protein"

/translation="MKGHQFKSWIFELREILREIKNSHYFLDSWTQFNSVGSFIHIFF

HQERFIKLFDPRIWSILLLRNSQGSASNRYFTIKGVILFVVAVLIYRINSRNRVERQN

FYLIGLLPIPMNSIGPRNDTLEESVGSSNINRLIVSLLYLPKGKKISESCFLNPKEST

WVLPITKKCSMPESNWGSRWWRNWIGKKRDSSQLKGSSDQSRDPLDSLSNEDSEYHTL

INQREIQQLKERSILWDPSFLQTERTEIESDRFPKSLSGYSSMSRLFTEREKQMINHL

LPEEIEEFLGNPTRSVRSFFSDRWSELHLGSNPTERSTRDQKWLKKQQDLSFVPSRRS

EKKEMVNIFKIITYLQNTVSIHPISSYPGCDMVPKDEPDMDSSNKISFLNKNTFLDLF

HLFHDRKRGGYTLHHDFESEERFQEMADLFTLSITEPDLVYHKGFAFSIDSYGLDQKQ

FLNEVFNSRDESKKKSLLALPPIFYEENESFYRRIIKKWVRISCGNDLEDPKQKKVVF

ASNNIMEAVNQYRLIRNLIQIQYSTYGYIRNVLNRFFLMNRSDRNFEYGIQRDQIGKD

TLNHKTRMKYTINQHLSNLKKSQKRWFDPLILISRTERSMNRDPDAYRYKWSNGSNNF

QEHLEHFVSEQKSRFQVVFDRLRINQYSIDWSEVIDKKGLSKPFRFFLSKLLFFLSNS

LPFFFLSFGNIPIHRSEIYIYELKGPNDQLCNQLLESIGLQIVHLQKWKPFLLDDHDT

SQKSKFLINGGTISPFLFNKIPKWMIDSFHTRKNRRKSFANTDSYFSTIFHDQDNWLN

PLKPFHRSSLISSFYKANRLRFLNNPHHFCFYCNKRFPFYVEKARINNDDFTYGQFLN

ILFIHNKIFSLCVGKKKHAFWGRDTISPIESQVSNIFIPNDFPQGGGDETYNLYKSSH

FPSRSNPFVRRAIYSIADISGTPLTEGQIVNFERTYCQPLSDLNLSDSEGKNSHQYLN

FNSNMGLIHTPCSEKYLPSEKRKKRSLFLKKYVEKGQMYRTFQRDSAFSTLSKWNLFQ

TYIPWFLTSTGYKYLNWIFLETFSDLLPIHLLPIHRLPILSSSHTFVSIFHDIMHVLD

IAWRILQQKWGLPQRNPIRKISSQCLHNLLLSEEMIHRNNESPLISTHLRSPNVREFL

YSILFLLLVAGYLVRTHLFFVSRASSELQTEFEKVKSLMIPSSMIELRKLLDRYPTSE

PNFFWLKNLFLVALEQLGDSLEEIRGSASGGNMLLGGDPAYGVKSIRSKKKYLNINLI

DLISIIPNPINRITFSRNTRHLSHTSKEIYSLIRKRKNVNGDWIDDKIESWVANSDSI

DDEEREFLVQFSTLTTEKRIDQILLSLTHSDHLSKNDSGYQMIEQPGAIYLRYLVDIH

KKSLMNYEFNTSCLAERRIFLAHYQTITYSQTSCGANSFHFPSHGKPFSLRLALSPSR

GILVIGSIGTGRSYLVKYLATNSYVPFITVFLNKFLDNKSKGFLSDDIDIDIDDIDIDDI

DIDASEDIDASDDIDASDDIDRDLHTELELLTTDMMSEKDQFSITLQFELAKAMSPCI

IWIPNIHDLDVNESNDFSLGLLVNHLSRDCERCSTRNILVIASTHIPQKVDPALIAPN

KLNTCIKIRRLLIPQQRKHLFTLSYTRGFRLEKKMFHTNGFGSITMGSNARDLVALTN

EALSISITQKKSIIDTNTIRSALHRQTWDLRSQVRSVQDHGILFYQIGRAVAQNVLLS

NCPIDPISIYIKKKSCNEGDSYLYKWYFELGTSMKKLTILLYLLSCSAGLVAQDLWSL

PGPDEKNGITSYGLVENDSDLVHGLLEVEGALVGSSRTERDCSQFDNDRVTLLLRPEP

RNPLDMMQNGSCSILDQRFLYEKNESGFEEGEGALDPQQIEEDLFNHIVWAPRIWHPW

GILFDCIERPNGLGFPYWSRSFRGKRILYDEEDELQENDSEFLQSGTMQSQTRDRSSK

EQGLFRISQFIWDPADPLFFLFKDQPFVSVFSHRELFADEEMSKGLLTSQTDPPTSIY

KRWFIKKTQEEHFELLINRQRGFRTTSSLSNGSFRSNTLSESYQYLSNLFLSNGTLLD
QMTKTLLRKRWLFPDEMKIGFMEQEKDLPFLRRKGMWP"

gene 153371..153652
/gene="rpl23"
/info="annotated by Chloe v0.1.0; blatX_hit
rpl23_Vvinifera_GeSeq-SRS_v6, position 1 - 282, psl score
99.3, coverage 100.00%, match 99.29%; merged"
/annotator="Chloe, blatX; merged"

CDS 153371..153652
/gene="rpl23"
/annotator="Chloe, blatX; merged"
/product="ribosomal protein L23"

/translation="MDGIKYAVFTDKSIRLLGKNQYTSNVESGSTRTEIKHWVELFFG

VKVIAMNSHRLPGKGRRMGPIMGQTMHYRRMIITLQPGYSIPPLRKKRT"

gene 153671..155150

/gene="rpl2"
/info="annotated by Chloe v0.1.0; blatX_hit
rpl2_Stuberosum_GeSeq-SRS_v6, position 1 - 825, psl score
95.9, coverage 100.00%, match 98.30%; merged"
/annotator="Chloe, blatX; merged"
CDS join(153671..154061,154717..155150)
/gene="rpl2"
/annotator="Chloe, blatX; merged"
/product="ribosomal protein L2"

/translation="MAIHLYKTSTPSTRNRTVDSQVKSNPRTNLIYGQHRCGKGRNAR

GIITAGHRGGGHKRLYRKIDFRRNEKDIYGRIVTIEYDPNRNAYICLIHYGDGEKRYI

LHPRGAIIGDTIVSGTEVPIKMGNALPLTDMPLGTAIHNIEITRGRGGQLARAAGAVA

KLIAKEGKSATLKLPSGEVRLISKNCSATVGQVGNVGVNQKSLGRAGSKRWLGKRPVV

RGVVMNPVDHPHGGGEGRAPIGRKKPTTPWGYPALGRRSRKRNKYSDNLILRRRSK"
exon 153671..154061
/gene="rpl2"
/annotator="Chloe, blatX; merged"
/number=1
intron 154062..154716
/gene="rpl2"
/annotator="Chloe, blatX; merged"
/number=1
exon 154717..155150
/gene="rpl2"
/annotator="Chloe, blatX; merged"
/number=2
ORIGIN
1 ATAAGCTTAA CACAAAAGCA GAAAAAGAAA TAATAATAAC TTGGTCCCGG GCATCGACCA
61 TTATACCCAC AATGATCGGT CATACCATTG CTATCCATAA TGGAAAGGAG CATTTACCTA
121 TTTATATAAC CGATCGTATG GTAGGCCACA AATTGGGAGA ATTTGCACCT ACTTTAAATT
181 TCCGAGGACA TGCGAAAAGC GATAATAGAT CTCGTCGTTA AGAAAAGAAG GGGAAAATAT
241 ATATATATAT GCTTATTATT CATTAGTAGG AGGCGAATTT TATATTTATG TTAAAGAAGT
301 TAAAAAAGAT AAAAACGGAA GTATACGCTT TAGGCCAACA TATATCTATG TCTGCTAACA

361 AAGCGCGAAG AGTAGTTGAT CAAATTCGTG GACGTTCCTA TGAAGAAACA CTTATGATAC

421 TAGAACTCAT GCCTTATCGA GCATGTTATC CCATTTTGAA ATTGGTTTAC TCTGCAGCAG

481 CAAACGCTAG TTACACTATG GATTCCAACG AATCGAATTT AGTTATTAGT AAAGCTCAAG

541 TCTGCGAGGG TACTGCCACG AAGAAATTAA AACCTAGAGC TCGAGGGCGT AGTTTTACGA

601 TAAAAAGACC TACTTGCCAT ATAGCTATTG TAGTGAAAGA TATATCCTTA GATGAATATA

661 TCGAGATAGA CTTTCTAGAC TCTTTGAGAT GGTCAAAAAA ACTGAAATCG AAAAATAAGT

721 ATACAGCTTT GGCGTATCAC GATATGTATA GTAATGGGGG GGTATGGGAC AAAAAATAAA

781 TCCACTCGGT TTCAGACTTG GTACAACCCA AAGTCATCAT TCCCTTTGGT TTGCACAACC

841 AAAAAATTAT TCCGAAGGTC TACAAGAAGA TCAAAAAATA AGAGATTTTA TCAAGAATTA

901 TGTACAAAAA AATATGAAGA TGTCTTCCGG CGTCGAGGGA ATTGCACGTA TAGAGATTCA

961 AAAACGAATC GATCTGATTC AGGTCATTGT CTATATGGGA TTCCCAAAGT TATTAATAGA

1021 AAATCGACCG CGAGGAATCG AAGAATTGCA GATGAATCTA CAAAAAGAAT TTAATTGTGT

1081 CAACCGAAAA CTTAATATTA CTATCACAAG AATTGCAAAA CCTTACGGAA GCCCTACTAT

1141 TCTTGCAGAA TTTATAGCTG GTCAATTAAA GAATAGAGTT TCTTTTCGAA AAGCAATGAA

1201 AAAAGCTATT GAATTAACTG AACAAGCAGA TACAAAAGGA ATTCAAGTGC AAATTGCAGG

1261 GCGTATTGAC GGAAAAGAAA TTGCGCGTGT CGAATGGATC AGAGAAGGTA GGGTTCCCCT

1321 ACAAACCATT CGAGCTAAAA TAGATTATTG TTCCTATACA GTTCGAACTA TCTATGGAGT

1381 ATTAGGCATT AAAATTTGGA TATTTATAGA CGGGGAATAA TAAACCTTTA CTTGCCTTTC

1441 CTTTCGATCC AGCGATGGAA CAAAAAAAAG AGAAATTGTT CCTTTTTATT TTTCTGTTCA

1501 ATCAAAATCA AAACGAACAA TTTTAAATTC TATAGGGTTG AATAAAAATT CGATTGACCT

1561 TTTGAAATAA TTGCTATGCT TAGTGTGCGA CTCGTTGGTT TTTTAGGATT AGCTTAAAAA

1621 AAAGACGAGC CTCTTAGTAT AAACTAATAA CTTAACTATA CAACTAATGA CCAACTCATC

1681 ACTTCGCATT ATCTGGATCC AAAGAACTAG TCAAGATATG ATATATCAAT CATATCACTG

1741 TAGCAACTGA AATCTTTTTT GCATAAACAA AAGACAAAGA AATCTTATTC TACGCCGATT

1801 CTAAGTTGTG AAGCGAAATA GAGAAGAAAG GTGTGGATAA ATGGAAGGGT GAAAGAAAGG

1861 GAGAAAAAAA CAAAAACAAT GCTATAAAAT TCCATACAAT ATGTAAGGTC TACGAGTCAT

1921 CTCATAAAAT CAAAAGCAGT GTAATAAAGC ATTAATACGG ATTCGTAAAA AAGAAAATGA

1981 ATCTGTTCCT AGAACAAAAA AATAAGAGCT TCGAGCCAAT AAAGACTAAG AAAATTGGCT

2041 CAAGAAGAAA TTTCATTATG AGCTCCATTG TAGAAATCCG ACCTAACCAT TAAGTAAGAA

2101 GCGATGGGAA CGATGGAACC TGTGAATCCA AATCTATTGA AAACAGATTC ATTGATCGGG

2161 ATGGCGAAAC AAACCATAGA CAAATTCATT CATCTATTGG GCAAAGTCAT GAGCTAACCC

2221 TACAACTGAA ATAGCGACAA AAAGAGTAAA TATTCGCCCG CGAAAACTTT ATTTTCTTGG

2281 ATTGATAATT TTTGTTCAAT CCAATAGGAT AAAATAAAAG GCAAAACAAA ATAAAGATTC

2341 GTTAGAACAT TATAAAAATA AAAAAGAATA CAATATTTCA AAATTATAAA AAGAAATATT

2401 AATATGTTTA GTTATCTAAA AAAACAAGAT ATAGAACTGA ATAAGAAACT ATTCAAATTT

2461 TTTATTATTC GTGAGGAGCT GGATGAGAAG AAACTCTCAT GTCCAGTTCT GTAGTAGAGA

2521 TGGAATTCAG AACCAACCAT CAATTATAAC CCCAAAAGAA CCAGATTCCG TAAACAACAT

2581 AGAGGAAGAA TGAAGGGAAT ATCTTATCGA GGTAATCATA TTTCTTTCGG TAAATATGCC

2641 CTTCAGGCAC TTGAACCTGC TTGGATCACG TCTAGACAAA TAGAAGCAGG TCGGCGAGCA

2701 ATGACACGAA ATGCACGCCG CGGTGGAAAA ATATGGGTAC GTGTATTTCC AGACAAACCG

2761 GTTACAGTAA GACCCGCAGA AACACGTATG GGTTCGGGGA AAGGATCCCC TGAATATTGG

2821 GTAGCTGTTG TTAAACCAGG TCGAATACTT TATGAAATGG GCGGAGTCAC AGAAAATATA

2881 GCCAGAAGAG CTATTTCAAT AGCGTCGTCC AAAATGCCTA TACGAACTCA ATTCATTATT

2941 TCGGGATAAA AACGAATCAA AGGAAATAGG TCTTGGGGAT AAAAAAACAA TCGCAGGTGC

3001 CGGTTTCTTT GGACAAACAA TATTTCTTTT TTTTTCTTCG CCCTTTGCCT TGAAAGAATA

3061 GATTCTAAAA AAATGATATG ATTCAACCTC AGACCCTTTT GAATGTAGCG GATAACAGTG

3121 GGGCTCGAGA ATTGATGTGT ATTCGAATTA TAGGAGCTAG CAATCGTCGA TATGCTCATA

3181 TAGGTGACGT TATTGTTGCT GTGATCAAAG AAGCAGTGCC AAATATGCCC CTAGCAAGAT

3241 CAGAAGTGGT CAGAGCTGTA ATTGTACGTA CTTGTAAAGA ACTCAAACGC GATAACGGTA

3301 TGATAATACG ATATGATGAC AATGCTGCGG TTGTCATTGA CCAAGAAGGA AACCCAAAGG

3361 GAACTCGGGT TTTTGGCGCG ATTGCCCGGG AATTGAGACA GTTAAATTTT ACTAAAATAG

3421 TTTCATTAGC TCCGGAGGTA TTATAAGGTA TTCTAAAATG AGACCATCAT ATGGTTAAAG

3481 TAAGGTATTT GAAAGAAAGA GATTAAGAGA TATATTCAGA TTTTTAATAA ATAGTGTGTC

3541 ACGCATATGC CTTTAAGAAT TCAAATTCAT AAAAAAAATA AGTAAAAAAA CAAGAAAGAC

3601 ATGTTGATTA TATCAAAATT TGGGAGCACC AAGAATTTTA ATTCATCATG GGTAGGGATA

3661 CTATTGCTGA CATAATAACC TCTATACGAA ATGCGGATAT GGATAGAAAA AGAGTGGTTC

3721 GAATAGCAGC TACTAATATC GCTGAAAATA TTGTTAAAAT ACTTTTACAA GAAGGTTTTA

3781 TCGAAAACGT GAGAAAACAC CAAGAAACCA AAAAATCTTT TTTGGTTTTA ACCCTACGGC

3841 ATAGAAGGAA TAGGAAAAGG CCCTATAGAA ATATTTTAAA TTTAAAACGC ATCAGTCGGC

3901 CTGGTCTACG AATCTATTCT AACTACCAAC GAATTCCTAG AATTTTAGGT GGGATGGGAA

3961 TTGTAATTCT TTCCACTTCT CGAGGTATAA TGACAGACCG AGAGGCCCGA CTAGAAAGAA

4021 TTGGCGGAGA AGTTTTGTGT TATATATGGT GATCCTTCTA ATATCCGAAT TGGATCCGAA

4081 ACTTATTATT CGTGAAAAAA AGAAAAGAGA CGGGTTGTCG AATACTCCTC CATCAGTTGA

4141 TACTTCAAGG AGGCTTTACC TGGAATGAAA GAACAAAAAT GGATTCATGA AGGTTTAATT

4201 ACTGAATCCC TTCCCAATGG TATGTTCCGG ATTCGCTTAG ATAATCAAGA TCTAATTTTA

4261 GGTTATGTTT CAGGAAAGAT CCGACGTAGT TTTATACGGA TACTGCCAGG CGATAGAGTC

4321 AAAATTGAAG TAAGTCGTTA TGATTCAACC AGAGGACGTA TAATTTATCG ACTTCGCAAT

4381 AAGGATTCGA AAGATTAGGT GTTTTTATCA ACTTCAACAT TCCTTTCATG GGAATATGAT

4441 TCCAGAAGAA AAATTTAAAG AAACCTATTT TCTTCCAAAA AGTAGATTCA GAATTAAGAT

4501 GAGGAATGCC AAATATGAAA ATAAGAGCTT CTGTTCGTAA AATTTGTGAA AAATGTCGAC

4561 TAATCCGCAG GCGGGGACGA ATTATAGTAA TTTGTTCCAA TCCAAGACAT AAACAAAGAC

4621 AGGGGTAATC AGACTTGTTA AAATACCCGA CGGAAAAGTC AAAAGAGGGA TCTTTTTTTG

4681 ACACGAAATG GATATATCCA TATATCTCTG ACTCATATTT ATGAGATGAA AAAATATGGC

4741 AAAAGCTATA CCGAGAATTG GTTCACGTAG GAATGTACGT ATTGGTTCAC GTAAGAATAC

4801 ACGTAGAATA CCAAAGGGGG TTATTCATGT TCAAGCAAGT TTCAATAATA CTATTGTGAC

4861 TGTTACCGAT GTACGGGGGC GAGTGGTTTC TTGGTCCTCT GCCGGTACTT GTGGATTCAA

4921 GGGCACAAGA AGAGGCACAC CGTTTGCTGC TCAAACCGCA GCGGGAAACG CTATTCGTAC

4981 AGTAGTGGAT CAAGGCATGC AACGAGCAGA AGTCATGATA AAAGGACCCG GTCTAGGAAG

5041 AGACGCGGCA TTACGCGCTA TTCGCAGAAG TGGTATACTA TTAACTTTCG TTCGGGATGT

5101 AACCCCTATG CCACATAATG GCTGTAGACC TCCGAAAAAA CGACGTGTGT AGAAATAAAA

5161 ATTGAAGAGA TTTCAAGAGA AACAAGAGAA AAAAATGATT CAATGATCTG ATCAAATAAT

5221 ATTACTATGG TTCGAGAGAA AGTAACAGTA TCTACTCGGA CACTACAATG GAAGTGTGTT

5281 GAATCAAAGG CAGACAATAA GCGTCTTTAT TATGGACGCT TTATTCTGTC TCCACTTATG

5341 AAAGGTCAAG CTGACACAAT AGGTATTGCG ATGCGAAGAG CTTTGCTTGG AGAAATAGAA

5401 GGAACATGTA TTACACGTGC AAAATCTGAG AAAATACCAC ACGAGTATTC TACCCTAATA

5461 GGTATTCAAG AATCGGTCCA TGACATTTTA ATGAATTTGA AAGCAATTGT ATTGAGAAGT

5521 AATCTATATG GAACTTGCAC CGCGGCTATT TGTGTCAGGG GTCCTGGATC TGTAACTGCT

5581 CAAGATATCA TCTTACCGCC TTATGTAGAA ATCGTTGACA ATACACAGCA TATAGCTAGC

5641 TTGACGGAAC CAATTGAGTT GTATATTGGA TTACAAATAG AAAGGAATCG TGGATATCTT

5701 ATAAAAACGC CAAATAACAA CTTCCAAGAC GGAAGTTATC CTATAGATGC TGTATTCATG

5761 CCTGTTCGAA ACGCCAATCA CAGTATTCAT TCTTATGGGA ATGGGAATGA TAAACAAGAG

5821 ATACTCTTTC TTGAAATATG GACAAATGGA AGTTTAACTC CTAAAGAAGC ACTTCATGAA

5881 GCCTCCCGGA ATTTGATTGA TTTATTTATT CCTTTTTTAC ATACAGAAGA AGAAAATGTA

5941 CATTTAGCGG ACAATGAACA CATGGTTCCT TTACCCCCTT TTACCTTTCA CGATAAATTG

6001 GCTAAACTAA GAAAAAACAA AAAAAAAATA GCATTGAAAT CAATTTTTAT TGACCAATCA

6061 GAACTACCTC CCAGGATCTA TAATTGCCTT CTAAGGTCCA ATATATATAC ATTATTGGAC

6121 CTTTTGAATA ACAGTCCGGA AGGCCTTATG AAAATTGAAC ATTTTCGAAT AGAAGATGTA

6181 AAACAGATAT TGGGCATTCT AGAAAAGCAT TTCGCAGTTT ATTTACCGAA AAAGCCAAAA

6241 ATGGGTTTTG AATCTTTAGC ACAATTCAGA TATTCGGAAT CTATCCCGAT TCCTGAATGA

6301 AATTGAATTC ATAGAATAAT AAATGAAATT AAATAATATA TTATATAATC AAAAACTAGA

6361 AATATTCTAA TAAAAGAATA GATGTGTATC TAGGGAGAAT TCGCTTTGTT TGAAGCGACT

6421 ATTCCCTAGA TACACACGTC GTGAGATTTT AATTTTATTT CACAATTGGC TCAATTTAAA

6481 AATTAAAAAA GACCTAAAGT GAGGGATTTA TCAATAGGTA ATGTTGCACC AATACCCAAC

6541 CAAAGGGCCA CTGCGGTACC AACCAAAAAG ACGGTTGTCG CTACTGGACG ACGAAATGGG

6601 TTTTGGAATT TATTAACATT CTCTAAAAAA GGTACTGTTA ATAATCCTGC GGGTACTGAA

6661 ACCATTAAAA GAACACCCAA TAATTTATTG GGTACTGTAC GAAGTATTTG AAATACGGGA

6721 AAGAAATACC ATTCGGGTAA TATTTCCAAA GGGGTTGCAA ATGGATCTGC AGGTTCACCA

6781 ATCATTGATG GTTCTAGAAC CGCTAAGCCT ACGTTACATG CAATAGTCCC TAGAATTACT

6841 ACTGGAAAAA TATATAAAAG ATCATTGGGC CAGGCGGGCT CTCCATAATA ATTATGCCCC

6901 ATACCTTTGG CCAATTTGGC TCTTAATACA GGATCGTTCA AGTCAGGCTT TTTTGTTATT

6961 GGGATAGGTG AATTCTTATA GATCCATCCC CCGAAGGAAC CGGACATGAT AATTTTTCAT

7021 CATCCGGCTC GAGCAAGAAT AAAAAGAACC AAATAGGAAT CCATAAAAGA TCTATAGGTT

7081 ATCTAGATCC ACACATATAT GATATATGAA TGGGTTTTTG TAAGAAACAT ATTACTGGAT

7141 TTTTTCTGGA GTTGCACAAC TATCCAGTGC AAAGAATTCA GTTCTTACAA GTAAATACTC

7201 AATACCCCAC TCAATACCCA AGTAGGCTTA CAAAGTTGAT CTTGATATGA TCAAGTGCTT

7261 TTTGGGTTGT CTCAAACCTT ACGATTGATG TTTATCCCCA AATCGAAATA TTGGAAGACT

7321 TTTTACAGAC ACAAAAGGTT TCCTTGTTAC TAATATAGCC TAGCCTCCTT CTTCTTTAGA

7381 TCCCTTGTTT CACTCCGATA GTATCATAGA TCACGCCAAT GCAGAGGAAA TGAATGCATT

7441 TCCATACTAT TCAATTTTAA GTAAGTGAAC TAGAGAAACC ATAATCAAGA TTATGGTTTC

7501 TCTAGTTCAC TTATTCTACT GGATACTGGA TCTTACGGAG CCGGTTCATG TACGACATGA

7561 TTCGGATTTG ATCATAGAAA GGATTTTCTT CAAGCGAACC AGCCTATCCT CTGTATGGAG

7621 CTTATGCAGT GGTTGGAGCA AAGACACATT TGGTTGTGAA TTCCAATGCA ATGTGGCAGA

7681 TAGATAAAGG CAGATATCTG CACAGACCAA TCAATAGTTC AAGTCACACA CTCCCATAAT

7741 CCATTTCCTC TTCGGAAAAT TCCCTTCAAC TATATGAAAA ACTCTATTCA AAAGATTGCA

7801 GCGTTCAAGG GAAATATCCA AATACATGTC TTATTATTTT CAATAATCCA TACTTGTAGC

7861 AATGAAATAC TATTGTTCTT TTCCCAAGTG ATAAAGGATG GATTACAAAT ATCTATGATA

7921 TATCTTCTCT ATATCTATAA AGAACCAGAA ATCTCTATAT CTATAAAGGA CCAGAAATGC

7981 CTTGTTTACG TATCATTGGA AAGTGCATTA ACATAAATAC GGCAGTAAGA AGCGGCAATA

8041 CAAAAGTGTG TAAACTATAA AAACGAGTCA AAGTGGATTG CCCCACACTA GCACTTCCAC

8101 GCAATAATTC TACTAAAGGC GATCCTATTA CAGGAATAGC TTCGGGTACA CCTGTTACAA

8161 TTTTCACTGC CCAATAACCA ATTTGGTCCC AAGGTAAGGA ATAACCAGTT ACACCAAAAG

8221 ATGCAGTCAA TACGGCCAGA ACCACACCTG TAACCCAAGT TAATTCGCGA GGTTTTTTAA

8281 ATCCACCGGT CAGGTACACA CGAAATACAT GCAGGATCAT CATTAGAACC ATCATACTTG

8341 CTGACCAACG ATGAACTGAT CGGATTAACC AACCAAAATT AGCTTCAGTC ATTATGTATT

8401 GAACCGAAGC AAAAGCGTCA GTAACGGTCG GACGGTAGTA AAAAGTCATA GCAAACCCTG

8461 TAGCCACTTG TACTAAAAAA CAAGTAAGCG TAATGCCTCC TAGACAATAA AATATATTGA

8521 CATGAGGAGG AACGTATTTA CTAGTTATAT CATCCGCAAT CGCCTGAATC TCGAGACGTT

8581 CTTCAAACCA ATCATAGACT TTATTGAGAT AGGTGGGACT CCCCCTCCGA GAACCGTATA

8641 TGAGACTTTC ATCTCGTACA GCTCAAGCAA AACACCAAAA TACTAGTTGG AACGGATATG

8701 TAATAGAAAG TTTCAAACTT CTTTTATTAA TCTCTAATTC AATCTTCTCT GAAGATAAAA

8761 GGGAAGACGA GAAAATCCGA AAGCTTTTCT TTATGGAGAT AATACCAAAA TATCAAGTTG

8821 GCTCAGTCGT CTTTATCTTG ATCTTTACGG GCCTCCTGAA TTTTCTTTTT CCCTATTGAT

8881 TTTTATTTGA TTTTTTGTAT GGAATGTAGC TTTTATTAGT TGTTTTCTTT ATTATTGATA

8941 GGAATTCTCT TGTTACGACC CCATGAATCA ATTAAAACCT CAGATTCATA CTAGAACCAC

9001 GATGATTCAA TAAAAATCCT AACCTAAGCC CAAGGATTCC CTGAGTAAGA ACCTTTGGAT

9061 CATCACCTAT TCCATGAATA GATATAATGA CTATAAATCG AAAGAAAGGT TTGTCTTTTA

9121 GTCAAACCTT TCTTTCGATT TACAAATTCT AATTCTTTGA AAATTTTTTG GAGTCCGGCC

9181 GCGAGGTTTG AATAGTAAGT ATGAATCATA GTTCAAATCT TTCTTAATCT ATTTCATATA

9241 TTCCGTGTTT CGGATACTAG AATAAATATC CGACGAAGTA GAACCACCTC TATCAAATCA

9301 AGATGACAGA CCCGTTCTCT GTATTATCAA TAGGATCAAT TATAACAAGT CACACACTCA

9361 TATTCCAGAA AAAAAGAAAC AAAGAAATTT CGCGGTCGAA CTACCAAAAA AGAATGGGCG

9421 AAAAATCCAC GTTCTAAATG ATTCTTTTTT ATTGAAAAAC TAGGACTTTG AGGTTCTTAT

9481 AGGATCTAAT TCATTGAAAT TTCATCCAAT AAAACGGAAG AATTATAAAT CTCCAAAATA

9541 ATAGATAGAA ATATAGCAAA TAGGGCCATT GCGACACCCA TTAAAGGAGT CGTTCCCCAC

9601 CCCGGAGCTA CTTTACCATA TTCCGAATTC AGTGGCTTCA ATAATTTCCC TACAGCAGTT

9661 GGTTTTGGAC GAGATCTAGA ACCGCTCTCA ACAGTTTGTG TAGCCATAAA TCCTATTGTA

9721 TTCATTGAGA TCTGTTGACT TTGTATACCA TTCTGTTGTA AATAAACGAT CTTATCATAG

9781 ATCTGTTATA GTCTTGAAAT TGTAAAATAA TGGAAACAGC AACCCTAGTC GCCATCTTTA

9841 TATCTGGTTT ACTTGTAAGT TTTACCGGGT ACGCCTTATA TACCGCTTTT GGGCAACCTT

9901 CTCAACAACT AAGAGATCCG TTCGAGGAAC ATGGGGACTA GTTGAAGTAA TGAGCCCCCC

9961 AAATTTTGGG GGGCTCATTA CTGCAATTGA GATAATGAAA AATCATTTCA TCTTTTTAGT

10021 TGGAACTTTA CTTTTTTGTT GGAACTTTAG GAGGTTCTCT AAAAAAGATG GCGAAAAAAA

10081 TGATACCTAG AGTCGAGACT AAGAGGAATG TATAAACCAA TGCTTCCATA GATTCGATCG

10141 TGGTTTACAA TTATAGCTTC CCTACCTGTT TATTTTTTGT TTATTTTTCT TTATGATCAT

10201 CTCCCGGAAA AAGAAaGAGC AAGAATCAAA AAACGGCGAT TCAAATCAAG ATTTCTCTGG

10261 TACCCTATAG AAAGTAAAGA AGAAAGATCA AATCACAAAA AGAAAAATAC AGTCGAAAGA

10321 CACCAAAGCA ATGGTGTATC AGACTACCTG TCTTCTTGTA GTAGGATCTC CTAGTTTTTG

10381 GAAGGCTCCA AATTCTACTT GAGCATCTAA ATCTGGGTCA ATACCAGCAA AAACATCTCT

10441 GAACAGGGTT CTAGCACCAT GCCAAATATG TCCAAAGAAG AAGAGCAAAG CAAAAGAAGC

10501 ATGTCCAAAA GTAAACCAAC CCCTTGGGCT GCTACGAAAA ACACCATCGG ATTTCAAAGT

10561 AGCACGATCT AATTCAAAAA TTTCACCCAA TTGCGCGCGT CTAGCATATT TTTTCACAGT

10621 GGCAGGATCA CTATAACTGA CTCCGTTGAG TTCGCCACCG TAAAACTCAA CAGTTACACC

10681 TACTTGTTCG ACACTATACT TCGACTCGGC CCTTCGAAAA GGAACATCGG CTCTAACAAT

10741 TCCGTCACCA TCTACCAAAA CGACCGGAAA GGTTTCAAAA AAGGTAGGCA TACGACGTAC

10801 AAAAAGTTCA CGCCCCTCTT TATCTCTAAA GATAGGATGT CCTAACCATC CAACCGCTAT

10861 TCCATCCCCA TTATCCATTG AACCCGCTCT GAATAATCCC CCTTTTGCGG GATTATTGCC

10921 AATGTAATCA TAAAAAGCCA ATTTTTCAGG AATTTTAGAC CAAGCTTCTG ATAAACTTTG

10981 ATTTTCGGCT AGCCCAGCAC TAACTCTTCG ATATATCTCT TGCTGGAAGT ACCCCTGATC

11041 CCATTGATAA CGAGTAGGCC CAAATAATTC GATCGGGGTA GTTGCTGAAC CATACCACAT

11101 AGTTCCGGCA ACAACAAAAG CTGCAAAAAA GACAGCCGCA ATACTACTGG AAAGCACAGT

11161 TTCAATATTG CCCATACGCA ATCCTTTGTA TAGACGTTGG GGCGGACGGA CGCTAAGATG

11221 GAATAGACCT GCCAATATGC CCAATGTACC TGCTGCAATA TGATGAGAGG CTATTCCTCC

11281 CGGAACAAAA GGATCAAAAC CTTCCACGCC CCACGCTGGA TTTACAGATT GTACTTTTCC

11341 CGTTAGTCCA TAAGGATCGG ACACCCATAT TCCAGGACCA TACAAGCCTG TTACATGAAA

11401 TGCACCAAAA CCAAAGCAAG CCACCCCTGC AAGAAATAAA TGAATTCCAA AGATCTTGGG

11461 CAAATCCAAA GAAGGTTTTC CTGTACGTTC GTCAGAAAAG ATTTCTAGGT CCCAATACAC

11521 CCAATGCCAA ATAGCTGCCA AGAAGCACAA GCCAGAAAAC ACAATATGTG CCCCGGCCAC

11581 ACCTTCGTAA CTCCAAATAC CCGGATTCGG CATAGCCCCT CCCGTGATAC TCCAACCGCC

11641 CCATGAAGTA GTTATTCCTA AACGAGTCAT GAAGGGTATA ACGAACATAC CCTGTCTCCA

11701 CATTGGATCA AGAACAGGGT CAGAGGGATC AAAGACTGCT AATTCATATA GAGCCATCGA

11761 ACCGGCCCAA CCAGCAACCA GAGCTGTATG CATTATATGG ACCGAAATTA ACCGACCGGG

11821 ATCATTCAAT ACAACAGTAT GAACACGATA CCAAGGCAAA CCCATGGAAA TACCCCTTCT

11881 ATCAAAGACA AAAAGACACT ACGTAACTTT ATTGCATTGC AAAAACTATA CTATGACTAT

11941 GTTATGGACC TCCCCTGTTC AGGGGATGGT TTCAGAGCAA GGGTTAATAT TTGTTCTATT

12001 CTGTTGGTAA TAATGGAACA ATTTCATTCG TAGGAACAAA GAGAAGCAGG TTTATTCTAT

12061 ACTCGATAAG TAACAATACG CAATGGGGGG TTGATACCGT TTTCTATGAG CGAATGGGCC

12121 CATACTTTTT CATCATTAGA AAGGCCCTAT GCGTATTCGT AATAATTTTT TCTTATTCAT

12181 TCTGTCTTTC TTTATGAATT TTTCTAATCT ATGGATAATC TATGGATAAA TGGATAAAAT

12241 ATGATAATAA AATATGATAA AGGACACTAT TCTAAAAACA AGAAACCCAT TCTATTATTA

12301 CGTTTCCACA TCAAAGTGAA AAATAGTACT TAGTTCTTTT TTCTTTCATT TCATGCCTAT

12361 TGGTGTTCCA AAAGTGCCCT TTCGAAGTCC TGGAGAGGAA GATGCATCCT GGGTTGACGT

12421 ATAGTGCGAC TTGTCAGATA TATTGGGTCA TATGGGATTT CCCCGTTCTC TCCCCCGACC

12481 GAGATATCCT CTGTTTCGCC CAAGAAAGAT TCATTGAATC ATCACAAATT TGGAGCGTGA

12541 AGTGCAATTA GATCCATTTT TGGAGGGCTT CAGATTACTA TTATCAATTA GAATAATTTA

12601 TGGTTGTCTT GGTTGAACTC AAAAAATGAA GTATCCAGGC TCCGTTTAGA AAAATCCCAA

12661 TTGGAAATAG ATCTATGATT ACTATTTGTA TCATAAATGG TTGCTTTTTG TATTGTAAAG

12721 AGAAGCCATC TCAAACTCTT AATCAATTGA GTAATATGCA TGAATACATC AAATTTTTGG

12781 CGGAAGACGT AAATTGACAA AGGGTAAGTC ATAACAAAAA GAAGTGGTAA TGGAAACATT

12841 TGTCCTATAT GTACAAATCA AAATCGGGCG GATCTTTACC CGGAGTAGAG CATCAACCTA

12901 AAAAGATTAA AGGGCCCATT CAGGAACAAG AAAACGACAT CGTGATTTGG ATTGGATCTC

12961 GATGAAACAG TATATCAATG AGAAGTTCAT TCGATAAGTT TAGTGGCTTC TTTTTTTATT

13021 ATTTTTCTAT TATAAGGATA TTAATTATAA GGAAAGGCAC AAAAACTCAC CTTTTTTGAA

13081 AAATCGAAGA AAGGCCCTTT CGTTAGAAGT CAGAAAGAGC CTTCTATGAA TATGAAAAAA

13141 GAAAGAGGAT TGGAATCTGC ACATTGATTC TTTTTTTGCA ATTTTTGTTG AACCGTATGC

13201 GCCAAAAGAC GCCTGTACGG TTCCTAAGGG AGACAATTTT CCCCTAATCA ACCGACTTTA

13261 TCGAGAAAGA TTACTTTTTT TAGGCCAAGA GGTTGATAGC GAGATCTCGA ATCAACTTAT

13321 TGGTCTTATG GTCTATCTCA GTATCGAGAA TGATACCAAA GATTTGTATT TGTTTATAAA

13381 CTCTCCTGGT GGATGGGTAA TCCCTGGAAT AGCTATTTAT GATACAATGC AATTTGTGCG

13441 ACCAGATGTG CAGACAATAT GCATGGGGTT AGCCGCTTCA ATGGGATCTT TTATCTTGGC

13501 CGGAGGAGAA ATTACCAAAC GTCTAGCATT CCCTCATGCT TGGCGCCAAT GAGGTTTTTT

13561 TTATTTGAGA GAAAAAATAA GACTATGCCT TCGCCATATG AATATTAAGT AATAATAGCA

13621 TGGCACTTTG AATTCGATAG AAAAAAAACT TTTTTTTTTC AAACATTAGA TTATGTATCG

13681 AGAGAGTAGT ATGAGATAAG AGGTATTTCC TATTTTTGTA TTGGGGATTC CAGTTCAGCG

13741 TCACAAACTT TTTTATTTTC ACACCGGGGA CTCTTAACAA CAGTTATGTT CTGAAAGAGT

13801 TTGAAAAAAA AAAAAAGATT ATTTTTTCCT TATTTGACAA AAAGCTACTT TGGGATTGCT

13861 GAATCACAGA CAAACCAAAT AATATAATAA ATATAGAAAG CAACGGAACC ATCATAGTAT

13921 TTTTGAACTC CTACGAAGGG AAGGGTGGTA ATTTGATCAT TTACCGATCT GGGTCTGATA

13981 GATCCGAGTT TTCTCTTTCT TTGGGTAAAG GTCAATTTGA TTCTAGAGCC GTATGCAATG

14041 CACAAAAGAT GCCCGTACGG TTGTTCAATT CTGTCTTTTT TTTTTCTTCT TTTCTTTATC

14101 TTTCTTTTCT ATTCATCATG CAAAATAGAG AACTCCTTTT TTGTTATACC ATCAGGGTAA

14161 TGATTCATCA ACCTGCTAGT TCTTTTTATG AGGCACAAAC GGGAGAATTT ATCCTGGAAG

14221 CGGAAGAGCT GCTCAAACTG CGTGAAACCC TCACAAGAGT TTATGTACAA AGAACGGACA

14281 AACCCTTATG GGTTGTCTCG GAAGACATGG AAAGAGATGT TTTTATGTCA GCAACAGAAG

14341 CCCAAGCTTA TGGAATTGTT GATCTTGTAG CGGTGGTTGA GTGAAAATAG CCCAGACTTC

14401 TTTCGCGTAA ATTCTCGATT TCCTATTTTC TCTTTTTGGC GAATTTACTA GAAAATTCAA

14461 GAGAAGTAAT TCATTCAAGA GAAGTAATTC ATAATCATCC GGTTAGGATC GATCTAAACC

14521 AGCCCATTAT TTATTTTATG ATTCAACATG CCAACTATTA AACAACTTAT TAGAAATACA

14581 AGACAGCCAA TCAGAAATGT CACAAAATCC CCCGCGCTTC GGGGATGCCC TCAGCGTCGA

14641 GGAACATGTA CTAGGGTGTA TGTGCGACTC GTTCAGATCA GGAGCTGGGC TAAAAGAAAG

14701 AAAACTTTTT CCAGTATCAA TGATCAGTAC CGGCGAATAG GATAGAATAG AAAAAGAAGT

14761 CCATTCAATT CAGTATCTAA AAAAAGCGAA TCTATCCATT CTGTTGTGTA TTAAATTTAT

14821 GGTTTCCATT GGTGCAAATC CAATCACCTC AATTAAAGAT GAGAAGCAAT TCTCCATTGG

14881 TAGCAAATGG TTATCCATTA AGCGGAGGAA ATCTTACTTA AAAACAGAAA GATTAGGTCC

14941 CCTCTTGCAA GAACGGACTA ACAGGGTTAG CTACCCAGCC AACTTTCATA ATTAAATACC

15001 GTTACTGTGT AGGTAGATCT CATCGTGAAA GACCTATTAC TGGATAATTC ATGGGTAGAG

15061 CCAAAGAATG TGAACTATAC AAGTTACCAA TAAAATTGAT TAAATGAAGT AAAGGCTCCG

15121 GTGTATAGAG AAAACCTCAC CGTTTAAGAA GTAACCATAT AAACGAAGGA AGCCGCTATT

15181 TCTTTATCCA TTTCTATTTT TTTATTTACT CTATTTTTTT TTTGATACTT AGTAGAATAA

15241 AATTTATTAT TTCGAAGTGA AATGTCTAGA AAAAAGAAAA ATAGTGGTCG GGAAGGTTAT

15301 AGTAGCCAAA GCCATTGGAA TTATTAATTT ATACATTGGA AAAAAGCTTT GTTATTAATA

15361 GACTAGGAAA GGGAAAAAGA ATAACTGAAA GAAAGGAAAT CTATTAGTTA TTCGCCAAAG

15421 TTTCATTTAT TCAATGACCA GAATTAGACG GGGATATATA GCTCGGAGAC GTAGAACAAA

15481 AATTCGTTTA TTTGCATCAA GCTTTCGAGG GGCTCATTCA AGACTTACTC GAACAATTAC

15541 TCAACAGAAA ATAAAAGCTT TGGTTTCGTC TCATCGGGAT AGGGGTAGGC AAAAGAGAAA

15601 TTTTCGCCGT TTGTGGATCA CTCGAATAAA CGCAGTAATT CGTGAAATGG GGGTATCCTA

15661 TAGTTATAGT AGATTAATAC ATGATTTGTA TAAGAAACAG GTGCTTCTTA ATCGTAAAAT

15721 ACTTGCACAA ATAGCTATCT CAAATAGAAA TTGTCTTTAT ATGATTTCCA ATGAGATCAT

15781 AAAAGAAGTA GATTGGAAAG AATCCACCGG AATAATTTAA ACGGAGTTCC CCGGAGAATG

15841 AACTCCGGGA GGGTAGAGTC AAAATGATAT GATAAATAAA GTAGTAAAAA TGAATGAACA

15901 CATTCAATAA AAAAAGATTT CTTCTTCCCC AATTCTTTCC CAATTCTTTT TTTTCTTACG

15961 ACACGATAAT GAAATCTAGA TTTTATTTTT TTTTGAACAA AACATCAATC CGCGTTTGAG

16021 TTCGGATTGG AATTTTGATT CAAGTGACGA AAGAGTAAGC CTATTTATTT CTGGCTCGAA

16081 GACCAGCAGT TCTAGCGGTC GACTCGGTTC TTTCAAATTG TTTCTCATTA TTAAGAAAAG

16141 GTAACAAAGA TAAAATACGA GCTTGTTTTA TAGCAATAGT AATTAATCGT TGTTGTTTCA

16201 AGGTCAATCT ATTCACCCGT CTAGATAATA TTTTTCCTTG TTCACTAATA AATCGACTAA

16261 TTAAACTCAT GTTTCTATAA TCAATTCGAT CCCCCGATTG GATCGGGGGC AAACGCCTAC

16321 GAAAAGATCG CTTGGATTTA AGAAAAGGTC GCTTGGATTT ATCCATGGTT TGTTTAGTTT

16381 ATCCCTTATC CCGAAAATCC TATTTCGATC GGATCGAAAA TGAATTAGAA TAAATAGGAT

16441 TTGGTTTGTT TCTATTTAAC GTTTCTATTT AACTATGTTA TATATAATAT ATATATATAT

16501 TATATATAAT TATAATGTCA TTTTTTCTCC TTCCAAGGAA GGGTTACATA CAGACAGGTG

16561 TACGATTCGA TCTATTTCTT TATCTCCCCA TGAATCGTAT GTTTGTAACA GTATGGACAG

16621 AATTTTCTTA ATTCCAATCG ATTAGGCGTG TTGTGCCGGT TCTTTTGAGT AATATATCTG

16681 GAAATACCCG TTGATACCTT ATTTTCATCG TTTCGAACAC AACTAGTACA TTCCAAAATA

16741 ACCGTTATTC GGACATCTTT CCCTTTGGCC ATGAACCCCC TTTGGATTTT TGATTTACTC

16801 AACTCTTCTA TTTTCGATCC GAAACGAAGA aGAAGAAAGG AAGGAAAAAT AGAAGTTACT

16861 AACTTGAAAT CCAATTATAA AAAATTTCGA TGCAATGCAA TAAAAAATAA ATAGAATAAA

16921 TAATATTCTA TAATATAACG ATTTCTAAAC TTTATTTCAA ATCGCAGTAT TTGATTTACA

16981 TTTAAGTATC TTGTATAAGA GTCTTGCCCT AGACCTCAAC CCCACATTGT TTATTCTACC

17041 TCCATCACTA TCACTATTTA ATTCAAATTT GAACCCAAGC CTCGCAGCTG TTGAATCCAT

17101 TTTTTCTTTT TTTCTCTTTC CTCCCTTATT CTAAAAAGAA AAAGGGAAAA AAGAAAAAAG

17161 GGGGGCGAAG GATTAGTCAC AAATAGTTAA TTTGATCTCG AATCTTCGTT ATTTGTTACC

17221 ATGTCAATAA CTAGAATCAA AAAAAGGGGA ATGTCAACGC ATCTGGGAAA AAACGATTAA

17281 TCTCTATCAA GAGACCTGCT AAAGACCCGA ACCATAGAGT ACTTAATACC GGCGCCACGG

17341 AGAGATATGT TTTTAGATCT CGCATTGAAT GAAAAACCTC TTTCCTTCTT TTATTGTAAT

17401 ATCTTTTTTT ATTGTAATAT AAAATGGTAA TACATATATG ATCAGATGCA TATGCAGTTA

17461 AGGACCGCTT ATAGTTGTAC ACGGAATCCC CAATTCAAAT TGAAATGTAC AACTATCCGG

17521 TGAATAAGAT TTTTTTCTTA AAACATAAAA AAAAGCATAC CCTTCTTCCC GAGCATTCCC

17581 GAAAACCACT CATTTAGTTT TACGACAGGT TCCGTTCCGT ATTTCATATG TATAAATATA

17641 AGTCTTTTAC TATTATTATA TGTTAAATGG GACCAAAAAG ACGAAATGTC CATATAAATT

17701 ATATATATCA ATGGCGGAAA TAACGATGTG GAAAACAAGA CAGGAATTAT CTACAATGAC

17761 ACTGTAGACC AATTGAAGGG ATGTAGCGCA GCTTGGTAGC GCGTTTGTTT TGGGTACAAA

17821 ATGTCACGGG TTCAAATCCT GTCATCCCTA CCTATTACTT CTCCGTTGAG CAGTAACGAG

17881 GGAGTAATTC AGATCTATTC AAATTGAACA TATCTAATCG TTCATTCAAA GAAAAGATGT

17941 ATTGGGGTTA AAAAAAGTAT CTTTACAGCC TTCGCTTTTC TGATAAGAAA GCGCTCTTAG

18001 TTCAGTTCGG TAGAACGCGG GTCTCCAAAA CCCGATGTCG TAGGTTCAAA TCCTACAGAG

18061 CGTGAGTTCG CCCTTATTAT TCTTAGATCA AATTAGACTG AAAGAGCTTC ACTAACTTGA

18121 ACCGGAATCT TAATTTGACC TCCCTTCTAT TAAAGAAAGT AGGAGGTCAA TCAAAAAAAG

18181 AGATAATAAT TAATCAAAGG TCCGACTGAT CACCCCGTCT ATATTGTAAA TAGGCAGTTA

18241 CGAATAATCC AGCCAAAGTA ACAGGAATTA GGCCTAACAC GATTCCAAAT AGAAAAACTT

18301 CAATCATTTC AATTTGTTTG AAAGGAGAAA AAGGAGGTAA TATCTATATC TAAATATTAA

18361 TTCGAATTAC CACGAATCTC AATGACTAAG AATTGGCAAT TTACACAGAA TCCTATCGAA

18421 AGGTTTCTTT TTATGAATTG CGCATTTCAA AGACTCATTT CAGATAAGTC GTATTTTGCT

18481 CAGACCAATA AATAGAGCCG AGGTTATCGT TAAAGACGCT AGTAGAAAAC CAAAATAACT

18541 AGTTATAGTA GGCATGAAGG AACTAAATGA AATATGTTCT TTTTATACAT ATGTTTATAA

18601 AAAAGCACTT ACCTAAGTTT CCCTTTTTTC AAAATAGGAG ATAAGCCAAA ATACCAATTG

18661 AAAGAATAAG TGAAATTGAA CGTTTTTGAT TTATCTATCA TTATAGACGG TACTAACAAA

18721 GATAAAGTGA CAGGCGTATC AAAAGAAATT GATTCATCAT CTACGACATC TAACCATCCC

18781 GCGATTCCAA TCAACCGATT TATTGAACAA CTCTTGGGTA AACTTATATT AGAAACTAAT

18841 AAAAAGAACT GGGCCGTATA ACTTCACTCA AAAATGAAAC TCTTTTTGAA TCATTACTTT

18901 TTGGAAGAAT GGAAGAAAAT GTTTTCTTTT TGAATTCTAT CGAATCCATT TTCCATTTTA

18961 GAGCAAACAT AAACCTTATA TTCTAAAAAA AAATGTTTCC TTTCATTTTT TTTAACTCAT

19021 TAGATTCTGT ACTAGGTTCG TTCACTTAAT ATCTTGAGTG ACTGAGAAGA AAAAAGCTCT

19081 CACGCGATCA CTCGAAAAAG GCAAATCTTT CTTTTGGTCC AACTAGGTTC TAAGACTAGT

19141 CATTTCTATT CTCTTTTCCT TTTTTTACGT TCTACATTTT ATCTTTCTGT ATAAAACCGC

19201 TTCTACACCT ACAAAAAGGA GGATTTCTAG ACCTCGGATC TACTTAATGC GGGAAGATGA

19261 TAATATCTTT CTTCATGACA GACCTGTCAG GTTCATATTT TATCCGATCC TCAGGTTCTA

19321 CCCCGAAACT AATAATGGAG AGAAATATAC TATTTTGCAG AATTTTATTA ATTTGATATT

19381 GAACGGGACA TCATTGTACA CTGATAAGTA ACAAGTAAAG TAAGAAAGAC CTTTTTTAGC

19441 ATTTCCTTTC GATACTGATT GAAAGTGCGT TGCTGTGTCA GAAGAAGGAT AGCTATACTG

19501 ATTCGGTATA CTCTAAAGAC GCCCTCGGTA CAATATTGAC GATCTCACAA GGATTTAGTT

19561 TTAGTAAATG GAAATTGACT GATCTAATCT TTTACGGAAT CGATCCCCCT TTGACTGTAC

19621 AATAATATGT GGAGCTCGGC ATGTCTGGAA GCACAGGAGA ACGTTCTTTT GCTGATATTA

19681 TTACCAGTAT TCGCTACTGG GTCATTCATA GCATTACTAT ACCTTCCCTA TTCATTGCGG

19741 GTTGGTTATT CGTCAGCACC GGTTTAGCGT ACGATGTGTT TGGAAGCCCT CGGCCAAACG

19801 AGTATTTCAC AGAGAACCGA CAAGGAATTC CATTAATAAC TGGCCGTTTT GATCCTTTGG

19861 AACAACTCGA TGAATTTAGT AGATCTTTTT AGGAGGCCCC AATGACCATA GATCGAACCT

19921 ATCCAATTTT TACAGTGCGA TGGTTAGCTG TTCACGGCCT AGCTGTACCT ACCGTTTCTT

19981 TTTTGGGGTC AATATCAGCA ATGCAGTTCA TCCAACGATA AACCTAATCC GAATTATAGA

20041 GCTATGACAC AATCAAACCC GAACGAACAA AGTGTTGAAT TGAATCGTAC CAGCCTCTAC

20101 TGGGGGTTAT TACTCATTTT TGTACTTGCT GTTTTATTTT CCAATTATTT CTTCAATTAA

20161 GAAAACAAAG GATAATAATA ATTATAGGCA CTCTCTCTTA GCCCATTCGG AAGGATCTCA

20221 TCTCATAATT ATCCATGGCT GTTTATGTCT CTAGCATGAC CGCTTGATGA AATGTGGAGG

20281 GAAGTGGGGT AAATGGCTGA TACTACTGGA AGGATTCCTC TTTGGATAAT AGGTACTGTA

20341 GCTGGTATTC TTGTGATCGG TTTAATAGGT ATTTTCTTTT ATGGTTCATA TTCCGGGTTG

20401 GGTTCATCTC TGTAGTAATC AGATGAATTG AGTTGTAAGT TGTAGCCATG AAAGCGTAAG

20461 AACTCAACGG GACCTCCCCC CTCAAATCAT ACAAACAAAG AAGGGAATCC TGTTGAGTTC

20521 TTAAGAATTC AGAATCCTTT TTCGTCTAAA TGTCAAAACA AATTTCTTTT TCTGTTGTTT

20581 CAAAAAAATC CATTCCATTT TTTTCTGATG GTTTTGAAAA ATGAACTTCA TTGCCCCATT

20641 CATCTATTCC TCGATAGTAT CTATCGATAC TTTCAAAGTT AGAAGAAAGA AGGAATCACT

20701 CGATTTCCTG GATGACATAC TATCTATTTC TGTAGATTAG ATATCCTAAA AAATTTTCTA

20761 TTATTATAAA ACCTTGTACT AATAAACCCT TAGTCCTTTT TTAGTATCTC ATAAAAAATG

20821 GAAATTTTTC TAAGTTTATT GAAGAAGTTC TATTTACTCA ATAAACCTCC CGCTCTTTCT

20881 TTTGTTTGTC CACCACTATA TATTATACGT AATATATATC AAACTATATA AATGATATGT

20941 CTGATCTATT TTGAAAAATT CAAAACTAGG CTAGGAATCT TTGACGAACA GGATCAAGAA

21001 TCCTTTTTTT ATTTCGACAC AAGAAAGAGG TGTGGAAAAT TCCTTTTCTT GTGTCGAAGA

21061 CTAGGAAGAC GAATAATCGT CCCTAGAATT TTTTGTTCCA CGCATTTATC ATTTATCCGT

21121 TCTATTCCCC GCTTACTTAT GTCTAGTATC TAGTCCACTT TCATTCGATT CGAATAGAAA

21181 ACACTATTGA TGCATTTTCT GACATTGAAA TGCGAGAATA GTAACTATAG TAATACCGCC

21241 CCCATTTTAT TTGGATTAAA AAAACAAAGT TAAACAGCCT TCTTCACAAT CTACAGACTA

21301 TGACTAGGAA TGCGATCCAA GGAATTCCCG GCATCTCGTA GAAAAAAACG AGGATAAATA

21361 AGAAAAAGGC TTTTCATTTT CATAATGTAA TTTTTTTATC AAAGAGAATT GTCAAAAAAA

21421 AATTGTTATT TTTACCTACG TCTTACGTTA TGAGTGCAAT GTTGATAAAT CCGCGAATCT

21481 AGAAATTCAT TTCTGACAAT TGAACCTTCT CGAACTGTTT CTTTTTAAGA ACCAAAAAGA

21541 TTTGTGCCAA AATAACAGAT GCCAAGAAGA ACAAAAGGCC TTGGACACGT AAGGGATCTT

21601 GAAGTACTAT TTCCGCATCT CCCTGACCAA ATCCGCCCAC ATTAGGATTA CTTGTCAATG

21661 GTTGATCCAA TTTGATAGAT TCGCCTTCTG AAACAAGAAG TTCTGGCCCC GGAGGGATAA

21721 TATCAACCAC TTGACGTCCA TCCGATGTAT CCGCTATGGT TATCTCGTAA CCCCCTTTTT

21781 CTTTTCGTAT TATTTTACCT ACTATACCTG CTGCTGTAGC ATTATAAACT GTATTATTAC

21841 TCTTGCTCCC GTCGGGATAA ATCTGACCCC TTCCCCTGTT TCCGCCTACG TATATAGGAT

21901 ATTTTAAGAA GTGAGCCTCT TTCTTAGTAG CGGGGTCCGG GGAAAGGATA GGAAAGGTGA

21961 TTTCACTATA TTTCTGACCA GGAACAGGGC CTATCACAAG AATATTTTTT TGATTGGGGC

22021 GATAGCTCTG AAAAGACAGA TTGCCTATCT TTTCTTTCAT CTCGGGAGAA ATCCGATCGG

22081 CAGGAGCTAA TTCAAAACCC TCCGGTAAAA TAAGAACAGC CCCTACATTC AAAGCACCTT

22141 TTTTACCATT AGCAAGAACT TGTTTTACTT GCATATCATA AGGGATTCGA ACAACCGCTT

22201 CAAATACAGT ATCCGGAAGT ACCGCTTGCG GAACTTCAAT ATCCACGGGC TTATTAGCTA

22261 AATGGCAATT GGCACATACA ATACGCCCAG TCGCTTCTCG TGGATTTTCA TAACCCTGCT

22321 GTGCAAAAAT GGGATATGCA CTTGAAATGG ATGTCCGAGT TATGATATAT ATCATAAGCG

22381 ATACGGAAAT GGATCGAGTT ATTTGTTTCT TTATCCAAGA AAGAGTCTTT CTAGTTTGCA

22441 TGGTCCAACC GTTGATCCCG AAAATTGATA CAATAAATTC GGTAGGTCGC TAATCTAGTT

22501 CCCTATCCGC GATTCTGCTA TTTACTGGAA TAATTTTACT GGAATACAAA ATATTACTAT

22561 TTAATATATA CTTTGGGATA TATATAATAA ATCTTTGGGA TCTTTGGGAT GGGCAGAAAT

22621 ATAATATAAA AAGTAAGTCT GATTTTCCAT CCTTTGCTTA TGTACAACTA CAAAGTAAGC

22681 AACATTAGAA TTGAATTGGA TTAATATCAG TGGATCCTTT TTTAATCATT CATTGAATGA

22741 TAAATCACTA CAAGTGACGG AGAAACACGA TTTAAATAAC GAAAAATCCA AAATTTAAAA

22801 ATTGTATCTA GAATGACTGG AAAAGTAGAA ACAAGACCAG ATACAATTTG ATCATTATGA

22861 ACAAATCCAA AATCTTTGTA GACAGAGCCA ATCATCAGTT CCCAACCATG GGGCGAATGG

22921 AATCCGATAC ATAAATCGGT TAATAAAAGA ATAGAAAAAG CTTTTATTGT GTCACTTAAA

22981 TTATAGAGGA ATTCTCGAGC CCAAGAGTTA AGAATAACAA GTTCTTCATT ACCCAAAATA

23041 GAATAACCAC TTAGAATAAC GAAACAGATT AGATTTGTCG AGAAGTGTAA AATCGTGTGG

23101 ATACGATCCT CGTTGTGTAT CTTGATTAAT TGGATCGTTT CTTTATGGAT TCCTATACGA

23161 AGGTTTTGTA GATGTGTCTC CGAGTATTCC TTGATCATTT CCTCCAAGAG AAGGATTTCC

23221 TCTAATTCTA TGAATTTTTC TAGAATATTC TTTTCTTGAA TATCATTCAA AAAATTTTCG

23281 GATTGCCTAG TATTCCACCA ATAAGTAATC CAAGATTCCA GACTTTTATT AAATGAGAGA

23341 GAAATACCCC AAGGCAAAAA TACTATAGAT GCAAGATATA AAAGAGGAGT GAGTGCTTTC

23401 TTTTTTGCCA TTTTTCATTT CATCTATCAA TTTTTAATGA ACCGTCCTCA TTAGTTGACT

23461 CTACGCGATT CAATATTTAT CTCATGCATT AGTTCTATTA TTATATTACT ACAAGGTATC

23521 GAACCTATGA ATCTATTTTT TATTTATGAT TTCGCAGTTA GTCTTTGAAT GTATAAAGAA

23581 GACAGAAATA GATTAAGAAT CCACTTCGAA TTCGAATGAA GAAATCATCA ACAAAAAAAT

23641 ATTGTTTCTA GATATCGCAA TTAGTCCAAT TCGATTTGGT CATTAAGGAA CACAAAAGAA

23701 AGTATAAAAA GAAACGTCGA TTGACAAAAA AGAAAGAATT TCCAAAATAG GATCTATCGG

23761 AATCTAAACT ATCAATTCCA ACAAATAGAA TCTATTTTTC GATTTGTTAC TTATTTTGTT

23821 ATTGAATCGA GTTGTGTAGA TTTCCATACA CATAAAAAAC CGTAAAAAGA GTTTTGTTTT

23881 ATAATTCTTC CGTATACGTC TTCTTTGACT CGAATGAGCG TTATGAAGAA ACTTCGGTTA

23941 AATTATTATT ATTATGTTAT AGAAAAAGGA GGGGATTCTT GAGTTTTTCC TTTCTGCTTA

24001 GAAAGCGTTA ATTCTCTCAG CAAATTTTTC TCAAAAAACT TCAATTGGTA CACGCAAGAA

24061 ATAGGCCAAT TCGGCAGCTT TTTGCTCAAT TTCCCGCGGA GTAACATTCT CATCAGTACG

24121 AGTCAAGGGA ATGGCCCCCT GGCCTCTGAT GTCCATATAA AGGACACGGC GAGCATAAAT

24181 ACCCTCTTTA ACTTCTATTC TGACGGACTG AATATCCTTT ATAAGAAATC GGAGGAAGAT

24241 GCGACGATTT TTTCCAGGGA ATCCCCAACG AAAAATACAC ACCATTCCTT CTTTTCTATC

24301 GAATCGATCA TAACCGCCCC CTACATTCCA CGAAATTGTG CACCACAAAT AGGAGCTAAT

24361 AAAGAGACCC GCGATCCCAT AAAAAGACAT CACAATCCCT TGTGGAAAAA AAAGGATTTG

24421 CTGAGACGGA AATAAAGATA TCAAGTTTCT ACCAAGATAA CTGGAAGTTC CAACCAATAA

24481 GAATCCTAAT GAACCTAAAA AAAGGATAAC GGCCCAGCAG AAATTGCTTA TTTTTCGCGA

24541 CCCCGTTATA GGTTCTATCC ATATATGTTC TGATCGACAA CTCATAGTTG ATCCGGTTTG

24601 TTTCGTTTTA TTGTAATTGA GAGAATACCC CAGACTTACC CTTTTTGTTT CCGAAATAAC

24661 TCTCATCAAC TAGTAATAGT TTGCTGTGAA CCTTTAAGAG TAATTCATCA AATCAAATTG

24721 GTTAGATCTA AAAGAAAAAC ATACCCTTCA TATGATCCCC TCGATATACA TATAGATACA

24781 CCGACTTTAC ATTTCAGCCA TCCAGCGATC CTGATAGTTT TGCAAATAGA TAGAATTCCG

24841 TTACCCATAG ATCATCTTTC TACAGGCCTA AGAGTTCCTC CTTTTTGTTC GGCAGAAGCC

24901 ACCTGTTATA ACTTATTCCA CTAAAATAAA TAGATATCTG TGTTATGGAT ACAAATACAA

24961 GTCTAAGTTT TGAAAAAAAA AATGGATGAG AGTTGGGCCC GTCAGATCTA AACAGTCGTA

25021 TTTTTTTGAA CATGAAGAGA TAAAGAAGCC ATTGCCATTG CCGGAAATAC TAGGCCCACT

25081 AAAGGTACCA AAACAGAGGG AAAGTCGAAA GTTGTCATAG AAAGGATACC TCAATTTACT

25141 ATTTGTACCT GTTATATTAT TATTATTTAT TTATTATTTA TATTTATTTA GAATTTATAA

25201 AGATAGAAAG ACTTATAATA AGAATTAATA ATTCTAATTA TTATATAGAT TCTATTAATT

25261 ATATTCATAT CATTTTTATT AAATTCAAAT TTGAATTAGT ATAGATATAG ATCTATAAGT

25321 ATCTATCTAA GTGAGTATAG AATGGACTTA GTCATCTTTG CTACAAATAA AAAAGGAACT

25381 TAATGTTCTA TTCTACTTGA TTTCATTTTG CTTCAAAGGA AAGAAAGCGT GGAGCTGAAA

25441 TAACTCACTC AGAACGCTTT TTAAAGGATT ACGTGGTACG ATTAGATCGA ATAAGCCCTT

25501 CTGGAATAAA TTTTCAGCCG CTTGCGAACC TTCAGGTACT GTTTTATTCA ATGTTTGTTC

25561 AATGACTCTT TTACCCGCAA AAGCAATGTA GGCATTGGGT TCGGCAATAA TGATATCCCC

25621 CAACATACCA AAACTAGCAG TCACCCCACC TGTAGTAGGA GATGTAAGGA TTGGTACATA

25681 GAATAACTTT TTCGTTGATT GATAATCATA TAAAGCAGAA GATATTTTAG CCATTTGCAT

25741 CAAGCTCAAA CTACCTTCTT GCATACGCGC CCCCCCGGAA GCACACGCTA TAATAAGAGG

25801 GAGACATTTT TGAGTAGCGT ACTCAATCAA ACGGGTGATT TTCTCCCCGA CCACGGATCC

25861 CATACTACCC CCCATAAACT GAAAATCCAT AACCCCAAGT GCTACGGGAA TACCGTTTAG

25921 TTGGCCTATG CCTGTTTGAA CAGCTTCAGT TAATCCTGTA TTTCTTTGAT AAGAATCGAT

25981 ACGATCTTTA TAAGGATCCT CTTCCGAATG AAATTCAATG GGATCCAAAG AAACCATGTC

26041 TTCATCCATA GGATCCCAAG TACCCGGATC AATCGAAAGT TCGATTCTAT CTGAACTACT

26101 CATTTTCAAA TGATATCCGC ATTGTTCACA AAGATTCATT TTTGATTTAA AAAATTTCTT

26161 ATAATTTAAT CCATAGCAAT TTTCGCATTG AACCCACAAA TGCCTGTATT TTTGAGTTAC

26221 ATCCAGATCA TTAGAACTAT TTTTTATAGC GAAATCGCTA CCATTCGTAC TGGTACTTAG

26281 ATTGGAATCT CCACTTTCAC CACAAAGGGA ACTAGAAATG TAACTGTTAC TGTCATTGTC

26341 ACTACCACTT ACAATGTCAG TATCAATAAA GATTTGAGAC TGAAGATAAT TGTCAATACA

26401 ACTATTAATG TGATTATTCC AACTATCTTG AGTATCATCC ATGTAACGAT CATAGTGAGG

26461 ATCGTCAGTT AGATAACTAA AAGTCCAATA ACTAGAAAAA GAACTTTCTA GTTCACTCTG

26521 AAAAAAATGA CCACTATCAA CCTCGAAAAT CTGATTTTCA AGATCAAAAT ATATAGAATA

26581 ACTGTTACCC TTACTATCCA TAACTAAAAA AGTTTCATCA GAGATAAAAT TCCGAATGTC

26641 CTTGACGCCA AATAAATGAT CAACATTGCT GTAACTAGAA TTATCACGAC CACCCCAACT

26701 ATGAATATTT TTATTCATAT CATTTCTATT CGGATCTTCA CTTTCGCCGG TATTTTCAAT

26761 AGGACCAAGA CCACCCATCG ATTTACGTAG CCCACACCTG CGTTCGAACT CTTTCTTAAA

26821 CAGTATTGAA CCGAGCCACC ATCTTTTCAT AGAGTTTTTT TGCCCCCTAT TTGCATGAAA

26881 ATACAATAGA TGAATAGTCA TTCGATGAAA AATCATTTAT TTGAATCTCT TTTTCCTGTC

26941 ATAATAAACA TATAAATCCA ATCAAAGGAG TTTCAATATT GAAAAAAGGG ATTTTTGTGG

27001 GAATCAAGGT TACCACTTCA CTACTATATA TGAAAATGGT GGATATGAAT ATGATAGAAC

27061 CTCTTTCCAA TTATTAGATC CAATTCGAAA TTGAATTGGG TTTTCTACTC TACTATGTCG

27121 TGGCAAATTC TCATCGAAAA AAGAAAGATT TGTTTTCTCC TATTTCTCCT ATGAAAATTT

27181 TTTTTGTAAA ATCTCGTCTA CGAACTAACT AATAAAAAGG GAATAAGTTT CTATTCCAAC

27241 ACGGAACGAA AAGGACAATA TACAGGATGG GTAGAAAAAG TTGTATAAAA GAATATACCA

27301 ATCCTAAGGG TCCATCGAAA AAATTGTGGA TCCAACATAC CAATAGAAAC ATTTGAGTTG

27361 TTTAGTTTTC AATTTTGTAT ATTCTTCTAT ATCTAGATCA GTATGTATCT ATCTAAAATC

27421 AAAATAGATG CAATAGGTTC TTTGTTTTCG GCCCAATCTT TTACTAAAAG GATTGGGCCG

27481 AGTTTAATTG CAATTCAATT CAGAAAACGA ACGGTAATTA GAATGAGTGG ATTACAAAGT

27541 ATCCATTGCA GCAAATTCAA ATTTGATCTC CTTCCATACC TCACAAGCAG CAGCTAGTTC

27601 GGGGCTCCAT TTAGCAGCCT CACGGATAAT TTCATTACCT TCACTAGCAA GATCACGTCC

27661 CTCATTACGA GCTTGTACAC ATGCTTCTAG AGCTACTCGA TTAGCTACGG CACCAGGTGC

27721 ATTTCCCCAA GGGTGGCCTA AAGTTCCGCC ACCGAACTGT AGTACGGAAT CATCCCCAAA

27781 GATCTCGGTC AGAGCAGGCA TATGCCAAAC GTGAATACCT CCCGAAGCCA CAGGCAGAAC

27841 ACCAGGTAGA GAGACCCAAT CTTGGGTGAA ATAAATACCG CGACTTCGAT CTTTTTCAAT

27901 GAAATCATCG CGCAGTAAGT CGACAAAGCC CAAAGTGATG TCTCTTTCCC CTTCAAGTTT

27961 ACCTACTACG GTACCGGAGT GAATATGATC TCCACCAGAC ATACGTAACG CTTTAGCTAG

28021 TACACGAAAG TGCATACCAT GATTCTTCTG TCTATCAATA ACTGCATGCA TTGCGCGGTG

28081 GATGTGAAGA AGTAGACCAT TATCCCGGCA ATAATGAGCC AAGGTAGTAT TTGCAGTGAA

28141 TCCCCCTGTT AAGTAATCAT GCATTACGAT AGGAACTCCC AATTCTCTGG CAAATATAGC

28201 CCTTTTCATC ATTTCTTCGC ATGTACCTGC AGTAGCATTC AAGTAATGCC CTTTGATTTC

28261 ACCAGTTTCA GTCTGTGCTT TATAAAGGGC TTCGGCACAA AATAAGAAAC GGTCTCTCCA

28321 ACGCATAAAT GGTTGGGAGT TCACGTTCTC ATCGTCTTTG GTAAAATCAA GTCCACCACG

28381 GAGACATTCA TAAACCGCCC TACCGTAGTT TTTAGCAGAT AACCCCAATT TAGGTTTAAT

28441 AGTACATCCC AACAGGGGAC GACCATACTT GTTCAATTTA TCTCTCTCAA CTTGGATACC

28501 ATGAGGCGGT CCTTGGAAAG TTTTAACATA AGAAATAGGG ATTCGCAGAT CTTCCAGACG

28561 TAGAGCGCGC AGGGCTTTGA ACCCAAATAC ATTACCTACA ATGGAAGTAA ACATGTTAGT

28621 AACAGAACCT TCTTCAAAAA GGTCTAATGG GTAAGCTACA TAAGCAATAA ATTGATTTTC

28681 TTCTCCAGCC ACAGGCTCGA TGTCGTAGCA TCGCCCTTTG TAACGATCAA GGCTGGTAAG

28741 TCCATCGGTC CACACAGTTG TCCATGTACC AGTAGAAGAT TCGGCAGCTA CTGCGGCCCC

28801 TGCTTCTTCA GGTGGAACTC CAGGTTGAGG AGTTACTCGG AATGCTGCCA AGATATCAGT

28861 ATCTTTGGTT TCATAGTCAG GAGTATAATA AGTCAATTTG TAATCTTTAA CTCCAGCTTT

28921 GAATCCAACG CCTGCTTTAG TCTCTGTTTG TGGTGACATA AATACCTCCC TACAACTATT

28981 GAATTAAGAA GTCTCCCAAT AATAACAAGG TCTACTCGAC ATGAATTAGG CGTTAATGAA

29041 ACTTTTTACA AGAATCTTTC AAAAAACCCC CAACAAATAC TAATATTATC AACTAAGCAA

29101 AATGCTTTAT TATTAGACCA TGGTATTTGA TTCGTCAAAT ACATAATTAT TGTATACTCT

29161 TTCATCTATA TGGCGCAACC CAATTCTTAT TTTTTACTTT TTTTTTTAAT TGAAATACCT

29221 AAATAGAACT TGAATAATAA TAAATTCCCT CTTGACAGTG GTATATGTTG TATATGTAAA

29281 TCCTAGATAC CAAAATCTCT GGAATTTGTC TAGGAAAAGA TATAAAAAAG AAAAAATACA

29341 AAAGAGGCTA GACATAATAG GATGAAATAA GAAGAGCCGA TGAGATAGAA AAAATGAATC

29401 GTAAATAGAG TTCAGGTTCG AATCCCATAG ATAATATAGA GATAATATAG ATGGGCAGGT

29461 CGATGTCTAT AATGATAGAC AAATGAAAGC TTTTCTCAAG ATTCTTATAC TTTCGGTTTT

29521 GAAAACGGGT TGGTTGAACT CGAAAATTCA CTCAGTGAAT AAGTAAACAA TTGAATTGAT

29581 TCGATTGGAT GGTACCAACG AAATCGCGCG CTAACTCCCA TTTCTTATTT TGAATTAACC

29641 GATCAACTTG CTATCGGACA TTTCTTTTGG AATTCGATCA TTTTCGCAAA AAATTTCGAC

29701 ATATTTTACT TTATTTATTA TTATATTATT ATGAGAATCA ATCCTACTAC TTCTGATTCT

29761 GGGGTTTCCA CGCTTGAAAA AAAAAACCTC GGGAGTATCA CTCAAATCAT TGGTCCGGTA

29821 CTAGATGTAG CTTTTCCACC GGGCAAGATG CCTAATATTT ATAACGCTCT GGTAGTTAAG

29881 GGTCGAGATA CTGCTGGTCA ACAAATTAAT GTGACTTGTG AGGTACAGCA ATTATTAGGA

29941 AACAATCGAG TTAGGGCTGT AGCTATGAGT GCTACAGAAG GTCTAACGAG AGGGATGGAA

30001 GTGATTGATA CGGGAGCTCC TCTAAGTGTT CCGGTCGGTG GAACGACTCT TGGACGAATT

30061 TTCAACGTAC TCGGAGAGCC TGTTGATAAT TTAGGGCCTG TAGATACTCG CACAACATCT

30121 CCTATTCATA GATCTGCGCC CGCCTTTATA CAGTTAGATA CAAAATTATC TATTTTTGAA

30181 ACCGGAATTA AAGTAGTAGA TCTTCTAGCC CCTTATCGTC GCGGAGGAAA AATCGGACTC

30241 TTCGGTGGGG CTGGAGTGGG TAAAACAGTC CTCATTATGG AATTGATTAA CAATATTGCC

30301 AAAGCTCATG GGGGTGTATC TGTATTTGGT GGAGTAGGTG AACGTACTCG TGAAGGAAAT

30361 GATCTTTACA TGGAAATGAA AGAGTCTGGG GTGATTAATG AAGAAAATAT TGCCGAATCG

30421 AAAGTGGCTC TAGTTTACGG TCAGATGAAT GAACCACCGG GAGCTCGTAT GAGAGTTGGT

30481 TTGACTGCCC TCACTATGGC TGAATATTTT CGAGATGTTA ATGAACAAGA CGTACTTCTA

30541 TTTATCGACA ATATCTTCCG TTTCGTCCAA GCAGGATCTG AAGTATCCGC CTTATTGGGT

30601 AGAATGCCTT CCGCTGTGGG TTATCAACCC ACCCTTAGTA CCGAAATGGG TACGTTACAA

30661 GAAAGAATTA CTTCGACCAA AGAGGGGTCC ATAACTTCTA TTCAAGCAGT TTATGTACCT

30721 GCAGACGATT TGACTGACCC TGCTCCTGCT ACGACATTTG CACATTTGGA TGCTACTACT

30781 GTACTATCAA GAGGATTAGC CGCCAAAGGT ATCTATCCAG CAGTAGATCC TTTAGATTCG

30841 ACGTCAACTA TGCTCCAACC TCGGATTGTT GGTGAGGAAC ATTATGAAAC TGCGCAAAGG

30901 GTTAAGCAAA CTTTACAACG TTACAAAGAA CTTCAGGACA TTATAGCTAT CCTTGGGTTG

30961 GACGAATTAT CCGAAGAGGA CCGCTTAACC GTAGCAAGAG CGCGAAAAAT TGAGCGTTTC

31021 TTATCACAAC CCTTTTTCGT AGCAGAAGTT TTTACTGGTT CTCCAGGGAA ATATGTTGGT

31081 TTAGCAGAAA CAATTAGAGG GTTTCAGTTG ATCCTTTCCG GAGAATTAGA TGGTCTTCCT

31141 GAACAGGCCT TTTATTTGGT AGGTAACATC GATGAAGCTA CCGCGAAGGC TATGAACTTA

31201 GAAATGGAGA GCAATTCGAA GAAATGACCT TAAATCTTTG TGTACTGACT CCTAATAGAA

31261 CCGTTTGGAA TTCAAACGTG AACGAAATCA TTTTATCTAC TAATAGTGGT CAAATTGGCG

31321 TATTACCGGA TCACGCCTCT ATTGCCACAG CCGTAGATAT AGGTATTTTG AGAATACGCC

31381 TTGATGGCCA ATGGTTAACG ATGGCTCTGA TGGGTGGTTT TGCTAGAATA GGTAATAATG

31441 AGATCACTGT TTTAGTAAAT GATGCGGAGA AGAGTAGTGA CATCGATTCA CAAGAAGCCC

31501 AGCAAACTCT TGAAATAGCG GAAGAGAATT TCAAAAAAGC TGAAGGAAAG AGACAAAAAA

31561 TTGAGGCAAA TCTGGCTCTC CGACGAGCTA GGACACGAGT AGAGACGATC AATGCGATTT

31621 CGTAACGAGT CGGTGTGTCC GAATAATCAA AAGAAGTTCG GTTTATACAC TCTATTTTTT

31681 TGTTTGGTTA TGTCGAGTGA ATATAATCAA AAAAAATCAA GCGGAATCCA ATTTTGATGC

31741 AACAAAAAAA AGAAATAGAA AAGATACAAA AGAAATAATA AATTTCAAAA AAAGATGGGT

31801 AGAAAAACTT ATTAGATACC GGGGTCAGTG GATCTAATAA GTTATACCTA CTATTGGATT

31861 TGAACCAATG ACTCCCGCCG TATGAAAGCA ATACTCTAAC CACTGAGTTA AGTAGGTCAT

31921 TTATCATCAC AAAGAAAACC AAATGGGACC CGTCCCTTCG ATGGATTATA AATACCATAT

31981 TACTTATAAG CAATACCACG CAAACAGATC AAAGAGATAT GATCTTGAAT CAGGGTATAT

32041 TCATCTTGAC AAGAAATTAT CTACATGATA AAATATGTAT CACAAGCACT AAGGGCTATA

32101 GCTCAGTTGG TAGAGCACCT CGTTTACACG CGCGCCAATG TTTTTCAGGG GAGTCCATCA

32161 TGCAATCAAA AGAATTGATC TTATTGATTG ATAAATCAAT GTCTTACTCC ATAACTTTGA

32221 GGGAACAAGA GAACAATAGC CTGACATGAC AAAAGGGTTC TCGGGTGCCC CCCGTTTAGG

32281 TGCCAAACGG ACCCCATTGT TGATTTGAGA TATTGATAAG GTAAATGTTG ATTCAATGCT

32341 AGGCATAATG AGTATAAGGA CCTCAAAAAA TCTCTTTTCG CCCTATGAAC TTTAAGGTGT

32401 ATGAAGTTTT ATATTTTCTT TTTAAGCAGA GCGATAGAGA CGTCATCTAA CTTAGGTTAA

32461 TCTAGGCCAG AAGCAAACCT ACGTCAAGAT AACCTCCTTT GAAACACTTT GGTAGTGTTC

32521 CTGGATCAGA ATTCAAATAA TGACTCAGAG CACATGGAGC CATCTCCTAT TTTTCTATCA

32581 GAAAAATATG GCGGACTAGC TGATATTTCT ATCAGTTAAT GGAAGAGCCC AATGCACAAA

32641 AAACGCATGT TGGGTCTTTG AAACAGTTCA GATCATTTTG ATAATAAAAA GTTTGATCTG

32701 TTTTACCGAG AAAGTCTACG GTTCGAGTCC GTATAGCCCT AGAGCCCTAA AAAAAAAACT

32761 TCCTAAAAGA TCCCTATAAG CTCACTCACG GGGATCTTTT ATAAAGCAGA ACATCAAAAT

32821 GAAAGCAAAA ACACATTTCA TTTTAATGGA CTTAACCGAT ATTTATCTAA TCGTGTGAAT

32881 AAACAAACCT ACTTTCTTCA TTAATCTTCA AAGGCGACTT CCCTATGAAT TTTCCTCTTT

32941 TCCTGTCCAC AATCAACGAG TTAGTGATAC TCCCTCTCTT TGGTACCCAA ACCTAGTGAA

33001 TTTTGGAAGT AAAAATCATG ACGCAAGTCC CGTTAAAAAC TATAATCTAA CCCAACCAAA

33061 ATGTAGTAAT TCCTATAGGT ATAGGACGGA CTTGTGCACA AGATCAAGTT TGAAAAACTT

33121 GTATCTTTGG TAAGTTTCAT TGTAAACATT GTCAACAACG TCGACGTCAA ATAGGCTTTC

33181 TTTTTCTTTG TATACCTTCC CGAAACCTAG CAAAGCACAT CACAAAAGAA GTAGTATTCT

33241 CGTTACGTAT TCTAATTCTA AGAAAAGGAA TATACCAACA CGAATAGAAA TTCCAAATCT

33301 TGTCTTGAGA TGGAAAATTT TTAAAATTAT CTGACATCTG ACTACTTGAT CTATTCTAAC

33361 TCTCTAAGAA AAAGGGAAAC CCCCCTTCTA TTCATACAAG AATAGAACTA GATAAAGATC

33421 CTAAAAAAGG ATTTCGATAT CTAATGAGAT AATTAAGATA ATAATTTGTA AAAAATTAGA

33481 TAAACTCAAA TTCAAATTTG AGTTCTATTT AGAATTCCTT TTTTTCTTTA GCTAAATTCC

33541 TAGGTAAGAT AAAAATAAGA TGAAAACAAG AGAATTTGTC TAAGAACAAT AGTTAGTTAT

33601 ACTAACTATA ACTAAATCAA ATTGAAATAC GTGTAATGGA AATAGGATCT CAGTCGATAA

33661 ATCTTGAAAG GACACATGCC CCGCAGTAAA CAAAGGAAAC TGGACGGTTC GATCAAAAAA

33721 AAATTCTTGC TTTTTGACTA AACGGTTATG AATGACTTGA CAATTTCAAG TCGAATCAAC

33781 TAATCCGGTA TATTTTTCAT GTTCATAGGA GTGCGTCTAT GTTTCTGCTT TACGAATATG

33841 ATATTTTTTG GTCATTTCTA ATAATATCAA GTCTTATTCC TATTTTGGCA TTTTTCATTT

33901 CCGGAGTTTT AGCCCCGATT AACAAGGGGC CAGAGAAACT TTCTAGTTAT GAATCGGGTA

33961 TAGAACCAAT GGGCAACGCT TGGTTACAAT TTAGAATCCG TTATTATATG TTTGCTCTAG

34021 TTTTTGTTGT TTTTGATGTT GAAACGGTTT TTCTTTATCC ATGGGCAATG AGTTTCGATG

34081 TATTAGGTGT ATCTGTAGTT ATCGAAGCTT TCATTTTCGT GCTTATCTTA ATTGTTGGTT

34141 TAGTTTATGC ATGGCGAAAG GGGGCATTGG AATGGTCTTA GCTCCTAAAT ATTCAGACAA

34201 TAAAAAGAAA AAAATAATAT TTAGACAGTT ATGAATTCTA TCGAGTTTCC CTTACTTGAT

34261 CGAACAACCC AAAATTCAGT TATTTCAACT ACATCAAACG ATCTTTCAAA TTGGTCAAGA

34321 CTCTCTAGTT TATGGCCGCT TCTCTATGGT ACCAGTTGTT GCTTCATTGA ATTTGCTTCA

34381 CTAATAGGCT CACGATTCGA CTTTGATCGT TATGGACTAG TCCCAAGATC AAGTCCTAGA

34441 CAAGCGGATC TAATTTTAAC AGCCGGAACA GTAACAATGA AAATGGCTCC CTCTTTAGTG

34501 AGATTATATG AGCAAATGCC TGAACCAAAA TATGTTATTG CTATGGGAGC GTGTACAATT

34561 ACAGGGGGGA TGTTCAGTAC TGATTCTTAT AGTACTGTTC GGGGAGTCGA TAAGCTAATT

34621 CCTGTGGATG TCTATTTGCC AGGCTGTCCA CCGAAACCGG AAGCAGTTAT AGATGCTATA

34681 ACAAAACTTC GTAAGAAAAT ATCTCGAGAA ATCTATGAAG ATAGAATTAA GTCTCAACGG

34741 GAGAATCGGT GTTTTACAAC CAATCACAAA TTTCAGGTTG GGCGTAGTAT TCATACTGGA

34801 AATTATGATC GAGAATTCCT TTATCAACCG CCATCTACTT CAGAGATCCC TCCCGAAACA

34861 TTTTTCAAAT ACAAAAGTTC AGTCTCTTCC CGCGAATTAG TGAATTAGAC AGGATTCCTT

34921 TGTACAGAAA AACAAGGAAT GGGTCAATCT GAAACAATTT CATCGTCAAT GCGAAATACT

34981 TATACAAATA ACAATGCGGG AGAGATAAAA AAGATGCAGG GTCCTTTGTC TGCTTGGTTA

35041 GGCAAGCATG GGCTAATTCA TAGATCTTTG GGCTTTGATT ACCAAGGAAT AGAGACTTTA

35101 CAAATAAAAC CCGAGGATTG GCATTCCATT GCTGTCATTT TATATATATA TGGTTACAAT

35161 TATCTACGCT CCCAATGTGC CTATGATGTA GCACCAGGCG GGCTGTTGGC TAGTGTGTAT

35221 CATCTTACGA GAATAGAGTA TGGCGTGGAT CAACCAGAGG AGGTATGCAT AAAAGTATTT

35281 GCCCCGAGGG GGAATCCTCG AATTCCATCT GTTTTCTGGG TTTGGAAAAG TGTGGATTTT

35341 CAAGAACGGG AATCTTATGA TATGTTGGGA ATCTCTTATG AAAATCATCC ACGTTTGAAA

35401 CGCATCTTAA TGCCTGAAAG TTGGATAGGA TGGCCTTTAC GTAAGGATTA TATTGCCCCC

35461 AATTTTTATG AAATACAAGA TGCTCATTGA AAAAAAAGAA ACTTACCTTC ACTTCTAAAA

35521 TTCCAGAGAT TGTTCTAGAT TAACCAGAGT CTCATTTGTT TTATGGATAG GATAACAACC

35581 AATTTAACCT TTCGAATATG TATATGTGTA TGAGGTATTA ACTTTTTGAA CGAGAGAGAA

35641 AAAAAGAAGA GAAAATAGAA TTTATTTTTG TTACATACTT TGTATAATAA ACTCTTTACC

35701 CATCTATTTT ATAGATGGGG TATTTCTCTA AAGACCGAAA GTATGTAATA TGATCTAGTG

35761 ACCTAGACTA TTAATGCATA TGTATGTCGA TTCATATCAA TTAATTTGTA GAAAAGAGAC

35821 TCATACAAAT TAATCATGTG CCAGGAACCA GATTTGAACT GGTGACACGA GGATTTTCAG

35881 TCCTCTGCTC TACCAGCTGA GCTATCCCGA CCATTCCAGA TACCGCATTC TTAGTTTACT

35941 AGATAACTTA GGTCTATGTC AATTCAAGGG GTATTACAAA GTCTTATCCA GGTGCTAGAA

36001 TTTCTTGGAT CTTCAAAAAA AGGAAGACTT TGTAAGTTTC AGTATGAATA ATGATATCGA

36061 CCGTGACTCG TTCAAATGGG GAGTCTTTGG TCAAAGATGT TCATTTGTAC ATGTATCTTA

36121 TATCACAAGA CTTGTCATAA GATAAAAATT TTTCACTCAG ATCCATTTGT AAAAGAGTAG

36181 GGTGAATGAG AAAGATAACG AATTTTTAAC TACTAACGAA AAAAAAGAGA AGATAAATAG

36241 TTAAGGAGTC AAATGGGCTT TTTTGGGGAT AGAGGGACTT GAACCCTCAC GATTTTTAAA

36301 GTCAACGGAT TTTCCTCTTA CTATAAATTT CATTGTTGCC GATATTGACA TGTAGAATGG

36361 GACTCTATCT TTATTCTCGT CCGATTAATT AGTTCTTCAA AAGAACTATC AGACTGTGGG

36421 GTGAATGCTT TGATCGATGC ATATTCCATT CTTTCTTCAA TATGGAATCG ATTCACAACA

36481 AATTTTTCCG TTTTTCATAT AAAGAATACA GATGCAGGTC GTTATTAATC ATTTGATAGA

36541 GTATTTCAGT ACGTATATGT ATATACGTAT ATCCTTCATC TTTTCGGAAG TTTCAATGGA

36601 AGGATTCCTC TACCAATGCA ACACAGTCAA CTCCATTTGT TAGAACAGCT TCCATTGAGT

36661 CTCTGCACCT ATCCTTTTTC ACCTTCTGAA CCTTTGTTTG TTTTTGGAAA AAGGATTTGG

36721 CTCAGGATTG CCCATTTTTT TACTTCCGGG GTTTCTCTGA CTTTGAAAGT TCTCACTTAG

36781 TAGGTTTCCA TACCAAGGCT CAATCCAATT AAGTCCGCAG CGTCTACCAA TTTCGCCATA

36841 TCCCCCTTTC TTTTTGTTTT GAGATTAGGA TCTCATTTTT GTTGTGATGT TGTTCTACTT

36901 TTTCTTTCAT TTCTTCATTT CTGCTGGAAC CATTGAATTC ATTAAATTAA ATAAGATTCC

36961 TGTTTCGAAA AAGTTGTTCT CTTCTTTGAG TTCTTGGTTA TCTTGTTTCA TCCTCTATAG

37021 GAAACCCACA TTTGGTCCAA TCAAAAACGA TTCTATCATT TCTGTATCCG CAATTCAATA

37081 TAGATGGATA TATACCACGT ATATATGTCT CTCTTCCGCT CGTTTTTCCC ATACAATTTG

37141 GAATACTTGA ACGGTCGATT CTTTTTTCTA AATAGAAAAA TGGAATAAAA TAGAGCAGTG

37201 TAATAAAAAT ACTTTCAAAT TTGATTTGAA AGCAAAAATC AAAATTATTG AATCTAAAAA

37261 TCTATCGAAT ATTAAATAAT TGAATTCTAT ATAATTGATA TAATTGAGAA AAAGAAATCA

37321 AAATTCTATA TCGCTAATTT AATCGTTAGA TTATCGAGTT ATATTCTCTA GTTATATTTT

37381 ATAGTAAGTA TGAGTATTTA ATATTTCCTT TCCTTTTTTA TTCTATTTTT CGATAGTCAT

37441 TATGACTAGC TACTAGCTAA TGAATCACTA AGAATGCAAA TCTTATAAGT ATATGTATAC

37501 TATACATATA TATTATTATA TATATTTTTA TTTTATAATA ATTTTTATTT TATTAATTTA

37561 TAGCTAAGAT TCCAATAATT TATTGAATTC CTATGCATCT ATAATTTCTC TTATGTGAGC

37621 CTGCTTAGCT CAGAGGTTAG AGCATCGCAT TTGTAATGCG ATGGTCATCG GTTCGATTCC

37681 GATAGCCGGC TTTTCTCTAT TTATTTTATT CGAGTTTTTA CATGATTGAG AATGTCCCTT

37741 TGAAATCCGA AAGAAAAGAA TATTGAAAAA ACTTCTCCCT CTTTTTCCAA AAAGTTGTCG

37801 ATTTTTTATG TTATAGGAAC TTCCAACTAT ATCACGAAAA TACTTTTTTT CTCTTTTTTC

37861 TATATCTATA TAGATTATAT CTATATAGAA TATATATATA GATATATAGA AGAATAGAGA

37921 AGGTCTGGAT ATTTGTCCAA TTCATCTCAT TATCCAAATA CATGTCTTCT TTTTTTCTTT

37981 ATAGTACAAG TACACTACTT CCGTAAACTT CGCTTCATTT ATCATTTAGT TCAGTTTTAG

38041 TTTAGCTTTA TTCTGTAAAA TGAAATTTCA TCAATAAGAA TAAAAAATAA GAAAATAAGG

38101 AGTCTTTATG TCGCGTTACC GGGGACCTCG TTTCAAAAAA ATACGCCGCC TGGGAGCTTT

38161 ACCGGGCCTA ACTAATAAAA GGCCTAGAGC CGGAAGTGAT CTTAGAAACC AATCGCGTTC

38221 CGGGAAAAAA TCTCAATATC GTATTCGTCT AGAAGAAAAA CAAAAGTTGC GTTTTCATTA

38281 TGGTCTTACA GAACGACAAT TACTTAAATA CGTTCGTATC GCCGGCAAAG CCAAGGGGTC

38341 AACAGGTCAG GTTTTACTCC AATTACTTGA AATGCGCTTG GATAACATCC TTTTTCGATT

38401 GGGTATGGCT CCGACTATTC CCGGAGCCCG TCAATTAGTT AACCATAGAC ATATTTTAGT

38461 CAATGGTCGT ATCGTAGATA TACCAAGTTA TCGTTGCAAA CCCCGAGATA TTATTACGGC

38521 GAGGGATGAA CAAAATTCTA GAGCTCTGAT TCAAAATTCT TTCGATTCAT CCTCTCATGA

38581 CGAATTGCCA AAACATTTGA CTCTTTACCC ATTCCAATAT AAAGGATTAG TCAATCAAAT

38641 AATAGATAGT AAATGGGTCG GTTTGAAAAT AAATGAATTG CTAGTCGTAG AATATTATTC

38701 GCGCCAGACC TAAAACTAAC GAAACACAAA GGTTCGGACA ATTTTGCTCC CTTTCGTCGA

38761 AATAAATTAA CCTAAGGTTA AGATAAATTA AGGGTTTGAT CCAGATTTTT ATCCCATTTA

38821 GGTATCATAG AACGAAAGGG AGATTTTCAC TCCTTGTCTA CTCTTTTCTT TTCAGTATTT

38881 TCCGTGCCCT AGCAAGGGAA TCTATATCAA AGAAGGGTCT AATTGTTGGA ATAATTTTAG

38941 CCAGTGCTAT TTGATCTATT CTGGTTAGTA GGATCCGTGA AGTAGCTGAA GGTACGGAAA

39001 GAGAGGGATT CGAACCCTCG GTAAACAAAA GCCTACATAG CAGTTCCAAT GCTACGCCTT

39061 CAACCACTCG GCCATCTCTC CTACATAATG ATTATGACCC AAAAACCGAG TGAATAGCGA

39121 GCCATTCCTA TTAGATTATT GGATAGGAAC GAATCAATTC TAACTATCAA AATAGATACA

39181 TTTTCATCCA AGTCAAAGAC AAATCTTTCT TTGAAGGCTC TGCGGATAAA TAGAAAATAC

39241 GACAACTGTT AATTCTATTC GTGTTTGAAA AAACAACGTT CTCCTCTTTT TCTGTTATTT

39301 TATTTTATCC CGGTACCGGA CTAAATTAGA TATTTTTTTG CACTGCGGTT AATGGATCTC

39361 TTTAGATCAT GATTCTATTT CGACTCTGAT TCTCAAATTC CTTTCCGGTC CAGTTTTAGA

39421 GTTCTCTCTC AACAAAAAAC CCCTAACCCG TAGATCTATT ACAAATATTC ATAAGAATTC

39481 ATAAGAATAA GAATTATATA TAACTATATA ATATAGTTGA TTGTATAGTG TATACCTCTT

39541 ATGCACACAA AACAGGCGGT TATTTTCATC ATAGAATGGT TATTCGATCC TTTTTCTTCT

39601 TTGGATGATA ATTCAATCAA AAAATTTCGA GTTATTCTAA TCTGTAACTT CAATGAAATT

39661 GGAATACTCT CTTTCGTTGG TTACTACTAC ATTAGGATGA AATCGAATCG TATTTCAATA

39721 TTTCCTTCTT TGTTTTTCTC GATTCCTACC AAAAAGAACC ATTTTAATAA TTTGTTTGAA

39781 TATAGGCCCA TATCTTTTTT TCTTAATGAA TCTTTTTTTA TGTTATTTCG TACTGTACAA

39841 TTCTTTTCTT TGTGGGATCG GTTTACCACA ACGAGAGGTA ATGAGAATAA TTATAGAATG

39901 GATTGCTACC TATGCCTAGA TCGCGGATAA ATGGAAATTT TATTGATAAG ACCTTTTCAA

39961 TTGTAGCCAA TATCTTATTA CGAATAATTC CGACAACTTC AGGAGAAAAA GAGGCATTTA

40021 CCTATTACCG AGATGGTGCG ATTTGATTCT TTTTTTATTG TTCTCAATCT TACGAGACAG

40081 ACACATTATT CGGGCTCGGG ATAGAAATAA AAATATTGAA AAAAAAATCT ACGCTTGTTG

40141 AAGGTAAAGT TTGGAACTAG GACAATTCCT TCTGTCGTGT ATCCTCGATT AATGCAACCT

40201 CAGATGCTAT GTTTTAATTG TCGATTCTAG TATTGAGCGA AAGGCTACAC CTATAGGTTC

40261 TGTATTTACA GGTCAATCCT ACTTCACCAG TACCAATAGG CCACATAGGG GAAGAAGCAC

40321 TACACCTAGG AATCAACAAC ACGAAAACTT TGTTATAAAT GAAGCCTTTC CTTATCAGGA

40381 TCGGAACTAA CAAGAATGGT CGGGACAACA AACATCCATC TCGTTTGTAT TTTGGATACC

40441 TGTATAACCA TCGAAGGCTG TTGAAGTGAC TAATTCCTGG AAATTAGGAG GCGTTTAGAA

40501 CAAATAAATT GTTGGAGTTA TCATTTCTAT CTAGTATCAA CACGTTTGAT GTTAAGAACA

40561 GATCTCTTGC AGTAAGGTTG GCTAGAGATT TCTTGTAAAA ACACTAGCCC TGCTCAGTTC

40621 ATAATGAGAA TAGTTTCATC TTTTTTGATT CCCTGTTTTA TTTTCATTAT GCACATAAAA

40681 GAGGAGCCGT ATGAGATGAA AATCTCATGT ACGGTTCTGG AACGGAGATT CTTTGAATGG

40741 AATGACGACC GTAACGGATG TCAGCCCAAT CCGAAGGAAA TTATGCGGAA GCTTTGCAGA

40801 ATTATTATGA AGCTATGCGA CTAGAAATTG ATCCCTATGA TCGAAGTTAT ATACTCTATA

40861 ACATAGGACT TATCCACACA AGTAATGGAG AACATACGAA AGCTCTGGAA TATTATTTTC

40921 GAGCGCTAGA ACGAAATCCG TTCTTACCAC AAGCTTTTAA TAATATGGCT GTGATCTGTC

40981 ATTACGTGCG ACTATCTCCA CTATAGAAAG AAAAAAGGAA AGAAGGATTA AATCCGCTAG

41041 TAAATACTAG AAACAAAAAG GGCTTTCTAC ATATGAATCG TCTAAAACAA CGATTTTTAT

41101 CAGCTGTAGC AAAGAAAGAA ACTTCATAGA AGTCGAAATA TGAAGTGAAG AAAGAGACTA

41161 GCTGTTTTAT TCTATGGATA AAGGATTTAA TTGATAGAGT AAGCATCGTA AAGATCAATT

41221 AGCGAGGTTT TGGGCCGATA CAATAATAAC TGCTTACTTA TGTCATGATG TGAGATAAGC

41281 GTTAGGAATC AACTTATGTA ATAGAGTTGA TCCACTAAGG TATTGAGCAG CGGTGTAGGA

41341 TCAGATCCCA AAGATAGTAA TTCTTTTTTT TATTATGAAT AAAAAGTTTT TTCAAAAATT

41401 CTATAGATAT ATATATCTAT TCTATATATC TATACTATAT AGAAAATAAT ATATATATAA

41461 AATAGAAATA TATTAAATAT AAATTCAATT TAGATATGAA ACCGAGATAG TTACCTTTCA

41521 GAAACTTCTA ACGATGATAG GGATAAATGC CTATGCTTTT TTTCTGAAGG TGGGAGAAAA

41581 GATAAAACTA AAATAAAACG GATTCTTAAT CCAAATTTAA GATTTCAGTT TAAAACTCAT

41641 GTCATTAACT TCTTTTCTTT TGGTTAACCC CCAAAACCAG ATAGATCTAT GAGAAATCTC

41701 GTTTTGTTAG ATATGGTAGA TTAAATGAAA TGGGATACCA AAAGAATTGA CTACTGAGCC

41761 GTATGAGGTA GGAAACTCTC AAGTACGGTT CTAAGGGAAG GAATTGACTC GCCTATTCCG

41821 ACCGGGGAGA ACAGGCCATT CGACAGGGAG ATTCTGAAAT TGCGGAGGCT TGGTTCGATC

41881 AAGCCGCTGA GTATTGGAAA CAAGCTATAG CGCTTACTCC TGGTAATTAT ATTGAAGCGC

41941 ATAATTGGTT GAAGATCACG AGGCGTTTCG AATAAAAGAA GACACCTTCT TTTTTTTCTA

42001 TTATTTTGAT TTAGTTCATT TCTTATTTAG GATTAGGATT TATATATATA TATATATATA

42061 TATATATATT TTTTATTTGT TATATAATTA ATAATTAATT GTTTGTTGGT CCCATCAGTC

42121 AAATCAGTCA AATAATAAAA AAGGATCATT CCTCTGGGAA GAACCAATCG AATAATTTCA

42181 TTATATCCCT TCTAAGCGTT CTATTTTGTC TGGTATTTTG TAGGGATAAA GGATACACAG

42241 ATAGAGTACA AAATAGACTT CGTTCTTGTC TTTCTTTTAA AATAAAAGAG ACCTTTGAAT

42301 CACCAAATAG ATATATCAGG ATATCTCTTA GTAATGGCTA ATAGCTGCTC GGGTGATTTT

42361 GAATTTGGAA TCGATAAAGA AATATGTTCT ATGTAAAACA TGAAGCAGCT CCATATAAGA

42421 ACTTCTAATT GAGATATGAA TAGACACTAG GTTACACAAA AAGGCCTTTT TTGCATTAAT

42481 CCAAAGCCCA GAAAGGGTTT TAGAAGATTA AAAAGGTCCG TTGAGCGCCT CATGGCTATG

42541 TCATAATAGA TCCGAACACT TGCCCAGGAT CGACTTCCAG ATCATAATTG CTCCAGTGAA

42601 TAACTAAAGA AAATAAATAG AGGGGAGATA GAAAAATAAA AGAGAAACAA ACTCATTGTA

42661 TAAATATAAA TATCTCTCAA AGATTCACTA ATTTTTGACT GTTGGCAGGT CTCTTTGTAT

42721 GTGTTGTCCG GAAAGAGGAG GACTCAATGA TTATTCGTTC GCCGGAACCA GAAGTAAAAA

42781 TTTTGGTAGA TAGGGATCAC ATAAAAACTT CTTTCGAGGA ATGGGCCAGA CCGGGTCATT

42841 TCTCAAGAAC ACTAGCTAAA GGCCCTGACA CCACCACTTG GATCTGGAAC CTACATGCTG

42901 ATGCTCACGA TTTCGATAGC CATACCAGTG ATTTGGAGGA GATCTCTCGA AAAGTCTTTA

42961 GTGCACATTT CGGCCAACTC TCCGTCATCT TTCTTTGGCT GAGTGGCATG TATTTCCACG

43021 GTGCTCGTTT TTCTAATTAT GAAGCATGGC TAAGTGATCC GACTCACATT GGGCCGAGTG

43081 CCCAGGTGGT TTGGCCAATA GTGGGCCAAG AAATATTGAA TGGTGATGTG GGTGGGGGCT

43141 TCCGAGGAAT ACAAATAACC TCTGGTTTTT TTCAGCTTTG GCGAGCATCT GGAATAACTA

43201 GTGAATTGCA ACTCTATTGT ACCGCAATTG GTGCATTGAT CTTTGCAGCG TTAATGCTTT

43261 TTGCCGGTTG GTTTCATTAT CATAAAGCTG CTCCAAAATT GGCTTGGTTT CAAGATGTAG

43321 AATCTATGTT GAATCACCAT TTAGCGGGAC TACTAGGACT GGGGTCTCTC TCTTGGGCGG

43381 GGCATCAAGT ACATGTATCT TTACCGATTA ACCAATTTCT AAACGCTGGA GTAGATCCTA

43441 AAGAGATACC ACTTCCTCAT GAATTTATCT TGAATCGGGA TCTTTTGGCT CAACTTTATC

43501 CCAGTTTTGC TGAGGGAGCA ACCCCCTTTT TCACCTTGAA TTGGTCAAAA TATGCAGACT

43561 TTCTTACTTT TCGTGGAGGA TTAGATCCAG TAACTGGGGG TCTGTGGCTG ACCGATATCG

43621 CACACCATCA TTTAGCTATT GCAATTCTTT TCCTGATAGC GGGTCATATG TATAAGACCA

43681 ACTGGGGCAT TGGTCACGGC CTAAAAGATA TTTTAGAAGC TCATAAAGGT CCATTTACAG

43741 GTCAGGGCCA TAAAGGCCTA TATGAGATTC TAACAACATC ATGGCATGCT CAATTATCTC

43801 TTAACCTAGC TATGTTAGGC TCTTTAACCA TTGTTGTAGC TCACCATATG TATTCCATGC

43861 CCCCTTATCC GTATCTAGCT ACTGACTATG GTACACAACT GTCGTTGTTC ACACATCACA

43921 TGTGGATTGG TGGATTTCTC ATAGTTGGCG CTGCTGCGCA TGCAGCCATT TTTATGGTAA

43981 GAGACTATGA TCCAACTACG CGATACAATG ATCTATTAGA TCGTGTACTT AGGCATCGCG

44041 ATGCAATCAT ATCACATCTC AACTGGGCAT GTATATTTCT AGGCTTTCAC AGTTTTGGTT

44101 TGTATATTCA TAATGATACC ATGAGCGCTT TGGGGCGTCC TCAAGATATG TTTTCAGATA

44161 CCGCTATCCA ATTACAACCT GTCTTTGCTC AATGGATACA AAACACCCAT GCTTTAGCAC

44221 CTGGTGCAAC GGCTCCCGGT GCAACAACAA GCACCAGTTT AACTTGGGGG GGCAGTGATT

44281 TAGTAGCAGT GGGCGGCAAA GTGGCTTTGT TACCTATTCC ATTAGGAACT GCAGATTTTT

44341 TGGTACATCA CATTCATGCA TTTACTATTC ATGTGACGGT ATTGATACTT CTGAAGGGTG

44401 TTCTATTTGC TCGTAGCTCC CGTTTGATAC CGGATAAAGC AAATCTTGGT TTTCGTTTTC

44461 CTTGTGATGG GCCTGGAAGA GGGGGGACGT GTCAAGTATC GGCTTGGGAT CATGTCTTCT

44521 TAGGACTATT CTGGATGTAC AATGCCATTT CGGTAGTTAT ATTCCATTTC AGTTGGAAAA

44581 TGCAGTCAGA TGTTTGGGGC AGTATAAGTG ATCAAGGGGT AGTAACTCAT ATCACGGGAG

44641 GAAACTTTGC GCAGAGTTCC ATTACTATTA ATGGGTGGCT CCGCGATTTC TTATGGGCAC

44701 AGGCATCCCA GGTAATTCAG TCTTATGGTT CTTCATTATC TGCATATGGA CTTTTCTTCC

44761 TAGGTGCTCA TTTTGTATGG GCCTTTAGTT TAATGTTTCT ATTCAGTGGA CGTGGTTATT

44821 GGCAAGAACT TATTGAATCT ATCGTTTGGG CTCATAATAA ATTAAAAGTT GCTCCTGCTA

44881 CTCAGCCGAG AGCCTTAAGC ATTGTACAAG GACGTGCTGT AGGAGTAACC CATTACCTTC

44941 TGGGTGGAAT TGCCACAACA TGGGCGTTCT TCTTAGCAAG AATTATTGCA GTAGGATAAT

45001 GGCTAGGAGG ATTTGAAAGG CATTATGGCA TTAAGATTTC CAAGGTTTAG CCAAGGCTTA

45061 GCTCAGGACC CCACTACTCG TCGTATTTGG TTTGGTATTG CTACCGCACA TGACTTTGAG

45121 AGTCATGATG ATATTACTGA GGAACGTCTT TATCAGAATA TTTTTGCTTC TCACTTCGGG

45181 CAATTAGCAA TAATTTTTCT GTGGACTTCC GGAAATCTGT TTCATGTAGC TTGGCAAGGA

45241 AATTTTGAGT CATGGGTACA GGACCCTTTA CATGTAAGAC CTATTGCTCA TGCAATTTGG

45301 GATCCCCATT TTGGTCAACC AGCTGTAGAA GCTTTTACTC GAGGGGGTGC TCTTGGCCCA

45361 GTGAATATTG CTTATTCTGG TGTTTATCAG TGGTGGTATA CAATCGGTTT ACGCACTAAT

45421 GAGGATCTTT ATACTGGAGC TCTTTTTCTA TTATTTATTT CTGCTATATC TTTAATAGCA

45481 GGTTGGTTAC ACCTACAACC GAAATGGAAA CCGAGTGTTT CGTGGTTCAA AAATGCCGAA

45541 TCGCGTCTCA ATCACCATTT GTCAGGACTC TTCGGCGTAA GTTCCTTGGC TTGGACAGGG

45601 CATTTAGTAC ATGTGGCTAT TCCTGCATCC AGGGGGGAGT ACGTTCGATG GAATAATTTC

45661 TTAGATGTAT TACCACACCC CCAGGGGTTA GGCCCACTTT TTACAGGGCA GTGGAATCTT

45721 TATGCTCAAA ACCCCGACTC AAGTAGTCAT TTATTTGGTA CCTCCCAAGG AGCAGGAACT

45781 GCCATTCTAA CCCTTCTCGG GGGATTCCAT CCACAAACGC AAAGTTTATG GCTGACTGAT

45841 ATGGCTCATC ATCATTTAGC TATTGCATTT ATTTTTCTCG TTGCCGGTCA TATGTATAGA

45901 ACTAACTTCG GTATTGGGCA CAGTATGAAA GATCTTTTAG ATGCACATAT TCCCCCGGGG

45961 GGACGATTGG GACGTGGGCA TAAGGGTCTT TATGACACAA TCAATAATTC CCTTCATTTT

46021 CAATTAGGTC TTGCTCTAGC CTCTTTAGGG GTTATTACTT CCTTGGTAGC TCAACACATG

46081 TACTCTTTAC CCGCTTATGC ATTCATAGCA CAAGACTTTA CTACTCAAGC TGCGTTATAT

46141 ACTCATCACC AATACATCGC AGGATTCATC ATGACAGGAG CTTTTGCTCA TGGAGCTATA

46201 TTTTTCATTA GAGATTATAG TCCGGAACAG AATGAGGATA ATGTGTTGGC AAGAATGTTA

46261 GACCACAAGG AAGCTATCAT ATCTCATTTA AGTTGGGCCA GCCTCTTTCT GGGGTTCCAT

46321 ACCTTGGGAC TTTATGTTCA TAATGATGTC ATGCTTGCTT TTGGTACTCC GGAGAAACAA

46381 ATCTTGATCG AACCCATATT TGCTCAATGG ATACAATCTG CTCATGGTAA AACTTCATAT

46441 GGTTTCGATG TACTTTTATC TTCAACGAAT GGCCCGGCAT TCAATGCGGG TCGAAGCATA

46501 TGGTTGCCGG GTTGGTTAAA TGCTGTTAAT GAGACTAGTA ATTCACTATT CTTAACAATA

46561 GGTCCGGGGG ACTTTTTAGT TCATCATGCT ATTGCTCTGG GTTTGCATAC AACTACATTG

46621 ATCTTAGTAA AGGGTGCTTT AGATGCACGT GGGTCCAAGT TAATGCCAGA TAAAAAGGAT

46681 TTCGGTTATA GTTTTCCGTG CGACGGCCCG GGGCGAGGCG GTACTTGTGA TATTTCGGCT

46741 TGGGACGCAT TTTATTTGGC AGTTTTTTGG ATGTTAAATA CTATCGGATG GGTTACTTTT

46801 TATTGGCATT GGAAACATAT CACATTATGG CAGGGTAACG TTTCACAGTT TAATGAATCT

46861 TCCACTTATT TGATGGGCTG GTTAAGAGAT TATTTATGGT TAAACTCTTC ACAACTTATC

46921 AATGGATATA ACCCATTTGG TATGAATAGT TTATCGGTCT GGGCGTGGAT GTTCTTATTT

46981 GGACATCTTG TTTGGGCTAC TGGATTTATG TTCTTAATTT CCTGGCGTGG CTATTGGCAA

47041 GAATTGATTG AAACTTTAGC CTGGGCTCAT GAACGCACAC CTTTGGCCAA TTTGATTCGT

47101 TGGAGAGATA AACCGGTGGC TCTTTCCATT GTGCAAGCAC GATTGGTTGG ATTAGCCCAC

47161 TTTTCTGTAG GTTATATATT CACTTATGCG GCTTTCTTGA TTGCATCGAC ATCAGGCAAA

47221 TTCGGTTAAT TTTTTTGAAT GTCTTGTATT TGCGATAATC TCATTTCTTT AGACGGCGGA

47281 GAGGTCCGCC TTCTTATATT TCTACATCTA GGATTCGACT TGTATCATGG ATACTAATAG

47341 GAACTGAAGC ATTATGGCAA GGAAAGGTTT GATTCAGAGG GAGAAGAAGA GGCAAAAATT

47401 GGAACAGAAA TATCATTTGA TTCGTCGATC CTCAAAAAAA GAAATAAGCA AAGTTCTGTC

47461 GTTAAGCGAC AAATGGGAAA TTTATGGAAA GTTACAATCT CCACCGCGGA ATAGTGCACC

47521 TACACGCCTT CATCGACGTT GTTTTGCGAC CGGAAGGCCG AGAGCTAACT ATCGAGACTT

47581 TGGACTATCT GGACACATAC TTCGTGAAAT GGTTCACGCA TGTTTATTGC CAGGGGCGAC

47641 AAGATCGAGT TGGTAAGGAT TAAAATATCC TCTCATTTCA TTTCTATGAT CGATGATCAT

47701 AGAAAGCCCC TTTACCATTC TGTATAAATG GGCTATTCTA TTTGTACAGG TATGGTAGAG

47761 GGGCGCATTC AATTCTTGTT TGTCTATAAG TTTTCAATTC TTCTCGTCAG CGCGGGGTAG

47821 AGCAGTTTGG TAGCTCGCAA GGCTCATAAC CTTGAGGTCA CGGGTTCAAA TCCTGTCTCC

47881 GCAATATCTT TTTTTGTCAA CCTTTTTTAG GTTTGACTCT GTTAACTAGT AATTAATTAA

47941 CACCTTTTTG GGGGATGGGA GGAAAAGAAG AAGGGGTGGA TAGAATCACT ACACTATTAC

48001 GGCCAACTCT AGCGAATCCT TTATTTCTCT TTTTATCAAC AATAAAAAAT ATGACGATGA

48061 TATGAAATAA ATGACTCCAT CTTGGGCGGA TAGCGGGAAT CGAACCCGCG TCTTCTCCTT

48121 GGCAAAGAGA AATTTTACCA TTCAACCATA TCCGCATTTT TTTCGTTCTT GATACACAAC

48181 ATGCGCACAC ATATATCATA TATACGCCTG GATCGTATTT GTGCAGTGTC AGGCCACAGA

48241 CTCTTTTCAG GGTAAGTTCA ATTACTTTCT TAATTTTAAA TTTTTTTCAT TTAAATTCAA

48301 GAACAAGAAG TGTGACCCCC CCTCTAATTT TTTGTTTTAT TTATTTGCTT TTTATTTTGG

48361 GGACTTCTAT TAAATTTGAC TGTGTCTCAC AACCGAGAAT AATTCGGGGG GGTCATTTCG

48421 GTTTTGGATC TGGAACGAAT AGGTTCAAGA GATGAGAGAA TTAAGGATAC CCACCAGAAA

48481 GACTAATCCA ATCCATAATG ATGTACCAGA AAATAAAACA TTTTTGTTAC TTGACCAACC

48541 ATCAGGAGAA GCAAATACAA CAGGTACACC AATTAATAAG ATTGATGAAG TAGCAATTAA

48601 TGCAAAAACA GCCAATTGGA AAGCAAGAGT CATGCTTTTA ATCCTCCAAG CTACCAACAA

48661 ATGAACTATA CCATTTAATC CCTCAAAAAT TGTACTAAAA TACAGCATTA GAGGGATTAT

48721 ACCATAAATC TATTGAGTTG CTATGACTTA TTACTACCCC TTATTGTTAC CATTTTTTTC

48781 GTACACGTAG TCGGGGGAGA ATGGAATTTT TTTCACAAAC AAAAGGGCAT GCTGGATCAT

48841 ACTTCCTATA TATATATATA TATACGGATA GATAGATTGA CCAGTGGATT CACACCCGAG

48901 ATCTTTCTAC AGATAGTAAT GTTCTATCCA CTCATATAGC CATGTTCTAT TCGGAGGAAT

48961 AAAATAAAAC TGTCTTTCGG AGAGATGGCT GAGTGGTTGA TAGCTCCGGT CTTGAAAACC

49021 GGCATAGTCT TGAACAAAGA ACTATCGAGG GTTCGAATCC CTCTCTCTCC TTTTTGCTCG

49081 TTGAATAGAC TTTTTCTTTA TTTGTTTTGC CCGGACAGGA TCATAACGAA AGGGCAGTGG

49141 CTCGGCTATC CCACCTAGCC AAGCCAGAAA AAAAGATTCT ATTTATATCT ATATATTTAA

49201 TTTATATATA TAATATAATA ATAAAGAATA AAGAAATGAG TTGAAAAAAT TACTTTTTTC

49261 AGTATGACCT GATTCCAATA TGGATGATAG AATCAAAGTC ATTTATACTT CAATCAAGTA

49321 TTGGATCTCC TATCTCAATT AATTAATTAA GAGGGGTCAT GGAAAGAACA GGTTCAAAAT

49381 CACGATCAAT TCCTTTTTCA AACCCTGCTG CAGCTGCACG GGCCCTTCCC GCGTGCCACA

49441 AATGACCTAC GAACAAGAAG AATCCTAGAA CAAAATGAGA AGTAGCTAAC CAACTTCTAG

49501 GAGAGACATA ATTGACTGCA TTGATCTCGG TAGCGACACC ACCCACGGAA TTTAAAGAAC

49561 CTAAAGGAGC ATGAGTCATA TATTCTGCAG AGCGCCGTTC TTGCCAAGGT TGTATGTCTT

49621 TTTTCAGCCT ACTTAAGTCC AACCCATTTG GACCCCTTAG AGGTTCTAAC CAAGGGGCAC

49681 GCAGATCCCA AAAACGCATA GTTTCTCCGC CAAAAATGAC TTCTCCAGTG GGTGAACGCA

49741 TTAAATATTT ACCTAAACCG GTAGGTCCTT GAGCGGATCC CACGTTAGCC CCAAGACGTT

49801 GGTCTCTAAC TAAAAAAGTA AATGCTTGAG CTTGGGAAGC TTCTGGTCCA GTGGGTCCGT

49861 AAAACTCGCT AGGATAGGCG GTATTATTGA ACCAGACAAA ACAACAAGCA ATGAAACCAA

49921 AGACGGATAA AGCCGCTAAA CTATAAGATA AGTAAGCCTC TCCCGACCAT ACAAGTGCGC

49981 GTCGAGCCCA TGCGAAGGGT TTGGTTAAGA TATGCCAGAT TCCACCAAGT ATACAAATGG

50041 AACCTAACCA TACATGTCCT CCGATTATAT CTTCCAAATC GTCTACACTA ACAATCCACC

50101 CTTCCCCTCC AAAGGGGGAT TTTAGTAAAT AACCAAATAT AATACTTGGG CTGAGGGTCA

50161 AGTTGGTAAT TTTTCTTACA TCTCCCCCTC CCGGAGCCCA GGTATCATAT ACACCACCAA

50221 AATAAAGAGC CTTGAATACT AGAAGAAAAG CACCTACACC TAACAAGATT AAGTGAATAC

50281 CTAAAATTGT GGTCATTTTA TTTCTATCTT TCCATACATA ACCGAAGAAT GGAAAAGATT

50341 CTTCAAGCGT CTCAGGTCCT AGAAGTGCGT GATAAATACC ACCAAAGCCC AATACTGCAG

50401 AGGAAATTAA ATGAAGTACT CCAGATACAA AATATGGAAA AGTGTCTATA ACTTCCCCAC

50461 CAGGACCTAC CCCCCAACCT AGAGTAGCTA GGTGGGGAAG TAAAATTAAT CCCTGTTCAT

50521 ACATAGGCTT CTCTGGTACG AAATGAGCCA CTTCAAATAG ATTCATTCCT CCGGCCCAGA

50581 ATACGATTAA TCCGGCATGG GCTACATGAG CCCCTAGTAG TTTACCGGAT AAATTGATAA

50641 GTCGGGCATT CCCGGCCCAC CAAGCGAAGC CGGTGGTTTC TTGGTCACGA CCAGATAAAG

50701 CTAAAGTTCC ATTAAAGAGC GTTTCCACGG GGTAGAACCT CCTCAGGGAA TATAAGGTTT

50761 TCATGAGGCT GATCTTGAGC CGCCATCCAA GCACGAATAC CTTCGTTTAA GAGAATATTT

50821 TTGGTGTAGA AAGTCTCAAA TTCAGGATCT TCCGCCGCGC GAATTTCCTG AGAAACGAAA

50881 TCATAGGCAC GTAGGTTCAG GGCCAGACCA ACTACTCCAA GAGCACTCAT CCATAAACCG

50941 GTTACTGGTA CAAATAACAT AAAGAAATGT AACCAACGTT TATTGGAAAA AGCAACCCCA

51001 AAAATTTGGG ACCAAAAACG GTTCGCGGTA ACCATTGAAT AAGTTTCTTC AGCTTGAGTT

51061 GGGTTAAAAG CACGGAATGT ATTTGCACCA TCACCATCTT CAAATAAAGT ATTTTCTACA

51121 GTAGCACCAT GAATAGCGCA TAGCAAAGCA GCGCCCAATA CCCCGGCAAC TCCCATCATA

51181 TGAAATGGGT TTAACGTCCA ATTATGAAAT CCTTGAAAAA AGAGGATGAA GCGAAATATA

51241 GCTGCTACAC CAAAACTGGG CGCGAAGAAC CAACCAGATT GCCCTAGTGG ATAAATCAAG

51301 AATACAGAAA CAAAAACAGC AATTGGACCA GAGAACGCGA TTGCATTATA AGGGCGCAAT

51361 TGAACAGATC GAGCAAGTTC GAATTGACGT AACATGAAAC CTATTAGTCC GAAAGCACCA

51421 TGGAGAGCAA CAAAAGTCCA CAGACCGCCT AATTGACACC AACGAGTAAA ATCCCCTTGT

51481 GCTTCAGGGC CCCATAGTAA CAACAAAGAA TGTGCTAAAC TATTAGCAGG AGTAGAAACT

51541 GCGGCAGTTA AGAAATTGCA GCCTTCCAAA TAGGAACTGG CCAATCCATG GGTATACCAT

51601 GAAGTTACAA AGGTTGTACC TGTGAACCAA CCTCCTAAAG CGAAATAGGC ACAAGGAAAG

51661 AGCAATAGAC CGGACCATCC TACAAAAACG AAACGGTCCC TCCGTAACCA GTCATCCATA

51721 AAATCAAATA AATCCTTTTC GTCTTTGGTA AATTTACCAA GGGCTATAGT CATAGTAATC

51781 CTCCTATTCA ACTACTTCGA CCATTTCCGA ACACCTCATA GCATTTTCGG GGTGTCCACC

51841 GATTCGATCA TTTCTATATG ATTTCTCGTG CACTCCCCCT TTCAATGGGT TTCGAAGATA

51901 AAAATCCTTT CTTGCTTTAT TGATTGTCCC ATAAATTCAC CTAGGTAAAT CCACGAACTT

51961 CATTTTATTA TTTCTTTTAT TAGTTTTTTT ATTCTTATAG CTGTTAGAAT TATAATAGGA

52021 GACTGGAAAT GAAAGTTTTT TTCACGCATT CATTCTCCAC GACGCTGTCG GAACAAAAAT

52081 ATCGGATGCA GTAATATGGA AATGTTTGGC ACATGAAGCT TCGATCTGAT AGATATAGAT

52141 ATACATTTAA ATAGATCAAT CAATCTTATT ATAAATCTTA TATATATATA AATGAACCTT

52201 GATAAAGATC AAAGTCTGTA ATCAAAGTAA TCAAAGAAAG GCTCTTATGG TGTGACTTGG

52261 AATAAAATTT TTCTATGGAG GAACGAAATA ACCATTTCTT CCTATGGAAG ATCTAGGAAC

52321 AATAAGATTG AATTGGAAAT ATCGACAAAT TCTTGCGGAG TCCAAAGAAA TAAAAAGGGG

52381 TTAACTTGTT CCAATTTTGT CTCTATCGAA TTTGAATGAA AGTAGATTCT CTTTCCATTC

52441 ATTTAAATAT CAGAATAGCG GATATCGGCA TGATTCAATG GGTCAGGTCC ACTTATTTTT

52501 TCTTTTGTTG ATTTCTAATC TTTACAATGC ATTTGATTGG CAAAATGGAA AATAGGAGGG

52561 TTTGGTGATT CCGGAGAGAC GACTTATCAT CCTGCTAAAT TAGAATTAGA GTATAGTAGT

52621 TTAGTGAATA GTTAGAAAGT TGAACATTTG TTCTATTTAA AATCTCTCTA TTCTCGCTTC

52681 AAATATGAAT ACTACGGCTC TAGTTTTATG AAAAAAGCCA AAGCCCTTTA TCGGATTTGA

52741 ACCGATGACT TACGCCTTAC CATGGCGTTA CTCTACCGTT GAGTTAAAAG GGCTCGTTTG

52801 ATTCAATTCC TGAATCAGGA ACTCTATGGA TAAAATCACT CTATGGATAA AATATATCCA

52861 TGTACATCTA TTATAGACTT AAGTATATAT ACATAAGTCC ATGCACAGTA ACTTCGTAAT

52921 GGCTAGTGGC TTATTTTTGA ATAATCAAAT GAATTGATTT ACCTATCAAG TCTAGCAATG

52981 AATTGTTTCA AAACCGATCC TAATTGCTTT TATTTTATTG AACGAAATGG TACTCATCAG

53041 AACGAAATTC ATTTGATTGA TACATGCACA CCTCGCAATT CGACATAGAT TCAAGATATT

53101 TCATCGAATC ATCATGTGAT GAATAGCTTC TACTTGTCCC TGAACTAAAT TATTATAGAA

53161 ATATTTAGTT TTTAGATAAC TTCTTGATCC CTCTTTTTTT ATGTTTGTTA ATTTCCGTAG

53221 TCCGAGTCCC CTTTTATTTT CTGGCGGACG AACCACTCCC TAAAAGAATC CTATCTTCCG

53281 TATTATTTCT ATCTATTTTT TTTATGAATA TTCTTTTTAT TTAGTCTATT TCTATTAATT

53341 TTTCTTAATT ATTAATAATA GAAAATAAGA AGTATTGTTA TTATACTTTC TAATAAATTA

53401 TTCTTATTTA TTGACAATTA GAATAACCTA TTCGTACAAT GGGCATCATA TGATTCTGAT

53461 GGATGTTGGG CAAATAAGCC CCCATCGTCT AGTGGTTTAG GACATCTCTC TTTCAAGGAG

53521 GCAACGGGGA TTCGACCTCC CCTGGGGGTA GGATACTACA AAAGGAGGTT GATCGTGGAT

53581 TACCAATAAA CCTCAAACGG GTTTTCCTGG GTCGATGCCC GAGTGGTTAA TGGGGACGGA

53641 CTGTAAATTC GTTGGCAATA TGTCTACGCT GGTTCAAATC CAGCTCGGCC CAACAATTCG

53701 CCAATATACC ATGAGATGAT ATAACCCCCC TGTCCTTCAG AAATACCCGA TATGGAGAAA

53761 TAAAAAAGAA TCAAAATTTC TGCTAGATCC CTTATTTCCC TGGGATTGTA GTTCAATTGG

53821 TCAGAGCACC GCCCTGTCAA GGCGGAAGCT GCGGGTTCGA GCCCCGTCAG TCCCGACAGA

53881 TTCAATAAGT ACATCAAATC ATCTTTCCTT TTTCTGTCAA CGGGGGAACA AGAAGAAAAA

53941 TTTCATTCCC CGGGGGGTTC TTTTTTCTTT CCTTTTTCGT TTCGCCCTTC TCATCAAACA

54001 AATAGATAGG ATAATTTTCG TTACTTCAAC TAGTACTCAT TGGTAGTAGA AAGACATATG

54061 TAGATTAGTA CAATTGATGG AGCAATAGAG TCAGTATTCC AAGAGATACT GAGTCTGCTT

54121 TTTCGATGGA ATAGGGGACT ATATGTTTCT CAAGCGCACA TACAAAAAAA TGGAATTCCT

54181 TTCGTGTACA ATACAAAACA AAATGAATTT AACAGAATTC CCCCGATAGA ATACTTTTCC

54241 GGCTTAAAAA ATAACCCCTC CCCGTTTTGG TTTTGTTTGT GTAACTTCAA TTACTAATGG

54301 GAAATTGAAA ATTTGATTCA AATTTTTGAT TGGGCTGGGA ATCCTATTTT GGATTCAGTA

54361 TGGAAAAAAT GTCTTCCTAT GTTATACTAT TTAATTCGCG ACGACGAATT GATTTGGTAG

54421 CTCAGATATT ATATTGTTAT TCATGATATT GATCTGATTC AAGATCATTG AGAGGTAATT

54481 CATTCATTGA ATTTACAGGC GAAAGGTTTA TCATCTCTAT GGGATTAAAT CCCGAGTTAT

54541 TGTGAAGTAA AAAAAAGATG AGATTATGGA AGTAAATATT CTTGCATTTA TTGCTACTGC

54601 GCTGTTCATT CTAGTTCCTA CTGCTTTTCT GCTTATCATT TATGTAAAAA CAGAAAGCCA

54661 AAATCAAAAT AAAAAAGATT AATTAATTTG AATGAAACTT GACTTCTGAG TTCTTATCAA

54721 CGATTGAAGA AAAAAGGAAA ATGATTCCAA GATTCCAATT TCGTGTCTTA AAATGAAATG

54781 AGCGGATTAT AATCGACAGT ATTCTATCAG ATCCGATACT ATATATAGTA TAGTAAGAAA

54841 GAAATCTAGA TTTCTTTCTT ACTATACTAT CTATTAGTGT TATTGTAGTG TATAAAGTTC

54901 TACTTCTTAA TTAAAGAATT GTCCTACTTT AAAGTTCTAC TTTATAGATT TCGTATTCTT

54961 TTCAATTTTA GTCTCGTGAT CTATCGAAAG AATCAAATCA ACGAAGGAAA AAGCATTATG

55021 AGCATATATT AACATTAACA TATAGTAAAG AGTAGTAATC TTTTTTCTAG CATTTCAATT

55081 CAAAGAAAAA ATGGATTGAT CCATTGACAG GAGTTCTATG GGCACTTCTT CGGATTTTGA

55141 AAGGGAATAA ATAATAAACC TCGCCGATTT TTAAGGCTTG AACCCTGATA TTAAGATATG

55201 AAATGAGTCG ATAAAGTAGA AATTCCATTT TTCGCAAAAA ATAAAAGAAT CGGTAAGTGC

55261 GCGCAATATG TGACTCGCCC AAGGTAAGAT CTTATTATGC ATAGCATATC ATCATCATTA

55321 GACAACGACT ACGTCTATAT TTTTGCTCTA TAGAAAGCGC TTATACACTT AAAAAATTCC

55381 TTTTTGTTGA GCAGTAAAAG TAAAGAGGGA AAAGAGAGAA ACAAAAAAAG GGAGGTCAGG

55441 ACTTCTTCAG TATCTATCTC CAATTCTGGT AAGAGCCCGT TCATTGAAAT AGAAAATTTA

55501 GGAAAAATTG GGTTGGAATA AAATTCCAAT AATATGTATC CCTTTCCTTT CGAGATACAA

55561 ACATGGGAAT TATTGAAATA TATCTTTAAT TGAAATAATT ATTGAAATAA TTTTAGTCAT

55621 ATAATACATT ACTCCACTAG AATCCAATAG AATTCCGAAC TGAAGATCTT TTTTGAATAA

55681 TTCAAAATCC GTTGCAGAAG GATCTCTAAA ACAATTGATA CGGTTTGATT CCTCAATCAA

55741 TTAGTAGTAC CTTTAGAGTC CACTTCTTCC CCATACGACG AGTGAAAGGG AAAATGTAAA

55801 GACTACCATT AAAGCAGCCC AAGCGAGACT TACTATATCC ATGTGAATTA TGTCCCCTAT

55861 CTTATATGAA GGAATTATTC CATTATTGCT CATTAATAAT AGTGGAATCA ACGGCGCAGA

55921 GTCAAAAGGG GGCTCTGACC GAACACTGTT GATGAACCAA TCTAATAAAT CCACTTCTAC

55981 TAAAGTCCTA TAGGACTTAT AATTGGGAAA GACTAAACAA AAGGCAATGA CATCTCAAGG

56041 AAATGTTTCT AATGGAACAT ATATAGGAAT AAAAAGGCTT ATTCTTTTTG CCCTTTTTTA

56101 TATCTTTTAT ATCTATACTA ATACTATATC TATAGATTTT TCGTGTTTCA AATCTGAATT

56161 TTCATGGAAT CAAAAATAAA CAAAGAGTAG CAATAAAGGA AAGGGCCTAT ATATAGATAT

56221 CAGTCTAATA TGGAATGTGA ACACTCAGAG ATTCTTGTGA GTCTGTATAT ATAAAGTTAT

56281 TTATTTCGAT TTTCTATATT TTCTTTTTTT TAAAGGATCT TCCGGTCTTT ATACAACTAA

56341 ACTACTAATT TAATTAGATT TCGTATCCGT CTATTCAACG GAATTCAAGA CCCAGCCAGT

56401 AGACACTACA TTAGTAGTAT AATAGAAAGA AATCGGGAAG TCGTGATAGC CCTTCGACCA

56461 TTAGAATCTT TAATCCTAGA TGCAAAGATT TACAATCTTT TAGAAAACCT CCAGACCGAA

56521 TGCACAATCC GTTTCAGTTG CCGGGGAAAA ATTCGTTGAG TTATATATCA ACAGAGCAGA

56581 ATAAGAGATT TCGAGTCTTT TATTGATCAG GCGACACCCG GATTTGAACT GGGGAAAAAG

56641 GATTTGCAGT CCCCCGCCTT ACCACTCGGC CATATCGCCA AAACGATACA AAAAAATAGA

56701 CGAAAATTTT CCCACTTAGG GTTGGATTAC GGAACTTCTT TTTTTGTACT TGTATCTTGA

56761 ATCCTGTTTT TCTTAATCTG AAAACCCACA TTTGACTTTT CAATAGAAAG GTCAGGGGAA

56821 GTTTTTAGGC AGTGTGGGTT TTCAGTCACA AAAGAAAAAG TTGATGAAAA ATTGGAACCA

56881 TTAACTATTG ACTCCTGGCT GCGAATTCAT GAATAAGTCT TTGATTTGAA TATCAAATAG

56941 TGTTTTCCTA ATGACACAAG AAAATCAAAA TTGATACATA ACTAATCGGT GTTTATTCAG

57001 TAATTTTTTC TTTTCAATAA AAAATCCTTC TCAATTTCAA TTATAACAAT ATATAGGAGT

57061 ATATGTAAAC CCCAGAACAT AAATTGTTAC ACAGATATCT AATAGGTTAT CTTATTTCTA

57121 TATGTTGGCT ATTATATATT TCTATTTTTA TTAGATATAT TTAGATTCTA TTTTTATTAG

57181 ATATATTTAG ATTATAGGGA ATTCTCAGTA ATTTTTCGTG TTTCAAATCT GAATTTTCAT

57241 GGAATCAAAA ATAAACAAAG AGTAGCAATA AAGGAAAGGG CCTATATATA GATATATCAG

57301 TCTATATTTG CCCGTGCCTA TTTTTTTATT TATTTAATAA ATACAACCCC TCTTATACAC

57361 TTCACGATCC CCCGCGTATG CTAAAAATTC TACTAAAAAA AAATAGGTAA AGTAAAAATG

57421 CTTCTCTTCT CTGTGAATCC TTTTTTTGAA CAATGGAATA TCTAAAAGTG ATTTAGTACT

57481 AAAAAATCCA CTCTATATTG TTTCTGAGTT TTATTTATGT TTTTTTTATC TTAGCGAAAA

57541 GATCAACTGC TGAATCCATT GAGTTTTTCT GGTATTCAAG CAGATTGGAC TATGTCTATG

57601 TAATATCATA ATAATGGTAG AAATTGAATC TCTTTTGATA TTCTCTACAA AATTCCATAA

57661 TATAGTAAGC CCAAGTGGCA GAATTCTGTT TCTAGAATTG TTTTATCAAT TCCTTTCCAT

57721 TTGTATCTGT TTCTCGAATT CGAATTTGAG TAGAAAATTT TCTTTATTCC TCCGTTCATA

57781 CATATTTCAT ACATTCTATT CCAGATATGG AGTTGTGTTA AATTTCATGT GATTCAGTAA

57841 ACAGAATAAA AATTCCATCA TTGCTAGATC ATCTATATGA TTTCATGAAG AATGAGCGAA

57901 GAATGGGATT TTTTGATAAA TGGGAAATGA AAAATGCTCC GAGACGGAAA TGAGGGAATG

57961 TCTACAATAC CTGGGTTTAC TCAGATACAA TTTGAAGGAT TTTGTAGGTT CATTGATCAG

58021 GGCTTGAGGG AAGAGCTTTA TAAGTTTCCA AAAATTGAAG ATACCGATCA AGAAATTGAA

58081 TTTCAATTAT TTGTGGAAAC ATATCAATTA GTAGAACCAT TAATAAAAGA AAGAGATGCT

58141 GCGTATGAAT CACTCACCTA TTCTTCTGAA TTATATGTAT CCGCGGGACT CATTTGGAAA

58201 ACCGGTAGGG ATATGCAAGA ACAAACTATT TTTCTTGGAA ACATTCCTCT AATGAATTCT

58261 CTGGGAACTT CTATAGTCAA TGGAATATAT AGAATTGTGA TCAATCAAAT ATTGCAAAGC

58321 CCCGGTATTT ATTACCGGTC AGAATTGGAC CATAACGGAA TTTCGGTGTA TACCGGCACC

58381 ATAATATCGG ATTGGGGAGG AAGATCAGAA TTAGAGATTG ATAGAAAAGC AAGGATATGG

58441 GCTCGCGTGA GTAGGAAACA AAAAATATCT ATTCTAGTTC TATCATCAGC TATGGGTTCG

58501 AATCTAAGAG AAATTCTAGA AAATGTCTGC TATCCTGAAA TATTTTTGTC TTTTCTGACT

58561 GATAAGGAAA AAAACAAAAT CGGGTCAAAA GAAAATGCCA TTTTGGAGTT TTACCAACAA

58621 TTTGCTTGTG TAGGCGGGGA TCCGGTATTT TCTGAATCCT TATGTAAGGA ATTACAAAAG

58681 AAATTCTTTC AACAAAGATG TGAATTAGGA AAGATTGGTC GACGAAATAT GAATCGGCGA

58741 CTGAACCTTG ATATATCCCA GACCAATACA TTTTTATTAC CACGAGATAT ATTGGCAGCC

58801 GCGGATCGTT TGATTGGAAT GAAATTTGGA ATGGGTACAC TCGACGATAT GAATCATTTG

58861 AAAAATAAAC GTATTCGTTC TGTAGCAGAT CTTATACAAG ATCAATTCGG ATTGGCTCTG

58921 GTTCGTTTAG AAAATGTGAT TCGAGGAACT ATATGTGGAG CACTTAGGCA TAAATTTATA

58981 CCGGCCCCTC AGAATTTGGT AACTTCAACT CCATTAACAA CTACTTATGA ATCCTTTTTC

59041 GGTTTACACC CATTATCTCA AGTTTTGGAT CGAACTAATC CATTGACACA AATAGTTCAT

59101 GGGAGAAAAT TGAGTTATTT GGGCCCTGGA GGAGTGACAG GGCGAACTGC TAGTTTTCGA

59161 ATACGAGATA TCCATCCTAG TCACTATGGG CGTATTTGCC CAATTGACAC GTCTGAAGGA

59221 ATAAATGTCG GACTTATTGG ATCCTTGGCA ATTCATGCGA AGATCGGGCG TTGGGGATCT

59281 CTAGAGAGCC CGTTTTATGA GATTTCTGAT AGATCAAAAG GGGCACGGAT TCTTTATTTA

59341 TCACCAGGTA AAGATGAATA CTATATGGTA GCGGCGGGAA ATCCTTTGGC CTTGAATCAG

59401 GGTATTCAGG AAGAACAGGT TGTTCCAGCT CGATATCGTC AAGAATTCTT GACTATTGCA

59461 TGGGAACAGG TTCATCTTCG AAGTATTTTT TCCTTTCAAT ATTTTTCTAT TGGCGCTTCT

59521 CTAATTCCCT TTATCGAGCA TAATGATGCG AATCGGGCTT TAATGAGTTC GAATATGCAA

59581 CGTCAAGCAG TTCCTCTTTC TCGGTCCGAG AAGTGCATTG TCGGAACTGG GTTGGAACGC

59641 CAAGCGGCTC TAGATTCAGG GGTTCTCGCG ATAGCCGAAC ACGAGGGAAA GGTCATTTAT

59701 ACCGATACTG ACAAGATCCT TTTATCAGGT AATGGAGATA CTCTAAGCAT TCCATTAGTT

59761 ATGTATCAAC GTTCCAACAA AAACACTTGT ATGCATCAAA AACCCCAAGT TCAGAGGGGT

59821 AAATACATTA AAAAGGGACA AATTTTAGCG TACGGCGCTG CTACAGTTGG TGGCGAACTC

59881 GCTTTGGGGA AAAACGTATT AGTAGCTTAT ATGCCGTGGG AAGGTTACAA TTTTGAAGAT

59941 GCAGTGCTCA TTAGCGAGCG CTTGGTTTAT GAAGATATTT ATACTTCTTT TCACATACGA

60001 AAATATGAAA TTCAGACTCA TGTGACAAGC CAAGGCCCCG AAAGGATCAC TAAGGCAATA

60061 CCGCATTTAG AAGCCCATTT ACTCCGCAAT TTAGACAAAA ACGGAATTGT AATGTTGGGA

60121 TCTTGGGTAG AGACGGGTGA GATCTTAGTA GGTAAATTAA CGCCCCAAAT GGTGAAAGAA

60181 TCATCGTATG CCCCCGAAGA TCGATTGTTA CGAGCCATAC TTGGCATTCA GGTATCTACT

60241 TCAAAAGAAA CTTGTCTAAA ACTACCTATA GGCGGTAGGG GTCGGGTTAT TGATGTGAGA

60301 TGGATCCACA AAAGGGGAGG TTCTAGTTAT AATCCAGAAA CAATTCGTGT ATATATTTCA

60361 CAGAAACGTG AAATTAAGGT AGGCGATAAG GTAGCTGGAA GACATGGAAA TAAGGGTATC

60421 ATTTCAAAAA TTTTGCCTAG ACAAGATATG CCTTATTTGC AAGATGGAAG ACCTGTTGAT

60481 ATGGTCTTCA ACCCATTAGG AGTACCTTCA CGAATGAATG TAGGACAGAT CTTTGAATGT

60541 TCACTCGGGT TAGCGGGGAG TCTGCTAAAC AGACATTATC GAATAGCGCC TTTTGATGAG

60601 AGATATGAAC AAGAAGCTTC GAGAAAACTA GTGTTTTCTG AATTATATCA AGCCAGTAAG

60661 CAAACGGCAA ATCCATGGGT ATTTGAACCC GAATACCCAG GAAAAAGTAG AATATTTGAT

60721 GGAAGAACGG GGGATCCTTT TGAACAACCT GTTATAATAG GAAAGCCTTA TATCTTGAAA

60781 TTAATTCATC AAGTTGATGA TAAAATCCAT GGACGTTCCA GCGGGCATTA TGCACTTGTT

60841 ACACAACAGC CCCTTAGAGG AAGGGCCAAG CAAGGCGGAC AGCGGGTAGG AGAGATGGAG

60901 GTTTGGGCCC TAGAGGGATT TGGTGTTGCT CATATTTTAC AAGAGATGCT TACTTATAAA

60961 TCGGATCATA TTAGAGCTCG CCAGGAAGTA CTTGGTACTA CGATCATTGG AGGAGCAATA

61021 CCGAATCCTC AGGATGCTCC AGAATCTTTT CGATTGCTCG TTCGAGAACT ACGATCTTTG

61081 GCTCTGGAAC TGAATCATTT CTTTGTATCT GAGAAGAATT TCCAGATTAA TAGGAAGGAA

61141 GCTTAATCGG AATGAATCAA AATTTTTATT CTATGATTGA TCGGTATAAA CATCAACAAC

61201 TCCGAATTGG ATCAGTTTCT CCTCAACAAA TAAGTACTTG GGCCAATAAA ATCCTGCCTA

61261 ATGGAGAGAT AGTTGGAGAG GTGACAAAAC CTTATACTTT TCATTATAAA ACCAATAAAC

61321 CCGAAAAAGA TGGATTATTT TGTGAAAGAA TTTTTGGGCC TATCAAAAGT GGGATTTGTG

61381 CTTGTGGAAA TTATCGAGTA ATCGGAGATG AAAAAGACGA CCCGAAATTT TGTGAACAAT

61441 GCGGAGTCGA ATTTGTTGAT TCTCGGATAC GAAGATACCA AATGGGCTAC ATCAAACTCG

61501 CATGCCCAGT AACCCACGTG TGGTATTTGA AACGTCTTCC TAGTTATATT GCGAATCTTT

61561 TAGATAAACC TCTTAAAGAA TTAGAAGGCC TAGTATACTG CGATGTGTGA TTTGATCGAA

61621 ATTCTGATTT TACAGATGAT TCGGAATGAA ATTCTGTCAT CCCATTCAAT CCAATCGGGA

61681 TGCCCTGGAC CTGACATGTC GCTTGGGAGG AGTAACATGA AGCTCAGAAT TTAGGGTGTA

61741 TTTAATACTC CCCAATAATA AGGGTAATTG ATCTATGGTA GATTTCGTAA CAAAAAAACG

61801 GGAATTTTTA GTTATACCTC GTAAAAAAGA CTTTTTGGGG GAATTAACCA GCTCCTTTCT

61861 TTCTTTTATT TTAGAAATAA ATTAAGGAAA TAGAAAATAT GTCACGGTTA CAGTAGTCTA

61921 TCCATCGCAT ATAGACTTTA AGGGCATCGT GGCATAACCG TCGAGGTGAA GTCGGGACCT

61981 AAAAGATTGA ACGGAACGGT ACATAGACAA GTAAATCCCT TATGAATTCG AAGGTACTCA

62041 CTTTTTGAAA ATGAAAATGA AGAATTGCGG GATTCATCAT TCGGGGGGAG TAGACTACTC

62101 AAGAATTGCA CATGTTATTT ATCTCCTAAT TGAATAAAGA ATTCAGAAAA TCTAAATAGA

62161 AAGGAAGACG TAATTAAGGA AATCTTCCTT AGTCTTCGTT GGAAACTTGA GTAAGGAGTA

62221 GATCTTTTTT TTTTTGGGGG GGGGTTATAG AATTTTAAAG TTAGAACTCC TTATCTTTAT

62281 TTGATGTACC TACTTGAGCT GTATGAAAGG AAACTTTCAC GTCCGGTTTT GAAGGGGGGG

62341 AGATCCTATA GGATCCTATC CCAATTTTTC TTTTGCTAGG CCCATAGCTA AAAAACCCAC

62401 TTTCTTACGA TTACGAGGTT TGTTCGAATA TGAAATCCAA TCTTGGAAAT ACAGTATCCC

62461 GCTTTTTTTT ACTACCCAAG GCTTCGATAC ATTTCGAAAT CGAGAGATCT CTACTGGAGC

62521 AGGTGCTATC CGAGAACAAT TAGCCGATCT AGATTTACGA ATTATTATAG ATTCTTCATT

62581 GGTAGAATGG AAAGAGTTGG GGGAAGACGG GCCCACAGGG AATGAATGGG AAGATCGAAA

62641 GGTTGGAAGA CGAAAGGATT TTTTGGTTAG ACGTATGGAA TTAGCTAAGC ATTTTATTCG

62701 AACAAATATA GACCCAGAAT GGATGGTTTT GTGTCTATTA CCTGTTCTTC CTCCTGAGTT

62761 GAGACCGATC ATTCAGATAG ATGGGGGTAA ACTAATGAGC TCGGATATTA ATGAACTCTA

62821 TAGAAGAGTT ATCTATCGGA ACAATACTCT TACGGACCTA TTAACAACAA GTAGATCTAC

62881 GCCAGGAGAA TTAGTAATGT GTCAGGAGAA ATTAGTCCAA GAAGCCGTGG ATACACTTCT

62941 TGATAATGGA ATCCGCGGAC AACCAATGAG GGACGGTCAT AATAAAGTTT ACAAGTCGTT

63001 TTCAGATGTA ATTGAGGGCA AAGAGGGAAG ATTTCGTGAG ACTCTGCTTG GCAAACGGGT

63061 CGATTATTCA GGGCGTTCCG TCATTGTCGT GGGCCCCTCA CTTTCATTAC ATCAATGCGG

63121 ATTGCCCCGC GAAATAGCAA TAGAGCTTTT CCAGACATTT GTAATTCGCG GTCTAATTAG

63181 ACAACATCTT GCCTCGAATA TAGGAGTTGC TAAGAGTAAA ATTCGGGAAA AAAAACCGAT

63241 TGTATGGGAA ATACTTCGGG AAGTTATGCA GGGGCATCCT GTATTGCTAA ATAGAGCACC

63301 TACTCTGCAT AGATTAGGTA TACAGGCATT CCAGCCGGTT TTAGTGGAAG GTCGCGCTAT

63361 TTGTTTACAT CCATTAGTTC GTAAAGGATT CAATGCAGAC TTTGACGGGG ATCAAATGGC

63421 TGTTCATGTG CCTTTATCTT TTGAGGCTCA AGCGGAAGCA CGTTTACTTA TGTTTTCTCA

63481 TATTAACCTT TTATCTCCGG CGATTGGGGA TCCTATTTCC GTACCAACTC AAGATATGCT

63541 TATCGGACTT TATGTCTTAA CGAGTGGGAA TCGTCGAGGT ATTTGTGTAA ATAGGTATAA

63601 TCCATGTAAT CACAGAAACT ATCAAAATGA AATAATTTAT GAGAATAACT CTAAGTATAC

63661 GAAAGAAAAA GAACCCTTTT TTTGTAATTC TTATGATGCA ATTGGAGCTT ATCGGCAGAA

63721 ACGAATCAAT TTAAATAGTC CTTTGTGGCT CCGGTGGCGA CTAGATCGAC GCGTGATTGC

63781 TGCAAGAGAA GCCCCCCTCG AAGTTCACTA TGAATCTTTG GGTACCTATT ATGATATTTA

63841 TGGGCAATAT CTAATAGTAA GAAGTATAAA AAAAGAAATT ATTTCGATAT ATATTCGAAC

63901 TACTGTTGGT CATATTTCTA TTTATCGAGA AATCGAAGAA GCTATACAAG GGTTTTGTCA

63961 GGCCTGTTCA TATGGTCCTT AAACTAAGTA ATTCTAGGAT ACCAGTTAGA ATTTATGTCT

64021 CTCCGGTTCA ACTAGGACGG GAATTCCGGA ATTTCTACGC GAATTAAGAT TCGAGAAAAG

64081 GAAGTTTTCC AATCACTGAC TCAAACCCAT TGTAAAATCC TACTCGGCGG AATATGGAAG

64141 TACTTATGCT CAAACTCATT GTCAAATTCT ACTCAGCGGA ATATGGAGGT ACTTATGCCA

64201 GAACGGGCCA ATCTGGTCTT TCACAATAAA GTGATAGACG GAACTGCCAT GAAACGACTT

64261 ATTAGTAGAT TAATAGATCA CTTCGGAATG GCATATACAT CACACATCCT GGATCAAGTA

64321 AAGACTTTGG GTTTTCAACA AGCTACTGCT ACATCCATTT CATTAGGAAT TGATGATCTT

64381 TTAACAATTC CTTCTAAGAG ATGGCTAGTT CAAGATGCTG AGCAACAAAG TTTCATTTTG

64441 GAAAAACACC ACCATTATGG GAATGTACAT GCGGTAGAAA AATTACGCCA ATCTATTGAG

64501 ATATGGTATG CTACAAGTGA ATTTTTGCGA CAAGAAATGA ATCCGAATTT TAGGATGACT

64561 GACCCTTTTA ATCCAGTCCA TATAATGTCT TTTTCGGGAG CTAGAGGAAA TGCGTCTCAG

64621 GTACATCAAT TAGTAGGTAT GAGAGGATTA ATGTCGGATC CTCAAGGACA AATGATTGAT

64681 TTACCCATCC AAAGCAATTT ACGCGAAGGA CTCTCTTTAA CAGAATATAT TATTTCTTGC

64741 TACGGAGCCC GTAAGGGGGT TGTGGATACT GCTGTACGAA CTTCAGATGC TGGATATCTC

64801 ACTCGTAGAC TTGTTGAAGT AGTTCAACAC ATTGTTGTAC GTCGAAAAGA TTGTGGCACC

64861 GCCCGAGGTA TTTCTGTGAG TCCTCAAAAC GGGATGATGC CGGAAAGGAT TTTTATCCAA

64921 ACATTAATTG GTCGTGTATT AGCGGATGAT ATATATATGG GTCCACGATG TATTGCCATT

64981 AGAAATCAAG ATATTGGTAG TGGACTTGTG AATCAATTCA TAACCTTTCG AGCACAACCA

65041 ATATATATTC GAACTCCCTT TACTTGTAGG AGTACATCTT GGATCTGTCG ATTATGTTAT

65101 GGCCGGAGTC CTACTCATGG CGACCTGGTC GAATTGGGAG AAGCTGTGGG TATTATTGCG

65161 GGTCAATCTA TTGGAGAACC CGGTACTCAA TTAACATTAA GAACCTTTCA TACCGGCGGA

65221 GTATTCACAG GGGGTACTGC AGAACATGTA CGAGCCTCAT CGAATGGAAA AATCAAATTC

65281 AATGAGGATT TGGTTCATCC GACACGTACA CGTCATGGCC ATCCTGCTTT TCTATGTTCT

65341 ATCGACTTGT ATGTAACTAT TGAAAGTGAA GATATTCTAC ATAATGTGCA TATTCCGCCC

65401 AAAAGTTTTC TTTTAGTTCA AAATGATCAA TATGTAGAAT CAGAACAAGT GATTGCCGAG

65461 ATTCGCGCCG GAACATCCAC TTTGAATTTT AAAGAGAAGG TTCGAAAACA TATTTATTCT

65521 GACTCAAAGG GAGAAATGCA CTGGAATACC GATGTGTATC ATGCACCCGA ATTTACATAT

65581 GGGAATGTTC ATCTCTTACC AAAAACAAGT CATTTATGGA TATTATTAGG AGAGCCGCGC

65641 AGATCTGATC TAGTCTCCCT CTCGGTCCAC AAGGATCAAG ATCAAACGAA CGCCCGTTCT

65701 TTTTCTGCCA AGAAAAGATA TATTTCTAAC CTCTCAGTAA CTAATGATCA AGTGAGACAA

65761 AAATTCTTTA GTTCGGATTT TTCTGGTCAA AAAGAAGAAA AACGCCCTGA TTATTCAGAA

65821 CTTAATCGAA AGGGTCGTTG TAATCTCAGA GACCCCGATA CTCTCCACGC GAATTATGAT

65881 TTATTGGCAA AGAGACGAAG AAAAAGATTC ATCATTCCAC TCCAATCGAT TCAAGAACGA

65941 GAGACCGAAC TAATGCCCTT TTCAGGCATC TCGATCGAAA TACCCATAAA TGGTATTTTC

66001 CGTAGAAATA GTATTCTTGC TTATTTCGAT GATCCTCAAT ACAGAAGAAA GAGTTCGGGA

66061 ATTACTAAAT ATGGCACTAC AGAAGTGTAT TCAATCGTCA AAAAAGAGGA TTTGATTGAG

66121 TATCGGGGAG TCAAGGAGTT TCGGCCAAAA TACCAACTGA AAGTGGATCG ATTCTTTTTC

66181 ATTCCCGAGG AAGTGCATAT CTTACCCGGA TCTTCTTCCA TAATGGTACG GAACAATAGT

66241 ATTATTGGGG TAGATACACA AATCACTTTA ACTATAAGAA GCCGAGTAGG CGGATTGGTT

66301 CGAGTGGAGA GAAAAAAAAA AAAAATCGAA CTTAAAATAT TTTCGGGAGA TATCCATTTT

66361 CCTGGAGAGA CGGATAAGAT ATCCCGACAT AGTGGCGTCT TGATACCACC GGGAACGGGA

66421 AAAACAAATT CCAAGGAATC CAAAAGGAAA AATTGGATCT ATGTCCAACG AATCACACCT

66481 ACTAAGAAAA AGTATTTTGT TTTGGTTCGA CCTGTAGTCA CATATGAAAT AACAGACGGT

66541 ATAAATTTAG TAACACTTTT CCCCTCGGAT CTGTTGCAGG AAAGGGATAA TATGCAACTT

66601 CGAGTTGTCA ATTATATACT TTATGGAAAC GGCAAACCAA TTCGGGAAAT TTATGACACA

66661 AGTATTCAAT TAGTTAGGAC TTGTTTAGTA TTAAATTGTA CCCAAGACAA AAAAAGTTCT

66721 TATATCGAAG AGGCCTGTAC TTCCTTTGTT GAAATAAGGA TAAAGGGTTT GATTCGAGAT

66781 TTTATAAAAA TCGACTTATC GAAATCCCCT ATTTCATATA CCCCAAAAAG GAATGATCCG

66841 TCGGGTTCAG GATGGATCTC TGAGAATGGG TCATATCGCA CCAATAGAAA TCCGTTTTTT

66901 TCCAGTTTTT TCTACTATTC CACGGAAAGG ATTAAAGAAT CCCTGAATCA AAACCAAGGA

66961 ACTATTCATA CATTGTTGAA TAGAAATAAG GAATGCCAGT CTTTGATAAT TTTGTCATCC

67021 TCCAATTGTT TTCGAATGGG TCCATTCAAC GATGTAAACT ATCACAATGT GATAAAAGAA

67081 TCAATTAAAA AAGATCCCCT AATTCCAATT AGAAACTTGT TGGGCCCTTT AGGAACAGCC

67141 CTTCAAATTC AAATTGCTAA TTTTTATTCA TTTTCCCATT TAATAACTTA TAATCAGATC

67201 TTCGTAACTA ACTATTTGCA ACTTGAAAAC TTAAAACAGA CCTTTCAAGT ACTTCAATAT

67261 TATTTAATGG ATGAAAACGA GAAAATTTAT AATCCCGATT CCTGCAGTAA CATTATTTTG

67321 AATCCATTCA ATTTGAATTG GTATTTTCTT CATTACAATG ATTGTGAAGA GATGTCTACA

67381 ATAATGAGTC TTGGGCAGTT TATTTGTGAA AATGTATGTA TAGTCAAAAA CGGACCACAC

67441 CTCAAATCGG GTCAAGTTAT AATTGTTCAA GTTGACTCTG TAGTAATACG ATCCGCTAAG

67501 CCTTATTTGG CCACCCCAGG AGCAACCGTT CATGGCGATT ATGGGGAAAT CCTTTATGAA

67561 GGAGATACAT TAGTTACATT TATATATGAA AAATCGCGAT CCGGTGATAT AACGCAGGGT

67621 CTTCCAAAAG TGGAACAGGT GTTAGAAGTG CGTTCGATTG ATTCAATATC GATGAATCTC

67681 GAAAAGAGGG TTGAGGGCTG GAACGAATGT ATAACAAGAA CTCTTGGACT TCCTTGGGGG

67741 TTCTTGATTG GTGCTGAATT AACTATAGTG CAAAGTCGTA TCTCTTTGGT TAATAAGATC

67801 CAAAAGGTTT ATCGATCCCA GGGGGTGCAG ATCCATAATA GGCATATAGA AATTATTGTA

67861 CGTCAAATAA CATCAAAAGT GTTGGTTTCA GAAGATGGGA TGTCTAATGT TTTTTTACCC

67921 GGAGAATTTA TTGGATTGTT GCGGGCTGAA CGAATGGGGC GTGCTTTGGA AGAAGCGATC

67981 TGTTACCGAG CCGTCTTATT GGGAATAACA AAAGCATCTC TGAATACTCA AAGTTTCATA

68041 TCCGAAGCAA GTTTTCAAGA AACTGCTCGA GTTTTAGCAA AAGCAGCTCT CCGGGGTCGT

68101 ATCGATTGGT TGAAAGGCTT GAAAGAGAAC GTTGTTTTGG GGGGGATGAT ACCCGTTGGT

68161 ACCGGATTTG GGGGGTTGGT GCACCCTTCG GGGCAACATA ACAACATTTC CTTGGAAAGC

68221 AAAAAAAAGA ATCTATTTGA GGGGGAAATG AGAGATATTT TGTTCCACCA CAGAAAATTT

68281 TTTGATTCTT GCCTTTCAAA GAATTTCCAT GATACATCAG AACAATCATT TCTAGGATTT

68341 AATGATTCCT AAAAGCGGAT TCATCTTTTT ATTTGAAACC AACCTTTATT TTGTCCGGTC

68401 ATTTTATTTA GTAATATTAA AAACAAAAAT AACGAATAGA AAAGAATAAT GTAATGACGT

68461 AGGTCGTGTA TCTACGGCTA ATTTGCGGTA GAAGAGAAGG TTCCATCGGA ACAATTATTT

68521 ATTTTAGGAT ACCCTCGTCT CTTTTTTTTT TTTGAAAAAA GGAGGGGGGT GCGGGGAAAA

68581 ATGACAAACA GATATTGGAA CATCAATTTG GAAGAGATGA TGAAGGCCGG AGTTCATTTT

68641 GGACATGGTA CTAGGAAATG GAATCCTAAA ATGGCCCCTT ATATCTCGGC AAAGCGTAAA

68701 GGTATTCATA TTACAAATCT TACAAGAACC GCTCGTTTTT TATCAGAAGC TTGTGATTTG

68761 GTTTTTGATG CAGCAAGTAG GGGAAAACAA TTCTTAATCG TTGGCACTAA AAATAAAGCA

68821 GCTGACCTAG TAGCATGGGC TGCAATACGG GCTCGGTGTC ATTATGTTAA TAAAAAATGG

68881 CTCGGCGGTA TGTTAACGAA TTGGTCCACT ACAGAAACGA GACTTCATAA GTTTAGAGAC

68941 TTGAGAACAG AACAAAAAAT GGGGGGACTC AACCGTCTTC CGAAAAGAGA TGCGGCCATG

69001 TTGAAAAGAC AATTATCTCA CTTGCAAACA TATCTGGGCG GGATTAAATA TATGACAGGG

69061 TTACCAGATA TTGTAATCAT CGTTGATCAG CACGAAGAAT ATACGGCTCT TCAAGAATGT

69121 ATCACTTTGG GAATTCCAAC AATTTGTTTA ATCGATACAA ATTGTGACCC CGATCTTGCA

69181 GATATTTCGA TTCCAGCCAA CGATGACGCT ATATCTTCAA TCCGATTAAT TCTTAACAAA

69241 CTAGTATTCG CAATGTGTGA GGGGCGTTCT AGCTATATAA GAAATCCGTG ATTAATAATA

69301 ATTAATATAA TTAATAATAA GAGAAATAAC TTAACTTACT TTGGAAATTT TATAGATTTA

69361 TTATGGAATC GTTTACTATT TCTGAATCTC AAATACTATT TATGATTAGG AATCTCAAAA

69421 AAGATAAAAA CCCGGGTATA TTATGTGATT GGTTGGTATT CAATAATGGA ATTGGTATCC

69481 TAAATATAGA TATAGGGTTT AAATAGTCAA GTAGAGAAAG AGATGTTTGA ATCAAAATAA

69541 TTTTCTATAT GTTTGTCAGA GGGCAATATG AATGTTCTAT CCTGTTCCAT CAACACGTTA

69601 AATGGGTTAT ACGAGATTTC TGGTGTGGAA GTAGGCCAAC ATTTCTATTG GAAAATTGGG

69661 GGTTTCCAAG TCCACGGCCA AGTACTTATT ACTTCTTGGG TTGTAATTGC TATCTTATTA

69721 GGTTCAGCCA CTATAGCTGT TCGGAGCCCA CAAACCATTC CGACCGGCGG TCAGAATTTC

69781 TTCGAATATG TACTTGAATT CATTCGAGAT GTAAGTAAAA CTCAAATTGG AGAAGAATAT

69841 GGTCCCTGGG TTCCTTTTAT TGGAACTATG TTTCTATTTA TTTTTGTTTC TAATTGGTCA

69901 GGGGCGCTTT TACCTTGGAA AATCATACAA TTACCTCATG GGGAGTTAGC CGCACCCACG

69961 AATGATATAA ATACTACTGT TGCTTTGGCT TTACTCACAT CAGTGGCATA TTTCTATGCG

70021 GGTCTTAGCA AAAAGGGATT AGGGTATTTC GGGAAATATA TTCAACCAAC CCCAATCCTT

70081 TTACCCATTA ACATCTTAGA AGATTTCACA AAGCCTTTAT CACTTAGTTT TCGACTTTTC

70141 GGAAATATAT TAGCTGATGA GTTAGTAGTT GTTGTTCTTG TTTCTTTAGT ACCCTTAGTG

70201 GTTCCTATAC CTGTCATGTT CCTTGGATTA TTTACAAGTG GTATTCAAGC TCTGATTTTT

70261 GCAACTTTAG CCGCGGCTTA TATAGGTGAA TCCATGGAGG GCCATCATTG ACTAGCATTG

70321 ACTAGTTTTC GAAAGATTCT TTTTTTAGCT TATTCCAAGG CAACGTAGGC ACGGCTCAAA

70381 AAAATAGAAT TTAATTAGGA AATAGAAATA TAAACTCACT ATATACAATC ACTATATACA

70441 ATCTAGTATA CAATCTAGAG TAATAGCCAA AAAGATTACG ATATTAGAGT AGAGAATTGT

70501 AAACTAAAAA AGGGAAAGAG ATCTAAAAGA TCTTTTTCCT AAAATTCCCG GTCGATCCGC

70561 TTATATCATA CCTCTATCCT GAATGAATAT TCGTTTTATA TGGGTCAGCC AATCTGAATA

70621 TCAAATATTC GGAGTCATAA TTTGAATAAG AATTATTGTG GTTTACGACG TGTGATTAAA

70681 AAAAAGGGGG GGATGTTCTA TAGAACGATT CCCCTATTCA GTTGATTTTC TTCAGCGATT

70741 GACCAAACTA AAATTTTCAG AAATCAAAAA TTGCATGCAA TCAAATAATG AGATTTTTAT

70801 CCAACGATTT GGCCTAAGCA ATTTGTCCAT TTAGATTAGA TTGTATCCAT GGGGGATAAT

70861 GATAAAGAAA GGGGAAGGAA AAGAAGAATT TACAAAAAAA GGGAATTCAG AAAAAATAGG

70921 GTGATTCCGA TCAGAGAAGG AGGTCGAACT AGGTATATGT AATTGAATCG CTATGCTATA

70981 AGCCAACTTG TTTCGACGCA CGATTCTCGA ATCCGATTGA ATCTAAGATG AATGAAGAGT

71041 GGGTTTACGT TATGGAAGAA AGACATGTAT ATGTGATATT AGATATTGAC TAGTTCTATA

71101 TGAACTAAAG ATATTGTCCT ACTACTAGTA CTAGAATAGA AATTTCGACG GGAATTCCAA

71161 TAGAAGTTCT TCCGCCCCCT CTGGGACTGT GAGTTGAATG AATAAACAGA TGAAATCCAT

71221 AAAAAGATCG AAGAATTCAA AGAATGGTTC GGACCGAAGA AATGGACGAA TGAAAGTCGT

71281 GCGTATTCAC AAAAACTTTG TTGATAGGAA CTAAAAAAGA GATATCGAAG TAAAGTAGTT

71341 TTGATCATTG AATAATATTA CTTCAATTCG AAGTTTGTAG TTACTTCGAC TGGAGGAATT

71401 GTTAGCGGTG GAATAATTAA GTTAGAATTC ATCGGCTGAT TGTATCATTA ACCATTTCTT

71461 TTTTTTGGTA TGAGGAACTT ATCATGAATC CACTGATTTC TGCCGCTTCC GTTATTGCTG

71521 CTGGATTGGC TGTAGGGCTT GCTTCTATTG GACCTGGAGT TGGTCAAGGT ACTGCTGCGG

71581 GTCAAGCTGT AGAAGGTATC GCGAGACAGC CCGAGGCGGA GGGAAAAATA CGAGGTACTT

71641 TATTGCTTAG TCTAGCTTTT ATGGAAGCTT TAACAATTTA TGGCCTGGTT GTAGCATTAG

71701 CACTTTTATT TGCGAATCCT TTTGTTTAAT CTTAGAACTA AGAACAATAA AATTTTTGCA

71761 TATTTTATTG CCTTGAACCT GTCATTTGCT TTTTCGAATT ATATCAAGAT TTCATTTCTA

71821 CAATGACTTT TTCGTTGAGA AAAAAATCCA TGGGAAGGAC TGATTTGCAG ATGAGGAATT

71881 AGCATCATAC CGATACCGAC TCGCTTTCAT CCTTCCCGCT CGTAGTCCAA GGAACCCCCC

71941 TTTTTTAGTT TTTTAGGAAG TGTTTCAATA CAAATAAAGG GGGTAATTCA CCATTTCAAA

72001 ATCGAATCTT TTTTGACTCG ATTTTGAAAT AGGAAAAGTT CTATATAAAA AAGAAAGGGG

72061 GTAGGGCGAT ACAAAAAGAA CAGAGTTCTT TTTTTTTTTT TTTTAGTCTA TCTATAAGAG

72121 GAGATCGTAT GAAAAATGTA ACCGATTCTT TCGTTTCTTT GGGACACTGG CCGTCCGCCG

72181 GGAGTTTCGG GTTTAATACC GATATTTTAG CAACAAATCT AATAAATCTA AGTGTAGTGC

72241 TTGGGGTATT GATCTTTTTT GGAAAGGGAG TGTGTGCGAG TTGTTTATTT CAAGAATAGG

72301 CTGGATCCAA CCAGCTGTAC TTTTTCCGTT ATAACTAGGA AAGAGAGGTA CATGATCTAA

72361 CGAATGACTT CTGAATAAAG AAATTCAATC ATATGTAAGA ACCATAGCAT TTCGTGATTC

72421 GTTGGTAAAT CCACTTTGAT TCTCTATCAA CCAATAATGT GGGACCATTA ACCATGGTTA

72481 AAGCTAAATC GTTTGAAGTC CAGACGCAGC ATGGTATTCT TTCTACCACT ATATTAATAG

72541 TAATAGAGAG ATGCTTTCAA AATGAATAAT TGATCAATAT AGAACACTCA TATGGATCAA

72601 ATTGTTTGAA CCGCTTATTA ACAAAATTGG GACGAAATCC CCCTTTTTTC CAATGCTGAA

72661 TCGACGACCT ATGTATTGTG TAGAAAGGAA GAAAACCCCT TTTTATTTTT GGATAAAAAA

72721 AAGAAACAAC TTTGCTGACA ATTACAGATT TTTGTTTGGT CAGAAGAGTC CTCCAACTTT

72781 TTGTTTTTGG ATTAGTGATT CGTTTTGATA TTTTTATTTT GGAATATGAA GAGAGAATAG

72841 AGGATAGGCT CATTACATTC AAAAAATATA TGGGGATTTA CCCTAAGTAA TTGAGCGTGA

72901 GAGCCAAATG AATCGAAAGA GTCATGTTTG GTTCGGGAAG GGATCATGGA AGTTTTTAAA

72961 TGAAATGAAT GGAAAGAGAA TCTACTTTCA TTAAGTGATT TATTAGATAA TCGAAAACAG

73021 AGGATCTTGA ATACTATTCG AAATTCAGAA GAACTGCGTG GGGGGGCCAT TGAACAGCTG

73081 GAAAAGGCCC GGGCTCGCTT ACGAAAAGTG GAACTGGACG CAGATCAGTT TCGAGTCAAT

73141 GGATACTCTG AGATAGAGCG AGAAAAATTG AATTTTCTGA ATTCCATTTC TAAGACTTTG

73201 GAACAACTAG AAAATTACAA AAATGAAACT ATTCATTTTG AACAACAAAG GGCGATTAAT

73261 CAAGTACGAC AACGGGTTTT CCAACAAGCC TTACAGGGGG CTCTAGGCAC TTTGAGTAGT

73321 TGTTTGAACA ACGAGTTACA TTTACGTACC ATCAGTGCCA ATATTGGCGT GTTGGGGGCG

73381 ATGAAAGAAA TAACTAATTA GTCCTTCTAC TCTAGGTATT ATTTTTTTTT TCAAAAAAGA

73441 AGTCAGAAAT ATTCATGGTA ACCATTCGAG CCGACGAAAT TAGTAATATT ATTCGTGAAC

73501 GTATTGAGCA ATATAATAGA GAAGTAAAGA TTGTAAATAC CGGTACCGTA CTGCAAGTAG

73561 GCGACGGTAT TGCTCGTATT CATGGTCTTG ATGAAGTAAT GGCGGGCGAA TTAGTAGAAT

73621 TTGAAGAGGG TACAGTAGGC ATTGCTCTTA ATTTGGAATC AACAAATGTC GGTGTTGTCT

73681 TAATGGGCGA TGGTTTGCTG ATACAAGAAG GAAGTTCTGT AAAAGCAACA GGAAGAATTG

73741 CTCAGATACC CGTGAGTGAG GCCTATTTGG GTCGTGTTGT AAATGCCTTG GCTAAACCTA

73801 TTGATGGTAG AGGTGAAATT TCAGCTTCTG AATATCGGTT AATTGAATCT CCCGCTCCGG

73861 GTATTATTTC ACGACGTTCT GTATATGAGC CTCTTCAAAC GGGGCTTATT GCTATTGATT

73921 CGATGATCCC TATAGGACGT GGTCAGCGAG AATTAATTAT TGGGGACAGA CAGACCGGTA

73981 AAACAGCAGT AGCAACAGAT ACGATTCTCA ACCAACAAGG CCAAAATGTA ATATGTGTTT

74041 ATGTAGCTAT TGGTCAAAAA GCATCTTCCG TGGCTCAGGT AGTGACTACT TTCCAGGAAA

74101 GGGGAGCGAT GGAATACACT ATTGTGGTAG CTGAAACGGC GGATTCTCCT GCTACATTAC

74161 AATATCTCGC TCCTTATACA GGCGCTGCTC TGGCGGAATA TTTTATGTAC CGTGAACGAC

74221 ACACTTCAAT CATTTATGAT GATCCCTCCA AACAGGCACA AGCTTATCGA CAAATGTCTC

74281 TTCTATTACG AAGACCGCCC GGTCGCGAAG CTTATCCAGG GGATGTTTTT TATTTGCACT

74341 CACGTCTTTT GGAAAGAGCT GCTAAATTAG GTTCTCGTTT AGGTGAAGGA AGTATGACTG

74401 CTTTGCCAAT AGTTGAAACT CAATCGGGAG ATGTTTCGGC TTATATTCCT ACTAATGTAA

74461 TTTCGATTAC TGATGGACAA ATATTCTTAT CTGCCGATCT ATTCAATGCT GGAATCAGAC

74521 CTGCTATTAA TGTGGGTATC TCCGTTTCCA GAGTGGGATC TGCAGCTCAA ATTAAAGCTA

74581 TGAAACAAGT TGCTGGCAAA TTAAAATTGG AACTGGCGCA ATTCGCAGAA TTAGAAGCCT

74641 TTGCACAATT TGCTTCTGAT CTCGATAAAG CTACTCAGAA TCAATTGGCA AGAGGTCAAC

74701 GACTACGTGA ATTGCTCAAA CAATCCCAAT CAGCCCCTCT CGCAGTAGAA GAACAGGTAA

74761 TGACTATTTA TACCGGAACA AATGGTTATC TTGATTCATT AGAAATCGGA CAGGTAAGGA

74821 AATTTCTTGT TGAGTTACGT ACTTACCTAA AAACTAATAA ACCCCAGTTT CAAGAAATTA

74881 TATCTTCTAC CAAGATATTC ACCGAGGAAG CCGAAGCCCT TTTGAAAGAA GCTATTCAGG

74941 AACAGATGGA ACGTTTTATA CTTCAGGAAC AACCATAAAT AATTTTAGAA AAATTCTCAA

75001 AAACAAAAAA AGGATAATTG AGCGTCTTGC ATTCCAATCT TTCAAAAAGG CTTTCTTGAT

75061 ATCGAAATAT AAAATATATA TGGAAATAAA TTGCGTCCAA TAGGATTTGA ACCTATACCA

75121 AAGGTTTAGA AGACCTCTGT CCTATCCATT AGACAATGGA CGCCCTTCAT TGAGATTATT

75181 TCGTATTTTG ATTTTCCTAC TTCTTGTTTT TATTTGTTGT TTGGATAAAA AGAATAGTTT

75241 GAGATAATTT TTGGAATTCT ATATTCAATG ATGAATCAAT TCGAATGAAT AATTCGAGTC

75301 TATAAATAAT AATATATCGA AAAAAAATTA TGAATATATT CTAGGGGCTA TATAGATATA

75361 GCGGGTAGCG GGAATCGAAC CCGCATCGTT AGCTTGGAAG GCTAGGGGTT ATAGTCGACG

75421 TCGATTCAGC ATTGTTAACG TCTCTAATTC AAAACCGAAC ATGAAACTTT GGTTTCATTC

75481 GGCTCCTTTA TGGAAGATGG ATAAATTCCT AGATATAAGA TGTGAACTAA ATTACAAAAA

75541 ATTAGACCCA TAACCTCTAT GTCAGCTCTT TGTTTGAATA CATTCAAAAC GATTCGCTTT

75601 CTAGAGGATC CCTCTAGAAA AAGGTGATTA TGACAACCTT TCTAGTTACT TCGTTCTCTA

75661 TTTCTATTTG AAAGGATCCC GAGGAAAAAA GTTTTGTTTC CACCGAGCTA CAATAATATG

75721 TCGATAACTC TAGTAAACTA AAGTCATCAT TTAATAGCTA TACTATACTA TTTTGCTTCA

75781 ATTTAGTTTC TACAAATAAA AACAAAATTG GAAGATTTAG TTACGATTAG AAATCTACTT

75841 TTTTATCTCC ATCCATGGAT CCTTTACTCA TACTCAATCA ATTGGAAGTA TTGATCCAAT

75901 TCAAAAATTA AATAATTATG TTTCGCGATT TCGTAATCCA GTTTTTAAGT GACCTTTGGA

75961 TACAAATCAC GAGAATTTCT ATTTTTCCTC GAAATCTGTC ATTGAGAGGA AAAGGATTAA

76021 ATCCTTTTAA GAAATAAAGT TTTTTGATCG GAATATGAAT TAAACCGAAA GACCCCTTAA

76081 CTATTTAAGG GAGTTACTAG AACGAATCAC ACTTTTACCA CTAAACTATA CCCGCTACAA

76141 TGTGATTATT ATATACAAAG GGCCTTTTGT CGAACAGGGG GTATTAGGGC CAGCAAGATA

76201 AGATCATAAA AGAATCATGA AAATGAAAGT ATTGACGTTT TTCGTGGGGG ATTCAAATTG

76261 AAAAAAATTC CTCAAAAACA AAAAAGAGGG CTAATTTAAA TAACTAAATA AAAAGTTCGA

76321 TCTTTTCTTT GCTGGAGGCG CTTTGATAGA TACAATTCGC CAAAACCGTG GAAATTCTAA

76381 TAAAAAATGC ATTGTGTAAA AATCCAAAGT TTCCTTTTTT TATTTTATTC CAGTAAGAGT

76441 AAGGTCGTGA AGTAAATAAA AAAGTAAGAA AGAAGAATAA AAAATTTATA AAGATTTTTT

76501 TCTATAATAA AAAGAATATA GAATATATAA ATAGAATAGA ATGGAAAAAG TCCTTCGTCT

76561 TTTTGTTGTT GCTTTAATAG CAAACTTTTA GTTTTAAAAT CGGGAGAAGC CGTTTAGCCA

76621 GGATTCGTTG GAACAAAAAA GGGCCCGGCT AGGTACTTAC CAGGCCAGGC CGCGGGAATT

76681 AAAAAGGGCC CCTCCTTTCG AACAAAATCA AAGTAAAAGG GGTCGTCTTT CTTTTTCTAT

76741 TGACTCTTTC GAGCCCTTAC TTGAACTAAA TGGAATTTGC TAATAAAAGT TGTAGATATA

76801 TAATATAGAA AAAGAAAGAG AAAGAAAGAC TTTTTCAACT TCAACCCTTA TTTGATGTGT

76861 AATGTAACAT AGTAATTATC TTTTTCACTT GGGAGAGATG GCTGAGTGGA CTAAAGCGGC

76921 GGATTGCTAA TCCGTTGTAC GAGTTATTCG TACCGAGGGT TCGAATCCCT CTCTTTCCGT

76981 CTTTGTTGAT GACTTGATAT TTGATTTATC TTTCCAATTT TGCCAATACT TTTTATTTTT

77041 TCCTAGTTAA ACGTATGGAA TAGATAAAAA AAATCTTAAG AAAATTCAAA AATCAAGCAA

77101 CTAAAACCTT GTTTTATTCT TCACGTCCAG GATTACGACC TGGATCATTA GATAGGAATC

77161 CAAAGATAAA TAGAGAAACA AAGAATATCA CTACTGTGTA AACGAAAAGT TTGAGAGTAA

77221 GCATTACACA ATCTCCAAGA TCCTTTTTTT GGAAAAATGA GAAAAGAGAA TAGATCCTCC

77281 ATTTTTTAAT ACGAAATATT TTGACACCAA TAAATAATAA ATGAAGTGCT TTCTAGAAAC

77341 AGAAAATCAT TAATGAAAAT TCAGTTGTCA TAAGAGTCTT TCATTACTAA AAATTTCTTT

77401 CATACTCACA ATTGGATATT GTGGGGACCC TAACCTAACC CTATGTAGGG TCCTTCAATG

77461 GAAAAGGGCA GAATGGGGAA TACGGGGTGA GCCAGATTTG TATTTCTCGT GGCTATCCAA

77521 GACTTTCAAT GTTTGAATCG AGGGTTCATA ATGTAAGACT TATTTGATCT TATTCATTGT

77581 TAGAATTTTT GCTCGAATAA ATCATGAATT TTATAGGATA ATAGGAAAGA TCTCATCGAA

77641 AACTTACAGC AGCTTGCCAA ACAAAGGCTA AGAGAAAAAA GAACAAAGGT ATGACTGGCA

77701 TAAGATCTAC GATTGGATTC AAAAAAGCAT AGGCCTCGGG CAATTTGGCG AAGAAAAGAC

77761 TACTCGAATA AAAGGCAGAA TTAAGACAGA TACAGATCAA ACTAAAGATA TTAAGCATAA

77821 CAGACATTTT GTTCTTGGAG ATAATTGCAT TTTGATTGAG TTATTGCTAT AAGGAAAAAT

77881 GAAGTAAAGT AAGGTAGACA CAAATCCTTC TTTTTCTTTG AACCCACCCA ATCAAAAATT

77941 CCCCAATTCT CAAACAAAGG AGAATAGAAG AAATCATACT TTCATAGAAT ATCTTAATTG

78001 AATTATATGT AATGATCAAT AAATTCAGTT GAATTCTATA TCTATATCTA CACGTGTAAA

78061 AATTATAGAA AGAATCTAAT TTCTTCTATA AAGATTTTCT ATAGACATTT GTCCTGGAAT

78121 TGACCAAGGC CCAATAATAC TATTATTCTT TATTAGTGTC GAATGGAAAT CTAAATGGGG

78181 CGTGGCCAAG TGGTAAGGCA ACGGGTTTTG GTCCCGGTAT TCGGAGGTTC GAATCCTTTC

78241 GTCCCAGGAC ATATACATTG TATCAGAGTC AAAATTTCTT TTGAAAATAA CGTTCTGTTT

78301 AAATGCCTAA TATTAGATTG GATAGAATGT AATTTTTGTT TCTTTTATGA TTTTGAATGA

78361 TTCTTCTATG AATCATATCT TTGTTTTTCA CAAACCGAAC TAACTGAATT GAGTGCAAAC

78421 GACATGCAGG TTGAACTAAA TATATTTATT TGTGGTCGCG GATTTTTCAT CATATGGTCA

78481 TTTCCCTCAT ATTGTCATAT ATGAAGCGGT TTAGTGACTT AATTTGAAGA GAAACCTTAT

78541 GTCTCATATT TAATTTTCAA CGGTCTACTT TGAATCGCAA TAAGAAAGAA CCTCTTGAGA

78601 CGGGGGGAAT TCGTAAAATT CAGTCACTTT ACTTTATGCC AAGGTTGCAT AAAGATTACT

78661 AAATTAAATC CCGGGTCAAA CAAAAAAATA CATATAAAAT GAATGAGTAA ATGCTTAGCT

78721 TAAGTTAATT AAATGTATTG TTATCATTTG ATTTCGTTCT ATTTCATTGA CAGCAGTCTT

78781 TCGGTTTTTG AGAAAAAGAA TTGGAATCCC TTCAATATTG ATTAATATTG AAATAGCGCA

78841 TTTTCTTTTT TAATATAGAC TATCCATAAC AGAGACTGTT GTTCAGTCTA TCCGATAGGT

78901 ACGAAAAACC CATACTCGTT CATCTGATTA ATAAAATTAG AATCGAAAAA AAAAGTACCC

78961 AAATCTTCCC CTCTATCAGT CTTTTTTATC CATCTCACGA AAAATTGGGT TTCTTGGTCA

79021 ATCTATTTAT CTGCTTTAAA TCATCATATC ATCCTTGATG CTTCAATTCG AAAAGAAACA

79081 GAGATTTCTC TTTTTTTTTT TATGCTGATC GAATGTAGTC TTTAATGGAA AACTTTATTC

79141 AAGAAAACTT TATTCAAATC ATTTTTTCAT TTTTCTAATC GAAATTGGAA GCTCTTTTGA

79201 TTAGAAAAAT GATTAATGAT TGCTTATGAG TTGAATCTTT GGTATTCAAA GAAGTAGGGG

79261 ATGGGTCAAT ATCTCCTATT GAGTCACTTT AAGATGCAGA GTCAAAAAGA ATTCATTTAT

79321 TCGTGTTATT TCTCTATTTA TTTATATTAG TTTGTTTTTT TTTTATAAAA ACTCTTTTAT

79381 TCATATAATA ATATATAATA ACATAAAAAA AAGGGGTCTT GCTAAGATGA TTTCTTCAGA

79441 AAAACTCTTT ACCCCCTTGG TATAGAAGAA AACAAGGTAT AGATTCATAT GTCTATGTAC

79501 CGGCTGAACC AATGACTATT CATGATTCCA CAATTGAATC AATTGCATCC GGGTTCCAAA

79561 TGAGGGGAAT GTTATGTTAT GGTAAAACTT CGTTTGAAAC GATGTGGTAG AAAGCAACGT

79621 GCGACTTGAA GGACACGATC CGATGTGGAT TCTTATTCTT ACATCCGCCA TCTTTTATAG

79681 GACCGAAGGT GCTCTCGGCC CGACATCTGT TCTATTCCAC TATAATCCCT CTTTTTGTTG

79741 GATTGTAATT AATATAGTAC ATGATGGAGC TCGAGTAGAA AGTATTGATT CATCTTTCAG

79801 GGGGTAAGGA TCTAGGGTTA ATGCCAATCA ATAAATTGGA ACAACTTCGT AAGTATATCA

79861 TCAATATAGA AATCGAAAGG ATGCGATTCG GTCAAGTTTT CAATTCAAAA GGAAAATTTA

79921 TTGGAATTGA GAAAACTCTT TCGATTCAAA GTGTATCACG CGGGAATCGA CCGTTCAGAT

79981 GATTCTTTGA TAGAAAGAAA TCACAAAAGG GGCGTGTTGC TGCCATTTTG GAACGATTAA

80041 GAAGCACCGA AGTAATGTCT AAACCCACTG ATTTAAAACA AAGAAAAAAG GATCCCAGAA

80101 CAAGGAAACG CCATTTCAAT TGTCTCAATA ACTGGATCAT ATCATAATAA AGAATCCAAA

80161 TATTTTTTCT TTTAAACGAG ACAAACAAGA GGGGGGTTAA AGACCATTCA ATAAATGAAA

80221 TAAATTGCCG AATTTAGAAT TATCCAACTT GAGTTATGGG TACAAATGAT TTTTCTTTTT

80281 TCAGGAAGGG CGAACAAAAA AGGAGTGAAA TCCCAGTGTA ATTTATTTTA TCAACCTCCT

80341 TGCCATTCAT TAGAATACGT AGACAAAACT GCGAATCATT TTTTCTCGAG CCGTACGAGG

80401 AGAAAACTTC CTATACGTTT CTAGGGGGGG GGTCTTGTTT ATTTACTTAC ATCTATCCCA

80461 ATGAGCCGTC TATCGAATCG TTGCAATTGA TGTTCGATCC CGAAGAGAAG GAAGAGATCT

80521 TCGCAACGTG GGTTTTTATG ATCCGATAAA GAATCAAAGT TATTTAAACG TTCCTGCTAT

80581 TCTAGATTTC CTTGAAAAGG GCGCTCAGCC TACGGGAACT GTTCGAGATA TTTTAAGAAA

80641 GGCGGAGGTT TTTAAGTAGC TTTGCCCCAA TCAAACGAAA TTTAATTAAG GAACTAAAAA

80701 AGGGATAAAA ATTTGTATAT TGTATAACTT TTATATACAC TTTTTTATTT ATTTATTTTT

80761 ATTTCTCCCC TTTTGGGTAC TATTCGTATC TATTTTGCAT TGCTTCTATA AACTCTTATG

80821 TTCTATAACG ACAAAACCCA GTTGGTTGAT CCTAGAAATC GAAAATTACG GTCTTTCCTT

80881 CAATTGGGGG TTTTCGTTGG AACTATACTA ACAAGTAATA ACCAAGGAAT CCAATTTGTT

80941 TATTCTAGTT ACTTATTTTT GATTGAATAA ATCGAAATCC TATATTTTTT CTACTTCTTC

81001 CTTTATTATT CCTCTATCTA AACTTTTTGC GTCGATGTAG TGCCAATCCA ACACAAGTCC

81061 TTTTTTTTAC TATATTATAT ATTATTGAAA TGGAATTTTT TTGTTTTTCC ATTCTATTTT

81121 TTTTTCTCAA CATTTATATT GACAACAGTG TATCGAACAA ATATAATTCA TCGTGATAAG

81181 TAGAGAAATT TCTTTCTATC CCATAGGTTT GATTTTGACC TTCCATCATT CAAATGGTGG

81241 GTTCCTTGGA TTCGCTTTGA TACCATTTTT TATAAGATAT AAAAAGAATA AATAGGGTTT

81301 TTTGATTGAA TGAAAAGTCG ATACCATAAG AGATAAGAAG TGGTCTATCC ATTTTTACAT

81361 TTAAATTATA TTTCTCTATC TTTTCCTTTT TTTATCGGAG AATTTCTTGT ATTTTCGTCT

81421 CATCTATTCA TTTTGATGTT CCGATTCAAT TTCAAAATGT TTTTTTCTTT CTTAGTCTTA

81481 GTTCAAAGCT TTGATTGCCT TGTCTAATCT TTTTTTATTT TGATTGTATC TCTTTTTTTA

81541 TTCGGGTTGC TAACTCAACG GTAGAGTACT CGGCTTTTAA GTGCGGCTAG GCTCTTTTAC

81601 ACATTTGGAT GAAGCGAGGG ATTCGTCCAT ACCATTGGTA AAGTTTGGAA GACCACGACT

81661 GATCCTGAAA GGGGATGAAT GGAAAAAATA GCATGTCGTA TCAACAAAGA GTTCTGAGAA

81721 TATTTCATTC TTTCCGGATC GGTACAAAGC CTTGTGTTCT TTTTTGAAAC GGAAGAAAAT

81781 GAATTCAATG TCAAGTTGGG TCGAGTGAAT AAATGGATAG AGATATAGCC CTATGGCTTC

81841 AATTTATTGA TTATAGGGAA AAAGCAACGA GCTTCTGTTC TTAATTTGAA TGATTACCCG

81901 ATCTAATTAG ACGTTAAAAA TATATTAGTG CCTGATACGG GAAGGGATTC TCCTATGAAT

81961 GGATTCTTTT TTTTTTTTTT GAATCCTAAC TATTGCTATT GCCATTTTTT TTTATGAAAT

82021 GGAAATGTGT GTGTAGAAGA AACAGTATAT TGATCAAAAA ATTCCAAAAT CAAAAGAGCG

82081 ATTGGGTTGA AAAAATAAAG GATTTCTAAC CATCTTGTTA TCCTATAACA AACATAAACC

82141 AATTCGTTTA AATGGAACAG AGAGGATAGA GAATCTGTTG ACAAGTTTAC CTGTATCCGA

82201 GGTATCTATT CGTTTATTAC TAGAATACCT TGTTTTAACT GTATCGCACT ATGTATCATT

82261 TGATAAACCC CAAAATCCTC TCTCTTTAGT TCAACTAGAA TTTCAAATGG AGGAATTCCA

82321 AAGATATTTA AAGCTAAATA GATCTCAACA ACACTACTTC TTATATCCAC TTATCTTTCA

82381 GGAGTATATT TATGCACTTG CGCATGATCA TGGTTTAAAT AGAAATAGAT CGGTTTTGTT

82441 GGAAAATGTA GGTTATGACA ATAAATTCAG CTTACAAATT GTGAAACGTT TAATTGCTCG

82501 AATGTATCAG TATCAACAGA AAATTTTTAC TCTTTCTGCT AATTCTTTTA ACCAAAATAT

82561 CTTTTTTGGG CACAACAAGA ATTTGTATTC TCAAATGATA TCAGATGGGT TTGCAGTCAT

82621 TGTGGAAATT CCATTTTCTT TACGATTAAT ATCTTCGCTA GAAAGGAACG GGATAGTAAA

82681 ATCTCAAAAG TTACGATCAA TTCATTCAAT ATTTCCATTT TTAGAGGACA AATTTTCACA

82741 TTTAATTTAT GTGTTAGAGA TACTAATACC CTACCCGGCC CATCTGGAAA TATTGGTTCA

82801 AGCTCTTCGC TACTGGATAA AAGACGCCTC TTCTTTGCAT TTATTACGAT TCTTTCTTCA

82861 CGAGTATCGT AGTTGGAATA CTCCAAATAA AGCCAGTTCT TTTTTTGCAA AAAGAAATCA

82921 AAGATTATTC TTCGTCCTAT ATAATTCTCA TCTATGTGAA TATGAATCCA TCTTCGTCTT

82981 TCTCCGTAAC CAATCTTCTC ATTTACGCTC AACATCTTCT GGAACCCTTC TTGAACGAAT

83041 CTTTTTCTAT GGAAAAACAG AACATTTTGT AGAAGTTTTT GCTAAGGCTT TTCAGGCCAA

83101 TCTGTGGTTG TTGAGGAATC CTTTCATGCA TTATGTTAGG TATCAAGGAA AATCGATTCT

83161 TGCTTCAAAA GGGACACCCC TTTTGATGAA AAAATGGACA TATTACTTTG TAAATTTATG

83221 GAAATTTCAT TTTGATCTGT GGTATCAACC AGGAAGGATC TGTCTAAACC AATTATCTAA

83281 TCATTCCCTC GACCTTCTGG GCTATCTATC AAGTGCGCGG CTAAACCCTT CAATGGTACG

83341 CGGTCAAATG CTGGAAAATG CATTTCTAAT TGATAATGCT ATTACTAAGT TCGATACTAT

83401 TGTTCCAATT ATTTCTCTGA TTGGATCATT GGCTAAGGCG AAATTTTGTA CCGTATTGGG

83461 ACATCCTATT AGCAAAGCTG TTTGGTCGGA TTTCTCAGAC TCTGATATTG TTGGCCGGTT

83521 TGGGCGTATA TGCAGAAAGA TTTCTCATTA TTATAGCGGA TCCTCGCAAA AAAAGAGCTT

83581 GTATCGAATA AAGTATATAC TTAGACTTTC TTGTGCTAGA ACTTTGGCTC GTAAACACAA

83641 AAGTACTGTA CGTGCTTTTT TGAACAGATC GGGCTCGGGA TTCTTGGAAG AATTCTTTAC

83701 GGCGGAAGAA CAAGTTCTTT ACTTGACCTT TCCAAGAGCC TCTTCCGCTT CGCAGAGGTT

83761 TTATAGAAGG CGTATTTGGT ATTTGGATAT TCTTTGTATC AATGATTTGG CCAATAATGA

83821 ATGATTCGTT AGGAATTAAT GTAAATGTAA ATTGTCATTC AAAAATATAA GTAATGAAGA

83881 TAAATAATGA AGAGATAACG AAAAAAGCCA TTTTTTTTTT CTGAAATGTT GATGTAGGAT

83941 GTAGTAAGGG TTAAATCAAC CAAGTATTCA ACTTTTTGAA AGTTCTAAGT AAGGAACCGA

84001 GTTTTAGATG TATACATAGG GAAAGCCGTG TGCAATGAAA AATGCAAGCA CGGCTTGGGG

84061 AGGGGTGTTT ACCTATTTAA CAAGGAAATT ATCTACTCCA TCCGACTAGT TCCGGGTTCG

84121 AATCCCGGGC AACCCACTGC CACAAAAAAA GGATATGTAA AAGGGGATAT GTATAGAATT

84181 CCTTTTGTTA TTGTTTCAAT CCACTTCCAA AAAATTTGCA TAGATCTAGA TAGATATTGG

84241 TTGACAAGGA CATATAAGCC GTGTTATACT GTTGAATAAC AAGCCCTCTG TTTTCTCTAT

84301 TTCGAGTTAT AGAGAATTCG TGTGCTTGGG AGTCCCTGAT GATTAAATAA ACCGAGATTT

84361 TACCATGACT GCTATTTTAG AGAGACGCGA AAGCGAAAGC CTATGGGGTC GCTTCTGTAA

84421 CTGGATAACG AGCACTGAAA ACCGTCTTTA CATTGGATGG TTTGGTGTTT TGATGATCCC

84481 TACTTTATTG ACCGCAACTT CTGTATTTAT TATCGCCTTC ATTGCTGCTC CTCCAGTAGA

84541 TATTGATGGT ATTCGTGAAC CTGTTTCTGG ATCTCTACTT TATGGCAACA ATATTATTTC

84601 AGGTGCCATT ATTCCTACTT CTGCAGCTAT AGGTTTGCAC TTTTACCCAA TCTGGGAAGC

84661 GGCATCTGTT GATGAATGGT TATACAATGG TGGTCCTTAT GAATTAATCG TTCTACACTT

84721 TTTACTTGGT GTAGCTTGTT ACATGGGTCG TGAGTGGGAG CTTAGTTTCC GTCTGGGTAT

84781 GCGACCTTGG ATTGCTGTTG CATATTCAGC TCCTGTTGCA GCTGCTACTG CTGTTTTCTT

84841 GATCTACCCA ATTGGTCAAG GAAGTTTTTC TGATGGTATG CCTCTAGGAA TCTCTGGTAC

84901 TTTCAACTTC ATGATTGTAT TCCAGGCTGA GCACAACATC CTTATGCACC CATTTCACAT

84961 GTTAGGCGTA GCTGGTGTAT TCGGCGGCTC CCTATTTAGT GCTATGCATG GTTCCTTGGT

85021 AACCTCTAGT TTGATCAGGG AAACCACAGA AAATGAATCT GCTAATGAAG GTTACAGATT

85081 CGGGCAAGAG GAAGAAACTT ATAATATCGT AGCCGCTCAT GGTTATTTTG GCCGATTGAT

85141 CTTCCAATAT GCTAGTTTCA ACAACTCTCG TTCTTTACAT TTCTTCCTAG CTGCTTGGCC

85201 TGTAGTAGGT ATCTGGTTCA CTGCTTTAGG TATCAGTACT ATGGCTTTCA ACCTAAATGG

85261 TTTCAATTTC AACCAATCTG TAGTTGATAG TCAAGGCCGT GTAATTAATA CTTGGGCTGA

85321 TATCATTAAC CGTGCTAACC TTGGTATGGA AGTTATGCAC GAACGTAATG CTCATAACTT

85381 CCCTCTAGAT CTAGCTGCTG TTGAAGCTCC ATCTACAAAT GGGTAAGACC GGGTCTTAGT

85441 ATATACGAGT TTTTTGAAAT AATAAAGGAG CAATAACCTC TTTCTTGGTT TATCAAGAGG

85501 GCGGTATTGC TCCTTTATTT ATTTATGGGG TTGGTTCATG ATTGAGTATC TGTATTAATT

85561 AATTAATCAT TATAATATAC AATTCGGCTT CCTTTTTACC TTCCCAACTT TTGGGAAAGT

85621 GAAGTATTTA TACAAAATGG GATTGGAATT GGATTGGAAT ATAATATATA ATACTTATAT

85681 TAAAATTAGA ATATTAGAAT TCTAATACTT ATTATTATTA GAAATATATA TTTTTTTTCT

85741 CGAAAAAACG AAAGAAATTG TTTTGAATGA TCAAAAATGT CAATAAAAGA AAACGGGGGG

85801 GCGGATGTAG CCAAGTGGAT CAAGGCAGTG GATTGTGAAT CCACCACGCG CGGGTTCAAT

85861 TCCCGTCGTT CGCAATTTTT CTTAATAAAC GATTCGCTAC AAAAGGATTT TTTTTTAGTG

85921 AACGTGTCAC AGCTTACTCC TATTTTTTTT TTTTTTTTTt TTTAAAGACG AAGAAATAAA

85981 TTCTATTTTC TCTCCTATTT ACTACGGCGA CGAAGAATCA AATTATCACT ATATTTATTC

86041 CTTTTTCTAC TTCTTCTCCC AAGTGCAGGA TAACCCCAAG GGGTTGTGGG TTTTTTTCTC

86101 CCAATTGGGG CCCTTCCTTC ACCACCCCCA TGGGGATGGT CTACAGGGTT CATAACTACT

86161 CCTCTTACTA CAGGACGCTT ACCTAGCCAA CGCTTAGATC CGGCTCTACC CAAACTTTTC

86221 TGGTTCACCC CAACATTCCC CACTTGTCCG ACCGTTGCTG AGCAGTTTTT GGATATCAAA

86281 CGGACCTCTC CAGAAGGTAA TTTTAATGTG GCCGATTTCC CCTCTTTTGC AATCAGTTTC

86341 GCTACAGCAC CCGCTGCTCT AGCTAATTGT CCACCCCTTC CACGTGTGAT TTCTATGTTA

86401 TGTATGGCCG TGCCTAAGGG CATATCGGTT GAAGTAGATT CTTCTTTTTG ATCAATCAAA

86461 ACCCCTTCCC AAACTGTACA AGCTTCTTCC AAAGCATACG GCTTTCTGGA TGTAGATGAT

86521 ATCTATACAG ATGGATCTTA TATATCCTAC AATGAAGTAC CACATGGATA TATAGGAATC

86581 CAAATCCGCC GAATCACTCA TGTTATGATC TTCTACATCC TAGGTCTTCC CGTTCCGTCA

86641 TCTGGCTTAT GTTCTTCATG TAGCATTCAG ACCGAATGAC TCTATGAAAT TACGTTGATA

86701 CTTCCACATA TTATGGGTAA CGTAGGAGAC ATCTCTATTT TCCCCCGGGG AATCTTTAGA

86761 ATTACCACTG CTTAGCTTTC AATTCGCCTC TGACCATCAA ATGAAATGTG AATAACCCGT

86821 CCTCTTCTTT TTGAAAGAAG GGGCGCTTCC GGTTCTGTCG GTGCTTGAAA CAATTTTGTC

86881 TTCTCCATAT TACTATATCT CTAGAGTCAA TAATTTTATA TGAGGAACTA CTGAACTCAA

86941 TCACTTGCTG CCGTTACTCT TCAGTTTTCT GTTGAGGTCT ATCCTGTAGA GGTACTCAAA

87001 TTGGATCAGT GATCGATTTA TAGGTTTCGT CGTAAACCTA ATTGGTTACT TCCAATTACG

87061 TAAATCTATA GTTCAAACCG CACTCAAAGG TAGGGCATTT CCCATTTTTA TAGGAACTTC

87121 TGTACCAGAA ACAATGGTAT CTCCAATTAT AGCCCCTCTG GGATGTAAAA TATATCTCTT

87181 CTCACCATCC CCATAGTGTA TGAGACAAAT GTATGCATTT CGATTAGGGT CGTATTCTAT

87241 GGTTACGATT CTACCATATA TGTCTTTTTC ATTCCGCCGA AAATCGATTT TACGGTATAG

87301 ACGCTTATGA CCTCCCCCTC TATGCCCTGC GGTAATGATT CCTCTGGCAT TACGACCTTT

87361 CCCACAACGA TGCTGTCCAT AGATCAAATT AGTTCGTGGA TTGGATTTCA CTTGACTGTC

87421 TACGGTTCGA TTGCGTGTGC TCGGGGTAGA AGTTTTGTAT AAATGTATCG CCATGCTATT

87481 AAGTATTTTG ATTTAAGTTC TTTTCTTTCT AAGAGGTGGA ATAGAATAAC CCGGTTGAAG

87541 CGTAATGATC ATACGTCTGT AATGCATTGT CTGTCCCATA ATAGGTCCCA TTCTTCTACC

87601 CTTTCCCGGA AGTCGATGAC TATTCATAGC TATTACCTTG ACACCAAAGA AGAGTTCGAC

87661 CCAATGCTTT ATTTCTGTCC TAGTTGATCC TGATTCGACA TTAGAAGTAT ATTGATTTTT

87721 CCCCAATAAC CGAATACTTT TGTCTGTAAA TACTGCATAT TTGATTCCAT CCATAACTAT

87781 ATTTTCTTCC CTATGAGTTC TAGTCTCAAT AAGAATGCTA GTTCTTACTG TTCATATATT

87841 ATTATGATAT GAATATATGA TATGAATATA CCACACTAAT TCGTTATGTA TGGATGATGA

87901 GATTCCATTG ATACAGAGCC AATTCCAATA GACTTATTGG AGGGTCCCAT TGGCGTGCAT

87961 CCAGTAGGAA TTGAACCTAC GAATTCGCCA ATTATGAGTT GGGCGCTTTA ACCATTCAGC

88021 CATGGATGCT TAGCGGGGAT CCTCGTACAT GGTGAATAAC CAAATTCCAA TTGAAACGAA

88081 ATCTTTAGAA TAAATCAATG CAATTTAGGA GGAATCAATG AAAGGACACC AATTCAAATC

88141 CTGGATTTTC GAATTGAGAG AGATATTGAG AGAGATCAAG AATTCTCACT ATTTCTTAGA

88201 TTCATGGACC CAATTCAATT CAGTGGGATC TTTCATTCAC ATTTTTTTCC ACCAAGAACG

88261 TTTTATAAAA CTTTTTGACC CCCGAATTTG GAGTATCCTA CTTTTACGCA ATTCACAGGG

88321 TTCAGCAAGC AATCGATATT TCACGATCAA GGGTGTAATA CTCTTTGTAG TAGCGGTCCT

88381 TATATATCGT ATTAACAGTC GAAATAGGGT CGAAAGACAA AATTTCTATT TGATAGGGCT

88441 TCTTCCTATA CCTATGAATT CCATTGGACC CAGAAATGAT ACATTGGAAG AATCCGTTGG

88501 GTCTTCCAAT ATCAATAGGT TGATTGTTTC CCTCCTGTAT CTTCCAAAAG GAAAAAAGAT

88561 CTCTGAGAGT TGTTTCCTGA ATCCGAAAGA GAGTACTTGG GTTCTCCCAA TAACTAAAAA

88621 GTGTAGCATG CCTGAATCTA ACTGGGGTTC GCGGTGGTGG AGGAACTGGA TCGGAAAAAA

88681 GAGGGATTCT AGCCAATTGA AAGGATCTTC TGATCAATCC AGAGATCCTT TGGATTCCCT

88741 TAGTAATGAG GATTCGGAAT ATCACACATT GATCAATCAA AGAGAGATTC AACAACTAAA

88801 AGAAAGATCG ATTCTTTGGG ATCCTTCCTT TCTTCAAACG GAACGAACAG AGATAGAATC

88861 AGACCGATTC CCGAAAAGCC TTTCTGGATA TTCCTCAATG TCCCGGCTAT TCACGGAACG

88921 TGAGAAGCAG ATGATTAATC ATCTGCTTCC GGAAGAAATC GAAGAATTTC TTGGGAATCC

88981 TACAAGATCC GTTCGTTCTT TTTTCTCTGA CAGATGGTCA GAACTTCATC TGGGTTCGAA

89041 TCCTACTGAG AGGTCCACTA GAGATCAGAA ATGGTTGAAG AAACAACAAG ATCTTTCTTT

89101 TGTCCCTTCG AGGCGGTCGG AAAAGAAAGA AATGGTTAAT ATATTCAAGA TAATTACGTA

89161 TTTACAAAAT ACCGTCTCAA TTCATCCTAT TTCATCATAT CCGGGATGTG ATATGGTTCC

89221 GAAGGATGAA CCGGATATGG ACAGTTCTAA TAAGATTTCA TTCTTGAACA AAAATACATT

89281 TCTTGATTTA TTTCATCTAT TCCATGACCG GAAGAGGGGA GGATACACGT TACACCACGA

89341 TTTTGAATCA GAAGAGAGAT TTCAAGAAAT GGCAGATTTA TTCACTCTAT CAATAACCGA

89401 GCCGGATCTG GTGTATCATA AGGGATTTGC CTTTTCTATT GATTCCTACG GATTGGATCA

89461 AAAACAATTC TTGAATGAGG TATTCAACTC CAGGGATGAA TCGAAAAAAA AATCTTTATT

89521 GGCTCTACCT CCTATTTTTT ATGAAGAGAA TGAATCTTTT TATCGAAGGA TCATAAAAAA

89581 ATGGGTCCGG ATCTCCTGCG GGAATGATTT GGAAGATCCA AAACAAAAAA AAGTGGTATT

89641 TGCTAGCAAC AACATAATGG AGGCAGTCAA TCAATATAGA TTGATCCGAA ATCTGATTCA

89701 AATCCAATAT AGCACCTATG GGTACATAAG AAATGTATTG AATCGATTCT TTTTAATGAA

89761 TAGATCCGAT CGCAACTTCG AATATGGAAT TCAAAGGGAT CAAATAGGAA AGGATACTCT

89821 GAATCATAAA ACTAGAATGA AATATACGAT CAACCAACAT TTATCGAATT TGAAAAAGAG

89881 TCAGAAGAGA TGGTTCGATC CTCTTATTTT GATTTCTCGA ACCGAGAGAT CCATGAATCG

89941 GGATCCTGAT GCATATAGAT ACAAATGGTC CAATGGGAGC AATAATTTCC AGGAACATTT

90001 GGAACATTTC GTTTCTGAGC AGAAGAGCCG TTTTCAAGTA GTGTTCGATC GATTACGTAT

90061 TAATCAATAT TCGATTGATT GGTCTGAGGT TATCGACAAA AAAGGTTTGT CTAAGCCATT

90121 TCGTTTCTTT TTGTCCAAGT TGCTTTTCTT TTTGTCTAAC TCACTTCCTT TTTTCTTTTT

90181 GAGTTTCGGG AATATCCCCA TTCATAGGTC CGAGATCTAC ATCTATGAAT TGAAAGGTCC

90241 GAATGATCAA CTCTGCAATC AGTTGTTAGA ATCAATAGGT CTTCAAATCG TTCATTTGCA

90301 AAAATGGAAA CCCTTCTTAT TGGATGATCA TGATACTTCC CAAAAATCTA AATTCTTGAT

90361 CAATGGAGGA ACAATATCAC CATTTTTGTT CAATAAGATA CCAAAGTGGA TGATTGACTC

90421 ATTCCACACT AGAAAGAATC GTAGGAAATC CTTTGCTAAC ACGGATTCCT ATTTCTCAAC

90481 GATATTCCAC GATCAAGACA ATTGGCTGAA TCCCCTGAAA CCATTTCATA GAAGTTCATT

90541 GATATCTTCT TTTTATAAAG CAAATCGACT TCGATTCTTG AATAATCCAC ATCACTTCTG

90601 CTTCTATTGT AATAAAAGAT TCCCTTTTTA TGTGGAAAAG GCCCGTATCA ATAATGATGA

90661 TTTTACGTAT GGACAATTCC TCAATATCTT GTTCATTCAC AACAAAATTT TTTCTTTGTG

90721 CGTCGGTAAA AAAAAACATG CTTTTTGGGG GAGAGATACT ATTTCACCAA TCGAGTCACA

90781 GGTATCTAAC ATATTCATAC CTAACGATTT TCCACAAGGT GGTGGTGACG AAACGTATAA

90841 CTTGTACAAA TCTTCCCATT TTCCAAGTCG ATCCAATCCA TTCGTTCGTA GAGCTATTTA

90901 CTCGATCGCA GACATTTCTG GAACACCTCT AACAGAGGGA CAGATAGTCA ATTTTGAAAG

90961 AACTTATTGT CAACCTCTTT CAGATCTGAA TCTATCTGAT TCAGAAGGGA AGAACTCGCA

91021 TCAGTATCTC AATTTCAATT CAAACATGGG TTTGATTCAC ACTCCATGTT CTGAGAAATA

91081 TTTACCATCC GAAAAGAGGA AAAAACGGAG TCTTTTTCTA AAGAAATACG TTGAGAAAGG

91141 GCAGATGTAT AGAACTTTTC AACGAGATAG TGCTTTTTCA ACTCTCTCAA AATGGAATCT

91201 ATTCCAAACA TATATACCAT GGTTCCTTAC TTCGACAGGG TACAAATATC TAAATTGGAT

91261 ATTTTTAGAG ACTTTTTCAG ACCTATTGCC GATACACCTA TTGCCGATAC ACCGATTGCC

91321 GATACTAAGT AGCAGTCATA CATTTGTATC CATTTTTCAT GATATTATGC ATGTATTAGA

91381 TATAGCATGG CGAATTCTTC AGCAAAAATG GGGTCTTCCA CAACGGAATC CGATACGTAA

91441 GATTTCGAGT CAGTGTTTAC ATAATCTTCT TCTGTCCGAA GAAATGATTC ATCGAAATAA

91501 TGAGTCGCCA TTGATATCGA CACATCTGAG ATCGCCAAAT GTTCGGGAGT TCCTCTATTC

91561 AATCCTTTTC CTTCTTCTTG TTGCTGGATA TCTCGTTCGT ACACATCTTT TCTTTGTTTC

91621 CCGGGCCTCT AGTGAGTTAC AGACAGAGTT TGAAAAGGTC AAATCTTTGA TGATTCCATC

91681 ATCTATGATT GAGTTGCGAA AACTTCTGGA TAGGTATCCT ACATCTGAAC CGAATTTTTT

91741 CTGGTTAAAG AATCTCTTTC TAGTTGCTCT AGAACAATTA GGAGATTCTC TAGAAGAAAT

91801 ACGGGGTTCG GCTTCTGGCG GCAACATGCT ATTGGGTGGT GATCCCGCTT ATGGGGTCAA

91861 ATCAATACGT TCTAAGAAGA AATATTTGAA TATCAATCTC ATCGATCTCA TAAGTATCAT

91921 ACCAAACCCC ATCAATCGAA TCACTTTTTC GAGAAATACG AGACATCTAA GTCATACAAG

91981 TAAAGAGATC TATTCATTGA TAAGAAAAAG AAAAAATGTG AACGGGGATT GGATTGATGA

92041 TAAAATAGAA TCCTGGGTCG CAAACAGTGA TTCGATTGAT GATGAAGAAA GAGAATTCTT

92101 GGTTCAGTTC TCCACCTTAA CGACAGAAAA AAGGATTGAT CAAATTCTAT TGAGTCTGAC

92161 TCATAGTGAT CATTTATCAA AGAATGACTC TGGCTATCAA ATGATTGAAC AACCGGGAGC

92221 AATTTACTTA CGATACTTAG TTGACATTCA TAAAAAGTCT CTAATGAATT ATGAGTTCAA

92281 TACATCCTGT TTAGCAGAAA GACGGATATT CCTTGCTCAT TATCAGACAA TCACTTATTC

92341 ACAAACCTCG TGTGGGGCTA ATAGTTTTCA TTTCCCATCT CATGGAAAAC CCTTTTCGCT

92401 CCGCTTAGCC TTATCCCCCT CTAGGGGTAT TTTAGTGATA GGTTCTATAG GAACTGGACG

92461 ATCCTATTTG GTCAAATATC TAGCGACAAA CTCCTATGTT CCTTTCATTA CGGTATTTCT

92521 GAACAAGTTC CTGGATAACA AGTCTAAAGG TTTTCTTAGT GATGATATCG ATATTGATGA

92581 TATCGATATT GATGATATCG ATATTGATGC TAGTGAGGAT ATTGATGCTA GTGACGATAT

92641 TGATGCTAGT GACGATATCG ATCGTGACCT TCATACGGAG CTGGAACTGC TAACTACGGA

92701 TATGATGTCG GAAAAAGACC AATTTTCTAT CACCCTTCAA TTCGAATTAG CAAAAGCAAT

92761 GTCTCCTTGC ATAATATGGA TTCCAAACAT TCATGATCTG GATGTGAATG AGTCGAATGA

92821 CTTCTCCCTC GGTCTATTAG TGAACCATCT CTCCAGGGAT TGTGAAAGAT GTTCCACTAG

92881 AAATATTCTT GTTATTGCTT CGACTCATAT TCCCCAAAAA GTGGATCCCG CTCTAATAGC

92941 TCCGAATAAA TTAAATACGT GCATTAAGAT ACGAAGGCTT CTTATTCCAC AACAACGAAA

93001 GCACCTTTTC ACCCTTTCAT ATACTAGGGG ATTTCGCTTG GAAAAGAAAA TGTTCCATAC

93061 TAATGGATTC GGGTCCATAA CCATGGGTTC CAATGCACGA GATCTTGTAG CACTTACCAA

93121 TGAGGCCCTA TCGATTAGTA TTACACAGAA GAAATCAATT ATAGACACTA ATACAATTAG

93181 ATCCGCTCTT CATAGACAAA CTTGGGATTT GCGATCCCAG GTAAGATCGG TTCAGGATCA

93241 TGGGATCCTT TTCTATCAGA TAGGAAGGGC TGTAGCACAA AATGTACTTC TAAGTAATTG

93301 CCCCATAGAT CCTATATCTA TCTATATTAA GAAGAAATCA TGTAACGAAG GGGATTCCTA

93361 TTTGTACAAA TGGTACTTCG AACTTGGAAC GAGCATGAAG AAATTAACGA TACTTCTTTA

93421 TCTTTTGAGT TGTTCTGCCG GATTGGTCGC TCAAGATCTT TGGTCTCTAC CCGGACCCGA

93481 TGAAAAAAAT GGGATCACTT CTTATGGACT CGTTGAGAAT GATTCTGATC TAGTTCATGG

93541 CCTATTAGAA GTAGAAGGCG CTCTGGTGGG ATCCTCGCGG ACAGAAAGAG ATTGCAGTCA

93601 GTTTGATAAT GATCGGGTGA CATTGCTTCT TCGGCCCGAA CCAAGGAATC CCCTAGATAT

93661 GATGCAAAAC GGATCTTGTT CTATCCTTGA TCAGAGATTT CTCTATGAAA AAAACGAATC

93721 AGGGTTTGAA GAAGGGGAAG GGGCCCTCGA CCCGCAACAG ATAGAGGAGG ATTTATTTAA

93781 TCACATAGTT TGGGCTCCTA GAATATGGCA CCCTTGGGGC ATTCTATTTG ATTGTATCGA

93841 AAGGCCCAAT GGATTGGGAT TTCCCTATTG GTCCAGGTCA TTTCGGGGCA AGCGGATCCT

93901 TTATGATGAA GAGGATGAGC TTCAAGAGAA TGATTCGGAG TTCTTGCAGA GTGGAACCAT

93961 GCAGTCCCAG ACACGAGATA GATCTTCCAA AGAACAAGGC CTTTTTCGAA TAAGCCAATT

94021 CATTTGGGAC CCTGCAGATC CACTCTTTTT CCTATTCAAA GATCAGCCCT TTGTCTCTGT

94081 GTTTTCACAT CGAGAATTAT TTGCAGATGA AGAGATGTCA AAGGGGCTTC TTACTTCCCA

94141 AACAGATCCT CCTACATCTA TATATAAACG CTGGTTTATC AAGAAGACGC AAGAAGAGCA

94201 CTTCGAATTG TTGATTAATC GCCAGAGAGG GTTTAGAACC ACTAGTTCAT TATCTAATGG

94261 ATCTTTCCGT TCTAATACTC TATCCGAGAG TTATCAGTAT TTATCAAATC TATTCCTATC

94321 TAACGGAACG CTGTTGGATC AAATGACAAA GACATTGTTA AGAAAAAGAT GGCTTTTCCC

94381 GGATGAAATG AAAATTGGAT TCATGGAACA GGAGAAAGAT TTACCATTCC TTAGGCGGAA

94441 AGGTATGTGG CCATGAAAGA AAGAGGGATT AAGTGGAACA GAATTGACTG GGTGGTAGAG

94501 TCGCGGAAAC GCTTGTTTCT TCCCTATTTT GGACCTTAGC TCCATGGAAC AATAGGCTGC

94561 TGCTGAAACA CGGAAGAATT GAAATCTTAG ATCAAAACAC TATGTATGGA TGGTATGAAC

94621 TGCCTAAACA AGAATTCTTG AACAGCGAAC AACCAGTTCA TATATTCACG ACCAAGAAAA

94681 GAAGTACTGG ATTCTCTAAA GAAGTACTGG ATTCTCTTTC GGATAGGCCC TGAAAGCGAA

94741 GGAAGGCTGG AATGCCACCA GGCGTCTATT ATTGAATTCA CCCGACCCGA CAGTACCCAT

94801 TTTGGGAACG TCCAGTGCCA AAGTCACTGA ATGGGTAAAT CGCCAATCCC TAAAACGGAC

94861 TATGTAATGT ACTTTATCTG CTGGGTTACG GGCGGGCATT TTACCAGAGG TTTCTAATCT

94921 ACCCTTGTGT GATTACTGTT GAAGCATATA CTCGGGGGGG TGGGTGCAGG GCGGACGATT

94981 TTAAAGCAGA CTCCCCATTC ATTAGATAGA GAAGATCACC AAGATTTAGT GATCCGCTGC

95041 CGAGCTTATG GAATTGCCCA TTCAATGAGC ATTCTCAATA TTATGCCTTG AAGAGGACTC

95101 GAACCTCCAC GCTCTTTAGC ACGAGATTTT GAGTCTCGCG TGTCTACCAT TTCACCACCA

95161 AGGCATCTTG AAAGTGAATC GTATTCCATG AATATGATAT CTATCTAGTG TGATGTATGG

95221 AATATATGAC AAGGGTGGAG TATTTCTATT GATTGGTCAT GTTATATAGG CCCGAGTTGG

95281 ATATCCAATT GTTTCGATTT GAATTATCCG GAGAATGCCT TATATAGATA TCCAATCTAT

95341 CAAAAAGATG GACACAATCA AACCCATTTC TCGATTCAAT AGAAGCCCAA AGAGATGAAT

95401 AGGGTCCCAA ATAACGAGAG ATATGTAAAA AGCAGGTCCG ATTGCTACGC CTATTCCTAA

95461 TCCTAACTGT AATGTAACGA CGTAGGGATC CATATGTAAA CATAGTATCT ATTTAGATAC

95521 GCTCAAATGA CCCCTTCTCA TAATGAGAAT GTATATAACC CTATTCCGGT CTGGTCCGGT

95581 ATGGAATGAA CTTATAATCA TGGAATCGAC TCGATCATCA GATTATAGAT TATAAGTTCA

95641 TAACCCTAGC CCATTCCCAT TTTGGGCGGA ACAGATCTAC TAATTCTTTG ATTCCAGTTA

95701 GTAAGAGGGA TCTTGAACTA AGAAATAGAC CCTAGAAGCT AAAAAAGGGT ATCCTGAGCA

95761 ATTGCAATAA TCGGGTTCAT TGAGATTCCT GGTATAGTAG ATGCTATCAC ACATACAATC

95821 ATACTCAATT CGATGGAATT GTTTGATCTG AAAGGAGATC TTCTATAATT TCGCACGTGA

95881 GGGGTTATTT CTTGGGTTCG TCCAGTCATT AATAACTTGA TTATTTTTAG ATAATAGTAG

95941 ATAGAAACAA CGCTTGTAAG GAGTCCTATT AAAACCAAGA AATATAGGCC TGCCTGCCAC

96001 CCACACCAGA ATAAATAGAG TTTTCCGAAA AAACCTGCTA GTGGAGGAAG ACCTCCTAGG

96061 GATAAGAGAC ATAGGGCTAA AGAGAGAGCC AAAAAAGGAT CTTTTGTATA TAATCCTGCA

96121 TAATCTCGAA TGTTATCAGT TCCGGTACGT AGACCAAATA ATACAATGCA AGCAAAAGTT

96181 CCTAGATTCA TGGAGATATA GAACAGCATA TAAGTTATCA TGCTTGCATA TCCATCATTT

96241 GAGTCTCCAA CAATTATTCC AATAATTACA TATCCGATTT GACCTATGGA CGAATATGCA

96301 AGCATACGTT TCATGCTTGT TTGAGTAATA GCAATGAGAT TCCCCAATAT CATGCTAAGA

96361 ATAGCTAGTG TTTCCAGAAG AAGATGCCAT TCGTTTGATG AGAAATAAAA AGGAATATCG

96421 AAAATTCGAG TGGCTGAAGC TGGAGCAGCT ACTTTCGAAG TAACAGAAAG AAAAGCAACG

96481 ACTGGAGTGG GAGAGTCAGA GTCGAAAAGA GGATTCCTCA CTTCTTTCTC TCATTCAAAA

96541 CCGTGCATGA GACTTTCATC TCACACGGCT CCTAAGTGAT CAAAAAAGAA AGAAGAACTC

96601 ATCTTCTTTC TTTTTGATT ACCTTCCTCG CGTATGTATA AGACCGAATC CATTCGATTT

96661 CTAAAAAAGA TTACTAATCC TTAACTTTTC GAGGAATCCT TCATCAGTGG TTGTGAATGA

96721 CTTGTTTTTT CAATCTTTTC GATCTTGGTT CCGTAGGAGG AGCAAATCAG AAAGATTGAG

96781 AAATAGAACC ATCTGATTTG ATTCGTTCTC AATAGCCATG AGATGATCAT CTTAGGGTGA

96841 TCCTTTTGTC GACGGATGCT CCTATTATAC TCGTAGTCTC TGAAGGATGA GAACCAACTA

96901 TGTAGCATCT ACACCGAGAA TTCAAGTATT GTATACGTCA TTGGTCCGAT CCTTTGTAGG

96961 AACTACCCAT AATAACGAAC TTGCAAAATG GATCTGTTTA TCATAAAGAG ATTCGTCGTT

97021 CCTGACCCTG CTTCGCCTTA ATTGTTATTT GAACAAGTAA AAGTTCTGTC TTGGTCCGAG

97081 TGTGGATAGC ATTTCTCTTC TGCATGTCCA TGGAGTTTTG AAAAATCCAA ACATCTCAGA

97141 GATAGATAGA GAGGTAGGAA TTTATCGAAC GAACCGCACT CCTTCGTATA CGTCAGGAGT

97201 CCATTGATGA GAAGGGGCTG GGGAAAGCTT GAACCCAATT CCTACAGTGA TGAATATGAG

97261 CGCAATTGAA ATTCCTGGGG AGTTATACAT TTGTGTATTG ATAAGACCAT TCACTATTTC

97321 TTGAAGCTCG ATCTCTCCCC CGGATAAACC ATATAGCCAA GAGAAACCAT GAACCAGAAT

97381 AGAAGAGCTT GCCCCACCCA TGAGTAAATA TTTCATAGTA GCCTCATTAG ACCGTACATC

97441 TTTCTTGGTA TATCCAGATA ATAGGTAGGA GCATAAACTG AAACATTCTG GAGCTACAAA

97501 GATAGTTATT AAATCGTTAG CACCGCATAA AAACATTCCT CCTAGAGTAG CTGTTAATAC

97561 GAATAAGAGA AACTCTGTTA TAGCCATTTC TGTACATTCG ATGTACTCTA CGGATAGAGG

97621 AATACATAGA GTTGAACATA GTAAAATAAG AAATTGAAAG ATTTCGTTGA AATTGTTCGT

97681 TTGGAAATTT CCCGAAAAAC TAATCATAGG TTCTTCTCTC CATCGGAACA AGAGGGCCGT

97741 TATGCTCATT ACTAAACTTG TTGAAGAGAT GAAATATAAC CAAGGTATAT CTTTTTGATC

97801 AGAGGTTGAA TCGATCATCA GAAGAAGAAT TAGGCCAAAA ATTAGGATAC ATTCTGGGAA

97861 AATCAAACTT CCATCGAAGA GAAGCAAATG AAAGGCTTTC ATAAAAATTC TCGTAGAATC

97921 GAGAATGAAG TTTTCATTCT GTACATGCCA GATCATGAAT TAGTAACTGC ATCCAATTTC

97981 AAAAAAAAAA TCCCAATTGT TTCGAACTTT CCATTTTTAG AATGGAATAT TTACGGAATC

98041 TCCATGAATA GGACCAAACC TTATTCCATG GTATTTACAT GAGGTTCCTC TTTCTTATTC

98101 TTAAGAAAGT CCCCGAGAGG GCTTAGTTGA TCCATGATTT CTGTTTCATC TTTCGTTTCC

98161 TTTTCATTTG TTTCGAGAAA TATATCGATC AATTCCGATT CTTTCTTTTT CTCTTGATTC

98221 TTTTCCGATC GAGATGTATA GATCCTGTTC ATGGATTAAC GAAAATGTGC AAAAGCTCTA

98281 TTTGCCTCTG CCATTCTATG AGTCTCTTCC TTTTTGCGTA TGGCATCGCC ACTCCCTTTG

98341 GCAGCATCCA CTAATTCGGA ACTTAATTTG AAAGCCATAT TTCGACCCGG ACGTTTTCGG

98401 GATGCCGCTA ATAACCAACG AATGGCAAGT GCTTTTCCTT GTGTGGATCC TATTTCAATG

98461 GGAACTTGAT GAGTCGATCC ACCTACACGT CTTGCTTTTA CTGCTATATC GGGAGTTACT

98521 CCACGTATTG CTTGACGTAA AACAGATAGT GGATTTGTTT CTGTCTTTTG TTGAATCTTT

98581 TTCACGGCTC GATAGATAAT TTGATAAGCC AATGATTTTT TTCCGTGTTT CAGAATACGG

98641 TTAACCAACA TGTTAACTAA TCGATTACGA TAAATTGGAT CGGATTTTGC AGTTTTTTCT

98701 TCTGCAGTAC CTCGACGTGA CATGAGCGTG AAGGGGGTTC AAGAATCAGT TTTCTTTTTT

98761 TATAAGGGCT AAAATCACTT ATTTTGGCTT TTTTACTCCA TATTGTAGGG TGGATCTCGA

98821 AAGATATGAA AGATCTCCCT CCAAGCCGTA CATACGACTT TCATCGAATA CGGCTTTCCG

98881 CAGAATTCTA TATGTATCTA TGAGATCGAG TATGGAATTC TGTTTACTCA CTTTAAATTG

98941 AGTATCCGTT TCCCTCCTTT TCCTGCTAGG ATTGGAAATC CTGTATTTTA CATATCCATA

99001 CGATTGAGTC CTTGGGTTTC CGAAATAGTG TAAAAAGAAG TGCTTCGAAT CATTGCTATT

99061 TGACTCGGAC CTGTTCTAAA AAAGTCGAGG TATTTCGAAT TGTTTGTTGA CACGGACAAA

99121 GTCAGGGAAA ACCTCTGAAA TTATTTCAAT ATTGAACCTT GGACATCTAA GAGTTCCGAA

99181 TCGAATCTCT TTAGAAAGAA GATCTTTTGT CTCATGGTAG CCTGCTCCAG TCCCCTTACG

99241 AAACTTTCGT TATTGGGTTA GCCATACACT TCACATGTTT CTAGCGATTC ACATGGCATC

99301 ATCAAATGAT ACAAGTCTTG GATAAGAATC TACAACGCAC TAGAACGCCC TTGTTGACGA

99361 TCCTTTACTC CGACAGCATC TAGGGTTCCT CGAACAATGT GATATCTCAC ACCGGGTAAA

99421 TCCTTAACCC TTCCCCCTCT TACTAAGACT ACAGAATGTT CTTGTGAATT ATGGCCAATA

99481 CCGGGTATAT AAGCAGTGAT TTCAAATCCA GAGGTTAATC GTACTCTGGC AACTTTACGT

99541 AGGGCAGAGT TTGGTTTTTT GGGGGTGATA GTGGAAAAGT TGACAGATAA GTCACCCTTA

99601 CTGCCACTCT ACGGAAACGT ACATGAGATT TTCACCTCAT ACGGCTCCTC GTTCAATTCT

99661 TTCGAAGTCA TTGGATCCTT TTCCGCGTTC GAGAATCCCC TCCCCCTTTC CACTCCGTCC

99721 CGAAGAGTAA CTAGGACCAA TTGAGTCACG TTTTCATGTT CCAATTGAAC ACTTTCCTTT

99781 TTTGATTATT CTCAAACTCA AAGGAGAAGA TTATTCTCTT TACCAAACAT ATGCGTATCC

99841 AATCACGATC TTATAATAAG AACAAGAGAT CTTTCTCGAT CAATCCAATC CCTTTGCCCC

99901 TCATTCTTCG AGAATCAGAA AGATCCTTTT CAAGTTTGAA TTTGTTCATT TGGAATCTGG

99961 GTTCTTCTAT ATTTTTTTTA CTTAGTTTAC TTATTTTCTT TTTTTTTATT ATTGAATATT

100021 TTTCCCTCTC TTTTTTTATA TCATTCCTTA AGTCCCATAG GTTTGATCCT TTAGAATTTG

100081 ACCCATTTTC TCATTGAGCG AAGGGTACGA AATAAATCAG ATTTCTTTTT TGATCAAAAA

100141 TACTATGTGA AATCTTCTGT TTTTTCCTCT TCCTATATCC CATAGGTACA GTGTTTGAAT

100201 CAATAGAGAA CCTTTTCTTC TGTATGAATC GATATTATTC CATTCGAATT CCTTCCCGAT

100261 ACCTCCCGAG GAAAATTTTG AATTGGATCC CAAATTGACG GGTTAGTGTG AGCTTATCCA

100321 TGCGGTTATG CACTTTTCGA ATAGGAATCC ATTTTCTGAA AGATCCTGGC TTTCGTGCTT

100381 TGGTGGGTCT CCGAGATCCT TTCGATGACC TATGTTGTGT TGAAGGGATA TCTATATAAT

100441 CCGATCGATT GCGTAAAGCC CGCGGTAGCA ACGGAACCGG GGAAAGTATA CAGAAAAGAC

100501 AGTTCTTTTC TATTATATTA GTATTTTCTA GTATTTCTAT TATATTAGAA TATTAGATAT

100561 TAGATTAGTA TTAGTTAGTG TTAGATTAGT ATTAGTTAGT GATCCCGGCT TAGTGAGTCC

100621 TTTCTTTCGT GATGAGCTGT TGGCGCCAGT CTTACATTTT GTATCTGTGG ACCGAGGAGA

100681 AAGGGGGCTC GGCGGGAAGA GGATTGTACC GTGAGAGAAG CAAGGAGGTC AACCTCTTTC

100741 AAATATACAA CATGGATTCT GGCAATGCAA TGTAGTTGGA CTCTCATGTC GATCCGAATG

100801 AATCATTCTT TCCACGGAGG TAAATCTTTG CCTGCTAGGC AGGAGGATAG CAAGTTACAA

100861 ATTCTGTCTC GGTAGGACAT GTATTTCTAT TACTATGAAA TTCATAAATG AAGTAGTTAA

100921 TGGTGGGGTT ACCGTTATCC TTTTTGTGAC GAATCTTGTA TGTGTTCCTA AGAAAAGGAA

100981 TTTGTCCATT TTTCGGGGTC TCAAGGGGGC GTGGAAACAC ATAAGAACTC TTGAATGGAA

101041 ATGGAAAAGA GATGGAACTC CAGTTCCTTC GGAAATGGTA AGATCTTTGG CGCAAGAAGA

101101 AGGGGTTGAT CCGTATCATC TTGACTTGTT TCTGCTTCCT CTATTTTTTT AATAATACCG

101161 GGTCGGGTTC TTCTCCGACC CATATCGAAT AGAACATGCT GAGCCAAACC TTCTTCATGT

101221 AAAACCTGCT TGATTTAGAT CGGGAAAGTC GTGTGGTTTT ATGAAACCAT GTGCTATGGC

101281 TCGAATCCGT AGTCAATCCT ATTTCCGATA GGGACAGTTG ACAACGGAAT CCGACTTTTC

101341 CCATAATTTG GATATCCGTA ATAGTGCGAA AAGAAGGCCC GGCTCCAAGT TGTTCAAGAA

101401 TAGTGGCGTT GAGTTTCTCG ACCCTTTGCC TTAGGATTAG TCAGTTCTAT TTCTCGATGG

101461 GGGCAGGGAA GGGATATAAC TCAGCGGTAG AGTGTCACCT TGACGTGGTG GAAGTCATCA

101521 GTTCGAGCCT GATTATCCCT AAACCCAATG TGAGTTTTTC GACTTTGATT TGCTCCCCCG

101581 CCGTGATTGA ACGAGAATGG ATAAGAGGCT CGTGGGATTG ACGTGAGGGG GCAGGGATGG

101641 CTATATTTCT GGGAGCGAAC TCCGGGCGAA TATGAAGCGC ATGGATACAA GTTAGGCCTT

101701 GGAATGAAAG AAAATTCCGA ATCCGCTTTG TCTACGAACA AGGAAGCTAT AAGTAATGCA

101761 ACTATGAATC TCATGGAGAG TTCGATCCTG GCTCAGGATG AACGCTGGCG GCATGCTTAA

101821 CACATGCAAG TCGGACGGGA AGTGGTGTTT CCAGTGGCGG ACGGGTGAGT AACGCGTAAG

101881 AACCTGCCCT TGGGAGGGGA ACAACAGCTG GAAACGGCTG CTAATACCCC GTAGGCTGAG

101941 GAGCAAAAGG AGGAATCCGC CCGAGGAGGG GCTCGCGTCT GATTAGCTAG TTGGTGAGGC

102001 AATAGCTTAC CAAGGCGATG ATCAGTAGCT GGTCCGAGAG GATGATCAGC CACACTGGGA

102061 CTGAGACACG GCCCAGACTC CTACGGGAGG CAGCAGTGGG GAATTTTCCG CAATGGGCGA

102121 AAGCCTGACG GAGCAATGCC GCGTGGAGGT AGAAGGCCCA CGGGTCGTGA ACTTCTTTTC

102181 CCGGAGAAGA AGCAATGACG GTATCTGGGG AATAAGCATC GGCTAACTCT GTGCCAGCAG

102241 CCGCGGTAAT ACAGAGGATG CAAGCGTTAT CCGGAATGAT TGGGCGTAAA GCGTCTGTAG

102301 GTGGCTTTTT AAGTCCGCCG TCAAATCCCA GGGCTCAACC CTGGACAGGC GGTGGAAACT

102361 ACCAAGCTGG AGTACGGTAG GGGCAGAGGG AATTTCCGGT GGAGCGGTGA AATGCGTAGA

102421 GATCGGAAAG AACACCAACG GCGAAAGCAC TCTGCTGGGC CGACACTGAC ACTGAGAGAC

102481 GAAAGCTAGG GGAGCGAATG GGATTAGATA CCCCAGTAGT CCTAGCCGTA AACGATGGAT

102541 ACTAGGCGCT GTGCGTATCG ACCCGTGCAG TGCTGTAGCT AACGCGTTAA GTATCCCGCC

102601 TGGGGAGTAC GTTCGCAAGA ATGAAACTCA AAGGAATTGA CGGGGGCCCG CACAAGCGGT

102661 GGAGCATGTG GTTTAATTCG ATGCAAAGCG AAGAACCTTA CCAGGGCTTG ACATGCCGCG

102721 AATCCTCTTG AAAGAGAGGG GTGCCTTCGG GAACGCGGAC ACAGGTGGTG CATGGCTGTC

102781 GTCAGCTCGT GCCGTAAGGT GTTGGGTTAA GTCCCGCAAC GAGCGCAACC CTCGTGTTTA

102841 GTTGCCATCG TTGAGTTTGG AACCCTGAAC AGACTGCCGG TGATAAGCCG GAGGAAGGTG

102901 AGGATGACGT CAAGTCATCA TGCCCCTTAT GCCCTGGGCG ACACACGTGC TACAATGGCC

102961 GGGACAAAGG GTCGCGATCC CGCGAGGGTG AGCTAACTCC AAAAACCCGT CCTCAGTTCG

103021 GATTGCAGGC TGCAACTCGC CTGCATGAAG CCGGAATCGC TAGTAATCGC CGGTCAGCCA

103081 TACGGCGGTG AATTCGTTCC CGGGCCTTGT ACACACCGCC CGTCACACTA TGGGAGCTGG

103141 CCATGCCCGA AGTCGTTACC TTAACCGCAA GGAGGGGGAT GCCGAAGGCA GGGCTAGTGA

103201 CTGGAGTGAA GTCGTAACAA GGTAGCCGTA CTGGAAGGTG CGGCTGGATC ACCTCCTTTT

103261 CAGGGAGAGC TAATGTTTAT TGGGTATTTT GGTTTGACAC TGCTTCACAC CCAAAAAGAA

103321 GGGAGCTAGG TCTGAGTGAA ACTTGGAGAT GGAAGTCTTC TTTCGTTTCT CGACGGTGAA

103381 GTAAGACCAA GCTCATGAGC TTATTATCCC AGGTCGGAAC AAGTTGATAG GATCCCCTTT

103441 TTTACGTCCC CATGTCCCCC CGTGTGGCGA TATGAGGGCG AAAAAAGgaa aGAGAGaGGg

103501 ATGGGGTTTC TCTCGCTTCT GGCATAGCGG GCCCCCAGTG GGAGGCTCGC ACGACGGGCT

103561 ATTAGCTCAG TGGTAGAGCG CGCCCCTGAT AATTGCGTCG TTGTGCCTGG GCTGTGAGGG

103621 CTCTCAGCCA CATGGATAGT TCAATGTGCT CATCGGCGCC TGACCCTGAG ATGTGGATCA

103681 TCCAAGGCAC ATTAGCATGG CGTACTCCTC CTGTTCGAAC CGGGGTTTGA AACCAAACTT

103741 CTCCTCAGGA GGATAGATGG GGCGATTCAG GTGAGATCCA ATGTAGATCC AACTTTCGAT

103801 TCACTCGTGG GATCCGGGCG GTCCGGGGGG GACCACCATG GCTCCTCTCT TCTCGAGAAT

103861 CCATACATCC CTTATCAGTG TATGGACAGC TATCTCTCGA GCACAGGTTT AGGTTCGGCC

103921 TCAATGGGAA AATAAAATGG AGCACCTAAC AACGCATCTT CACAGACCAA GAACTACGAG

103981 ATCACCCCTT TCATTCTGGG GTGACGGAGG GATCATACCA TTCGAGCCGT TTTTTTTCAT

104041 GCTTTTCCCG GAGGTCTGGA GAAAGCTGCA ATCAAAAGGA TCTTAGAGTG TCTAGGGTTG

104101 GGCCAGGAGG GTCTCTTAAC GCCTTCTTTT TTCTTCTCAT CGGAGTTCTT TCACAAAGAC

104161 TTGCCATGGT AAGGAAGAAG GGGGGAACAA GCACACTTGG AGAGCGCAGT ACAACGGAGA

104221 GTTGTATGCT GCGTTCGGGA AGGATGAATC GCTCCCGAAA AGGAATCTAT TGATTCTCTC

104281 CCAATTGGTT GGACCGTAGG TGCGATGATT TACTTCACGG GCGAGGTCTC TGGTTCAAGT

104341 CCAGGATGGC CCAGCTGCGC CAGGGAAAAG AATAGAAGAA GCATCTGACT CCTTCATGCA

104401 TGCTCCGCTT GGCTCGGGGG GATATAGCTC AGTTGGTAGA GCTCCGCTCT TGCAATTGGG

104461 TCGTTGCGAT TACGGGTTGG ATGTCTAATT GTCCAGGCGG TAATGATAGT ATCTTGTACC

104521 TGAACCGGTG GCTCACTTTT TCTAAGTAAT GGGGAAGAGG ACCGAAACAT GCCACTGAAA

104581 GACTCTACTG AGACAAAGAC GGGCTGTCAA GAACGTAGAG GAGGTAGGAT GGGCAGTTGG

104641 TCAGATCTAG TATGGATCGT ACATGGACGG TAGTTGGAGT CGGCGGCTCT CCTAGGGTTC

104701 CCTAATCTGG GATCTCTGGG GAAGAGGATC AAGTTGGCCT TGCGAACAGC TTGATGCACT

104761 ATCTCCCTTC AACCCTTTGA GCGAAATGCG GAAAAAGGAA GGAAAATCCA TGGACCGACC

104821 CCATCATCTC CACCCCGTAG GAACTACGAG ATCACCCCAA GGACGCCTTC GGCATCCAGG

104881 GGTCACGGAC CGACCATAGA ACCCTGTTCA ATAAGTGGAA CGCATTAGCT GTCCGCTCTC

104941 AGGTTGGGCA GTAAGGGTCG GAGAAGGGCA ATCACTCATT CTTAAAACCT GCGTTCGTAA

105001 GACCAAAGAG TCGGCGGAAA GGGGGGGAAA GCTCTCCGTT CCCGGTTCTC CTGTAGCTGG

105061 ATCCTCCGGA ACCACAAGAA TCCTTAGTTA GAATGGGATT CCAACTCAGC ACCTTTTGGG

105121 TGAGATTTTG AGAAGAGTTG CTCTTTGGAG AGCACAGTAC GATGAAAGTT GTAAGCTGTG

105181 TTCGGGGGGG AGTTATTGTC TATCATTGGC CTCTATGGTA GAATCAGTCG GGGGACCTGA

105241 GAGGCGGTGG TTTACCCTGC GGCGGATGTC AGCGGTTCGA GTCCGCTTAT CTCCAACTCG

105301 TGAACTTAGC CGATACAAAG CTATATGATA GCACCCAATT TTTCCGATTC GGCGGTTCGA

105361 TCTATGATTT ATCATTCATG GACGTTGATA AGATCCATCC ATTTAGCAGC ACCTTAGGAT

105421 GGCATAGACT TAAAGTTAAG GGCGAGGTTC AAACGAGGAA AGGCTTACGG TGGATACCTA

105481 GGCACCCAGA GACGAGGAAG GGCGTAGTAA GCGACGAAAT GCTTCGGGGA GTTGAAAATA

105541 AGCATAGATC CGGAGATTCC CGAATAGGTC AACCTTTCAA ACTGCTGCTG AATCCATGGG

105601 CAGGCAAGAG ACAACCTGGT GAACTGAAAC ATCTTAGTAG CCAGAGGAAA AGAAAGCAAA

105661 AGCGATTCCC GTAGTAGCGG CGAGCGAAAT GGGAGCAGCC TAAACCGTGA AAACGGGGTT

105721 GTGGGAGAGC AATACAAGCG TCGTGCTGCT AGGCGAAGCG GTCGAGTACT GCACCCTAGA

105781 TGGCGAGAGT CCAGTAGCCG AAAGCATCAC TAGCTTACGC TCTGACCCGA GTAGCATGGG

105841 GCACGTGGAA TCCCGTGTGA ATCAGCAAGG ACCACCTTGC AAGGCTAAAT ACTCCTGGGT

105901 GACCGATAGC GAAGTAGTAC CGTGAGGGAA GGGTGAAAAG AACCCCCATC GGGGAGTGAA

105961 ATAGAACATG AAACCGTAAG CTCCCAAGCA GTGGGAGGAG CCCGGGGCTC TGACCGCGTG

106021 CCTGTTGAAG AATGAGCCGG CGACTCATAG GCAGTGGCTT GGTTAAGGGA ACCCACCGGA

106081 GCCGTAGCGA AAGCGAGTCT TCATAGGGCA ATTGTCACTG CTTATGGACC CGAACCTGGG

106141 TGATCTATCC ATGACCAGGA TGAAGCTTGG GTGAAACTAA GTGGAGGTCC GAACCGACTG

106201 ATGTTGAAGA ATCAGCGGAT GAGTTGTGGT TAGGGGTGAA ATGCCACTCG AACCCAGAGC

106261 TAGCTGGTTC TCCCCGAAAT GCGTTGAGGC GCAGCAGTTG ACTGGACATC TAGGGGTAAA

106321 GCACTGTTTC GGTGCGGGCC GCGAGAGCGG TACCAAATCG AGGCAAACTC TGAATACTAG

106381 ATATGACCTC AAAATAACAG GGGTCAAGGT CGGCCAGTGA GACGATGGGG GATAAGCTTC

106441 ATCGTCGAGA GGGAAACAGC CCGGATCACC AGCTAAGGCC CCTAAATGAC CGCTCAGTGA

106501 TAAAGGAGGT AGGGGTGCAG AGACAGCCAG GAGGTTTGCC TAGAAGCAGC CACCCTTGAA

106561 AGAGTGCGTA ATAGCTCACT GATCGAGCGC TCTTGCGCCG AAGATGAACG GGGCTAAGCG

106621 ATCTGCCGAA GCTGTGGGAT GTAAAAATAC ATCGGTAGGG GAGCGTTCCG CCTTAGAGGG

106681 AAGCACCCGC GCGAGCGGTA GTGGACGAAG CGGAAGCGAG AATGTCGGCT TGAGTAACGC

106741 AAACATTGGT GAGAATCCAA TGCCCCGAAA ACCTAAGGGT TCCTCCGCAA GGTTCGTCCA

106801 CGGAGGGTGA GTCAGGGCCT AAGATCAGGC CGAAAGGCGT AGTCGATGGA CAACAGGTGA

106861 ATATTCCTGT ACTACCCCTT GTTGGTCCCG AGGGACGGAG GAGGCTAGGT TAGCCGAAAG

106921 ATGGTTATCG GTTCAAGGAC GTAAGGTGAC CCTGCTTTTT CAGGGTAAGA AGGGGTAGAG

106981 AAAATGCCTC GAGCCAATGT TCGAGTACCA GGCGCTACGG CGCTGAAGTA ACCCATGCCA

107041 TACTCCCAGG AAAAGCTCGA ACGACCTTCA ACAAAAGGGT ACCTGTACCC GAAACCGACA

107101 CAGGTAGGTA GGTAGAGAAT ACCTAGGGGC GCGAGACAAC TCTCTCTAAG GAACTCGGCA

107161 AAATAGCCCC GTAACTTCGG GAGAAGGGGT GCCTCCTCAC AAAGGGGGTC GCAGTGACCA

107221 GGCCCGGGCG ACTGTTTACC AAAAACACAG GTCTCCGCAA AGTCGTAAGA CCATGTATGG

107281 GGGCTGACGC CTGCCCAGTG CCGGAAGGTC AAGGAAGTTG GTGACCTGAT GACAGGGGAG

107341 CCGGCGACCG AAGCCCCGGT GAACGGCGGC CGTAACTATA ACGGTCCTAA GGTAGCGAAA

107401 TTCCTTGTCG GGTAAGTTCC GACCCGCACG AAAGGCGTAA CGATCTGGGC ACTGTCTCGG

107461 AGAGAGGCTC GGTGAAATAG ACATGTCTGT GAAGATGCGG ACTACCTGCA CCTGGACAGA

107521 AAGACCCTAT GAAGCTTCAC TGTTCCCTGG GATTGGCTTT GGGCCTTTCC TGCGCAGCTT

107581 AGGTGGAAGG CGAAGAAGGC CTCCTTCCGG GGGGGCCCGA GCCATCAGTG AGATACCACT

107641 CTGGAAGAGC TAGAATTCTA ACCTTGTGTC AGGACCTACG GGCCAAGGGA CAGTCTCAGG

107701 TAGACAGTTT CTATGGGGCG TAGGCCTCCC AAAAGGTAAC GGAGGCGTGC AAAGGTTTCC

107761 TCGGGCCGGA CGGAGATTGG CCCTCGAGTG CAAAGGCAGA AGGGAGCTTG ACTGCAAGAC

107821 CTACCCGTCG AGCAGGGACG AAAGTCGGCC TTAGTGATCC GACGGTGCCG AGTGGAAGGG

107881 CCGTCGCTCA ACGGATAAAA GTTACTCTAG GGATAACAGG CTGATCTTCC CCAAGAGCTC

107941 ACATCGACGG GAAGGTTTGG CACCTCGATG TCGGCTCTTC GCCACCTGGG GCTGTAGTAT

108001 GTTCCAAGGG TTGGGCTGTT CGCCCATTAA AGCGGTACGT GAGCTGGGTT CAGAACGTCG

108061 TGAGACAGTT CGGTCCATAT CCGGTGTGGG CGTTAGAGCA TTGAGAGGAC CTTTCCCTAG

108121 TACGAGAGGA CCGGGAAGGA CGCACCTCTG GTGTACCAGT TATCGTGCCC ACGGTAAACG

108181 CTGGGTAGCC AAGTGCGGAG CGGATAACTG CTGAAAGCAT CTAAGTAGTA AGCCCACCCC

108241 AAGATGAGTG CTCTCCTATT CCGACTTCCC CAGAGCCTCC GGTAGCACAG CCAAGATAGC

108301 GACGGGTTCT CTGCCCCTGC GGGGATGGAG CGACAGAAGT TTTGAGAATT CAAGAGAAGG

108361 TCACGGCGAG ACGAGCCGTT TATCATTACG ATAGGTGTCA AGTGGAAGTA CAGTGATGTA

108421 TGCAGCTGAG GCATCCTAAC AGACCGATAG ACTTGAACCT TGTTCCTACA TGACCCGATC

108481 AATTCGATCA GGCACTCGCC ATCTATTTTC ATTGTTCAAC TCTTTGACAA CACGAAAAAA

108541 CCATTGTTCA ACTCTTTGAC AACATGAAAA AACCAAAAGC TCTGCCCTCT CTATCTATCC

108601 AAGGGATGGA AGGGCAGAGG CCTTTGGTGT CCCCTCCAGT CAAGAATTGG GGCCTCACAA

108661 TCACTAGCCA ATATGCTTTT CTCTCATGCC TTTCTTCGTT CATGGTTCGA TATTCTGGTG

108721 TCCTAGGCGT AGAGGAACCA CACCAATCCA TCCCGAACTT GGTAGTTAAA CTCTACTGCG

108781 GTGACGATAC TGTAGGGGAG GTCCTGCGGA AAAATAGCTC GACGCCAGGA TGATAAAAAG

108841 CTTAACACCT CCCATTCTTA TTTCTATTTC ATATTGAAAA AGAAATAAAT GAAAAAGAAA

108901 AAGGTCGTCT TATTCAAAAC CCCAATTAAT TATGACATCC CTTCTCTCCC GCTTCACACC

108961 TCGGAACGCA CCGTTCCGTT CTTATAGAGA GAAAGGCGCT TTCACATCTT CTTAACCCGA

109021 AATGGCTGGG GAGAGGAAAG GTTCCTTTTT TTGAGGGTAC TCCCGGGAAC AGATCCAGTG

109081 GAGACGGGGT GGGGCCTGTA GCTCAGAGGA TTAGAGCACG TGGCTACGAA CCACGGTGTC

109141 GGGGGTTCGA ATCCCTCCTC GCCCACAACC GGCCCAAAAG GGAAGTGCCT TTCCCTCTGG

109201 GGGTAGGAAA ATCATGATCG GGATAATGGA ACTTGGGTGT GGGTCTTTTG TCGAAACGGA

109261 ATGGCCTTTT TTGTATTTCT CGTATTTTTA TTTCTCGTAA ATGGGTTTTA ATTACACATA

109321 GTATGCACTA CCCCCATCAG CGTATTTTTT GTTTTACGCC CCGTAACTCT TCCTCAGCCA

109381 GGCTGGGGCA GAATAGCAGA GCAAGTACAA GTATTAGTAG CCTAACAAAA AAGCGTTCCT

109441 CGTCATTAAT ATGTTTGCTC ACGGCAATTG TGGCCTCTCG GGAGAATCGA TGACTGCATC

109501 TTTGATGCAC TGCTAGTACT AGTCCATCTG AAAATTCTTA ATTGGATAGT TGTAAATAGC

109561 CCCAGGGCTA TGGAACAAAG GATTATCCCG GACCTACACC GAGGTATTGT ATTGACGGTG

109621 ATTTTCAAAT CTCGCAGAAC AGGATGTGAT ACGATGAGAT AGAATGCAAT AGAAACAAAG

109681 ACGGGGAAGG GGTTACCTAC TCTTAACGGT CAAAGCGAGC CCTTTTCTTT TATTCTGAAT

109741 TCTTTAATTC AGAATGAATC AAATCTCCCC AGGTAGGATT CGAACCTACG ACCAGTCAGT

109801 TAACAGCCGA CCGCTCTACC ACTGAGCTAC TGAGGAACAA CGAGAGATTA GATCTCATAG

109861 AGTTCAATTC CCGTTCTCAA CCCATGACCA ATATGAGCTC GAAGCTTCCT TCGTAACTCC

109921 CGGAACTTCT TCGTAGTGGC CCCCTTCCAT GCCTCATTTC ATAGGGAACC TCAAAATGGC

109981 TCTATTTCAT TATATTCCAT CCATATCCCA ATTCCATTCA TTTAATATCC CTTTGGTGTC

110041 ATTGACATAA CAGATGTCGT TTCTAGTCTA TCTCTTTCTA TTTCTTTTCT ATATATGGAA

110101 AGTTCAAAAA TCATCATATA ATAATCCAGA AATTGCAATA GAAAAGAAAA AGGGAGGTTT

110161 GTGATGATTT TGAAATCTTT TCTACTAGGT AATCTAGTAT CCTTATGCAT GAAGATAATC

110221 AATTCGGTCG TTGTGGTCGG ACTCTATTAT GGATTTCTGA CCACATTCTC CATAGGGCCC

110281 TCTTATCTCT TCCTTCTCCG AGCTCAGGTT ATGGAAGAAG GAGAAGAAGG GACCGAGAAG

110341 AAGGTATCGG CAACAACAGG TTTTATTACG GGACAGCTCA TGATGTTCAT ATCGATCTAT

110401 TATGCGCCTC TGCATCTAGC ATTGGGTAGA CCTCATACAA TAACTGTACT AGCTCTACCG

110461 TATCTTTTGT TTCATTTCTT CTGGAATAAT CACAAAAACT TTTTTGATTA TGGATCTACT

110521 ACCAGAAATT CAATGCGTAA TCTCAGCATT CAATGCGTAT TCCTGAATAA TCTAATTTTT

110581 CAATTATTCA ACCATTTCAT TTTACCAAGT TCAATGCTAG CCAGATTAGT CAACATTTAT

110641 ATGTTTCGAT GCAACAACAA GATGTTATTT GTAACAAGCA GTTTTGTTGG TTGGTTAATT

110701 GGTCACATTT TATTCATGAA ATGGGTTGGA TTGGTATTAG TCTGGATACG GCAAAATAAT

110761 TCTATTAGAT CGAATAAGTA CATTCGATCT AATAAGTATC TTGTGTCAGA ATTGAGAAAT

110821 TCTATGGCTC GAATATTTAG TATTCTCTTA TTTATTACCT GTGTCTACTA TTTAGGCAGA

110881 ATACCGTCAC CCATTTTTAC TAACAAACTG AACCCACAAA CGGAAGAAGG GTGGGAAAGT

110941 GAGGAAGAAA CAGCTTCCGA AACGAAGGGG ACTAAACAGG AACAAGAGGG ATCCACCGAA

111001 GAAGATCCTT CTCCTTCCCT TTTTTCGGAA GAAAAGGAGG ATCCGCACAA AATCGATGAA

111061 ACGGAAGAGA TCCGAGTGAA TGGAAAGGAA AAAACAAAGG ATGAATTCCA CTTTACAGAG

111121 ACACGCTATA ACAATAGCCC AGTTTATAAA GGCCTCTTCT GGTTTGAAAA ACCTCTTCTG

111181 ACCTTTCTTT TCGATTATAA ACGATGGAAT CGCCCATTAC GATACATAAA AAATAAGAGA

111241 TTTGAAAGGA CTGCAAGAAA AGAAATGTCA CAATATTTTT TTTATACATG CCGAAGTGAT

111301 GGAAAAGAAA GAATATCTTT TACGTATCCG CCTAGCTTGT CAACTTTTGG AGAAATGATA

111361 CAAAGAAGGA TGTCCTTGCC CACACTAGAA AAACTCTCTT CGGATGAACT GTACAATCAT

111421 TGGGTTTCTA CTAACCAACA CAAAAATAAC AACTTAAACA ACGAGTTTTT AAATAGAATT

111481 GAGGCTCTAG ATACGGGATT TTTTTCTCGG GATATACTCG AAAAAAGTAC TAGATTGTGT

111541 AATGATAAGA CTAGAAAAGA TTACTTGCCT AAAATGTATG ATCCCATTTT GAATGGGTCA

111601 TATCGTGGAA CAATCAAAAA AAAATTTTCA CCGTCAATCA TAAAAAAAAT TTCGTTAGAA

111661 AATTTCATAG AGACAATGGA AATTAATAAG ATTCATAGTA TCCTTCTTCC GGATACTGAT

111721 TACCAAGAGT TTGAACAGAA AATAGACCGA TTTGATAAAA AAACTTTTTC AACGGAAAAT

111781 CGTCATTTCT TTACTTTAAT CAGTAAATTT GATAGAGAAT CGGGATCGAG TTTAAATTTG

111841 AAGGACCTCT CTTTATTTTC AGAAAAAGAA CAAGGAAGGA TTGGTTCAGC AAAAAGAGCA

111901 AAATTTTACA AATTTTTATT GAATACAATT CTAACTAGCC CTAATGGTCA AAAAAGAAAA

111961 AATGTTGTAA TAAAAGAAAT AAGTAAAAAA GTCCCTCGAT GGTCATACAA ACTTATTACC

112021 GAGTTGGAAT TCATGATAGA TACAAAAAGT AATAAATTAA TAAAAAAATT GATACAAAGG

112081 ATAAATACAA GAAAAGATAA GAAGAGATGC GCCCGCCACC TACAGATTTG ATACCTTCGC

112141 CTACAAAGAA AGTCAAAACA CCAACTCCAT TAGTAATTCC ATCAATTACT CGTCTATCAA

112201 AAAAAGAAGT TAACTTGGCG ACTCCTCTTA TCCCCTCAGT ACAGAATGTT GCATAAAAGG

112261 CATCTATGTA ACCACGATTA TATGACCAAT CGTATATACT ATTTATTATT TTGTCCAAAA

112321 AAATTCTCTT AGGACCTGTT TTCACAAAGG AGTTCATTAA ATCTAAATTT AGAAAAGATG

112381 AATAAACAGG TTTATATAAA AAGAAGGCTA TAAATATTCC AAAAAAGGCT ATACTAACTG

112441 AAAAAAGGGC ATCTTTCGAA AATTCATACC AATCTATGGA ATTATTCAAA TTTTGATGTA

112501 AAGGGTTTAT AGATGGAGTT AACCATTTTG ATAATATGTC TAAATACACT CCTTCTTGAT

112561 TGAAAGGAAT TCCTAGGAAT CCAATGAACA AAGTAAATAG AACCAATATA AGTATAGGAA

112621 ATAACATAGT ATTTTCTGAT TCATAAGGAT ACGAAAAAAT CTTCTTATTT CCAAAACATG

112681 GAATAGTAAT AAAAGGTTGT GTGATGTTTT TGGCACTCTG ATCAATTCGA TACGCTTTTT

112741 TTGAAAAAAA AGAAGAAATC TCATAATTAT TCATTGTTAA TAACGTTAAT AAACGAACAT

112801 TTTTGGTAAT TCTTTTCGAA TCTTCTTTAC CCCATAGAGA TATTGAATAG AAGGGAGTAT

112861 TTTTTTTGCC ACTATAATTT TGAAAATGAA CGTGTAAATG TCCTTCAAAA GTAAGTAAAT

112921 AGATTCGAAA CATATAAAAA GCGGTTAATC CTGCTGTAGA CCAAGCTATT ATTGCGAAAA

112981 TGGGTGAATA CAACCAAGTA TCATTCAGAA TTTCATCTTT AGACCAAAAA CAAGCAAGAG

113041 GCGGAATACC ACAAAGAGAA AGTGTACCTA CTAAAAAAGA CGTTTTCGTA ATTGGCACAT

113101 GCTTTGTTAA ACCCCCCATC AAAACCATAT TCTGACTTTT ATTTGGAGAA TATCCAACAA

113161 GAGTTTCCAT TGAATGAATA ACAGATCCAG ATCCTAAAAA CAATAATGCT TTCGAATAAG

113221 CATGAGTAAT CAAATGAAAT AAAGCACTTC GATAAGACCC CATACCTAGA GCTAGCATCA

113281 TATAACCCAA TTGAGACATT GTGGAATAGG CTAAACCTCT CTTAATATCT TTTTGAGCAA

113341 GAGCAAAAGT CGCTCCGAAT AATACTGTTA TTATTCCTAC CAAAGAGATG AAATTCATTA

113401 TGTGAGGGAT AACTATAAAA AGAGGAATAA GCCGAGCTAC AAGAAAAATG CCCGCTGCTA

113461 CCATAGTAGC AGCATGTATA AGAGCCGAAA TCGGAGTAGG TCCCTCCATG GCATCCGGTA

113521 ACCATACATG AAGGGGGAAT TGTGCAGATT TAGCAACCGC GCCCGCAAAT AATAGAACGG

113581 CACAAAAAGT AACAAATAAA GAATTTACTT CATTATTAGA AATCAAAGTA TTGACTATTT

113641 TGAATAAATC TCGAAATTCG AAACTCCCTG TTATCCAATA AAAACCTAAA ATTCCTAATA

113701 ATAAACCAAA ATCCCCGACA CGATTAGTTA CAAACGCCTT TTGGCAAGCA TTTGCCGCAA

113761 CAGGTCGTGT GAACCAAAAC CCTATTAATA GATACGAAGA CATTCCCACC AATTCCCAAA

113821 AAATATAAAT TTGTATCAAA TTAGAACTAG TAACTAATCC TAACATAGAA GTACTGAAAA

113881 AACTCATATA AGCGAAAAAT CTCAAATATC CTTGATCATA AGACATATAA TTATCGCTAT

113941 AAACAAGAAC CAGAATTCCA ATAGTAGTGA TTAATATTGA CATAATAGAG GTAAGTGGGT

114001 CGATCAAGTA ACCAAATTCT AAAGAAAAAT CATTATTGAT GATCCAAGAC CATACATATT

114061 GATAGATAGA ACTTCCATTT ATTTGCTGAA TAGCAAGATT TATCGAAAAA ATCATGACTA

114121 TACTTAACAA GAAAACACTT TGAAAAGCCC ACATACGACG AAGACTTTTT GTTGCCGACG

114181 GAAAAAGAAG AAGTCCCGCT CCTATTAACA GAGGAACTGG AAGTGGAAGG AAAGGTATTA

114241 TCCATGCATA TTGATATGTT TGTTCCATAA AAAAGTTTTT TATTCTTAAT TAATTGTTTC

114301 CGATTTATCG GATTCTACTC CTTTTCAAAA GGAGTCAATA AAAAAAAATC AAGAGATGTA

114361 CTAACTTAAA GATCATTTTA TTATTATATA TTCTTACTTA TTCTGAGTCT TTCCAAAATA

114421 CTTCAAATAG TCAAATAAAG AAGTTACAAT TGGTCAAACG ATATGACCAA CTTGTTCATA

114481 CATAAAAGGC GAGTACTTAG TTATTCGTCT AGTAACTAAG ATTTCTATGA CACAACAGCG

114541 AATTCTATAA CTATAGATAT AGATATAGAT GTGAATCATA AAGAACAACA AATAAAGATA

114601 CCAATCAATA AAAGGAGTCT TTCTTACGAA TATTGTATGT ATATAGAAAA CTTTTTTGTT

114661 GAAAAAATTA CATATCATGC TCTTTTTATT TTTTCTATTT CTAGTAATAT TTTGTATGAT

114721 TTCGCATATC TCTAGTTTTT TCTTTTCTGA AGAGAATATA ATATTGTCTA GAGAATAAAT

114781 AGATAATGAA TAGTAAAGAA CGATTCGTTT TGACCAATAA ATGTCTTTCA CATCCAACTA

114841 TAACAACAAG TAACCTCTAC ATTTTTAAAT GGCAGTTCCA AAAAAACGTA CCTCTATATC

114901 AAAAAAGCGT ATTCGTAAAA ATATTTGGAA GGGGAAGGGG TATTGGGCAG CCTTAAAGGC

114961 GTTGTCATTA GGGAAATCTC TTTCTACCGG AAACTCAAAA AGTTTTTTTG TGCGACAAAC

115021 AAATAAATCA TAAAACAAAA CATTGGAATA ATCTGAATCG AAAAAAAGGC TCATTTTATT

115081 CTTAATATGG AATTAACATT TTCCATTATC TGTTGTTTGG GGCTGATGAA TATAAACTAG

115141 AACTCGCTTC ACTCTTAGGA TATGTAGAAT AAAAACTCTT CGATTTAGGG TTTAGTAAAA

115201 TAAAAAAACT TTTGAAAAAA GAATAAAGAC AAGATACAAG GTTTGAGCCT TTATTTTAGT

115261 AAATTTTATC GGTTTTGGGG GGTGGGGAGT CTTTTTTCCC CATCAACTTA TTTGTCACAA

115321 TTACAATAAT TGAAGTTTTT CTTTTCTTCT TCTATATCTA TATATATCTT GTATATATAT

115381 AATATATAAT ATAATTTATT AAATATATTA AATAAATATA GAAATATATA TCTATAGAAA

115441 GAAGGATAAA TATAAGATCC TTAGGAAAAA TTAATGCATC GTCGAAACTC ATTGATTCCA

115501 TTTCTTTTGA AAAATTTTTT GTGTTACTTT ATGACTTCTT ATTTCTCTGA ATGAATCTAA

115561 GTAATATATT TATCTCCCAT ATATCTGCCG CTTTCAACCC AACCGACAAA AGAAAAGCCT

115621 GGAATTTTTT GTCCGAATAA TGTGTCAGTT TTGAGTAATA AAAGGGGGTC TATTTTGTGT

115681 TCATTGAAAA AATAGATTTT TTAACTTTTA GGAACTTACA TATATATAAA TGAGAAAACT

115741 CCGCTGAAAA AAAAGAAACC CTTTTTAGTT CTATCAAGAA CTAGAGGGTC GAACTTTCTG

115801 AATTCGATTA TAGAAGAGCA TAAACTACAC CTAAGGTAAA TAAAAGATAA ATAAAACCCG

115861 CACCCTAGAA CCTTCAAATT CCTATTTGAA CGAGGTTTTA TGCAGTAATT TAAATCTTTA

115921 CTAAAAAGAT TTCATTGAGT TAATTCCTTC AGTCTCGACG ATTGACTATG AATAGGCTAT

115981 TATGAATTCG AGAAAGCCGC TATGGTGAAA TCGGTAGACA CGCTGCTCTT AGGAAGCAGT

116041 GCGAGAGCAT CTCGGTTCGA GTCCGAGTGG CGGCAGGCCT TCTTCTAAAA AAGATACAAT

116101 AAATTCTATA ATGAATTCAA TTCCCGATTT ATATTTCAAG ATTACTCCCC TTTTATGATA

116161 TTTTCAACTT TAGAGCATAT ATTGACTCAT ATTTCCTTTT CGATCGTTTC AATTGTAATT

116221 ACAATTCATT TGATAACCTT ATTAATCGAT GAAATCATAA AACTATATGA TTCGTCAGAA

116281 AGGGGAATGA TAGCTACTTT TTTATGTATC ACAGGATTAT TAATCACTCG TTGGATTTAT

116341 TCGGGGCATT TCCCACTAAG TGATTTATAT GAATCATTAA TCTTTCTTTC ATGGGGTTTA

116401 TCAGTTATTC ATATAGTTCC CTATTTCAAA AAAAAGCAAA GTCATTTAAA CACAATAACT

116461 GCGTCAAGCG TGATTTTTAC CCAAGGCTTT GCTACTTCGG GTCTTTTAAC TGAAATACAT

116521 CAATCTTCAA TATTAGTACC CGCTCTCCAA TCTGAGTGGT TAATAATGCA CGTAAGTATG

116581 ATGATATTGG GCTATGCAGC TCTTCTATGT GGATCATTAT TATCAGTAGC ACTTCTAGTC

116641 ATTACATTTC GAAAAAACAT AAATCTTTTT TGTAAAAGGA ATCCTTTATT AAATTTAAAA

116701 TTAAACGAGT CATTGTCCTT TGGTGAAATA CAATACATGA ATGAAAGAAA CAATATTTTA

116761 GGAAATGTTT CTTTTTTTTC TGCTAAAAAT TATTACAGGT TCCAATTGAT TCAACAGTTG

116821 GATTATTGGA GTTATCGTGT GATTAGTCTA GGGTTTATCT TGTTAACCAT TGGTATCCTT

116881 TCAGGAGCAG TATGGGCTAA TGAAGCATGG GGATCATATT GGAATTGGGA CCCAAAGGAA

116941 ACTTGGGCAT TTATCACTTG GATCGTATTT TCGGTTTATT TACATACTCG AACCAATAGA

117001 AATTTACAGG TTGAAAATTC CGCAATTGTG GCGGCTATGG GCTTTCTTAT AATTTGGATA

117061 TGCTATTTTG GGGTCAATCT TTTAGGAATA GGGCTGCATA GTTATGGTTC ATTTACGTTA

117121 ACGTCTAATT GAATTCAATA TTCAATATCA ATATAAAGGC TCTTACACAT ATTTACGAAT

117181 AGAAAAATAG AAAAAATAGG TTGTTTGTAT ATAAAAAAAC TTTATGTGAA CCGGTGAGAA

117241 CCATGCGAAT CCCCACACTA CTTGATTCAC ATGGTTCTCA CAAAGACCAA ACATATCGGG

117301 TGAAAATTCT TTGTTTAGCG TATTTTAACT TAAGAGAGGG AAAAGTCTTC TTTTTTTCAT

117361 TCTCTAAATC CTAGGATTTT TTATGAAAAC TACCTATAAA AGAAATTCGA TAGAATAACT

117421 TCGACCTTGT CCACTGAGAG TGAAAGAACA AAATCCGGGT ACATACCAAT ACCTATTACG

117481 GGTAGAAAGA TAGCGATCGA AACAAATAAT TCTCGCGGCC CAGAATCAAA AATATATGAG

117541 TTTGGAGCAT TAAATAGCTT GTATCCATAG AACATCTGGC GTGACATAGA TAATGAATAA

117601 ATAGGAGTTA ATATCATCCC AATTGCCATT ACAAAAGTAA TTAATATTTT GGATATTAAA

117661 AGATATTTTT GGCTTGTAAT GATTCCAAAA AAAACTATCA ATTCTGCAAC AAAACCACTC

117721 ATACCCGGTA ATGCAAGAGA AGCCATCGAA AAGCTACTGA ACATCGTGAA AATTTTTGGC

117781 ATTGGGATAG CTATTCCGCC CATTTCGTCG AGATAAACAA GACGTATTCT ATCATAACTC

117841 GTTCCTGCCA AGAAAAAAAG CGCAGCACCA ATAAATCCAT GAGAGATTAT TTGTAAAATA

117901 GCTCCATTGA GTCCCGTATC GGTTATAGAA CCAATTCCTA TAAGTATGAA ACCCATATGA

117961 GATACAGAGG AATAGGCTAT TCTTTTTTTT AAATTACGTT GGCCGGGAGA TGTTGAAGCT

118021 GCATAGATTA TTTGTATTGT GCCTACTATC ACCAACCATG GAGAAAATAG AGAATGGGCG

118081 TGAGGTAATA ATTCCATATT AATCCGAATC AACCCATACG CTCCCATTTT TAATAAAATT

118141 CCGGCTAAAA GCATACAAGT ACTGTAATGT GCTTCTCCAT GGGTATCTGG TAACCATGTA

118201 TGAAGGGGTA TAATCGGCGA TTTGACAGCA AAAGCAATAA AAAATCCAAT ATAGAATATT

118261 ATTTCCAAGG CCACAGGATA TGACTGATTA ACTACTGTTT GAAAATTTAA TGTTGGTTCA

118321 TTAGAACCAT ATAAACCGAC ACCCAGAACT CCCAGTAAGA GAAAAACGGA ACCTCCCGCC

118381 GTGTATAAAA TAAATTTTGT AGCCGAGTAA AGACGTTTCT TTCCCCCCCA CATGGATAAA

118441 AGTAGATAAA CCGGAATTAA TTCTAACTCC CACATGATGA AAAAAAGTAA AAGGTCCCGA

118501 GAAGAAAATG ATCCTATTTG CCCGCTGTAC ATTGCTAACA TCAGGAAATG AAATAATCGA

118561 GAATCTCGAG TGACTGGCCA AGCCGCTAAA GTAGCTAAAG TAGTGATGAA TCCCGTTAGT

118621 AAAATCGGTC CTATAGAAAG TCCATCTATT CCTAATCTCC AATGGAAATC AAAAAAATTG

118681 ATCCATTTAT AGTCCTCCTC TAGTTGGATT AATGGATCAT CCAATTGAAA CTGATAACAG

118741 AATGCATAGG TTGTTAGAAG GAGTTCTAAA ACACATATAC CTATAGTATA CCACCTAATT

118801 ACTCTATTTC CTCTATGGGG AAGAAATAAA ATTAAGGATC CTGCGAATAT TGGCAAAACT

118861 ACAATTATTG TTAACCAAGG AAAAAAATTC GTGGTAAAGA CAAGATACAC TTGGACCAGA

118921 AAAATCCGTG CTCAAAAAAA ATATGTTATT TTGAGCACGG ATTTTGTCGG TAAAAAAAAT

118981 CAAATGGATT CAAGAAAAGT TTTCTGGAAC ATATCAATAA GCTAGACCCA TACTACGAGT

119041 TGTTTCATGC CATAAAGAAA CTCGAACACT CAAGAAATCG GTTGGACAGG CGGATTCACA

119101 TCTCTTACAA CCGACACAGT CTTCTGTTCT TGGAGCAGAA GCGATTTGTT TAGCTTTACA

119161 TCCGTCCCAG GGTATCATTT CTAATACATC GGTGGGGCAG GCTCGGACGC ATTGAGTACA

119221 CCCTATACAT GTATCATAAA TCTTTACTGA ATGTGACATT GGATCTATAC ATTTTTGAAC

119281 GTCATAAATT TTCGATATAG TAAACTTTTA AATAAATGGT ATATTTAGAT ACTAGACGAA

119341 TCAATGAGTT ATCAGAAAAT TTTTAATTGA CTTCTGCATT GGCTCATGAG AAAGGGCCAA

119401 AATACTTTGA TTTCTTATGT TTGTGTAAAC ATGATTAAAC CCTACGTGGC AGACATGCTG

119461 ATTTAAATTG ATTTATTTTA TGAATATAAT ATTAGAATTT ATATGGTTAA GACTACTTAT

119521 TCAACAAATT CGATTGATTG ATACGAGTTG ATTTTCTGTT ACGATAAATT GCTGAAACAA

119581 TAGCCAGCCC AATAGCCGCT TCCGCGGCTG CAATAGCTAT AACAAAAATA GAGAAAATGG

119641 CTCCTTTTAA TTGACGACTA TCAAAAAAAT CAGAAAATGT TACAAAATTG ATATTAACTG

119701 CATTCAAAAT AAGTTCAAGA CACATAAGAG CCCTAACCAT ATTTCGACTG GTAATCAATC

119761 CATAAAGACC AATAGAAAAT AAATAGGCAC TCAAAACAAG TACATGTTCG AGCATCATTA

119821 AAAAACTCCT TATCAATCTC GATTCATTTC AATATGAACA AAAATTCAAC CAATTAGATT

119881 GACTAGAATA TAACAAATAC GGACCAAAAG AATATATTGG TAATAGATCG AACTAAAGGA

119941 TTTCTATTTG ATATATGAAT TCCAAAAAAA TGAAGGGAAA TCAATGTGAT AACCCAGAAC

120001 AAAGTTTTAT TTGGATTCCT AGTTCGAAAG ATTTATTATT GACGAGCTAC AGCAATTGCA

120061 CCTATTAACG AAACTAAAAG AATTATTGAA ATGAGTTCAA ATGGAAGAAA AAAATCTGTT

120121 GATAAATGAA TTCCAATTTG TTGACTATTA CTTATCAAAT CTTGCTCTAT AATCTGGTTT

120181 GATTTTGTAG TCCAAATAAT CCCGTACCAT GACGTATCTG GAATAGTAGT AATTAGTGAA

120241 ACAAAAAGAC TTGTACAAAC CATCGACGTA ACCCCATCCC CAACGGTCCA AAGGTGAAAA

120301 TCCTTGTAAT ATTCGGAACC ATTCATGAAC ATCACAGCAA AAATGATTAA AACATTTATA

120361 GCCCCTACAT AAATAAGGAG TTGCGCAGCA GCTACAAAAT GGGAGTTCGA TAGAATATAG

120421 AATAAGGATG TACAAACAAG AACCAATCCC AATGAAAAGG CAGAATAAAT TGGATTGGGA

120481 AGTAATACCA CTCCCAAACC TCCTAATATA AGACCCGATC CCAGAAAGAC TAAAAGAAAA

120541 TCATGTATTG GTCCAGGTAA ATCCATTCTA TATATATAAA TAGATAAAAA ATCGAAATCT

120601 TTCATAACTT TATTGACCCG GCCAGGACTT CTTAATTATT AATTGAATTA AATTTGAATA

120661 GATGCGGGTT GATCTAGATA TAGTTATTGT ACCCATTTCA TTCTTTATCC GAAAGAGTAG

120721 TTTGAAACTA TATATTTTTA AAGCATTAAT TTCAATCATT AATAAAATTA ATTAATTAAT

120781 AATCATTAAT ATATAGATTT CAAATCCAAA TGAAATTACG ATTTTCACCA ACAAAACCAA

120841 TTTGGAGTTG TAGTTATTCA CCAAGCAAAA ACCCCATTCT TTTACATCAA AATGGAGTCT

120901 TTTTAATTGG GAATTGCTCT TGAATCAAGC ATTTTTTATT TCAGTCAAAT TCAAAATCGT

120961 TCGAGTTGTG TAATCGTTAA TTATTGACAT TGGTAAACGC CCCAAAGCAA TTTGATTATA

121021 ATTCAATTCG TGACGATCAT ATGTAGAAAG TTCATATTCT TCAGTCATTG ATAAACAATT

121081 TGTTGGACAA TACTCAACGC AATTACCACA AAATATACAG ATTCCGAAAT CAATACTGTA

121141 ATTAAGCAAT CGTTTCTTTC TAATATCCGT TTCCAATTTC CAATCAACAA CAGGGAGATC

121201 TATAGGACAT ACACGAACAC ATACTTCACA AGCAATGCAT TTATCAAATT CAAAGTGGAT

121261 TCGGCCGCGG AAACGCTCCG CTGTAATCAA TTTTTCATAG GGATATTGAA TAGTTACAGG

121321 TAAACGACTC GCGTGTGATA AGGTAATCAT GAAACCTTGA CCGATGTACC TTGCGGCTCG

121381 TACTGTTTGT TGACCATAAT TCATGAACTC AGTTACCATA GAGAACATAT GGTGAATATT

121441 TATAACAATT TATATGCTTG GTTTGTTTCT TTCTCTTGGT TGAGACAAGT TATGAAAATA

121501 AAATTAAAAT ATCGTATTCC TTTACAGTGA AAGAAGTTGG GAAGAAGTTG TTAATAATAG

121561 ATTGCCGAGA GAAATAGGTA AAAGAAATTT CCACCCAAGA TTTAATAGCT GATCCATTCT

121621 TAGCCTTGGT AAAGTCCATC TTGTTATGAT AGAAATGAAT AAAAACAGAT AAGTTTTAGC

121681 TAATGTAATA AAGATACCAA TTATTGTTCC AAAGACTCTA CCCGCCTTAT TTATTTCAAA

121741 AATATCAGGA CCGGGTATGT ACGGAATAGA AAGATTCCAA CCTCCCAAAT AAAGAACCGT

121801 TACAAATAAC GAAGAAACTA GTAAATTCAG ATACGAAGCA ACGTAAAATA AACCAAATTT

121861 TATACCCGAA TATTCGGTTT GATAACCTGC TACCAATTCT TCTTCTGCTT CGGGTAAATC

121921 AAAAGGTAAT CTTTCACACT CGGCTAGGGA CGAAATTAGA AAAACGATAA ACCCTATAGG

121981 TTGACGCCAC AAATTCCACC CCCAAAAACC ATATTTTGAC TGCGCCTCAA CTATATCAAC

122041 TGTACTTGAA CTGTTAGATA ATCATAGTCG ATGATAACAT CACTGTTCGC AGCGCTATTC

122101 CAGAACCGTA CATGAGATTT TCACCTCATA CGGCTCCTCG AAGGTCACAA ATAAATCTAA

122161 GGACCCATTT GCTATTATTT TGATTTTGAT ATGTTTGTAA GATAGAATCA CAATCTATCG

122221 TAAAGTCCCC AATTAGACAA TGGAATTCTG TCTGCTAGAT ATTCTTTTTT ATTAGAAATT

122281 CTAAAGTGCT TCTGAATTGA TCTTATCTTT TAAAAATTTT AAATTTTTTA TTGAGTAATA

122341 ACTTAACCTT TGAATAAAAT GTTTCTTGCA GAAATATCAA TAAATATTCC AACCCGGCAT

122401 TTTTTATTAC GAAAAAATGA GACATTGCAT TAGTTCACGA AACATTACAA GAATCCTATC

122461 TCTATAAAAA AAGATCTCCT TTTGGAATGC AATTCTTTCT ATTTTCTTTT TTCGTTCCTA

122521 TTCTCCTTTC TCATAAAAGG GGACTTTAAA CAAAGCAAAG TAAAGGATTA CTTCGTTCTT

122581 GATAGTTATT TACTTAATCG GTGGATAGGA GCATACTCTG GATCGGAATC GTGGGAAGTA

122641 CTCCTTCATT ATTTCTACTA ACATAAAGTC CCAATTAGAA TCCCTTTTAT GCTATGCAGT

122701 ATGCACAGAA AAATCCTGTT GAATAACTTA CATAATCTCT ATTACGAATC CTTTGTGTAC

122761 CTTGGTGTTC CTAACTATCC ACTCTTTTTG GTCAAAAATA CCCCGTTAGG GTAATACATG

122821 TATGTTAATA ACTAGTAAAA TCCCCATACA GTTGCTCTTT TGAACCCGCT TCAAGTCATG

122881 ATAACTAATC AATAAATCTT GGGATACACA GTATCTAATG TTTATGTTTA CTTTCACTTA

122941 ACGCTTGTAC ATAGGAAATG AGACTGAACT TTTTACTGCA AAATTTGAAG CCGTTTTTTT

123001 CACTCATATA ACTATTTGGT TTAGTTCATC AACCCGAATG CTGAATAAAA AAGGAAAATA

123061 TATATTATAT ATTCAACTCA TGAAACTTCC TTCCTATAAA GAAAACAAAG AGAGGAAATT

123121 TGATGTCTCA ACGAATCACA CGTAGAGATA TTGATAACAC ACATAGAGTT AATGGTATTT

123181 CATAACTAAT TGATTGAGCA GCAGCCCGTA AACCACCTAA AAAGGAATAT TTATTATTTG

123241 ATCCATAGCC TGACATAAGA AGGCCAACGG GAGCAATACT TGAAATGGCA ATCCATAAAA

123301 AAACACCGAT ACTGAGATCG GCTAGAACAA GGTGATAGCC AAAAGGAATT ACTAAATAAC

123361 TTAATAGAAT TGATGTGACT GCTATGGATG GTCCGATACT GAATAAACGA GTATCCCCTC

123421 TTGATGGAAG AAGATTCTCT TTGAAAAGTA ATTTTGTACC ATCTGCTAAA GCTTGAAGAA

123481 GGCCCAAAGG TCCGGCGTAT TCAGGTCCAA TACGTTGTTG TATCCCCGCA GATATTTCTC

123541 TTTCTAACCA AACAATTACT AGTACACCTA TTGTGATTCC TAATACAGGA GTAAAAATAG

123601 GGACAAGCAT CCATATGATC CCATATACCT CTTTTAAGGA TTCCAATCTA AAAAAAGAAT

123661 TGATAGCTTG TCCTTCTGTT GTATCTATTA TCATTTTAAC GATCAACTTC TCCCATAATG

123721 ATATCTATGC TACCTAGTAT CGTCATAATA TCAGCCAATT TCATTCTTTT AACTAACTGA

123781 GGAAGGATTT GCAAATTGAT AAAACCTGGC GGTCGGATTT TCCATCTCCA AGGAAAAACA

123841 CCCTTATCTC CTATCAGAAA AATTCCTAAT TCTCCTTTTG GGGCTTCGAC TCTCACATAA

123901 AGTTCTTGTT TTGACAATTC AAAAGTCGGA GAAGGTTTTT TACTGATGAA TCGATAGTCA

123961 AAATCATTCC ATTCGGGATC CCGTACTTTA TCAAAGCGTC GAATTTCTAA ATTTTCATAG

124021 GGCCCCCCCG GAATTCCTTC TAACGCCTGT TGAATAATTT TTATGGATTC TGTCATTTCA

124081 CTCATTCGTA CTAAATAACG AGCTAATGAA TCCCCCTCTT TTTGCCATTG GACTCCCCAA

124141 TCAAATTCAT CGTAAGACTC ATAATGATCA ACTTTACGAA GATCCCATTG TATTCCGGAA

124201 GCTCGTAGCA TTGGTCCTGA TAAACCCCAA TTTATTGCCT CCTCTCCGCC AATAACGCCT

124261 ACTCCTTCAA CTCGCTCTAA AAAAATAGGA TTCCGTGTAA TAAGCCTTTG ATATTCAGTA

124321 ATTCTTGTTA AAAAATAATC ACAAAAATCC AAACATTTAT CTATCCAGCC ATGAGGTAAA

124381 TCAGCAGCCA CTCCTCCGAC ACGAAAATAA TTATGCATCA TTCGCATACC GGTGGCAGCT

124441 TCGAATAGGT CATATATCAA TTCTCTTTCT CGAAAAATAT AAAAGAAAGG GGTCTGTGCG

124501 CCAATATCCG CCATAAAGGG ACCAAGCCAT AACAAATGCG AAGCTATACG ACTTAACTCC

124561 AACATAATAA CTCTGACATA GCTGGCCCTT TTAGGCACTT GAATATTGCC TAACTGCTCC

124621 GGTGCATTTA CAGTTATTGC TTCTGTGAAC ATAGTAGCTA AATAATCCCA ACGTGTTACA

124681 TAAGGTAAAT ATTGTATAAT TGTTCGGTTT TCCGCAATTT TTTCCATCCC TCTATGTAAA

124741 TAACCCAATA TAGGTTCACA GTCAATAACA TCTTCACCAT CTAGAGTAAC AATGAGTCGA

124801 AGAACACCGT GCATTGATGG GTGCTGAGGA CCCATATTGA CTATCATAAG GTCATTTCGT

124861 GTAGCTGGTG CAGTCATAGG TTTTTTCCCG ATTCATTCTT CCATGAATTG CTGAAAGCAA

124921 AAAGACGTTC ATCAAAATTT AAGCAAAAAT TGAAAACAAT TCTTAAAATT AACGATTTTT

124981 TGTTTCTCGA ATCTCCAACC GACTAATTAA GTCTTTATAA CGTACTCTAT TTTTTTTTGA

125041 CAAATAAGCC AACAGCCGTT GACGTTTTCC TAGAATTTTA CGCAGACCTC TCTGAGATAA

125101 AAAGTCTTTT TTGTGCAATT CCAAATGCGA AGTCAGTCTC CGTATCCTAT TGGTGAAACA

125161 TAGTACTTGA AATTCAACAG ACCCCTTGTT GTCTTTGTTT TCCTCTTTCA AAATAATTAC

125221 ATTGAATGGG TTTTTTATCA TAAAAAAAAA GATCCTCCTA CCTTTTTTAC ATATATTAAT

125281 TTTCTCGATC AGTAATAATA ATATCAGTCA TTTTAATGTA GTATTTAGTA TACACAAGAA

125341 TCTGAATTTC TTTATGGACT TCCTATTTTA TCAATGAAAA CGAATCAATC AAAATTTGAA

125401 TTTGATTCGA GCAGGGATAA AAGGGGGGAT GGTACGTTTT TACACTCACA AGGCGAATAA

125461 TTTAGACCTA AAATTAGGAA GATTCCGTTG ATTCGTTTAT GAATTGTATC CTATCAAATT

125521 GACAAATTGA TACGTCATTT ACTTAGTATT CAATTCAAAT ATGTTATATC TTTTATTAGG

125581 CTGGGCTATA AGACAGGACA TATGCACATC TGTTTGCTTT TCTATATGGT ACATAATAGA

125641 TAGGAAAAAT GTAGTATCAA CCTATTTTTA ATTGTGGATA TGTCCTTAAC ATACTGAAAT

125701 GGCTTCCATT ATTGGTATCA AACCAATATC GATTCATACA AGCTAAGTCT TCTAATCGAT

125761 AATTAGGCCA AAGAAAGAAT TTTAATTTAA TGAATTCGTT TTTATCTCTA TCAAGATGTT

125821 TACTTTGATC CAAAAAAGGA TTCCCACTTT TGAGGTTCTT CTTGTTGCAA AATACTGGAT

125881 TTATATCCAA ACCATTTCTA TTCTTTGAAT TGAAATAAAA AAGAATTCTC AATTCTCTAC

125941 GACGTCTAGA CGATAAAATC TTTTCAGGAA CAAGAAAACC ATAATGCTTT TTGTCTCTAT

126001 TTTGAGTTCG TCTTTGATAT CTTGGGATGG ATTCCTCAAA TTGATTCTTA TCAATATATC

126061 TTTGTTCTCG GTATCTTTGA GGAGTTTGGT ATTTATTCTT ATGAACCAAT GAAATACCTA

126121 TGGTTTGATA CATTAGAAAG TGCCCGTCGT TTTTTACAGA CAAACGGATG GGTTCGATAA

126181 AAAGGATACC CTTTTTTTTC CATTTTATAG GAGTCAATTT CTTCCGAATC ACCATTATAT

126241 CCAGATTTAG TTCTTTCCTT TGAATAGACG ATATAGTAGT ATCTCGTGGA TTTTTCAGTC

126301 CAAGCAAGTA ACAATATATT TTGATATTAT TGATCATTCT TTCAGTTAAA TTCGGAATTA

126361 AACCATCGTC TATTATCCAT TGAAAAAGCA AATAGTGTTC CCGTAATAAA ATGACTTCTT

126421 GTTTCATCTT ATTCCTGATC TTGTCTTGTT TTTTCGTTTT ACCTTGGTTT TGTGCATATG

126481 ATCTAAAACC TCTTTGGCCT GCGGGTTCTT TTTCGTCTTG ATTTCGATTC ATTAATTCAA

126541 AATTTTCAGT TTTCTTCTCT GGTCTAAAAA AATTCCATTT TTGCTTTTCA TTGATGTTTT

126601 TCTTTTTATT AAAATTTAAA AGAAGTAATT TGCTTGGTAT AATCCATCTT TTTATTTTGT

126661 ATACATTAGA AAGTGGCACA ACTTCTGGGA AGAGCGGAAG TTTCAGATTC GTCGATCTAA

126721 GATTTAGGAT TTCTTCATTC ATTTCTATCC AATCAAAATA TTTTCTATCC GAATTTTTTT

126781 CCATATACAT AATATCATCC TTTTCTAGAT AATTATTGAT AGGAATATCC TCGAGTCTTT

126841 CAAAAAATTT TTGTTTATAA TTGTTGTAAT TAAAAGAAAT CTTTTGGTTG TTATTTACTT

126901 CTAATGGCGA TTCATAAATA ATATAGGAGT CCTTTTTCAT TTCATAATTA ATAGATTTAT

126961 ATGATAAAAG ATTATGTATA GAATATTTTG GAAAATTATC TTTTTGGTTT GGTAATGGAT

127021 ATATTTGAAA ATCTTTTTGT TTTTTGTGAT GAAGTAATCG GTCTTTTTCA TATGAATTCC

127081 ATTTTTTGAA ATCTTTCTTT TTAGTCATAT GGCCTTGATT GATTGTAGTT CGCCATTTTT

127141 GTGGTATTAA TCCAGACCAT CTAATCTGAG AGAAATTATA TTGATAATGA CCTCGTAACC

127201 AGTTTTTCCA TTGATTCATT CCTAAACTTG AGAGTTTAGT ATGCCTTAAT TCGGAATGAA

127261 ACATTCCTTG TGTTACAAAA GAATTCTTTA TTGCAGTCGT AAGAAAAAAA GATGTTCCAC

127321 AATAGTCAAG AATGGATCTT AACTTATACA AATGAATAAT ATGGCTTTGT GATAATTTGT

127381 AAAATAAATA TGCTTGCGAC AAGTAGGATA AGTCAAAAAA AAGATGTGAA TTTTCCTTAC

127441 TATTCTTAAT ATTGTAAAGT GCCCTTTTTA GAGTCGAAAT AAAGTGACTT TCTTTTGGAT

127501 TTTTTTTTTT TTTTTTTtCA TGGTTTGTTT CATTATTGTA ACTGTATTTT TTGAATAAAA

127561 AAATGTTTGA TTCAAGAACA AGTCGTGTAG GAATTCTGGC AATATGAATG ATACATAGAA

127621 AGAAATCTGT GTATATTTTT TTAATGAAAA TTTTTATAAA AAAATCTAAT TTATAGATTA

127681 ATCGAGTGCT TCTTCTTTTT ATTTTTAGTA TTTGCCAAAT ATTTTTTGGT GATTTTACAT

127741 TATAACTGGT TGTGTTAGGA CTACTTTTTT TTATTGGCGT TACTTTTTTT TTTTCTTTAG

127801 TAATACTCTT TATTTTAGTT CTGATTGTAC TTGTTCTATC AGTCAGATTT TGAATTTTTT

127861 TTTCTCTCAG TAAAAAATTT GTCCTATCTC TAAATTTACT TTGACTAAAG GAATCATGAA

127921 TTGTCTGATT GTTGATTATA GGATCTTTTT CTTGGGTAGT TTCAATGTAT TCATACCCTT

127981 CTCTCAATCT AAATAATCGA GTTGGATTTA CTTTTGAGAG TTCTTTTTTT ATTCTTTTAA

128041 TATACAGAAA TAAACCGTTT TTGGTAACCC CCCTTTTTGT TTCTTTTGAG TCTTGGAGAA

128101 TTTTTTTTGT TTTTTCTTTA AAAATTATTA GAACTTGAAA ATTATTCTTT TTCATTTTTC

128161 GAATTTTTTT TTTTACTTCC TTAAAAAATGG GCTTAAAAAA GGAATATTTT TTGTGGGTAG

128221 AACCAAAAGG AATGTTAGTT TGCGTTCCCC AAGCCGTTAA AAAACAAAAC TTTTTTTGTT

128281 CTTTTTTTAG ATCTTTATAA GAAGAAAAAG AGGAACGCGG CTTAGATTTG TGCCAAGGTT

128341 TCAAATAGAA AGGAAATACT ATTTTTATCT GAATACCCTC TTTGAACCAA TTTCTCGGAA

128401 GTTCTAGTTC TGGTACTTGA ACACCATCAT AGGTACATAT AATATGCTTT TCTCTATTCC

128461 ACTCCTCGAA ATCCTCAGTC CACTCGGGGG GTTGGGACAA TAAGATACGT CCAATATTTT

128521 TAACTATTAT CAATGAGGAT AAGAAAATAT ATTTTCTAAA AATCGATTGA ATTAGTAATA

128581 TAAAACTTCT TGTTGCTTGC GGATATGGAA GGAAATCCCA GGCCTCCGCG ATTTTTAGCA

128641 ACGCTTTCTC CTTTATTTTG TTCTCCTCTT CTTCCCTTTC TCGTTGGTTC TCCTCTTCTT

128701 CCCTTTCTCG TAGGTTCGCC TCTTCTTTCT TTTGTCTTTT TTTTTGTTCT TCTGTATAAT

128761 CTTTGAATTC TGTGCCTTTA CCCATCCAAT TTTTAAAAAT TAAATTCATC TCTTCGAGTA

128821 TATTAAAAGA AAAAAGGAAG GATTTTTTTA TTCTGTCCAA AAAAAGCGGG GAATGCGCAT

128881 TTGCTTGAAA CAGTTTAAAA ATAACAGTTT TACGCCTTTG AGCACGTATA GAACCCTTGA

128941 TTAAGTCGCG ACGAAAATCA GATTCTTCCG CATAGTCTCT AATAAGCACC CAGTTTTTGG

129001 CACTAGCCTT GCGACTACGT CGTTTGGCAG GCTTATAAAG CATTGTACGG TCACATTTTC

129061 TTGAACGTAT ATCATAATCC GTCGGTATAC CTTCGTGAGA TTCGCCCGAC ATGAATTCCA

129121 ACTCGGTAAT AAGTTTGTAT GACCATCGAG GGACTTTTTT ACTTATTTCT TTTATTACAA

129181 CATTTTTTCT TTTTTGACCA TTAGGGCTAG TTAGAATTGT ATTCAATAAA AATTTGTAAA

129241 ATTTTGCTCT TTTTGCTGAA CCAATCCTTC CTTGTTCTTT TTCTGAAAAT AAAGAGAGGT

129301 CCTTCAAATT TAAACTCGAT CCCGATTCTC TATCAAATTT ACTGATTAAA GTAAAGAAAT

129361 GACGATTTTC CGTTGAAAAA GTTTTTTTAT CAAATCGGTC TATTTTCTGT TCAAACTCTT

129421 GGTAATCAGT ATCCGGAAGA AGGATACTAT GAATCTTATT AATTTCCATT GTCTCTATGA

129481 AATTTTCTAA CGAAATTTTT TTTATGATTG ACGGTGAAAA TTTTTTTTTG ATTGTTCCAC

129541 GATATGACCC ATTCAAAATG GGATCATACA TTTTAGGCAA GTAATCTTTT CTAGTCTTAT

129601 CATTACACAA TCTAGTACTT TTTTCGAGTA TATCCCGAGA AAAAAATCCC GTATCTAGAG

129661 CCTCAATTCT ATTTAAAAAC TCGTTGTTTA AGTTGTTATT TTTGTGTTGG TTAGTAGAAA

129721 CCCAATGATT GTACAGTTCA TCCGAAGAGA GTTTTTCTAG TGTGGGCAAG GACATCCTTC

129781 TTTGTATCAT TTCTCCAAAA GTTGACAAGC TAGGCGGATA CGTAAAAGAT ATTCTTTCTT

129841 TTCCATCACT TCGGCATGTA TAAAAAAAAT ATTGTGACAT TTCTTTTCTT GCAGTCCTTT

129901 CAAATCTCTT ATTTTTTATG TATCGTAATG GGCGATTCCA TCGTTTATAA TCGAAAAGAA

129961 AGGTCAGAAG AGGTTTTTCA AACCAGAAGA GGCCTTTATA AACTGGGCTA TTGTTATAGC

130021 GTGTCTCTGT AAAGTGGAAT TCATCCTTTG TTTTTTCCTT TCCATTCACT CGGATCTCTT

130081 CCGTTTCATC GATTTTGTGC GGATCCTCCT TTTCTTCCGA AAAAAGGGAA GGAGAAGGAT

130141 CTTCTTCGGT GGATCCCTCT TGTTCCTGTT TAGTCCCCTT CGTTTCGGAA GCTGTTTCTT

130201 CCTCACTTTC CCACCCTTCT TCCGTTTGTG GGTTCAGTTT GTTAGTAAAA ATGGGTGACG

130261 GTATTCTGCC TAAATAGTAG ACACAGGTAA TAAATAAGAG AATACTAAAT ATTCGAGCCA

130321 TAGAATTTCT CAATTCTGAC ACAAGATACT TATTAGATCG AATGTACTTA TTCGATCTAA

130381 TAGAATTATT TTGCCGTATC CAGACTAATA CCAATCCAAC CCATTTCATG AATAAAATGT

130441 GACCAATTAA CCAACCAACA AAACTGCTTG TTACAAATAA CATCTTGTTG TTGCATCGAA

130501 ACATATAAAT GTTGACTAAT CTGGCTAGCA TTGAACTTGG TAAAATGAAA TGGTTGAATA

130561 ATTGAAAAAT TAGATTATTC AGGAATACGC ATTGAATGCT GAGATTACGC ATTGAATTTC

130621 TGGTAGTAGA TCCATAATCA AAAAAGTTTT TGTGATTATT CCAGAAGAAA TGAAACAAAA

130681 GATACGGTAG AGCTAGTACA GTTATTGTAT GAGGTCTACC CAATGCTAGA TGCAGAGGCG

130741 CATAATAGAT CGATATGAAC ATCATGAGCT GTCCCGTAAT AAAACCTGTT GTTGCCGATA

130801 CCTTCTTCTC GGTCCCTTCT TCTCCTTCTT CCATAACCTG AGCTCGGAGA AGGAAGAGAT

130861 AAGAGGGCCC TATGGAGAAT GTGGTCAGAA ATCCATAATA GAGTCCGACC ACAACGACCG

130921 AATTGATTAT CTTCATGCAT AAGGATACTA GATTACCTAG TAGAAAAGAT TTCAAAATCA

130981 TCACAAACCT CCCTTTTTCT TTTCTATTGC AATTTCTGGA TTATTATATG ATGATTTTTG

131041 AACTTTCCAT ATATAGAAAA GAAATAGAAA GAGATAGACT AGAAACGACA TCTGTTATGT

131101 CAATGACACC AAAGGGATAT TAAATGAATG GAATTGGGAT ATGGATGGAA TATAATGAAA

131161 TAGAGCCATT TTGAGGTTCC CTATGAAATG AGGCATGGAA GGGGGCCACT ACGAAGAAGT

131221 TCCGGGAGTT ACGAAGGAAG CTTCGAGCTC ATATTGGTCA TGGGTTGAGA ACGGGAATTG

131281 AACTCTATGA GATCTAATCT CTCGTTGTTC CTCAGTAGCT CAGTGGTAGA GCGGTCGGCT

131341 GTTAACTGAC TGGTCGTAGG TTCGAATCCT ACCTGGGGAG ATTTGATTCA TTCTGAATTA

131401 AAGAATTCAG AATAAAAGAA AAGGGCTCGC TTTGACCGTT AAGAGTAGGT AACCCCTTCC

131461 CCGTCTTTGT TTCTATTGCA TTCTATCTCA TCGTATCACA TCCTGTTCTG CGAGATTTGA

131521 AAATCACCGT CAATACAATA CCTCGGTGTA GGTCCGGGAT AATCCTTTGT TCCATAGCCC

131581 TGGGGCTATT TACAACTATC CAATTAAGAA TTTTCAGATG GACTAGTACT AGCAGTGCAT

131641 CAAAGATGCA GTCATCGATT CTCCCGAGAG GCCACAATTG CCGTGAGCAA ACATATTAAT

131701 GACGAGGAAC GCTTTTTTGT TAGGCTACTA ATACTTGTAC TTGCTCTGCT ATTCTGCCCC

131761 AGCCTGGCTG AGGAAGAGTT ACGGGGCGTA AAACAAAAAA TACGCTGATG GGGGTAGTGC

131821 ATACTATGTG TAATTAAAAC CCATTTACGA GAAATAAAAA TACGAGAAAT ACAAAAAAGG

131881 CCATTCCGTT TCGACAAAAG ACCCACACCC AAGTTCCATT ATCCCGATCA TGATTTTCCT

131941 ACCCCCAGAG GGAAAGGCAC TTCCCTTTTG GGCCGGTTGT GGGCGAGGAG GGATTCGAAC

132001 CCCCGACACC GTGGTTCGTA GCCACGTGCT CTAATCCTCT GAGCTACAGG CCCCACCCCG

132061 TCTCCACTGG ATCTGTTCCC GGGAGTACCC TCAAAAAAAG GAACCTTTCC TCTCCCCAGC

132121 CATTTCGGGT TAAGAAGATG TGAAAGCGCC TTTCTCTCTA TAAGAACGGA ACGGTGCGTT

132181 CCGAGGTGTG AAGCGGGAGA GAAGGGATGT CATAATTAAT TGGGGTTTTG AATAAGACGA

132241 CCTTTTTCTT TTTCATTTAT TTCTTTTTCA ATATGAAATA GAAATAAGAA TGGGAGGTGT

132301 TAAGCTTTTT ATCATCCTGG CGTCGAGCTA TTTTTCCGCA GGACCTCCCC TACAGTATCG

132361 TCACCGCAGT AGAGTTTAAC TACCAAGTTC GGGATGGATT GGTGTGGTTC CTCTACGCCT

132421 AGGACACCAG AATATCGAAC CATGAACGAA GAAAGGCATG AGAGAAAAGC ATATTGGCTA

132481 GTGATTGTGA GGCCCCAATT CTTGACTGGA GGGGACACCA AAGGCCTCTG CCCTTCCATC

132541 CCTTGGATAG ATAGAGAGGG CAGAGCTTTT GGTTTTTTCA TGTTGTCAAA GAGTTGAACA

132601 ATGGTTTTTT CGTGTTGTCA AAGAGTTGAA CAATGAAAAT AGATGGCGAG TGCCTGATCG

132661 AATTGATCGG GTCATGTAGG AACAAGGTTC AAGTCTATCG GTCTGTTAGG ATGCCTCAGC

132721 TGCATACATC ACTGTACTTC CACTTGACAC CTATCGTAAT GATAAACGGC TCGTCTCGCC

132781 GTGACCTTCT CTTGAATTCT CAAAACTTCT GTCGCTCCAT CCCCGCAGGG GCAGAGAACC

132841 CGTCGCTATC TTGGCTGTGC TACCGGAGGC TCTGGGGAAG TCGGAATAGG AGAGCACTCA

132901 TCTTGGGGTG GGCTTACTAC TTAGATGCTT TCAGCAGTTA TCCGCTCCGC ACTTGGCTAC

132961 CCAGCGTTTA CCGTGGGCAC GATAACTGGT ACACCAGAGG TGCGTCCTTC CCGGTCCTCT

133021 CGTACTAGGG AAAGGTCCTC TCAATGCTCT AACGCCCACA CCGGATATGG ACCGAACTGT

133081 CTCACGACGT TCTGAACCCA GCTCACGTAC CGCTTTAATG GGCGAACAGC CCAACCCTTG

133141 GAACATACTA CAGCCCCAGG TGGCGAAGAG CCGACATCGA GGTGCCAAAC CTTCCCGTCG

133201 ATGTGAGCTC TTGGGGAAGA TCAGCCTGTT ATCCCTAGAG TAACTTTTAT CCGTTGAGCG

133261 ACGGCCCTTC CACTCGGCAC CGTCGGATCA CTAAGGCCGA CTTTCGTCCC TGCTCGACGG

133321 GTAGGTCTTG CAGTCAAGCT CCCTTCTGCC TTTGCACTCG AGGGCCAATC TCCGTCCGGC

133381 CCGAGGAAAC CTTTGCACGC CTCCGTTACC TTTTGGGAGG CCTACGCCCC ATAGAAACTG

133441 TCTACCTGAG ACTGTCCCTT GGCCCGTAGG TCCTGACACA AGGTTAGAAT TCTAGCTCTT

133501 CCAGAGTGGT ATCTCACTGA TGGCTCGGGC CCCCCCGGAA GGAGGCCTTC TTCGCCTTCC

133561 ACCTAAGCTG CGCAGGAAAG GCCCAAAGCC AATCCCAGGG AACAGTGAAG CTTCATAGGG

133621 TCTTTCTGTC CAGGTGCAGG TAGTCCGCAT CTTCACAGAC ATGTCTATTT CACCGAGCCT

133681 CTCTCCGAGA CAGTGCCCAG ATCGTTACGC CTTTCGTGCG GGTCGGAACT TACCCGACAA

133741 GGAATTTCGC TACCTTAGGA CCGTTATAGT TACGGCCGCC GTTCACCGGG GCTTCGGTCG

133801 CCGGCTCCCC TGTCATCAGG TCACCAACTT CCTTGACCTT CCGGCACTGG GCAGGCGTCA

133861 GCCCCCATAC ATGGTCTTAC GACTTTGCGG AGACCTGTGT TTTTGGTAAA CAGTCGCCCG

133921 GGCCTGGTCA CTGCGACCCC CTTTGTGAGG AGGCACCCCT TCTCCCGAAG TTACGGGGCT

133981 ATTTTGCCGA GTTCCTTAGA GAGAGTTGTC TCGCGCCCCT AGGTATTCTC TACCTACCTA

134041 CCTGTGTCGG TTTCGGGTAC AGGTACCCTT TTGTTGAAGG TCGTTCGAGC TTTTCCTGGG

134101 AGTATGGCAT GGGTTACTTC AGCGCCGTAG CGCCTGGTAC TCGAACATTG GCTCGAGGCA

134161 TTTTCTCTAC CCCTTCTTAC CCTGAAAAAG CAGGGTCACC TTACGTCCTT GAACCGATAA

134221 CCATCTTTCG GCTAACCTAG CCTCCTCCGT CCCTCGGGAC CAACAAGGGG TAGTACAGGA

134281 ATATTCACCT GTTGTCCATC GACTACGCCT TTCGGCCTGA TCTTAGGCCC TGACTCACCC

134341 TCCGTGGACG AACCTTGCGG AGGAACCCTT AGGTTTTCGG GGCATTGGAT TCTCACCAAT

134401 GTTTGCGTTA CTCAAGCCGA CATTCTCGCT TCCGCTTCGT CCACTACCGC TCGCGCGGGT

134461 GCTTCCCTCT AAGGCGGAAC GCTCCCCTAC CGATGTATTT TTACATCCCA CAGCTTCGGC

134521 AGATCGCTTA GCCCCGTTCA TCTTCGGCGC AAGAGCGCTC GATCAGTGAG CTATTACGCA

134581 CTCTTTCAAG GGTGGCTGCT TCTAGGCAAA CCTCCTGGCT GTCTCTGCAC CCCTACCTCC

134641 TTTATCACTG AGCGGTCATT TAGGGGCCTT AGCTGGTGAT CCGGGCTGTT TCCCTCTCGA

134701 CGATGAAGCT TATCCCCCAT CGTCTCACTG GCCGACCTTG ACCCCTGTTA TTTTGAGGTC

134761 ATATCTAGTA TTCAGAGTTT GCCTCGATTT GGTACCGCTC TCGCGGCCCG CACCGAAACA

134821 GTGCTTTACC CCTAGATGTC CAGTCAACTG CTGCGCCTCA ACGCATTTCG GGGAGAACCA

134881 GCTAGCTCTG GGTTCGAGTG GCATTTCACC CCTAACCACA ACTCATCCGC TGATTCTTCA

134941 ACATCAGTCG GTTCGGACCT CCACTTAGTT TCACCCAAGC TTCATCCTGG TCATGGATAG

135001 ATCACCCAGG TTCGGGTCCA TAAGCAGTGA CAATTGCCCT ATGAAGACTC GCTTTCGCTA

135061 CGGCTCCGGT GGGTTCCCTT AACCAAGCCA CTGCCTATGA GTCGCCGGCT CATTCTTCAA

135121 CAGGCACGCG GTCAGAGCCC CGGGCTCCTC CCACTGCTTG GGAGCTTACG GTTTCATGTT

135181 CTATTTCACT CCCCGATGGG GGTTCTTTTC ACCCTTCCCT CACGGTACTA CTTCGCTATC

135241 GGTCACCCAG GAGTATTTAG CCTTGCAAGG TGGTCCTTGC TGATTCACAC GGGATTCCAC

135301 GTGCCCCATG CTACTCGGGT CAGAGCGTAA GCTAGTGATG CTTTCGGCTA CTGGACTCTC

135361 GCCATCTAGG GTGCAGTACT CGACCGCTTC GCCTAGCAGC ACGACGCTTG TATTGCTCTC

135421 CCACAACCCC GTTTTCACGG TTTAGGCTGC TCCCATTTCG CTCGCCGCTA CTACGGGAAT

135481 CGCTTTTGCT TTCTTTTCCT CTGGCTACTA AGATGTTTCA GTTCACCAGG TTGTCTCTTG

135541 CCTGCCCATG GATTCAGCAG CAGTTTGAAA GGTTGACCTA TTCGGGAATC TCCGGATCTA

135601 TGCTTATTTT CAACTCCCCG AAGCATTTCG TCGCTTACTA CGCCCTTCCT CGTCTCTGGG

135661 TGCCTAGGTA TCCACCGTAA GCCTTTCCTC GTTTGAACCT CGCCCTTAAC TTTAAGTCTA

135721 TGCCATCCTA AGGTGCTGCT AAATGGATGG ATCTTATCAA CGTCCATGAA TGATAAATCA

135781 TAGATCGAAC CGCCGAATCG GAAAAATTGG GTGCTATCAT ATAGCTTTGT ATCGGCTAAG

135841 TTCACGAGTT GGAGATAAGC GGACTCGAAC CGCTGACATC CGCCGCAGGG TAAACCACCG

135901 CCTCTCAGGT CCCCCGACTG ATTCTACCAT AGAGGCCAAT GATAGACAAT AACTCCCCCC

135961 CGAACACAGC TTACAACTTT CATCGTACTG TGCTCTCCAA AGAGCAACTC TTCTCAAAAT

136021 CTCACCCAAA AGGTGCTGAG TTGGAATCCC ATTCTAACTA AGGATTCTTG TGGTTCCGGA

136081 GGATCCAGCT ACAGGAGAAC CGGGAACGGA GAGCTTTCCC CCCCTTTCCG CCGACTCTTT

136141 GGTCTTACGA ACGCAGGTTT TAAGAATGAG TGATTGCCCT TCTCCGACCC TTACTGCCCA

136201 ACCTGAGAGC GGACAGCTAA TGCGTTCCAC TTATTGAACA GGGTTCTATG GTCGGTCCGT

136261 GACCCCTGGA TGCCGAAGGC GTCCTTGGGG TGATCTCGTA GTTCCTACGG GGTGGAGATG

136321 ATGGGGTCGG TCCATGGATT TTCCTTCCTT TTTCCGCATT TCGCTCAAAG GGTTGAAGGG

136381 AGATAGTGCA TCAAGCTGTT CGCAAGGCCA ACTTGATCCT CTTCCCCAGA GATCCCAGAT

136441 TAGGGAACCC TAGGAGAGCC GCCGACTCCA ACTACCGTCC ATGTACGATC CATACTAGAT

136501 CTGACCAACT GCCCATCCTA CCTCCTCTAC GTTCTTGACA GCCCGTCTTT GTCTCAGTAG

136561 AGTCTTTCAG TGGCATGTTT CGGTCCTCTT CCCCATTACT TAGAAAAAGT GAGCCACCGG

136621 TTCAGGTACA AGATACTATC ATTACCGCCT GGACAATTAG ACATCCAACC CGTAATCGCA

136681 ACGACCCAAT TGCAAGAGCG GAGCTCTACC AACTGAGCTA TATCCCCCCG AGCCAAGCGG

136741 AGCATGCATG AAGGAGTCAG ATGCTTCTTC TATTCTTTTC CCTGGCGCAG CTGGGCCATC

136801 CTGGACTTGA ACCAGAGACC TCGCCCGTGA AGTAAATCAT CGCACCTACG GTCCAACCAA

136861 TTGGGAGAGA ATCAATAGAT TCCTTTTCGG GAGCGATTCA TCCTTCCCGA ACGCAGCATA

136921 CAACTCTCCG TTGTACTGCG CTCTCCAAGT GTGCTTGTTC CCCCCTTCTT CCTTACCATG

136981 GCAAGTCTTT GTGAAAGAAC TCCGATGAGA AGAAAAAAGA AGGCGTTAAG AGACCCTCCT

137041 GGCCCAACCC TAGACACTCT AAGATCCTTT TGATTGCAGC TTTCTCCAGA CCTCCGGGAA

137101 AAGCATGAAA AAAAACGGCT CGAATGGTAT GATCCCTCCG TCACCCCAGA ATGAAAGGGG

137161 TGATCTCGTA GTTCTTGGTC TGTGAAGATG CGTTGTTAGG TGCTCCATTT TATTTTCCCA

137221 TTGAGGCCGA ACCTAAACCT GTGCTCGAGA GATAGCTGTC CATACACTGA TAAGGGATGT

137281 ATGGATTCTC GAGAAGAGAG GAGCCATGGT GGTCCCCCCC GGACCGCCCG GATCCCACGA

137341 GTGAATCGAA AGTTGGATCT ACATTGGATC TCACCTGAAT CGCCCCATCT ATCCTCCTGA

137401 GGAGAAGTTT GGTTTCAAAC CCCGGTTCGA ACAGGAGGAG TACGCCATGC TAATGTGCCT

137461 TGGATGATCC ACATCTCAGG GTCAGGCGCC GATGAGCACA TTGAACTATC CATGTGGCTG

137521 AGAGCCCTCA CAGCCCAGGC ACAACGACGC AATTATCAGG GGCGCGCTCT ACCACTGAGC

137581 TAATAGCCCG TCGTGCGAGC CTCCCACTGG GGGCCCGCTA TGCCAGAAGC GAGAGAAACC

137641 CCATcCCtCT CTCtttcCTT TTTTCGCCCT CATATCGCCA CACGGGGGGA CATGGGGACG

137701 TAAAAAAGGG GATCCTATCA ACTTGTTCCG ACCTGGGATA ATAAGCTCAT GAGCTTGGTC

137761 TTACTTCACC GTCGAGAAAC GAAAGAAGAC TTCCATCTCC AAGTTTCACT CAGACCTAGC

137821 TCCCTTCTTT TTGGGTGTGA AGCAGTGTCA AACCAAAATA CCCAATAAAC ATTAGCTCTC

137881 CCTGAAAAGG AGGTGATCCA GCCGCACCTT CCAGTACGGC TACCTTGTTA CGACTTCACT

137941 CCAGTCACTA GCCCTGCCTT CGGCATCCCC CTCCTTGCGG TTAAGGTAAC GACTTCGGGC

138001 ATGGCCAGCT CCCATAGTGT GACGGGCGGT GTGTACAAGG CCCGGGAACG AATTCACCGC

138061 CGTATGGCTG ACCGGCGATT ACTAGCGATT CCGGCTTCAT GCAGGCGAGT TGCAGCCTGC

138121 AATCCGAACT GAGGACGGGT TTTTGGAGTT AGCTCACCCT CGCGGGATCG CGACCCTTTG

138181 TCCCGGCCAT TGTAGCACGT GTGTCGCCCA GGGCATAAGG GGCATGATGA CTTGACGTCA

138241 TCCTCACCTT CCTCCGGCTT ATCACCGGCA GTCTGTTCAG GGTTCCAAAC TCAACGATGG

138301 CAACTAAACA CGAGGGTTGC GCTCGTTGCG GGACTTAACC CAACACCTTA CGGCACGAGC

138361 TGACGACAGC CATGCACCAC CTGTGTCCGC GTTCCCGAAG GCACCCCTCT CTTTCAAGAG

138421 GATTCGCGGC ATGTCAAGCC CTGGTAAGGT TCTTCGCTTT GCATCGAATT AAACCACATG

138481 CTCCACCGCT TGTGCGGGCC CCCGTCAATT CCTTTGAGTT TCATTCTTGC GAACGTACTC

138541 CCCAGGCGGG ATACTTAACG CGTTAGCTAC AGCACTGCAC GGGTCGATAC GCACAGCGCC

138601 TAGTATCCAT CGTTTACGGC TAGGACTACT GGGGTATCTA ATCCCATTCG CTCCCCTAGC

138661 TTTCGTCTCT CAGTGTCAGT GTCGGCCCAG CAGAGTGCTT TCGCCGTTGG TGTTCTTTCC

138721 GATCTCTACG CATTTCACCG CTCCACCGGA AATTCCCTCT GCCCCTACCG TACTCCAGCT

138781 TGGTAGTTTC CACCGCCTGT CCAGGGTTGA GCCCTGGGAT TTGACGGCGG ACTTAAAAAG

138841 CCACCTACAG ACGCTTTACG CCCAATCATT CCGGATAACG CTTGCATCCT CTGTATTACC

138901 GCGGCTGCTG GCACAGAGTT AGCCGATGCT TATTCCCCAG ATACCGTCAT TGCTTCTTCT

138961 CCGGGAAAAG AAGTTCACGA CCCGTGGGCC TTCTACCTCC ACGCGGCATT GCTCCGTCAG

139021 GCTTTCGCCC ATTGCGGAAA ATTCCCCACT GCTGCCTCCC GTAGGAGTCT GGGCCGTGTC

139081 TCAGTCCCAG TGTGGCTGAT CATCCTCTCG GACCAGCTAC TGATCATCGC CTTGGTAAGC

139141 TATTGCCTCA CCAACTAGCT AATCAGACGC GAGCCCCTCC TCGGGCGGAT TCCTCCTTTT

139201 GCTCCTCAGC CTACGGGGTA TTAGCAGCCG TTTCCAGCTG TTGTTCCCCT CCCAAGGGCA

139261 GGTTCTTACG CGTTACTCAC CCGTCCGCCA CTGGAAACAC CACTTCCCGT CCGACTTGCA

139321 TGTGTTAAGC ATGCCGCCAG CGTTCATCCT GAGCCAGGAT CGAACTCTCC ATGAGATTCA

139381 TAGTTGCATT ACTTATAGCT TCCTTGTTCG TAGACAAAGC GGATTCGGAA TTTTCTTTCA

139441 TTCCAAGGCC TAACTTGTAT CCATGCGCTT CATATTCGCC CGGAGTTCGC TCCCAGAAAT

139501 ATAGCCATCC CTGCCCCCTC ACGTCAATCC CACGAGCCTC TTATCCATTC TCGTTCAATC

139561 ACGGCGGGGG AGCAAATCAA AGTCGAAAAA CTCACATTGG GTTTAGGGAT AATCAGGCTC

139621 GAACTGATGA CTTCCACCAC GTCAAGGTGA CACTCTACCG CTGAGTTATA TCCCTTCCCT

139681 GCCCCCATCG AGAAATAGAA CTGACTAATC CTAAGGCAAA GGGTCGAGAA ACTCAACGCC

139741 ACTATTCTTG AACAACTTGG AGCCGGGCCT TCTTTTCGCA CTATTACGGA TATCCAAATT

139801 ATGGGAAAAG TCGGATTCCG TTGTCAACTG TCCCTATCGG AAATAGGATT GACTACGGAT

139861 TCGAGCCATA GCACATGGTT TCATAAAACC ACACGACTTT CCCGATCTAA ATCAAGCAGG

139921 TTTTACATGA AGAAGGTTTG GCTCAGCATG TTCTATTCGA TATGGGTCGG AGAAGAACCC

139981 GACCCGGTAT TATTAAAAAA ATAGAGGAAG CAGAAACAAG TCAAGATGAT ACGGATCAAC

140041 CCCTTCTTCT TGCGCCAAAG ATCTTACCAT TTCCGAAGGA ACTGGAGTTC CATCTCTTTT

140101 CCATTTCCAT TCAAGAGTTC TTATGTGTTT CCACGCCCCC TTGAGACCCC GAAAAATGGA

140161 CAAATTCCTT TTCTTAGGAA CACATACAAG ATTCGTCACA AAAAGGATAA CGGTAACCCC

140221 ACCATTAACT ACTTCATTTA TGAATTTCAT AGTAATAGAA ATACATGTCC TACCGAGACA

140281 GAATTTGTAA CTTGCTATCC TCCTGCCTAG CAGGCAAAGA TTTACCTCCG TGGAAAGAAT

140341 GATTCATTCG GATCGACATG AGAGTCCAAC TACATTGCAT TGCCAGAATC CATGTTGTAT

140401 ATTTGAAAGA GGTTGACCTC CTTGCTTCTC TCACGGTACA ATCCTCTTCC CGCCGAGCCC

140461 CCTTTCTCCT CGGTCCACAG ATACAAAATG TAAGACTGGC GCCAACAGCT CATCACGAAA

140521 GAAAGGACTC ACTAAGCCGG GATCACTAAC TAATACTAAT CTAACACTAA CTAATACTAA

140581 TCTAATATCT AATATTCTAA TATAATAGAA ATACTAGAAA ATACTAATAT AATAGAAAAG

140641 AACTGTCTTT TCTGTATACT TTCCCCGGTT CCGTTGCTAC CGCGGGCTTT ACGCAATCGA

140701 TCGGATTATA TAGATATCCC TTCAACACAA CATAGGTCAT CGAAAGGATC TCGGAGACCC

140761 ACCAAAGCAC GAAAGCCAGG ATCTTTCAGA AAATGGATTC CTATTCGAAA AGTGCATAAC

140821 CGCATGGATA AGCTCACACT AACCCGTCAA TTTGGGATCC AATTCAAAAT TTTCCTCGGG

140881 AGGTATCGGG AAGGAATTCG AATGGAATAA TATCGATTCA TACAGAAGAA AAGGTTCTCT

140941 ATTGATTCAA ACACTGTACC TATGGGATAT AGGAAGAGGA AAAAACAGAA GATTTCACAT

141001 AGTATTTTTG ATCAAAAAAG AAATCTGATT TATTTCGTAC CCTTCGCTCA ATGAGAAAAT

141061 GGGTCAAATT CTAAAGGATC AAACCTATGG GACTTAAGGA ATGATATAAA AAAAGAGAGG

141121 GAAAAATATT CAATAATAAA AAAAAAGAAA ATAAGTAAAC TAAGTAAAAA AAATATAGAA

141181 GAACCCAGAT TCCAAATGAA CAAATTCAAA CTTGAAAAGG ATCTTTCTGA TTCTCGAAGA

141241 ATGAGGGGCA AAGGGATTGG ATTGATCGAG AAAGATCTCT TGTTCTTATT ATAAGATCGT

141301 GATTGGATAC GCATATGTTT GGTAAAGAGA ATAATCTTCT CCTTTGAGTT TGAGAATAAT

141361 CAAAAAAGGA AAGTGTTCAA TTGGAACATG AAAACGTGAC TCAATTGGTC CTAGTTACTC

141421 TTCGGGACGG AGTGGAAAGG GGGAGGGGAT TCTCGAACGC GGAAAAGGAT CCAATGACTT

141481 CGAAAGAATT GAACGAGGAG CCGTATGAGG TGAAAATCTC ATGTACGTTT CCGTAGAGTG

141541 GCAGTAAGGG TGACTTATCT GTCAACTTTT CCACTATCAC CCCCAAAAAA CCAAACTCTG

141601 CCCTACGTAA AGTTGCCAGA GTACGATTAA CCTCTGGATT TGAAATCACT GCTTATATAC

141661 CCGGTATTGG CCATAATTCA CAAGAACATT CTGTAGTCTT AGTAAGAGGG GGAAGGGTTA

141721 AGGATTTACC CGGTGTGAGA TATCACATTG TTCGAGGAAC CCTAGATGCT GTCGGAGTAA

141781 AGGATCGTCA ACAAGGGCGT TCTAGTGCGT TGTAGATTCT TATCCAAGAC TTGTATCATT

141841 TGATGATGCC ATGTGAATCG CTAGAAACAT GTGAAGTGTA TGGCTAACCC AATAACGAAA

141901 GTTTCGTAAG GGGACTGGAG CAGGCTACCA TGAGACAAAA GATCTTCTTT CTAAAGAGAT

141961 TCGATTCGGA ACTCTTAGAT GTCCAAGGTT CAATATTGAA ATAATTTCAG AGGTTTTCCC

142021 TGACTTTGTC CGTGTCAACA AACAATTCGA AATACCTCGA CTTTTTTAGA ACAGGTCCGA

142081 GTCAAATAGC AATGATTCGA AGCACTTCTT TTTACACTAT TTCGGAAACC CAAGGACTCA

142141 ATCGTATGGA TATGTAAAAT ACAGGATTTC CAATCCTAGC AGGAAAAGGA GGGAAACGGA

142201 TACTCAATTT AAAGTGAGTA AACAGAATTC CATACTCGAT CTCATAGATA CATATAGAAT

142261 TCTGCGGAAA GCCGTATTCG ATGAAAGTCG TATGTACGGC TTGGAGGGAG ATCTTTCATA

142321 TCTTTCGAGA TCCACCCTAC AATATGGAGT AAAAAAGCCA AAATAAGTGA TTTTAGCCCT

142381 TATAAAAAAA GAAAACTGAT TCTTGAACCC CCTTCACGCT CATGTCACGT CGAGGTACTG

142441 CAGAAGAAAA AACTGCAAAA TCCGATCCAA TTTATCGTAA TCGATTAGTT AACATGTTGG

142501 TTAACCGTAT TCTGAAACAC GGAAAAAAAT CATTGGCTTA TCAAATTATC TATCGAGCCG

142561 TGAAAAAGAT TCAACAAAAG ACAGAAACAA ATCCACTATC TGTTTTACGT CAAGCAATAC

142621 GTGGAGTAAC TCCCGATATA GCAGTAAAAG CAAGACGTGT AGGTGGATCG ACTCATCAAG

142681 TTCCCATTGA AATAGGATCC ACACAAGGAA AAGCACTTGC CATTCGTTGG TTATTAGCGG

142741 CATCCCGAAA ACGTCCGGGT CGAAATATGG CTTTCAAATT AAGTTCCGAA TTAGTGGATG

142801 CTGCCAAAGG GAGTGGCGAT GCCATACGCA AAAAGGAAGA GACTCATAGA ATGGCAGAGG

142861 CAAATAGAGC TTTTGCACAT TTTCGTTAAT CCATGAACAG GATCTATACA TCTCGATCGG

142921 AAAAGAATCA AGAGAAAAAG AAAGAATCGG AATTGATCGA TATATTTCTC GAAACAAATG

142981 AAAAGGAAAC GAAAGATGAA ACAGAAATCA TGGATCAACT AAGCCCTCTC GGGGACTTTC

143041 TTAAGAATAA GAAAGAGGAA CCTCATGTAA ATACCATGGA ATAAGGTTTG GTCCTATTCA

143101 TGGAGATTCC GTAAATATTC CATTCTAAAA ATGGAAAGTT CGAAACAATT GGGATTTTTT

143161 TTTTGAAATT GGATGCAGTT ACTAATTCAT GATCTGGCAT GTACAGAATG AAAACTTCAT

143221 TCTCGATTCT ACGAGAATTT TTATGAAAGC CTTTCATTTG CTTCTCTTCG ATGGAAGTTT

143281 GATTTTCCCA GAATGTATCC TAATTTTTGG CCTAATTCTT CTTCTGATGA TCGATTCAAC

143341 CTCTGATCAA AAAGATATAC CTTGGTTATA TTTCATCTCT TCAACAAGTT TAGTAATGAG

143401 CATAACGGCC CTCTTGTTCC GATGGAGAGA AGAACCTATG ATTAGTTTTT CGGGAAATTT

143461 CCAAACGAAC AATTTCAACG AAATCTTTCA ATTTCTTATT TTACTATGTT CAACTCTATG

143521 TATTCCTCTA TCCGTAGAGT ACATCGAATG TACAGAAATG GCTATAACAG AGTTTCTCTT

143581 ATTCGTATTA ACAGCTACTC TAGGAGGAAT GTTTTTATGC GGTGCTAACG ATTTAATAAC

143641 TATCTTTGTA GCTCCAGAAT GTTTCAGTTT ATGCTCCTAC CTATTATCTG GATATACCAA

143701 GAAAGATGTA CGGTCTAATG AGGCTACTAT GAAATATTTA CTCATGGGTG GGGCAAGCTC

143761 TTCTATTCTG GTTCATGGTT TCTCTTGGCT ATATGGTTTA TCCGGGGGAG AGATCGAGCT

143821 TCAAGAAATA GTGAATGGTC TTATCAATAC ACAAATGTAT AACTCCCCAG GAATTTCAAT

143881 TGCGCTCATA TTCATCACTG TAGGAATTGG GTTCAAGCTT TCCCCAGCCC CTTCTCATCA

143941 ATGGACTCCT GACGTATACG AAGGAGTGCG GTTCGTTCGA TAAATTCCTA CCTCTCTATC

144001 TATCTCTGAG ATGTTTGGAT TTTTCAAAAC TCCATGGACA TGCAGAAGAG AAATGCTATC

144061 CACACTCGGA CCAAGACAGA ACTTTTACTT GTTCAAATAA CAATTAAGGC GAAGCAGGGT

144121 CAGGAACGAC GAATCTCTTT ATGATAAACA GATCCATTTT GCAAGTTCGT TATTATGGGT

144181 AGTTCCTACA AAGGATCGGA CCAATGACGT ATACAATACT TGAATTCTCG GTGTAGATGC

144241 TACATAGTTG GTTCTCATCC TTCAGAGACT ACGAGTATAA TAGGAGCATC CGTCGACAAA

144301 AGGATCACCC TAAGATGATC ATCTCATGGC TATTGAGAAC GAATCAAATC AGATGGTTCT

144361 ATTTCTCAAT CTTTCTGATT TGCTCCTCCT ACGGAACCAA GATCGAAAAG ATTGAAAAAA

144421 CAAGTCATTC ACAACCACTG ATGAAGGATT CCTCGAAAAG TTAAGGATTA GTAATCTTTT

144481 TTAGAAATCG AATGGATTCG GTCTTATACA TACGCGAGGA AGGTAATCAA AAAAGAAAGA

144541 AGATGAGTTC TTCTTTCTTT TTTGATCACT TAGGAGCCGT GTGAGATGAA AGTCTCATGC

144601 ACGGTTTTGA ATGAGAGAAA GAAGTGAGGA ATCCTCTTTT CGACTCTGAC TCTCCCACTC

144661 CAGTCGTTGC TTTTCTTTCT GTTACTTCGA AAGTAGCTGC TCCAGCTTCA GCCACTCGAA

144721 TTTTCGATAT TCCTTTTTAT TTCTCATCAA ACGAATGGCA TCTTCTTCTG GAAACACTAG

144781 CTATTCTTAG CATGATATTG GGGAATCTCA TTGCTATTAC TCAAACAAGC ATGAAACGTA

144841 TGCTTGCATA TTCGTCCATA GGTCAAATCG GATATGTAAT TATTGGAATA ATTGTTGGAG

144901 ACTCAAATGA TGGATATGCA AGCATGATAA CTTATATGCT GTTCTATATC TCCATGAATC

144961 TAGGAACTTT TGCTTGCATT GTATTATTTG GTCTACGTAC CGGAACTGAT AACATTCGAG

145021 ATTATGCAGG ATTATATACA AAAGATCCTT TTTTGGCTCT CTCTTTAGCC CTATGTCTCT

145081 TATCCCTAGG AGGTCTTCCT CCACTAGCAG GTTTTTTCGG AAAACTCTAT TTATTCTGGT

145141 GTGGGTGGCA GGCAGGCCTA TATTTCTTGG TTTTAATAGG ACTCCTTACA AGCGTTGTTT

145201 CTATCTACTA TTATCTAAAA ATAATCAAGT TATTAATGAC TGGACGAACC CAAGAAATAA

145261 CCCCTCACGT GCGAAATTAT AGAAGATCTC CTTTCAGATC AAACAATTCC ATCGAATTGA

145321 GTATGATTGT ATGTGTGATA GCATCTACTA TACCAGGAAT CTCAATGAAC CCGATTATTG

145381 CAATTGCTCA GGATACCCTT TTTTAGCTTC TAGGGTCTAT TTCTTAGTTC AAGATCCCTC

145441 TTACTAACTG GAATCAAAGA ATTAGTAGAT CTGTTCCGCC CAAAATGGGA ATGGGCTAGG

145501 GTTATGAACT TATAATCTAT AATCTGATGA TCGAGTCGAT TCCATGATTA TAAGTTCATT

145561 CCATACCGGA CCAGACCGGA ATAGGGTTAT ATACATTCTC ATTATGAGAA GGGGTCATTT

145621 GAGCGTATCT AAATAGATAC TATGTTTACA TATGGATCCC TACGTCGTTA CATTACAGTT

145681 AGGATTAGGA ATAGGCGTAG CAATCGGACC TGCTTTTTAC ATATCTCTCG TTATTTGGGA

145741 CCCTATTCAT CTCTTTGGGC TTCTATTGAA TCGAGAAATG GGTTTGATTG TGTCCATCTT

145801 TTTGATAGAT TGGATATCTA TATAAGGCAT TCTCCGGATA ATTCAAATCG AAACAATTGG

145861 ATATCCAACT CGGGCCTATA TAACATGACC AATCAATAGA AATACTCCAC CCTTGTCATA

145921 TATTCCATAC ATCACACTAG ATAGATATCA TATTCATGGA ATACGATTCA CTTTCAAGAT

145981 GCCTTGGTGG TGAAATGGTA GACACGCGAG ACTCAAAATC TCGTGCTAAA GAGCGTGGAG

146041 GTTCGAGTCC TCTTCAAGGC ATAATATTGA GAATGCTCAT TGAATGGGCA ATTCCATAAG

146101 CTCGGCAGCG GATCACTAAA TCTTGGTGAT CTTCTCTATC TAATGAATGG GGAGTCTGCT

146161 TTAAAATCGT CCGCCCTGCA CCCACCCCCC CGAGTATATG CTTCAACAGT AATCACACAA

146221 GGGTAGATTA GAAACCTCTG GTAAAATGCC CGCCCGTAAC CCAGCAGATA AAGTACATTA

146281 CATAGTCCGT TTTAGGGATT GGCGATTTAC CCATTCAGTG ACTTTGGCAC TGGACGTTCC

146341 CAAAATGGGT ACTGTCGGGT CGGGTGAATT CAATAATAGA CGCCTGGTGG CATTCCAGCC

146401 TTCCTTCGCT TTCAGGGCCT ATCCGAAAGA GAATCCAGTA CTTCTTTAGA GAATCCAGTA

146461 CTTCTTTTCT TGGTCGTGAA TATATGAACT GGTTGTTCGC TGTTCAAGAA TTCTTGTTTA

146521 GGCAGTTCAT ACCATCCATA CATAGTGTTT TGATCTAAGA TTTCAATTCT TCCGTGTTTC

146581 AGCAGCAGCC TATTGTTCCA TGGAGCTAAG GTCCAAAATA GGGAAGAAAC AAGCGTTTCC

146641 GCGACTCTAC CACCCAGTCA ATTCTGTTCC ACTTAATCCC TCTTTCTTTC ATGGCCACAT

146701 ACCTTTCCGC CTAAGGAATG GTAAATCTTT CTCCTGTTCC ATGAATCCAA TTTTCATTTC

146761 ATCCGGGAAA AGCCATCTTT TTCTTAACAA TGTCTTTGTC ATTTGATCCA ACAGCGTTCC

146821 GTTAGATAGG AATAGATTTG ATAAATACTG ATAACTCTCG GATAGAGTAT TAGAACGGAA

146881 AGATCCATTA GATAATGAAC TAGTGGTTCT AAACCCTCTC TGGCGATTAA TCAACAATTC

146941 GAAGTGCTCT TCTTGCGTCT TCTTGATAAA CCAGCGTTTA TATATAGATG TAGGAGGATC

147001 TGTTTGGGAA GTAAGAAGCC CCTTTGACAT CTCTTCATCT GCAAATAATT CTCGATGTGA

147061 AAACACAGAG ACAAAGGGCT GATCTTTGAA TAGGAAAAAG AGTGGATCTG CAGGGTCCCA

147121 AATGAATTGG CTTATTCGAA AAAGGCCTTG TTCTTTGGAA GATCTATCTC GTGTCTGGGA

147181 CTGCATGGTT CCACTCTGCA AGAACTCCGA ATCATTCTCT TGAAGCTCAT CCTCTTCATC

147241 ATAAAGGATC CGCTTGCCCC GAAATGACCT GGACCAATAG GGAAATCCCA ATCCATTGGG

147301 CCTTTCGATA CAATCAAATA GAATGCCCCA AGGGTGCCAT ATTCTAGGAG CCCAAACTAT

147361 GTGATTAAAT AAATCCTCCT CTATCTGTTG CGGGTCGAGG GCCCCTTCCC CTTCTTCAAA

147421 CCCTGATTCG TTTTTTTCAT AGAGAAATCT CTGATCAAGG ATAGAACAAG ATCCGTTTTG

147481 CATCATATCT AGGGGATTCC TTGGTTCGGG CCGAAGAAGC AATGTCACCC GATCATTATC

147541 AAACTGACTG CAATCTCTTT CTGTCCGCGA GGATCCCACC AGAGCGCCTT CTACTTCTAA

147601 TAGGCCATGA ACTAGATCAG AATCATTCTC AACGAGTCCA TAAGAAGTGA TCCCATTTTT

147661 TTCATCGGGT CCGGGTAGAG ACCAAAGATC TTGAGCGACC AATCCGGCAG AACAACTCAA

147721 AAGATAAAGA AGTATCGTTA ATTTCTTCAT GCTCGTTCCA AGTTCGAAGT ACCATTTGTA

147781 CAAATAGGAA TCCCCTTCGT TACATGATTT CTTCTTAATA TAGATAGATA TAGGATCTAT

147841 GGGGCAATTA CTTAGAAGTA CATTTTGTGC TACAGCCCTT CCTATCTGAT AGAAAAGGAT

147901 CCCATGATCC TGAACCGATC TTACCTGGGA TCGCAAATCC CAAGTTTGTC TATGAAGAGC

147961 GGATCTAATT GTATTAGTGT CTATAATTGA TTTCTTCTGT GTAATACTAA TCGATAGGGC

148021 CTCATTGGTA AGTGCTACAA GATCTCGTGC ATTGGAACCC ATGGTTATGG ACCCGAATCC

148081 ATTAGTATGG AACATTTTCT TTTCCAAGCG AAATCCCCTA GTATATGAAA GGGTGAAAAG

148141 GTGCTTTCGT TGTTGTGGAA TAAGAAGCCT TCGTATCTTA ATGCACGTAT TTAATTTATT

148201 CGGAGCTATT AGAGCGGGAT CCACTTTTTG GGGAATATGA GTCGAAGCAA TAACAAGAAT

148261 ATTTCTAGTG GAACATCTTT CACAATCCCT GGAGAGATGG TTCACTAATA GACCGAGGGA

148321 GAAGTCATTC GACTCATTCA CATCCAGATC ATGAATGTTT GGAATCCATA TTATGCAAGG

148381 AGACATTGCT TTTGCTAATT CGAATTGAAG GGTGATAGAA AATTGGTCTT TTTCCGACAT

148441 CATATCCGTA GTTAGCAGTT CCAGCTCCGT ATGAAGGTCA CGATCGATAT CGTCACTAGC

148501 ATCAATATCG TCACTAGCAT CAATATCCTC ACTAGCATCA ATATCGATAT CATCAATATC

148561 GATATCATCA ATATCGATAT CATCACTAAG AAAACCTTTA GACTTGTTAT CCAGGAACTT

148621 GTTCAGAAAT ACCGTAATGA AAGGAACATA GGAGTTTGTC GCTAGATATT TGACCAAATA

148681 GGATCGTCCA GTTCCTATAG AACCTATCAC TAAAATACCC CTAGAGGGGG ATAAGGCTAA

148741 GCGGAGCGAA AAGGGTTTTC CATGAGATGG GAAATGAAAA CTATTAGCCC CACACGAGGT

148801 TTGTGAATAA GTGATTGTCT GATAATGAGC AAGGAATATC CGTCTTTCTG CTAAACAGGA

148861 TGTATTGAAC TCATAATTCA TTAGAGACTT TTTATGAATG TCAACTAAGT ATCGTAAGTA

148921 AATTGCTCCC GGTTGTTCAA TCATTTGATA GCCAGAGTCA TTCTTTGATA AATGATCACT

148981 ATGAGTCAGA CTCAATAGAA TTTGATCAAT CCTTTTTTCT GTCGTTAAGG TGGAGAACTG

149041 AACCAAGAAT TCTCTTTCTT CATCATCAAT CGAATCACTG TTTGCGACCC AGGATTCTAT

149101 TTTATCATCA ATCCAATCCC CGTTCACATT TTTTCTTTTT CTTATCAATG AATAGATCTC

149161 TTTACTTGTA TGACTTAGAT GTCTCGTATT TCTCGAAAAA GTGATTCGAT TGATGGGGTT

149221 TGGTATGATA CTTATGAGAT CGATGAGATT GATATTCAAA TATTTCTTCT TAGAACGTAT

149281 TGATTTGACC CCATAAGCGG GATCACCACC CAATAGCATG TTGCCGCCAG AAGCCGAACC

149341 CCGTATTTCT TCTAGAGAAT CTCCTAATTG TTCTAGAGCA ACTAGAAAGA GATTCTTTAA

149401 CCAGAAAAAA TTCGGTTCAG ATGTAGGATA CCTATCCAGA AGTTTTCGCA ACTCAATCAT

149461 AGATGATGGA ATCATCAAAG ATTTGACCTT TTCAAACTCT GTCTGTAACT CACTAGAGGC

149521 CCGGGAAACA AAGAAAAGAT GTGTACGAAC GAGATATCCA GCAACAAGAA GAAGGAAAAG

149581 GATTGAATAG AGGAACTCCC GAACATTTGG CGATCTCAGA TGTGTCGATA TCAATGGCGA

149641 CTCATTATTT CGATGAATCA TTTCTTCGGA CAGAAGAAGA TTATGTAAAC ACTGACTCGA

149701 AATCTTACGT ATCGGATTCC GTTGTGGAAG ACCCCATTTT TGCTGAAGAA TTCGCCATGC

149761 TATATCTAAT ACATGCATAA TATCATGAAA AATGGATACA AATGTATGAC TGCTACTTAG

149821 TATCGGCAAT CGGTGTATCG GCAATAGGTG TATCGGCAAT AGGTCTGAAA AAGTCTCTAA

149881 AAATATCCAA TTTAGATATT TGTACCCTGT CGAAGTAAGG AACCATGGTA TATATGTTTG

149941 GAATAGATTC CATTTTGAGA GAGTTGAAAA AGCACTATCT CGTTGAAAAG TTCTATACAT

150001 CTGCCCTTTC TCAACGTATT TCTTTAGAAA AAGACTCCGT TTTTTCCTCT TTTCGGATGG

150061 TAAATATTTC TCAGAACATG GAGTGTGAAT CAAACCCATG TTTGAATTGA AATTGAGATA

150121 CTGATGCGAG TTCTTCCCTT CTGAATCAGA TAGATTCAGA TCTGAAAGAG GTTGACAATA

150181 AGTTCTTTCA AAATTGACTA TCTGTCCCTC TGTTAGAGGT GTTCCAGAAA TGTCTGCGAT

150241 CGAGTAAATA GCTCTACGAA CGAATGGATT GGATCGACTT GGAAAATGGG AAGATTTGTA

150301 CAAGTTATAC GTTTCGTCAC CACCACCTTG TGGAAAATCG TTAGGTATGA ATATGTTAGA

150361 TACCTGTGAC TCGATTGGTG AAATAGTATC TCTCCCCCAA AAAGCATGTT TTTTTTTACC

150421 GACGCACAAA GAAAAAATTT TGTTGTGAAT GAACAAGATA TTGAGGAATT GTCCATACGT

150481 AAAATCATCA TTATTGATAC GGGCCTTTTC CACATAAAAA GGGAATCTTT TATTACAATA

150541 GAAGCAGAAG TGATGTGGAT TATTCAAGAA TCGAAGTCGA TTTGCTTTAT AAAAAGAAGA

150601 TATCAATGAA CTTCTATGAA ATGGTTTCAG GGGATTCAGC CAATTGTCTT GATCGTGGAA

150661 TATCGTTGAG AAATAGGAAT CCGTGTTAGC AAAGGATTTC CTACGATTCT TTCTAGTGTG

150721 GAATGAGTCA ATCATCCACT TTGGTATCTT ATTGAACAAA AATGGTGATA TTGTTCCTCC

150781 ATTGATCAAG AATTTAGATT TTTGGGAAGT ATCATGATCA TCCAATAAGA AGGGTTTCCA

150841 TTTTTGCAAA TGAACGATTT GAAGACCTAT TGATTCTAAC AACTGATTGC AGAGTTGATC

150901 ATTCGGACCT TTCAATTCAT AGATGTAGAT CTCGGACCTA TGAATGGGGA TATTCCCGAA

150961 ACTCAAAAAG AAAAAAGGAA GTGAGTTAGA CAAAAAGAAA AGCAACTTGG ACAAAAAGAA

151021 ACGAAATGGC TTAGACAAAC CTTTTTTGTC GATAACCTCA GACCAATCAA TCGAATATTG

151081 ATTAATACGT AATCGATCGA ACACTACTTG AAAACGGCTC TTCTGCTCAG AAACGAAATG

151141 TTCCAAATGT TCCTGGAAAT TATTGCTCCC ATTGGACCAT TTGTATCTAT ATGCATCAGG

151201 ATCCCGATTC ATGGATCTCT CGGTTCGAGA AATCAAAATA AGAGGATCGA ACCATCTCTT

151261 CTGACTCTTT TTCAAATTCG ATAAATGTTG GTTGATCGTA TATTTCATTC TAGTTTTATG

151321 ATTCAGAGTA TCCTTTCCTA TTTGATCCCT TTGAATTCCA TATTCGAAGT TGCGATCGGA

151381 TCTATTCATT AAAAAGAATC GATTCAATAC ATTTCTTATG TACCCATAGG TGCTATATTG

151441 GATTTGAATC AGATTTCGGA TCAATCTATA TTGATTGACT GCCTCCATTA TGTTGTTGCT

151501 AGCAAATACC ACTTTTTTTT GTTTTGGATC TTCCAAATCA TTCCCGCAGG AGATCCGGAC

151561 CCATTTTTTT ATGATCCTTC GATAAAAAGA TTCATTCTCT TCATAAAAAA TAGGAGGTAG

151621 AGCCAATAAA GATTTTTTTT TCGATTCATC CCTGGAGTTG AATACCTCAT TCAAGAATTG

151681 TTTTTGATCC AATCCGTAGG AATCAATAGA AAAGGCAAAT CCCTTATGAT ACACCAGATC

151741 CGGCTCGGTT ATTGATAGAG TGAATAAATC TGCCATTTCT TGAAATCTCT CTTCTGATTC

151801 AAAATCGTGG TGTAACGTGT ATCCTCCCCT CTTCCGGTCA TGGAATAGAT GAAATAAATC

151861 AAGAAATGTA TTTTTGTTCA AGAATGAAAT CTTATTAGAA CTGTCCATAT CCGGTTCATC

151921 CTTCGGAACC ATATCACATC CCGGATATGA TGAAATAGGA TGAATTGAGA CGGTATTTTG

151981 TAAATACGTA ATTATCTTGA ATATATTAAC CATTTCTTTC TTTTCCGACC GCCTCGAAGG

152041 GACAAAAGAA AGATCTTGTT GTTTCTTCAA CCATTTCTGA TCTCTAGTGG ACCTCTCAGT

152101 AGGATTCGAA CCCAGATGAA GTTCTGACCA TCTGTCAGAG AAAAAAGAAC GAACGGATCT

152161 TGTAGGATTC CCAAGAAATT CTTCGATTTC TTCCGGAAGC AGATGATTAA TCATCTGCTT

152221 CTCACGTTCC GTGAATAGCC GGGACATTGA GGAATATCCA GAAAGGCTTT TCGGGAATCG

152281 GTCTGATTCT ATCTCTGTTC GTTCCGTTTG AAGAAAGGAA GGATCCCAAA GAATCGATCT

152341 TTCTTTTAGT TGTTGAATCT CTCTTTGATT GATCAATGTG TGATATTCCG AATCCTCATT

152401 ACTAAGGGAA TCCAAAGGAT CTCTGGATTG ATCAGAAGAT CCTTTCAATT GGCTAGAATC

152461 CCTCTTTTTT CCGATCCAGT TCCTCCACCA CCGCGAACCC CAGTTAGATT CAGGCATGCT

152521 ACACTTTTTA GTTATTGGGA GAACCCAAGT ACTCTCTTTC GGATTCAGGA AACAACTCTC

152581 AGAGATCTTT TTTCCTTTTG GAAGATACAG GAGGGAAACA ATCAACCTAT TGATATTGGA

152641 AGACCCAACG GATTCTTCCA ATGTATCATT TCTGGGTCCA ATGGAATTCA TAGGTATAGG

152701 AAGAAGCCCT ATCAAATAGA AATTTTGTCT TTCGACCCTA TTTCGACTGT TAATACGATA

152761 TATAAGGACC GCTACTACAA AGAGTATTAC ACCCTTGATC GTGAAATATC GATTGCTTGC

152821 TGAACCCTGT GAATTGCGTA AAAGTAGGAT ACTCCAAATT CGGGGGTCAA AAAGTTTTAT

152881 AAAACGTTCT TGGTGGAAAA AAATGTGAAT GAAAGATCCC ACTGAATTGA ATTGGGTCCA

152941 TGAATCTAAG AAATAGTGAG AATTCTTGAT CTCTCTCAAT ATCTCTCTCA ATTCGAAAAT

153001 CCAGGATTTG AATTGGTGTC CTTTCATTGA TTCCTCCTAA ATTGCATTGA TTTATTCTAA

153061 AGATTTCGTT TCAATTGGAA TTTGGTTATT CACCATGTAC GAGGATCCCC GCTAAGCATC

153121 CATGGCTGAA TGGTTAAAGC GCCCAACTCA TAATTGGCGA ATTCGTAGGT TCAATTCCTA

153181 CTGGATGCAC GCCAATGGGA CCCTCCAATA AGTCTATTGG AATTGGCTCT GTATCAATGG

153241 AATCTCATCA TCCATACATA ACGAATTAGT GTGGTATATT CATATCATAT ATTCATATCA

153301 TAATAATATA TGAACAGTAA GAACTAGCAT TCTTATTGAG ACTAGAACTC ATAGGGAAGA

153361 AAATATAGTT ATGGATGGAA TCAAATATGC AGTATTTACA GACAAAAGTA TTCGGTTATT

153421 GGGGAAAAAT CAATATACTT CTAATGTCGA ATCAGGATCA ACTAGGACAG AAATAAAGCA

153481 TTGGGTCGAA CTCTTCTTTG GTGTCAAGGT AATAGCTATG AATAGTCATC GACTTCCGGG

153541 AAAGGGTAGA AGAATGGGAC CTATTATGGG ACAGACAATG CATTACAGAC GTATGATCAT

153601 TACGCTTCAA CCGGGTTATT CTATTCCACC TCTTAGAAAG AAAAGAACTT AAATCAAAAT

153661 ACTTAATAGC ATGGCGATAC ATTTATACAA AACTTCTACC CCGAGCACAC GCAATCGAAC

153721 CGTAGACAGT CAAGTGAAAT CCAATCCACG AACTAATTTG ATCTATGGAC AGCATCGTTG

153781 TGGGAAAGGT CGTAATGCCA GAGGAATCAT TACCGCAGGG CATAGAGGGG GAGGTCATAA

153841 GCGTCTATAC CGTAAAATCG ATTTTCGGCG GAATGAAAAA GACATATATG GTAGAATCGT

153901 AACCATAGAA TACGACCCTA ATCGAAATGC ATACATTTGT CTCATACACT ATGGGGATGG

153961 TGAGAAGAGA TATATTTTAC ATCCCAGAGG GGCTATAATT GGAGATACCA TTGTTTCTGG

154021 TACAGAAGTT CCTATAAAAA TGGGAAATGC CCTACCTTTG AGTGCGGTTT GAACTATAGA

154081 TTTACGTAAT TGGAAGTAAC CAATTAGGTT TACGACGAAA CCTATAAATC GATCACTGAT

154141 CCAATTTGAG TACCTCTACA GGATAGACCT CAACAGAAAA CTGAAGAGTA ACGGCAGCAA

154201 GTGATTGAGT TCAGTAGTTC CTCATATAAA ATTATTGACT CTAGAGATAT AGTAATATGG

154261 AGAAGACAAA ATTGTTTCAA GCACCGACAG AACCGGAAGC GCCCCTTCTT TCAAAAAGAA

154321 GAGGACGGGT TATTCACATT TCATTTGATG GTCAGAGGCG AATTGAAAGC TAAGCAGTGG

154381 TAATTCTAAA GATTCCCCGG GGGAAAATAG AGATGTCTCC TACGTTACCC ATAATATGTG

154441 GAAGTATCAA CGTAATTTCA TAGAGTCATT CGGTCTGAAT GCTACATGAA GAACATAAGC

154501 CAGATGACGG AACGGGAAGA CCTAGGATGT AGAAGATCAT AACATGAGTG ATTCGGCGGA

154561 TTTGGATTCC TATATATCCA TGTGGTACTT CATTGTAGGA TATATAAGAT CCATCTGTAT

154621 AGATATCATC TACATCCAGA AAGCCGTATG CTTTGGAAGA AGCTTGTACA GTTTGGGAAG

154681 GGGTTTTGAT TGATCAAAAA GAAGAATCTA CTTCAACCGA TATGCCCTTA GGCACGGCCA

154741 TACATAACAT AGAAATCACA CGTGGAAGGG GTGGACAATT AGCTAGAGCA GCGGGTGCTG

154801 TAGCGAAACT GATTGCAAAA GAGGGGAAAT CGGCCACATT AAAATTACCT TCTGGAGAGG

154861 TCCGTTTGAT ATCCAAAAAC TGCTCAGCAA CGGTCGGACA AGTGGGGAAT GTTGGGGTGA

154921 ACCAGAAAAG TTTGGGTAGA GCCGGATCTA AGCGTTGGCT AGGTAAGCGT CCTGTAGTAA

154981 GAGGAGTAGT TATGAACCCT GTAGACCATC CCCATGGGGG TGGTGAAGGA AGGGCCCCAA

155041 TTGGGAGAAA AAAACCCACA ACCCCTTGGG GTTATCCTGC ACTTGGGAGA AGAAGTAGAA

155101 AAAGGAATAA ATATAGTGAT AATTTGATTC TTCGTCGCCG TAGTAAATAG GAGAGAAAAT

155161 AGAATTTATT TCTTCGTCTT TAAAaAAAAA AAAAAAAAAA AATAGGAGTA AGCTGTGACA

155221 CGTTCACTAA AAAAAAATCC TTTTGTAGCG AATCGTTTAT TAAGAAAAAT TG

//
